# Whole CMV Proteome Pattern Recognition Analysis after HSCT Identifies Unique Epitope Targets Associated with the CMV Status

**DOI:** 10.1371/journal.pone.0089648

**Published:** 2014-04-16

**Authors:** Lena Pérez-Bercoff, Davide Valentini, Simani Gaseitsiwe, Shahnaz Mahdavifar, Mike Schutkowski, Thomas Poiret, Åsa Pérez-Bercoff, Per Ljungman, Markus J. Maeurer

**Affiliations:** 1 Department of Medicine Huddinge, Karolinska Institutet; Dept. of Hematology, Karolinska University Hospital, Stockholm, Sweden; 2 CAST (Center for allogeneic stem cell transplantation), Karolinska Hospital; 3 Microbiology, Tumor and Cell Biology (MTC), Stockholm, Sweden; 4 The Swedish Institute for Infectious Disease Control (SMI), Stockholm, Sweden; 5 Department of Enzymology, Institute for Biochemistry and Biotechnology, Martin Luther University Halle-Wittenberg, Halle, Germany; 6 Division of Therapeutic Immunology (TIM), LabMed Karolinska Institutet, Stockholm, Sweden; 7 Department of Genome Biology, John Curtin School of Medical Research, The Australian National University, Canberra, Australia; Karolinska Institutet, Sweden

## Abstract

Cytomegalovirus (CMV) infection represents a vital complication after Hematopoietic Stem Cell Transplantation (HSCT). We screened the entire CMV proteome to visualize the humoral target epitope-focus profile in serum after HSCT. IgG profiling from four patient groups (donor and/or recipient +/− for CMV) was performed at 6, 12 and 24 months after HSCT using microarray slides containing 17174 of 15mer-peptides overlapping by 4 aa covering 214 proteins from CMV. Data were analyzed using maSigPro, PAM and the ‘exclusive recognition analysis (ERA)’ to identify unique CMV epitope responses for each patient group. The ‘exclusive recognition analysis’ of serum epitope patterns segregated best 12 months after HSCT for the D+/R+ group (versus D−/R−). Epitopes were derived from UL123 (IE1), UL99 (pp28), UL32 (pp150), this changed at 24 months to 2 strongly recognized peptides provided from UL123 and UL100. Strongly (IgG) recognized CMV targets elicited also robust cytokine production in T-cells from patients after HSCT defined by intracellular cytokine staining (IL-2, TNF, IFN and IL-17). High-content peptide microarrays allow epitope profiling of entire viral proteomes; this approach can be useful to map relevant targets for diagnostics and therapy in patients with well defined clinical endpoints. Peptide microarray analysis visualizes the breadth of B-cell immune reconstitution after HSCT and provides a useful tool to gauge immune reconstitution.

## Introduction

Patients after hematopoietic stem cell transplantation (HSCT) remain at increased risk to cytomegalovirus (CMV) disease despite advances in clinical management [1]; a similar situation is true for patients after solid organ transplantation [2]. Protective immune responses directed against CMV are predominantly mediated by CD8+ T-cells targeting either CMVpp65 or immediate early (IE)-1 proteins [3]. The importance of specific antibodies (Abs) as part of the immune protection against CMV has been controversial in the stem cell transplant setting [4]–[7], yet anti-CMV directed serum antibodies may be clinically relevant in the post-transplant setting in the absence of antibody producing B-cells due to the half-life of serum IgG of 40–60 days. CMV targets recognized by serum IgG include surface-exposed virion glycoproteins, e.g. glycoproteins B (gB), gH, and gM/gN [8]–[10]. HSCT recipients frequently lose specific antibodies after HSCT [11]–[13] and the functional recovery of B-lymphocytes after HSCT may take up to 2 years [14], [15].

The aim of this study was to map the CMV epitope IgG recognition pattern in an unbiased way to answer unmet clinical needs: i) target proteome mapping is currently being performed to decipher biologically relevant epitopes in CMV vaccine development, i.e. prevention of maternal cytomegalovirus infection [16]. Identification of biologically relevant CMV epitopes may aid to develop improved strategies to boost anti-CMV directed immune responses in CMV-discordant transplant situations. ii) Post-HSCT vaccination CMV-strategies lack epitope recognition patterns which would help to differentiate between already existing anti-CMV humoral responses and new CMV epitope recognition patterns associated with CMV infection(s) or CMV vaccines. iii) CMV− epitope mapping may help to decipher the quality of immune responses in CMV-discordant transplant recipients; iv) Mapping anti-CMV humoral reactivity will aid to reflect the breadth of B-cell immune-reconstitution in transplant recipients and possibly perturbations in the B-cell compartment associated with graft-versus-host-disease (GVHD) [17].

CMV – recognition mapping could be performed in different ways, e.g. with a selected set of target CMV proteins or alternatively, with a more comprehensive ‘omics’ approach which enables an unbiased view of humoral immune reactivity [18] including peptide microarray platforms. Such unbiased approaches helped to successfully decipher antibody signatures in infectious disease, e.g. in the development of yellow fever vaccination [19] and a system biology approach was instrumental to map protective immune responses in seasonal influenza [20].

We took advantage of peptide-microarray technology to gauge the global anti-CMV epitope recognition pattern in order to understand i) when the humoral immune response against CMV is formed in a post-transplant setting ii) if the CMV status impacts on the epitope focus based on the CMV status of the donor/recipient at the time of transplantation and iii) whether most of the CMV epitope specific IgG responses are common or ‘private’ for each individual.

## Materials and Methods

### Patient samples and peptide microarray slide preparation

The Stockholm regional ethical review board approved the study (Ref 2007/735-31/1). Each patient agreed to the study and signed the informed consent form, which is on file at the Dept. of Hematology (Prof Ljungman), Karolinska University Hospital, Stockholm, Sweden. 54 plasma samples were selected from 18 HSCT-patients (pat A to T); the patients had not received intravenous immunoglobulin infusions; clinical information is provided in Table S1. We recruited additional patients, designated as P1–7, also listed in Table S1, from whom we had sufficient matching peripheral blood mononuclear cells (PBMCs) available to test for T-cell reactivity against CMV peptide targets identified by antibody reactivity. All patients received conventional myeloablative conditioning, i.e. cyclophosphamide (Cy) at 60 mg/kg for two days in combination with fractionated TBI (FTBI) at 3 Gy/day for four days (n = 15), or busulphan (Bu) at 4 mg/kg/day for four days; RIC (reduced-intensity conditioning) was provided to 6 patients [21]. Immunosuppressive treatment for GVHD prophylaxis consisted of cyclosporine (CsA) in combination with a short course of methotrexate (MTX). Patients with an unrelated donor or a non-malignant disease received anti-thymocyte-globuline (ATG, Thymoglobulin, Genzyme, MA, USA for 2–4 days during conditioning [22]. During the first month, blood CsA levels were kept at 100 ng/mL in patients with malignancies when a sibling donor was used and at 200–300 ng/mL when an unrelated donor was used. Higher CsA levels were also used for patients with non-malignant disorders regardless of the donor type. In the absence of GVHD, CsA was discontinued after three to six months for patients with malignancies and after 12–24 months for patients with non-malignant disorders. *In vitro* T-cell depletion was not used and no patient received anti-CD20 (rituximab) treatment. Patients were monitored weekly in peripheral blood for HCMV DNA with a real-time polymerase chain reaction (PCR) [23], [24] for the first hundred days after HSCT.

Serum samples from CMV-negative recipients with CMV-negative donors (D−R−) were used as negative controls and compared against the other groups (D+R+, D+R− and D−R+); each consisting of samples from 5 patients, except for the D+R− group (four patients). Serum samples from each patient were selected from three different time points, i.e. +6, +12 and +24 months after HSCT. Peptide microarray slides were incubated with serum and antibody binding to individual peptides was identified by a secondary reagent as described earlier [25], [26].

**Figure 1:**
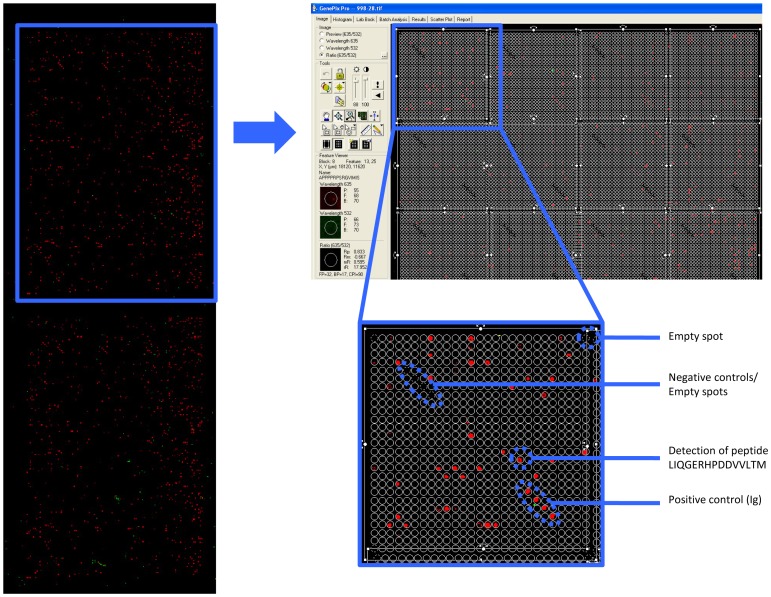
Overview of HCMV peptide microarray chip analysis. The microarray chips (left) consists of two identical subarrays, each with 17496 spots arranged in 24 blocks of 729 spots arranged in columns and rows of 27. The peptide spots represented 17174 unique peptides, 305 positive control spots (4 repetitions each of IgG, IgA, IgM, and IgE) and 268 negative controls. Screenshot (right, top) and closeup (right, bottom) of empty spots, positive spots and a positive response, detecting an serum Ab-peptide complex.

Slides with overlapping peptides covering the whole proteome of HCMV (see Table S2) were manufactured by JPT, Berlin Germany. The slides consisted of two identical subarrays, each with 17496 spots arranged in 24 blocks of 729 spots arranged in columns and rows of 27. The peptide spots represented 17174 unique peptides, 305 control spots (4 repetitions each of IgG, IgA, IgM, and IgE), 268 negative controls and 31 other control spots. All slides belonged to the same batch (a representative example is provided in **Fig. 1**; CMV proteins with their accession numbers are listed in the Table S2). The choice of CMV targets printed on the chip was of critical importance in gauging the diversity of the humoral recognition pattern. Chee and coworkers [27] estimated that AD169 has 208 ORFs of which several are repeated (TRL1–14 and IRL 1–14). Therefore, we choose only TRL 1–14 and not IRL 1–14. Later studies from. Murphy and coworkers [28], examining sequences of clinical HCMV strains, showed that the number of protein-coding ORFs range from 165 ORFs, that are conserved between clinical CMV isolates, up to 252 potentially functional ORFs. Note, that an ORF was considered a coding ORF if it encoded a polypeptide of 100 amino acids or more and did not overlap a larger ORF across more than 60% of its length.) We choose 214 ORFs in the current CMV chip layout, All ORFs from AD169 were selected plus 19 additional ORFs from the Toledo strain (discovered to be missing in the AD169 genome) and 3 additional ORFs from the Town strain. Therefore, there was no premeditated choice of ORFs. We used the identical ‘template’, i.e. ORFs from AD169 in order to visualize differences in B-cell epitope recognition between different groups of patients, based on their CMV donor/recipient serotype.

### Slide scanning and analysis

Slides were scanned at two wavelengths: 532 and 635 nm with a GenePix 4000B microarray scanner (Axon Instruments) and images were saved in .tiff and .jpg formats. GenePix Pro 6.0 software and GenePix Array List (.gal) files were used to analyze images with circular feature alignment using the following criteria to flag spots with non-uniform foreground or background signal for IgG detection:
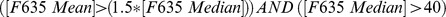
OR
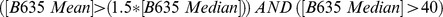
F designates the ‘foreground’ and B the ‘background’ fluorescence intensity measured at 635 nm wavelength. In addition, GenePix categorized spots as “empty”, “not found”, “bad” or “good”. Each sub-array was converted to a digitized image and saved as a .gpr (GenePix Result) file. To analyze the IgG responses, the median fore- and background intensities for the 635 nm wavelength from individual spots were used. All .gpr files were saved in a common folder and imported into R software for subsequent analyses.

Data quality assessment, data reduction, identification and removal of false positive peptide responses from slides were performed as previously reported [25]. Data for each of the five groups of slides (IgG responses from the four CMV serological transplant combinations and IgG from six control slides, i.e. buffer and secondary antibody for IgG detection, without clinical samples) were arranged in a matrix with identifiers for slide, subarray, and block, and these master datasets were used in all analyses described below.

### Statistical analysis

After pre-processing and normalization of peptide responses (as described [25]), we used three different statistical methods: (i) PAM (Prediction Analysis for Microarrays) [29], a predictive analysis which performs sample classification from peptide recognition data providing a list of significant peptides whose response level characterizes each diagnostic class. Compared to other differential recognition analysis methods, PAM is highly selective and allows examining in detail each time point of consecutive serum testing. This reveals only the peptide target with good predictive power associated with the differentiation of the patient groups; (ii) ‘exclusive recognition analysis’ (ERA) of epitopes predicted by PAM. The latter approach identifies epitopes recognized in serum from individuals exclusively in one group but *never in serum from any individual* in a control group, i.e. in the current report the ‘reference’ D−R− patient group (termed ‘exclusive’ epitopes). The peptide recognition pattern in this D−R− ‘reference’ group is listed in the Table S3A/B and the serum recognition pattern concerning commonly shared and ‘private’ epitopes are shown in Figure S1.

Finally, (iii) MaSigPro (Microarray Significant Profiles) [30] was used to follow the dynamic changes in the peptide recognition pattern over time comparing different (patient) groups. This method identifies peptides with significant temporal recognition changes and significant differences between experimental groups via a two-step regression strategy. By using the coefficients obtained in the regression model, significant peptides with similar recognition patterns are then clustered together to visualize the results (k-means clustering, Ward's method).

The advantages of using both PAM and MaSigPro analysis is the ability to identify CMV epitopes which would differentiate the patients groups (defined by their CMV status, based on donor and recipient serology) at each time point with statistical robustness. Furthermore, the use of PAM and MaSigPro allows to follow dynamic changes of serum antibody reactivity over time reflecting the evolution of the transplanted donor immune system in the host. This is usually performed using a selection of different ELISA-based target assays, yet the humoral recognition pattern using peptide microarray technology allows to appreciate serum reactivity to thousands of epitopes simultaneously.

In order to define a peptide response as detectable or non-detectable, we calculated a cutoff value based on the mean plus 2 standard deviation units of the normalized negative controls (0.2947). The use of this detection limit enables to determine which set of peptides is mutually excluded between each pair of the comparison groups in the ‘exclusive’ peptide analysis. If a peptide response is above the detection limit for all peptide responses in one (test) group, but below the detection limit in the reference group, the peptide is considered to be exclusively detectable in the test group.

The gal file (GenePix Array List) was used to identify the original protein origin of the identified peptides. The peptide responses were ranked by i) the strength of their response and ii) the number of replicates (x/n) in which the peptides were recognized by serum antibody binding. We classified the CMV peptides according to their function of the protein from which they derived (c = capsid, m = matrix or tegument, e = envelope, gp = glycoprotein, r = DNA or regulatory, o = other, unk = unknown).

GenBank files of the complete human herpesviruses (HHV) genomes were downloaded from www.ncbi.nlm.nih.gov/nuccore/: Herpes Simplex virus-1 (HSV-1 or HHV-1, accession number: AB618031.1), Herpes Simplex virus-2 (HSV-2 or HHV-2, accession number: NC_001798.1), Varicella-Zoster virus (VZV or HHV-3, accession number: NC_001348.1), Epstein-Barr virus (EBV or HHV-4, accession number: NC_009334.1), HHV-6A (accession number: NC_001664.2), HHV-6B (accession number: AB021506.1), HHV-7 (accession number: NC_001716.2) and HHV-8 (accession number: AF148805.2). Using a custom made script, incorporating PyCogent [31], translated regions from the GenBank files were extracted and one FASTA file was created for each herpesvirus as a reference database to create eight herpesvirus protein databases. A data file was created containing a list of i) all CMV peptides found in the top layer of the ‘exclusive’ peptide analysis (with the peptide being recognized in 4 or 5 patients in one group but never in the D−R− group), ii) peptides found in the PAM analysis and iii) all peptides intersected in both PAM and the ‘exclusive recognition analysis’. This list of CMV peptides was searched and compared against herpesvirus protein databases via the *BLASTP* program (http://blast.ncbi.nlm.nih.gov/Blast.cgi?PAGE=Proteins).

### CMV specific antibody detection by RecomBlot and ELISA

Qualitative *in vitro* testing for detection and identification of IgG antibodies against CMV were performed using a commercially available Western Blot (RecomBlot CMV IgG test, Mikrogen, Neuried, Germany). Serum antibody responses in serum samples from patient A–D in the D−R− group were compared to serum reactivity from patients in the D+R+ group before and +6 months after HSCT. CMV antibody responses in sera from seven additional HSCT-patients (pat 1–7) were also mapped at +6 months post-HSCT since we had sufficient numbers of PBMCs (from the identical time point) available from these patients to be tested for CMV peptide target reactivity by cytokine production analysis). Both patient groups (A to T and 1–7) were tested with the identical batch of CMV high content peptide microarray slides. IgG responses were additionally tested using a commercially available ELISA (Platelia) CMV IgG, from BioRad, Mames-la-Coquette, France. The output of this ELISA are anti-CMV antibody titers; a titer of <0,25 AU/mL is negative (absence of acquired immunity, recent infection cannot be excluded), the result is determined to be ‘equivocal’ with a titer of 0,25–0,5 AU/mL (suggesting a recent infection of the patient; requires re-testing in 2 weeks) and the titer of >0,5 AU/mL reflects a positive result, i.e. a past CMV-infection. This does not exclude a recent infection and an anti-CMV IgM ELISA may be required in the latter case.

### Intracellular cytokine staining

Cytokine production by intracellular cytokine staining (ICS) was analyzed in PBMCs from 5 patients harvested at 6 months after HSCT as described by Magalhaes *et al* 2010 [32]. Cells were stimulated with peptide mixes from CMV, covering the entire protein UL94 (P168000), CMV UL55 glycoprotein B (P06473), CMV UL99 p28 (P13200) and CMVpp65, all purchased from JPT Peptide Technologies, GmbH, Berlin, Germany. Medium and PMA/ionomycin (1 mg/ml) from Sigma-Aldrich were used as negative and positive controls, respectively. For quality reasons, all PBMC samples were run twice. The following antibodies were used to gauge intracellular cytokine production: PE-conjugated anti-IL-2 (MQ1-17H12), PE-Cy7-conjugated anti-IFN-γ (B27), and APC-conjugated anti-TNF-α, all obtained from BD Biosciences, the PE-conjugated anti-IL-17 antibody (eBio64DEC17) was purchased from eBioscience (San Diego, CA). Cells were analyzed using a Gallios Beckman Coulter flow cytometer and data analysis was performed using Kaluza software.

## Results

### CMV infection

Serum samples from patients after HSCT (Table S1), sampled weekly from the time of hematopoietic stem cell infusion (time point 0) until day 100 were tested for CMV DNA. Four out of five individuals in the D−R+ group, one out of five in the D+R− group and in three out of five individuals in the D+R+ group tested positive for CMV DNA at a single time-point within the 100 day observation period after HSCT. In the D−R− group, one patient developed a primary CMV infection at 8 months after HSCT and was therefore removed from the analysis (since the D−R− served as the ‘control cohort’) No patient developed CMV disease and no pre-emptive antiviral therapy was provided since the viral load was below the intervention limit. ELISA-based analysis of serum samples to gauge humoral IgG immune responses (at 6, 12 and 24 months after HSCT) revealed that samples from all recipients in the D+/R+, as well as in the D−/R+ group tested positive (for all three time points after HSCT) for CMV. Serum samples from the D−/R− (reference) group tested negative for all time points after HSCT (except for a single patient that experienced CMV infection at 12 month after HSCT, the serum samples from this individual was therefore removed from the reference group, see above). Serum samples from the D+/R− group showed a different CMV reactivity pattern after HSCT: samples from 2 individuals tested negative for CMV IgG (at all time points, i.e. 6, 12 and 24 months after HSCT) and serum samples from 2 individuals (who tested previously negative at the time of HSCT) tested positive at 6, 12 and 24 months after HSCT. We could not test serum samples from the fifth patient in this cohort, since there was no biological material available.

### Chimerism analysis

PCR amplification of variable number of tandem repeats was used to evaluate donor/recipient chimerism in CD3+, CD19+ and CD33+ cells [33]. All patients were complete donor chimeras for CD19+ B-cells at 6 months.

### Differential CMV peptide recognition defined by the CMV status of the recipient

**Figure 2:**
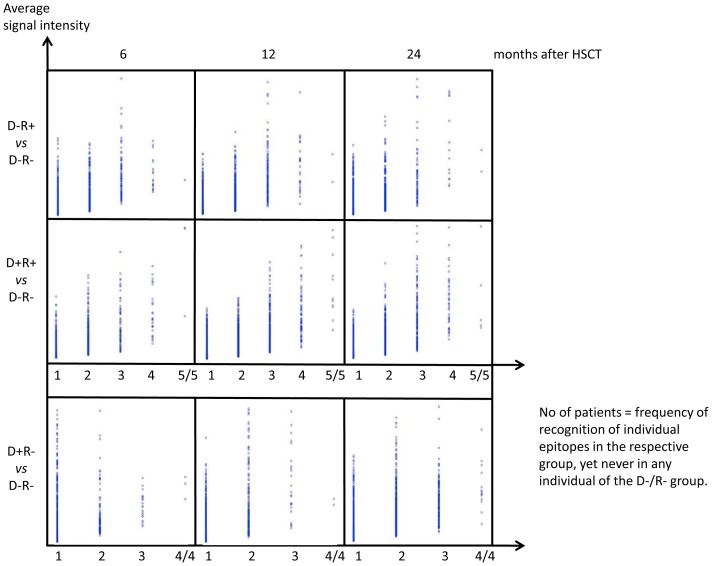
Differential CMV peptide recognition after HSCT segregates with the CMV status of the donor and recipient. Comparisons between serum reactivity in the D+ and D− groups and R+ and R− groups reflecting the number of CMV epitopes predicted by the ‘exclusive recognition analysis. Some peptides are uniquely recognized in serum from all individuals in each test group (but never in the control group, D−/R−). The list of epitopes is provided in Table 1.

Serum samples were tested for CMV peptide reactivity (**Fig. 1**) and several peptide groups could be identified: i) peptides only recognized in serum from some individuals and CMV peptides commonly recognized in serum from individuals in each group (based on donor/recipient CMV reactivity defined by the time of HSCT, see Figure S1). Strongly recognized CMV peptide epitopes (based on fluorescence intensity) were commonly recognized in each patient group, i.e. in serum from each individual patient in the respective donor/recipient group based on the CMV status at the time of transplantation. ii) We identified CMV peptides recognized in serum from all individuals in the respective patient-group, but never in serum from any individual in the (reference) D−R− group (**Fig. 2** and Table 1).

**Table 1:** Epitopes predicted by the Exclusive RECOGNITION analysis.

EPITOPE	HCMV ORF	EPITOPE	HCMV ORF
**6 months after HSCT**		KRCLVPEVFCTRDLA	UL29
*Serological group D−R+*		LRDLGHRVQTYCEDL	UL77
TRVFFSPCAPHVAFI	J1I	PVGSMYRGSDALPAG	US34
HNVTREVNVRKRAYL	UL56	PRNVMTHEEAESRLY	UL36
LFATTLFIGYMPIHC	UL125	GGDWADSASDFDADC	TRL 1
PLLAYAYFRSVFYVI	UL57	GGGGGLDRNSGNYFN	UL44 (pp50)
SACTWTSCTSLSPCS	US20	MELDSVEEEDDFGAS	UL45
WSFGMLFFIWAMFTT	UL100	VALFPSSPPSLKDSC	UL21
RVSAFVAYAVARNRR	UL70	FDDYGNTKSYLGAYT	TRL 3
CFLRTCLRLVTPVGF	UL102	DDAPPTYEQAMGLCP	UL42 rev
GTTGSYTPPQDGSFP	UL139 Toledo	TRFQGPDSMPSTSYG	UL35
ITTYNEYEILNYFDN	UL1	KEKYEQHKITSYLTS	UL44 (pp50)
TPCPNGTYVSGLYNC	UL144 Toledo	DESGRPRRIANRIGD	US22
ARVFCLSADWIRFLS	UL114	IRSSLILYATETLIY	US14
DKLIAWMTWLSSRAT	US17	TTPPMIDLTSHHRPL	UL117
YLPKDAFFSLLGASR	UL80	LRLLACPDRPIIGDT	UL25
FICRDNCTLSDQFTL	US16	AVVWGNARLDALMSA	UL45
RRITRPRQIPLCTGV	UL49	SMSLGARDAELYHLP	UL104
TWTLFVACNGVAWEH	US14	HDDGPGLDNDLMNEP	UL44 (pp50)
TLQHMSKKQESIATI	UL143 Toledo	ELPHTASLRALAGCM	UL23
DLLREVQRNLTRTMA	US17	AGGAAAGPRPPPPPM	UL50
		SYPASYGAPVVGYDQ	UL80
*Serological group D+R−*		SVEEEDDFGASLCKV	UL45
GEDDVLATIRNTLSA	UL150 Toledo	NLSLPPSNALSSKDY	UL 9
CFLRTCLRLVTPVGF	UL102	KAVLGLNAACAVYDH	UL49
YCDLLRVGYFGHLNI	UL43 rev	LLIQDGMYGRGEKEL	UL121
		LSDVTQRRNRPLRCL	UL35
*Serological group D+R+*		EREEDTLREMALKAF	UL25
SQKPVLGKRVATPHA	UL32 (pp150)	LERRSHEELVLCPPE	UL88
TTSTSQKPVLGKRVA	UL32 (pp150)	SSTEGNWSVTNLTES	UL20
RACRPFDHMPAADFR	US22	GMSLNQSTRDISYMG	UL100
SLKPTLGGKAVVGRP	UL32 (pp150)	KVPEDSEPQCNPLLP	UL18
HRANETIYNTTLKYG	UL55 (gB)	LAQFRGTMDDDEAAL	UL31
EVHDALLFHYEHGLG	UL77	DTLREMALKAFMEAN	UL25
VLSHHDSLESRRLRE	UL100	DFMRDFTQLLESCDI	UL52
STFTTVYSTFNTSYA	TRL12	SKATRRTSPRYYPPS	IRL14
HDSLESRRLREEEDD	UL100	GLAWTRQQNQWKEPD	UL83 (pp65)
APPSPVKGRGSRVGV	UL32 (pp150)	AADYLCCDDTLEAVG	US22
AFLHYFTTLKQYLRN	UL18	SVGVNSKVRACVIGY	UL105
CHTETTIIRFKETNT	TRL12	IRYIPATQGDVYHGR	UL 3
TTETNMTTARESSVH	UL132	SMHCRSRHQRTPPSA	UL150 Toledo
KLPYSITVTYDHRTS	UL107	IPNDVSESFERYKEL	UL53
AFRFTPANTTTNSST	UL20	YDDESWRPLSTVDDH	IRS1
CFLRTCLRLVTPVGF	UL102	SGNAYNHTIDTCKNT	UL20
GRASVVFVHHVVKYS	UL45	IPNRIRYIPATQGDV	UL 3
VERLLATSDGLYLYN	UL97	NHGAGGTAAVSYQGA	UL54
ITTYNEYEILNYFDN	UL 1	EDFAHQCLQAAKKRP	US 6
EEAVNLLDDTDDSGG	UL132	SVPVSQRMEHGQEET	UL105
NVTFRGLQNKTEDFL	UL 4 (gp48)	EDEEGGEKGGDDPGR	UL52
DTVLLMHFFYTHYRS	UL46	EGGWGGEEGEDDVLA	UL150 Toledo
YCDLLRVGYFGHLNI	UL43 rev	ALCFCLLCEAVETNA	UL116
RVSAFVAYAVARNRR	UL70		
VGFDRVPQYDFLISA	UL45	**24 months after HSCT**	
SQDHVQIVYGSTRIC	UL117	*Serological group D−R+*	
ARVFCLSADWIRFLS	UL114	LENVTVYPTYDCVLS	UL77
TWTLFVACNGVAWEH	US14	PPLPGHARRPRRKRC	UL29
SACTWTSCTSLSPCS	US20	PGEPLKDALGRQVSL	UL99 (pp28)
NRKASGTGVAAVGAY	UL89	TPEQSTPSRIRKAKL	UL32 (pp150)
		LKDALGRQVSLRSYD	UL99 (pp28)
**12 months after HSCT**		SQKPVLGKRVATPHA	UL32 (pp150)
*Serological group D−R+*		FGTTPGEPLKDALGR	UL99 (pp28)
YLHFSAYKLLKKIQS	UL35	VGVPSLKPTLGGKAV	UL32 (pp150)
IPNDVSESFERYKEL	UL53	NPANWPRERAWALKN	UL32 (pp150)
AQLDLEADPTAREGE	UL35	PRHTFDMDMMEMPAT	UL42 rev
DEKNIFTPIKKPGTS	UL32 (pp150)	TSPNALLPEWMDAVH	UL136 Toledo
RRDSAWDVRPLTETR	UL32 (pp150)	FDMDMMEMPATMHPT	UL42 rev
IFTPIKKPGTSGKGA	UL32 (pp150)	WSFGMLFFIWAMFTT	UL100
TPEQSTPSRIRKAKL	UL32 (pp150)	AFIRRRRPPHHTQLV	J1I
SQKPVLGKRVATPHA	UL32 (pp150)	IFTEHVLGFELVPPS	UL115
AKRKMDPDNPDEGPS	UL122 (IE2)	HYLMYSHTNNECVGE	US27
LSDVTQRRNRPLRCL	UL35		
TTTAELTTEFDYDED	US28	*Serological group D+R−*	
QKKISQRPPTPGTKK	UL99 (pp28)	GMRAVSQFLVTHPLG	US33
WLNFRVDLFGDEHRR	UL35	FSGNGVERSLNVSSM	UL57
VRKLMKRGARLRHDS	UL38	AFIRRRRPPHHTQLV	J1I
CQEYLHPFGFVEGPG	UL36	KCRITEPITMLGAYS	TRL10
EFDYDEDATPCVFTD	US28	NFEAVLARGMHVEAG	UL93
VTFEFVPNTKKQKCG	UL44 (pp50)	HLQLRHALELQMMQD	UL35
ERRIREGKIPMTFVD	UL38	TNQYLIKGISYPVST	UL75 (gH)
RTRVSLGHRVAFGCS	UL 6	MPPPVAELCERGRDD	US19
QNTVLITDQSREEFD	UL89	VREEIPASDDVLFFV	UL57
GTTGSYTPPQDGSFP	UL139 Toledo	LQLDRLVFEAAQRGL	UL87
RVINMKAALSSIAAS	UL87	NLQARDASGLMFPII	UL46
NRELPSLFCDCPGGG	UL87	GYSAVFLLETEDAVT	UL103
IWLGIPDSHNICQHE	US13	DYVLKFLTRLAEAAT	UL86
PTEISEATHPVLATM	UL122 (IE2)	KFFVDRLCCETMIMG	US26
QRQAVSRYSGWSTEY	UL52		
QHERPSLYHDLCRSC	TRL12	*Serological group D+R+*	
PLPPWLRKKKACALT	UL37	RLPRPDTPRTPRQKK	UL99 (pp28)
		GGGGGLDRNSGNYFN	UL44 (pp50)
*Serological group D+R−*		PAPPADIDTGMSPWA	UL141 Toledo
VTKLYTSRMVTNLTV	UL16	TPRHRRRPERSKTPD	J1I
YRSGAGTFLVTHRHL	US35	ESPVPATIPLSSVIV	UL123 (IE1)
		HDSLESRRLREEEDD	UL100
*Serological group D+R+*		CQEYLHPFGFVEGPG	UL36
RLPRPDTPRTPRQKK	UL99 (pp28)	VLSHHDSLESRRLRE	UL100
ESDEEEAIVAYTLAT	UL123 (IE1)	YAEKHGGRIDGVSLL	US18
MFLGYSDCVDPGLAV	UL 5	SVSNAPPVASPSILK	UL32 (pp150)
YPAVTTVYPPSSTAK	UL32 (pp150)	TTVYPPSSTAKSSVS	UL32 (pp150)
EFDYDEDATPCVFTD	US28	LREEEDDDDDEDFED	UL100
MADSVCLPPCLSPDM	UL45	GLLRDPRLMNRQKER	UL113
IRKPPWLMEQPPPPS	IRS1	AFLHYFTTLKQYLRN	UL18
PNCCQVSVDRSRVPE	UL30	FLGARSPSLEFDDDA	UL32 (pp150)
RNGATFSKGDIEGNF	US30	RTPRQKKISQRPPTP	UL99 (pp28)
LYKGTDGLPTTDYLS	UL10	TPEQSTPSRIRKAKL	UL32 (pp150)
QTYCEDLEGRVSEAE	UL77	APGPTVANKRDEKHR	UL122 (IE2)
MESSAKRKMDPDNPD	UL122 (IE2)	SYTPPQDGSFPPPPR	UL139 Toledo
HDSLESRRLREEEDD	UL100	QMNHPPLPDPLGRPD	UL122 (IE2)
PDTPRTPRQKKISQR	UL99 (pp28)	SLSDGAPLDNGTLTA	US20
AKRKMDPDNPDEGPS	UL122 (IE2)	FLPQSPGLPPTEEEE	UL82 (pp71)
SQKPVLGKRVATPHA	UL32 (pp150)	LKDALGRQVSLRSYD	UL99 (pp28)
VLSHHDSLESRRLRE	UL100	NSGNYFNDAKEESDS	UL44 (pp50)
QQQQRHAAFSLVSPQ	UL32 (pp150)	QRGDPFDKNYVGNSG	UL44 (pp50)
IGPVDRSSLYEANPE	UL29	REPTKDLDDSFDYLV	UL78
CQEYLHPFGFVEGPG	UL36	GLDRNSGNYFNDAKE	UL44 (pp50)
FLGARSPSLEFDDDA	UL32 (pp150)	EDTSIYLSPPPVPPV	UL99 (pp28)
DEKNIFTPIKKPGTS	UL32 (pp150)	HPSPMIAAAPPAQPP	UL69
TASGEEVAVLSHHDS	UL100	SYPASYGAPVVGYDQ	UL80
QKKISQRPPTPGTKK	UL99 (pp28)	DPTYDELPSRPPQKH	IRS1
RTPRQKKISQRPPTP	UL99 (pp28)	NPANWPRERAWALKN	UL32 (pp150)
DDDEDPTYDELPSRP	IRS1	TTPPMIDLTSHHRPL	UL117
TTTAELTTEFDYDED	US28	TSPNALLPEWMDAVH	UL136 Toledo
ETRGDLFSGDEDSDS	UL32 (pp150)	SLLTAVRRHLNQRLC	IRS1
LFVGNLQARDASGLM	UL46	LIVLIGQRGGIYCYD	UL36
VTKATTFLQTMLRKE	UL122 (IE2)	PRPPPLGRGRGAGGP	IRS1
TTVYPPSSTAKSSVS	UL32 (pp150)	MTLRTFLQTYFSSDK	US15
TGNDGGGGDQIMGDK	UL31	AFIRRRRPPHHTQLV	J1I
PLCASEPEDDDEDPT	IRS1	CDGPPGSPTDSARHM	UL24
TPEQSTPSRIRKAKL	UL32 (pp150)	SFAATLLHRYPINPS	TRL 2
FGLRNCQFLAVGPDD	UL45	TVPRRRSMPAPNGPL	UL15
SHRPVCYNDTGDCTD	US 6	PATIPLSSVIVAENS	UL123 (IE1)
WLNFRVDLFGDEHRR	UL35		

The peptides presented are only detectable in serum from at least 4 out of 5 individuals in the respective group (D−R+, D+R− or D+R+, 6, 12 and 24 months post-HSCT) but never in the group D−R− at 6, 12, and 24 months post-HSCT.

**Figure 3:**
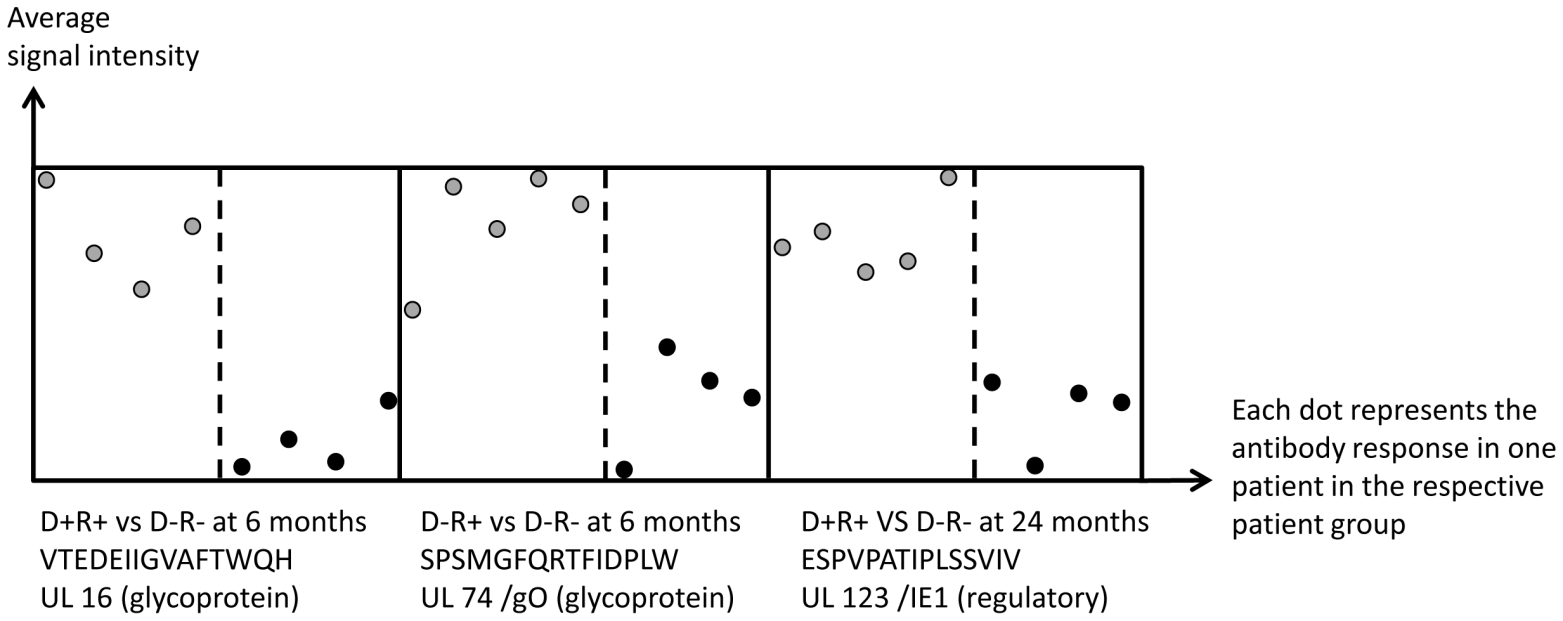
PAM analysis segregates CMV epitope responses. PAM visualizes the difference in antibody response against specific epitopes in different patient groups: distinct CMV epitope are always strongly recognized in one patient group, and always weakly recognized in the control group (or vice versa). Paradigm of weak antibody responses in patients in the D−R− group (black dots) in all patients, yet strong recognition in serum from all patients in the other serological groups (grey dots). A detailed listing of PAM-defined targets is provided in the Table S3.

In addition to peptide recognition that clusters according to the CMV status of the donor or the HSCT recipient, we identified a distinct CMV recognition pattern common to all study subjects: 372/17496 peptides (see Table S4), defined by PAM, were differentially recognized by serum IgG at all time points after HSCT. These CMV epitopes were recognized in serum from both the test- and the reference (D−R−) group with a constant strong recognition in one group (4/4 or 5/5 individuals) and constant weak recognition in the other groups (**Fig. 3**). We analyzed then if these unique peptide targets are recognized in serum from all individuals (from a patient group), but never in any individuals from the D−R− group. This intersection of PAM and the ‘exclusive recognition analysis’ allowed to identify a few CMV epitopes with the power to differentiate between patient groups (defined by CMV reactivity of the donor/recipient at the time of HSCT): 48 CMV peptides were uniquely recognized at different time points after HSCT that allowed to associate the peptide recognition pattern to the CMV profile of the donor or the recipient (Table 2).

**Table 2:** Intersection between PAM and the Exclusive Recognition analysis.

EPITOPE	HCMV ORF	Q-value
**6 months after HSCT**		
*Serological group D−R+*		
QPGENEVRPHAGVID	UL102	0.0879
RACRPFDHMPAADFR	US22	0.0466
GEVVNTMFENASTWT	UL77	0.0661
*Serological group D+R−*		
RACRPFDHMPAADFR	US22	0.2054
NISNVTYNGQRLREF	UL118	0.139
HNVTREVNVRKRAYL	UL56	0.0624
YCDLLRVGYFGHLNI*	UL 43 rev	0.025
HREKVLYLAIACFFG	UL11	0.009
*Serological group D+R+*		
SQKPVLGKRVATPHA*	UL32 (pp150)	0.7497
TTSTSQKPVLGKRVA*	UL32 (pp150)	0.4908
**12 months after HSCT**		
*Serological group D−R+*		
AQLDLEADPTAREGE	UL35	0.0207
SQKPVLGKRVATPHA	UL32 (pp150)	0.0036
*Serological group D+R−*		
VTKLYTSRMVTNLTV*	UL 11	0.0616
*Serological group D+R+*		
PNCCQVSVDRSRVPE*	UL 30	1.121
RLPRPDTPRTPRQKK*	UL 99 (pp28)	0.8275
IRKPPWLMEQPPPPS*	IRS1	0.5896
MFLGYSDCVDPGLAV*	UL 5	0.4723
HDSLESRRLREEEDD	UL100	0.3524
DESGRPRRIANRIGD	US22	0.2426
ESDEEEAIVAYTLAT*	UL123 (IE1)	0.2171
MESSAKRKMDPDNPD	UL122 (IE2)	0.2167
PDTPRTPRQKKISQR	UL 99 (pp28)	0.1748
TPEQSTPSRIRKAKL	UL 32 (pp150)	0.0961
LSDVTQRRNRPLRCL	UL 35	0.0875
RNGATFSKGDIEGNF*	US30	0.0698
YPAVTTVYPPSSTAK*	UL 32 (pp150)	0.0682
SQKPVLGKRVATPHA	UL 32 (pp150)	0.0385
YDDESWRPLSTVDDH	IRS1	0.0202
**24 months after HSCT**		
*Serological group D−R+*		
LKDALGRQVSLRSYD	UL99 (pp28)	0.1663
SQKPVLGKRVATPHA	UL32 (pp150)	0.1604
PGEPLKDALGRQVSL	UL99 (pp28)	0.1357
FGTTPGEPLKDALGR	UL99 (pp28)	0.1228
SLKPTLGGKAVVGRP	UL32 (pp150)	0.0608
TRPFKVIIKPPVPPA	UL122 (IE2)	0.0574
TSPNALLPEWMDAVH	UL136 Toledo	0.025
*Serological group D+R−*		
TNQYLIKGISYPVST*	UL75 (gH)	0.4478
ERDWRRVIHDSHGLW	US32	0.4154
AFIRRRRPPHHTQLV*	J1I	0.333
DYVLKFLTRLAEAAT*	UL86	0.2286
FAALQEQGVEDFSLE	UL57	0.0908
PRHTFDMDMMEMPAT	UL42 rev	0.0886
RWKDNKQYGQVFMTD	UL 8	0.0636
GMRAVSQFLVTHPLG*	US33	0.0354
LQLDRLVFEAAQRGL*	UL 87	0.0104
SVMLAKRPLITKPEV	UL123 (IE1)	0.0014
*Serological group D+R+*		
HDSLESRRLREEEDD	UL100	0.2181
ESPVPATIPLSSVIV*	UL123 (IE1)	0.0481
PDTPRTPRQKKISQR	UL 99 (pp28)	0.1748
TPEQSTPSRIRKAKL	UL 32 (pp150)	0.0961

The table lists peptides that were commonly defined by PAM (peptides with significant differences in the intensity of the response vs. the reference group D−R−) and as well in the ‘exclusive recognition analysis’ (never above a threshold for detection in the D−R− group). Peptides marked with a star (*) were above this threshold in all patients in the respective group but never in serum from D−R− patients. The average Q-value is the absolute difference between PAM Q-score for D−R− and the Q-score for each respective group. Higher average Q-value indicates the probability that the peptide is differently recognized between the respective group and the D−R− (reference) group.

At 6 months, possible cross-reactivity to serum IgG for the epitope QPGENEVRPHAGVID (HCMV UL102) and the aminoacid sequence from a DNA packaging tegument protein UL17 from *Herpes Simplex virus-1*, which shows a matching alignment of 5 amino acids without any mismatch. Note at 12 months, a possible cross-reactivity of serum IgG for the epitope AQLDLEADPTAREGE (HCMV UL35) and the aminoacid sequence from a protein from *Epstein-Barr virus*, RNGATFSKGDIEGNF (HCMV US30), the *Human herpesvirus-6A* protein, the epitope YPAVTTVYPPSSTAK and YDDESWRPLSTVDDH directed against proteins from *Human herpesvirus 8*, could be found. At 24 months, no matches (using the criteria outlined in materials and methods) between serum CMV epitope recognition and proteomes of other human herpesviruses were found.

### The dynamics of unique CMV immune recognition in serum is associated with the serological CMV status of the donor and recipient

Uniquely recognized CMV peptides, i.e. defined only in a particular patient group but never in the control group (‘exclusive recognition analysis’) in serum from individuals in the D−R+, D+R− or D+R+ (but never in D−R−), changed over time (**Fig. 2**). The ‘exclusive recognition analysis’ segregated best at 12 months after HSCT for the D+/R+ group (versus D−/R−). Peptides were derived from the targets UL123 (IE1), UL99 (pp28), UL32 (pp150). This recognition pattern changed at 24 months after HSCT to the IgG recognition of two strongly recognized peptides derived from UL123 (IE1, but a different epitope as compared to the 12 months analysis) and a target epitope from UL100.

CMV epitope serum reactivity in the D−R− group and the D+R− group exhibited a ‘contraction’ of the number of CMV peptides that were exclusively recognized at 12 months after HSCT. This opposite was found to be true for the ‘expansion’ of CMV epitopes recognized in serum from the D+R+ group (Figure S1). Almost 17% (8 out of 48) of the most strongly recognized CMV epitopes in this intersectional analysis originated from CMV pp150, five of 48 (10.4%) from CMV pp28 and 8.3% from CMV IE1. Other epitopes originated from the CMV gene products gH, UL100 and other structural as well as non-structural CMV proteins (see Tables 1 and 2).

### Significant changes in humoral CMV reactivity over time defined by maSigPro segregate patient groups

**Figure 4:**
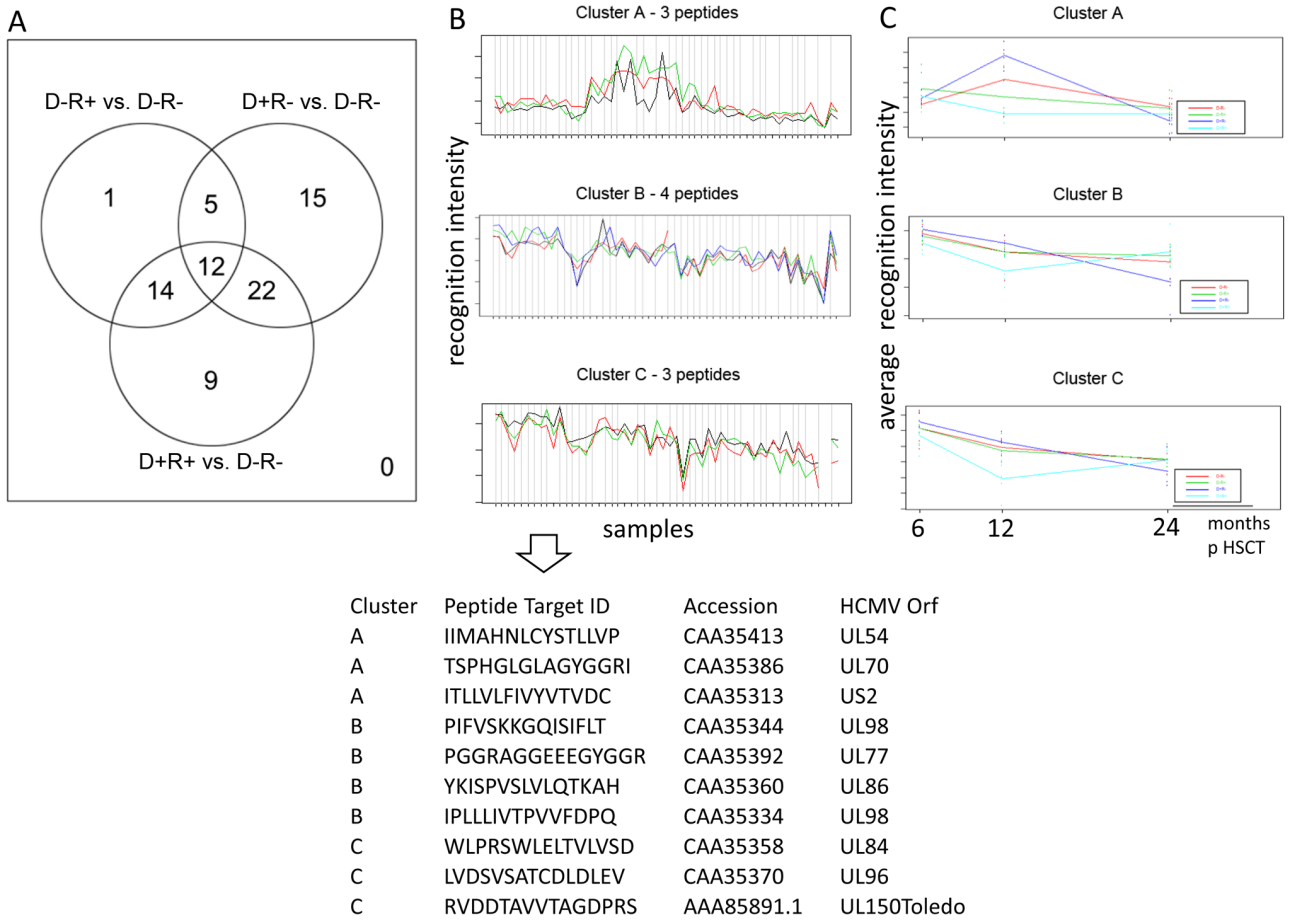
Result of the microarray significant profiles analysis (MaSigPro). **a**) Venn diagram with the number of significant peptides obtained in the three comparisons (each patient group vs. D−R−. The lists of peptides provided in the Table S5 represents the entire set of peptides contained in the Venn diagram. These peptides were also grouped into 9 clusters (default value) according to their recognition profile (see Table S5). **b–c**) Cluster analyses using CMV peptides that were differentially recognized in serum from patients, based on the D−/R− status. Three representative peptide clusters are reported, one for each analysis: D−R+ vs. D−R− (top), D+R− vs. D−R− (middle), D+R+ vs. D−R− (bottom). b) The consistency of the CMV epitope response in the cluster is visualized using the continuous peptide recognition profile across all the samples. Each peptide in the cluster is represented with a different color. c) The group-averaged CMV epitope recognition profiles (for different time points after HSCT) are shown to visualize differences (between the different patient groups) for CMV peptides selected in each cluster. Each group is represented with a different color (red = D−R−, green = D−R+, blue = D+R−, cyan = D+R+). Below the figures, peptides in the three clusters are listed. All the identified clusters and peptides are reported in the Table S4 in greater detail.

The serum CMV peptide recognition patterns for each time point after HSCT for the different patient groups were compared using the program MaSigPro (for details, see methods section). We searched for CMV peptide targets, that would allow the segregation of each patient group if *all* time points for the patient groups were considered simultaneously in the analysis. Significant differences were identified for 143 CMV epitopes in the dynamic changes of peptide recognition over time between the respective groups and the reference group D−R− (see Table S4). The resulting CMV epitopes were grouped in clusters according to the peptide recognition profiles and according to the average serum recognition signals. CMV epitope recognition patterns were identified in each patient group (see example in **Fig. 4**; the entire peptide list is provided in Table S5): 32 unique CMV epitopes segregated D−R+ between D−R− if all time points (up to 24 months) after HSCT were considered, 54 epitope segregated D+R− between D−R−, and 57 segregated the D+R+ group as compared to the D−R− control group. A Venn-diagram describing the number of exclusive and shared epitopes showed that 12 epitopes were commonly recognized in serum from all three patient groups (at all time points after HSCT, **Fig. 4**). Thus, these unique CMV peptide epitopes are capable of segregating the CMV status of the donor and recipient; these peptides are also commonly recognized in serum from patients up to 24 months after HSCT as compared to the D−R− control group. The CMV epitope NGVWVVVFLVNVLIV, recognized in serum from all three patient groups, is derived from the membrane protein US16 [34], a tropism factor that regulates, the pre-immediate-early phase of the HCMV replication cycle.

### Shift of CMV protein recognition focus over time

The origin of each individual target peptide defined by PAM and maSigPro analysis (see above) was examined. The CMV epitopes segregating each patient group are distributed among different CMV proteins. The peptide targets, defined by antibody reactivity, that segregated the patient groups (compared to the D−R− reference group) at 6 months after HSCT were directed against CMV proteins with unknown function (40%), matrix proteins (25%), glycoproteins (13%), regulatory proteins (12%), capsid (5%), and envelope (1%) proteins. This pattern was different at 24 months after HSCT: a higher proportion of antibodies were directed against proteins with regulatory function (21%). Thus, the source of uniquely recognized target epitopes was different, dependent on the time point after HSCT.

### Potential cross-reacting antibodies

Since the peptide sequences displayed as targets on the chip were provided by 15 amino acid (aa) stretches, we searched for identical epitopes which were at least 5 aa in length but we did not allow any aa mismatches within these stretches. We identified epitopes (using these filtering criteria) from related herpes virus species that could give rise to cross-reacting antibodies: 42 out of 372 (12.4%) hits (identified in PAM) and 26 out of 248 hits (12.5%) in the ‘exclusive recognition analysis’ showed at least a stretch of five identical amino acid residues within the 15mer target epitopes displayed on the microarray chip. A different situation was found to be true for the intersection between PAM and the ‘exclusive recognition analysis’: the number of CMV peptides, displaying five identical aa residues between CMV and other herpervirus species, was low, i.e. 3 out of 42 (7%) peptides shared a stretch of five amino acid residues; most of these possible hits derived from HHV-8: In PAM, 8.7% of the hits matched a protein originating from HHV-8; in the ‘exclusive recognition analysis’, 16.1% (CMV epitope) hits matched HHV-8. The intersection between PAM and the ‘exclusive recognition analysis’ revealed that 60% of all closely related epitopes were derived from the HHV-8 proteome.

### Correlation of CMV specific antibodies defined by Western Blot and peptide microarray technology

We examined the CMV-serological status pre-HSCT in the 4 patients in the D−R− group and the patients in the D+R+ group. Our results confirmed that all CMV-negative patients lacked IgG against the linear CMV test antigens at all time points before and after HSCT in contrast to the D+R+ group from whom all serum samples tested positive by Western Blot analysis (data not shown). A strong serum recognition in Western Blot analysis (targets gB1 and p150) from D+/R+ patients was associated with a high number of linear peptide epitopes defined by peptide microarray technology (see Figure S2).

### IgG recognition is associated with CD4+/CD8+ cellular target recognition and distinct cytokine production in response to individual CMV targets

**Figure 5:**
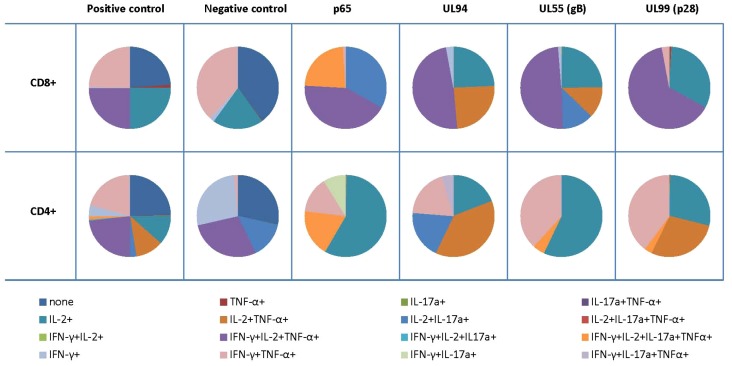
Analysis of polyfunctional T cells to previously defined CMV targets and targets defined by peptide array technology. PBMCs were incubated for 6-2 (IL-2), interferon-γ (IFN-γ) and tumor necrosis factor-alpha, interleukin-17a (IL-17a) productions were measured by intracellular cytokine staining (ICS) on the single-cell level. The cytokine response of one representative individual is shown.

The cytokine profiles of CD4+ and CD8+ T cells were analyzed upon stimulation with peptides covering the entire CMV pp65, CMV UL94, CMV UL55 (gB) and CMV UL99 (p28) proteins. We chose some of the peptide targets based on the situation that i) a T-cell response had been described previously (i.e. CMV pp65, gB) and ii) to test whether IgG CMV recognition (see Figure S3) would guide the choice for CD4+ or CD8+ T-cell recognition of other CMV targets. IL-2, TNF-α, IFN-γ and IL-17a were measured simultaneously on the single cell level to assess the presence of antigen-specific polyfunctional CD8+ and CD4+ T cells upon stimulation with peptide pools from different CMV proteins. The profile of one representative PBMC sample is shown in **Fig. 5**. In general, CD8+ T cells displayed a polyfunctional profile with co-production of IL-2, IFN-γ and TNF-α. Stimulation with pp65 showed that 25% of CD8+ T-cells exhibit co-production of all four cytokines including IL-17a. In contrast, CD4+ T cells showed a poly-functional cytokine production profile with co-production of all four cytokines after stimulation with p65; this was not found to be true for other CMV targets. Mono-functional IL-2 producing cells were predominantly seen after stimulation with CMV p65 and CMV UL55 (glycoprotein B) peptides. We did not run statistics since the number of patient's samples (n = 5 patients) was too low. Cytokine production was also seen also found in the negative control (stimulation only with medium, no peptides); most likely due to GVHD and other inflammatory processes after HSCT; yet these T-cell responses showed a different profile as compared to the CMV antigen-specific responses.

## Discussion

Entire proteomes can now be screened for immune recognition in the context of personalized medicine [35] and peptide microarray technology has enabled to pinpoint clinically and biologically relevant humoral target epitopes, e.g. for the identification of tumor associated antigens (TAAs) [36], gauging HIV vaccination [37], epitope reactivity in bacterial infections [38], [39], or targets in autoimmune diseases [39]–[42].

Due to the high resolution epitope-recognition matrix, peptide arrays allow the description of both ‘common’ (i.e. present in all patients with the same clinical endpoint) and ‘private’ (i.e. only in individual patients) CMV specific humoral recognition patterns [26]. This will be helpful in searching for anti-CMV specific B-cell responses associated to clinical outcomes; it will also aid to appreciate in more general terms the nature of the B-cell immune reconstitution using the entire CMV proteome as a target matrix to describe the dynamic nature of the immune response in individuals over time [43]. Of interest, strongly recognized CMV epitope targets appear to be shared among patients (Figure S1).

B-cell reconstitution after HSCT develops through certain stages. Transitional B-cells (CD19+, CD24high, CD38high) constitute only 4% of the circulating B-cells in adults, yet they account for 50% of B-cells in cord blood and for the majority of B-cells early after HSCT in the peripheral circulation. This B-cell population decreases as newly mature B-cells are formed, usually starting at 6 months after HSCT [44]. A large number of naïve B-cells contribute to the plasma Ig production, yet these Igs are believed to be of too low-affinity to be detected using microarray technology based on the assumption that a fluorescent-based Ig array detects approximately 10 attomoles of IgG [45]. Differences in the Ig recognition of CMV targets may stem from many different sources: i) the plasma half-life of the IgG, ii) the pattern of B-cell reconstitution, iii) the transfer of mature B-cells with the graft, iv) stimulation of the developing B-cell repertoire by commensal microbes, v) stimulation of B-cells by specific pathogens and autoantigens, vi) the cytokine/inflammation milieu including the nature of immune suppression, or the regimen used for conditioning of the host.

The antibody profile against CMV was dependent on pre-transplant defined serological groups (see Tables 1, 2). The capacity to develop antibodies to previously not recognized peptide targets was stronger in the CMV donor positive groups (see Tables 1 and 2). Comparisons over time show that the patterns of humoral responses changed after HSCT; our results indicate that the early humoral response is not originating from the transplanted donor immune system, but represents rather a recipient derived response and is thereby a reflection of pre-existing antibodies or persistent recipient B-cells [11], [13]. To prove this point, we examined in addition sera from patients pre-HSCT, followed by serum analysis after HSCT. Comparison of pre- versus (6 month) post-transplant serum showed no significant differences concerning CMV peptide recognition using PAM analysis (see Figure S4).

At 12 months, the humoral CMV-specific response vanished in all groups with exception of the group were both donor and recipient were CMV seropositive, most likely reflecting that both the earlier, older humoral response from the recipient and the new humoral response from the donor is needed to avoid a general contraction of the CMV humoral immune system after stem cell transplantation, when recipient antibody producing cells no longer persist and no antibody producing donor plasma cells are functioning yet. This phenomenon was recognized by Wahren and coworkers almost 20 years ago examining the transfer and persistence of specific B-cell responses in serologically disparate donor-recipient pairs [46]. At 24 months after HSCT – as the donor B-lymphocyte repertoire is being reconstituted - the CMV-specific humoral immune response was re-established in the groups that had a contracted humoral response at 12 months (D−R+ and D+R−). Not mutually exclusive, the T-cell compartment may also contribute to (CMV) antibody diversity: CD8+ TCRalpha/beta repertoire diversity, yet not the magnitude of the T-cell response, was inversely related to anti-CMV antibody levels [47]. These results suggested that CD8+ T-cell diversity appears to be crucial in curbing CMV infections. The association of CMV – directed humoral and T-cell responses implies that appreciation of the breadth of humoral immune response, defined by peptide recognition pattern analysis, may have to be complemented by TCR CDR3 (complementarity determining region 3) diversity analysis in CD8+ T-cells from the respective patient.

Anti- glycoprotein B directed antibodies have been incriminated in anti-virus specific neutralizing capacity, these antibodies differ in their fine specificity. Target-directed antibody diversity has clinical consequences (e.g. neutralization), particularly if an epitope-specific B-cell response arises from a single parental B-cell clone. This impacts on vaccine development, since vaccines may not only need to active and expand different B-cell clones, yet also drive the evolution of single B-cell clones [48]; peptide microarray technology will therefore help to visualize the antibody recognition pattern in serum and provide biologically relevant information whether the anti-CMV directed immune responses is rather focused, or directed against a broad spectrum of CMV epitope targets.

If we hypothesize that the humoral recognition patterns (displayed in the D−R− group at different time points after HSCT) reflects either natural occurring antibodies or “cross-reactive” antibodies, due to exposure to similar epitopes displayed by different pathogens or commensal microbes, then a very focused B-cell recognition would take place if CMV is encountered from a CMV naïve donor in an CMV+ recipient. Of note, all CMV epitopes in the highly selective intersection of PAM and the ‘exclusive recognition analysis’ in this patient group (D−R+) at 24 months originate from CMV gene products that have already been described to stimulate a strong immune response in CD8+ cells (i.e. CMV pp28, CMV pp150 and CMV IE2), reinforcing the hypothesis that CD8+ immune response and CD4+ immune responses, providing help to B-cells, are closely orchestrated.

Antibodies against epitopes from closely related amino acid sequences from different herpesviruses may cross-react [49], [50] and this observation may in part account for the serum recognition pattern found in the D−R− group. We identified a higher percentage of possible CMV peptide targets showing similar amino acid sequences to other herpesviral species (12.5% and 12.4%) using PAM as well as in the ‘exclusive analysis. If PAM is combined with the ‘exclusive recognition analysis’ at least 10.4% of potential cross-reactive epitopes are identified, suggesting that antibodies directed against the CMV epitope listed in the latter group may be much more precisely directed against CMV. The potential cross-reaction with HHV-8 appears to be not that clinically relevant, since HHV-8 infections are quite rare in the Swedish population [51]. It is therefore likely that the HHV-8 epitopes, defined in this report, reflect in fact anti-CMV directed reactivity [52].

During natural CMV infection, potentially neutralizing antibodies targeting CMV glycoproteins, e.g. gH, gB and gM have been identified [8], [10], [53]. These target proteins are rather conserved among human herpesvirus species [54], which might explain why antibodies recognizing these epitopes (and differentiating the different patient groups) are not found in our study, since we used the D−R− group as a negative reference group (see material and methods section).

Several genetically different CMV strains circulate in the population [55]–[58] and the presence of more than one CMV strain has been observed in immune competent individuals [59], [60]. Therefore, by focusing the statistical analysis on peptides recognized by all individuals in one group (for serum harvested at a single time point after HSCT but never in any individual from the D−R− group), we postulated that this approach would aid to antibodies independent of the CMV strain. Due to the use of the additional analysis with MaSigPro, which considers the dynamics of the peptide recognition over time, we are able to reliably identify humoral CMV targets peptides that would distinguish the seropositive donors/recipients against the D−R− reference group. A potential limitation to our findings is clinical course of CMV infection. We selected a group of long-term survivors and CMV has a major impact on the survival after allogeneic HSCT [61]. Thus, our patient group might represent a positive selection. Our data also show that B-cell responses, identified by peptide microarrays, may be used to identify functional CD8+ and CD4+ T-cell responses directed against already described and newly identified cellular CMV targets in PBMCs from patients after HSCT,. This has to be confirmed with a broader variety of CMV proteins and with a larger patient cohort - in order to appreciate the diversity of the immune response in patients with different genetic backgrounds.

To conclude, CMV epitope proteome mapping aids to describe the breadth of humoral immune reconstitution after HSCT; it will also help to map, in an unbiased fashion, immune responses induced by CMV vaccines in a post-transplant setting.

In the early phase of a CMV infection, antibodies will be detected to tegument proteins p150 and p65 as well as to non-structural-proteins IE1 and CM2. Antibodies to glycoproteins gB1 and gB2 are detectable 6–8 weeks after a primary CMV infection.

## Supporting Information

Figure S1 **Compilation of the entire CMV epitope recognition profile in the patient groups, defined by D/R CMV serological status.**
(PDF)

Figure S2 **Comparison between Western Blotresults and peptide array recognition patterns.**
(PDF)

Figure S3 **Choice of CMV proteins for CD4+ and CD8+ T-cell recognition, defined by intracellular cytokine staining, and peptide- microarry based antibody recognition. C.**
(PDF)

Figure S4 **Very similar CMV peptide recognition pattern in pre- and posttransplantation serum samples. S.**
(PDF)

Table S1 **Patient characteristics.**
(PDF)

Table S2 **Chip Design: HCMV Proteins on the Microarray Slides.**
(PDF)

Table S3 (PDF)

Table S4 **Epitopes Predicted By PAM - Serum CCMV Epitope Recognition After HSCT.**
(PDF)

Table S5 **MaSigPro analysis.**
(PDF)
