# Supplementary material for: Whole CMV Proteome Pattern Recognition Analysis after HSCT Identifies Unique Epitope Targets Associated with the CMV Status
Source: PLoS One. 2014 Apr 16;9(4):e89648. doi: 10.1371/journal.pone.0089648 (PMC3989190; doi:10.1371/journal.pone.0089648)
Supplement: Figure S1 — Compilation of the entire CMV epitope recognition profile in the patient groups, defined by D/R CMV serological status. (PDF) [file pone.0089648.s001.pdf]

Supplementary Figure S 1

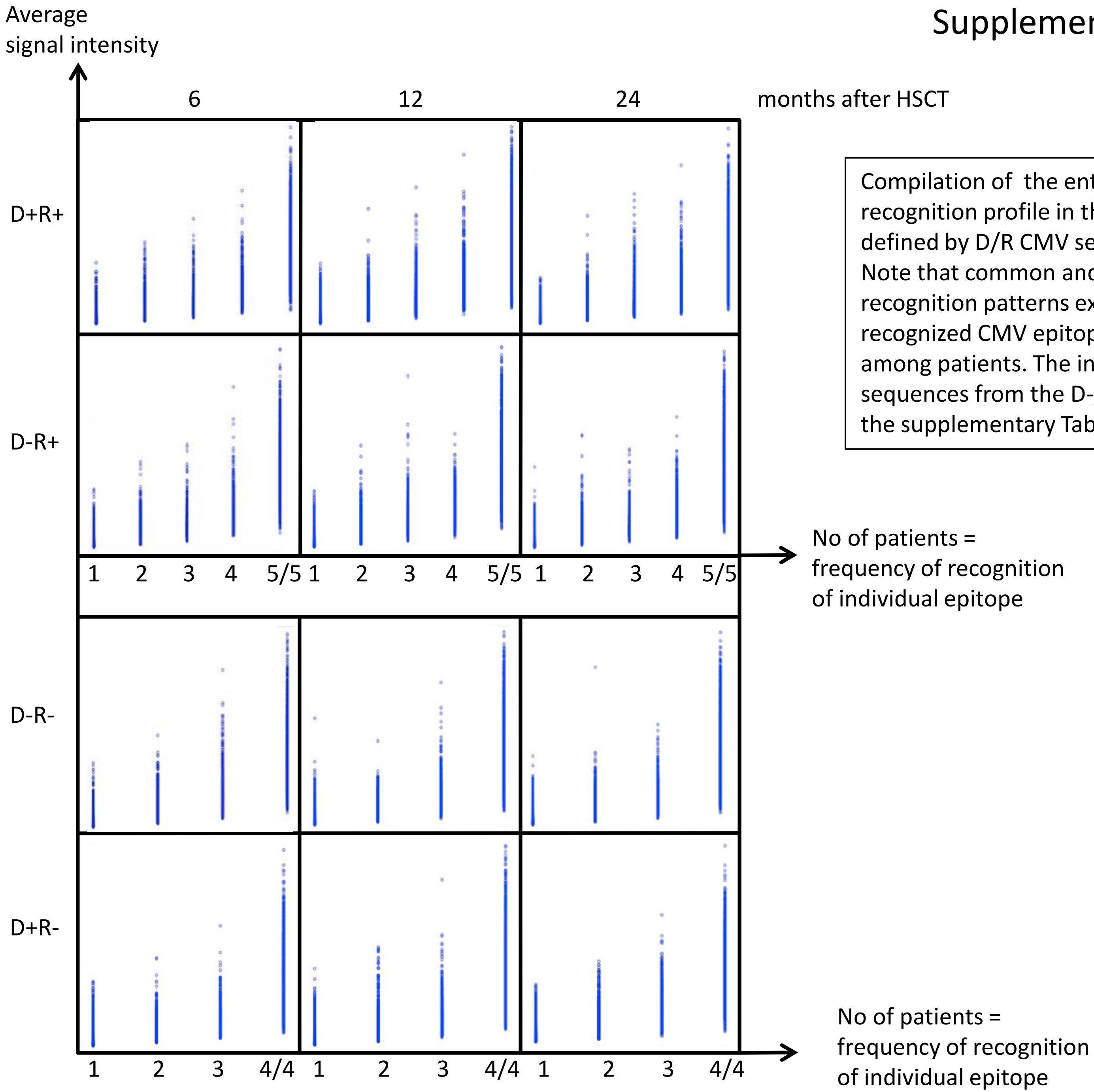

Compilation of the entire CMV epitope recognition profile in the patient groups, defined by D/R CMV serological status. Note that common and 'private' recognition patterns exist. Strongly recognized CMV epitopes are shared among patients. The individual peptide sequences from the D-/R- are listed in the supplementary Table S3A and S3B.
