# Supplementary material for: Whole CMV Proteome Pattern Recognition Analysis after HSCT Identifies Unique Epitope Targets Associated with the CMV Status
Source: PLoS One. 2014 Apr 16;9(4):e89648. doi: 10.1371/journal.pone.0089648 (PMC3989190; doi:10.1371/journal.pone.0089648)
Supplement: Figure S2 — Comparison between Western Blotresults and peptide array recognition patterns. (PDF) [file pone.0089648.s002.pdf]

Supplementary Figure S2

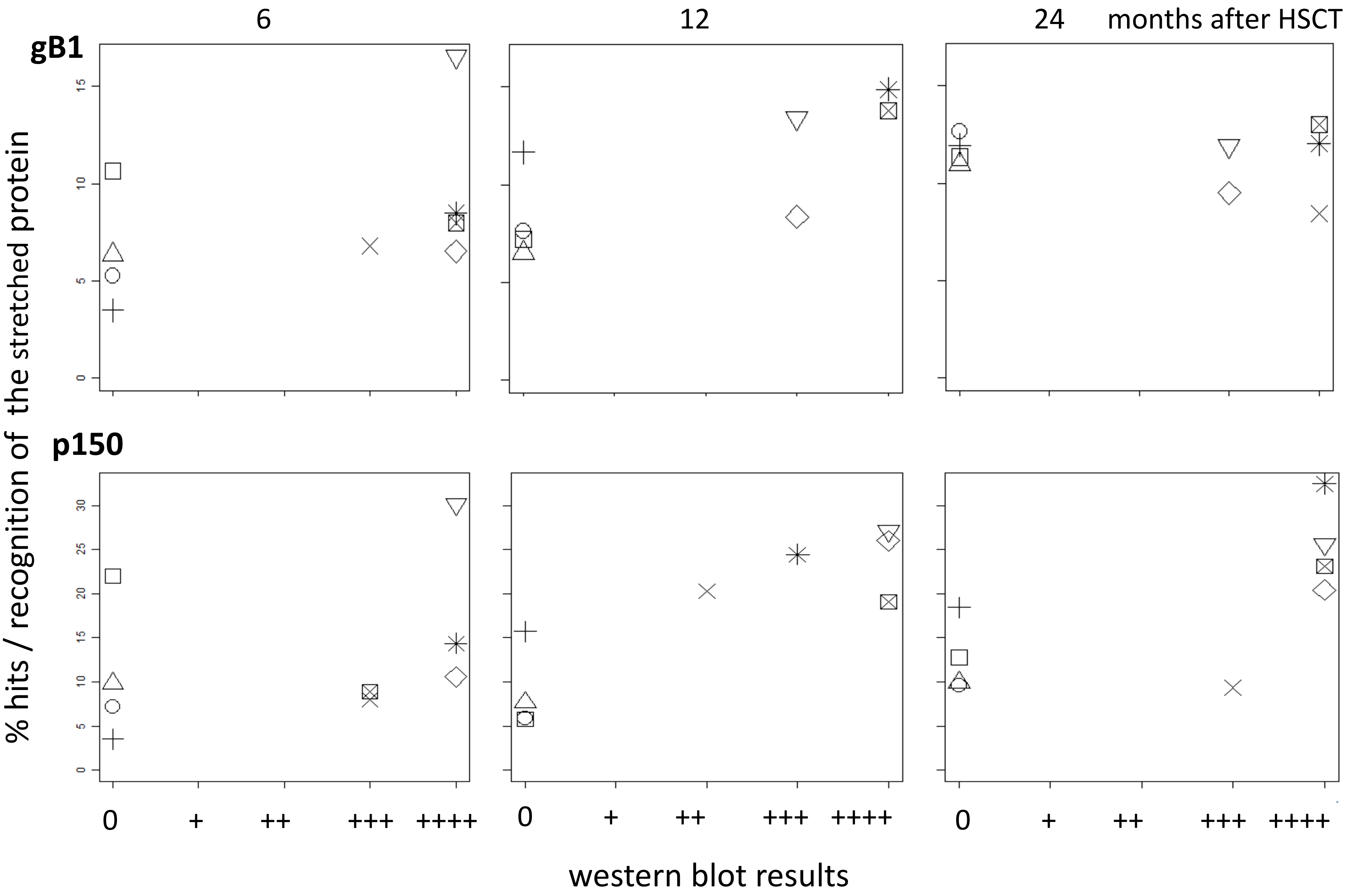

Comparison between Western Blot results and peptide array recognition patterns. Serum samples from patients were tested for CMV protein recognition using Western Blot as described in the material and methods section; recognition of CMV proteins was graded according to the manufacturers guidelines, i.e zero and + to ++++.

The strength of recognition of gB1 and p150 was compared to the peptide microarry results The length of each individual target protein from the first till the last amino acid residue represents 100 % of the length of the target protein. The % of hits indicates the area of the target protein (in its linear format as peptid stretches) recognized by serum antibodies. We show, as a paradigm, the result from eight patients (designated with different symbols, i.e. star, circle, triangle and quadrant for D-/R- patients; diamond, inversed triangle, closed quandrant and double-star, D+/R+ patients).

Strong recognition of CMV targets in the Western Blot (D+/R+ patients) is associated with the highest number of hits (number of linear peptides); despite negative results in the Western Blot (D-/R- patients), recognition of linear CMV epitopes using the peptide microarray platform. Linearized proteins, displayed by Western Blot, may not display the identical set of linear epitopes displayed by microarry technology. In addition, antiboy 'cross-reactivity' directed to 15 mer peptides and related other proteins (e.g. other *herpesvirus species* or even non-related pathogens) may be more likely as compared to entire, complex proteins.
