# Supplementary material for: Whole CMV Proteome Pattern Recognition Analysis after HSCT Identifies Unique Epitope Targets Associated with the CMV Status
Source: PLoS One. 2014 Apr 16;9(4):e89648. doi: 10.1371/journal.pone.0089648 (PMC3989190; doi:10.1371/journal.pone.0089648)
Supplement: Figure S3 — Choice of CMV proteins for CD4+ and CD8+ T-cell recognition, defined by intracellular cytokine staining, and peptide- microarry based antibody recognition. C. (PDF) [file pone.0089648.s003.pdf]

Supplementary Figure S3

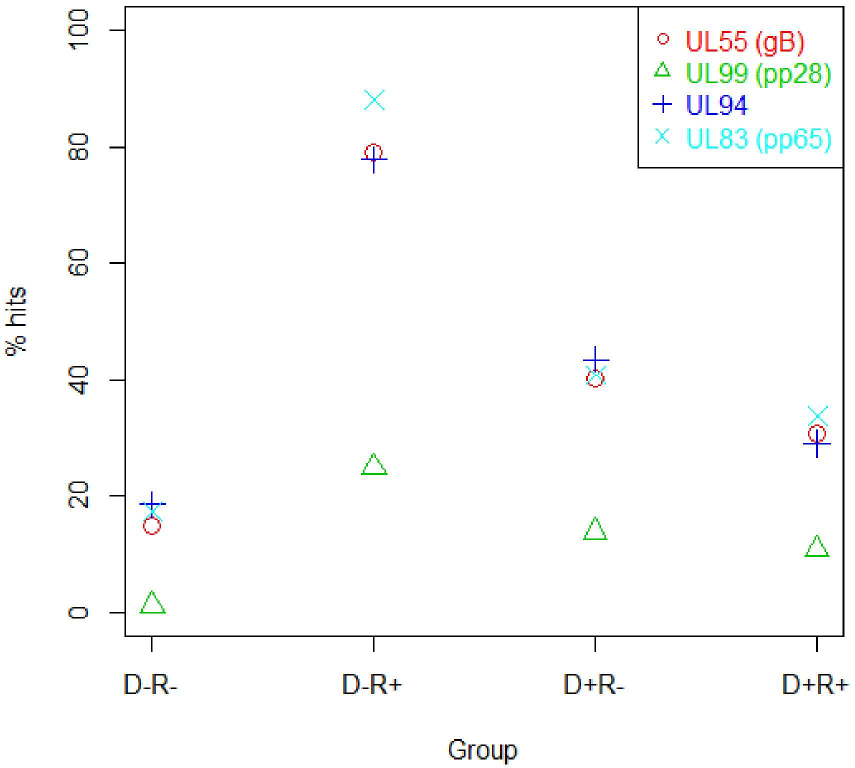

Choice of CMV proteins for CD4+ and CD8+ T-cell recognition, defined by intracellular cytokine staining, and peptide-microarry based antibody recognition. CMV proteins UL55, UL99, UL94 and UL85 were tested for antibody recognition defined as fluorescence intensity using peptide microarry technology. The length of each individual target protein from the first till the last amino acid residue represents 100 % of the length of the target protein. The % (mean) of hits indicates the area of the target protein (in its linear format as peptid stretches) recognized by serum antibodies in serum from patients A – R and 1-7, segregated by the serum status (see below). Weak recognition of the target in D-/R- patients. (Note the strong recognition in D-/R+ individuals). Data are from the timepoint 12 month after HSCT. The peptide cocktails (covering the entire proteins UL55, UL99, UL94 and UL85) were tested for T-cell recognition using PBMCs from patients 1-7, from whom we had sufficient numbers of PBMCs available.

		D-R-	D-R+	D+R-	D+R+
**UL55(gB)**	CAA35414	14.94	79.11	40.13	30.79
**UL99(pp28)**	CAA35335	1.2	24.89	13.82	10.76
**UL94**	CAA35368	18.55	77.78	43.42	29
**UL83(pp65)**	CAA35357	17.23	88	40.79	33.63
