# Supplementary material for: Whole CMV Proteome Pattern Recognition Analysis after HSCT Identifies Unique Epitope Targets Associated with the CMV Status
Source: PLoS One. 2014 Apr 16;9(4):e89648. doi: 10.1371/journal.pone.0089648 (PMC3989190; doi:10.1371/journal.pone.0089648)
Supplement: Figure S4 — Very similar CMV peptide recognition pattern in pre- and posttransplantation serum samples. S. (PDF) [file pone.0089648.s004.pdf]

Supplementary Figure S4

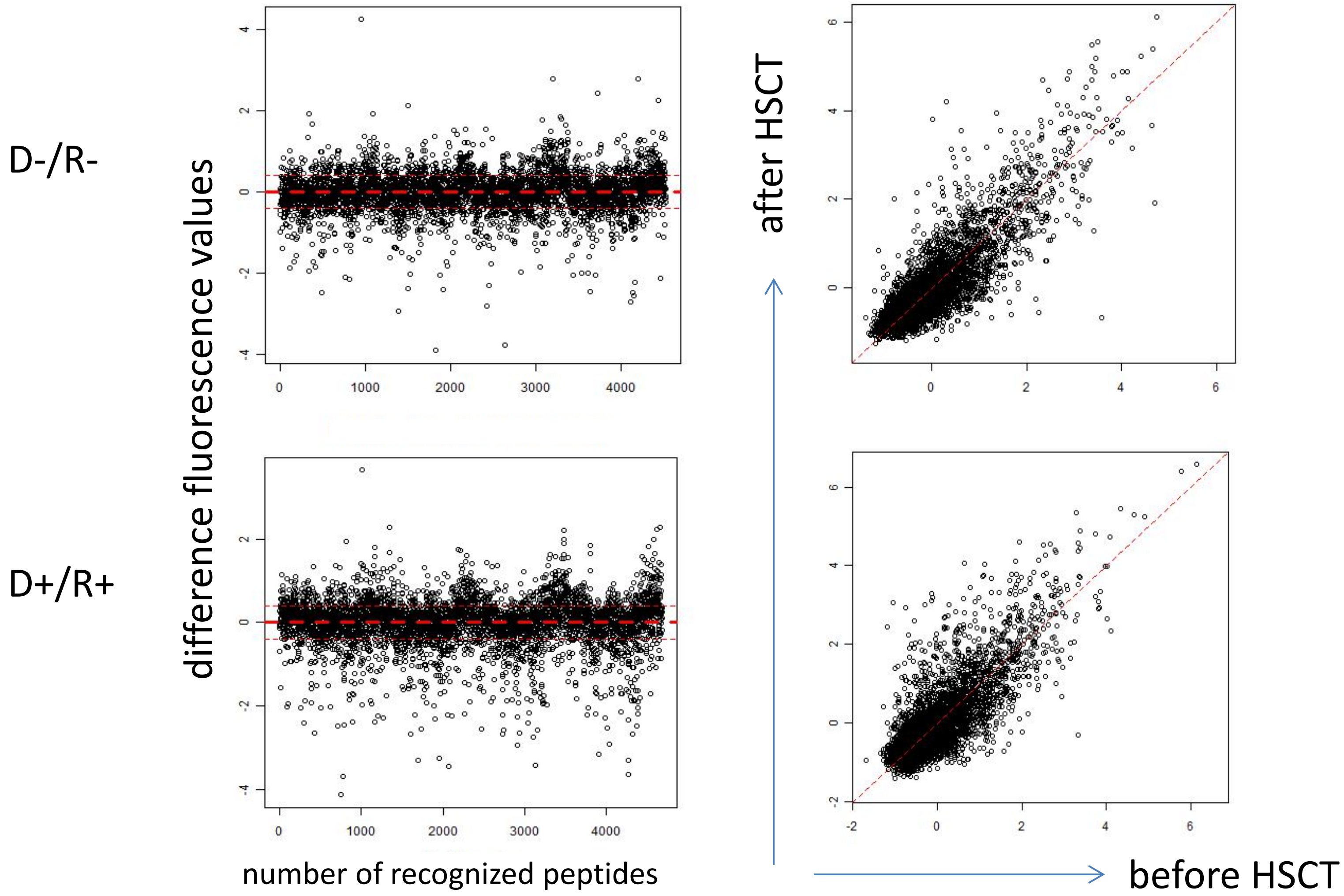

Very similar CMV peptide recognition pattern in pre- and post transplantation serum samples. Serum from patients A, B and C (D-/R-) and patients Q, R (D+/R+) was tested - matched pairs for each individual patient - prior to HSCT and 6 month after HSCT. PAM analysis was performed in order to identify significant differences pre and after HSCT. Although we identified variations, no significant difference concerning the CMV recognition pattern could be observed pre and post – HSCT. Note that PAM identified differences in serum recognition between months 6, 12 and 24th. This demonstrates the CMV epitope pattern recognition in serum at 6 month past HSCT is quite similar to the recognition pattern prior to HSCT. We show here 2 examples as a paradigm.
