# Supplementary material for: Whole CMV Proteome Pattern Recognition Analysis after HSCT Identifies Unique Epitope Targets Associated with the CMV Status
Source: PLoS One. 2014 Apr 16;9(4):e89648. doi: 10.1371/journal.pone.0089648 (PMC3989190; doi:10.1371/journal.pone.0089648)
Supplement: Table S1 — Patient characteristics. (PDF) [file pone.0089648.s005.pdf]

Supplementary Tables S1

Table S1 - Patient characteristics

Serological group	Pat	Age at HSCT	SCT-type	Cond.	TBI 0=no 1=yes	HSC source	Primary diagnosis	CMV-infection 0=no; 1=yes	CMV-disease	Anti-CMV treatment
D-R-	A	17.1	MUD	conv	0	PB	CML chronic phase	0	0	-
D-R-	B	35.7	MUD	conv	0	BM	CML	0	0	-
D-R-	C	7.5	SIB	red	0	BM	Fanconi's anemia	0	0	-
D-R-	D	53.1	SIB	conv	0	PB	AML	0	0	-
D-R-	E (removed)	14.9	SIB	conv	1	BM	ALL CR3	1	0	-
D-R+	F	52.8	MUD	red	0	PB	Polycytemia Vera	1 (low VL)	0	-
D-R+	G	14.8	SIB	conv	1	BM	ALL CR2	1	0	GCV
D-R+	H	11.6	MUD	conv	0	BM	AML CR	0	0	-
D-R+	I	53.8	SIB	red	0	PB	CML	1	0	GCV
D-R+	J	45.4	MUD	conv	0	PB	AML CR1	1	0	GCV/ Foscarnet
D+R-	K (removed)	0.8	misMUD	conv	0	CB	AML M5B CR	0	0	-
D+R-	L	6.5	SIB	red	0	BM	Fanconi's anemia	1 (low VL)	0	-
D+R-	M	0.5	MUD	conv	0	PB	FHL	0	0	-
D+R-	N	65.8	SIB	red	0	PB	Colorectal cancer	0	0	-
D+R-	O	1.3	FAM	conv	0	BM	Thalassemia major	0	0	-
D+R+	P	41.7	SIB	red	0	PB	AML	0	0	-

D+R+	Q	3.6	SIB	conv	1	BM	T cell ALL	1	0	GCV
D+R+	R	44.4	MUD	conv	0	PB	AML CR2	1	0	GCV
D+R+	S	3.5	MUD	conv	0	BM	AML CR2	0	0	-
D+R+	T	40.9	MUD	conv	0	PB	AML CR	1	0	GCV
R+D-	ICS pat 1	58.2	SIB	conv	1	PB	NHL	1	0	-
R+D+	ICS pat 2	36.0	MUD	conv	1	BM	Hodgkin lymphoma	1	0	GCV
R+D+	ICS pat 3	38.6	MUD	conv	0	BM	AML CR1	1	0	GCV
R+D+	ICS pat 4	53.8	MUD	red	0	PB	AML CR1	1	0	GCV
R+D+	ICS pat 5	56.6	MUD	conv	0	PB	AML CR1	1	0	GCV
R+D+	ICS pat 6	63.3	MUD	red	0	PB	AML CR1	0	0	-
R+D+	ICS pat 7	37.5	MUD	red	0	PB	AML CR1	0	0	-

Pat, patient; HSCT, hematopoietic stem cell transplantation; SCT-typ, stem cell transplantation type; Cond., conditioning; TBI, total body irradiation; HSC source, hematopoietic stem cell source; CMV, *Cytomegalovirus*; D+, CMV seropositive donor; R-, CMV seronegative recipient; MUD, matching unrelated donor; SIB, sibling donor; misMUD, mis-matching MUD ([1]Pat K HLA-B allel mismatch but was HLA-DR allele identical); FAM, family donor; conv, conventional; red, reduced; PB, peripheral blood; BM, bone marrow; CB, cord blood; CML, chronic myeloid leukemia; AML, acute myeloid leukemia; ALL, acute lymphoblastic leukemia; CR, clinical regression; FHL, familial hemophagocytic lymphohistiocytosis; VL, viral load; GCV, ganciclovir. The material from patients 'ICS1-7' was used to analyze both CMV epitope recognition patterns as well as intracellular cytokine staining in response to the corresponding peptide pools.
