# Supplementary material for: Whole CMV Proteome Pattern Recognition Analysis after HSCT Identifies Unique Epitope Targets Associated with the CMV Status
Source: PLoS One. 2014 Apr 16;9(4):e89648. doi: 10.1371/journal.pone.0089648 (PMC3989190; doi:10.1371/journal.pone.0089648)
Supplement: Table S2 — Chip Design: HCMV Proteins on the Microarray Slides. (PDF) [file pone.0089648.s006.pdf]

Supplementary Table S 2

CHIP DESIGN: HCMV PROTEINS ON THE MICROARRAY SLIDES

No	HCMV ORF [1]	Product Function [2]	Gene Bank or Swiss Prot ID [3]	Other Notes
1	IRL14	unk	CAA35296	
2	IRS1	r	CAA35311	
3	J1I	unk	CAA35310	
4	TRL1	r	CAA35449	
5	TRL2	unk	CAA35450	
6	TRL3	unk	CAA35451	
7	TRL4	unk	CAA35453	
8	TRL5	unk	CAA35452	
9	TRL6	unk	CAA35454	
10	TRL7	unk	CAA35455	
11	TRL8	unk	CAA35456	
12	TRL9	unk	CAA35457	
13	TRL10	gp	CAA35458	
14	TRL11	gp	CAA35459	
15	TRL12	unk	CAA35460	
16	TRL13	unk	CAA35461	
17	TRL14	unk	CAA35433	
18	TRS 1 part	m	CAA35269	[4]
19	UL1	e	CAA35434	
20	UL2	unk	CAA35435	
21	UL3	unk	CAA35436	
22	UL4 (gp48)	gp	CAA35437	
23	UL5	unk	CAA35438	
24	UL6	unk	CAA35439	
25	UL7	unk	CAA35440	
26	UL8	unk	CAA35441	
27	UL9	unk	CAA35442	
28	UL10	unk	CAA35443	
29	UL11	unk	CAA35444	
30	UL12	unk	CAA35446	
31	UL13	unk	CAA35445	
32	UL14	unk	CAA35447	
33	UL15	unk	CAA35415	
34	UL16	gp	CAA35448	
35	UL17	unk	CAA35416	
36	UL18	gp	CAA35417	
37	UL19	unk	CAA35418	
38	UL20	gp	CAA35419	
39	UL21	unk	CAA35420	
40	UL22A	gp	P16845	[10]
41	UL22	gp	CAA35421	
42	UL23	m	CAA35422	
43	UL24	m	CAA35423	
44	UL25	m	CAA35424	
45	UL26	m	CAA35425	

46	UL27	unk	CAA35426	
47	UL28	unk	CAA35427	
48	UL29	unk	CAA35428	
49	UL30	unk	CAA35429	
50	UL31	unk	CAA35430	
51	UL32 (pp150)	m	CAA35431	
52	UL33	r	CAA35432*	[5]
53	UL34	r	CAA35393	
54	UL35	m	CAA35394	
55	UL36	m	CAA35395	
56	UL37	r	CAA35396	
57	UL38	unk	CAA35397	
58	UL39	unk	CAA35398	
59	UL40	r	CAA35399	
60	UL41	unk	CAA35400	
61	UL41 alt	unk	CAA74073	[6]
62	UL42 rev	unk	CAA74074	[6]
63	UL43 rev	m	CAA74075	[6]
64	UL44 (pp50)	r	CAA35403	
65	UL45	r	CAA35404	
66	UL46	c	CAA35405	
67	UL47	m	CAA35406	
68	UL48 (pp212)	c	CAA35407	
69	UL48A (SCP)	c	Q7M6N6	[10]
70	UL49	c	CAA35408	
71	UL50	m?	CAA35409	
72	UL51	m	CAA35410	
73	UL52	m	CAA35411	
74	UL53	m	CAA35412	
75	UL54	r	CAA35413	
76	UL55 (gB)	gp	CAA35414	
77	UL56	m	CAA35371	
78	UL57	r	CAA35372	
79	UL58	unk	CAA35373	
80	UL59	unk	CAA35374	
81	UL60		CAA35375	
82	UL61	unk	CAA35376	
83	UL62	unk	CAA35377	
84	UL63	unk	CAA35378	
85	UL64	unk	CAA35379	
86	UL65	m?	CAA35380	
87	UL66	unk	CAA35381	
88	UL67	unk	CAA35382	
89	UL68	unk	CAA35383	
90	UL69	r	CAA35384	
91	UL70	r	CAA35386	
92	UL71	unk	CAA35385	
93	UL72	r	CAA35387	
94	UL73 (gN)	gp	CAA35388	
95	UL74 (gO)	gp	CAA35389	
96	UL75 (gH)	gp	CAA35390	
97	UL76	m?	CAA35391	
98	UL77	gp	CAA35392	

99	UL78	o	CAA35351	
100	UL79	unk	CAA35352	
101	UL80	c	CAA35353	
102	UL80A	o	CAA35354	
103	UL81	unk	CAA35355	
104	UL82 (pp71)	m	CAA35356	
105	UL83 (pp65)	m	CAA35357	
106	UL84	r	CAA35358	
107	UL85	c	CAA35359	
108	UL86 (MCP)	c	CAA35360	
109	UL87	m?	CAA35361	
110	UL88	m	CAA35362	
111	UL89	r	CAA35363	
112	UL90	unk	CAA35364	
113	UL91	unk	CAA35365	
114	UL92	unk	CAA35366	
115	UL93	m?	CAA35367	
116	UL94	m	CAA35368	[7]
117	UL95	m?	CAA35369	
118	UL96	m?	CAA35370	
119	UL97	r	CAA35333	
120	UL98	m?	CAA35334	
121	UL99 (pp28)	m	CAA35335	
122	UL100 (gM)	gp	CAA35336	
123	UL101	unk	CAA35337	
124	UL102	o	CAA35338	
125	UL103	unk	CAA35339	
126	UL104	m	CAA35341	
127	UL105	o	CAA35340	
128	UL106	unk	CAA35342	
129	UL107	unk	CAA35343	
130	UL108	unk	CAA35344	
131	UL109	unk	CAA35347	
132	UL110	unk	CAA35348	
133	UL111	unk	CAA35349	
134	UL111A rev	unk	CAA35350	
135	UL112 part	unk	P16768	[8]
136	UL113	unk	CAA35315	
137	UL114	o	CAA35316	
138	UL115 (gL)	gp	P16832	
139	UL116	unk	CAA35318	
140	UL117	unk	CAA35319	
141	UL118	unk	CAA35320	
142	UL119	gp	CAA35321	
143	UL120	unk	CAA35322	
144	UL121	unk	CAA35323	
145	UL122 (IE2)	r	P19893	[10]
146	UL123 (IE1 aka pp72)	r	CAA35325	[9]
147	UL124	unk	CAA35326	
148	UL125	unk	CAA35327	
149	UL126	unk	CAA35328	
150	UL127	unk	CAA35329	
151	UL128 (viral entry)	gp	CAA35330	

152	UL129 (viral entry)	gp	CAA35331
153	UL130 (viral entry)	gp	CAA35332
154	UL131	unk	CAA35294
155	UL132	unk	CAA35295
156	UL133 Toledo	unk	AAA85872.1
157	UL134 Toledo	unk	AAA85873.1
158	UL135 Toledo	unk	AAA85874.1
159	UL136 Toledo	unk	AAA85875.1
160	UL137 Toledo	unk	AAA85876.1
161	UL138 Toledo	unk	AAA85877.1
162	UL139 Toledo	unk	AAA85878.1
163	UL140 Toledo	unk	AAA85879.1
164	UL141 Toledo	unk	AAA85880.1
165	UL142 Toledo	unk	AAA85881.1
166	UL143 Toledo	unk	AAA85882.1
167	UL144 Toledo	unk	AAA85883.1
168	UL145 Toledo	unk	AAA85884.1
169	UL146 Toledo	unk	AAA85885.1
170	UL147 Toledo	r?	AAA85886.1
171	UL148 Toledo	unk	AAA85887.1
172	UL149 Toledo	unk	AAA85890.1
173	UL150 Toledo	unk	AAA85891.1
174	UL151 Toledo	unk	AAA85892.1
175	UL152 Towne	o	AAA85894.1
176	UL153 Towne	unk	AAA85895.1
177	UL154 Towne	unk	AAA85896.1
178	US1	unk	CAA35312
179	US2	r	CAA35313
180	US3	r	CAA35314
181	US4	unk	CAA35271
182	US5	unk	CAA35272
183	US6	r	CAA35273
184	US7	unk	CAA35274
185	US8	unk	CAA35275
186	US9	gp	CAA35276
187	US10	unk	CAA35277
188	US11	r	CAA35278
189	US12	unk	CAA35279
190	US13	unk	CAA35280
191	US14	unk	CAA35281
192	US15	unk	CAA35282
193	US16	unk	CAA35283
194	US17	unk	CAA35284
195	US18	unk	CAA35285
196	US19	unk	CAA35286
197	US20	unk	P09724
198	US21	unk	CAA35288
199	US22	m	CAA35289
200	US23	m	CAA35290
201	US24	m	CAA35291
202	US25	unk	CAA35292
203	US26	unk	CAA35293
204	US27	r	CAA35259

205	US28	gp	P09704
206	US29	unk	CAA35261
207	US30	unk	CAA35262
208	US31	unk	CAA35263
209	US32	unk	CAA35264
210	US33	unk	CAA35266
211	US34	unk	CAA35265
212	US35	unk	CAA35267
213	US36	unk	CAA35268
214	Y9K	unk	P21600

NOTES. -

[1] All sequences are from AD169 unless otherwise indicated.

[2] c=Capsid, m=Matrix or tegument, e=Envelope, gp=Glycoprotein, r=DNA or regulatory, o=Other, unk=Unknown

[3] Sequence bank identifications:
Sequences have been downloaded from Genbank (GB; typical: 'CAA35350' and 'AAA85872.1'), found via the NCBI (protein database) site (www.ncbi.nlm.nih.gov/entrez/query.fcgi?db=Protein). In some cases the sequence was downloaded from the SwissProt database (SP; typical: 'P16768') using the expasy (expert protein analysis system) site (http:us.expasy.org/). SP was necessary, since some published changes were only updated in the SwissProt database.

The Protein Database contains sequence data from the translated coding regions from DNA sequences in GenBank, EMBL, and DDBJ as well as protein sequences submitted to Protein Information Resource (PIR), SWISS-PROT, Protein Research Foundation (PRF), and Protein Data Bank (PDB) (sequences from solved structures).

[4] TRS1 (part): Only part of the full sequence was tested in our mix. The N-terminal part of TRS1 is identical to IRS1 (aa 1-549; IRS peptides # 1-134), except for aa 190. Peptides for this portion of the ORF are not included in the file submitted. For the C-terminal part of TRS1 the sequence for overlapping peptides starts at aa 537.

[5] UL33: Exon1 was added to the sequence from Genebank according to: Davis-Poynter NJ et al, J Virol 1997 Feb;71(2):1521-9.

[6] UL41-UL43 (alternative and revised sequences): The originally published AD169 sequence lacked 929 base pairs, resulting in an additional ORF UL41 alt, which is completely different from UL41. The insertion affected the ORFs UL42 (different N-terminus) and UL43 (longer). The new revised UL42 and UL43 are therefore labelled with rev to exclude any confusion with the previously predicted ORFs UL42 and UL43, which are still showing up in the databases. Dargan J.Virol. 1997 and Mocarski J. Virol., 1997.

[7] UL94: Contains an epitope which is recognized from sera of patients with systemic sclerosis. Nature Medicine vol6 Nr10 Oct.2000 p1183-1186.

[8] UL112 (part): Only part of the full sequence was tested on our current CMV chip layout. Amino acids 1-253 are identical to UL113, so peptides for this portion were not included in the UL112 array print.

[9] UL123 (part): Only part of the full sequence was tested on our peptide microarray chip. Amino acids 1-85 are identical to UL122 (peptides UL122 #1-18). Peptides for this portion were not included in UL123. For the C-terminal portion of UL123 the sequence for overlapping peptides starts at amino acid 72(102 peptides).

[10] Mocarski Jr ES, Trends Microbiol., 10(7): 332-339, 2002 (Review)
