# Supplementary material for: Whole CMV Proteome Pattern Recognition Analysis after HSCT Identifies Unique Epitope Targets Associated with the CMV Status
Source: PLoS One. 2014 Apr 16;9(4):e89648. doi: 10.1371/journal.pone.0089648 (PMC3989190; doi:10.1371/journal.pone.0089648)
Supplement: Table S3 — (PDF) [file pone.0089648.s007.pdf]

Supplementary Table S3A

Top 20 CMV shared peptides (based on the fluorescence signal) in serum from 4/4 patients (D-/R-) at 6, 12 and 24 month after HSCT. See also supplementary Figure S1 for the distribution of commonly (shared) and private (individually) recognized CMV peptides in serum from individuals with the D-/R- constellation.

6 month after HSCT

Peptide	UL-ORF	Designation
PPDCSPPPYRPPYCL	CAA74074	UL42rev
VFPCFVPEPWQLMNL	CAA35387	UL72
GNARLDALMSASEWW	CAA35404	UL45
ACRDWDSMHCTPFWS	AAA85887.1	UL148Toledo
QWGRYENGSTPVLWC	CAA35439	UL6
EPHGQWEFMFREQRG	CAA35311	IRS1
SSFWYHGNCELCGWN	CAA35434	UL1
HALALWMDWADVRSC	CAA35367	UL93
LWLPLLIAWSEWGNC	CAA35447	UL14
PFHELRTWEIMEHMR	CAA35360	UL86(MCP=majorcapsidprotein)
LMIPKDMYLTWEETR	CAA35405	UL46
FRVEENQCWFHMGML	CAA35314	US3
YHGNCELCGWNGYLR	CAA35434	UL1
MGCDVHDPSWQCQWG	AAA85872.1	UL133Toledo
ETFSQPMHRAMAYVC	CAA35384	UL69
MACSYYDNVVLRELY	CAA35411	UL52
VRATAGRWLPLCWPP	CAA35427	UL28
YPAPEHVHRWSYLCP	CAA35316	UL114
WKPACPDEEPDRCWT	AAA85880.1	UL141Toledo
GWLHRHFPWMFSDQW	CAA35278	US11

12 month after HSCT

Peptide	UL-ORF	
FRVEENQCWFHMGML	CAA35314	US3
VFPCFVPEPWQLMNL	CAA35387	UL72
PFHELRTWEIMEHMR	CAA35360	UL86(MCP=majorcapsidprotein)
HALALWMDWADVRSC	CAA35367	UL93
ACRDWDSMHCTPFWS	AAA85887.1	UL148Toledo
FIALIVVCIMGWWKL	CAA35440	UL7
GWLHRHFPWMFSDQW	CAA35278	US11
AAHDVWCNCGDWQGH	CAA35312	US1
VRATAGRWLPLCWPP	CAA35427	UL28
WCCCMDWKAHVEYAH	CAA35312	US1
GNARLDALMSASEWW	CAA35404	UL45
FFGMCQLWKDWVTNA	CAA35419	UL20
AGRWLPLCWPPLHGI	CAA35427	UL28
CVWCTGLAWLMAWNM	CAA35279	US12

GQNLKYQEFFWDAND	CAA35357	UL83(pp65)
PPDCSPPPYRPPYCL	CAA74074	UL42rev
RSAHFRVEENQCWFH	CAA35314	US3
KNCSRTDVWHDIEWI	AAA85895.1	UL153Towne
QWGRYENGSTPVLWC	CAA35439	UL6
SGYLFFGMCQLWKDW	CAA35419	UL20

24 months after HSCT

Peptide	UL-ORF	
MGCDVHDPSWQCQWG	AAA85872.1	UL133Toledo
PFHELRTWEIMEHMR	CAA35360	UL86(MCP=majorcapsidprotein)
LEMVYPAPEHVHRWS	CAA35316	UL114
VFPCFVPEPWQLMNL	CAA35387	UL72
EPHGQWEFMFREQRG	CAA35311	IRS1
VSHETLERYRVCEPH	CAA35340	UL105
QWGRYENGSTPVLWC	CAA35439	UL6
FRVEENQCWFHMGML	CAA35314	US3
RSAHFRVEENQCWFH	CAA35314	US3
YEPAHLAASDLLNWY	CAA35406	UL47
YPAPEHVHRWSYLCP	CAA35316	UL114
HVSRNMSWRVVWELC	CAA35367	UL93
PSHVTSAFVPSVYMP	CAA35269	TRS1part
PWQLMNLPPPNEHRF	CAA35387	UL72
EDGMVSPERPAFMEH	CAA35456	TRL8
FVPEPWQLMNLPPPN	CAA35387	UL72
TMRGGGWREDVLMDR	CAA35392	UL77
GWLHRHFPWMFSDQW	CAA35278	US11
AAHDVWCNCGDWQGH	CAA35312	US1
HALALWMDWADVRSC	CAA35367	UL93

Supplementary Table S3B. Commonly (4/4 patients) and individually CMV epitopes recognized in serum from patients at 6, 12 and 24 month after HSCT. The entire set of peptide epitopes is listed.

Peptides recognized at 6 month after HSCT. 4 / 4 patients, light blue, 3 / 4 patients dark blue; 2 / 4 patients light yellow and 1 / 4 patients, green.

Peptide	UL-ORF

PPDCSPPPYRPPYCL	CAA74074	UL42rev
VFPCFVPEPWQLMNL	CAA35387	UL72
GNARLDALMSASEWW	CAA35404	UL45
ACRDWDSMHCTPFWS	AAA85887.<br>1	UL148Toledo
QWGRYENGSTPVLWC	CAA35439	UL6
EPHGQWEFMFREQRG	CAA35311	IRS1
SSFWYHGNCELCGWN	CAA35434	UL1
HALALWMDWADVRSC	CAA35367	UL93
LWLPLLIAWSEWGNC	CAA35447	UL14
PFHELRTWEIMEHMR	CAA35360	UL86(MCP=majorcapsidprotein)
LMIPKDMYLTWEETR	CAA35405	UL46
FRVEENQCWFHMGML	CAA35314	US3
YHGNCELCGWNGYLR	CAA35434	UL1
MGCDVHDPSWQCQWG	AAA85872.<br>1	UL133Toledo
ETFSQPMHRAMAYVC	CAA35384	UL69
MACSYYDNVVLRELY	CAA35411	UL52
VRATAGRWLPLCWPP	CAA35427	UL28
YPAPEHVHRWSYLCP	CAA35316	UL114
WKPACPDEEPDRCWT	AAA85880.<br>1	UL141Toledo
GWLHRHFPWMFSDQW	CAA35278	US11
HVSRNMSWRVVWELC	CAA35367	UL93
DSLMEFVTRGMTRCH	CAA35422	UL23
YLLSYWESRTDHVPC	CAA35409	UL50
EAEREYLYRDLHSKW	CAA35406	UL47
QVTIPCTVMTHSWPM	AAA85880.<br>1	UL141Toledo
PCNYSSFWYHGNCEL	CAA35434	UL1
FEGSCVSLGWPSQCI	CAA35428	UL29
WCCCMDWKAHVEYAH	CAA35312	US1
LEMVYPAPEHVHRWS	CAA35316	UL114
LTLMNNCVCDGAVWN	CAA35273	US6
GDIGVFPCFVPEPWQ	CAA35387	UL72
AGRWLPLCWPPLHGI	CAA35427	UL28
FFGMCQLWKDWVTNA	CAA35419	UL20
PAALGGCCCAAGGDW	CAA35338	UL102
QRSYFYCEYSDTDRL	CAA35413	UL54
HLYTVNCEASYSHDQ	CAA35321	UL119
RDGEWIICFCCNGRY	CAA35368	UL94
AWRRVADDSHDLWCC	CAA35312	US1
IRLYDWSEINDWRVM	CAA35422	UL23
FKPEDVKAWSHYLCC	CAA35423	UL24
PLFVDDYRATDDEWT	CAA35360	UL86(MCP=majorcapsidprotein)
VKNLTMNMTEFPQYY	CAA35389	UL74(gO)
TMNMTEFPQYYILAG	CAA35389	UL74(gO)

SVALTSLCHLLCYWC	CAA35447	UL14
LKDLYAAFCECGDGR	CAA35426	UL27
EADRALREFLEAPWE	CAA35406	UL47
FERDYVDEIVEGAWF	CAA35427	UL28
SGYLFFGMCQLWKDW	CAA35419	UL20
LAPGPFHELRTWEIM	CAA35360	UL86(MCP=majorcapsidprotein)
RALAGCMHIHAFAQW	CAA35422	UL23
FPMNVYRHDEVDRWI	CAA35360	UL86(MCP=majorcapsidprotein)
SRLRSINCGWEGERH	CAA35447	UL14
CYKETMIYDMVQYGH	AAA85872.<br>1	UL133Toledo
QTEKWHNVDWIHYEY	CAA35433	TRL14
NVFGQRSYFYCEYSD	CAA35413	UL54
LAPYRFMIAYCPFDE	CAA35384	UL69
WRDMLHDLFCGCHYP	AAA85875.<br>1	UL136Toledo
DLSRWFGENMDEYSG	CAA35454	TRL6
CRDDEEFCHQFLRAY	CAA35426	UL27
EIVRSLVVDARSGQV	CAA35447	UL14
YCVEYLLSYWESRTD	CAA35409	UL50
EQYHHDERGAYFEWN	CAA35314	US3
MLTPVVWSARWDQMF	CAA35281	US14
DDHKAWLDLDESHWV	CAA35311	IRS1
CSFFSWGRHHNATWD	CAA35320	UL118
RSAHFRVEENQCWFH	CAA35314	US3
FASWDLIERIFEHMY	CAA35404	UL45
PFDECPDTHFAFWTH	CAA35395	UL36
PWQLMNLPPPNEHRF	CAA35387	UL72
EHCQNDFFGEFRALH	CAA35428	UL29
LRLSWPNGWFFTYCD	CAA74075	UL43rev
EQVSKRSWDTTVYHR	CAA35416	UL17
LGWAACRDWDSMHCT	AAA85887.<br>1	UL148Toledo
MGESYFLQDEKCVFW	CAA35374	UL59
VPFVPHACPHYAVPF	CAA35430	UL31
YVDEIVEGAWFKHTF	CAA35427	UL28
PSHVTSAFVPSVYMP	CAA35269	TRS1part
LPELDREQWERPRWD	CAA35311	IRS1
QELQYLVEEQRRRNQ	CAA35445	UL13
SDDIVIQISCVCYET	CAA35413	UL54
HLLCYWCSESYRRLN	CAA35447	UL14
SMDTAGMYECVLRNY	AAA85880.<br>1	UL141Toledo
GQNLKYQEFFWDAND	CAA35357	UL83(pp65)
GLQEAYILDKGRRYM	CAA35419	UL20
NKTEDFLHWLLGWGH	CAA35437	UL4(gp48)
HGPALIAWVEEMLRY	CAA35407	UL48(pp212)

YEPAHLAASDLLNWY	CAA35406	UL47
NAYLDTHYRETMDWR	CAA35316	UL114
SPYDVINLFVDDCMR	CAA35422	UL23
FIALIVVCIMGWWKL	CAA35440	UL7
RDPLTTYEYLDDCRD	CAA35426	UL27
HDLRGMMDYHDGLSR	CAA35429	UL30
CSSSSATWLEERDEW	CAA35338	UL102
EVACIFPAHDWPEVS	CAA35447	UL14
SVMTAMIFYRYSETC	CAA35443	UL10
MATLVFPQDMLQCLW	CAA35407	UL48(pp212)
WFRWDGNDSHLICFY	CAA35440	UL7
AYVCCQEYLHPFGFV	CAA35395	UL36
HVHDGDYVYWSFGGG	CAA35444	UL11
VSHETLERYRVCEPH	CAA35340	UL105
FFDQFDTNNAMGTYR	CAA35384	UL69
DLRSNPYPIRWCYCW	CAA35420	UL21
MPVSDSFVCLRPVDF	CAA35407	UL48(pp212)
TCNGSLYTVYKHSNL	AAA85895.<br>1	UL153Towne
WFGENMDEYSGDVWH	CAA35454	TRL6
TVCLLCELMACSYYD	CAA35411	UL52
FVAGHGETDFYMNWT	AAA85887.<br>1	UL148Toledo
DLTYQRLIYWACTLM	CAA35406	UL47
HSLTEIDLEHCQNDF	CAA35428	UL29
SDFDADCWCMWGRFG	CAA35449	TRL1
PMEITRYVHRNEGRC	CAA35291	US24
EDGMVSPERPAFMEH	CAA35456	TRL8
PVADYMFAQSSKQYG	CAA35392	UL77
WDSMHCTPFWSTDLE	AAA85887.<br>1	UL148Toledo
RWMTVMRGYSECGDG	P16832	UL115(gL)
TLIDDYFLLPAGWAC	CAA35426	UL27
WPDGSYRDWEFLARD	CAA35367	UL93
EIDLEHCQNDFFGEF	CAA35428	UL29
SFVAGYEGFGWDGET	CAA35417	UL18
SGAYTEHVYECDLSC	CAA35434	UL1
VRQQDAFICTDYVYC	CAA35424	UL25
GDRLEVACIFPAHDW	CAA35447	UL14
DWIHYEYPTHKMCEL	CAA35433	TRL14
TDYYRTMTTFVHQSH	CAA35444	UL11
VIDCAPFHGVWAEQG	CAA35338	UL102
CLRGFYSVFLRHCDV	CAA35374	UL59
LDAFLLNWLHHGLDL	CAA35407	UL48(pp212)
TAFAHEYHNWLRSPF	CAA35360	UL86(MCP=majorcapsidprotein)
LPLCWPPLHGIMLGD	CAA35427	UL28
RIYTSLLDECACRDF	CAA35413	UL54

TDTVYCVEYLLSYWE	CAA35409	UL50
FSCRDCESSASWLSS	CAA35415	UL15
CFMAMGESYFLQDEK	CAA35374	UL59
GYDQLAARHFADYVD	CAA35354	UL80A
RLPGEDSWYDLDETF	CAA35269	TRS1part
MRCQTPDYEDMLCYS	CAA35384	UL69
LDTHYRETMDWRLCG	CAA35316	UL114
MEDYRTFAGTLSRHP	CAA35352	UL79
KNCSRTDVWHDIEWI	AAA85895.<br>1	UL153Towne
TWTLHGMCISICYYE	CAA35448	UL16
LCWHRVEGGISGPRG	CAA35358	UL84
TINWYLQRSMRDDNW	CAA35314	US3
QYTLNATVEWYNKSE	CAA35419	UL20
YAVQLHAETTRTWRW	CAA35449	TRL1
TMRGGGWREDVLMDR	CAA35392	UL77
DMLQCLWLELKPQYA	CAA35407	UL48(pp212)
ERLNMSAYNVMHLHT	CAA35336	UL100(gM)
GMIRCDPVHESICAR	CAA35428	UL29
TSLCHLLCYWCSESY	CAA35447	UL14
NYFLCQVCLYELDED	CAA35362	UL88
DAFICTDYVYCALRL	CAA35424	UL25
YADNDDYGLYVDWCV	CAA35341	UL104
HFADYVDPHYPGWGR	CAA35354	UL80A
LCAWTFGLAGPCAAW	CAA35420	UL21
TTLPVWTPECKGWTY	CAA35396	UL37
DFLHWLLGWGHKSIC	CAA35437	UL4(gp48)
SCYIAGGRWRFEDGG	CAA35447	UL14
FFAKGMIRCDPVHES	CAA35428	UL29
LINVTEEYTDYYRTM	CAA35444	UL11
WKPSRLPGEDSWYDL	CAA35269	TRS1part
GLPRCEWFERTIYQE	CAA35404	UL45
FFLAPSMAQFWHGAI	CAA35397	UL38
VHDPSWQCQWGVPTI	AAA85872.<br>1	UL133Toledo
LTVFTVYLLSHLPSQ	CAA35390	UL75(gH)
FSPCHQCQTYYVECE	CAA35278	US11
ETGIFTFIMEDYRTF	CAA35352	UL79
QPLDGWSWIASPWKG	CAA35431	UL32(pp150)
NMRYCVRFASDSDFQ	CAA35387	UL72
VFAYDAQEDCLYELA	CAA35428	UL29
RWLIRCCELYGEYER	AAA85877.<br>1	UL138Toledo
DLPLWCLCRLKCERH	CAA35408	UL49
RPIIGDTGGSSSSQR	CAA35424	UL25
LNAVHVHDGDYVYWS	CAA35444	UL11
KDLDDSFDYLVERCQ	CAA35351	UL78

RVMVGSNHVEPLGWL	CAA35422	UL23
RSTSYHETGVYQMWV	CAA35319	UL117
AWLDLDESHWVLGDS	CAA35311	IRS1
NMTSVWRFEGQFNPH	CAA35274	US7
RWQYQELQYLVEEQR	CAA35445	UL13
TVNRTCDLLTPPPWY	CAA35398	UL39
PSLKDSCHLCAWTFG	CAA35420	UL21
GLADWNVVRCRGTGF	CAA35449	TRL1
HQDSWRDMLHDLFCG	AAA85875.<br>1	UL136Toledo
DYFLLPAGWACANPR	CAA35426	UL27
MALVDQESCLRGFYS	CAA35374	UL59
FPLPTAFAHEYHNWL	CAA35360	UL86(MCP=majorcapsidprotein)
SPSRCFMAMGESYFL	CAA35374	UL59
HDSGLYVCICDPSYE	CAA35397	UL38
SATWLEERDEWVRSL	CAA35338	UL102
QRPMVQYDDYWNAVM	CAA35395	UL36
FQGWAYAVYHQGDMA	CAA35399	UL40
ELCRHALALWMDWAD	CAA35367	UL93
SCFVHDLRGMMDYHD	CAA35429	UL30
FDGCYHSEAYRMLFQ	CAA35393	UL34
KFSSIWRGLRDAWTH	CAA35427	UL28
HCTPFWSTDLEQMTD	AAA85887.<br>1	UL148Toledo
AGMYECVLRNYSHGL	AAA85880.<br>1	UL141Toledo
ASNNPCNYSSFWYHG	CAA35434	UL1
MLSHYGTVYVYDWET	CAA35289	US22
AEAALKDLYAAFCEC	CAA35426	UL27
DGTRYQMCVMKLESW	CAA35332	UL130(viralentry)
WHNHGNKWTLDTCYY	CAA35444	UL11
AFYWRLFLQSQHVEA	CAA35434	UL1
LYCGPRSFCARDTCF	CAA35369	UL95
YENGSTPVLWCTLWG	CAA35439	UL6
AFFTRHWGAEAYEPL	CAA35352	UL79
AWIASKNVQYEFMGL	CAA35430	UL31
LLIAWSEWGNCCLDA	CAA35447	UL14
LYVCICDPSYEFLQM	CAA35397	UL38
LDALMSASEWWVESA	CAA35404	UL45
SGLLLLFTCCFCCFW	AAA85878.<br>1	UL139Toledo
CLTERFDPHEGAWER	CAA35291	US24
EEDEWLREIQGATYQ	CAA35445	UL13
YATSLRRLDEELRHR	CAA35424	UL25
HYYSTSPLNGMCLDC	AAA85895.<br>1	UL153Towne
LHDLFCGCHYPEKCR	AAA85875.	UL136Toledo

	1	
CLGGYCDLIREKEVH	CAA35404	UL45
LLLYRDGEWIICFCC	CAA35368	UL94
ASELRPGSGGWPEHA	CAA35312	US1
LHQGFYLMDELRYVK	CAA35390	UL75(gH)
DEDPETYMGFLWDIP	CAA35395	UL36
YHETGVYQMWVSGAT	CAA35319	UL117
GAMYLWTDHIYSDSL	CAA35422	UL23
EGFTRSGENETFLWY	CAA35440	UL7
LGFADWSQTLIDDYF	CAA35426	UL27
RIHRFWECRLRVWWL	AAA85875.<br>1	UL136Toledo
DTAPRWMTVMRGYSE	P16832	UL115(gL)
LGCILDHQDGWGDHC	CAA35384	UL69
AYGAVFAYDAQEDCL	CAA35428	UL29
LGASGAMYLWTDHIY	CAA35422	UL23
HFHTMRDYQRPMVQY	CAA35395	UL36
FYILCYDLFTSCGNR	CAA35368	UL94
IFNSYATTAWPMQCE	CAA35432*	UL33
DYTTGVCVMRRLINH	CAA35443	UL10
AAHDVWCNCGDWQGH	CAA35312	US1
DKGRRYMYLFSVSCA	CAA35419	UL20
FCTRDLADLCVRRDY	CAA35428	UL29
ATADIAEKMWAENYE	AAA85880.<br>1	UL141Toledo
FVFPYLVLPNCCQVS	CAA35429	UL30
WHSRGSTWLYRETCN	CAA35414	UL55(gB)
DRWCLCNAWRDHALR	CAA35263	US31
YTNTSCSPQFMCINE	CAA35434	UL1
PHVWMPPQTTPHDWK	CAA35390	UL75(gH)
DNEIHNPAVFTWPPW	CAA35357	UL83(pp65)
AVSETLFYYVYTSWC	CAA35372	UL57
AYVLSSMLCVWCTGL	CAA35279	US12
LPFWSTLLPCALRCH	CAA35279	US12
PGQMSAWLRDDVCDL	CAA35384	UL69
GISLLSEFCRVLCCY	CAA35325	UL123(pp72=MIprotein=IE1)
VWSYVGRVCTFYVTC	CAA35351	UL78
STLLPCALRCHAYWL	CAA35279	US12
AHPHHEYLSDLYTPC	CAA35390	UL75(gH)
KYAESDYIFLQDMCP	CAA35396	UL37
AYVNALHDHRLWPPF	CAA35360	UL86(MCP=majorcapsidprotein)
TVWRNLFYVYYELAR	CAA35361	UL87
NYLKHHYDLCFTCDR	AAA85896.<br>1	UL154Towne
ACLLAAYGHALWEGR	CAA35392	UL77
GVEMPEMTWDLDVRN	AAA85875.<br>1	UL136Toledo

LRLVWPDGSYRDWEF	CAA35367	UL93
NGVAWEHRLSSVWRD	CAA35281	US14
MVTNLTVGRYDCLRC	CAA35448	UL16
VLEQAHFVVIGWMEP	CAA35427	UL28
ASVSYALRYDDESWR	CAA35311	IRS1
RNGFRHRDHFHTMRD	CAA35395	UL36
NFPLNSMFYYRDRQE	CAA35408	UL49
AAMQSVRDGLFCLGC	CAA35353	UL80
WAHGLDIVEEDEWLR	CAA35445	UL13
ELPSRDGIRWQYQEL	CAA35445	UL13
PHYTKLPKYDPDEFW	CAA35433	TRL14
DQYVKVYLESFCEDV	CAA35357	UL83(pp65)
VYYHVVDFERLNMSA	CAA35336	UL100(gM)
AVETLHCMRYLTSSL	CAA35423	UL24
RFMIAYCPFDEQSLL	CAA35384	UL69
YFRIPQKLWLLWQHD	CAA35444	UL11
RTDVWHDIEWIKYGP	AAA85895.1	UL153Towne
TENGSFVAGYEGFGW	CAA35417	UL18
FWFRCPRRFCFSPLD	CAA35427	UL28
PVDDWLNFRVDLFGD	CAA35394	UL35
PFTELGWAACRDWDS	AAA85887.1	UL148Toledo
GEVREFKHLVYFHHA	CAA35386	UL70
QNYTCRVTHGNWTVE	CAA35417	UL18
LSLSSFAAWWTMLNA	CAA35388	UL73(gN)
NVVSYVCEEHLHSFT	CAA35363	UL89
LEDDDEYDELWFPLY	CAA35318	UL116
GAVFGYCPLDGHVYP	CAA35311	IRS1
PYFVFLAYVYSMDCL	CAA35408	UL49
GYRSQSVLTWTHECN	CAA35417	UL18
YHECSQTMLHEYVRK	CAA35333	UL97
ANDIYRIFAELEGVW	CAA35357	UL83(pp65)
FAYDSGILFFLAPSM	CAA35397	UL38
FMTPKWDVFAYDSGI	CAA35397	UL38
RFTEDTFVETFCDFL	CAA35426	UL27
VDISHFLKKQHKKKM	CAA35381	UL66
RSMLLSREEELVPWS	CAA35438	UL5
VYSFFERDYVDEIVE	CAA35427	UL28
IDDFRYESIGPVDRS	CAA35428	UL29
LASIHPGETWTLHGM	CAA35448	UL16
YLDDCRDDEEFCHQF	CAA35426	UL27
LRGDSCFVHDLRGMM	CAA35429	UL30
CELCGWNGYLRNVTH	CAA35434	UL1
RVVSYCQNNVKMVDR	CAA35411	UL52
FVPEPWQLMNLPPPN	CAA35387	UL72
QFTTVAMVHYHQEYT	AAA85877.	UL138Toledo

	1	
CLKQDCDQSWLLEYS	CAA35411	UL52
HVPTQPLDGWSWIAS	CAA35431	UL32(pp150)
LPYMYKMDQDDGYFM	CAA35386	UL70
LWYVNSFWRSRELSY	CAA35367	UL93
AEAFCTSYGFFPGEI	CAA35417	UL18
AGVSRLREVWDVQHR	CAA35367	UL93
LCRGLRRVWMTVWAS	CAA35386	UL70
GNKWTLDTCYYVYVT	CAA35444	UL11
VTSVIFIHCYETSHP	CAA35282	US15
EETVWSLCPPNRECY	CAA35404	UL45
KYQEFFWDANDIYRI	CAA35357	UL83(pp65)
ADVNSVIRCLGGYCD	CAA35404	UL45
VMLDYYWIQLITNND	CAA35419	UL20
YPRGYTLFVCDVEET	CAA35425	UL26
IYGTLDMSSLYYNET	CAA35389	UL74(gO)
VLRFFGATEHGYSIC	CAA35413	UL54
GEQGCGNFTTFNPMF	CAA35389	UL74(gO)
ATVQGQNLKYQEFFW	CAA35357	UL83(pp65)
PVIADIVDKCLNMWE	CAA35340	UL105
HPHASPRSDHTLFPV	CAA35449	TRL1
SDEGYVFCYVREDTA	CAA35423	UL24
YEDDDYYYYREDEPR	CAA35275	US8
ECACRDFILPNHYSK	CAA35413	UL54
RRDHVWSYVGRVCTF	CAA35351	UL78
QALTELEYQAMGAVW	CAA35311	IRS1
VAGVAADGSVLCYEI	CAA35425	UL26
DAVVRRCLREYVGDY	CAA35385	UL71
YEYPTHKMCELGNYH	CAA35433	TRL14
PRAALWAREPHGQWE	CAA35311	IRS1
AADGSVLCYEISREN	CAA35425	UL26
YAAFCECGDGRDNGG	CAA35426	UL27
HDERGAYFEWNIGGH	CAA35314	US3
IGQRGGIYCYDDLRD	CAA35395	UL36
LVFMLWGADAHTCEY	CAA35316	UL114
GVPLPPDPQHFRWLN	CAA35426	UL27
IYVAMSRVTDPEHLM	CAA35340	UL105
LSAFCYAAPATWFHH	CAA35282	US15
STWDCLSVAWIRHYN	CAA35460	TRL12
YHQGDMALMTLDVYC	CAA35399	UL40
VQDSVSRDLGFADWS	CAA35426	UL27
RLPPLWLPLLIAWSE	CAA35447	UL14
TEEYTDYYRTMTTFV	CAA35444	UL11
GGIYCYDDLRDCVYE	CAA35395	UL36
TQTGMGGGRLPPLWL	CAA35447	UL14
LYVTYIYYTLCTPNC	CAA35378	UL63

RSVSLRIKRELLCLH	CAA35426	UL27
CYLQHQLQSISELCY	CAA35424	UL25
EAAEETAAGEASAVA	CAA35447	UL14
YTLFFYILCYDLFTS	CAA35368	UL94
GLFAVYSFFERDYVD	CAA35427	UL28
EYLYRDLHSKWGVHL	CAA35406	UL47
VVWSARWDQMFSYLA	CAA35281	US14
VGLRLHDCAAFESCC	CAA35438	UL5
DYWNAVMLYRGDVES	CAA35395	UL36
LGGSPACTFTFGSWN	CAA35427	UL28
YDDDEELTRLLAVWD	CAA35334	UL98
REHPELELKYLNMMK	CAA35412	UL53
PQLLERGLLHSYFED	CAA35425	UL26
LDIVEEDEWLREIQG	CAA35445	UL13
TEHSYALWASLWSRC	CAA35386	UL70
PHAGVIDCAPFHGVW	CAA35338	UL102
WSLCPPNRECYFPTV	CAA35404	UL45
CPLVFQGWAYAVYHQ	CAA35399	UL40
CNRLCDALYFCYTQA	CAA35394	UL35
MSVKGVEMPEMTWDL	AAA85875.<br>1	UL136Toledo
VTPCLHQGFYLMDEL	CAA35390	UL75(gH)
VKAAWSLKELHTHQL	P19893	UL122(IE2)
LGDTQYFGVVRDHKT	CAA35427	UL28
INHIVNHDLFRWSVM	CAA35443	UL10
VAPPMWEIHHINKFA	CAA35414	UL55(gB)
RPEGYTLFFYILCYD	CAA35368	UL94
YLETVGGMRQLLFNK	CAA35384	UL69
HPTTGAYFDNGWKWT	CAA74074	UL42rev
REYVGDYMSRIILCC	CAA35385	UL71
VCLISDEGYVFCYVR	CAA35423	UL24
FCDSDIEDFERECYR	CAA35424	UL25
GFYQIRKPPWLMEQP	CAA35311	IRS1
GRCPHENFPFWDRFG	CAA35293	US26
LYAAEQLHEQLDRFL	CAA35406	UL47
SWGRHHNATWDLFTY	CAA35320	UL118
LRRFLRGDSCFVHDL	CAA35429	UL30
GFLLYRHHERLFPEC	CAA35372	UL57
EVNTISVRYLYHADH	CAA35405	UL46
WLFRRLFFPREDSEP	CAA35416	UL17
CVWCTGLAWLMAWNM	CAA35279	US12
IVYDYDGPETRPGIY	CAA35405	UL46
YVYTSWCQSLRFSEP	CAA35372	UL57
GLRRYLRRFEGSCVS	CAA35428	UL29
YEPVRDYMTYMNLAE	CAA35369	UL95
RHHTLMSTTCRCWSS	CAA35380	UL65

RCCELYGEYERRFAD	AAA85877.<br>1	UL138Toledo
RLLPMVLLAAYCYCV	CAA35437	UL4(gp48)
NNHHHHHHHHNAVTD	CAA35315	UL113
VDCYWRKLFGGDDPG	CAA35338	UL102
KNTVSGYLFFGMCQL	CAA35419	UL20
KGATEAEREYLYRDL	CAA35406	UL47
AFLGFWSIFTVCFLC	AAA85880.<br>1	UL141Toledo
ALDEEDLEQYLLVWS	CAA35341	UL104
WLLGWGHKSICSFFP	CAA35437	UL4(gp48)
RTMAFLRFERYDTDY	CAA35408	UL49
QKYFSLDNFLHDYVE	CAA35386	UL70
GAMYYGSGCRFDTVE	AAA85875.<br>1	UL136Toledo
PETQFYYTRHEVFNE	CAA35386	UL70
YFYIGRADAEDCWKP	AAA85880.<br>1	UL141Toledo
FRLAAFFTRHWGAEA	CAA35352	UL79
QARVRDSHDRWCLCN	CAA35263	US31
ETFWVLGSNRKNDVY	CAA35269	TRS1part
NYASLLAFSHHPEFP	CAA35406	UL47
FTFIMEDYRTFAGTL	CAA35352	UL79
MPAETFTCPKDKRPW	CAA35449	TRL1
LENCAFCQSALLEYD	CAA35390	UL75(gH)
EFARVGLRAVETLHC	CAA35423	UL24
CYDLFTSCGNRCDIP	CAA35368	UL94
HGMCISICYYENVTE	CAA35448	UL16
QNSAECWSVRETKRC	CAA35420	UL21
LHPFGFVEGPGFMRY	CAA35395	UL36
SSVFETRASGRFFHR	CAA35427	UL28
WHNVDWIHYEYPTHK	CAA35433	TRL14
ELIMFGVIEAWEEAS	CAA35397	UL38
SICVNVFGQRSYFYC	CAA35413	UL54
LHPFFDFTHCQENSE	CAA35360	UL86(MCP=majorcapsidprotein)
AETLRGFIRQGSFWF	CAA35427	UL28
QKLGWCLADDIHTSF	CAA35405	UL46
FDMLNVVSYVCEEHL	CAA35363	UL89
FKTTVTSPNYPELCY	CAA35360	UL86(MCP=majorcapsidprotein)
GIGWYEPEVSMAYIY	CAA35322	UL120
NLFVFCTERDYRKFH	CAA35393	UL34
FYSVFLRHCDVEPAI	CAA35374	UL59
ERLFPECGLPCLQFW	CAA35372	UL57
LHGPAPLSCNVTQWG	CAA35439	UL6
LLPRQYTLNATVEWY	CAA35419	UL20
WTHNTEVMKFKETSF	CAA35395	UL36
FWECRLRVWWLSDAG	AAA85875.	UL136Toledo

	1	
VDCNLSMMWMRFFVC	CAA35313	US2
RDYEGLRRYLRRFEG	CAA35428	UL29
WMTVWASLFGYTHPD	CAA35386	UL70
SYVVCSQHGAFFPAR	AAA85892.1	UL151Toledo
ARRLLPELDREQWER	CAA35311	IRS1
TEYTCSFFSWGRHHN	CAA35320	UL118
EWKLHAALFPYRALD	CAA35341	UL104
PPDPQHFRWLNAGAF	CAA35426	UL27
VAFTWQHNESVVDLW	CAA35448	UL16
LDMSSLYYNETMFVE	CAA35389	UL74(gO)
RSRHETGIFTFIMED	CAA35352	UL79
STCFLTRLNNAPFDM	CAA35363	UL89
IIVIDECGLMLRYML	CAA35340	UL105
CAEGTVYPSEWMVVK	CAA35371	UL56
PSTLETFPDLFCLPL	CAA35390	UL75(gH)
LLPRDVVEHWLHAQG	CAA35353	UL80
HPSEQNQNFNLQIHP	AAA85896.1	UL154Towne
TGPRHVIWPGTSVLW	CAA35362	UL88
DLAGFFAKGMIRCDP	CAA35428	UL29
SAMYYFYVSPYTTEE	CAA35340	UL105
FYRAFRSGRFDLCTD	CAA35391	UL76
MMTDRTERRRRLTHA	CAA35342	UL106
CYASELCDESVRRFV	CAA35404	UL45
AEVCVRTVVDCYWRK	CAA35338	UL102
RTMTTFVHQSHNWHN	CAA35444	UL11
DKVYVLGLSFGEFFE	CAA35427	UL28
PVKLEFEKVFVSLMM	CAA35413	UL54
QDEKNVTCQLTFWEA	CAA35414	UL55(gB)
VADDSHDLWCCCMDW	CAA35312	US1
DAFYTGLSVWRGGEP	CAA35430	UL31
HDWPEVSIRVHLCYW	CAA35447	UL14
EWNIGGHPVTHTVDM	CAA35314	US3
EWHTNYLKHHYDLCF	AAA85896.1	UL154Towne
HNEEPATFFCESDDA	CAA35368	UL94
CPPNGNCEFPTCFTL	CAA35443	UL10
YFIGLVSVYECVPDA	CAA35427	UL28
DLADLCVRRDYEGLR	CAA35428	UL29
LHSYFEDVERAAQGR	CAA35425	UL26
PVCLAPDHHLSKWLD	AAA85894.1	UL152Towne
LMNLTYLWYLGDYGA	CAA35277	US10
KSNFTFCTPPSPPCC	CAA35330	UL128(viralentry)
CCYGIITTLRPGLWC	CAA35400	UL41

AQGTDLIRFERNIIC	CAA35414	UL55(gB)
TLDTCYYVYVTQNGT	CAA35444	UL11
FVIPVTSVIFIHCYE	CAA35282	US15
PYLYRLNFCLIDTCL	CAA35378	UL63
VVSQTATRVRTWFVE	CAA35445	UL13
VAPDCVLSYVESRFH	CAA35372	UL57
QFVLKEVEFRCRHTF	CAA35363	UL89
LIAYLRYYHQDSWRD	AAA85875.1	UL136Toledo
DSCHLCAWTFGLAGP	CAA35420	UL21
MDLPTTVVRKYWTFA	CAA35315	UL113
IYNVSESSGGTYYQK	CAA35439	UL6
PMLRDRDHDDAPPTY	CAA74074	UL42rev
LRPATFGLETWAMYT	CAA35399	UL40
VIQISCVCYETGGNT	CAA35413	UL54
CWTVIQRYRLPGDCY	AAA85880.1	UL141Toledo
LLACEDTAARCAYVE	CAA35428	UL29
VDPASRERLLCFSPA	CAA35355	UL81
DVIYWAVSQNYDYAL	CAA35424	UL25
EDSWYDLDETFWVLG	CAA35269	TRS1part
SHDLWCCCMDWKAHV	CAA35312	US1
VWDVQHRVRLRVLWY	CAA35367	UL93
LLPYGDRLEVACIFP	CAA35447	UL14
YLYRQNLQRLNENHR	CAA35424	UL25
KRPPETFSQPMHRAM	CAA35384	UL69
WKAWVGLWTSMGPLI	CAA35313	US2
SRERLLCFSPACFSH	CAA35355	UL81
GYEGFGWDGETLMEL	CAA35417	UL18
AWFKHTFAGMYELSQ	CAA35427	UL28
LISISFLLVSFINCK	CAA35389	UL74(gO)
RRVRIFMIVCVLWCV	CAA35331	UL129(viralentry)
LSEFCRVLCCYVLEE	CAA35325	UL123(pp72=MIprotein=IE1)
REFMLARDLLALWRL	CAA74075	UL43rev
FLRVVRQQDAFICTD	CAA35424	UL25
GGCCCAAGGDWLSAV	CAA35338	UL102
ELQMMQDWVVERCNR	CAA35394	UL35
FTFGSWNVAEADEAN	CAA35427	UL28
PLLRHLDKYYAGLPP	P16832	UL115(gL)
RDISYMGDSLTAFLF	CAA35336	UL100(gM)
FCSSSPYQRLETRDW	CAA35295	UL132
TLILAARDADEWFRH	CAA35311	IRS1
GLNAACAVYDHRLAF	CAA35408	UL49
VLGLSFGEFFENGLF	CAA35427	UL28
NFMTDFKKWLDGGFS	CAA35363	UL89
ESYLTTIWLLNCADN	CAA35419	UL20
ADSLPQLLERGLLHS	CAA35425	UL26

TWEKGDALCVLPPLF	CAA35359	UL85
REDYAQLSDVIYWAV	CAA35424	UL25
SSMLCVWCTGLAWLM	CAA35279	US12
EQLHEQLDRFLRHQH	CAA35406	UL47
LSYDDHEVELYRALD	CAA35367	UL93
TCDGITPDVIYEVCM	CAA35368	UL94
EEMFEALRIYYGDDP	CAA35360	UL86(MCP=majorcapsidprotein)
ETYMGFLWDIPSCDR	CAA35395	UL36
RNNLDNGSDRRLPEA	CAA35367	UL93
GSVIDLPLWCLCRLK	CAA35408	UL49
ARWEALRADMLEFGL	CAA35404	UL45
IFIHCYETSHPSNIG	CAA35282	US15
PAVYTCVDDLCRGYD	P16832	UL115(gL)
IIYIQNSAECWSVRE	CAA35420	UL21
TVYFDAAYVHAPGIC	CAA35387	UL72
GWNGYLRNVTHYYTN	CAA35434	UL1
NIEVSRPSVLCCFQE	CAA35356	UL82(pp71)
HATCVLYFVAEEVHT	CAA35432*	UL33
GNEQVSRIACTSCED	CAA35412	UL53
WPNGWFFTYCDLLRV	CAA74075	UL43rev
HYEVNGTELRCRCLH	AAA85885.<br>1	UL146Toledo
PHDAATFYCPFLYPS	CAA35332	UL130(viralentry)
GDYVYWSFGGGGANR	CAA35444	UL11
IPLCTGVIQKLGWCL	CAA35405	UL46
TLVECYVMHGREPVR	CAA35334	UL98
CDPRMFLRLTHPELC	CAA35333	UL97
CTFYVTCLMLFVPYY	CAA35351	UL78
DTEVQRIEENLEGVR	CAA35431	UL32(pp150)
DWSQTLIDDYFLLPA	CAA35426	UL27
ARRRYHLRRDYWLTD	CAA35447	UL14
FDSAYQPAESMLFSE	CAA35372	UL57
PGSRTLEDALNDMYL	CAA35394	UL35
YSVIRVSTIRLYDWS	CAA35422	UL23
LYTELHPFFDFTHCQ	CAA35360	UL86(MCP=majorcapsidprotein)
YWCSESYRRLNTEEE	CAA35447	UL14
YLRQLCSMTEELYLR	CAA35426	UL27
LGAMDADEPLFVDDY	CAA35360	UL86(MCP=majorcapsidprotein)
MLRLLSVEEICEEHT	CAA35424	UL25
FPHLGYPCVYYHVVD	CAA35336	UL100(gM)
EVSIRVHLCYWPEIV	CAA35447	UL14
PTAATGSLDYRWLGC	CAA35351	UL78
DCLYELASDLAGFFA	CAA35428	UL29
HRRAVNLSTLNSLWW	CAA35389	UL74(gO)
CGGPIGPRELSKWMT	CAA35292	US25
QPASAAGTGFGIMDY	CAA35351	UL78

KEKLHCQQNFPLNSM	CAA35408	UL49
YMHDSDDVLFALDPY	CAA35390	UL75(gH)
NQHVCIVADSLMEFV	CAA35422	UL23
GQYECRPQLQLPWVP	CAA35274	US7
DTFIERTPCEQAAYA	CAA35340	UL105
SFPFATADIAEKMWA	AAA85880.1	UL141Toledo
FLRFERYDTDYLLRR	CAA35408	UL49
HSLKILHSRILCEWH	AAA85896.1	UL154Towne
LLAFSHHPEFPSIFA	CAA35406	UL47
TPDPSRLRSINCGWE	CAA35447	UL14
RLTHHASYHANYGAY	AAA85884.1	UL145Toledo
CSQGAYVCCQEYLHP	CAA35395	UL36
FADYVDPHYPGWGRR	CAA35353	UL80
HLFVTDKRFLNRELG	CAA35408	UL49
ESLERFLTQLWENEY	CAA35341	UL104
PHYDLPLICAATWTA	CAA35355	UL81
RPFFSDAPLPYFVPP	CAA35358	UL84
THFAVQYTEEDFEAH	CAA35406	UL47
CCSACYKETMIYDMV	AAA85872.1	UL133Toledo
QNYDYALYASTPALF	CAA35424	UL25
YNHTIDTCKNTVSGY	CAA35419	UL20
LCYLQCCGRWCPTPG	AAA85880.1	UL141Toledo
VLTCGLQEAYILDKG	CAA35419	UL20
ERLFRDPLTTYEYLD	CAA35426	UL27
VGEFMVRDPLLRDPR	CAA35424	UL25
PRLTEVYQTLRDYNV	CAA35424	UL25
SLLDECACRDFILPN	CAA35413	UL54
HLHGLPVNPHDPYLE	CAA35384	UL69
DIEDFERECYRVSVA	CAA35424	UL25
VGCTPDMGRCLCYVP	CAA35368	UL94
AGRLYFIGLVSVYEC	CAA35427	UL28
RPHPQQQQHHHPGPP	CAA35352	UL79
GFQRTFIDPLWDYLD	CAA35389	UL74(gO)
RFFFRLTGQDEAHSF	CAA35339	UL103
SNVTIKGNSTWDCLS	CAA35460	TRL12
ETFPDLFCLPLGESF	CAA35390	UL75(gH)
IAHTSPFESYVRWEE	CAA35396	UL37
VLLTMAYYLFEGQYS	CAA35386	UL70
SINCGWEGERHRVVH	CAA35447	UL14
LHDCAAFESCCYDIT	CAA35438	UL5
AFSFYLLTSAQRGYD	CAA35430	UL31
STELLDVMQKYFSLD	CAA35386	UL70

IIDETLSYMKYHHLH	CAA35384	UL69
YVRYSHRLHTYAVCE	CAA35338	UL102
PVYVGGFLARYDQSP	CAA35353	UL80
YASYTIDDPFDECPD	CAA35395	UL36
LHFTMFDSGVDRDYA	CAA35290	US23
DRPMPVVPEECYDQR	CAA35311	IRS1
LAEETARFVELAGCW	CAA35404	UL45
HVIWPGTSVLWAPDV	CAA35362	UL88
IPEGFFGITFYKCLD	CAA35387	UL72
LQDFDVQHLRRLNEC	CAA35424	UL25
FDGQHFFTYHVNSSD	CAA35417	UL18
CPRGFELLPEFTEEE	CAA35419	UL20
LPHEDAFYTGLSVWR	CAA35430	UL31
ADLAVYHRNQWCHQR	CAA35408	UL49
LLNWLHHGLDLQRMH	CAA35407	UL48(pp212)
QYSTISTVEEYVRSF	CAA35386	UL70
SHNLWCTCGNWQSHV	CAA35263	US31
MAMYTSESERDWRRV	CAA35264	US32
MLGIRAMLVMLDYYW	CAA35419	UL20
MAAMEANIFCTFDHK	CAA35359	UL85
YLYHADHQALTARFF	CAA35405	UL46
HLLGTESDDEETTVW	CAA74073	UL41alt
NYHQTTPRHDICFDC	CAA35433	TRL14
HMYELSLSSFAAWWT	CAA35388	UL73(gN)
ADAVIHASGKQMWQA	CAA35357	UL83(pp65)
VVAYTGAVYACDVRD	CAA35423	UL24
SRERFAPEDFSFQWF	CAA35340	UL105
YLRRFEGSCVSLGWP	CAA35428	UL29
KLISLYVTYIYYTLC	CAA35378	UL63
HCCFQNFTATTTKGY	CAA35388	UL73(gN)
CCWVTLAHAGNPYED	CAA35275	US8
FPRDALLGRLYFISS	CAA35358	UL84
DDVRGFTVFSHAACG	CAA35420	UL21
STPVLWCTLWGSRTR	CAA35439	UL6
VDQCVALVFYYDSGM	CAA35404	UL45
SLMDPLSPSRWEVAL	CAA35420	UL21
RRLQPMVLLGAWQEL	CAA35311	IRS1
TAPVEWKSPDRQIPK	CAA35437	UL4(gp48)
TTTVTCDGFNYTVHK	CAA35461	TRL13
FLPRTPSPSNTVCCI	AAA85891.1	UL150Toledo
KNTADAMERGLIHSF	CAA35360	UL86(MCP=majorcapsidprotein)
ELGDRLYQRFLREWL	CAA35408	UL49
EHPTFTSQYRIQGKL	CAA35357	UL83(pp65)
RVSTIRLYDWSEIND	CAA35422	UL23
NWTLRRSQTHYLEEM	AAA85887.1	UL148Toledo

VASCKFLSVIYYSSC	CAA35432*	UL33
LDGTFHQGCYVAIFC	CAA35417	UL18
LRRVWMTVWASLFGY	CAA35386	UL70
CRVLCCYVLEETSVM	CAA35325	UL123(pp72=MIprotein=IE1)
QYLNTLLITMMAAIW	AAA85887.1	UL148Toledo
NLFYVYYELARDLGS	CAA35361	UL87
SLFNVNDIYELLYFL	CAA35392	UL77
MNLPPPNEHRFFSLR	CAA35387	UL72
DAEDDVVFASELCFY	CAA35362	UL88
NTSHHSVVWQRYDIY	CAA35459	TRL11
DLIERIFEHMYFAAV	CAA35404	UL45
HGPLCPLVFQGWAYA	CAA35399	UL40
WSLKELHTHQLCPRS	P19893	UL122(IE2)
FYCEYSDTDRLREVI	CAA35413	UL54
VEYHHEVTSEFFGRV	CAA35361	UL87
NTMFENASTWTFSFG	CAA35392	UL77
PSPPRYPFLVGWSWG	CAA35453	TRL4
RVAEEWKLHAALFPY	CAA35341	UL104
DSIYFTFNKVFRSMH	CAA35366	UL92
RWHACVPQKCEKSLC	CAA35275	US8
YCWTMFPPMYPVLLL	CAA35420	UL21
SRKTPQPYWPHLYRE	CAA35352	UL79
MKYHHLHGLPVNPHD	CAA35384	UL69
AFRELLACEDTAARC	CAA35428	UL29
EALRIYYGDDPERYN	CAA35360	UL86(MCP=majorcapsidprotein)
TYYVECEPRCLVPWV	CAA35278	US11
GSNHVEPLGWLVSPY	CAA35422	UL23
SPSMGFQRTFIDPLW	CAA35389	UL74(gO)
IGLRRDLLEDFRYIC	CAA35396	UL37
YFYVSPYTTEEMLRE	CAA35340	UL105
HGIMLGDTQYFGVVR	CAA35427	UL28
SVLCYEISRENFVVR	CAA35425	UL26
KFLSVIYYSSCTVGF	CAA35432*	UL33
AMLKTDTVYCVEYLL	CAA35409	UL50
NCTQWSVIYSGFQPP	CAA35417	UL18
FAIVSFKHMGPFEGY	CAA35351	UL78
MMTMWCLTLFVLWML	CAA35417	UL18
CEWFERTIYQEGKFI	CAA35404	UL45
FSFSPGPVVLLWCCL	P16832	UL115(gL)
LPEQVKAFCITPTQW	CAA35407	UL48(pp212)
EDSLDKLIAWMTWLS	CAA35284	US17
NDFFGEFRALHLIGT	CAA35428	UL29
FVDDCMRVFAANNQH	CAA35422	UL23
LCRGDRFRAEMLNNW	CAA35290	US23
LFSKWLDKHNDNRWY	AAA85885.1	UL146Toledo

LRGDEEFIYHAGPLE	CAA35367	UL93
YSGIYYFDSLYTYGW	CAA35442	UL9
AQWRQQVHAAHDVWC	CAA35312	US1
ISICYYENVTEDEII	CAA35448	UL16
VIKDCFLNLLDRWRP	CAA35422	UL23
LRDLKLCDGSYEEGF	CAA35407	UL48(pp212)
GTIEGDIGVFPCFVP	CAA35387	UL72
YCLVSSPSPRHTFDM	CAA74074	UL42rev
MDILTTCVETMCNEY	CAA35325	UL123(pp72=MIprotein=IE1)
YRIFAELEGVWQPAA	CAA35357	UL83(pp65)
LWILSLLAVTLTVAL	P16845	UL22A
TLNDSGAYTEHVYEC	CAA35434	UL1
SLDNFLHDYVETHLL	CAA35386	UL70
RIYRRFYGPYLGVFV	CAA35358	UL84
EVFALMIPKDMYLTW	CAA35405	UL46
ASRGTVFEEETVWSL	CAA35404	UL45
ATAPSFDEAFLTDRL	CAA35407	UL48(pp212)
DCESSASWLSSFTPA	CAA35415	UL15
APPTPETARVQRLLW	CAA35426	UL27
YLCLMPAMTNNRACG	CAA35360	UL86(MCP=majorcapsidprotein)
MAYYLFEGQYSTIST	CAA35386	UL70
AHAKPRPTYVLVTVN	CAA35407	UL48(pp212)
LTSHYSGIYYFDSLY	CAA35442	UL9
HVLRYGYTGIFDDTS	CAA35417	UL18
FFTKNQTSTVCLLCE	CAA35411	UL52
DMFHHDQWKLACIDS	CAA35333	UL97
ALHLIGTVSHATCRY	CAA35428	UL29
RPTRQLVLFMTPKWD	CAA35397	UL38
GNSSESESKTTHAYT	AAA85878.<br>1	UL139Toledo
ISWMANVSAAYPTYL	CAA35417	UL18
TEGQVLLTMAYYLFE	CAA35386	UL70
VAILCYLAYHWHDTF	AAA85877.<br>1	UL138Toledo
YRHHERLFPECGLPC	CAA35372	UL57
RDKAAFTSSVSTRTP	CAA35438	UL5
PIRFLRENTTQCTYN	CAA35390	UL75(gH)
NLPGYEHASEGWRFC	CAA35264	US32
TASRDAADEVWALRD	CAA35431	UL32(pp150)
HLDLRPEELRDPFQI	CAA35358	UL84
MDHTSLYADPFFLKY	CAA35340	UL105
LKLFCYLVSTAWHQR	CAA35407	UL48(pp212)
VTHYYTNTSCSPQFM	CAA35434	UL1
STSTIAYRPDSSFMK	CAA35389	UL74(gO)
ITPDVIYEVCMLVPQ	CAA35368	UL94
FMDAHGGIHVLLYGT	CAA35427	UL28

NDNRAEAFCTSYGFF	CAA35417	UL18
HTPDQNHIEQPFYLM	CAA35363	UL89
MFPPMYPVLLLTASP	CAA35420	UL21
PGFFSWSNPACDDGL	CAA35342	UL106
MYLDMCTSSGHRPRP	CAA35311	IRS1
HHHPGPPHPPLSHPA	CAA35352	UL79
EHVHRWSYLCPPEQV	CAA35316	UL114
HGYWGKFRFCGVQEP	CAA35404	UL45
EIEGAEDKTFFHRVR	CAA35428	UL29
DVPEEFMDYVILTPL	CAA35419	UL20
MPSVVDISHFLKKQH	CAA35381	UL66
HIPCNVHVSPGWIEA	CAA35415	UL15
KKTAVCLISDEGYVF	CAA35423	UL24
FFENGLFAVYSFFER	CAA35427	UL28
LYVPAVSETLFYYVY	CAA35372	UL57
LRLCRHMDPEQDYRL	CAA35430	UL31
HHYDLCFTCDRNLSL	AAA85896.1	UL154Towne
LSQILHDRANLLRVC	CAA35427	UL28
CLSFDIECMSGEGGF	CAA35413	UL54
LFEGQYSTISTVEEY	CAA35386	UL70
IPNGNDGRGCTSEGV	CAA35413	UL54
MYATDPHDRDEVART	CAA35367	UL93
SVQHFLWMVRLYGTV	CAA35279	US12
ITEHRDLFADVFRRP	CAA35394	UL35
GQSRNSVWHLLRMDT	CAA35334	UL98
VINLFVDDCMRVFAA	CAA35422	UL23
YLVERCQQSCHGHFV	CAA35351	UL78
AMIFYRYSETCMEVT	CAA35443	UL10
VFCAVASETWHRSLF	CAA35386	UL70
CTDYVYCALRLLACP	CAA35424	UL25
SYGNSVDNLRRLHYE	CAA35275	US8
VMKLESWAHVFRDYS	CAA35332	UL130(viralentry)
MGGGRLPPLWLPLLI	CAA35447	UL14
PRYHIRYFSYGNSVD	CAA35275	US8
HLAASDLLNWYIVPV	CAA35406	UL47
VDTFGVVYGYDPAMD	CAA35395	UL36
AMYSVELAVCYFSTS	CAA35351	UL78
PCLSPDMASCHFGEC	CAA35404	UL45
LMRLGFTYFASWDLI	CAA35404	UL45
YLASNAVLALRIIRL	CAA35426	UL27
SELYLGASGAMYLWT	CAA35422	UL23
RHRDHFHTMRDYQRP	CAA35395	UL36
EDTAARCAYVEAHRE	CAA35428	UL29
SYVVTNQYLIKGISY	CAA35390	UL75(gH)

SVFSIYWQKHSDLVY	CAA35362	UL88
GIYVLTSSIAHWQTL	CAA35405	UL46
ALLFHYEHGLGRLLS	CAA35392	UL77
AGGRWRFEDGGAAQR	CAA35447	UL14
DEFSFCDSDIEDFER	CAA35424	UL25
RSFCTRDLGTIIPTH	CAA35386	UL70
VIGTIGLANLFSWDR	CAA35425	UL26
PETARVQRLLWHSLR	CAA35426	UL27
TVHRRFHTDMFHHDQ	CAA35333	UL97
FLVGVELMITHFQRT	CAA35424	UL25
ALSSKDYTFSWYKDS	CAA35442	UL9
AYYLRWHACVPQKCE	CAA35275	US8
AFCQSALLEYDDTQG	CAA35390	UL75(gH)
FFFPVGLYLPEDRGY	CAA35360	UL86(MCP=majorcapsidprotein)
LLTVTQSRWTIHHMY	CAA35440	UL7
AAEGDEFSFCDSDIE	CAA35424	UL25
CYRVSVADNLGFEPS	CAA35424	UL25
EWKSPDRQIPKNITC	CAA35437	UL4(gp48)
SLYACTRCFRTHLCD	CAA35366	UL92
FGWCSVNRYDWRQQG	CAA35413	UL54
GWPSQCIYVVGGEHS	CAA35428	UL29
ILHSRILCEWHTNYL	AAA85896.1	UL154Towne
AGPCAAWLSTRRELV	CAA35420	UL21
PRHCRLEMLILDEQV	CAA35416	UL17
VGRVCTFYVTCLMLF	CAA35351	UL78
VPYKWMPSSFIVKQC	CAA35387	UL72
EANIFCTFDHKLSIA	CAA35359	UL85
TLNTNAYDYFGKTLY	CAA35363	UL89
WVMFIHNKRCTDLDF	CAA35340	UL105
RVTFSNIATHYHYNA	CAA35269	TRS1part
VYLIIVYDYDGPETR	CAA35405	UL46
HQLQSISELCYLIYV	CAA35424	UL25
ATLKSRPGFPCHVVW	CAA35283	US16
PACDDGLFLYRTTVS	CAA35342	UL106
ESYIPGALCLYMDLM	CAA35288	US21
PEHGGEVREFKHLVY	CAA35386	UL70
PQKLWLLWQHDKHGI	CAA35444	UL11
LAGPIQNYSITYLWF	CAA35389	UL74(gO)
RTHRAAVFFHATFMA	CAA35426	UL27
YHLRRDYWLTDPKIG	CAA35447	UL14
PPMAAGSWRLCRCEA	CAA35409	UL50
IGLANLFSWDRSVAG	CAA35425	UL26
RLWCFCQDWKCHALY	CAA35264	US32
YLSPERMFFHPGLIS	CAA35406	UL47

AQLSDVIYWAVSQNY	CAA35424	UL25
RSHPGFFSCRYHPST	CAA35342	UL106
LDVMQKYFSLDNFLH	CAA35386	UL70
TATRVRTWFVERTTF	CAA35445	UL13
LAVATGQYVVCTLLD	CAA35426	UL27
VAAVGAYRHQFLIYG	CAA35363	UL89
KGTVFLCCTGFMPPL	CAA35284	US17
KDSLKALNMLCYYTE	CAA35442	UL9
ALDPHAFHLLLNTYG	CAA35390	UL75(gH)
VLANRVLQYLIHAFQ	CAA35363	UL89
FYTAHYTSRGALYLY	CAA35424	UL25
HIIMGTAGFRGGHRA	CAA35384	UL69
SHATCRYQVFVDAYG	CAA35428	UL29
SPAFYYEALFLYMLD	CAA35407	UL48(pp212)
METHLYSDLAFEARF	CAA35367	UL93
HQHDGGGDEDRLPFY	CAA35406	UL47
YDLDETFWVLGSNRK	CAA35269	TRS1part
TGQYVVCTLLDYKTF	CAA35426	UL27
SVPAAVYTTVVMHHD	CAA35432*	UL33
ELLIEDFDIYVDSFP	CAA35431	UL32(pp150)
TKKPTTTTRTTTTTT	CAA35444	UL11
SGRFNRRSSVFSIYW	CAA35362	UL88
ASTSPETQFYYTRHE	CAA35386	UL70
VSSHTSPAYDVSEYV	CAA35427	UL28
SVGELVPEPRTPYAV	CAA35413	UL54
RRHVDGISCQDHFRA	CAA35399	UL40
QLETLSRPDEPCCTP	AAA85880.1	UL141Toledo
ACYTVFGLGSIHPRF	CAA35328	UL126
EWKGAGVSRLREVWD	CAA35367	UL93
LASYLCCPEPLRFVG	CAA35422	UL23
MGTVCSQGAYVCCQE	CAA35395	UL36
ATLLCRVDHLTWISK	CAA35430	UL31
TLERYRVCEPHEETT	CAA35340	UL105
HDSHGLWCDCGDWRE	CAA35264	US32
YVREDTAVYYLARNL	CAA35423	UL24
RPLLRAWSLGLDTMA	CAA35423	UL24
DVKAWSHYLCCQTRL	CAA35423	UL24
IDTCKNTVSGYLFFG	CAA35419	UL20
LSVTTVFYTWCSDLP	CAA35280	US13
DDGDERLFRDPLTTY	CAA35426	UL27
YFMHRRLLPLFIVPD	CAA35386	UL70
TPCSSSGRRDHSLER	CAA35390	UL75(gH)
GFSKIIYIQNSAECW	CAA35420	UL21
LLTSFGCLTDCWPFE	CAA35290	US23

LREYLADLLYLNKAE	CAA35334	UL98
SKNGLLWCEYVYRHP	CAA35289	US22
PHDPYLETVGGMRQL	CAA35384	UL69
YGTRSMRKLNKPTCP	CAA35422	UL23
FYKAHCTSHMYELSL	CAA35388	UL73(gN)
DLTIKMWFLLGAPMI	CAA35351	UL78
FFWDANDIYRIFAEL	CAA35357	UL83(pp65)
LTSIATNSHYTMFVL	CAA35418	UL19
HAALFPYRALDEEDL	CAA35341	UL104
KIGEYLLEQGFPVYE	CAA35413	UL54
ATFTVHVRDATLHRV	CAA35338	UL102
NVMHLHTPMLFLDSV	CAA35336	UL100(gM)
EPNCEQPEPAHWLEY	CAA35263	US31
YVPKEDDFCHKICYA	CAA35333	UL97
GAAPVIMMTWFYAFF	CAA35432*	UL33
GCDSPRHLYISLYLL	CAA35405	UL46
GWSWIASPWKGHKPF	CAA35431	UL32(pp150)
NNTTSPWIYAIPMGA	AAA85881.1	UL142Toledo
EKLIQIIYNFYTFFM	CAA35406	UL47
LPPDSGSRGIVYCYV	CAA35391	UL76
PRPTYVLVTVNSLAR	CAA35407	UL48(pp212)
FERECYRVSVADNLG	CAA35424	UL25
TRISTVNLYLSPERM	CAA35406	UL47
CDALYFCYTQAPETR	CAA35394	UL35
ELRLERNRHLGAFHL	CAA35426	UL27
TEFPHYHRHDGGFPL	CAA35360	UL86(MCP=majorcapsidprotein)
LVVLLDELGAVFGYC	CAA35311	IRS1
TETALDYALGSWLFG	CAA35407	UL48(pp212)
GLRRCNFITVPEELP	CAA35422	UL23
HLQRIYSMMIEGASR	CAA35394	UL35
GPSSLAPGRCFSCVP	CAA35310	J1I
VMEAGGQMIHKKTKK	CAA35269	TRS1part
HNDASCYIAGGRWRF	CAA35447	UL14
SGTGVAAVGAYRHQF	CAA35363	UL89
YVFCYVREDTAVYYL	CAA35423	UL24
QYVLVDTFGVVYGYD	CAA35395	UL36
LIPIVVAYTGAVYAC	CAA35423	UL24
QFWHGAIVLEYWNAL	CAA35397	UL38
HRDNGQEENYYVTVL	CAA35433	TRL14
QLVFLAVTIYYLVCW	CAA35336	UL100(gM)
AVFTTHRFTYLPVGS	CAA35387	UL72
DTAVYYLARNLMEFA	CAA35423	UL24
LLDRWRPPKTSRPWK	CAA35422	UL23
LALTAEFGLGCLEAY	CAA35423	UL24

ELCDESVRRFVLRHM	CAA35404	UL45
NFEFLVRYYIGPWYA	CAA35392	UL77
CAAGGDWLSAVGHVL	CAA35338	UL102
VTSPNYPELCYLVDV	CAA35360	UL86(MCP=majorcapsidprotein)
GHTQTVYFDAAYVHA	CAA35387	UL72
YNVLFYTAHYTSRGA	CAA35424	UL25
LDLPYPRGYTLFVCD	CAA35425	UL26
TVFEEETVWSLCPPN	CAA35404	UL45
VDFWLLRFQPGENEV	CAA35338	UL102
ALFDFLRVVRQQDAF	CAA35424	UL25
FGTKDDVRGFTVFSH	CAA35420	UL21
YSRVIGGTVFVAYHR	CAA35414	UL55(gB)
RWCPTPGRGRRGGEG	AAA85880.1	UL141Toledo
SNEEAETLRYVYYRN	CAA35367	UL93
SICFGVPGETGGGCF	CAA35372	UL57
STPPLGKLWLYAMAT	CAA35407	UL48(pp212)
HGETDFYMNWTLRRS	AAA85887.1	UL148Toledo
FLAYVYSMDCLHTVA	CAA35408	UL49
GGLNLDDFMRRQRGR	CAA35425	UL26
CCLLLQSAWTHLYDV	CAA35338	UL102
PEFLYSLGVYRLHVN	CAA35361	UL87
RCDPYQVYFYGLQCP	CAA35261	US29
ELEYQAMGAVWRAAF	CAA35311	IRS1
TSIICEEDLDGDCRQ	CAA35333	UL97
EQKKAFYWRLFLQSQ	CAA35434	UL1
FRRSYAYIYTTYLLG	CAA35414	UL55(gB)
TFGLAGPCAAWLSTR	CAA35420	UL21
LEEAAPFGRVSVTRH	CAA35430	UL31
CVLYVTPDLDFYWVL	CAA35367	UL93
ALLTLLSSDTAPRWM	P16832	UL115(gL)
TDPSRCDPYQVYFYG	CAA35261	US29
VFVDAYGAVFAYDAQ	CAA35428	UL29
AMVYIRRSCLVHSAC	CAA35426	UL27
DCGDWREHLYCVYDS	CAA35264	US32
YRRRGLTEVLAYHLY	CAA35386	UL70
HTFAGMYELSQILHD	CAA35427	UL28
RVPCVDRWPFFPFRA	CAA35340	UL105
MERRRGTVPLGWVFF	CAA35448	UL16
RCTDLDFGDLLKYME	CAA35340	UL105
RDSHDRWCLCNAWRD	CAA35263	US31
PLFIVPDAYREHPLG	CAA35386	UL70
YMCPGIFDFLRYAHA	CAA35407	UL48(pp212)
DANFTFYYSCYNLTV	CAA35437	UL4(gp48)
PTCPYGVDHQLFDDA	CAA35422	UL23

VFPQDMLQCLWLELK	CAA35407	UL48(pp212)
SVYLSPYLSSVWVPM	AAA85873.1	UL134Toledo
TPGRGRRGGEGYRRL	AAA85880.1	UL141Toledo
LQMNLSKISWLERHC	CAA35397	UL38
RLLICPRGFELLPEF	CAA35419	UL20
ERTIRSEAEDSYHFS	CAA35414	UL55(gB)
RHLDELARYGMMYTE	CAA35290	US23
EECMWKLVGKSRKHR	CAA35368	UL94
MLFLDSVQLVCYAVF	CAA35336	UL100(gM)
MQDWVVERCNRLCDA	CAA35394	UL35
EFFKDSVIDLLTCRW	AAA85872.1	UL133Toledo
FDTNNAMGTYRCGAV	CAA35384	UL69
PMTTGSRVVKYYDGS	CAA35337	UL101
QLHEANVYLCPGYLH	CAA35394	UL35
GLLFSAMYYFYVSPY	CAA35340	UL105
KANWARESRTPLCYA	CAA35404	UL45
KKCNQTEKWHNVDWI	CAA35433	TRL14
TETCDLDGYMCPGIF	CAA35407	UL48(pp212)
LEEFEVRCVAVARRG	CAA35431	UL32(pp150)
YRIADNFHMFLKCGL	CAA35289	US22
FLPSFSQVTSSMTCD	CAA35368	UL94
FLNRELGDRLYQRFL	CAA35408	UL49
LLGTVATSFCHRVSD	CAA35430	UL31
LEYWNALFPVEVRSH	CAA35397	UL38
VVDYSHNLWCTCGNW	CAA35263	US31
STNFFFSQCEHYPSF	CAA35261	US29
AIKIWEFPCLRLHDG	CAA35400	UL41
DEGRDAQRLASYLCC	CAA35422	UL23
VLGSNRKNDVYQRRW	CAA35269	TRS1part
AYDDSKFCRYVELIC	CAA35341	UL104
HASYHANYGAYAVLM	AAA85884.1	UL145Toledo
HEVTSEFFGRVLAQL	CAA35361	UL87
DHEVELYRALDAYRA	CAA35367	UL93
YDVSEYVFSGRSVLD	CAA35427	UL28
HESQMINKRVKRKKL	CAA35453	TRL4
IWVSGHGHAFAYLPG	CAA35427	UL28
YLRLDGTLCLFLEPE	CAA35426	UL27
WAVSQNYDYALYAST	CAA35424	UL25
YRAVACRSTIFSPED	CAA35368	UL94
TWTHECNTTENGSFV	CAA35417	UL18
AGGTRVPCVDRWPFF	CAA35340	UL105
HVHDLKRIRFTEDTF	CAA35426	UL27
GGGVVSWRPESPSPD	CAA35404	UL45
RQGSFWFRCPRRFCF	CAA35427	UL28

KSGDSGFFDLSRWFG	CAA35454	TRL6
VTPDLDFYWVLPGGF	CAA35367	UL93
CQLWKDWVTNASHDT	CAA35419	UL20
SQLPEKYIGFYQIRK	CAA35311	IRS1
YQMCVMKLESWAHVF	CAA35332	UL130(viralentry)
GWLVSPYDVINLFVD	CAA35422	UL23
CAEKSDDIVIQISCV	CAA35413	UL54
YTGRLIMNVRRSWEE	CAA35431	UL32(pp150)
WASLFGYTHPDRHPV	CAA35386	UL70
INRGESYLTTIWLLN	CAA35419	UL20
LVLIVAILCYLAYHW	AAA85877.1	UL138Toledo
TTYEYLDDCRDDEEF	CAA35426	UL27
LHHGLDLQRMHDYLS	CAA35407	UL48(pp212)
VIGWMEPVNKAVFMD	CAA35427	UL28
SQRMEHGQEETHDIP	CAA35340	UL105
EIALGYRSQSVLTWT	CAA35417	UL18
DRFVQLLFYMWAGTG	CAA35411	UL52
ACALPHGWSVMNSCS	CAA35347	UL109
LKFVLQDFDVQHLRR	CAA35424	UL25
FWSIFTVCFLCYLCY	AAA85880.1	UL141Toledo
LVANLPHEDAFYTGL	CAA35430	UL31
AELLDLTYQRLIYWA	CAA35406	UL47
LRCRETSAMWSFEYD	CAA35333	UL97
RPTPLASYACYTVFG	CAA35328	UL126
QNVILPGVIFVSVGG	CAA35421	UL22
TDEQCCLLLQSAWTH	CAA35338	UL102
DAIKFLNHQCRVCHF	CAA35333	UL97
EWIKYGPRAHQLCSI	AAA85895.1	UL153Towne
VLSNFPHLGYPCVYY	CAA35336	UL100(gM)
VALVFYYDSGMTPDV	CAA35404	UL45
IPAGFCSSSPYQRLE	CAA35295	UL132
IFKNTGCAVSLCCFV	CAA35409	UL50
DREQWERPRWDALHL	CAA35311	IRS1
EQIQSQVDEIQDLRT	CAA35341	UL104
MARGTYGTYICSPNP	CAA35323	UL121
VCASESSTSWAVTSN	CAA35439	UL6
MRRHANDDFYKAHCT	CAA35388	UL73(gN)
ILTRLEYLYKVDSQR	CAA35413	UL54
CYVIEFKTTYSDADD	CAA35391	UL76
ILRQTEKHELLVLVK	CAA35390	UL75(gH)
SPSRDRFVQLLFYMW	CAA35411	UL52
AQDVVTSWIEALRDA	CAA35430	UL31
YLKGNCTQWSVIYSG	CAA35417	UL18
SVHLVLSNFPHLGYP	CAA35336	UL100(gM)

ITRYLAQFRGTMDDD	CAA35430	UL31
RPPPPPMAAGSWRLC	CAA35409	UL50
NVTCQLTFWEASERT	CAA35414	UL55(gB)
GAFRRLVHEAQYLPE	CAA35426	UL27
IAHWQTLVDVARGKF	CAA35405	UL46
AHQLCSIGHYYSTSP	AAA85895.1	UL153Towne
DGQVIRESACYVSRV	CAA35425	UL26
HNPAVFTWPPWQAGI	CAA35357	UL83(pp65)
PACTFTFGSWNVAEA	CAA35427	UL28
DFISRQHVLYNGCCV	CAA35360	UL86(MCP=majorcapsidprotein)
VASETWHRSLFPRDL	CAA35386	UL70
SRLKLVLSFVWLVGL	CAA35438	UL5
VIMMTWFYAFFYSTV	CAA35432*	UL33
VVELAYSDRRDHVWS	CAA35351	UL78
LTLWIACTGAVMVGD	CAA35280	US13
ASAAAGASSTWLAQC	CAA35392	UL77
REFYVYDGYSGHGPV	CAA35338	UL102
SSSSNNHHHHHHHHN	CAA35315	UL113
TEFPQYYILAGPIQN	CAA35389	UL74(gO)
FYREIKHLLSHDMVW	CAA35413	UL54
LWTDHIYSDSLTFVA	CAA35422	UL23
GEFRALHLIGTVSHA	CAA35428	UL29
LKFGFQYHLEGWFPL	CAA35396	UL37
SYHSILADFNSYKAH	CAA35360	UL86(MCP=majorcapsidprotein)
AWIRHYNRSTHGHHL	CAA35460	TRL12
PPVPVYAVHGLHTLM	CAA35338	UL102
PGPSVRYRAHIQKFE	CAA35426	UL27
PEEKGGGEGGGLRWF	CAA35375	UL60
VYLHSVESYSLQFHD	CAA35382	UL67
LRYAQRNCTHSFYLV	CAA35389	UL74(gO)
ISSFTSTNLTHTAVH	CAA35442	UL9
RHSMRCRRRDMASSA	AAA85891.1	UL150Toledo
AVNLSTLNSLWWWLQ	CAA35389	UL74(gO)
VSEAWRFEEAVNMAL	CAA35369	UL95
IAAPHLPLYNEFTSF	CAA35319	UL117
YNEQHYRYEVANLTY	CAA35437	UL4(gp48)
AADLLFVCTLPLWMQ	P09704	US28
WCLADDIHTSFLVHK	CAA35405	UL46
VIDRRITTFGWCSVN	CAA35413	UL54
KDDLSYKDIPRCFVA	CAA35413	UL54
VAVCQTLRTFWPQIS	CAA35407	UL48(pp212)
PEMTWDLDVRNKWRR	AAA85875.1	UL136Toledo
CASYNDTFYPTNFTP	CAA35382	UL67
TRSLTSIQNLLCAIP	CAA35415	UL15

GLNLKTLLVDLFYRP	CAA35360	UL86(MCP=majorcapsidprotein)
PTLTFSTIHSTTPWL	AAA85895.1	UL153Towne
CLIDTCLELCPPTFS	CAA35378	UL63
MSASEWWVESALEKL	CAA35404	UL45
GDWLSAVGHVLGRLP	CAA35338	UL102
DYSNTHSTRYVTVKD	CAA35414	UL55(gB)
LWLGVEYHHEVTSEF	CAA35361	UL87
VTCLMLFVPYYCFRV	CAA35351	UL78
KFERLHVRRFRPHEV	CAA35426	UL27
QNLQRLNENHRGMLR	CAA35424	UL25
RTFAGTLSRHPHRPH	CAA35352	UL79
HIRQQFDWLEEPLLR	CAA35384	UL69
CILLIGLRRDLLEDF	CAA35396	UL37
LGNYWLHRDPRGPGC	AAA85885.1	UL146Toledo
TLEDALNDMYLLLTL	CAA35394	UL35
TWFYAFFYSTVQRTS	CAA35432*	UL33
RVHLCYWPEIVRSLV	CAA35447	UL14
TGVIQKLGWCLADDI	CAA35405	UL46
SHWVPYPLRIPHYPP	CAA35436	UL3
WLDLGPHLLHRRLET	CAA35405	UL46
RFDLCTDSVLDYLGR	CAA35391	UL76
NDLAFLVGVELMITH	CAA35424	UL25
HLPTLYQLSFGGPLG	CAA35368	UL94
QLVMVPFVPHACPHY	CAA35430	UL31
HRLRECYHPAFRPMP	CAA35333	UL97
VPEVFCTRDLADLCV	CAA35428	UL29
STSELTGVCYAFNVP	CAA35404	UL45
PQETTEYTCSFFSWG	CAA35320	UL118
KDYTFSWYKDSLKAL	CAA35442	UL9
HATHSLQYAEGLRQL	CAA35391	UL76
SLYADPFFLKYVKPP	CAA35340	UL105
NRRSSVFSIYWQKHS	CAA35362	UL88
AGATMTLVLFATEYD	CAA35386	UL70
GRVRNFEFLVRYYIG	CAA35392	UL77
ANFTFYYSCYNLTVS	CAA35437	UL4(gp48)
GWEGERHRVVHYIPG	CAA35447	UL14
YALYASTPALFDFLR	CAA35424	UL25
NFLTEEPFQRGDPFD	CAA35403	UL44(pp50)
PQPYWPHLYRELRQA	CAA35352	UL79
PLWDYLDSLLFLDEI	CAA35389	UL74(gO)
HFTPVKFVYEVWRGQ	AAA85881.1	UL142Toledo
LTPPSNGCTVDVGRN	CAA35262	US30
PYPLRIPHYPPSWSR	CAA35436	UL3
LKDSDFLDAALDFNY	CAA35390	UL75(gH)

IYDRVPDCPKGRQHR	CAA35423	UL24
VYVTVDCNLSMMWMR	CAA35313	US2
CAPDFNMEFSSACVH	CAA35403	UL44(pp50)
TEELYLRLDGTLCLF	CAA35426	UL27
ISALSESCNQTCSCQ	CAA35461	TRL13
EGSFCGCEGRSFFRT	AAA85892.1	UL151Toledo
LPPHPGFFSWSNPAC	CAA35342	UL106
RREIFIVETGLCSLA	CAA35390	UL75(gH)
AHLHEEILRYDGLCR	CAA35341	UL104
WWTMLNALILMGAFC	CAA35388	UL73(gN)
AGRDDDTFVFMGARH	CAA35364	UL90
LALLIDDFRYESIGP	CAA35428	UL29
VALRHVVCAHELVCS	CAA35357	UL83(pp65)
FGYTHPDRHPVYFFK	CAA35386	UL70
ALDFWKRHFLARNVF	AAA85873.1	UL134Toledo
RAMLVMLDYYWIQLI	CAA35419	UL20
LLVVFFVIYAREEEK	CAA35458	TRL10
YVDPHYPGWGRRYEP	CAA35354	UL80A
VVGIFDGQHFFTYHV	CAA35417	UL18
FGNNFFVRTGHMVLA	CAA35336	UL100(gM)
RHLELGVVIAICMAM	CAA35275	US8
NGTYVSGLYNCTDCT	AAA85883.1	UL144Toledo
YLALRDDGRPLAWRR	CAA35407	UL48(pp212)
EAKRILVKGHGAMDL	CAA35368	UL94
ISDTDLQRLVVTRVW	CAA35386	UL70
LSAQASVSYALRYDD	CAA35311	IRS1
LVEPCARVYEIKCRY	CAA35334	UL98
EVAIAECAAHMIISV	CAA35363	UL89
GFTSGGGVVSWRPES	CAA35404	UL45
YYFDSLYTYGWVLRT	CAA35442	UL9
NTTVVAMALCYGFGN	CAA35336	UL100(gM)
RKKLEQHAAKKQNIY	CAA35458	TRL10
SWDIQDEKNVTCQLT	CAA35414	UL55(gB)
EKYWRMRTTHTVEFY	CAA35375	UL60
FCNTTACNSPFLASG	CAA35322	UL120
TPVRRPMGEVAYYGG	CAA35261	US29
KGRCTDIYALDFWKR	AAA85873.1	UL134Toledo
GNNMTTLPVWTPECK	CAA35396	UL37
WSVIYSGFQPPVTHP	CAA35417	UL18
TVNLYLSPERMFFHP	CAA35406	UL47
ELARYGMMYTEAVYR	CAA35290	US23
GMYELSQILHDRANL	CAA35427	UL28
PPRPRRRPTWMTAVF	AAA85875.1	UL136Toledo
EVYQTLRDYNVLFYT	CAA35424	UL25

DVAFTSHEHFGLLCP	CAA35357	UL83(pp65)
HLALVCGTCPQLVSG	CAA35372	UL57
RSPMPSHIHTMIFSP	AAA85873.1	UL134Toledo
PHRALFRLCLGLWVS	CAA35445	UL13
THSFYLVNAMSRNLF	CAA35389	UL74(gO)
AETLRYVYYRNVDSA	CAA35367	UL93
VVERCNRLCDALYFC	CAA35394	UL35
RSLLAFRELLACEDT	CAA35428	UL29
LDHLTLHLVPCTKCK	CAA35275	US8
KLCDGSYEEGFVVIR	CAA35407	UL48(pp212)
FRRFYHECSQTMLHE	CAA35333	UL97
TTPTSPSMGFQRTFI	CAA35389	UL74(gO)
CSCKCISGPCSRCCS	AAA85872.1	UL133Toledo
LSKKLDAFLLNWLHH	CAA35407	UL48(pp212)
MLGASVDRTYRLNRI	CAA35372	UL57
MRVISRARSACTWTS	P09724	US20
CPDEEPDRCWTVIQR	AAA85880.1	UL141Toledo
ATNSHYTMFVLDHGS	CAA35418	UL19
LYSDLAFEARFADDE	CAA35367	UL93
WPPLHGIMLGDTQYF	CAA35427	UL28
WYGRETRKRNSHKKV	CAA35452	TRL5
VVAAPGPSVRYRAHI	CAA35426	UL27
VRAEFFWGAAGEGSV	CAA35376	UL61
AEALERVAERCDDRH	CAA35334	UL98
SITYLWFDFYSTQLR	CAA35389	UL74(gO)
DLRAANATYAQMVKK	CAA35407	UL48(pp212)
RSEAEDSYHFSSAKM	CAA35414	UL55(gB)
LVGWSWGTGRRPVGF	CAA35453	TRL4
KELRMCLSFDSNYCR	CAA35431	UL32(pp150)
WLNAGAFRRLVHEAQ	CAA35426	UL27
TGSLDYRWLGCQIPI	CAA35351	UL78
CYYVYVTQNGTLPTT	CAA35444	UL11
PDPAARLCRDMRRVT	CAA35269	TRS1part
CRYQVFVDAYGAVFA	CAA35428	UL29
CLVIRRRWRLVRDEG	CAA35422	UL23
RGKTLLLSSHWVPYP	CAA35436	UL3
VRIELGVYFFSSPTS	CAA35383	UL68
WGHKSICSFFPKLQG	CAA35437	UL4(gp48)
LCYLIYVQLPSLRED	CAA35424	UL25
DFLDAALDFNYLDLS	CAA35390	UL75(gH)
ISCNHFLRDLLTDRF	CAA35446	UL12
YPYRVCSMAQGTDLI	CAA35414	UL55(gB)
SISELCYLIYVQLPS	CAA35424	UL25
ALPSSGYHFGFVRQN	CAA35406	UL47

VVPHLHCLINPILYA	CAA35432*	UL33
AAYCYCVFGTCSIGT	CAA35437	UL4(gp48)
SKFCRYVELICSREK	CAA35341	UL104
AARHFADYVDPHYPG	CAA35353	UL80
GIFDFLRYAHAKPRP	CAA35407	UL48(pp212)
LWAREPHGQLAFLLR	CAA35269	TRS1part
RYDELRDAIHELKRD	CAA35353	UL80
YHLKLRPATFGLETW	CAA35399	UL40
NMPRAFSFYLLTSAQ	CAA35430	UL31
VCGTCPQLVSGFVWY	CAA35372	UL57
EEDPIRYYVSVYDEL	CAA35295	UL132
RLPFLLLFQRPQWAH	CAA35445	UL13
DFGVVADLLKWIGPH	CAA35403	UL44(pp50)
FAPEDFSFQWFRSIS	CAA35340	UL105
TSPAYDVSEYVFSGR	CAA35427	UL28
LTGVCYAFNVPSVLM	CAA35404	UL45
VSRDLGFADWSQTLI	CAA35426	UL27
VFSLTVHAPYDIHFG	CAA35430	UL31
DARLSYVMLTVYPCS	CAA35276	US9
SIVRQAMQHAGFQVR	CAA35445	UL13
RLQYVYLIIVYDYDG	CAA35405	UL46
VWNAFRLIERHGFFA	CAA35273	US6
QSVLTWTHECNTTEN	CAA35417	UL18
TSPSWFISVFGHTEG	CAA35386	UL70
TVMRFDQRLLEEGDE	CAA35385	UL71
RLREKWDTRGYLYKG	CAA35443	UL10
EPLSLFLMNTFLLHQ	CAA35334	UL98
DGPETPWGQLICCEE	CAA35293	US26
IFGTFCNRLEWVYFL	CAA35360	UL86(MCP=majorcapsidprotein)
LWCTLWGSRTRVSLG	CAA35439	UL6
FEDVERAAQGRLRHG	CAA35425	UL26
NRQEAVRAGLLCRTP	CAA35426	UL27
IMRHGPCLIRHSPRC	CAA35404	UL45
HTYAVCEKFIENLRF	CAA35338	UL102
MVLLAAYCYCVFGTC	CAA35437	UL4(gp48)
RDGIRWQYQELQYLV	CAA35445	UL13
PPPRFPPRCSDAGTI	CAA35415	UL15
SEATHPVLATMLSKY	Q7M6N6	UL48A
FYAVFTTLGLRCPDN	CAA35367	UL93
ITARNMPRAFSFYLL	CAA35430	UL31
LIAWVEEMLRYVESK	CAA35407	UL48(pp212)
HDCGTYRGFQRNYFY	AAA85880.1	UL141Toledo
TFFHRVRILCGDTGT	CAA35428	UL29
LQRQREEYHGVYEHL	CAA35407	UL48(pp212)

MTLVLFATEYDSAHI	CAA35386	UL70
IPSWHVFASLDDLVP	CAA35356	UL82(pp71)
LQSAWTHLYDVLFRG	CAA35338	UL102
RHRGTPESPRLTEVY	CAA35424	UL25
GHSYVLRAIAHTSPF	CAA35396	UL37
LFLQKDTFFHEQFLA	CAA35451	TRL3
LIASLDEAETQPLYR	CAA35386	UL70
AARDADEWFRHGAGE	CAA35311	IRS1
LFFFKIPQRLREKWD	CAA35443	UL10
GMDEPPSGWERYDGG	CAA35353	UL80
PVEVRSHVRQHAHTM	CAA35397	UL38
LHCEAIYGEQMRTPL	CAA35293	US26
AERRAANWRRQMRRL	CAA35264	US32
AEDCWKPACPDEEPD	AAA85880.1	UL141Toledo
CVMGKKGHRNHRFSG	CAA35395	UL36
LNISLENCAFCQSAL	CAA35390	UL75(gH)
RGQPKFSSIWRGLRD	CAA35427	UL28
HTFWRLPVAVFFEPH	CAA35392	UL77
KQFYGGLIFHTTWVT	CAA35438	UL5
FMLIPWRVTAPYLRD	CAA35279	US12
RNLMEFARVGLRAVE	CAA35423	UL24
FVWLVGLRLHDCAAF	CAA35438	UL5
IQNYSITYLWFDFYS	CAA35389	UL74(gO)
SYSCGEGALPALGRY	CAA35407	UL48(pp212)
ALRIIRLLRASIRHE	CAA35426	UL27
AVEFQNYVKNSVRHM	CAA35406	UL47
YVEAHREAQLTLIWP	CAA35428	UL29
YVFVWYRGYEFPAPT	CAA35369	UL95
RFLWQRRQRARMLQH	CAA35396	UL37
QKILRCLKTGETCVW	CAA35378	UL63
QLCLTEATSLLHRHR	CAA35406	UL47
EHGGVLPSFFFSGSA	CAA35422	UL23
YSDCVDPGLAVYRVS	CAA35438	UL5
PHDGVYLPKDAFFSL	CAA35354	UL80A
ITVAVAVCQTLRTFW	CAA35407	UL48(pp212)
FVSNFPRKLGINSHN	CAA35443	UL10
CLSADWIRFLSLPDH	CAA35316	UL114
DIPSCDRCVRRRRFK	CAA35395	UL36
FCTERDYRKFHQGIA	CAA35393	UL34
VREVCFLRTCLRLVT	CAA35338	UL102
AEMLNNWDGWDAFTI	CAA35290	US23
IAQVFDGCYHSEAYR	CAA35393	UL34
LSLHLDDYPYLMVEI	CAA35361	UL87
ISTAFCGMIWLGIPD	CAA35280	US13

CDGAVWNAFRLIERH	CAA35273	US6
GHRTVAQMSKALKKT	CAA35406	UL47
CVSLGWPSQCIYVVG	CAA35428	UL29
VVVFFYYFYNALGDT	CAA35340	UL105
YSLFGRPVSRRLSDL	CAA35404	UL45
YPPSWSRTIPNRIRY	CAA35436	UL3
DLDGYMCPGIFDFLR	CAA35407	UL48(pp212)
QPEVCVLYVTPDLDF	CAA35367	UL93
LLLCAVWATPCLASP	CAA35332	UL130(viralentry)
LWSGSLPHLPVYDVR	CAA35445	UL13
HGGIHVLLYGTMLVK	CAA35427	UL28
ESWAHVFRDYSVSFQ	CAA35332	UL130(viralentry)
EYFFQHMVGRLGVGP	CAA35407	UL48(pp212)
CIICVGSPTQTEALE	CAA35340	UL105
EWTLQKVFYLCLMPA	CAA35360	UL86(MCP=majorcapsidprotein)
CTRCFRTHLCDLGSG	CAA35366	UL92
DRLVVGYFDSLSSLY	CAA35427	UL28
TYILPADCRYAPLFA	CAA35319	UL117
SSNVFDLEEIMREFN	CAA35414	UL55(gB)
SGILFFLAPSMAQFW	CAA35397	UL38
ASAAAAAAACEDLSE	CAA35340	UL105
SRKGFLFTKQIKSSK	CAA35372	UL57
RLITETAGTYTCVLG	CAA35447	UL14
DIGYGVYVDKAFAML	CAA35338	UL102
ARSKYPYHFFATSTG	CAA35414	UL55(gB)
QLQQVVHHVRVARKG	CAA35386	UL70
VRDDRYIRVGDNLNT	CAA35423	UL24
GRVLAQLHRDRARVM	CAA35361	UL87
VTTEHAGKYVLQRYS	AAA85895.1	UL153Towne
RLVFEAAQRGLFDYS	CAA35361	UL87
LGQQPTTVPPPIDLS	CAA35390	UL75(gH)
KLQPGVGLWIDFCVY	AAA85887.1	UL148Toledo
LAPGRCFSCVPRDPC	CAA35310	J1I
FGALIIFLAYVYHYE	AAA85885.1	UL146Toledo
PTHASMGEFARLLLG	CAA35386	UL70
QCIYVVGGEHSPHSL	CAA35428	UL29
ALKRAMYSVELAVCY	CAA35351	UL78
LLCFSPACFSHSLYL	CAA35355	UL81
LRVGYFGHLNIKGLE	CAA74075	UL43rev
QYVKVQFGYHLGAFF	CAA35336	UL100(gM)
RDYLRFPTRLEFIPL	CAA35407	UL48(pp212)
YAELFFLLCSTSESS	CAA35430	UL31
LGSWLFGIPVCLGVH	CAA35407	UL48(pp212)
KTFFIYNVSESSGGT	CAA35439	UL6

RVAFGCSWKTFFIYN	CAA35439	UL6
VELAGCWGLYAAILC	CAA35404	UL45
LHVRRFRPHEVGGHA	CAA35426	UL27
YDHRLAFDVILPCAA	CAA35408	UL49
DWFGAVYAIQMDDPN	CAA35291	US24
LENVTVYPTYDCVLS	CAA35392	UL77
FYVRQKTYHLLGTES	CAA74073	UL41alt
ASHLTAYESYLVSIT	CAA35430	UL31
WLLPRPEGYTLFFYI	CAA35368	UL94
QLLTRVHNHILNGFL	CAA35407	UL48(pp212)
TACIRMLSGDVQRLI	CAA35426	UL27
YDFLISADPFSRDAS	CAA35404	UL45
INPILYALLGHDFLQ	CAA35432*	UL33
CRVDADLGLLYAVCL	CAA35323	UL121
VQTLRKEMCAKSENS	AAA85873.1	UL134Toledo
YEHASEGWRFCSRRE	CAA35264	US32
LRLTSHGHGLLCARC	CAA35408	UL49
YYLARNLMEFARVGL	CAA35423	UL24
LLKEAIKIWEFPCLR	CAA35400	UL41
HEREILDLMRHSPDV	CAA35385	UL71
VVVSVALIALYMGSH	CAA35433	TRL14
VQVPIRTRRLLVPWI	CAA35419	UL20
QQRAFCRASRVLTDP	CAA35429	UL30
ERTIYQEGKFIFELY	CAA35404	UL45
VSRSGFRGFVQEGLR	CAA74075	UL43rev
RMRRATLPRALARAC	CAA35375	UL60
LYFWNNDVFRKLLRA	AAA85879.1	UL140Toledo
WSRTIPNRIRYIPAT	CAA35436	UL3
QRGERGAWMPAETFT	CAA35449	TRL1
NSGRYSRRCFKENYF	CAA35460	TRL12
AACSYAHTLSLHSDM	AAA85892.1	UL151Toledo
MRIEWVWWLFGYFVS	AAA85881.1	UL142Toledo
RYPFLVGWSWGTGRR	CAA35453	TRL4
LVSPDRLVVGYFDSL	CAA35427	UL28
LNKPTCPYGVDHQLF	CAA35422	UL23
RLYQRFLREWLVCRQ	CAA35408	UL49
PVKYRTHRAAVFFHA	CAA35426	UL27
PWRVTAPYLRDTLPF	CAA35279	US12
SRGHREFYVYDGYSG	CAA35338	UL102
DSRFSDEALTETVWL	CAA35386	UL70
ERYDTDYLLRRLRLY	CAA35408	UL49
IYYGDDPERYNIHFE	CAA35360	UL86(MCP=majorcapsidprotein)
VERVTLPYLCHILAL	CAA35341	UL104
FLKRTVKLTRNKTKH	CAA35396	UL37

LLQYCIVRFIGTRLF	CAA35274	US7
EEFCHQFLRAYLTPI	CAA35426	UL27
RGLTLESDLAVWAAL	CAA35426	UL27
LFDTITVRRVAEEWK	CAA35341	UL104
VVFVHHVVKYSIMAD	CAA35404	UL45
VRLSRLSLDEVKKYG	CAA35413	UL54
ALGAGFRVFVYDLAN	CAA35311	IRS1
TRSLLLICGYYPPRE	CAA35277	US10
PPEPLREYLADLLYL	CAA35334	UL98
TYLCDDIHAIRFRVS	CAA35363	UL89
EWDGKESDDESSASS	CAA35264	US32
VVCTLLDYKTFGTRT	CAA35426	UL27
QRLIYWACTLMPYVL	CAA35406	UL47
TGFMPPLSLMVPTIC	CAA35284	US17
EKEQRVRMFYAVFTT	CAA35367	UL93
TMCNLALSTPFLMEH	P19893	UL122(IE2)
NYPELCYLVDVLVHG	CAA35360	UL86(MCP=majorcapsidprotein)
LILDEQVSKRSWDTT	CAA35416	UL17
VITRSLLLICGYYPP	CAA35277	US10
RLFALIQLCRVLLPE	CAA35406	UL47
GGQMIHKKTKKPKGK	CAA35269	TRS1part
MILWSPSTCSFFWHW	CAA35276	US9
RNPAKGCLGFLLYRH	CAA35372	UL57
EPLSAYVNALHDHRL	CAA35360	UL86(MCP=majorcapsidprotein)
VGYFDSLSSLYLRGQ	CAA35427	UL28
NRKNDVYQRRWKKTV	CAA35269	TRS1part
EVKLAICHDNYYISR	CAA35411	UL52
PPRRRPARTDLYYYR	CAA35393	UL34
FRQRLFSRDVSWYHS	P09704	US28
GAIVLEYWNALFPVE	CAA35397	UL38
VQIGFLHTQLVMVPF	CAA35430	UL31
IIFLAYVYHYEVNGT	AAA85885.1	UL146Toledo
STPSRIRKAKLSSPM	CAA35431	UL32(pp150)
WGVTPSVWASRGTVF	CAA35404	UL45
RERCSFVNRRITRPR	CAA35405	UL46
CFLNLLDRWRPPKTS	CAA35422	UL23
TVTLEKEQRVRMFYA	CAA35367	UL93
VYHGRRLGRDPYSRR	CAA35436	UL3
DASWAAMCKWMSTLS	CAA35404	UL45
ETVWCLDRDRGVLYY	CAA35293	US26
HRRAGTMQLAQRLCE	CAA35385	UL71
CHGWTQQVSSQIRTR	CAA35445	UL13
DLSALLRNSFHRYAV	CAA35390	UL75(gH)
IPKKPHYTKLPKYDP	CAA35433	TRL14

EHTLNDLAFLVGVEL	CAA35424	UL25
TTYNLLFHPPPFTTV	CAA35394	UL35
TPYTIYGTLDMSSLY	CAA35389	UL74(gO)
QTLHLRLVWPDGSYR	CAA35367	UL93
SPRQQACVPRTKSHR	CAA35273	US6
FGCLTDCWPFEVAPA	CAA35290	US23
QLVLFMTPKWDVFAY	CAA35397	UL38
HNLCYSTLLVPGGEY	CAA35413	UL54
ANRYRHYEFQTLSLG	CAA35411	UL52
IPDYRSVSLRIKREL	CAA35426	UL27
HVPRSCVLHLFVTDK	CAA35408	UL49
DGVLDAVWRVQGTFY	CAA35275	US8
PFFLEYAKHHPKLSR	CAA35259	US27
RIICQKMWYFYLIGT	CAA35352	UL79
TRQAHFLARGLLGFF	CAA74075	UL43rev
SITEFLNIGLRRCNF	CAA35422	UL23
SVDRQFRRTTYDRWD	CAA35261	US29
RFTVISCNHFLRDLL	CAA35446	UL12
IHASGKQMWQARLTV	CAA35357	UL83(pp65)
TRLASDSLCLFHSSF	CAA35436	UL3
RGGGQVWSVVPSLVF	CAA35391	UL76
IQGATYQLSIVRQAM	CAA35445	UL13
FNMPYFQISIFPISP	CAA35329	UL127
ARWDQMFSYLAKLGT	CAA35281	US14
LPFTVLRLSYAYRIF	CAA35334	UL98
WKGHKPFRFEAHGSL	CAA35431	UL32(pp150)
VRGRFSGREVPAWPA	CAA35369	UL95
LPGEDKVYVLGLSFG	CAA35427	UL28
FGFVRQNVVFYLLSH	CAA35406	UL47
RYVTVKDQWHSRGST	CAA35414	UL55(gB)
MIFLCAYLIRYREFF	AAA85872.1	UL133Toledo
ALCFCLLCEAVETNA	CAA35318	UL116
VHPSLYIGFPCGIPG	CAA35375	UL60
RRHIIPGAANGMPPL	CAA35395	UL36
VQRRGLFFFSNGKSE	CAA35379	UL64
NEYFRTFRLRRLVTH	CAA35341	UL104
TKRPMPVSDSFVCLR	CAA35407	UL48(pp212)
THFGNYVVGEIIPLQ	CAA35360	UL86(MCP=majorcapsidprotein)
RGMTRCHENGIYYGT	CAA35422	UL23
YYGFKDYIGSLHGLT	CAA35372	UL57
TEFSEIFGIPQGLFQ	CAA35406	UL47
VTEDEIIGVAFTWQH	CAA35448	UL16
RYNIHFEAIFGTFCN	CAA35360	UL86(MCP=majorcapsidprotein)
FFMCLYVYSPTFLFD	CAA35406	UL47

LCQLLLLYRDGEWII	CAA35368	UL94
GVDRDYARQFRWLCR	CAA35290	US23
NKIILGNYWLHRDPR	AAA85885.1	UL146Toledo
VVRDHKTYRRFSCLR	CAA35427	UL28
VFPTATLKSRPGFPC	CAA35283	US16
ETVRRPFFSDAPLPY	CAA35358	UL84
RLRRETVRRPFFSDA	CAA35358	UL84
NTCVAYVNRVRTDMG	CAA35360	UL86(MCP=majorcapsidprotein)
CTNTTTTVTCDGFNY	CAA35461	TRL13
NETRRRLSTADWLWW	CAA35456	TRL8
QWEFMFREQRGDPIN	CAA35311	IRS1
LWAREPHGQWEFMFR	CAA35311	IRS1
NATVEWYNKSEGDVP	CAA35419	UL20
LGTGFHRAEGSFCGC	AAA85892.1	UL151Toledo
PVVTLLLARQRDGLA	CAA35449	TRL1
TGFRAHDSEDGVSVW	CAA35449	TRL1
GVCLSPDHLFSKWLD	AAA85885.1	UL146Toledo
AYTSKIGVLVVVCGF	CAA35451	TRL3
PATFFCESDDAKYLC	CAA35368	UL94
ERGLLHSYFEDVERA	CAA35425	UL26
SGFFDLSRWFGENMD	CAA35454	TRL6
LALLSFEETVHMYTT	CAA35340	UL105
DNLTLWTGPNYEISW	CAA35417	UL18
WERPRWDALHLHPRA	CAA35311	IRS1
QKEGHLYTVNCEASY	CAA35321	UL119
CLFLEPEERELIGRC	CAA35426	UL27
LAARHFADYVDPHYP	CAA35354	UL80A
RTAAHLSQNMRDMYL	CAA35311	IRS1
HQCQTYYVECEPRCL	CAA35278	US11
LWTGPNYEISWLKQN	CAA35417	UL18
RPAFMEHSRPVGYHP	CAA35456	TRL8
TEPITMLGAYSAWGA	CAA35458	TRL10
PENEGEYENLLRELY	CAA35409	UL50
NDQHVFCAVASETWH	CAA35386	UL70
IDGGNMTSVWRFEGQ	CAA35274	US7
GDALCVLPPLFHGPL	CAA35359	UL85
LELEDYDRRCRCNNQ	AAA85886.1	UL147Toledo
PKFFPPPMCRVPYNE	CAA35289	US22
KKMMWFVVLTVSFSY	CAA35381	UL66
AAIMSLLDEAEWRQT	CAA35397	UL38
IKSVFVFPYLVLPNC	CAA35429	UL30
FRHGAGEVVRLYRCN	CAA35311	IRS1
SCSPQFMCINETKGL	CAA35434	UL1
ADFSVSEAWRFEEAV	CAA35369	UL95

RRPACALPHGWSVMN	CAA35347	UL109
NMDEYSGDVWHLEVS	CAA35454	TRL6
MKTTPLPSPLLYECH	CAA35443	UL10
EETILTPRDVEYWKL	CAA35425	UL26
AHSCNEAFLPLMAFC	CAA35363	UL89
ADSASDFDADCWCMW	CAA35449	TRL1
RWDALHLHPRAALWA	CAA35311	IRS1
NEYKVTSDACMMTMY	CAA35325	UL123(pp72=MIprotein=IE1)
GHRVQTYCEDLEGRV	CAA35392	UL77
RQAGVTGIYKHFFCD	CAA35411	UL52
RGDSDMFDGVVASAY	CAA35362	UL88
TFNVSMDTAGMYECV	AAA85880.1	UL141Toledo
DRDHDDAPPTYEQAM	CAA74074	UL42rev
LICKNPNYSVCDAML	CAA35409	UL50
ARESRTPLCYASELC	CAA35404	UL45
YECDLSCNITTYNEY	CAA35434	UL1
LSVEEICEEHTLNDL	CAA35424	UL25
NYCFARDCFTHPESV	CAA74075	UL43rev
ESFCEDVPSGKLFMH	CAA35357	UL83(pp65)
PPPIDLSIPHVWMPP	CAA35390	UL75(gH)
MELHSRGRHDAPSLS	CAA35384	UL69
FRCRHTFARDYVVEN	CAA35363	UL89
TYSKPHDAATFYCPF	CAA35332	UL130(viralentry)
ARDPLYAAEQLHEQL	CAA35406	UL47
DGMYGRGEKELCIAH	CAA35323	UL121
SPTYHESQMINKRVK	CAA35453	TRL4
GQVLHNDASCYIAGG	CAA35447	UL14
TLRDYNVLFYTAHYT	CAA35424	UL25
ILCLDKVCRQLHGQD	CAA35404	UL45
LMKTVQQLHRIWPFC	CAA35291	US24
KDNQCMTDYDYLEVS	P09704	US28
LYGEYERRFADLSSL	AAA85877.1	UL138Toledo
ISTVEEYVRSFCTRD	CAA35386	UL70
PLYFEAECNRNYTLH	CAA35318	UL116
EIMEHMRLRPPPDYE	CAA35360	UL86(MCP=majorcapsidprotein)
SVCDAMLKTDTVYCV	CAA35409	UL50
LEVPGRCPHENFPFW	CAA35293	US26
VCRQCGHCLNLGKEK	CAA35408	UL49
VTGIYKHFFCDPQCA	CAA35411	UL52
TMLGAYSAWGAGSFV	CAA35458	TRL10
CGHCLNLGKEKLHCQ	CAA35408	UL49
AGASSTWLAQCAERP	CAA35392	UL77
GLEKTFLCCDKFLLP	CAA74075	UL43rev
YECVPDANTAPEIWV	CAA35427	UL28

FNMEFSSACVHGQDI	CAA35403	UL44(pp50)
NVTEVHGEVACFRND	CAA35396	UL37
CSIGHYYSTSPLNGM	AAA85895.1	UL153Towne
DFFVVTVSIDDDTPM	CAA35390	UL75(gH)
EPDVYYTSAFVFPTK	CAA35357	UL83(pp65)
LGAWQELAQYEPFAS	CAA35311	IRS1
VHMGEAARLHFTMFD	CAA35290	US23
PAMQRLLECRFQQEP	CAA35360	UL86(MCP=majorcapsidprotein)
RHPHRPHPQQQQHHH	CAA35352	UL79
WEFLARDLLREEMEA	CAA35367	UL93
WHDIEWIKYGPRAHQ	AAA85895.1	UL153Towne
PRLADDVSREIAAWE	CAA35392	UL77
FQIFTQQCEMVTEGY	CAA35407	UL48(pp212)
EFEGDFARYRSSQQK	CAA35407	UL48(pp212)
YLQNSFLHLLMNSGL	CAA35393	UL34
TTFNPMFFNVPRWNT	CAA35389	UL74(gO)
TQQVSSQIRTRWEES	CAA35445	UL13
IHRSASHLTAYESYL	CAA35430	UL31
NITLKNAIRLRNGTM	CAA35461	TRL13
SAYHRLRMSNIPRSS	CAA35362	UL88
VLRHCCFQNFTATTT	CAA35388	UL73(gN)
SSMAVYDEETMRQSQ	CAA35430	UL31
YAPLREELGYVRFET	CAA74075	UL43rev
SPSTCSFFWHWCLIA	CAA35276	US9
TVYLVSAIFREREES	CAA35334	UL98
ALLEKMQVVFDPYGR	CAA35407	UL48(pp212)
RVSSSVSECYVQHGV	CAA35276	US9
RSRELSYDDHEVELY	CAA35367	UL93
IDLRTEHSYALWASL	CAA35386	UL70
PMVLLGAWQELAQYE	CAA35311	IRS1
SSAVSSSSNNHHHHH	CAA35315	UL113
AKTMEMRFTIAWMWF	CAA35262	US30
AKLSSPMTTTSTSQK	CAA35431	UL32(pp150)
QSLIHNEEPATFFCE	CAA35368	UL94
MGPFEGYSMSADRAA	CAA35351	UL78
GHGAMDLTCQKAVTL	CAA35368	UL94
WVASEDELDVSRGDA	CAA35418	UL19
PLIMDLPSLSVELSA	AAA85891.1	UL150Toledo
TDIYALDFWKRHFLA	AAA85873.1	UL134Toledo
SFEETVHMYTTFRDI	CAA35340	UL105
KELRSSNVFDLEEIM	CAA35414	UL55(gB)
LLADYAETFSPLGSF	CAA35362	UL88
GTPQFFDQFDTNNAM	CAA35384	UL69
DMALMTLDVYCCRQT	CAA35399	UL40

TESDDEETTVWEKRR	CAA74073	UL41alt
RSLTRKKLEQHAAKK	CAA35458	TRL10
DAAVTMRGGGWREDV	CAA35392	UL77
EVHRPVVRVFVDMWD	CAA35404	UL45
SLHHFELSYRFHDED	CAA35395	UL36
DAQEDCLYELASDLA	CAA35428	UL29
RHGLLHCEAIYGEQM	CAA35293	US26
IKHLLSHDMVWPCPW	CAA35413	UL54
EWYNKSEGDVPEEFM	CAA35419	UL20
VFTAQLRHYCEHQDK	CAA35393	UL34
IFSTNQGGFMLPIYE	P19893	UL122(IE2)
RSSARLLEHCVGLAG	CAA35362	UL88
YGSGCRFDTVEMVDE	AAA85875.1	UL136Toledo
VWTPECKGWTYWTTL	CAA35396	UL37
RLLEDAAVTMRGGGW	CAA35392	UL77
ANRDDNFFAERTSGC	CAA35436	UL3
AALQWLDLGPHLLHR	CAA35405	UL46
RKTANMFMPGAFMDE	CAA35363	UL89
ITGSEFEGDFARYRS	CAA35407	UL48(pp212)
ALTAVRTDTFEVDML	CAA35360	UL86(MCP=majorcapsidprotein)
TCFGREKNGCPFPAL	CAA35369	UL95
FCLEPMEITRYVHRN	CAA35291	US24
FCTFDHKLSIADVGK	CAA35359	UL85
MWRTRWEDGAPTFTR	CAA35290	US23
FTSCGNRCDIPSMTR	CAA35368	UL94
FNGTTETCDLDGYMC	CAA35407	UL48(pp212)
RFVARRNDVDFWLLR	CAA35338	UL102
RLVELGFNHDTCAAY	CAA35406	UL47
YEISRENFVVRAADS	CAA35425	UL26
VDMVRHRIKEHMLKK	CAA35325	UL123(pp72=MIprotein=IE1)
HLMMNVNPLRLPYEK	CAA35340	UL105
TPDYEDMLCYSDDMD	CAA35384	UL69
DIFKQKATVFLVPRR	CAA35363	UL89
PFDKNYVGNSGKSRG	CAA35403	UL44(pp50)
AQGQQAATVRAEFFW	CAA35376	UL61
ILQKDTFIERTPCEQ	CAA35340	UL105
AYVYHYEVNGTELRC	AAA85885.1	UL146Toledo
NANVLTVCRHVEAHK	CAA35361	UL87
DVELRELQAFLDENF	CAA35385	UL71
TMVWGAAPVIMMTWF	CAA35432*	UL33
DSDLYRIADNFHMFL	CAA35289	US22
RIQSLGNEIRCMLLP	CAA35419	UL20
LWFDFYSTQLRKPAK	CAA35389	UL74(gO)
ILVKGHGAMDLTCQK	CAA35368	UL94

RDEVARTDEWKGAGV	CAA35367	UL93
HLLRMDTVSATKFYE	CAA35334	UL98
SSDEDESGRPRRIAN	CAA35289	US22
GRADAEDCWKPACPD	AAA85880.1	UL141Toledo
PRLENYFLCQVCLYE	CAA35362	UL88
TPFLMEHTMPVTHPP	P19893	UL122(IE2)
PDANTAPEIWVSGHG	CAA35427	UL28
VTNLTESCINRGESY	CAA35419	UL20
AKRPLITKPEVISVM	CAA35325	UL123(pp72=MIprotein=IE1)
FHRAEGSFCGCEGRS	AAA85892.1	UL151Toledo
EDHMLVFDPHSSAEC	CAA35407	UL48(pp212)
EELTRLLAVWDDEPL	CAA35334	UL98
PQSICRFCIDRLRDI	CAA35438	UL5
QTEKWHNVDWISKQP	CAA35296	IRL14
TIFSPEDDSSCILCQ	CAA35368	UL94
CKKRYIGKVEGASGL	CAA35413	UL54
RFSERPDEILVRWEE	AAA85877.1	UL138Toledo
RIRLRNSWVASEDEL	CAA35418	UL19
DLQRLVVTRVWPPLL	CAA35386	UL70
LLNFIRQRLCCEWYV	CAA35289	US22
STSVIATTQKEGHLY	CAA35321	UL119
DSAAKIQERYAELQK	CAA35363	UL89
YEVINVTGYVGGNIT	CAA35461	TRL13
RTPLCYASELCDESV	CAA35404	UL45
KDADPISTVTETRWF	CAA35342	UL106
EEEEEKEKLLTYKDI	CAA35419	UL20
RQELYLMGSLVHSML	CAA35390	UL75(gH)
ISADPFSRDASWAAM	CAA35404	UL45
NMRDMYLDMCTSSGH	CAA35311	IRS1
DVVEHWLHAQGQGQP	CAA35353	UL80
SETASTVSEDAVCWL	CAA35281	US14
VEPLGWLVSPYDVIN	CAA35422	UL23
FRCSVTSDARKDLQK	CAA74075	UL43rev
TERQSQLPEKYIGFY	CAA35311	IRS1
EGGHLLRNIKTAFGM	CAA35423	UL24
NQWKEPDVYYTSAFV	CAA35357	UL83(pp65)
MKREGSIFSWRDGNE	CAA74075	UL43rev
NMFMPGAFMDEIIGG	CAA35363	UL89
KMLRAHGTPVAEDFM	CAA35334	UL98
CESNIIVIDECGLML	CAA35340	UL105
ITFNSSCLYITDKSF	CAA35403	UL44(pp50)
GTELRLCNLRCFENS	CAA35423	UL24
NETFLWYNLTVKPKP	CAA35440	UL7
MLCYYTEKLEEIDSK	CAA35442	UL9

FPYRALDEEDLEQYL	CAA35341	UL104
YIVPEDKREMWMACI	CAA35325	UL123(pp72=MIprotein=IE1)
EEYVRSFCTRDLGTI	CAA35386	UL70
RPMGEVAYYGGCCMV	CAA35261	US29
ARTDEWKGAGVSRLR	CAA35367	UL93
LMQLSKSNPVADYMF	CAA35392	UL77
VRTDTFEVDMLLYSG	CAA35360	UL86(MCP=majorcapsidprotein)
QKDDKRSLFCYMREI	CAA35343	UL107
QLPYLSAERTVRWML	CAA35407	UL48(pp212)
VTPGKQEITDAMFEA	CAA35425	UL26
LITKGGLCSSMAVYD	CAA35430	UL31
VAFQPLLAYAYFRSV	CAA35372	UL57
NHRGMLRLLSVEEIC	CAA35424	UL25
EDTVGAASHHHRPCV	CAA35384	UL69
PVTLGSGHGYHPGQK	CAA35443	UL10
ESIMFAIVSFKHMGP	CAA35351	UL78
HHLAEDTVGAASHHH	CAA35384	UL69
QPFMRPHERNGFTVL	CAA35357	UL83(pp65)
FCESDDAKYLCAVGS	CAA35368	UL94
CVFEHEFEKIKRPIF	CAA35344	UL108
GVVYGYDPAMDAVYR	CAA35395	UL36
AECHNAAVYHCEGLH	CAA35407	UL48(pp212)
IRFHTDFRGEVVNTM	CAA35392	UL77
SYRDWEFLARDLLRE	CAA35367	UL93
FLEAPWESAPQPPRL	CAA35406	UL47
QWELVLPWIVPMPLA	CAA35359	UL85
RFLTQLWENEYFRTF	CAA35341	UL104
FHDRCASYNDTFYPT	CAA35382	UL67
VWCNCGDWQGHALRS	CAA35312	US1
MGDFQGIFECQYSAD	CAA35290	US23
TFYKCLDAQFVCMPE	CAA35387	UL72
QFLQEECMWKLVGKS	CAA35368	UL94
WPHLYRELRQAFPGL	CAA35352	UL79
CGNFTTFNPMFFNVP	CAA35389	UL74(gO)
LRRLDEELRHRGTPE	CAA35424	UL25
TDIPERIYSLSDFTY	CAA35371	UL56
TGMKTVAFDLSSPQK	CAA35431	UL32(pp150)
VRTWFVERTTFWRRT	CAA35445	UL13
KHVSQFVLKEVEFRC	CAA35363	UL89
FGVIEAWEEASVRPT	CAA35397	UL38
DSFDYLVERCQQSCH	CAA35351	UL78
QIWSPTPWRLRNHDC	AAA85880.1	UL141Toledo
LDDAFLDTLALLYNN	P16832	UL115(gL)
ENASTWTFSFGIWYY	CAA35392	UL77

PWIRESKMWVLPPPL	CAA35419	UL20
MDDLRDTLMAYGCIA	CAA35395	UL36
IKHEGLVKTLVECYV	CAA35334	UL98
EGELFFFSKNLYGNG	CAA35394	UL35
GYPCVYYHVVDFERL	CAA35336	UL100(gM)
VLFYHTPDQNHIEQP	CAA35363	UL89
NDTNNTNGHATCVLY	CAA35432*	UL33
VTVTGNNMTTLPVWT	CAA35396	UL37
AIPHRQPRTQSKNQK	CAA35415	UL15
VNHVHRRRRRICHLP	CAA35368	UL94
AAGTGFGIMDYVELA	CAA35351	UL78
VTPSDLERLFAERRY	CAA35276	US9
SNDKQKDDKRSLFCY	CAA35343	UL107
YVGLLSVTTVFYTWC	CAA35280	US13
TDTRHCTSCTHPYVI	CAA35323	UL121
YMPPTVPYPDPAARL	CAA35269	TRS1part
GLTEVLAYHLYGGDG	CAA35386	UL70
RSAAVGYYDEEEKRR	CAA35431	UL32(pp150)
QNLWTDLVTRHKMSG	CAA35407	UL48(pp212)
WFFTYCDLLRVGYFG	CAA74075	UL43rev
TFVHQSHNWHNHGNK	CAA35444	UL11
RYLWTPDPSRLRSIN	CAA35447	UL14
HYHRHDGGFPLPTAF	CAA35360	UL86(MCP=majorcapsidprotein)
FVGRFVNEGVLSPDQ	CAA35423	UL24
QAATVRAEFFWGAAG	CAA35376	UL61
ATQFTTVAMVHYHQE	AAA85877.1	UL138Toledo
ATYRGRLMVMGDYSV	CAA35422	UL23
EVLINYCDIADNWVM	CAA35340	UL105
LAVGPDDEVAHLWGV	CAA35404	UL45
MRSEYGNAPVFGSGV	CAA35366	UL92
SSSTDLRSNPYPIRW	CAA35420	UL21
AVFHVICAVLLTLMI	AAA85875.1	UL136Toledo
VTTIIVLICFKFPQK	CAA35442	UL9
LHLHPRAALWAREPH	CAA35311	IRS1
PDYEDMLCYSDDMDD	CAA35384	UL69
ASSRANGTISWMANV	CAA35417	UL18
LTVGRYDCLRCENGT	CAA35448	UL16
LVQLRLDVNPDLMYA	CAA35367	UL93
DLWLYQNDTVIRNFS	CAA35448	UL16
VGLWTSMGPLIRLPD	CAA35313	US2
HFEAIFGTFCNRLEW	CAA35360	UL86(MCP=majorcapsidprotein)
FKRLHEQIRLSERHR	CAA35340	UL105
SWRPLSTVDDHKAWL	CAA35311	IRS1
ERRARRARRFCLDYE	CAA35384	UL69

VARLLRGDEEFIYHA	CAA35367	UL93
VSLMIFNSYATTAWP	CAA35432*	UL33
NQTSTVCLLCELMAC	CAA35411	UL52
PEQPSRYLRRRMFVE	CAA35394	UL35
ADEWFRHGAGEVVRL	CAA35311	IRS1
VCLPPCLSPDMASCH	CAA35404	UL45
CEEDLDGDCRQLFPE	CAA35333	UL97
VCDVEETILTPRDVE	CAA35425	UL26
DLLTRYASRREDSMS	CAA35341	UL104
QLLFYMWAGTGVMST	CAA35411	UL52
FNELMLWLGYYRELR	CAA35431	UL32(pp150)
QQQQHHHPGPPHPPL	CAA35352	UL79
PDDEVAHLWGVTPSV	CAA35404	UL45
CQVCLYELDEDEMGE	CAA35362	UL88
LGSFSSFYSQIARSL	CAA35399	UL40
CIVADSLMEFVTRGM	CAA35422	UL23
LTLGQWELVLPWIVP	CAA35359	UL85
PSVWASRGTVFEEET	CAA35404	UL45
AAAAEADRALREFLE	CAA35406	UL47
MMEMPATMHPTTGAY	CAA74074	UL42rev
ILVRWEEVSSQCSYA	AAA85877.1	UL138Toledo
LVRATDRHGDTVVYK	CAA35384	UL69
GIVYCYVIEFKTTYS	CAA35391	UL76
PWESAPQPPRLRMTP	CAA35406	UL47
YRFHDEDPETYMGFL	CAA35395	UL36
TLRLFKTTVTSPNYP	CAA35360	UL86(MCP=majorcapsidprotein)
LERTARFIKDNFSEP	CAA35413	UL54
VVLAAAAAQAAAASQ	CAA35354	UL80A
FSHAACGASLMDPLS	CAA35420	UL21
VRHALCWHRVEGGIS	CAA35358	UL84
PMGVLMNLTYLWYLG	CAA35277	US10
DLTMTRNPQPFMRPH	CAA35357	UL83(pp65)
CRFDTVEMVDETRPA	AAA85875.1	UL136Toledo
EDLDRMEAGLSPYSV	CAA35424	UL25
ARSLAADYLCCDDTL	CAA35289	US22
RQRERSAPKPQELLF	CAA35367	UL93
TMRKLKRKQAPVKEQ	CAA35389	UL74(gO)
LLFPALCFCLLCEAV	CAA35318	UL116
YATTAWPMQCEHLTL	CAA35432*	UL33
ASVDLCKSGLPRCEW	CAA35404	UL45
ALLIRMETGCDSPRH	CAA35405	UL46
IMLFLHHDSPHPPTS	CAA35356	UL82(pp71)
TFIDPLWDYLDSLLF	CAA35389	UL74(gO)
SKYTRMSSLFNDKCA	Q7M6N6	UL48A

VQKIDFVDALKTLCH	CAA35360	UL86(MCP=majorcapsidprotein)
HAVLNAGPGKRFFSP	CAA35430	UL31
CNHFLRDLLTDRFEG	CAA35446	UL12
IYWQKHSDLVYALTG	CAA35362	UL88
FHQALRRLFAPLCVH	CAA35408	UL49
DCYRSQPHPPKFLPV	AAA85880.1	UL141Toledo
THKMCELGNYHQTTP	CAA35433	TRL14
LRYSTLNTNAYDYFG	CAA35363	UL89
CGCEGRSFFRTLGTG	AAA85892.1	UL151Toledo
CVSKITFNSSCLYIT	CAA35403	UL44(pp50)
NVTELASIHPGETWT	CAA35448	UL16
VEAQHDTATPHTMWI	CAA35442	UL9
EAMFAGFEEASGDED	CAA35356	UL82(pp71)
VDAAGAPFDDDDYLD	CAA35386	UL70
RKAAHHTALHDCLAL	CAA35358	UL84
MPLGEIEGAEDKTFF	CAA35428	UL29
PLDQELIMFGVIEAW	CAA35397	UL38
FITESSVFETRASGR	CAA35427	UL28
SVESYSLQFHDRCAS	CAA35382	UL67
EDMATFRTEKQWQQD	CAA35274	US7
SVGVSSLPMRELAWR	CAA35312	US1
VELMITHFQRTIRVL	CAA35424	UL25
PNYAGALGRTAHWLF	CAA35279	US12
AVWATPCLASPWSTL	CAA35332	UL130(viralentry)
LRDTLPFWSTLLPCA	CAA35279	US12
ADTDTVWRNLFYVYY	CAA35361	UL87
TDPEHLMMNVNPLRL	CAA35340	UL105
SLAELSHFTQLLAHP	CAA35390	UL75(gH)
EDAVCWLRRTAIVMR	CAA35281	US14
RHEYTACIRMLSGDV	CAA35426	UL27
FCQDWKCHALYAEWD	CAA35264	US32
GRCQMLDRRTVEMAF	CAA35390	UL75(gH)
GGSSSRTTSNSSRST	CAA35424	UL25
TETYHLQRIYSMMIE	CAA35394	UL35
LCCPEPLRFVGSICT	CAA35422	UL23
NLAFTYGSWGVAMLL	CAA35439	UL6
SRPGFPCHVVWAPEV	CAA35283	US16
YPTAVDLAKRALWTP	CAA35390	UL75(gH)
RDADRDNYGRCVRHA	CAA35430	UL31
CFRVLPRPLELLDYL	CAA35367	UL93
GDSGGMMGRGGRMLG	CAA35372	UL57
RFSRNPSLFFSGDAL	CAA35430	UL31
TYPPGTELRLCNLRC	CAA35423	UL24
RCRQQIPWDDTHRQC	CAA35310	J1I

MYPVLLLTASPVPTP	CAA35420	UL21
LGNEIRCMLLPRQYT	CAA35419	UL20
DRFQNFEAVLARGMH	CAA35367	UL93
KSEGDVPEEFMDYVI	CAA35419	UL20
DDDEAALPGEDEAWI	CAA35430	UL31
AVDLDRPPLWSGSLP	CAA35445	UL13
LAWPHDGVYLPKDAF	CAA35353	UL80
MSPTMVTIPPPQIPF	CAA35385	UL71
LKRIRFTEDTFVETF	CAA35426	UL27
QFMGYGTKNGLKNTW	AAA85891.1	UL150Toledo
VRRYSTVSPGKEVTL	AAA85887.1	UL148Toledo
PDMASCHFGECDMPV	CAA35404	UL45
GDGVFITESSVFETR	CAA35427	UL28
VQGSRTRRPIPPILQ	AAA85892.1	UL151Toledo
LYPEYIYTVLKYPVQ	CAA35407	UL48(pp212)
ATIGAGLYIGKHFTP	AAA85881.1	UL142Toledo
ESVRRFVLRHMEDLP	CAA35404	UL45
IGPVDRSSLYEANPE	CAA35428	UL29
HLSKWLDGKKDNSWH	AAA85894.1	UL152Towne
LWVLSRGHREFYVYD	CAA35338	UL102
LVFDQQGEDAVVRRC	CAA35385	UL71
GAYFDNGWKWTFALL	CAA74074	UL42rev
WEELERKCLARIQER	CAA35431	UL32(pp150)
QRNCTHSFYLVNAMS	CAA35389	UL74(gO)
DELRYVKITLTEDFF	CAA35390	UL75(gH)
LDDFMRRQRGRHLDL	CAA35425	UL26
EAELLLPRDVVEHWL	CAA35353	UL80
AALPGEDEAWIASKN	CAA35430	UL31
YGRSIFTEHVLGFEL	P16832	UL115(gL)
YMLQVVVFFYYFYNA	CAA35340	UL105
PGVLLVWGDERLVGP	CAA35338	UL102
DMGVRVQDLFRVFPM	CAA35360	UL86(MCP=majorcapsidprotein)
GWLALGAVLPARWLG	CAA35311	IRS1
RSASLSFLDWPDGSV	CAA74075	UL43rev
TASLRALAGCMHIHA	CAA35422	UL23
VKHIDAAVFKTVRDC	CAA35406	UL47
RREHLVFMLWGADAH	CAA35316	UL114
LFTHFVGRPRHCRLE	CAA35416	UL17
SWWLMPPPVAELCER	CAA35286	US19
LRDFKELFFCLEPME	CAA35291	US24
ECNTTENGSFVAGYE	CAA35417	UL18
ESSTSWAVTSNRLPN	CAA35439	UL6
LPLMFYREIKHLLSH	CAA35413	UL54
TVMFLTRRTRDGFCG	CAA35418	UL19

QLGLHQFVDHTRGYV	CAA35359	UL85
YQEGKFIFELYRLPR	CAA35404	UL45
HNPDLSSVLEEFEVR	CAA35431	UL32(pp150)
FRLLRGIFLITLVIW	CAA35435	UL2
CQHPKKTPDPMIMFD	CAA35395	UL36
TSSVDTSPPYCRCKG	CAA35386	UL70
TRRNAVDLDRPPLWS	CAA35445	UL13
RLLECRFQQEPMGGA	CAA35360	UL86(MCP=majorcapsidprotein)
YALRYDDESWRPLST	CAA35311	IRS1
FCGMIWLGIPDSHNI	CAA35280	US13
YHVNSSDKASSRANG	CAA35417	UL18
GCSWKTFFIYNVSES	CAA35439	UL6
LFIIAFFSREPTKDL	CAA35351	UL78
SVTCYARTDCKGPFT	CAA35321	UL119
ETGGGCFLVNAGEDE	CAA35372	UL57
RRVIHDSHGLWCDCG	CAA35264	US32
YEKNTAITPYICRAL	CAA35340	UL105
FVVFIINASFIWSWT	CAA35322	UL120
QAVAPVYVGGFLARY	CAA35353	UL80
SRILPVGSMYRGSDA	CAA35265	US34
VDAFLLIRTFVARCI	CAA35360	UL86(MCP=majorcapsidprotein)
HYTMFVLDHGSVRIE	CAA35418	UL19
GLVKTLVECYVMHGR	CAA35334	UL98
RLTQIHDLLHVIETL	CAA35407	UL48(pp212)
HHLGHRKNAHTQSWY	CAA35460	TRL12
ADNNTYWYSGNAYNH	CAA35419	UL20
QCLILMSVCAFCWLV	CAA35288	US21
DNFFAERTSGCITRL	CAA35436	UL3
VLQTKAHIHPGFALT	CAA35360	UL86(MCP=majorcapsidprotein)
ISCVENCNLTRKCLH	CAA35339	UL103
LRGNQRNRIRWWQHN	CAA35296	IRL14
NLDCDPEVMAVYEIL	CAA35372	UL57
QRIEENLEGVRRNMF	CAA35431	UL32(pp150)
CAALSDDIKRYVTEF	CAA35360	UL86(MCP=majorcapsidprotein)
VGPQVTELYERYQHE	CAA35407	UL48(pp212)
EQPPRQRRRMVSVTL	CAA35394	UL35
GGLIGSVIDLPLWCL	CAA35408	UL49
TPVEDVSESLVAKRY	CAA35278	US11
EMRFTIAWMWFPSVL	CAA35262	US30
KNSVRHMSSFVSSDI	CAA35406	UL47
DMSDESYRLGQGSFG	CAA35333	UL97
KFDLMSLLEREESWR	CAA35263	US31
VRNQNDNRAEAFCTS	CAA35417	UL18
RVSVSELEAVYREIL	CAA35338	UL102

DRNRERNPGSPQLLP	CAA35447	UL14
VYCALRLLACPDRPI	CAA35424	UL25
MEPTPMLRDRDHDDA	CAA74074	UL42rev
LLSYRGDPLVFKHTF	AAA85887.1	UL148Toledo
IKAFSKNGLLWCEYV	CAA35289	US22
TTVWEKRRMESDTDF	CAA74073	UL41alt
LDFYWVLPGGFAVSS	CAA35367	UL93
ATDAHTPLLQACREL	CAA35360	UL86(MCP=majorcapsidprotein)
SRQTTIMVTKYSEKS	CAA35360	UL86(MCP=majorcapsidprotein)
VQFGYHLGAFFGLCG	CAA35336	UL100(gM)
GIPLKEEHVAYVDRF	CAA35340	UL105
TVPYPDPAARLCRDM	CAA35269	TRS1part
SWLERHCPPLDQELI	CAA35397	UL38
CLAYRSLTRKKLEQH	CAA35458	TRL10
VPMEAVRHPLLFWRR	CAA35261	US29
NGTNRNASYFGENAD	CAA35414	UL55(gB)
VCELAFSFASVFFDS	CAA35372	UL57
GTHKYVLERDDEAVL	CAA35338	UL102
LVVVTEFSEIFGIPQ	CAA35406	UL47
LSGAQTLHLRLVWPD	CAA35367	UL93
RERASGVHLQRYVRA	CAA35427	UL28
AHTQSWYWLRILTSH	CAA35460	TRL12
YQPAESMLFSEWPLV	CAA35372	UL57
TWDLFTYPIYAVYGT	CAA35320	UL118
IHNKRCTDLDFGDLL	CAA35340	UL105
LQDTVSESEFIVRYH	CAA35460	TRL12
YAVDVLKSGRCQMLD	CAA35390	UL75(gH)
RVTYDGELIYGSYLL	CAA35369	UL95
VIYNNTQGCGYKYDW	CAA35430	UL31
RFCLDYEPVPRKFRR	CAA35384	UL69
AQVLNHAVCLDAELH	CAA35386	UL70
ELASDLAGFFAKGMI	CAA35428	UL29
GKVLHLNKGWLCATI	CAA35315	UL113
RVYELVSETLFGQRC	CAA35408	UL49
YMSQRLSSLEKDHLM	CAA35372	UL57
MIDLTSHHRPLTLFT	CAA35319	UL117
VMTKPYFVFLAYVYS	CAA35408	UL49
YSVSSDAPSSFELVR	CAA35424	UL25
FDEQSLLDLTVFAGT	CAA35384	UL69
VTVIMIYVLIHFNVP	CAA35434	UL1
RWGTAYSSGSSASSS	CAA35263	US31
KKTPDPMIMFDEDDD	CAA35395	UL36
LVVAVVSLGRWDVVT	CAA35322	UL120
DFDFLRLPRGGGQVW	CAA35391	UL76

GHHEDENFYLLVTPK	CAA35460	TRL12
LGRYETVWCLDRDRG	CAA35293	US26
EQESSSFFHSKAHFF	CAA35382	UL67
VVIQAYVLSSMLCVW	CAA35279	US12
MWSRVVFLRSSETQT	CAA35447	UL14
IDDDTPMLLIFGHLP	CAA35390	UL75(gH)
VAWITCAALGIWCLA	AAA85872.1	UL133Toledo
FLRRHDVLERFAAAA	CAA35369	UL95
GLRNYAPLREELGYV	CAA74075	UL43rev
VNHDLFRWSVMTAMI	CAA35443	UL10
HLAYNPFRMPTTSTA	CAA35431	UL32(pp150)
MCTDPRRTAGWERLT	AAA85884.1	UL145Toledo
FRSGRFDLCTDSVLD	CAA35391	UL76
AWPMQCEHLTLRRTI	CAA35432*	UL33
CNEGVKAAWSLKELH	P19893	UL122(IE2)
AEQTWYADVVRCVRA	CAA35367	UL93
ANNRVSFHGVKNMRI	CAA35403	UL44(pp50)
FRNQQVNKRLTKKNH	CAA35455	TRL7
TNQPPIFQIYYLLHA	CAA35412	UL53
KQLVLFLRACLLKLH	CAA35361	UL87
VVDFERLNMSAYNVM	CAA35336	UL100(gM)
AVSSLAWPHDGVYLP	CAA35353	UL80
GFTYFASWDLIERIF	CAA35404	UL45
GFGIMDYVELATRTL	CAA35351	UL78
HHYPSAAERKHRHLP	CAA35357	UL83(pp65)
SLFPVYHVGKLLDAL	CAA35369	UL95
FTVNVDSLCVDAEQR	CAA35430	UL31
PHACPHYAVPFTTPG	CAA35430	UL31
PADEEAEDSVFTSTR	CAA35424	UL25
RLHRRTVLFNELMLW	CAA35431	UL32(pp150)
VDLTFFFPVGLYLPE	CAA35360	UL86(MCP=majorcapsidprotein)
LLAELMARSVAHNLY	CAA35406	UL47
LSMDTFQLFTLTMSF	CAA35336	UL100(gM)
NAPFPHLRWPVDLIP	CAA35423	UL24
NTFLLHQEGFRNLPF	CAA35334	UL98
DEPCCTPALGRYSLG	AAA85880.1	UL141Toledo
CLRHQLDPPLLRHLD	P16832	UL115(gL)
CLLQRTVTTYVCRLP	CAA35348	UL110
ATEYFALLHGIQTFS	CAA35407	UL48(pp212)
LLLSSHWVPYPLRIP	CAA35436	UL3
ATRTTSPNALLPEWM	AAA85875.1	UL136Toledo
GGDWADSASDFDADC	CAA35449	TRL1
RDCYERFVCPVYDSG	CAA35277	US10
YYTSAFVFPTKDVAL	CAA35357	UL83(pp65)

IEALRDADRDNYGRC	CAA35430	UL31
WRKLFGGDDPGPTCR	CAA35338	UL102
VGFVAVAVTDEQCCL	CAA35338	UL102
TTNLVSKWMTQHFQS	CAA35372	UL57
YVCEEHLHSFTEKGD	CAA35363	UL89
KNITNLAFTYGSWGV	CAA35439	UL6
YKHFFCDPQCAGNIR	CAA35411	UL52
RDCVFDIATTLEHLS	CAA35406	UL47
KRAVEKRKQDSTRQK	CAA35452	TRL5
TWFPEHVRGTDPRHV	CAA35373	UL58
FYYFYNALGDTRLYR	CAA35340	UL105
RVLQYLIHAFQIDFL	CAA35363	UL89
CVLPPLFHGPLAREN	CAA35359	UL85
LQQQYDWLCLTERFD	CAA35291	US24
DMYPVCMAKTNSPNY	CAA35413	UL54
NTNGHATCVLYFVAE	CAA35432*	UL33
RVRILCGDTGTVYAA	CAA35428	UL29
AIKFHDLNKLTTGKM	CAA35360	UL86(MCP=majorcapsidprotein)
REQQRLDKSLAATAP	AAA85890.1	UL149Toledo
TMYGGISLLSEFCRV	CAA35325	UL123(pp72=MIprotein=IE1)
PHYAVPFTTPGKPGC	CAA35430	UL31
LYAAILCLDKVCRQL	CAA35404	UL45
LVVTRVWPPLLEHLT	CAA35386	UL70
ARDCFTHPESVAPAY	CAA74075	UL43rev
QIEIHIPCNVHVSPG	CAA35415	UL15
LFSRDVSWYHSMSFS	P09704	US28
YQKLDALTELYRDPQ	CAA35363	UL89
RNASYFGENADKFFI	CAA35414	UL55(gB)
LPTNHEREILDLMRH	CAA35385	UL71
HQCLQAAKKRPKTHK	CAA35273	US6
FVAYAVARNRRDYTE	CAA35386	UL70
FLHTQLVMVPFVPHA	CAA35430	UL31
FSPLGSFTRLGYDRL	CAA35362	UL88
QLRRAYQEHRRRKHL	CAA35361	UL87
NNTNGTHVNPWVCEE	AAA85896.1	UL154Towne
LATRSLAWVDCCRVA	CAA35282	US15
IGVMLVLIVAILCYL	AAA85877.1	UL138Toledo
VKDQWHSRGSTWLYR	CAA35414	UL55(gB)
ENYRQGPGFLLEKQH	CAA35457	TRL9
DVARVQDSVSRDLGF	CAA35426	UL27
LSRLKTTPNKHTQHK	CAA35443	UL10
SQRLVGEFMVRDPLL	CAA35424	UL25
MRPGLPPYLTVFTVY	CAA35390	UL75(gH)
TAYESYLVSITEQYN	CAA35430	UL31

PREPPHRALFRLCLG	CAA35445	UL13
ITHFQRTIRVLRCYL	CAA35424	UL25
QHNSKKCNQTEKWHN	CAA35433	TRL14
TWTFSFGIWYYRLKR	CAA35392	UL77
RRLITADEERRGPER	CAA35341	UL104
RMETGCDSPRHLYIS	CAA35405	UL46
IPIQYAAVDLTIKMW	CAA35351	UL78
FDRTVVIQAYVLSSM	CAA35279	US12
LFRLCLGLWVSSYLV	CAA35445	UL13
LATSDGLYLYNAFRR	CAA35333	UL97
HDICFDCNDTSLTIY	CAA35433	TRL14
DAAAEVLSWCGLPDI	CAA35338	UL102
QRLWWEIQYSSGRLT	CAA35323	UL121
WTHLYDVLFRGFAGQ	CAA35338	UL102
PVVGYDQLAARHFAD	CAA35353	UL80
PGPLVHPSLYIGFPC	CAA35375	UL60
FGSLISLLMAFMYYH	AAA85894.1	UL152Towne
MFDSGVDRDYARQFR	CAA35290	US23
SDEALTETVWLHDDD	CAA35386	UL70
DDPERYNIHFEAIFG	CAA35360	UL86(MCP=majorcapsidprotein)
RARLPDTVCVHYVYL	CAA35386	UL70
WCLTLFVLWMLRVVG	CAA35417	UL18
LRQDIRHLVRSYADM	CAA35293	US26
NVAPGERDLTRRIIT	CAA35424	UL25
RAVRDEIYAVLRRDG	CAA35367	UL93
TVSLGITSLLTCVMR	CAA35405	UL46
GAHPGARCHVCLPGA	CAA35282	US15
TRQQNQWKEPDVYYT	CAA35357	UL83(pp65)
RSLMKRTHRASRHAV	CAA35430	UL31
DERLVGPFNFFYGNG	CAA35338	UL102
LIGLLHQTPHMWARS	CAA35393	UL34
VVTKKDNQCMTDYDY	P09704	US28
NIYERIPYRPSRQKD	CAA35458	TRL10
ALREFLEAPWESAPQ	CAA35406	UL47
CPFLYPSPPRSPLQF	CAA35332	UL130(viralentry)
SITWLFATTLFIGYM	CAA35327	UL125
NLLHHHPPHDLPALP	CAA35386	UL70
LLGNSVDALYIRERL	CAA35353	UL80
SMHCRSRHQRTPPSA	AAA85891.1	UL150Toledo
VQSSTLIRVLFYHTP	CAA35363	UL89
TSAGEEMFEALRIYY	CAA35360	UL86(MCP=majorcapsidprotein)
LIITKQFYGGLIFHT	CAA35438	UL5
DFSFQWFRSISRVER	CAA35340	UL105
GVSGLARHTVFELCR	CAA35386	UL70

RPRRKRCLVPEVFCT	CAA35428	UL29
RVLRCYLQHQLQSIS	CAA35424	UL25
RVQRLLWHSLRHGDA	CAA35426	UL27
FSTTVLPRVHGPRSS	CAA35293	US26
ADSAVSHETLERYRV	CAA35340	UL105
VMVRIFSTNQGGFML	P19893	UL122(IE2)
VVLRELYRRVVSYCQ	CAA35411	UL52
WNVVRCRGTGFRAHD	CAA35449	TRL1
WMTGTAECPTVKSPH	CAA35292	US25
NNGALTLVIPSWHVF	CAA35356	UL82(pp71)
MEARAIIRRTAHHWA	CAA35356	UL82(pp71)
EIAYRDVIHTTLRRM	CAA35291	US24
FLSYRWLIRCCELYG	AAA85877.1	UL138Toledo
ALYYVHFPFFSDLAN	CAA35282	US15
RWWLYSGWWWLTFGC	CAA35261	US29
LCHVLIGLLHQTPHM	CAA35393	UL34
SDAPSSFELVRETGG	CAA35424	UL25
LAAGSVALTSLCHLL	CAA35447	UL14
QYEFMGLIFTVNVDS	CAA35430	UL31
DLLKYMEFGIPLKEE	CAA35340	UL105
HPGFALTAVRTDTFE	CAA35360	UL86(MCP=majorcapsidprotein)
IRVSRCRKKREGSPK	CAA35355	UL81
TNLYSTNFLTLTVLP	CAA35432*	UL33
IVAAAYDDSKFCRYV	CAA35341	UL104
HDGVYLPKDAFFSLL	CAA35353	UL80
QIIYNFYTFFMCLYV	CAA35406	UL47
LGLPQSAWRRWRSHV	CAA35439	UL6
QRLQINDLLAYWPVI	CAA35340	UL105
ALDAYRARIAVEYVL	CAA35367	UL93
LHQTPHMWARSIRLI	CAA35393	UL34
LINLSMDRYCVIVWG	CAA35259	US27
IYVQLPSLREDYAQL	CAA35424	UL25
WVLPPPLPPRPPHLI	CAA35419	UL20
PTAREGELFFFSKNL	CAA35394	UL35
SGTVLRLSWPNGWFF	CAA74075	UL43rev
ERVAERCDDRHGGSD	CAA35334	UL98
HNTLTALNTPSRTHH	CAA35310	J1I
YVRAILESERRIREG	CAA35397	UL38
ATFYCPFLYPSPPRS	CAA35332	UL130(viralentry)
RHSYLKDSDFLDAAL	CAA35390	UL75(gH)
YCVFGTCSIGTTTAP	CAA35437	UL4(gp48)
VQVFIDLRTEHSYAL	CAA35386	UL70
RKLDQSDWVRGAWIV	AAA85884.1	UL145Toledo
AALRGVPLPPDPQHF	CAA35426	UL27

SFGEFFENGLFAVYS	CAA35427	UL28
GKITTETYHLQRIYS	CAA35394	UL35
SGLVYRELHDFYGYL	CAA35265	US34
VEEPVSRMIVCSCPV	CAA35368	UL94
VYRHDEVDRWIRHAA	CAA35360	UL86(MCP=majorcapsidprotein)
GALNLCLPLMQKFPK	P19893	UL122(IE2)
TLPYLCHILALGTLD	CAA35341	UL104
ERLHRHDRRGLFRFR	CAA35400	UL41
RLVLDEAFPTFPLYD	CAA35407	UL48(pp212)
RAPDGGLNLDDFMRR	CAA35425	UL26
MLLVKCQELLMRLDR	CAA35359	UL85
LSDAELSNHANRCRR	CAA35426	UL27
LFVLWMLRVVGMHVL	CAA35417	UL18
PIPQRLHLIKHYQLG	CAA35359	UL85
FNYLDLSALLRNSFH	CAA35390	UL75(gH)
HVMLRTEDGIITAAK	CAA35291	US24
DENFYLLVTPKNHTE	CAA35460	TRL12
EDDFCHKICYAVDMS	CAA35333	UL97
RVYQKVLTFRRSYAY	CAA35414	UL55(gB)
TSTYNSLTISSFTST	CAA35442	UL9
FCNRLEWVYFLTSGL	CAA35360	UL86(MCP=majorcapsidprotein)
QVNKRLTKKNHLGIK	CAA35455	TRL7
RFSGHNGIYDRVPDC	CAA35423	UL24
PVRRYSVWCGMSSRL	CAA35293	US26
TPSRTHHAAPHRRCF	CAA35310	J1I
HTFSGVRRPFTELGW	AAA85887.1	UL148Toledo
RLVQALKRAMYSVEL	CAA35351	UL78
RVKRKKLQTFGYLSF	CAA35453	TRL4
QHFRWLNAGAFRRLV	CAA35426	UL27
VRLFFSSLFEYRKHN	CAA35443	UL10
GVTLLVVAVVSLGRW	CAA35322	UL120
GPCRDPPRGFTVFRT	CAA35377	UL62
LFVDDVGLYSTALFF	CAA35351	UL78
ALTARFFVPEGLVEF	CAA35405	UL46
HCGRFLRYHLLPLLL	CAA35445	UL13
LVFDPHSSAECHNAA	CAA35407	UL48(pp212)
TVPRITCYHQLLGAL	CAA35395	UL36
PSYEEQESSSFFHSK	CAA35382	UL67
IMATQLRDLATWVYT	CAA35389	UL74(gO)
APPRPKKCQTHAPHH	CAA35311	IRS1
LLRNSFHRYAVDVLK	CAA35390	UL75(gH)
GRLGVGPQVTELYER	CAA35407	UL48(pp212)
RRERWRTTNTDRWCS	CAA35381	UL66
AVSEFMKNTHVLIRN	CAA35389	UL74(gO)

FTEEIQLHSLYACTR	CAA35366	UL92
LSRFNVGDFHGASWE	CAA35265	US34
SVDTVLYQPPPSWKP	AAA85874.1	UL135Toledo
FEIQRSRHETGIFTF	CAA35352	UL79
EERYAMACLPRDLSL	CAA35361	UL87
VGYYDEEEKRRERQK	CAA35431	UL32(pp150)
SALFVMLRQLDDLIR	CAA35359	UL85
ATEKIPFVENAVLKE	CAA35272	US5
VFRVPEQPSRYLRRR	CAA35394	UL35
LHAETTRTWRWAQRG	CAA35449	TRL1
NFYTFFMCLYVYSPT	CAA35406	UL47
QVLRLFYDLRDLKLC	CAA35407	UL48(pp212)
CVVKDGVLDAVWRVQ	CAA35275	US8
PPPPPRHPSCSPTMV	CAA35409	UL50
SMRKLNKPTCPYGVD	CAA35422	UL23
GAEEAIVYSNYTVER	CAA35341	UL104
EAWEEASVRPTRQLV	CAA35397	UL38
AFAYLPGEDKVYVLG	CAA35427	UL28
ALLFAFLHYFTTLKQ	CAA35417	UL18
HHHRPCVPARRPRYS	CAA35384	UL69
LNQVFLCPTSPSWFI	CAA35386	UL70
ATDDEWTLQKVFYLC	CAA35360	UL86(MCP=majorcapsidprotein)
PPPNCASQVGGPLCY	CAA35349	UL111
LFSTVTPCLHQGFYL	CAA35390	UL75(gH)
GPPIPRPATRSSDPS	CAA35415	UL15
YCHTRYETFLRVMGD	CAA35290	US23
RLLAVWDDEPLSLFL	CAA35334	UL98
VLIEAALRQFVHDSQ	CAA35372	UL57
VCHSQHERPSLYHDL	CAA35460	TRL12
WYVNPFSLAHLLDAI	CAA35318	UL116
ARTGSLHHFELSYRF	CAA35395	UL36
YAHPASELRPGSGGW	CAA35312	US1
PRTGGRFIRRTASGT	CAA35415	UL15
NTGNGSKCHAMCKCR	CAA35458	TRL10
SVAASAARFDEIRRR	CAA74075	UL43rev
WKCHALYAEWDGKES	CAA35264	US32
FKLRRGCRAPPTPET	CAA35426	UL27
YSGEYDVLITDGDGS	P16845	UL22A
LGAVLPARWLGCAAG	CAA35311	IRS1
ASTPALFDFLRVVRQ	CAA35424	UL25
RLPALLRERVSVSEL	CAA35338	UL102
CGFLVRIELGVYFFS	CAA35383	UL68
KKREDALLKQMRSEY	CAA35366	UL92
LVSNFTSNISARWFR	CAA35440	UL7

HDLCWLFRRLFFPRE	CAA35416	UL17
TLFKAFQITKRTYKG	CAA35411	UL52
MNPVDQPPPPLPTQQ	CAA35426	UL27
QTMELMIRTVPRITC	CAA35395	UL36
SGPPAEQTWYADVVR	CAA35367	UL93
EICEEHTLNDLAFLV	CAA35424	UL25
EPLRFVGSICTYNFL	CAA35422	UL23
YAAVDLTIKMWFLLG	CAA35351	UL78
RVRMFYAVFTTLGLR	CAA35367	UL93
HFLARGLLGFFRVGF	CAA74075	UL43rev
TARFVELAGCWGLYA	CAA35404	UL45
LRSAACRRRPGDLGF	CAA35451	TRL3
YTVPFSGPSVPWRDE	CAA35381	UL66
IMATYLCDDIHAIRF	CAA35363	UL89
ALRADMLEFGLRNCQ	CAA35404	UL45
LFTHCTDRYPLSHNV	CAA35371	UL56
NLYTTVNPLIEDVMR	CAA35406	UL47
RCRDGQLSLSTFTMS	CAA35404	UL45
RARMLQHNGPQQSHH	CAA35396	UL37
FARLLLGSPFRQRVS	CAA35386	UL70
RSNNTDTIFVSLLTG	CAA35419	UL20
WRGWLLFPALCFCLL	CAA35318	UL116
AFVNPRHQYYFQMLI	CAA35334	UL98
LKRKQAPVKEQFEKK	CAA35389	UL74(gO)
LQRYTAESLRLAPYP	CAA35384	UL69
TPIRNRQEAVRAGLL	CAA35426	UL27
LTVYPCSACNRSVLH	CAA35276	US9
WPVPTAYKAFLWKYA	AAA85886.1	UL147Toledo
HSPQWASLLQLHHGL	CAA35280	US13
CRWVRYCSCSCKCSC	AAA85872.1	UL133Toledo
GITQNDPFIRFHTDF	CAA35392	UL77
VCWIKISMRKDKGMS	CAA35336	UL100(gM)
TKIPVLANRVLQYLI	CAA35363	UL89
HRRRRRICHLPTLYQ	CAA35368	UL94
LLLGYVLARTVYRVS	CAA35275	US8
HYRYEVANLTYNCTY	CAA35437	UL4(gp48)
GAGAWLLPRPEGYTL	CAA35368	UL94
WREHLYCVYDSHFQR	CAA35264	US32
YVLARTVYRVSSAYY	CAA35275	US8
LNEHSQLSERVAYHL	CAA35399	UL40
TYALVSKDLASYRSF	CAA35390	UL75(gH)
APVVGYDQLAARHFA	CAA35354	UL80A
LNITNSGRYSRRCFK	CAA35460	TRL12
DVTFGVLKRALVRLH	CAA35394	UL35

LKLSVVRLDNFSVEL	CAA35405	UL46
GGLIFHTTWVTGFVL	CAA35438	UL5
EILRQLETTISTKYN	CAA35389	UL74(gO)
LLIPKSFTLTRIHPE	CAA35356	UL82(pp71)
HNGIYDRVPDCPKGR	CAA35423	UL24
IERQQLQQVVHHVRV	CAA35386	UL70
RFPTRENRTKTRSFL	CAA35453	TRL4
VIWTVVWLKLLRDAL	CAA35435	UL2
KAHIHPGFALTAVRT	CAA35360	UL86(MCP=majorcapsidprotein)
KQMRSEYGNAPVFGS	CAA35366	UL92
RHWGAEAYEPLIRII	CAA35352	UL79
NTRHRERLGYNSKFY	CAA35360	UL86(MCP=majorcapsidprotein)
LLELEIALGYRSQSV	CAA35417	UL18
RGSYPEFLYSLGVYR	CAA35361	UL87
NLSLPPSNALSSKDY	CAA35442	UL9
HLRHYLQNSFLHLLM	CAA35393	UL34
PPLVEKYWRMRTTHT	CAA35375	UL60
DVRRFLLCMRVYSTV	CAA35284	US17
RLSTQLLTRVHNHIL	CAA35407	UL48(pp212)
GRLMVMGDYSVIRVS	CAA35422	UL23
VDSLCVDAEQRQLLG	CAA35430	UL31
ANGTISWMANVSAAY	CAA35417	UL18
REFVFYLNGTYTVVR	CAA35320	UL118
FACRIQKKKDRRRGV	CAA35421	UL22
SARRLICSALYLLFE	CAA35339	UL103
RIILRGKTLLLSSHW	CAA35436	UL3
GVQLSPAFYYEALFL	CAA35407	UL48(pp212)
TQSRWTIHHMYNKLL	CAA35440	UL7
ITVRRVAEEWKLHAA	CAA35341	UL104
LMSVCAFCWLVLPHR	CAA35288	US21
PAWKTVRAFSRAYHH	CAA35264	US32
GLFFFSNGKSESYTF	CAA35379	UL64
SNYTVERVTLPYLCH	CAA35341	UL104
WQARLTVSGLAWTRQ	CAA35357	UL83(pp65)
RELIGRCLPAALCRG	CAA35426	UL27
FCDTCPRYLVPLRAL	CAA35408	UL49
RVPQYDFLISADPFS	CAA35404	UL45
LLLTRIRFSNQRCFG	CAA35445	UL13
SSRFQRTQRTMRREK	CAA35282	US15
LQLHKTHLASFLSAF	CAA35390	UL75(gH)
PLVIVTTIIVLICFK	CAA35442	UL9
FMPLTYPPGTELRLC	CAA35423	UL24
LVAVVCRQCGHCLNL	CAA35408	UL49
HAGFQVRAASVMTRR	CAA35445	UL13

LICSREKARRRQMSR	CAA35341	UL104
SGCITRLASDSLCLF	CAA35436	UL3
DHYPPPLPPSSRHAL	CAA35384	UL69
FVGAVPRRVPLPQMG	CAA35415	UL15
DTGIYHHGRSVRLPY	CAA35386	UL70
VGYGKVIRCIKTQER	CAA35371	UL56
ACAVYDHRLAFDVIL	CAA35408	UL49
YRVCILGVEARLRPY	CAA35375	UL60
DIYRRLLRVFPSFVA	CAA35391	UL76
RSSNTLQHMSKKQES	AAA85882.1	UL143Toledo
RVVEQVLRLFYDLRD	CAA35407	UL48(pp212)
VFVPHNRQGLKMPVT	CAA35358	UL84
MLIQQYVLSQYYIKK	CAA35334	UL98
RFCNNYCFARDCFTH	CAA74075	UL43rev
YLVLPNCCQVSVDRS	CAA35429	UL30
AGRITPPPADFQQPV	CAA35407	UL48(pp212)
RFSNLEQRVARLLRG	CAA35367	UL93
ACRSTIFSPEDDSSC	CAA35368	UL94
PVERQALTELEYQAM	CAA35311	IRS1
MDWKAHVEYAHPASE	CAA35312	US1
YMEFGIPLKEEHVAY	CAA35340	UL105
VTLFTVNRTCDLLTP	CAA35398	UL39
TQGVINIMYMHDSDD	CAA35390	UL75(gH)
TVECVNDILDACSHP	CAA35365	UL91
ERTLEDALAVELVNE	CAA74075	UL43rev
IAEKMWAENYETTSP	AAA85880.1	UL141Toledo
ELDAMDEDELQQLSR	CAA35384	UL69
LMGGATQEPLDTGLY	CAA35269	TRS1part
GGRGGGGGGDSGGMM	CAA35372	UL57
VQYDDYWNAVMLYRG	CAA35395	UL36
QIFYNFHEGKITTET	CAA35394	UL35
RSHVRQHAHTMDDLV	CAA35397	UL38
PNYEISWLKQNKTYI	CAA35417	UL18
RQHAHTMDDLVMVFH	CAA35397	UL38
FWSTDLEQMTDSVRR	AAA85887.1	UL148Toledo
DAITDAELMDHTSLY	CAA35340	UL105
MCLSFDSNYCRNILK	CAA35431	UL32(pp150)
FETSGGLVVFWQGIK	CAA35414	UL55(gB)
DAELMDHTSLYADPF	CAA35340	UL105
MEANKRDRQHQLATT	CAA35367	UL93
VEIGRVLSVSEVDDY	CAA35361	UL87
FDEIRRRRQSINDEM	CAA74075	UL43rev
ALGHWALLSICTVAA	CAA35396	UL37
RTQMELDAADYAACA	CAA35449	TRL1

RHALELQMMQDWVVE	CAA35394	UL35
MPSTSYGSDVEDPRD	CAA35394	UL35
RKNAPMESGEEEFLL	CAA35259	US27
MNSLLAELNRLGVAH	CAA35365	UL91
KIERIVDKVKSLSRE	CAA35340	UL105
STLTKYAESDYIFLQ	CAA35396	UL37
TCKVILLNNTKNPDI	CAA35321	UL119
PISGHVLKAVFSRGD	CAA35357	UL83(pp65)
VMEACVFEHEFEKIK	CAA35344	UL108
LFLYMLDVATVPEAE	CAA35407	UL48(pp212)
SAWLRDDVCDLQKRP	CAA35384	UL69
LSPRDAWIVLVATVV	CAA35311	IRS1
DEETTVWEKRRMESD	CAA74073	UL41alt
HARFPVAGLRRYCMS	CAA35333	UL97
TTVMTERQSQLPEKY	CAA35311	IRS1
LYEANPELRLPFKKR	CAA35428	UL29
GEPSAAEGDEFSFCD	CAA35424	UL25
TGNDGGGGDQIMGDK	CAA35430	UL31
KVYLESFCEDVPSGK	CAA35357	UL83(pp65)
LKWIGPHTRVKRNVK	CAA35403	UL44(pp50)
GPHTRVKRNVKKAPC	CAA35403	UL44(pp50)
PRYRCLSFDIECMSG	CAA35413	UL54
VTSVEDVQRVLARAP	CAA35407	UL48(pp212)
RRACEDAIRCDYGVF	CAA35289	US22
LCELMACSYYDNVVL	CAA35411	UL52
ARHVGEFNVLKVNES	CAA35409	UL50
YVYSPVVESLYLVSR	CAA74075	UL43rev
TTSDSTCFLTRLNNA	CAA35363	UL89
RLFFPREDSEPLMSD	CAA35416	UL17
PRRRVYSVRCDHCVE	CAA35344	UL108
DMFDGVVASAYHRLR	CAA35362	UL88
VVKVARKHSETVLTV	CAA35333	UL97
FVVNDGTRYQMCVMK	CAA35332	UL130(viralentry)
LIREQVVFTVCDVSP	CAA35359	UL85
FLRHCDVEPAIATFD	CAA35374	UL59
LWGADAHTCEYLIDR	CAA35316	UL114
DMVQAVEAVWQRLEP	CAA35407	UL48(pp212)
YLGPDLFETGAARSF	CAA35338	UL102
PETLQIFYNFHEGKI	CAA35394	UL35
LYELDEDEMGEEMLG	CAA35362	UL88
FELLPEFTEEEEEKE	CAA35419	UL20
RNSWVASEDELDVSR	CAA35418	UL19
HVCTTVDYGLTSRTA	CAA35340	UL105
YTLFVCDVEETILTP	CAA35425	UL26

VCALFNQLVFTAQLR	CAA35393	UL34
SRFQRFWETPTLIMK	CAA35387	UL72
SVGVNSKVRACVIGY	CAA35340	UL105
RFLREWLVCRQAERE	CAA35408	UL49
SDDVLFALDPYNEVV	CAA35390	UL75(gH)
RVLSVSEVDDYVTAV	CAA35361	UL87
DRLREVIASVGELVP	CAA35413	UL54
GLGGLGGGGGGGGKK	CAA35403	UL44(pp50)
MRELAWRRVADDSHD	CAA35312	US1
SVIRCLGGYCDLIRE	CAA35404	UL45
RQHVLYNGCCVVTAP	CAA35360	UL86(MCP=majorcapsidprotein)
TQHRSPSRCFMAMGE	CAA35374	UL59
RLELVASVFEHLTVE	CAA35365	UL91
VIYEVCMLVPQDEAK	CAA35368	UL94
WQHNESVVDLWLYQN	CAA35448	UL16
ASIVATRVEDMATFR	CAA35274	US7
PEVISVMKRRIEEIC	CAA35325	UL123(pp72=MIprotein=IE1)
DWSEINDWRVMVGSN	CAA35422	UL23
NPWVCEEPKHEWDTS	AAA85896.1	UL154Towne
GGGSVIGSTGGNDET	CAA35340	UL105
SPGWIEANSVTFKRQ	CAA35415	UL15
GSAGVSTSLCSVERM	CAA35369	UL95
KQRTVPRCFCCGSLQ	CAA35457	TRL9
TLTEDFFVVTVSIDD	CAA35390	UL75(gH)
VGATDLRQLSPRDAW	CAA35311	IRS1
VSRREIWRLADSVDM	CAA35293	US26
ATPSFLRRHDVLERF	CAA35369	UL95
ALDDVNIISAVLSGV	CAA35366	UL92
GEVFQGDKYESWLRP	P16832	UL115(gL)
FAMSEADSLEMLLDK	CAA35385	UL71
LLLARQRDGLADWNV	CAA35449	TRL1
DAQRLASYLCCPEPL	CAA35422	UL23
VMGLVDMDCEKSAYM	CAA35385	UL71
YYHGVHSRELRCPCT	AAA85894.1	UL152Towne
RRDADGQVIRESACY	CAA35425	UL26
DEEHRTQMELDAADY	CAA35449	TRL1
AQPGALLIRMETGCD	CAA35405	UL46
EAARLHFTMFDSGVD	CAA35290	US23
QSHNWHNHGNKWTLD	CAA35444	UL11
DGLYLYNAFRRTTSI	CAA35333	UL97
RCFGCVGARPGGCVP	CAA35310	J1I
VRSKELPSDHESLEA	CAA35326	UL124
CAVMAPRTLILTVGL	CAA35399	UL40
MGLIFTVNVDSLCVD	CAA35430	UL31

QSKCAFLKGYLSEGC	CAA35385	UL71
PIISTRISTVNLYLS	CAA35406	UL47
SLSFRLITETAGTYT	CAA35447	UL14
AGNRGKKTIITEYRI	CAA35294	UL131
ELLGKATQQLPYLSA	CAA35407	UL48(pp212)
EEFIYHAGPLEPPSK	CAA35367	UL93
RGIFLITLVIWTVVW	CAA35435	UL2
TCGQVGPDVDVYEFP	CAA35413	UL54
MYCFLFLQKDTFFHE	CAA35451	TRL3
TKVCTDYTSVTCTPC	AAA85883.1	UL144Toledo
TRTHYLRQLCSMTEE	CAA35426	UL27
AARALLADYAETFSP	CAA35362	UL88
TCDGFNYTVHKRCDR	CAA35461	TRL13
FPGGETARKDKFLHM	CAA35413	UL54
GAPPSSGNNSNFWHG	CAA35311	IRS1
NEVRPHAGVIDCAPF	CAA35338	UL102
GTILDKILNVEAMHT	CAA35360	UL86(MCP=majorcapsidprotein)
LFNDKCAFKLDLLRM	Q7M6N6	UL48A
LRRLNECIPMPAFAL	CAA35424	UL25
TTVMSTLTKYAESDY	CAA35396	UL37
ECLVETTEAVFRLRQ	CAA35275	US8
QSATRGDSDMFDGVV	CAA35362	UL88
FLGDMQLPADNFLTS	CAA35430	UL31
EGIQLMGGATQEPLD	CAA35269	TRS1part
LIAVSVLSSRSKESL	CAA35276	US9
KKLVEMMEQHDRGSD	CAA35341	UL104
VATSFCHRVSDKITA	CAA35430	UL31
RFWETPTLIMKEESA	CAA35387	UL72
GSYEEGFVVIREQLS	CAA35407	UL48(pp212)
SVRDGLFCLGCVTSP	CAA35353	UL80
LLNCADNNTYWYSGN	CAA35419	UL20
DVQHLRRLNECIPMP	CAA35424	UL25
VSGYRVSSSVSECYV	CAA35276	US9
THVKTSAGEEMFEAL	CAA35360	UL86(MCP=majorcapsidprotein)
MVEAIRTTVRGDTVR	CAA35424	UL25
TTPVAHLHEEILRYD	CAA35341	UL104
IADYNDGGDMGSRFD	CAA35296	IRL14
VSSHLLTVTQSRWTI	CAA35440	UL7
TTTFFLGDMQLPADN	CAA35430	UL31
DPNVMRRHANDDFYK	CAA35388	UL73(gN)
LIPEETGVTRPMMSL	CAA35385	UL71
YRARIAVEYVLIRAV	CAA35367	UL93
SSQIRTRWEESNVVS	CAA35445	UL13
TVYPTYDCVLSDLEA	CAA35392	UL77

RYLGYMSQRLSSLEK	CAA35372	UL57
NPDLMYATDPHDRDE	CAA35367	UL93
YGMMYTEAVYRQPQT	CAA35290	US23
QLSYLMTGTVRDNVP	CAA35407	UL48(pp212)
DHIFMDILTTCVETM	CAA35325	UL123(pp72=MIprotein=IE1)
GDYMSRIILCCEQNE	CAA35385	UL71
RQAFPGLDFEAAVFD	CAA35352	UL79
VDMDCEKSAYMLEAG	CAA35385	UL71
VYAVHGLHTLMRETA	CAA35338	UL102
AVQTFCDTCPRYLVP	CAA35408	UL49
ELWFPLYFEAECNRN	CAA35318	UL116
INIMYMHDSDDVLFA	CAA35390	UL75(gH)
LIAALYPEYIYTVLK	CAA35407	UL48(pp212)
DKILNVEAMHTVLRA	CAA35360	UL86(MCP=majorcapsidprotein)
QMGAPTAERVARRRS	CAA35415	UL15
TTSIRGLTCDPRMFL	CAA35333	UL97
SLHPIDVNGSLFWHQ	CAA35328	UL126
AAYGHALWEGRDPPD	CAA35392	UL77
VLDSSSSGGDSGPED	CAA35369	UL95
GKLHLSYNATAQELL	CAA35320	UL118
WKLLVVTQGQLRVIG	CAA35425	UL26
FNYTVHKRCDRSYEV	CAA35461	TRL13
RCLCYVPCGPMTQSL	CAA35368	UL94
PSPPWSKLTYSKPHD	CAA35332	UL130(viralentry)
RLLVPWIRESKMWVL	CAA35419	UL20
ARFIKDNFSEPCFLH	CAA35413	UL54
TQTEALESRYDHYTQ	CAA35340	UL105
FLSSAVRIVSVGTNY	CAA35349	UL111
HLGRNANVLTVCRHV	CAA35361	UL87
KAFMEANGNHPEQIC	CAA35424	UL25
QLVARFFFRLTGQDE	CAA35339	UL103
QLLTQLHEANVYLCP	CAA35394	UL35
QDESVAHLLAATRRR	CAA35391	UL76
MTTSAMTAPDTRRQL	CAA35429	UL30
AEDVVMFTCVMGKKG	CAA35395	UL36
VLRHLNQVFLCPTSP	CAA35386	UL70
SWAVTSNRLPNCSTI	CAA35439	UL6
LICSALYLLFEEKEP	CAA35339	UL103
RLSLDEVKKYGVPRG	CAA35413	UL54
VEEMLRYVESKPTNE	CAA35407	UL48(pp212)
DIPCLSNHHDDSDAI	CAA35340	UL105
EAGRQEPETPRVSGR	CAA35367	UL93
INEDLDEGIMVVYKR	CAA35414	UL55(gB)
VSVYDELTASEMEEP	CAA35295	UL132

LVDRACEFFYFDVSR	CAA35293	US26
GSLFWHQNQRDFPKC	CAA35328	UL126
GVNESAFGLTHLQSC	CAA35399	UL40
IADNWVMFIHNKRCT	CAA35340	UL105
LLLSRMEALEWFKKF	P09724	US20
VGLLCMRIRSLLCSP	CAA35399	UL40
NEYRTGISWSFGMLF	CAA35336	UL100(gM)
EHEAIPKVASLFWTL	AAA85891.1	UL150Toledo
VNSFMKGIRDVGFGK	CAA35446	UL12
GYISEHVTSACASAG	CAA35338	UL102
DMQLPADNFLTSPHP	CAA35430	UL31
SSDKASSRANGTISW	CAA35417	UL18
VPTTQFCRGPLLYVH	CAA35361	UL87
TLHVNHSCDYSVRQS	CAA35318	UL116
PPPVPEEKGGGEGGG	CAA35375	UL60
KMVFLISISFLLVSF	CAA35389	UL74(gO)
GRRLSTELLDVMQKY	CAA35386	UL70
IQEFMITCLSQTPPR	CAA35390	UL75(gH)
FETGGDVGREFMLAR	CAA74075	UL43rev
LDAASQSDPLPGGDG	CAA35296	IRL14
GPAHTRFQGPDSMPS	CAA35394	UL35
HPSGTVPRRRSMPAP	CAA35415	UL15
LRDLGHRVQTYCEDL	CAA35392	UL77
KYSIMADSVCLPPCL	CAA35404	UL45
PLLGNFMYLTSSKDL	CAA35403	UL44(pp50)
SGHRPRPPAPPRPKK	CAA35311	IRS1
RVKRNVKKAPCPTGT	CAA35403	UL44(pp50)
QDSAVASGPGRIPQP	CAA35333	UL97
EDTVFDLKDVDEWFE	CAA35290	US23
CDPVHESICARLQPN	CAA35428	UL29
LRDCVYELAPTMKDF	CAA35395	UL36
SDRNALWREMDTVSR	CAA35404	UL45
SENGNLQVTYVRHYL	CAA35315	UL113
FDPSRYLRQHGARCP	CAA35415	UL15
AVELVNETFRCSVTS	CAA74075	UL43rev
ATITFGRITCCHPLA	AAA85882.1	UL143Toledo
HVETLRRFLRGDSCF	CAA35429	UL30
DRLSAISYGRDLWHH	CAA35351	UL78
FDARADLAVYHRNQW	CAA35408	UL49
TPTPTPLSEAMFAGF	CAA35356	UL82(pp71)
QSPAADFSVSEAWRF	CAA35369	UL95
YSPIVIQDCAAAVTD	CAA35369	UL95
DMGLGYTSNTCVAYV	CAA35360	UL86(MCP=majorcapsidprotein)
HQLLGALGHEVPERK	CAA35395	UL36

ARKHSETVLTVWMSG	CAA35333	UL97
GYNSKFYSPCAQYFN	CAA35360	UL86(MCP=majorcapsidprotein)
SLLDEAEWRQTQMDV	CAA35397	UL38
LFFVHARHDTLLPHR	CAA35400	UL41
LDLRNLLHHHPPHDL	CAA35386	UL70
RGAWMPAETFTCPKD	CAA35449	TRL1
DDYVTAVSGYLGEAA	CAA35361	UL87
LDFGDLLKYMEFGIP	CAA35340	UL105
GDEDYSGEYDVLITD	P16845	UL22A
CVFARSDEQKLHLPD	CAA35404	UL45
RRICHLPTLYQLSFG	CAA35368	UL94
PSCEEDERELCVPFI	CAA35447	UL14
PVVESLYLVSRSGFR	CAA74075	UL43rev
RLCRCEACMGRCGCA	CAA35409	UL50
ACGASLMDPLSPSRW	CAA35420	UL21
YCDLIREKEVHRPVV	CAA35404	UL45
LSRPDEPCCTPALGR	AAA85880.1	UL141Toledo
QAMGAVWRAAFLANS	CAA35311	IRS1
NMTTARESSVHDARN	CAA35295	UL132
HHPPHDLPALPLSPP	CAA35386	UL70
CYDDLRDCVYELAPT	CAA35395	UL36
SHFHEAALAVRYETP	CAA35282	US15
RAPSHRVMHQMQQTL	CAA35407	UL48(pp212)
VYYELARDLGSHGTE	CAA35361	UL87
SIRNPSYAAEMTRLF	CAA35340	UL105
LEQRVARLLRGDEEF	CAA35367	UL93
KNHFWKNGDISYTET	CAA35329	UL127
TYLRETFEGTILDKI	CAA35360	UL86(MCP=majorcapsidprotein)
ECQDPVRRMLLDKEQ	CAA35413	UL54
VSWRPESPSPDGTPS	CAA35404	UL45
PPPMCRVPYNECGVE	CAA35289	US22
EEPFQRGDPFDKNYV	CAA35403	UL44(pp50)
GGWREDVLMDRVRKR	CAA35392	UL77
ETTTCTNTTTTVTCD	CAA35461	TRL13
MTDSVRRYSTVSPGK	AAA85887.1	UL148Toledo
MLLRYITFHREKVLY	CAA35444	UL11
ARRGGGGGVGSNSSR	CAA35358	UL84
EMWMACIKELHDVSK	CAA35325	UL123(pp72=MIprotein=IE1)
APVSADWFRFSGRSP	CAA35364	UL90
EQRQLLGTVATSFCH	CAA35430	UL31
ESLKTFEQVTEDCNE	P19893	UL122(IE2)
TKRGYASYTIDDPFD	CAA35395	UL36
GNCCLDAPPVVRSPC	CAA35447	UL14
RDRHRRDRRDSGEYC	CAA35424	UL25

GKSLHVCTTVDYGLT	CAA35340	UL105
RISELDNEKVRNIMK	P19893	UL122(IE2)
HEYLSDLYTPCSSSG	CAA35390	UL75(gH)
AHELVCSMENTRATK	CAA35357	UL83(pp65)
SLNVSSMSGQDYRFM	CAA35372	UL57
RGTMDDDEAALPGED	CAA35430	UL31
DAWIVLVATVVHEVD	CAA35311	IRS1
PLPQRLLPRQFPPPP	CAA35415	UL15
SAFARQELYLMGSLV	CAA35390	UL75(gH)
CRVPYNECGVELPGG	CAA35289	US22
DVLYNTRHRERLGYN	CAA35360	UL86(MCP=majorcapsidprotein)
SSPSPRHTFDMDMME	CAA74074	UL42rev
YLMGSLVHSMLVHTT	CAA35390	UL75(gH)
GEKKELPAQAALSPP	CAA35261	US29
HPLSQMNHPPLPDPL	P19893	UL122(IE2)
NYFTGHHEDENFYLL	CAA35460	TRL12
RTTRSPVRLRVNIRN	CAA35415	UL15
HHHHHHNAVTDVAAG	CAA35315	UL113
AHDSEDGVSVWRQHL	CAA35449	TRL1
DAADEVWALRDQTAE	CAA35431	UL32(pp150)
SRHAGWDGRGLLGPC	CAA35377	UL62
AYSSGSSASSSGFVA	CAA35263	US31
SVRWHATHSLQYAEG	CAA35391	UL76
VRAQLDLRNLLHHHP	CAA35386	UL70
VSEEDPAAAATPSSS	CAA35397	UL38
SWRRVVDYSHNLWCT	CAA35263	US31
CVLSYVESRFHNKFL	CAA35372	UL57
PSSPPSLKDSCHLCA	CAA35420	UL21
FQNVQSLHRRVRIFM	CAA35331	UL129(viralentry)
IRQSVQSSTLIRVLF	CAA35363	UL89
PATMHPTTGAYFDNG	CAA74074	UL42rev
TMARKIGEYLLEQGF	CAA35413	UL54
EMALKAFMEANGNHP	CAA35424	UL25
RILLSHDELMHTDYL	CAA35384	UL69
TGGNDETAFFQDDDT	CAA35340	UL105
RVWPPLLEHLTQHYE	CAA35386	UL70
TFRPPPCGTVPSMTC	CAA35389	UL74(gO)
RRLAILCLLSFMLPI	CAA35279	US12
TIYNVTTEHAGKYVL	AAA85895.1	UL153Towne
KLCGAMISSSCSTTC	AAA85891.1	UL150Toledo
LLGRLNVYEVARFSL	CAA35334	UL98
LQISEKMSRGQPLSS	CAA35352	UL79
FFFQFLSSAVRIVSV	CAA35349	UL111
QLDPPLLRHLDKYYA	P16832	UL115(gL)

TTPRHDICFDCNDTS	CAA35433	TRL14
DDSWKQLGEDFAHQC	CAA35273	US6
PVVPEECYDQRFTTE	CAA35311	IRS1
DRPPLWSGSLPHLPV	CAA35445	UL13
NLVLQEIETDEDFKP	CAA35423	UL24
AMVAFINQTSNIMKN	CAA35412	UL53
SYLMTHAGRYADVIQ	CAA35366	UL92
SGLLPSCEEDERELC	CAA35447	UL14
GEINITFIHYGDKVP	CAA35417	UL18
ASEMEEPSNSTSWQI	CAA35295	UL132
APLDHAQRQGLPDFI	CAA35360	UL86(MCP=majorcapsidprotein)
LIEDVMRSSAGSLRN	CAA35406	UL47
NTSSNTGNGSKCHAM	CAA35458	TRL10
WSEWGNCCLDAPPVV	CAA35447	UL14
DKHADEEHRTQMELD	CAA35449	TRL1
GMSLNQSTRDISYMG	CAA35336	UL100(gM)
SEPEDDDEDPTYDEL	CAA35311	IRS1
GKLTVTSVEDVQRVL	CAA35407	UL48(pp212)
YRACPIHVRTEPERV	CAA35361	UL87
LTTFVKHIDAAVFKT	CAA35406	UL47
QRALAVFDVLSRENL	CAA35406	UL47
RPPPDYEETLRLFKT	CAA35360	UL86(MCP=majorcapsidprotein)
INDWRVMVGSNHVEP	CAA35422	UL23
DALNCPLLNEPNVFS	CAA35430	UL31
RRGRVKIDEVSRMFR	P19893	UL122(IE2)
TSSMTCDGITPDVIY	CAA35368	UL94
RLRVWWLSDAGVRET	AAA85875.1	UL136Toledo
VTQNINTVDMGLGYT	CAA35360	UL86(MCP=majorcapsidprotein)
CDLQKRPPETFSQPM	CAA35384	UL69
TRLSYGRSIFTEHVL	P16832	UL115(gL)
YRRVVAYDADAAPEA	CAA35386	UL70
GLFWVGRDPPNPPEC	AAA85894.1	UL152Towne
PQTSDLECAKQYWQE	CAA35293	US26
RRNSNLFVFCTERDY	CAA35393	UL34
TNVIPRESAEVPVLD	CAA35369	UL95
VDPHYPGWGRRYEPA	CAA35353	UL80
FMARAEAALKDLYAA	CAA35426	UL27
SIASARWEALRADML	CAA35404	UL45
STMQPSTLETFPDLF	CAA35390	UL75(gH)
GALQCHECQNEMCEL	CAA35384	UL69
YDWLCLTERFDPHEG	CAA35291	US24
DVPRLGAMDADEPLF	CAA35360	UL86(MCP=majorcapsidprotein)
PDLTLSSLTLYQDGI	CAA35358	UL84
VCNGIMFLHALHLGG	CAA35407	UL48(pp212)

TETLERYQQRLNTYA	CAA35390	UL75(gH)
HFGLLCPKSIPGLSI	CAA35357	UL83(pp65)
VSYARDELTKRCGEK	CAA35393	UL34
PLPPRDYPQRDERDR	CAA35424	UL25
FLLHRKTMKLAACLC	CAA35362	UL88
IFCNQNYTCRVTHGN	CAA35417	UL18
YGYTGIFDDTSHMTL	CAA35417	UL18
VYSQYNHTAKTITFR	CAA35389	UL74(gO)
LRDAIHELKRDLFAA	CAA35353	UL80
DRPSFYRAFRSGRFD	CAA35391	UL76
LYRERRVPCIICVGS	CAA35340	UL105
RDYPQRDERDRHRRD	CAA35424	UL25
VPPDEREEDTLREMA	CAA35424	UL25
TLVPILLHEQKKAFY	CAA35434	UL1
QQVHAAHDVWCNCGD	CAA35312	US1
VQNAFTEEIQLHSLY	CAA35366	UL92
QWFRSISRVERTTDN	CAA35340	UL105
PWRDIDSVTFVPRNL	CAA35318	UL116
SPLSMLSSASPSPAK	CAA35431	UL32(pp150)
HLPLYNEFTSFRLPT	CAA35319	UL117
RLPGDCYRSQPHPPK	AAA85880.1	UL141Toledo
LEHAELRLERNRHLG	CAA35426	UL27
SIFPGTIEGDIGVFP	CAA35387	UL72
NNLDLGCILDHQDGW	CAA35384	UL69
NNAPFDMLNVVSYVC	CAA35363	UL89
LLHELDAASQSDPLP	CAA35296	IRL14
EEMKCIGLTMQSMYE	CAA35325	UL123(pp72=MIprotein=IE1)
PDILSVTCYARTDCK	CAA35321	UL119
QLGNECCPPCGSGQR	AAA85883.1	UL144Toledo
LPRPLELLDYLRQSG	CAA35367	UL93
CDPGASLRRLWLPSV	CAA35286	US19
CEPSRNRTAVSEFMK	CAA35389	UL74(gO)
RQRREERKKMAAART	CAA35369	UL95
LNVYEVARFSLPAFV	CAA35334	UL98
SESEFIVRYNPEHED	AAA85896.1	UL154Towne
LATVVMEAGGQMIHK	CAA35269	TRS1part
DRHRRDRRDSGEYCC	CAA35424	UL25
GVGMRRCPTGSRPTP	CAA35261	US29
EEILRYDGLCRHQKI	CAA35341	UL104
IGTGKMRITPDAFEI	CAA35352	UL79
HDTFLEVVDVFGMRQ	CAA35407	UL48(pp212)
IDSYRRAFCTLADAI	CAA35333	UL97
VFGHTEGQVLLTMAY	CAA35386	UL70
QAMQHAGFQVRAASV	CAA35445	UL13

EAEGENKQFTEHTHK	CAA35429	UL30
MPLSGGRHYHPGTFD	CAA35392	UL77
SKSNPVADYMFAQSS	CAA35392	UL77
AQCAERPLPGNVPSY	CAA35392	UL77
FDLKDVDEWFEQRRL	CAA35290	US23
LNIKGLEKTFLCCDK	CAA74075	UL43rev
PLHGVVGGFAAGHCG	CAA35338	UL102
TESCINRGESYLTTI	CAA35419	UL20
LCCEQNERHRRAGTM	CAA35385	UL71
RHMDPEQDYRLPAQD	CAA35430	UL31
LDEAETQPLYRHLFR	CAA35386	UL70
VLVATVVHEVDPAAD	CAA35311	IRS1
DKQRTKNTHPSGTVP	CAA35415	UL15
FPPRCSDAGTIRNTS	CAA35415	UL15
TCNAFYGFTGVVNGM	CAA35413	UL54
WEHRLSSVWRDALFT	CAA35281	US14
LLLHVESFLRAAKDL	CAA35400	UL41
GSFTRLGYDRLVSAD	CAA35362	UL88
RFFVPEGLVEFEAQP	CAA35405	UL46
AECAAHMIISVLSLH	CAA35363	UL89
VSESEFIVRYHTEHE	CAA35460	TRL12
PFDHMPAADFRDLLN	CAA35289	US22
DDDDDEKNIFTPIKK	CAA35431	UL32(pp150)
RIFEHMYFAAVRASV	CAA35404	UL45
AAEAARRLLPELDRE	CAA35311	IRS1
IATTQKEGHLYTVNC	CAA35321	UL119
NTASTTFVTSVFSTP	CAA35460	TRL12
GSDDYVWLSRLLDLA	CAA35334	UL98
GLYASENYNGNYELT	CAA35441	UL8
GTIIPTHASMGEFAR	CAA35386	UL70
TAVSGYLGEAAAPRI	CAA35361	UL87
QLRHYCEHQDKVVSY	CAA35393	UL34
VVTQGQLRVIGTIGL	CAA35425	UL26
YVLERDDEAVLARLF	CAA35338	UL102
RFIINYVGKWHMQTL	CAA35259	US27
SDDIKRYVTEFPHYH	CAA35360	UL86(MCP=majorcapsidprotein)
GCWGLYAAILCLDKV	CAA35404	UL45
FFSGDALNCPLLNEP	CAA35430	UL31
LIMNVRRSWEELERK	CAA35431	UL32(pp150)
EVQRVMGLVDMDCEK	CAA35385	UL71
AAYPTYLDGERAKGD	CAA35417	UL18
LYYLAHSLDDFARHG	CAA35293	US26
FQRNYFYIGRADAED	AAA85880.1	UL141Toledo
RTTTTTTTKKTTTTS	CAA35444	UL11

TAYLHMFHPHTSPPF	CAA35267	US35
STHGHHLGHRKNAHT	CAA35460	TRL12
RLSERHRLFDLPVYC	CAA35340	UL105
LSFPSPTYHESQMIN	CAA35453	TRL4
AQMSYGACLLGEHGA	CAA35333	UL97
SQLSERVAYHLKLRP	CAA35399	UL40
FPPPDPPSQPAEDAR	CAA35445	UL13
FKFFHQDPNRVLDCI	CAA35319	UL117
PSMAQFWHGAIVLEY	CAA35397	UL38
YYAYDLAMSFRVGTH	CAA35338	UL102
GSGCALVSTLEGSVC	CAA35366	UL92
TRFQGPDSMPSTSYG	CAA35394	UL35
CRLVLFVDDVGLYST	CAA35351	UL78
SPRSDHTLFPVPSTP	CAA35449	TRL1
MRRAPAEAAEAPPQS	CAA35315	UL113
ETDERGQWIMLFLHH	CAA35356	UL82(pp71)
NIISWSSFFDVLLSS	CAA35344	UL108
SNYTNLTEAFRFTPA	CAA35419	UL20
IQRNYLKGNCTQWSV	CAA35417	UL18
PSPQYQHVATHALWV	CAA35444	UL11
EALWTLRGNLSVPTP	CAA35278	US11
HYNRSTHGHHLGHRK	CAA35460	TRL12
HIYSDSLTFVAESIT	CAA35422	UL23
HLSQNMRDMYLDMCT	CAA35311	IRS1
FPPSPPPSPGPMHMV	CAA35419	UL20
NTGTEVDQCLAYRSL	CAA35458	TRL10
VNLNDTFQSIFPGTI	CAA35387	UL72
KPPHPATSTASHHPH	CAA35449	TRL1
NVVTPKAAYAELFFL	CAA35430	UL31
SSCLYITDKSFQPKT	CAA35403	UL44(pp50)
AHFVVIGWMEPVNKA	CAA35427	UL28
SQSDLIIRPTIWLPG	CAA35387	UL72
PPDSVATVLGELPQL	CAA35392	UL77
VPSVYMPPTVPYPDP	CAA35269	TRS1part
AQSVQDTIQHMRFLY	CAA35407	UL48(pp212)
SVCVKTGLVYEALYP	CAA35366	UL92
PVYDPSSSPKKTPEK	CAA35407	UL48(pp212)
KQVEDSIYFTFNKVF	CAA35366	UL92
SKQPVKNLTMNMTEF	CAA35389	UL74(gO)
VYTASAARAALQWLD	CAA35405	UL46
SAATASSSSSSPPSR	CAA35428	UL29
EPSVVAPQHVEYLKF	CAA35424	UL25
FLTLALLSDADWLQK	P09724	US20
EVSYPIILNVELMLG	P09704	US28

LTSSIAHWQTLVDVA	CAA35405	UL46
RRWKKTVLRCGLEID	CAA35269	TRS1part
PSGGRRGRARNNTRG	CAA35315	UL113
LDDYPYLMVEIGRVL	CAA35361	UL87
CHECQNEMCELRIQR	CAA35384	UL69
LSFSIVAAALWKVDY	CAA35323	UL121
FQSIHGAFSTTSSRK	CAA35372	UL57
VVFTEDHMLVFDPHS	CAA35407	UL48(pp212)
STWLAQCAERPLPGN	CAA35392	UL77
PEAVLAARALHMPTL	CAA35352	UL79
TINGNVTFRGLQNKT	CAA35437	UL4(gp48)
ADPLGDSPQPVELWF	CAA35340	UL105
VLSRENLERRGAQLC	CAA35406	UL47
SMSFVYLHSVESYSL	CAA35382	UL67
LFTTEDSLDKLIAWM	CAA35284	US17
KRCLVPEVFCTRDLA	CAA35428	UL29
VISTSQHDTNRVTWF	CAA35373	UL58
MSLLHTFWRLPVAVF	CAA35392	UL77
SMAEYRSELLCTSAF	CAA35339	UL103
AFVFPTKDVALRHVV	CAA35357	UL83(pp65)
VDLFYRPAFLLMPAA	CAA35360	UL86(MCP=majorcapsidprotein)
ALHDHRLWPPFVTHL	CAA35360	UL86(MCP=majorcapsidprotein)
RCFSCVPRDPCCRPP	CAA35310	J1I
CNVTQWGRYENGSTP	CAA35439	UL6
YTEKLEEIDSKPDTI	CAA35442	UL9
EEEADKQRTKNTHPS	CAA35415	UL15
QEPETPRVSGRRLPF	CAA35367	UL93
VTAVQRALAVFDVLS	CAA35406	UL47
DWIRFLSLPDHDTVL	CAA35316	UL114
LVSYTIKLSHDPIEY	CAA35363	UL89
RLNTEEESEAAEETA	CAA35447	UL14
YILSKQNQQHLIPQW	CAA35390	UL75(gH)
IYILVGTQMRKDMWT	CAA35259	US27
TFEVDMLLYSGKSCT	CAA35360	UL86(MCP=majorcapsidprotein)
VVGEFAMSEADSLEM	CAA35385	UL71
LRAVAQVLNHAVCLD	CAA35386	UL70
RRLHRLWCFCQDWKC	CAA35264	US32
SKNVQYEFMGLIFTV	CAA35430	UL31
REEYHGVYEHLAGLI	CAA35407	UL48(pp212)
NLQARDASGLMFPII	CAA35406	UL47
TKLPKYDPDEFWTKA	CAA35433	TRL14
RALENGKLTHCSDAC	CAA35333	UL97
EANALLLSRMEALEW	P09724	US20
YAETFSPLGSFTRLG	CAA35362	UL88

WTSCTSLSPCSTSCP	P09724	US20
MKGKHSRYTRPTEPP	CAA35269	TRS1part
LYAVAFAWVLSIVAA	CAA35351	UL78
YGRQHGPALIAWVEE	CAA35407	UL48(pp212)
TRFVQRHTGLPVVFP	CAA35352	UL79
SPHPPTSVFLHFSVY	CAA35356	UL82(pp71)
PHSAESTVRHDASEN	CAA35445	UL13
CELGNYHQTTPRHDI	CAA35433	TRL14
VFFVLCLSASSSCAV	CAA35448	UL16
VYELAPTMKDFLRNG	CAA35395	UL36
SQSYMDRLQVSGEQY	CAA35314	US3
ATFLSKKQEVNMSDS	CAA35414	UL55(gB)
SVTFADTDTVWRNLF	CAA35361	UL87
GAYFEWNIGGHPVTH	CAA35314	US3
VLHSVMTLAAMLYKI	CAA35360	UL86(MCP=majorcapsidprotein)
VWDDEPLSLFLMNTF	CAA35334	UL98
PLGQMIVPPTPDIGF	CAA35387	UL72
YVCVEGTEQLIENPC	CAA35360	UL86(MCP=majorcapsidprotein)
SPCLQPVRDRNRERN	CAA35447	UL14
GQLRVIGTIGLANLF	CAA35425	UL26
TEKHELLVLVKKAQL	CAA35390	UL75(gH)
VADLLKWIGPHTRVK	CAA35403	UL44(pp50)
LLWHSLRHGDAPQDR	CAA35426	UL27
KYHMLQDTVSESEFI	CAA35460	TRL12
QLITNNDTRSNNTDT	CAA35419	UL20
FSLPAFVNPRHQYYF	CAA35334	UL98
TLFKTIDEYLLRAKD	CAA35360	UL86(MCP=majorcapsidprotein)
GYDLRFSRNPSLFFS	CAA35430	UL31
FCHRVSDKITARNMP	CAA35430	UL31
PSYAAEMTRLFLSHV	CAA35340	UL105
KARHMVEAIRTTVRG	CAA35424	UL25
ATLRLLIQDGMYGRG	CAA35323	UL121
SRYLRRRMFVERPET	CAA35394	UL35
LHFALCTVISCVENC	CAA35339	UL103
FYKVGNITLYTELHP	CAA35360	UL86(MCP=majorcapsidprotein)
LPGVIFVSVGGGPPL	CAA35421	UL22
ITADPTHPHHGSRTH	CAA35407	UL48(pp212)
CYVMHGREPVRDGLG	CAA35334	UL98
LIWVQCLILMSVCAF	CAA35288	US21
MRLARDESPRPTFFD	CAA35407	UL48(pp212)
SRDTALAADIGYGVY	CAA35338	UL102
YFHTRWIKSLQENHT	AAA85881.1	UL142Toledo
HAVNQRSCLRRPCGP	CAA35407	UL48(pp212)
YLLTSAQRGYDLRFS	CAA35430	UL31

YVLVTVNSLARAVVF	CAA35407	UL48(pp212)
LQDQYTGDVEAFYLI	CAA35459	TRL11
RCLVPWVPLWSSLED	CAA35278	US11
AGNVPRDSIPCITNV	CAA74075	UL43rev
RRRSLPQQRRRPSSS	P09724	US20
LETLGCVKTVSLGIT	CAA35405	UL46
LARQAVQTFCDTCPR	CAA35408	UL49
DLILGALQCHECQNE	CAA35384	UL69
DVVFASELCFYCSGR	CAA35362	UL88
RPEARCRQQIPWDDT	CAA35310	J1I
SNSSRSTSPVAPGEP	CAA35424	UL25
RRPPPLPQRLLPRQF	CAA35415	UL15
AEVPVLDSSSSGGDS	CAA35369	UL95
PDVPREAVMSPTMVT	CAA35385	UL71
AYPSFGTLPASHAQY	CAA35435	UL2
SASSSSRICPLSNSK	CAA35276	US9
FIPANIPNKIQNTRS	CAA35415	UL15
ECYFPTVVRRPLRVP	CAA35404	UL45
LCELADPLGDSPQPV	CAA35340	UL105
TTRLFEIAWSEADLL	CAA35284	US17
RLSFFDDYGNTKSYL	CAA35451	TRL3
AGGQKKKTPAPKHPK	CAA35426	UL27
DLFCLPLGESFSALT	CAA35390	UL75(gH)
IGKHFTPVKFVYEVW	AAA85881.1	UL142Toledo
LKGENAPFPHLRWPV	CAA35423	UL24
EEPELEDDDEYDELW	CAA35318	UL116
YTSRMVTNLTVGRYD	CAA35448	UL16
GTCSIGTTTAPVEWK	CAA35437	UL4(gp48)
MNQGLCVYYSDEEED	CAA35293	US26
TCPNVLHSVMTLAAM	CAA35360	UL86(MCP=majorcapsidprotein)
LTEVESRRWWWAVRA	CAA35291	US24
TVQTARDPLYAAEQL	CAA35406	UL47
LPDVVLGNTRRYFDL	CAA35404	UL45
GVSFIIYKFTYTTPP	CAA35327	UL125
AMLTACVEVWARELL	CAA35338	UL102
GTLSRHPHRPHPQQQ	CAA35352	UL79
AELCRRSRASARGRY	CAA35428	UL29
ITKAGEDALRPWKST	CAA35313	US2
CSHNLSIILYEEESQ	CAA35318	UL116
PRPVDSYVMLHSNAR	CAA35272	US5
LHRRLETLGCVKTVS	CAA35405	UL46
RYASRREDSMSLGAR	CAA35341	UL104
WHNVDWISKQPLRGR	CAA35296	IRL14
HVDLDFGVVADLLKW	CAA35403	UL44(pp50)

TSPRFLEIVRRASEK	CAA35353	UL80
RASTRHHELRYKWLI	CAA35293	US26
LTETVWLHDDDVAST	CAA35386	UL70
VVHYIPGTSGLLPSC	CAA35447	UL14
MRDYQRPMVQYDDYW	CAA35395	UL36
KRYVTEFPHYHRHDG	CAA35360	UL86(MCP=majorcapsidprotein)
STVSPGKEVTLQLHG	AAA85887.1	UL148Toledo
LRENTTQCTYNSSLR	CAA35390	UL75(gH)
SLGRYALWIYNIYSS	CAA35380	UL65
HGTDVNRTSNTTSMN	CAA35396	UL37
RACVIGYQGTVERFV	CAA35340	UL105
VERPQLLDTETISML	CAA35360	UL86(MCP=majorcapsidprotein)
PPPDKFDLMSLLERE	CAA35263	US31
RYETFLRVMGDFQGI	CAA35290	US23
PRSNVMRGAVSEFLP	CAA35356	UL82(pp71)
VILTQLETLSRPDEP	AAA85880.1	UL141Toledo
ELYRDPQFQQINNFM	CAA35363	UL89
HYPGWGRRYEPAPSL	CAA35354	UL80A
RQHSSPAFQPMLYNL	CAA35394	UL35
SQTMLHEYVRKNVER	CAA35333	UL97
GMVRKSYTHVAWTIV	CAA35313	US2
VKEFCLRHQLDPPLL	P16832	UL115(gL)
DFKKWLDGGFSTAVE	CAA35363	UL89
PRVYYQTLEGYADRV	CAA35278	US11
GRLEKQVSGTPSTVP	CAA35431	UL32(pp150)
NVERVRAVFAALYRA	CAA35361	UL87
DDVSREIAAWEGPVA	CAA35392	UL77
AGNPYEDDDYYYYRE	CAA35275	US8
ITCYHQLLGALGHEV	CAA35395	UL36
VTLGDGLDYHIGVKD	CAA35368	UL94
ELVGGFSKIIYIQNS	CAA35420	UL21
WLGCAAGPATGTAAG	CAA35311	IRS1
CLLAWLAFPDVQGQC	CAA35285	US18
SMKPINEDLDEGIMV	CAA35414	UL55(gB)
YAVSVTPATKTSIYG	CAA35413	UL54
VFIFVDRLFQHFSFL	CAA35365	UL91
TCDLLTPPPWYPITV	CAA35398	UL39
MREIIAVGNFSKYML	CAA35343	UL107
GISTFFLSVTLFTVN	CAA35398	UL39
TENSGKYYFKREDAN	CAA35437	UL4(gp48)
APQPPRLRMTPDTDH	CAA35406	UL47
CMSELSALGNVLGFC	CAA35333	UL97
LYCVYDSHFQRRPTT	CAA35264	US32
LSKISWLERHCPPLD	CAA35397	UL38

FFFSKNLYGNGEVFR	CAA35394	UL35
LTLSTIRSILAADER	CAA35341	UL104
APHVAFIRRRRPPHH	CAA35310	J1I
VRRSWEELERKCLAR	CAA35431	UL32(pp150)
GVPGGGAGGGGGRDV	CAA35372	UL57
MPAMTNNRACGLGLN	CAA35360	UL86(MCP=majorcapsidprotein)
IYNAQDHTVVRVLRP	CAA35407	UL48(pp212)
CPLLNEPNVFSLTVH	CAA35430	UL31
CARDTCFGREKNGCP	CAA35369	UL95
SALARKSRGLAGVTR	CAA35296	IRL14
LLPEPRRDHHDGMVA	CAA35291	US24
AAVFFHATFMARAEA	CAA35426	UL27
YYEALFLYMLDVATV	CAA35407	UL48(pp212)
STNFLTLTVLPFIVL	CAA35432*	UL33
NDPFIRFHTDFRGEV	CAA35392	UL77
EYVFSGRSVLDSVSG	CAA35427	UL28
AVFCRVHGLRGFQVS	CAA35396	UL37
ALLGRLYFISSKHTL	CAA35358	UL84
YQFGQIGYFSGNGVE	CAA35372	UL57
RFKVCDVGRRHIIPG	CAA35395	UL36
RIPHYPPSWSRTIPN	CAA35436	UL3
INDLLAYWPVIADIV	CAA35340	UL105
LILMVCASESSTSWA	CAA35439	UL6
LLEEGDEEDEVTVMS	CAA35385	UL71
FSGRSSMVTVLCPDL	AAA85876.1	UL137Toledo
RFLTSKVNYSNDMDN	CAA35322	UL120
APLLERPLPVYRVHL	CAA35386	UL70
NPSPRHDVSAYLCSL	CAA35450	TRL2
HLHRSLSGGPDVLYA	AAA85890.1	UL149Toledo
PCVPARRPRYSKDDD	CAA35384	UL69
MAFAYALALFAAARQ	CAA35390	UL75(gH)
VFDPYGRQHGPALIA	CAA35407	UL48(pp212)
LFRVFPMNVYRHDEV	CAA35360	UL86(MCP=majorcapsidprotein)
YLYNTLKTEVFALMI	CAA35405	UL46
RCHENGIYYGTRSMR	CAA35422	UL23
RFTSRNTAPRWYKRD	CAA35373	UL58
FLNIGLRRCNFITVP	CAA35422	UL23
HYLMYSHTNNECVGE	CAA35259	US27
KQVSGTPSTVPATLL	CAA35431	UL32(pp150)
VPTHGLLYTVLNTGP	CAA35359	UL85
PWSRLIITKQFYGGL	CAA35438	UL5
VSPERPAFMEHSRPV	CAA35456	TRL8
EICMKVFAQYILGAD	CAA35325	UL123(pp72=MIprotein=IE1)
GDLHRFLFGVDLRLP	CAA35408	UL49

DLFSGDEDSDSSDGY	CAA35431	UL32(pp150)
MDARAVAKRPRDPAD	CAA35405	UL46
GLWCDCGDWREHLYC	CAA35264	US32
HPSLEHGGVLPSFFF	CAA35422	UL23
FVACNGVAWEHRLSS	CAA35281	US14
FKTVRDCVFDIATTL	CAA35406	UL47
VPGTESLERFLTQLW	CAA35341	UL104
CTVRVRTPYTVSVND	CAA35383	UL68
SFLFQAEESGPRRLE	CAA35365	UL91
LREEEDDDDDEDFED	CAA35336	UL100(gM)
VTNVLSPVFPGGETA	CAA35413	UL54
QNYVKNSVRHMSSFV	CAA35406	UL47
YTRHHRDNGQEENYY	CAA35433	TRL14
TLIEYSLPVPFHRFY	CAA35360	UL86(MCP=majorcapsidprotein)
GNNSQSVATSLDTNS	CAA35432*	UL33
GGGGRDVSGGPSDGL	CAA35372	UL57
QVLFVRHVLMPRDVA	CAA35283	US16
FGTLPASHAQYGFRL	CAA35435	UL2
ANVYLCPGYLHFSAY	CAA35394	UL35
AYQELCELADPLGDS	CAA35340	UL105
AHGTPVAEDFMTRVA	CAA35334	UL98
EGRVNYASLLAFSHH	CAA35406	UL47
QWGVPTIIVAWITCA	AAA85872.1	UL133Toledo
KELCIAHLPTLRDIR	CAA35323	UL121
CHKICYAVDMSDESY	CAA35333	UL97
GGMDEPPSGWERYDG	CAA35354	UL80A
LVTLIAEHLADGALP	CAA35360	UL86(MCP=majorcapsidprotein)
DGTLCLFLEPEEREL	CAA35426	UL27
MEHRHLCPHHHERAV	CAA35291	US24
FIVETGLCSLAELSH	CAA35390	UL75(gH)
GPGFMRYQLIVLIGQ	CAA35395	UL36
VGQDKLVRLARDLRG	CAA35428	UL29
LASYACYTVFGLGSI	CAA35328	UL126
RDMRRVTFSNIATHY	CAA35269	TRS1part
PRPATRSSDPSASNP	CAA35415	UL15
NRNYTLHVNHSCDYS	CAA35318	UL116
KKACALTRRSRHRLR	CAA35396	UL37
RAWSLGLDTMARFII	CAA35423	UL24
ETHRLVAFLERADSV	CAA35414	UL55(gB)
GNGEVFRVPEQPSRY	CAA35394	UL35
LGRYSLGDQIWSPTP	AAA85880.1	UL141Toledo
TLPLHHSNTQPHVQT	CAA35449	TRL1
ECQYSADVLRDWVRN	CAA35290	US23
EVPAWPASSRTCLLC	CAA35369	UL95

SRTTSNSSRSTSPVA	CAA35424	UL25
RYMYLFSVSCAGITG	CAA35419	UL20
LLLFQRPQWAHGLDI	CAA35445	UL13
FTRNDEFLYCHTRYE	CAA35290	US23
NSSVTCWGSNGTFGA	CAA35322	UL120
AAFESCCYDITEAES	CAA35438	UL5
DSVTFEFVPNTKKQK	CAA35403	UL44(pp50)
NTVSVLAFALPLDRV	CAA35356	UL82(pp71)
IVLFFARRAFNKKYH	CAA35460	TRL12
LERRSHEELVLCPPE	CAA35362	UL88
AGAPGTYVNSSVTCW	CAA35322	UL120
TFDGVRSPPDVVRLY	CAA35374	UL59
RRCSLGRYALWIYNI	CAA35380	UL65
CSPDEIMAYAQKIFK	CAA35325	UL123(pp72=MIprotein=IE1)
LELARQHSSPAFQPM	CAA35394	UL35
SLLTPDEQARVFCLS	CAA35316	UL114
LAFEARFADDEQLPL	CAA35367	UL93
QKHKPPDKPPRLCKT	CAA35311	IRS1
VEDFSLENLRRVLDA	CAA35372	UL57
RPKKCQTHAPHHVHH	CAA35311	IRS1
TEHVYECDLSCNITT	CAA35434	UL1
MTTTTHSTAAIMSLL	CAA35397	UL38
SPFLASGMMISAPLK	CAA35322	UL120
DVSWDPRIRPDYPQT	CAA35293	US26
VASVFEHLTVECVND	CAA35365	UL91
HVATHVLRGLLSLTE	CAA35361	UL87
GTDPRHVLEDLTVYE	CAA35373	UL58
HIPVEHTGVSSVTLL	CAA35424	UL25
RGAKSTALFASCYNT	CAA35363	UL89
LCVYYSDEEEDQEED	CAA35293	US26
SDYIFLQDMCPRFLK	CAA35396	UL37
RRAFCTLADAIKFLN	CAA35333	UL97
AVSPELPSRDGIRWQ	CAA35445	UL13
FISLNSQVRKTANMF	CAA35363	UL89
MLRGDSAAKIQERYA	CAA35363	UL89
IISFLLKHMIGISIG	CAA35363	UL89
VGGGPPLTESYVLAG	CAA35421	UL22
DVFGMRQIVTQAGEP	CAA35407	UL48(pp212)
YGTVYVYDWETDGLY	CAA35289	US22
DESSASSSGEAPEQQ	CAA35264	US32
RELLCLHVFKLRRGC	CAA35426	UL27
RYNALTVRSRDSLLL	CAA35445	UL13
RERMKHDPENVIYFR	CAA35295	UL132
KKLLLLDVPRLENYF	CAA35362	UL88

RTRVSLGHRVAFGCS	CAA35439	UL6
SHEELVLCPPEMEER	CAA35362	UL88
TGQTMSFLRLLHGFL	CAA35341	UL104
DFLELVQRIPDYRSV	CAA35426	UL27
YNDPVAVFDFASLYP	CAA35413	UL54
QFCRGPLLYVHRRCC	CAA35361	UL87
WFEQRRLAEKERWHL	CAA35290	US23
DRGVLYYLAHSLDDF	CAA35293	US26
SLGDQIWSPTPWRLR	AAA85880.1	UL141Toledo
TLYQDGILRFNVTCD	CAA35358	UL84
GTQWSTNFFFSQCEH	CAA35261	US29
VTLQLHGNQTVQPSF	AAA85887.1	UL148Toledo
KLRYDKQLVGVTERE	CAA35353	UL80
DEVDRWIRHAAGVER	CAA35360	UL86(MCP=majorcapsidprotein)
KTAWLRHFNISTHGK	AAA85896.1	UL154Towne
MAVYEILSVREEIPA	CAA35372	UL57
YRATCEDRTRTLVTR	CAA35459	TRL11
MDEDELQQLSRLIEK	CAA35384	UL69
FVSVLLISFVALQTP	CAA35432*	UL33
ILAPEAGLEVCAQLL	CAA35371	UL56
GVERSLNVSSMSGQD	CAA35372	UL57
RLDVNPDLMYATDPH	CAA35367	UL93
PRSPGPSSLAPGRCF	CAA35310	J1I
QSAWRRWRSHVDDEE	CAA35439	UL6
NENVTLNHLAVVRAM	CAA35411	UL52
VPCFIFKNTGCAVSL	CAA35409	UL50
PISTVTETRWFLLNF	CAA35342	UL106
PDLTAADRDGLRAQW	CAA35353	UL80
EAEIAARLVSTYRDR	CAA35407	UL48(pp212)
CGYKYDWSNVVTPKA	CAA35430	UL31
TLKNSHTLRIYRRFY	CAA35358	UL84
TLIRVLFYHTPDQNH	CAA35363	UL89
NNSTPLLGNFMYLTS	CAA35403	UL44(pp50)
QFRRTTYDRWDGRRW	CAA35261	US29
SPPPRVILSVRDKIC	CAA35386	UL70
PYPADLKVPTAFPQD	CAA35384	UL69
LNLTTENSGKYYFKR	CAA35437	UL4(gp48)
PRIQVHYKLLFGLNV	CAA35361	UL87
LITLVIWTVVWLKLL	CAA35435	UL2
DVEFVPGESLKWNVR	CAA35313	US2
PHSATSPHGLGLAGY	CAA35386	UL70
MAQRNGMSPRPPPLG	CAA35311	IRS1
LHTALMRLGFTYFAS	CAA35404	UL45
YFTTLKQYLRNLAFA	CAA35417	UL18

LDGHVYPLAAELSHF	CAA35311	IRS1
KTGLVYEALYPVARS	CAA35366	UL92
LFLMNTFLLHQEGFR	CAA35334	UL98
CAYLIRYREFFKDSV	AAA85872.1	UL133Toledo
HRFFSLRTRQTLVIG	CAA35387	UL72
AGVPAGCGGGVCRRC	CAA35310	J1I
ALVFSANSVLYQHRL	CAA35372	UL57
CVPELRVDYTSSAYM	CAA35313	US2
GTIRNTSSTAGGRGR	CAA35415	UL15
VLPSFFFSGSARFLF	CAA35422	UL23
RLFHKRGVIQHLPGY	CAA35392	UL77
KWMSTLSCGVSVSVN	CAA35404	UL45
GRIDGTHLAGFFGTS	CAA35386	UL70
YLLEQGFPVYEVRVD	CAA35413	UL54
FTIWQARVVRGDFAE	CAA35290	US23
LAQFRGTMDDDEAAL	CAA35430	UL31
KVRNIMKDKNTPFCT	P19893	UL122(IE2)
ETDSRTLKQFLQEEC	CAA35368	UL94
FSPARSGRRGRRGLY	CAA35430	UL31
TKMPVSHVIRSSLIL	CAA35281	US14
LWYNLTVKPKPLETT	CAA35440	UL7
ALPPQLLFHYRNLVA	CAA35360	UL86(MCP=majorcapsidprotein)
DDDDELSLPRNVMTH	CAA35395	UL36
GSFRLFQLIMRHGPC	CAA35404	UL45
PVYDLQRYTAESLRL	CAA35384	UL69
LCYGFGNNFFVRTGH	CAA35336	UL100(gM)
ERLLFEDRRLMAYYA	CAA35278	US11
KTPTCQHPKKTPDPM	CAA35395	UL36
RQLKGALVDFDFLRL	CAA35391	UL76
IFTEHVLGFELVPPS	P16832	UL115(gL)
YDDESWRPLSTVDDH	CAA35311	IRS1
SNRATSQRASLNAVH	AAA85891.1	UL150Toledo
NSLTISSFTSTNLTH	CAA35442	UL9
RSRFDFRALVSPDRL	CAA35427	UL28
ILMGAFCIVLRHCCF	CAA35388	UL73(gN)
VALIPVVIILIGTLV	CAA35434	UL1
VLVDLGLPQSAWRRW	CAA35439	UL6
VALIALYMGSHRIPK	CAA35433	TRL14
VACPPREPPHRALFR	CAA35445	UL13
RELLFHERLKSALDK	CAA35363	UL89
GDVQRLIRLFKGEAA	CAA35426	UL27
FQRMVMTKPYFVFLA	CAA35408	UL49
PPFVTHLPRNMEGVQ	CAA35360	UL86(MCP=majorcapsidprotein)
LSMSLCVPPYFIHPT	CAA35268	US36

SWSNPACDDGLFLYR	CAA35342	UL106
HVGHHLALRGSSLHL	CAA35282	US15
REELGYVRFETGGDV	CAA74075	UL43rev
SLVSGLLFSAMYYFY	CAA35340	UL105
LWGSRTRVSLGHRVA	CAA35439	UL6
WEFPCLRLHDGRLFL	CAA35400	UL41
DADGGGVEESNRRGG	CAA35413	UL54
CLDRDRGVLYYLAHS	CAA35293	US26
WLIRKDRFIVRPDWC	CAA35293	US26
YHRRRKHLPRRRAPC	CAA35416	UL17
HVLKAVFSRGDTPVL	CAA35357	UL83(pp65)
RDFSNDDSVLRAAEM	CAA35393	UL34
LEAAACLLAAYGHAL	CAA35392	UL77
LSVISYCYYRISRIV	P09704	US28
TDYLLRRLRLYPPER	CAA35408	UL49
LRCENGTTKIIERLY	CAA35448	UL16
RYSQRTTQCVAAHLL	CAA35370	UL96
LNDIERFLCKMNYVY	CAA35371	UL56
LTVRVRHALCWHRVE	CAA35358	UL84
AITTDFRCAPPTKRR	CAA35373	UL58
IEVRLSRRIPDCVLV	CAA35391	UL76
YHHITFCSVPATDGR	CAA35343	UL107
SASWLSSFTPAAALP	CAA35415	UL15
PMPAFALTSLVDPVL	CAA35424	UL25
AAYAQMALIQPASQK	CAA35406	UL47
FLINLTSHYSGIYYF	CAA35442	UL9
VASALFVYFRYERPV	CAA35283	US16
QQQQQRYDELRDAIH	CAA35354	UL80A
HAMCKCRITEPITML	CAA35458	TRL10
EDEEGGEKGGDDPGR	CAA35411	UL52
RFLFGVDLRLPVLHP	CAA35408	UL49
SVGASRDYVHVRLLS	AAA85887.1	UL148Toledo
ARTLGLDLTTVMTER	CAA35311	IRS1
EILSVREEIPASDDV	CAA35372	UL57
VGYRLLKEAIKIWEF	CAA35400	UL41
LVSETLFGQRCVRVG	CAA35408	UL49
MSMTASSSTPRPTPK	CAA35340	UL105
TTEPRYHVQKFLCTD	CAA35289	US22
PSPSFYQKTHFSSCK	CAA35373	UL58
QESAPGVLLVWGDER	CAA35338	UL102
ERKEFLVRQYVLVDT	CAA35395	UL36
TSSFPRGITQTQTRV	CAA35310	J1I
FPSWARRWTQRHDSE	CAA35312	US1
ERLSGQVRDDVLSLW	CAA35392	UL77

AACLTYLRETFEGTI	CAA35360	UL86(MCP=majorcapsidprotein)
FDIATTLEHLSGVPV	CAA35406	UL47
IRRSCLVHSACATAH	CAA35426	UL27
CITPTQWPAMQLNKL	CAA35407	UL48(pp212)
AVWRAAFLANSTGRA	CAA35311	IRS1
DFRCAPPTKRRTSYT	CAA35373	UL58
LKIVRLICVLLFHRG	CAA35352	UL79
PQLLPRLADDVSREI	CAA35392	UL77
GYVRFETGGDVGREF	CAA74075	UL43rev
DFVILVDRACEFFYF	CAA35293	US26
GGGEGGGLRWFPFRQ	CAA35375	UL60
YVLIRAVRDEIYAVL	CAA35367	UL93
SVDRSRVPETGGRWL	CAA35429	UL30
IEDLSANFRRQLAPY	CAA35318	UL116
PPLTESYVLAGGGIT	CAA35421	UL22
GPCLIRHSPRCDLLL	CAA35404	UL45
YTGDVEAFYLIIHPR	CAA35459	TRL11
YSRRCFKENYFTGHH	CAA35460	TRL12
YPGWGRRYEPAPSLH	CAA35353	UL80
VRRRRFKVCDVGRRH	CAA35395	UL36
YLQLSFGEIVAAAYD	CAA35341	UL104
KKTQSTTTPYFSYTT	CAA35389	UL74(gO)
SYQIPFLAKQLVLFL	CAA35361	UL87
TSRLTRVPIFSLPTP	CAA35422	UL23
NNRRQHPPPPHTFTN	CAA35268	US36
RRESSCAVLVHHVGR	AAA85880.1	UL141Toledo
FWVLLSCPRLLEYSS	AAA85880.1	UL141Toledo
GRSLTYPGSIQTDKK	CAA35379	UL64
CTSYGFFPGEINITF	CAA35417	UL18
YALALFAAARQEEAG	CAA35390	UL75(gH)
IEKANIPLGIHAVWA	CAA35433	TRL14
AYAYSLVSGLLFSAM	CAA35340	UL105
LALLYNNPDQLRALL	P16832	UL115(gL)
QWHSDLTTELLWHAH	CAA35411	UL52
TPANTTTNSSTEGNW	CAA35419	UL20
YVATALYYVHFPFFS	CAA35282	US15
IAHLPTLRDIRTCRV	CAA35323	UL121
LHMVLPRRLHLEPAF	CAA35413	UL54
IVVCIMGWWKLLCSK	CAA35440	UL7
LLCTSAFLGYSAVFL	CAA35339	UL103
NDGRGCTSEGVIFGH	CAA35413	UL54
AQYGFRLLRGIFLIT	CAA35435	UL2
LTEWNRELPSLFCDC	CAA35361	UL87
VSTNTALISTPWLTN	CAA35460	TRL12

LRKQLLYGVCKTLFD	CAA35341	UL104
YHIGVKDSEGRWLPV	CAA35368	UL94
NRTRSAQVKTIYRVF	CAA35340	UL105
AEHLRTDLDTEPLLL	CAA35459	TRL11
VVGLPFFLEYAKHHP	CAA35259	US27
NNWDGWDAFTIWQAR	CAA35290	US23
YRRLSVTFADTDTVW	CAA35361	UL87
DPFFLKYVKPPSLAL	CAA35340	UL105
IPDCVLVFLPPDSGS	CAA35391	UL76
DGNDSHLICFYKRGE	CAA35440	UL7
LPNCSTITTTAGQDA	CAA35439	UL6
QSKKKKKPSKHHHHQ	CAA35335	UL99(pp28)
YRHTWDRHDEGAAQG	CAA35357	UL83(pp65)
RMLSGDVQRLIRLFK	CAA35426	UL27
EVVVSSPRTHYLMLL	CAA35390	UL75(gH)
AVAVTDEQCCLLLQS	CAA35338	UL102
IRCGKVNDKAQYLLG	CAA35330	UL128(viralentry)
VVALVNFLRHLTQKP	CAA35431	UL32(pp150)
PNCCQVSVDRSRVPE	CAA35429	UL30
NVARCVFARSDEQKL	CAA35404	UL45
PQQQQMLALIDDELD	CAA35384	UL69
PGTLMLVMVYTTLTT	CAA35279	US12
VPRRPRYTKLPKYDP	AAA85895.1	UL153Towne
HESDDDEWEDLGFDL	CAA35290	US23
HTCYQTAFVRVRTRL	CAA35372	UL57
ADIDTGMSPWATRGI	AAA85880.1	UL141Toledo
KAKREVNTISVRYLY	CAA35405	UL46
RCHGPLSTPKNACLA	CAA35265	US34
AAWLSTRRELVGGFS	CAA35420	UL21
RWAPSSWWRARSGPS	CAA35409	UL50
DEELRHRGTPESPRL	CAA35424	UL25
FSRVAVSLRRRALQV	CAA35384	UL69
KVSRKKREDALLKQM	CAA35366	UL92
FENRRFSGHNGIYDR	CAA35423	UL24
RCGLEIDRPMPTVPK	CAA35269	TRS1part
AFVGALPSSGYHFGF	CAA35406	UL47
RECTSPLGAAHEDVA	CAA35411	UL52
GNCEFPTCFTLSLIS	CAA35443	UL10
LLKHMIGISIGYVAH	CAA35363	UL89
PGGDSDYHGLSGVDG	CAA35411	UL52
CWLRRTAIVMRVYGL	CAA35281	US14
LQLHHGLMWLRRFAV	CAA35280	US13
WVWWLFGYFVSSVGS	AAA85881.1	UL142Toledo
PGRVRRDSAWDVRPL	CAA35431	UL32(pp150)

IFMIVCVLWCVWICL	CAA35331	UL129(viralentry)
AVSGASGGFLLHRKT	CAA35362	UL88
SHTNNVNVGWFKAAT	CAA35361	UL87
DDVLFFVDGCEALAA	CAA35372	UL57
LDVVVSEIRSAHFRV	CAA35314	US3
DDAKYLCAVGSKTAA	CAA35368	UL94
KMSHIYVAMSRVTDP	CAA35340	UL105
QTYCEDLEGRVSEAE	CAA35392	UL77
MISSVSTNDVRRFLL	CAA35284	US17
SPEWVKSFDFREHFV	CAA35290	US23
PDMGRCLCYVPCGPM	CAA35368	UL94
PATWFHHSRLCLTES	CAA35282	US15
HRVRVEDLVLSSVLS	CAA35413	UL54
CSLPGSRTSRHAGWD	CAA35377	UL62
EVCLSVYPSVYLSPY	AAA85873.1	UL134Toledo
RVLRRLLEDAAVTMR	CAA35392	UL77
FLCPTSPSWFISVFG	CAA35386	UL70
GALVDFDFLRLPRGG	CAA35391	UL76
FTYPIYAVYGTRLNA	CAA35320	UL118
HPFWQQCVRRRRTSR	AAA85876.1	UL137Toledo
QRIVNAYRTEAEVSE	CAA35290	US23
SCDLAFGSRSQTRYL	CAA35447	UL14
VAPQHVEYLKFVLQD	CAA35424	UL25
VYHRNQWCHQRQPRS	CAA35408	UL49
MNKFSNTRIGFTCAV	CAA35399	UL40
ERDWRRVIHDSHGLW	CAA35264	US32
CRGLPVKYRTHRAAV	CAA35426	UL27
RLTKKNHLGIKKSSI	CAA35455	TRL7
NQWCHQRQPRSPQLR	CAA35408	UL49
RDYMTYMNLAELYVF	CAA35369	UL95
GSHRIPKKPHYTKLP	CAA35433	TRL14
ICANVEDYLQDARRR	CAA35269	TRS1part
LPSLREDYAQLSDVI	CAA35424	UL25
QRLRIRLPHRYQRLR	CAA35448	UL16
RALPSYCRLDFFRPS	CAA35377	UL62
DVSWYHSMSFSRRSS	P09704	US28
MINKRVKRKKLQTFG	CAA35453	TRL4
IAVEYVLIRAVRDEI	CAA35367	UL93
NQWLLPAGVASCKFL	CAA35432*	UL33
VSEEYGTHAHKKLRR	CAA35338	UL102
AHRAKDAAHSAWILI	CAA35443	UL10
GIPGEKLRRTVVTTT	AAA85880.1	UL141Toledo
PALSSPETGDDSNDD	AAA85875.1	UL136Toledo
TPPPADFQQPVFKTL	CAA35407	UL48(pp212)

FAPRTGKFTLADLLG	CAA35392	UL77
VPVTPNVDLLAELMA	CAA35406	UL47
HLSGVPVTPNVDLLA	CAA35406	UL47
GGGGGGSGRMSLPLD	CAA35315	UL113
SSAAKIERIVDKVKS	CAA35340	UL105
RPAPPALSSPETGDD	AAA85875.1	UL136Toledo
LCSVERMVELSAQSP	CAA35369	UL95
ENIPGVSIEAGPLPD	CAA35356	UL82(pp71)
PSSSQALSVPSLSSE	CAA35380	UL65
LPGPCIASTPKKHRG	CAA35357	UL83(pp65)
TTLEHLSGVPVTPNV	CAA35406	UL47
VVAKRLPRPDTPRTP	CAA35335	UL99(pp28)
SYGSDVEDPRDDLAE	CAA35394	UL35
EKLRRTVVTTTPARR	AAA85880.1	UL141Toledo
PLHVVSTNGCGPSSS	CAA35412	UL53
NHLILQISEKMSRGQ	CAA35352	UL79
GVCVMRRLINHIVNH	CAA35443	UL10
KPPSLALLSFEETVH	CAA35340	UL105
RRRRQSINDEMKERT	CAA74075	UL43rev
SQSPPRSCLVAPQSS	AAA85890.1	UL149Toledo
SRFVESAQGKSLHVC	CAA35340	UL105
RSVRLPYMYKMDQDD	CAA35386	UL70
GKKTIITEYRITGTR	CAA35294	UL131
TGKFTLADLLGSDAV	CAA35392	UL77
GFQQVSTGPECRNET	CAA35332	UL130(viralentry)
LGGGGGGGGKKHDRG	CAA35403	UL44(pp50)
MIEGASRQTGLTPKR	CAA35394	UL35
HTMDDLVMVFHQLDY	CAA35397	UL38
ASDYTPTFDDLENIT	CAA35318	UL116
PMALYRQVLRDFKEL	CAA35291	US24
SAFFVPRDRRGRSFC	CAA35391	UL76
DKPKGRPKDKPPCEP	AAA85872.1	UL133Toledo
ALYLQPVPLGHKLFL	CAA35284	US17
ELHTLLDPISHPESS	CAA35386	UL70
IVGNMSRFVFDPKAD	CAA35313	US2
AGGEEEGYGGRHGRV	CAA35392	UL77
ASRQTGLTPKRFMEL	CAA35394	UL35
GRAKDKPKGRPKDKP	AAA85872.1	UL133Toledo
GDQKPGAEHMRDVSY	CAA35406	UL47
ATVQECTVLTAPNCG	CAA35261	US29
RLCNLRCFENSVEGG	CAA35423	UL24
TPPHINDTCNMTGPL	CAA35432*	UL33
TFREDKAPKPSKQSK	CAA35335	UL99(pp28)
HAALDLAPAAAFGLL	CAA35282	US15

NAVLALRIIRLLRAS	CAA35426	UL27
FPRKLGINSHNTDTT	CAA35443	UL10
RCPRKKEERTTRSPV	CAA35415	UL15
PVKGRGSRVGVPSLK	CAA35431	UL32(pp150)
SGRFFHRRRSRFDFR	CAA35427	UL28
TVNSLARAVVFTEDH	CAA35407	UL48(pp212)
LSPVSSSPSRSPSSS	CAA35456	TRL8
KRRTSYTPQKPARLR	CAA35373	UL58
AHPKILKKCGEKRLH	CAA35431	UL32(pp150)
APPTKRRTSYTPQKP	CAA35373	UL58
QALSVPSLSSEKKTA	CAA35380	UL65
DLSIPHVWMPPQTTP	CAA35390	UL75(gH)
MRYQLIVLIGQRGGI	CAA35395	UL36
GGGGGDSGGMMGRGG	CAA35372	UL57
HLLRLDESPHSATSP	CAA35386	UL70
REAVMSPTMVTIPPP	CAA35385	UL71
VDLEAHPKILKKCGE	CAA35431	UL32(pp150)
TETEAAGGDAPCAIA	CAA35311	IRS1
EGQDHGDAVHHSLDV	CAA35261	US29
AGAFATSETHFGNYV	CAA35360	UL86(MCP=majorcapsidprotein)
GTGLGDGGCAGRRWX	AAA85892.1	UL151Toledo
VTHQGAEEAIVYSNY	CAA35341	UL104
SLYYNETMFVENKTA	CAA35389	UL74(gO)
SPPPYRPPYCLVSSP	CAA74074	UL42rev
QNEMCELRIQRALAP	CAA35384	UL69
AVQSTTTVMTPTVVT	CAA35439	UL6
CIGLTMQSMYENYIV	CAA35325	UL123(pp72=MIprotein=IE1)
AIANGTTHKPSTASS	AAA85895.1	UL153Towne
FRAKRWELICSRVLT	CAA35280	US13
WYRGYEFPAPTPQAT	CAA35369	UL95
ERMVELSAQSPAADF	CAA35369	UL95
ALWAPVAGSMPELSL	CAA35278	US11
AFLTLLMCQPSPQAF	CAA35286	US19
FLDTLALLYNNPDQL	P16832	UL115(gL)
YNCTDCTQCNVTQVM	AAA85883.1	UL144Toledo
DYEPVPRKFRRERSP	CAA35384	UL69
LEDQAVIRSERSKVY	CAA35361	UL87
LERQQHQFLRRTYGP	CAA35370	UL96
MYLQANRDDNFFAER	CAA35436	UL3
QFWQKVCSNALPKNV	CAA35372	UL57
VLFGRLHPHHVQEVK	CAA35411	UL52
KVQHVEAVLRQVYTP	CAA35369	UL95
TGVVYRDISSTIATE	CAA35272	US5
IDYGLHRVFTQLELR	CAA35361	UL87

SLVRHVGAQNGMTSE	CAA35377	UL62
LNVNTHFAVQYTEED	CAA35406	UL47
PVADYVLLQPSEDVE	CAA35385	UL71
ASISGCDLLREVQRN	CAA35284	US17
QLVLADLLRECTSPL	CAA35411	UL52
VEDAKIFGAHMVPKQ	CAA35332	UL130(viralentry)
EANGNHPEQICRSPP	CAA35424	UL25
IHELKRDLFAARQSS	CAA35353	UL80
GGLAAASSSSSPHGL	CAA35408	UL49
DALLKQMRSEYGNAP	CAA35366	UL92
CDKFLLPVGTVSRCE	CAA74075	UL43rev
NVRLLVIGPRTGGRF	CAA35415	UL15
YEANPELRLPFKKRR	CAA35428	UL29
FSQVTSSMTCDGITP	CAA35368	UL94
SVADNLGFEPSVVAP	CAA35424	UL25
VAAWLVRGNFSDTAP	CAA35276	US9
EDVLMDRVRKRYLRQ	CAA35392	UL77
LERLEREWQEEAGKL	CAA35407	UL48(pp212)
GPSRPQSGPWLPARF	CAA35409	UL50
KEEHVAYVDRFVRPP	CAA35340	UL105
MIVPPTPDIGFTHTP	CAA35387	UL72
LIDDELDAMDEDELQ	CAA35384	UL69
QAGMRLCEKGKKRII	AAA85880.1	UL141Toledo
DYATRLQDLRVTFHR	CAA35360	UL86(MCP=majorcapsidprotein)
IRNRFIPANIPNKIQ	CAA35415	UL15
LDLARDEARTVSYSP	CAA35369	UL95
PATTVSTKPSKTTTQ	CAA35461	TRL13
LLFLDEIRNFSLRSP	CAA35389	UL74(gO)
KISMCAPDFNMEFSS	CAA35403	UL44(pp50)
DVASIGDIASYRLSP	CAA35329	UL127
LSTFTMSTVGFDRVP	CAA35404	UL45
RQFPPPPRFPPRCSD	CAA35415	UL15
VPQHIADYNDGGDMG	CAA35296	IRL14
RRRQWMREAAQAAAQ	CAA35333	UL97
RAMVMGEDTVPYNKP	CAA35411	UL52
TTVEAVDLCAENLSD	CAA35394	UL35
SSFYSQIARSLGVLP	CAA35399	UL40
GSVTAGRALSYHVLE	CAA35361	UL87
HRNEGRCLSLGPPKG	CAA35291	US24
MGMPHYLMYSHTNNE	CAA35259	US27
ERSKTPDKRSAETTQ	CAA35310	J1I
SPATSPLSMLSSASP	CAA35431	UL32(pp150)
TGKPTYNLLTYPVKG	CAA35274	US7
TRDLGTIIPTHASMG	CAA35386	UL70

SHAAVDRPRRTRRGD	CAA35457	TRL9
FHTYDQTDAVLFFDS	CAA35413	UL54
DATDSRLLMMSVYAL	CAA35390	UL75(gH)
MSRFVFDPKADYGGV	CAA35313	US2
EFCVLIAALYPEYIY	CAA35407	UL48(pp212)
VDRPRRTRRGDASPR	CAA35457	TRL9
VFVDMWDVAAIRVIN	CAA35404	UL45
GPEAMDSQAPYPSED	CAA35289	US22
VQHTYFTGSEVENVS	CAA35357	UL83(pp65)
RWKDNKQYGQVFMTD	CAA35441	UL8
DVNVSRFVESAQGKS	CAA35340	UL105
PQIYARSLAADYLCC	CAA35289	US22
DAKPGLNERDGFRQR	AAA85891.1	UL150Toledo
TLMPYVLFRRDTDTE	CAA35406	UL47
LVKKPGQMSAWLRDD	CAA35384	UL69
TRNNTPPHINDTCNM	CAA35432*	UL33
ANGMPPLTPPHVYMN	CAA35395	UL36
GTYSTLDRALLEKMQ	CAA35407	UL48(pp212)
VAILGIIFLAVVFTV	CAA74074	UL42rev
SLGRALRRDDEDWKP	CAA35269	TRS1part
PPPPSPAYYRRRDSP	CAA35353	UL80
SRPSVLCCFQENKSP	CAA35356	UL82(pp71)
AIISIIYFLLIEAVF	CAA35336	UL100(gM)
VSCPTVMRFDQRLLE	CAA35385	UL71
KRSGASEGEDGMVSP	CAA35456	TRL8
AVVWGNARLDALMSA	CAA35404	UL45
CNSENEDDTTVEGTS	CAA35289	US22
PVEEKKHPVPYFKQW	CAA35320	UL118
VPMVATVQGQNLKYQ	CAA35357	UL83(pp65)
RRNDVDFWLLRFQPG	CAA35338	UL102
QPVRDRNRERNPGSP	CAA35447	UL14
PPPSPGPMHMVVCMP	CAA35419	UL20
PIQSEAEGENKQFTE	CAA35429	UL30
GPEWMSGVHLDGCAP	CAA35310	J1I
QLLEKESRGQSRNSV	CAA35334	UL98
GDAGSVAAMFQMSPP	CAA35413	UL54
RLNFCLIDTCLELCP	CAA35378	UL63
DSRDRHHHDPFIDTP	CAA35330	UL128(viralentry)
SSTSPVYDLQRYTAE	CAA35384	UL69
FIENLRFRSRRAFWQ	CAA35338	UL102
LNLMTPSPSHGGSPP	CAA35384	UL69
PECGLPCLQFWQKVC	CAA35372	UL57
SLPHLPVYDVRSPRP	CAA35445	UL13
RRRPERSKTPDKRSA	CAA35310	J1I

ENLEGVRRNMFCVKP	CAA35431	UL32(pp150)
LARCPTKPVTSMWNS	CAA35294	UL131
LVKSEDTHLTCKCSP	CAA35458	TRL10
SDIDGDQKPGAEHMR	CAA35406	UL47
DLERLFAERRYLTFL	CAA35276	US9
TTCTPLIMDLPSLSV	AAA85891.1	UL150Toledo
REAIVRLEKTPTCQH	CAA35395	UL36
LGESVAGNSICFGVP	CAA35372	UL57
RLPRSRFQRFWETPT	CAA35387	UL72
TSGLLGASMDLCFGV	CAA35334	UL98
LKRFKLLMEVYHGLV	CAA35285	US18
YLDYRDERDTEEDED	CAA35386	UL70
SRMPKNHFWKNGDIS	CAA35329	UL127
VNNRAYQELCELADP	CAA35340	UL105
GGQKTASNDTSTKIP	CAA35432*	UL33
CELTRNMHTTHSITA	CAA35390	UL75(gH)
KATQQLPYLSAERTV	CAA35407	UL48(pp212)
NSVPVDAAGAPFDDD	CAA35386	UL70
ASVMTRRNAVDLDRP	CAA35445	UL13
TKKPKGKEDESLMKG	CAA35269	TRS1part
FVVRAVPVESQQRLD	CAA74075	UL43rev
ATDEFHQALRRLFAP	CAA35408	UL49
DDVWTSGSDSDEELV	CAA35357	UL83(pp65)
KDFLRNGFRHRDHFH	CAA35395	UL36
LWMDWADVRSCIIKA	CAA35367	UL93
SGGHAGSNQQQQQRY	CAA35354	UL80A
VSSLFVAGHGETDFY	AAA85887.1	UL148Toledo
LTSKGGSGGGGGGGG	CAA35403	UL44(pp50)
AYCPFDEQSLLDLTV	CAA35384	UL69
IYGLEHFFLRDLSES	CAA35363	UL89
TATAPATTVSTKPSK	CAA35461	TRL13
SVLDQRLWWEIQYSS	CAA35323	UL121
GETLMELKDNLTLWT	CAA35417	UL18
KQHKKKMMWFVVLTV	CAA35381	UL66
VMYPGAVAAPPSASP	CAA35354	UL80A
ADYAACAQARQHLYD	CAA35449	TRL1
EDVQRVLARAPSHRV	CAA35407	UL48(pp212)
DLDEEDTSIYLSPPP	CAA35335	UL99(pp28)
IHHPKLQPGVGLWID	AAA85887.1	UL148Toledo
ANKRYNTMTISSVLL	CAA35417	UL18
LSPSEPAEAASMSHP	CAA35353	UL80
LSVPTLIMVRVKNRP	CAA35372	UL57
PPLPPRPPHLIEFPP	CAA35419	UL20
DPMIMFDEDDDDELS	CAA35395	UL36

NGMPPLTPPHVYMNN	CAA35395	UL36
VCMFITYTLGNEHPS	P09724	US20
KTSLTIYNVTTEHAG	AAA85895.1	UL153Towne
DEASSPLPSPLKVPY	CAA35387	UL72
MCRRPDCGFSFSPGP	P16832	UL115(gL)
ESSLDTGIYHHGRSV	CAA35386	UL70
FRRGFWTLTPGKPDK	CAA35356	UL82(pp71)
FWTLTPGKPDKIKRP	CAA35356	UL82(pp71)
VTADYQEHDLLDVAP	CAA35372	UL57
ESKMWVLPPPLPPRP	CAA35419	UL20
QMNGVGATDLRQLSP	CAA35311	IRS1
KHPAICANVEDYLQD	CAA35269	TRS1part
SGLRPQETTEYTCSF	CAA35320	UL118
QHERPSLYHDLCRSC	CAA35460	TRL12
HDDSDAITDAELMDH	CAA35340	UL105
DTLREMALKAFMEAN	CAA35424	UL25
VIGSYPEVHEPRVVT	CAA35338	UL102
AAMDHSEFLTSFRRE	CAA35319	UL117
PVSVHGFSTAKSEKG	CAA35377	UL62
ALWKVDYDRSVAVGP	CAA35323	UL121
CRTANSTAGYVDMNV	CAA74073	UL41alt
SAKRQNRISDDLIIA	CAA35363	UL89
EADSLEMLLDKFSTD	CAA35385	UL71
DRFRAEMLNNWDGWD	CAA35290	US23
PPLEDPPRGPEWMSG	CAA35310	J1I
ALFAPSGVGAASGVG	CAA35372	UL57
SSGSIILAEKSVNMR	CAA35387	UL72
LIGSKEELQHVWSNV	CAA35406	UL47
PEAAVASLETAVSTP	CAA35367	UL93
AEGKKKLLLKHLPVP	CAA35393	UL34
LYPPTSTYNSLTISS	CAA35442	UL9
HDTCAAYAQMALIQP	CAA35406	UL47
SRLERAVKRLQQRIP	CAA35293	US26
MVFVSGTALGTGFHR	AAA85892.1	UL151Toledo
IGTTTAPVEWKSPDR	CAA35437	UL4(gp48)
KCQELLMRLDRERSV	CAA35359	UL85
GSIFSWRDGNEALTT	CAA74075	UL43rev
NNDTRSNNTDTIFVS	CAA35419	UL20
RRPLRVPVVNYAWLE	CAA35404	UL45
VLLFHRGLETLLLRE	CAA35352	UL79
TAPYLRDTLPFWSTL	CAA35279	US12
TPRVTGGGAMAGAST	CAA35357	UL83(pp65)
KQRSRTLTFVSVLLI	CAA35432*	UL33
TIDDPFDECPDTHFA	CAA35395	UL36

FSSACVHGQDIVRES	CAA35403	UL44(pp50)
QLREMLRRDEQTRLR	CAA35407	UL48(pp212)
HDGNHDGNDHSSLSP	CAA35426	UL27
SPDHLFSKWLDKHND	AAA85885.1	UL146Toledo
SLLGCDPGASLRRLW	CAA35286	US19
VSSIFSGLLSSGSQK	CAA35431	UL32(pp150)
AAEDPMALYRQVLRD	CAA35291	US24
VPATVPAALSILSTM	CAA35390	UL75(gH)
MAWRSGLCETDSRTL	CAA35368	UL94
ALKTLCHPVLHEPAP	CAA35360	UL86(MCP=majorcapsidprotein)
ETPAAPPRPVCEIKP	CAA35315	UL113
TTTNSSTEGNWSVTN	CAA35419	UL20
GSSSSQRLVGEFMVR	CAA35424	UL25
TADACNEGVKAAWSL	P19893	UL122(IE2)
VAQKKARHMVEAIRT	CAA35424	UL25
EAVNMALVACEAVSP	CAA35369	UL95
FQYHLEGWFPLRVLN	CAA35396	UL37
PSQSEVDCASLMETL	CAA35418	UL19
KVCSNALPKNVPIGD	CAA35372	UL57
TFLCCDKFLLPVGTV	CAA74075	UL43rev
EDKREMWMACIKELH	CAA35325	UL123(pp72=MIprotein=IE1)
SRLTWRLTWLTCPAP	CAA35364	UL90
PMEIKLSRLSVPTLI	CAA35372	UL57
FMKNTHVLIRNETPY	CAA35389	UL74(gO)
DSVVMRCQTPDYEDM	CAA35384	UL69
KQEITDAMFEAGNVP	CAA35425	UL26
RQLLFNKLNNLDLGC	CAA35384	UL69
LVKKAQLNRHSYLKD	CAA35390	UL75(gH)
VALLAPERTVRRQIV	CAA35282	US15
RVLSAPPSLPLPVSE	CAA35397	UL38
CVARLQAQPSSRHIP	CAA35424	UL25
CRVCRGRVAGVPAGC	CAA35310	J1I
EEETITDSIAAACRP	CAA35349	UL111
IVAAALWKVDYDRSV	CAA35323	UL121
MCTSSGHRPRPPAPP	CAA35311	IRS1
DLHLTDTRHCTSCTH	CAA35323	UL121
GSHRHPSPMIAAAPP	CAA35384	UL69
NMFCVKPLDLNLDRH	CAA35431	UL32(pp150)
RRMTVPVPRRYPKGA	CAA35291	US24
ILKESTSELTGVCYA	CAA35404	UL45
GRGAEEVTWLLNDSD	CAA35312	US1
ALPVAAEDPMALYRQ	CAA35291	US24
TEMSTTMFTSSNGNV	AAA85896.1	UL154Towne
ATCALGLTLQSVRSL	CAA35430	UL31

NEERQQKLRLCGSGC	CAA35335	UL99(pp28)
RFVDILQKDTFIERT	CAA35340	UL105
LSHFTQLLAHPHHEY	CAA35390	UL75(gH)
VPLPPPPAPPPLPSP	CAA35453	TRL4
VNDGPLLFPHRKKKN	CAA35383	UL68
LLRERVSVSELEAVY	CAA35338	UL102
KMQVVFDPYGRQHGP	CAA35407	UL48(pp212)
PEFTEEEEEKEKLLT	CAA35419	UL20
YEPEVSMAYIYQYND	CAA35322	UL120
GSLVRAANCPRPQRN	CAA35310	J1I
TSVDTQHRSPSRCFM	CAA35374	UL59
EECYDQRFTTEGHQV	CAA35311	IRS1
IDSVTFVPRNLSNCS	CAA35318	UL116
WLLWQHDKHGIVLIP	CAA35444	UL11
TLALMETKLKGGAGA	CAA35360	UL86(MCP=majorcapsidprotein)
SRGGGGGGGSLSSLA	CAA35403	UL44(pp50)
VNRTSNTTSMNCHLN	CAA35396	UL37
VADVHDLRHSDRSCD	CAA35447	UL14
ESNEMYTKIKHILEE	CAA35371	UL56
LDHQDGWGDHCSTLK	CAA35384	UL69
AGEKNGGGSRAKRRR	CAA35369	UL95
ACCAGCVVTHRHSSG	CAA35338	UL102
EYDDTQGVINIMYMH	CAA35390	UL75(gH)
MELLDRAPLGQESEP	CAA35394	UL35
VCEKFIENLRFRSRR	CAA35338	UL102
WRRRKALSRIHRFWE	AAA85875.1	UL136Toledo
HRSTLIGSKEELQHV	CAA35406	UL47
FFWGAAGEGSVTGQA	CAA35376	UL61
YNQASKQQTAPPCLL	CAA35348	UL110
ERCDDRHGGSDDYVW	CAA35334	UL98
ALGFLFVLDVNVSRF	CAA35340	UL105
SLYTREPVMPLGEIE	CAA35428	UL29
SDDDDFFVYVEEIEP	CAA35293	US26
ELTTEFDYDEDATPC	P09704	US28
VPVPRRYPKGARTMH	CAA35291	US24
AYAVYHQGDMALMTL	CAA35399	UL40
TKRTHYDRKNAPMES	CAA35259	US27
GGLCSSMAVYDEETM	CAA35430	UL31
DDDEDPTYDELPSRP	CAA35311	IRS1
PQKAWNKFTQYRYNS	CAA35442	UL9
WKSWLPKFGKNLPPP	CAA35375	UL60
NFHEGKITTETYHLQ	CAA35394	UL35
RRLFGSSADEDDDDD	CAA35431	UL32(pp150)
CLSMSMVANLASELT	CAA35359	UL85

FRLGNAKMLELQMDL	CAA35407	UL48(pp212)
QLRGLIAALRRYAGK	CAA35408	UL49
AHAARSAAVGYYDEE	CAA35431	UL32(pp150)
VPEDEWQVFGTEAGG	CAA35338	UL102
LLLVDEAHFIKKEAF	CAA35363	UL89
SFDEAFLTDRLQQLI	CAA35407	UL48(pp212)
TMSTVGFDRVPQYDF	CAA35404	UL45
QLALSRLYEEEEETQ	CAA35407	UL48(pp212)
RRSQTHYLEEMALQV	AAA85887.1	UL148Toledo
LESDLAVWAALRGVP	CAA35426	UL27
LRNQQFMGYGTKNGL	AAA85891.1	UL150Toledo
DTFVETFCDFLELVQ	CAA35426	UL27
LPHERHRELCHVLIG	CAA35393	UL34
NQSTRDISYMGDSLT	CAA35336	UL100(gM)
QFPHVLGASPRFSSP	CAA35372	UL57
ALLHGKRDEGSFTSP	CAA35284	US17
TTTAELTTEFDYDED	P09704	US28
YFRKDGNLDTSFVNP	CAA35295	UL132
DPCCRPPGTSSFPRG	CAA35310	J1I
NRMVRFIINYVGKWH	CAA35259	US27
ACEAVSPYDRFRLIE	CAA35369	UL95
LSFLDWPDGSVTEGV	CAA74075	UL43rev
AAAAACEDLSELCES	CAA35340	UL105
ACCCVKQEIPYQDID	CAA35259	US27
KMLNDCKEKRLCDLP	CAA35315	UL113
VHVRGVNESAFGLTH	CAA35399	UL40
EIDRPMPTVPKGCRP	CAA35269	TRS1part
GYPVPRAEIRRGGGD	CAA35449	TRL1
TQWPAMQLNKLFWEN	CAA35407	UL48(pp212)
SVTFKRQVELEDLLP	CAA35415	UL15
PNCSHRLRECYHPAF	CAA35333	UL97
IRTRRLLVPWIRESK	CAA35419	UL20
VKIDVTILQRKIDEM	CAA35412	UL53
AAGEQQQPPSLVGTG	CAA35333	UL97
MRHSPDVPREAVMSP	CAA35385	UL71
VGSHPLGQMIVPPTP	CAA35387	UL72
MEPEENNVVPNVERR	CAA35290	US23
VSAFACPSVSDSLIP	CAA35385	UL71
AAGTTSPPAASGTET	CAA35311	IRS1
TSLGVSLYIPEGFFG	CAA35387	UL72
LSDVSEYRVEYSEAR	CAA35278	US11
FSSMCAGFVIGEEDR	CAA35348	UL110
PWATRGIAAFLGFWS	AAA85880.1	UL141Toledo
PPPFTTVEAVDLCAE	CAA35394	UL35

AWMTWLSSRATGATN	CAA35284	US17
PPPPPPDCSPPPYRP	CAA74074	UL42rev
HPGETWTLHGMCISI	CAA35448	UL16
AIYGEQMRTPLLTTQ	CAA35293	US26
TLATKHPAICANVED	CAA35269	TRS1part
MDRLQVSGEQYHHDE	CAA35314	US3
LSSQLVRPSRGLGAY	CAA35337	UL101
REPVMPLGEIEGAED	CAA35428	UL29
AKSRALGHWALLSIC	CAA35396	UL37
SAVLQTEALDAIMEE	CAA35407	UL48(pp212)
ETFCDFLELVQRIPD	CAA35426	UL27
APKHRPEARCRQQIP	CAA35310	J1I
HLQLRHALELQMMQD	CAA35394	UL35
FPGESTFCLTAVSEC	AAA85891.1	UL150Toledo
PVVRVFVDMWDVAAI	CAA35404	UL45
SSCSTTCTPLIMDLP	AAA85891.1	UL150Toledo
KALHHPIGGLFWVGR	AAA85894.1	UL152Towne
ILYQYADNDDYGLYV	CAA35341	UL104
LKSALDKLTFRPCSE	CAA35363	UL89
LCVRDYALRNADRVT	CAA35369	UL95
ILGVEARLRPYGASP	CAA35375	UL60
LELLDYLRQSGLTVT	CAA35367	UL93
QEIETDEDFKPEDVK	CAA35423	UL24
TLARVVPHLHCLINP	CAA35432*	UL33
GWSRIIVLLPLMCMA	CAA35323	UL121
RSQTRYLWTPDPSRL	CAA35447	UL14
EQRRRNQSRNAIPRP	CAA35445	UL13
WRFEEAVNMALVACE	CAA35369	UL95
LRAAKDLPPPGYRVG	CAA35400	UL41
SLFFFSRLHPKLKGT	CAA35336	UL100(gM)
ITTSQHATTTMHTIP	AAA85881.1	UL142Toledo
AHIPALGVTCACQEP	CAA35283	US16
AQTYTTGTLTRYSQR	CAA35386	UL70
TSNRLPNCSTITTTA	CAA35439	UL6
RHLTQKPDVDLEAHP	CAA35431	UL32(pp150)
FGRLHCQVLRLITNV	CAA35356	UL82(pp71)
LSSLRLEELKIVRLI	CAA35352	UL79
IMFLHALHLGGTSAV	CAA35407	UL48(pp212)
KGVWSDDDSSMGGSD	AAA85884.1	UL145Toledo
FSQFVPGTESLERFL	CAA35341	UL104
LFALDPYNEVVVSSP	CAA35390	UL75(gH)
QPGSAGMGGAKTPSD	CAA35431	UL32(pp150)
FLLGGHGRRVQLERP	CAA35449	TRL1
DPPRGPEWMSGVHLD	CAA35310	J1I

SHVEVQAYFKRLHEQ	CAA35340	UL105
HDVLERFAAAAKPLP	CAA35369	UL95
GLAWTRQQNQWKEPD	CAA35357	UL83(pp65)
HILNGFLPVEDLKQM	CAA35407	UL48(pp212)
LSAGHKKKETPTEGG	AAA85891.1	UL150Toledo
PVLNNVAPGERDLTR	CAA35424	UL25
HQMQQTLTTKMQDFL	CAA35407	UL48(pp212)
NPSLMNQGLCVYYSD	CAA35293	US26
GTLLPLGRPYGFYAR	CAA35311	IRS1
VTSWIEALRDADRDN	CAA35430	UL31
RDDVCDLQKRPPETF	CAA35384	UL69
LLSVRHLSLIAYMLL	AAA85886.1	UL147Toledo
RAPGGDEDDAPASDD	CAA35367	UL93
TAREMSGLNLVIRTP	CAA35289	US22
SPVRLRVNIRNRFIP	CAA35415	UL15
TRRYFDLSVLRELVT	CAA35404	UL45
GYCPLDGHVYPLAAE	CAA35311	IRS1
AELYHLPVLEAVRKA	CAA35341	UL104
ETAGTYTCVLGNETH	CAA35447	UL14
PLVHPDHRAELCRRS	CAA35428	UL29
KAVLGLNAACAVYDH	CAA35408	UL49
FTDVLNQSKPVTLFL	P09704	US28
YPRLTTYNLLFHPPP	CAA35394	UL35
ARDLLREEMEANKRD	CAA35367	UL93
PPSQTPEQSTPSRIR	CAA35431	UL32(pp150)
SAVPVMYPGAVAAPP	CAA35354	UL80A
GVRDPAPRGGRGRAR	CAA35376	UL61
TSDDDDQEKETENPQ	CAA35293	US26
ALRLPEQTVCHLSTF	CAA35361	UL87
SLRFSEPRVLIEAAL	CAA35372	UL57
VTAISYHSILADFNS	CAA35360	UL86(MCP=majorcapsidprotein)
FWYFSYRWIQRKRLE	CAA35396	UL37
ILKDKGTKCLNPNAQ	AAA85886.1	UL147Toledo
FLVAVTADYQEHDLL	CAA35372	UL57
NDGGDMGSRFDNLPG	CAA35296	IRL14
DIRTCRVDADLGLLY	CAA35323	UL121
VSSRLLPETSGGTVV	CAA35315	UL113
LRRMFGLPPPPSVAP	CAA35261	US29
ISFFLGILAYDSLMV	P09724	US20
PKTINNSTPLLGNFM	CAA35403	UL44(pp50)
VLSSVLSKDISLYRQ	CAA35413	UL54
YGADAASEALDPHAF	CAA35390	UL75(gH)
FLNHQCRVCHFDITP	CAA35333	UL97
VLCMGGGTRLYIYEP	CAA35290	US23

EPEERELIGRCLPAA	CAA35426	UL27
DLASLALTAEFGLGC	CAA35423	UL24
TVAAVSAFACPSVSD	CAA35385	UL71
SATKFYEAFVSGCLP	CAA35334	UL98
NVFIVQTLRKEMCAK	AAA85873.1	UL134Toledo
DFVDALKTLCHPVLH	CAA35360	UL86(MCP=majorcapsidprotein)
RPVSRGYGVSCASRT	CAA35361	UL87
IQMDDPNHYVRRVAN	CAA35291	US24
DSPDLRYYMPLSGGR	CAA35392	UL77
GGGGDQIMGDKPTLV	CAA35430	UL31
LLSHDAALFRATLKR	CAA35334	UL98
GTTIDVSAESSSVLC	CAA35351	UL78
EREESWRRVVDYSHN	CAA35263	US31
VHFPFFSDLANPQLS	CAA35282	US15
ISAPPGWRLDFVEFE	CAA35293	US26
VLLWCCLLLPIVSSV	P16832	UL115(gL)
LFYDLRDLKLCDGSY	CAA35407	UL48(pp212)
LSRPFNGTTETCDLD	CAA35407	UL48(pp212)
GAPFDDDDYLDYRDE	CAA35386	UL70
KTHLASFLSAFARQE	CAA35390	UL75(gH)
STASTSSTPRSRPRI	CAA35356	UL82(pp71)
ARLERELQKKLPAGG	CAA35407	UL48(pp212)
SAPADIAKVLISLKP	CAA35274	US7
LILTESLAFCHCCLN	P09704	US28
YSQFVDHNLSSEITK	CAA35340	UL105
LLFFVRRALNKKYHP	AAA85896.1	UL154Towne
ALRRDDEDWKPSRLP	CAA35269	TRS1part
RLPSRSGFWGSPPLP	CAA35348	UL110
QPSLREPPTPADELQ	CAA35393	UL34
YYRRRDSPGGMDEPP	CAA35354	UL80A
LDEVRMGTEALLFKH	CAA35333	UL97
RSTGRAPGGDEDDAP	CAA35367	UL93
ILFDGHDLLFSTVTP	CAA35390	UL75(gH)
HANRCRRKAPLELGP	CAA35426	UL27
HFRRRSASLSFLDWP	CAA74075	UL43rev
RLRVLWYVNSFWRSR	CAA35367	UL93
FGWDGETLMELKDNL	CAA35417	UL18
RLLEYRRVVAYDADA	CAA35386	UL70
FQKTTSSVDTSPPYC	CAA35386	UL70
RPDEILVRWEEVSSQ	AAA85877.1	UL138Toledo
ACAQARQHLYDQTQP	CAA35449	TRL1
KTVLRCGLEIDRPMP	CAA35269	TRS1part
GFRGFVQEGLRNYAP	CAA74075	UL43rev
PEWVTQRFPDLTAAD	CAA35353	UL80

AEEKRRRKENYRQGP	CAA35457	TRL9
SFGGPLGPASIDFLP	CAA35368	UL94
FGLRNCQFLAVGPDD	CAA35404	UL45
MRQSYRYASGAVVRR	CAA35293	US26
PRTPYAVSVTPATKT	CAA35413	UL54
MTLDVYCCRQTSNNT	CAA35399	UL40
MLLVFLGPVNSFMKG	CAA35446	UL12
DHTLFPVPSTPSATV	CAA35449	TRL1
LSRERKVPTSLGVSL	CAA35387	UL72
YFLTSGLAAAAHAIK	CAA35360	UL86(MCP=majorcapsidprotein)
STTDGDEDYSGEYDV	P16845	UL22A
PAVRARRRTVAADAA	CAA35391	UL76
MGEDTVPYNKPRRHP	CAA35411	UL52
QSLLGYISEHVTSAC	CAA35338	UL102
IIYKFTYTTPPSPVP	CAA35327	UL125
ETALDAAAEVLSWCG	CAA35338	UL102
QFDWLEEPLLRKLVV	CAA35384	UL69
DETAFFQDDDTTTAP	CAA35340	UL105
VSLYIPEGFFGITFY	CAA35387	UL72
QDISDLSAKISMCAP	CAA35403	UL44(pp50)
SGAADEGLEVRVPYE	CAA35358	UL84
IVDKVKSLSRERFAP	CAA35340	UL105
REGKIPMTFVDRDSL	CAA35397	UL38
KESRGQSRNSVWHLL	CAA35334	UL98
FLPAWSKNAPRLSTP	CAA35377	UL62
EKVPAECPELTRRCL	P16832	UL115(gL)
SQTKCELTRNMHTTH	CAA35390	UL75(gH)
LDESPHSATSPHGLG	CAA35386	UL70
KAISRDKAAFTSSVS	CAA35438	UL5
IWPEKHSLVLRTARD	CAA35428	UL29
GWDGRGLLGPCRDPP	CAA35377	UL62
VSTLAMLRGFAEFRP	CAA35285	US18
RENLLTLGQWELVLP	CAA35359	UL85
DDAPPTYEQAMGLCP	CAA74074	UL42rev
ALPQRRPPPLPQRLL	CAA35415	UL15
GGNATYILPADCRYA	CAA35319	UL117
NPINNHVDADSSQGG	CAA35340	UL105
MTSRRAPDGGLNLDD	CAA35425	UL26
QSIADNNCSRRHRVD	CAA35447	UL14
ENSVEGGHLLRNIKT	CAA35423	UL24
HPIGGLFWVGRDPPN	AAA85894.1	UL152Towne
VAEEVHTVLLSWKVL	CAA35432*	UL33
DDDTEGDPDHYPPPL	CAA35384	UL69
VPFHRFYSNPTICAA	CAA35360	UL86(MCP=majorcapsidprotein)

TDGQYATSLRRLDEE	CAA35424	UL25
VLGNTRRYFDLSVLR	CAA35404	UL45
RSGFWGSPPLPIIFS	CAA35348	UL110
PSAATTAAATTTVHS	CAA35340	UL105
ASAAWRGEARRADRR	AAA85873.1	UL134Toledo
YVVNDGERPQQFFTP	AAA85887.1	UL148Toledo
PCAQYFNTEEIIAAN	CAA35360	UL86(MCP=majorcapsidprotein)
QNTVLITDQSREEFD	CAA35363	UL89
HHGQFMPLTYPPGTE	CAA35423	UL24
RWLSQPEVCVLYVTP	CAA35367	UL93
ISTSTVSGTRNTGNN	CAA35318	UL116
EPCALSLGVPEDEWQ	CAA35338	UL102
LLDGYQKKVQQDLQR	CAA35407	UL48(pp212)
QRQAVSRYSGWSTEY	CAA35411	UL52
DEIYAVLRRDGGALP	CAA35367	UL93
SSMVTVLCPDLRPSL	AAA85876.1	UL137Toledo
IRALTELKLHLSTHV	CAA35372	UL57
DAFFSLLGASRSAVP	CAA35353	UL80
SVALYNETFGKQLSI	CAA35371	UL56
PVRSAVRRTTCGKRV	AAA85890.1	UL149Toledo
IHRAVPQHIADYNDG	CAA35296	IRL14
VLARGMHVEAGRQEP	CAA35367	UL93
AMTAPDTRRQLQHVE	CAA35429	UL30
SSGNNSNFWHGPERL	CAA35311	IRS1
MELKDNLTLWTGPNY	CAA35417	UL18
RGDHGLGKAPDGVGY	CAA35400	UL41
GNNYYAYRDSPDLRY	CAA35392	UL77
LSVELSAGHKKKETP	AAA85891.1	UL150Toledo
STTTNTNVETTTCTN	CAA35461	TRL13
ECGQLGSSRLRWRDG	CAA35362	UL88
SYRWIQRKRLEDPLP	CAA35396	UL37
NFLKHKGDHNVPSEL	CAA35422	UL23
YDVLFRGFAGQPPLR	CAA35338	UL102
PDVVRLYPKPRYDYT	CAA35374	UL59
LRLPRGGGQVWSVVP	CAA35391	UL76
STRARSATEDLDRME	CAA35424	UL25
ITVGISSSLLIGHLT	AAA85881.1	UL142Toledo
LRQELRDLGHRVQTY	CAA35392	UL77
GVYLLQDQYTGDVEA	CAA35459	TRL11
VYLDASNNPCNYSSF	CAA35434	UL1
DLMYLFVSVLYFMPS	CAA35288	US21
FHATFMARAEAALKD	CAA35426	UL27
QAAAASQSPPKDMVD	CAA35354	UL80A
PPSEATAGRWPVDRF	CAA35296	IRL14

HHERQRRRRQAMDVP	AAA85875.1	UL136Toledo
FERYKELIQELCQSS	CAA35412	UL53
AFNKKYHMLQDTVSE	CAA35460	TRL12
EKQWQQDLQYRREFV	CAA35274	US7
TNRVTWFPEHVRGTD	CAA35373	UL58
HMRLRPPPDYEETLR	CAA35360	UL86(MCP=majorcapsidprotein)
PSFLSFTCRLQLEPV	AAA85887.1	UL148Toledo
QRDERDRHRRDRRDS	CAA35424	UL25
EKFTGAFNMMGGCLQ	CAA35325	UL123(pp72=MIprotein=IE1)
NAMGTYRCGAVSDLI	CAA35384	UL69
SQTYEDPAHGNWLKE	CAA74075	UL43rev
YLLPEPALAPLLERP	CAA35386	UL70
TDFRGEVVNTMFENA	CAA35392	UL77
TSNITCNGSLYTVYK	AAA85895.1	UL153Towne
LPPRPPRRRPARTDL	CAA35393	UL34
CAAAVTDVTLSHILP	CAA35369	UL95
YLLPPRGKPVCLAPD	AAA85894.1	UL152Towne
ITFIHYGDKVPEDSE	CAA35417	UL18
LHRVFTQLELRNSYQ	CAA35361	UL87
TSEKATLVEHAEGMA	CAA35351	UL78
IYSMMIEGASRQTGL	CAA35394	UL35
HRRATAMSVIKDCFL	CAA35422	UL23
QLDDLIREQVVFTVC	CAA35359	UL85
ELYRALDAYRARIAV	CAA35367	UL93
ISSTLRSVPAPRPSP	CAA35356	UL82(pp71)
PMPGTGLALPRCGRP	CAA35376	UL61
STAHEEEADKQRTKN	CAA35415	UL15
QMLALIDDELDAMDE	CAA35384	UL69
LFVGNLQARDASGLM	CAA35406	UL47
AVFFQYVKVQFGYHL	CAA35336	UL100(gM)
TLLHRYPINPSPRHD	CAA35450	TRL2
LEADPTAREGELFFF	CAA35394	UL35
LYIRERLPKLRYDKQ	CAA35353	UL80
LCKRICCEFGTTPGE	CAA35335	UL99(pp28)
ADFALQLHKTHLASF	CAA35390	UL75(gH)
MELDSVEEEDDFGAS	CAA35404	UL45
FNERLPVFNFVADFD	CAA35386	UL70
LSFMLPILWLFIAVQ	CAA35279	US12
AADYLCCDDTLEAVG	CAA35289	US22
DYLLHIRQQFDWLEE	CAA35384	UL69
DVEEDLTMTRNPQPF	CAA35357	UL83(pp65)
LVHQSRLVTYSDPFP	CAA35291	US24
GFAEFRPHTTNFAHL	CAA35285	US18
IVRPDWCSMRNSLDE	CAA35293	US26

MVTIPPPQIPFVGSA	CAA35385	UL71
QDKVVSYARDELTKR	CAA35393	UL34
RGAAGTPGFIGFQMP	CAA35376	UL61
GAYRHQFLIYGLEHF	CAA35363	UL89
PSLYHDLCRSCNNTE	CAA35460	TRL12
VAPPGEKKELPAQAA	CAA35261	US29
HDGGFPLPTAFAHEY	CAA35360	UL86(MCP=majorcapsidprotein)
SPFGSGSRRGSQIPA	CAA35295	UL132
SPDGTPSVLSLTRDS	CAA35404	UL45
VFPEDLARNGNILFS	CAA35352	UL79
LHEDTALLDRALMAY	CAA35371	UL56
HKKKETPTEGGWGGE	AAA85891.1	UL150Toledo
TSADGSNTTPSKNVT	P16845	UL22A
EVLSWCGLPDIVGSA	CAA35338	UL102
LAVKRRSVAQRSHVR	AAA85891.1	UL150Toledo
STTHDPNVMRRHAND	CAA35388	UL73(gN)
TTMMGVASTDDDSLL	CAA35358	UL84
LHDCLALHLPELTFE	CAA35358	UL84
DTNTTTEPGLLDVFI	CAA35407	UL48(pp212)
TPTFDDLENITTTRA	CAA35318	UL116
TNASHDTVRIQSLGN	CAA35419	UL20
DYRWLGCQIPIQYAA	CAA35351	UL78
VYSVRCDHCVEPEKA	CAA35344	UL108
NDLLKFFVDRLCCET	CAA35293	US26
CTLADAIKFLNHQCR	CAA35333	UL97
KDIEVQVPIRTRRLL	CAA35419	UL20
VKSLSRERFAPEDFS	CAA35340	UL105
AVPVMYPGAVAAPPS	CAA35353	UL80
GIYMIRVNDGGSTGP	CAA35362	UL88
SVTVEQPSTSADGSN	P16845	UL22A
VDRWPFFPFRALLVT	CAA35340	UL105
DARSGQVLHNDASCY	CAA35447	UL14
TCRVQESAPGVLLVW	CAA35338	UL102
SIQTDKKSVQRRGLF	CAA35379	UL64
YRPPYCLVSSPSPRH	CAA74074	UL42rev
HWVLGDSRPDDIKQR	CAA35311	IRS1
PVVRSPCLQPVRDRN	CAA35447	UL14
DALTELYRDPQFQQI	CAA35363	UL89
HTPQAVATFKFFHQD	CAA35319	UL117
SEIREKLIQIIYNFY	CAA35406	UL47
APLSCNVTQWGRYEN	CAA35439	UL6
DDDSRDELYDVPGIY	CAA35362	UL88
PYLGVFVPHNRQGLK	CAA35358	UL84
CPVYDSGTPMGVLMN	CAA35277	US10

SIAAQRQAVGEMLTE	CAA35360	UL86(MCP=majorcapsidprotein)
HGVSDVPRLGAMDAD	CAA35360	UL86(MCP=majorcapsidprotein)
GFLPVEDLKQMERVV	CAA35407	UL48(pp212)
DCKGPFTQVGYLSAF	CAA35321	UL119
RQTALREIALEERAL	CAA35386	UL70
EKKAKKTQSTTTPYF	CAA35389	UL74(gO)
TEISKDADPISTVTE	CAA35342	UL106
TVALIAADRYRVLHK	CAA35432*	UL33
CDRSYEVINVTGYVG	CAA35461	TRL13
HPSPMIAAAPPAQPP	CAA35384	UL69
AMQLNKLFWENKLVQ	CAA35407	UL48(pp212)
LPVYDVRSPRPLRPP	CAA35445	UL13
LVWGDERLVGPFNFF	CAA35338	UL102
ASYGAPVVGYDQLAA	CAA35354	UL80A
RETRKPSRSTPLPEL	CAA35376	UL61
AQRLSLSFRLITETA	CAA35447	UL14
SAARFDEIRRRRQSI	CAA74075	UL43rev
SQAMAALQNLPQCSP	CAA35325	UL123(pp72=MIprotein=IE1)
LCHPVLHEPAPCLQT	CAA35360	UL86(MCP=majorcapsidprotein)
SQGGSTTDGDEDYSG	P16845	UL22A
EPQVLDFTVRGDKLW	CAA35339	UL103
VSKDDLYNREGYGFK	CAA35379	UL64
TVDYGLTSRTAMTIA	CAA35340	UL105
RVLLQEHEHCLLNGS	CAA35320	UL118
VDVYEFPSEYELLLG	CAA35413	UL54
LPVCHHTLKRDVLRW	CAA35289	US22
TSIAAITTDFRCAPP	CAA35373	UL58
SCALSVLDQRLWWEI	CAA35323	UL121
VAVDFGDHPKNLKMS	CAA35340	UL105
DVISPPSQTPEQSTP	CAA35431	UL32(pp150)
SPYTTEEMLRELARV	CAA35340	UL105
HNSRTWDNVIKTVKN	AAA85895.1	UL153Towne
EEIIAANKTLFKTID	CAA35360	UL86(MCP=majorcapsidprotein)
LLCVATGSEEGEGKE	CAA35450	TRL2
VAVSPNAAIISTAAV	CAA35413	UL54
GFTVFSHAACGASLM	CAA35420	UL21
DEYDELWFPLYFEAE	CAA35318	UL116
DPSYVREHGVPIHAD	CAA35413	UL54
LKPVQLSSGQYECRP	CAA35274	US7
RPGSGGWPEHAEAQW	CAA35312	US1
YLESVKKHKRLDVCR	CAA35330	UL128(viralentry)
IWLGIPDSHNICQHE	CAA35280	US13
CRRESLRTLPWLFWV	AAA85880.1	UL141Toledo
CRRKCPPNGNCEFPT	CAA35443	UL10

TSHTTVCISPHTTVA	CAA35387	UL72
AAAAAPTVMVGSTAM	CAA35311	IRS1
VMRSSAGSLRNYLRH	CAA35406	UL47
VMVSVLASTYTWLHK	CAA35281	US14
TSSASTSVIATTQKE	CAA35321	UL119
CVRFASDSDFQTTFT	CAA35387	UL72
FAALQEQGVEDFSLE	CAA35372	UL57
SHRPVCYNDTGDCTD	CAA35273	US6
PFSLAHLLDAIYNVL	CAA35318	UL116
VRREVPRTVNEMKQD	CAA35360	UL86(MCP=majorcapsidprotein)
DDEDWKPSRLPGEDS	CAA35269	TRS1part
RSLGVLPNDHHYALK	CAA35399	UL40
DPEVMAVYEILSVRE	CAA35372	UL57
KVPEDSEPQCNPLLP	CAA35417	UL18
ELRGVKKKKPTAAAL	CAA35385	UL71
RFYSNPTICAALSDD	CAA35360	UL86(MCP=majorcapsidprotein)
GETRPPPDKFDLMSL	CAA35263	US31
APCGPQRPAEIPKRR	CAA35416	UL17
RQTSNNTVVAFSHHP	CAA35399	UL40
TRNKTKHNVTVTGNN	CAA35396	UL37
LLRNIKTAFGMRVLG	CAA35423	UL24
KPQELLFGPRNESGP	CAA35367	UL93
KSPGPRRINITLIGV	AAA85885.1	UL146Toledo
FRLIERHGFFAVTLY	CAA35273	US6
DTLLYVASRNGLFAV	CAA35403	UL44(pp50)
AAAIGIGWYEPEVSM	CAA35322	UL120
IITHAVIINYYYVAQ	CAA35424	UL25
LPCLQFWQKVCSNAL	CAA35372	UL57
SEAAVRLSRLSLDEV	CAA35413	UL54
HFGVQPRQTVELDLR	CAA35430	UL31
PGIASFAATLLHRYP	CAA35450	TRL2
DRRGLDEVRMGTEAL	CAA35333	UL97
AGRALSYHVLENHVA	CAA35361	UL87
RYRAREQRYSLFGRP	CAA35404	UL45
VQYDTFLSNEYRTGI	CAA35336	UL100(gM)
VRSPPDVVRLYPKPR	CAA35374	UL59
RCDHCVEPEKARLAR	CAA35344	UL108
SGGENPINNHVDADS	CAA35340	UL105
SMNCHLNCTRNHTQI	CAA35396	UL37
VANTITEFFRMGLLK	CAA35291	US24
SQHHAVSPELPSRDG	CAA35445	UL13
PIAFVHLKDTEVQRI	CAA35431	UL32(pp150)
AANKTLFKTIDEYLL	CAA35360	UL86(MCP=majorcapsidprotein)
VQIVYGSTRICKSLA	CAA35319	UL117

ADLLKGQRILVARHL	CAA35407	UL48(pp212)
RVINMKAALSSIAAS	CAA35361	UL87
ELNCKKLVEMMEQHD	CAA35341	UL104
HTEHGLLVSMAEYRS	CAA35339	UL103
AQLNRHSYLKDSDFL	CAA35390	UL75(gH)
TTPHTSVTSQASTIG	CAA35460	TRL12
LLEPIEEAALDDVNI	CAA35366	UL92
ASRHELNCKKLVEMM	CAA35341	UL104
NATSSENTTTVMSTL	CAA35396	UL37
NSKVRACVIGYQGTV	CAA35340	UL105
RLLDFRLYAQGTYAV	CAA35372	UL57
DLEQMTDSVRRYSTV	AAA85887.1	UL148Toledo
LRYRQNPFCEPSRNR	CAA35389	UL74(gO)
VDLAKRALWTPDQIT	CAA35390	UL75(gH)
LGLDQRAQPLLDKFN	CAA35407	UL48(pp212)
YQKGYNCTDKHITLS	CAA35439	UL6
PDLHLLYPSQSHRLS	CAA35331	UL129(viralentry)
ELRFHNPDLSSVLEE	CAA35431	UL32(pp150)
STTSSRKGFLFTKQI	CAA35372	UL57
WWWAVRANLATPWYV	CAA35291	US24
GYPSLGYKCSDDPSP	CAA35281	US14
MMHPELGLAHSCNEA	CAA35363	UL89
VVTDFYKVGNITLYT	CAA35360	UL86(MCP=majorcapsidprotein)
GSSGGGGGSGLLPAK	CAA35372	UL57
LRVNIRNRFIPANIP	CAA35415	UL15
LVLLLGRYETVWCLD	CAA35293	US26
KDVVYTAGEGDVVQM	CAA35397	UL38
GKLEYRHTWDRHDEG	CAA35357	UL83(pp65)
VKNTNIPLGIHAVWA	AAA85895.1	UL153Towne
GHARRPRRKRCLVPE	CAA35428	UL29
RVARRRSSSDIPFSC	CAA35415	UL15
LRTWRLLPMVLLAAY	CAA35437	UL4(gp48)
QMPRLGGRSGNFPPP	CAA35376	UL61
VSEVDDYVTAVSGYL	CAA35361	UL87
ETGVTRPMMSLAHIN	CAA35385	UL71
KKEERTTRSPVRLRV	CAA35415	UL15
PWLMEQPPPPSRQTK	CAA35311	IRS1
DQIMGDKPTLVTLLT	CAA35430	UL31
RGYLYKGTDGLPTTD	CAA35443	UL10
REQTRYSQRTTQCVA	CAA35370	UL96
CFGRYTVPFSGPSVP	CAA35381	UL66
TSIQNLLCAIPHRQP	CAA35415	UL15
PSQRYGADAASEALD	CAA35390	UL75(gH)
HQVIPLCASEPEDDD	CAA35311	IRS1

HVVCAHELVCSMENT	CAA35357	UL83(pp65)
AARCPRTGLWIVRDR	CAA35367	UL93
SPAYYRRRDSPGGMD	CAA35353	UL80
SAARAALQWLDLGPH	CAA35405	UL46
HLFQSQRRGGEENRP	CAA35356	UL82(pp71)
RNGLFAVENFLTEEP	CAA35403	UL44(pp50)
LTDRIDSQLVLSRLP	CAA35387	UL72
ILESERRIREGKIPM	CAA35397	UL38
LDFTVRGDKLWLHTE	CAA35339	UL103
EFDYDEDATPCVFTD	P09704	US28
ECWSVRETKRCCRIC	CAA35420	UL21
ALWREMDTVSRHSAG	CAA35404	UL45
QERCKLLVKELRMCL	CAA35431	UL32(pp150)
VNCEASYSHDQVSLN	CAA35321	UL119
RGGLRNNLDNGSDRR	CAA35367	UL93
QKLITRDICVARLQA	CAA35424	UL25
SEFFGRVLAQLHRDR	CAA35361	UL87
QEVLSNEEAETLRYV	CAA35367	UL93
RGSRRETRKPSRSTP	CAA35376	UL61
DKAFAMLTACVEVWA	CAA35338	UL102
YNECGVELPGGDSSD	CAA35289	US22
PWKPGQRVALVWPKD	CAA35422	UL23
TPRHRRRPERSKTPD	CAA35310	J1I
AGAVRNHRRLSFFDD	CAA35451	TRL3
GFRVFVYDLANNTLI	CAA35311	IRS1
REPPTPADELQTAVS	CAA35393	UL34
HSVETLPALQGGLWE	CAA35289	US22
LAVWAALRGVPLPPD	CAA35426	UL27
KSDPLFEDRLLAYGV	CAA35320	UL118
HYEHGLGRLLSVTLP	CAA35392	UL77
VSPYDRFRLIETPDE	CAA35369	UL95
SDAVVCELAFSFASV	CAA35372	UL57
SENAVRRRHERRRYN	CAA35445	UL13
VIREQLSYLMTGTVR	CAA35407	UL48(pp212)
ENKQFTEHTHKVVSF	CAA35429	UL30
TSAFVPSVYMPPTVP	CAA35269	TRS1part
KFHQGIAQLKRAPAE	CAA35393	UL34
AVCAETKVATNCLVK	CAA35458	TRL10
NVGPIYLCPVPAFFT	CAA35411	UL52
CCKHPGRFRFADEEA	CAA35295	UL132
VSEIRSAHFRVEENQ	CAA35314	US3
AIHELKRDLFAARQS	CAA35354	UL80A
RTTVVRRDDVNAERP	CAA35282	US15
LTPQALVARGPSLAH	CAA35413	UL54

KVAMQPVSLRDPEYD	CAA35295	UL132
AISYGRDLWHHETRE	CAA35351	UL78
GKSRKHREYRAVACR	CAA35368	UL94
MMKMAITGKESICLP	CAA35412	UL53
HHDGMVATPYVVFMG	CAA35291	US24
HLTIQVLESWFTPWV	AAA85881.1	UL142Toledo
TGEDTFSAHGKSDFV	CAA35372	UL57
PKPLETTTASNVTTI	CAA35440	UL7
VTSPATRTTSPNALL	AAA85875.1	UL136Toledo
ELPHTASLRALAGCM	CAA35422	UL23
PPSPGPMHMVVCMPA	CAA35419	UL20
FDMDMMEMPATMHPT	CAA74074	UL42rev
ASDSDFQTTFTLPQS	CAA35387	UL72
HDDGPGLDNDLMNEP	CAA35403	UL44(pp50)
MENLRRVYKNTDTKD	CAA35360	UL86(MCP=majorcapsidprotein)
WFLRPTRGTDKVPNN	CAA35289	US22
SQHDTNRVTWFPEHV	CAA35373	UL58
TGAVYACDVRDDRYI	CAA35423	UL24
MHVIFENPDVHIPCD	CAA35412	UL53
DVNPHNPSEIVRAAL	CAA35333	UL97
QRRRRQAMDVPDPEL	AAA85875.1	UL136Toledo
TSDPKGVWSDDDSSM	AAA85884.1	UL145Toledo
GQLSLSTFTMSTVGF	CAA35404	UL45
RKSRGLAGVTRQIHR	CAA35296	IRL14
EDDFGASLCKVSPPI	CAA35404	UL45
YAYIYTTYLLGSNTE	CAA35414	UL55(gB)
YSLQFHDRCASYNDT	CAA35382	UL67
GARAGNQCVCNGIMF	CAA35407	UL48(pp212)
GGLLTRFVQRHTGLP	CAA35352	UL79
LYNAVKEFCLRHQLD	P16832	UL115(gL)
VWRGGEPLKVTLWTR	CAA35430	UL31
DAVWRVQGTFYPEKG	CAA35275	US8
IFVSVGGGPPLTESY	CAA35421	UL22
DEKNIFTPIKKPGTS	CAA35431	UL32(pp150)
ISHPESSLDTGIYHH	CAA35386	UL70
QAVQSVMKDAESMQM	AAA85874.1	UL135Toledo
QEQGVEDFSLENLRR	CAA35372	UL57
IRYYVSVYDELTASE	CAA35295	UL132
AEVLTLATKHPAICA	CAA35269	TRS1part
DESLMKGKHSRYTRP	CAA35269	TRS1part
EESNVVSQTATRVRT	CAA35445	UL13
IHKKTKKPKGKEDES	CAA35269	TRS1part
GLAQRALRDRFQNFE	CAA35367	UL93
AADFEQLRRLCAPLA	CAA35411	UL52

ELQKLWLGVEYHHEV	CAA35361	UL87
TDVWDLVKVEEPVSR	CAA35368	UL94
ARMMIEEEEDEEGGE	CAA35411	UL52
SVAAMFQMSPPLQSA	CAA35413	UL54
GATKKDLFDAVTLCA	CAA35319	UL117
TSHEHFGLLCPKSIP	CAA35357	UL83(pp65)
IRNTLSAPTSPAAAT	AAA85891.1	UL150Toledo
ETTTASNVTTIVTTT	CAA35440	UL7
CDGEVEGHGEHLVPT	CAA35361	UL87
LLDPISHPESSLDTG	CAA35386	UL70
MATISTSITPMMGNP	AAA85876.1	UL137Toledo
EENEEEEEELFPSCK	CAA35393	UL34
AMISSSCSTTCTPLI	AAA85891.1	UL150Toledo
SMRDDNWGLLFRTLL	CAA35314	US3
TLARDIVLVSAITLF	CAA35448	UL16
LGPAVVAAPGPSVRY	CAA35426	UL27
RDSPGGMDEPPSGWE	CAA35354	UL80A
VGDNLNTFMCLGLNL	CAA35423	UL24
FEAAVFDETRAARLS	CAA35352	UL79
DAMFEAGNVPSALLP	CAA35425	UL26
IGGTVFVAYHRDSYE	CAA35414	UL55(gB)
EDFAHQCLQAAKKRP	CAA35273	US6
SHRLLTLMNNCVCDG	CAA35273	US6
RDFRCLNYTHRNPQE	CAA35386	UL70
QLLDDVICCPERLIV	CAA35290	US23
DLKRQWSGLSLHCAW	CAA35459	TRL11
YFGHLNIKGLEKTFL	CAA74075	UL43rev
DLELQETLATEYFAL	CAA35407	UL48(pp212)
FIGFQMPRLGGRSGN	CAA35376	UL61
FVERPETLQIFYNFH	CAA35394	UL35
EREEDTLREMALKAF	CAA35424	UL25
HVSPAQTYTLEGYTS	CAA35340	UL105
IQQFYQWWKPDTTSC	CAA35442	UL9
LLPVGTVSRCEAIGR	CAA74075	UL43rev
MTCLSEMLNVSKRND	CAA35389	UL74(gO)
LGQGSFGEVWPLDRY	CAA35333	UL97
NHAVCLDAELHTLLD	CAA35386	UL70
PCGSGQRVTKVCTDY	AAA85883.1	UL144Toledo
KHDPENVIYFRKDGN	CAA35295	UL132
CVEPEKARLARRLRI	CAA35344	UL108
DEDDDDDDDEKNIFT	CAA35431	UL32(pp150)
DLSAKISMCAPDFNM	CAA35403	UL44(pp50)
FGLIFFVFISLLWLL	CAA35421	UL22
GTGPQPGSAGMGGAK	CAA35431	UL32(pp150)

LIYHTTLLMLTPVVW	CAA35281	US14
ASGTEERYAMACLPR	CAA35361	UL87
HLAVLDRTEFDTDVR	CAA35431	UL32(pp150)
ITSIQKYLNTTCIER	AAA85881.1	UL142Toledo
VRQFSQSDLIIRPTI	CAA35387	UL72
CLHVFKLRRGCRAPP	CAA35426	UL27
YAALVGQDKLVRLAR	CAA35428	UL29
PSLILVSQYTPDSTP	CAA35357	UL83(pp65)
LFVCTLPLWMQYLLD	P09704	US28
PPPAPPPLPSPPRYP	CAA35453	TRL4
ASAARERMKHDPENV	CAA35295	UL132
TCRCWSSSIVLYEHL	CAA35380	UL65
RVRWEVYISRARLVN	CAA35328	UL126
GMHVEAGRQEPETPR	CAA35367	UL93
RHMEDLPKLAEETAR	CAA35404	UL45
SLAWPHDGVYLPKDA	CAA35354	UL80A
GGLHSNYTNLTEAFR	CAA35419	UL20
PPCGTVPSMTCLSEM	CAA35389	UL74(gO)
SYCRLDFFRPSAPVS	CAA35377	UL62
EGITLFYGLYNAVKE	P16832	UL115(gL)
AKEESDSEDSVTFEF	CAA35403	UL44(pp50)
VVQREKQQLKAWEER	CAA35431	UL32(pp150)
KLLRFVVNDGTRYQM	CAA35332	UL130(viralentry)
YQNDTVIRNFSDITT	CAA35448	UL16
TRTAESHELVNQRHK	CAA35455	TRL7
AAELSHFLRAGVLGA	CAA35311	IRS1
KSFQPKTINNSTPLL	CAA35403	UL44(pp50)
MSNTAPGPTVANKRD	Q7M6N6	UL48A
EDLTVYESIKQEKIR	CAA35373	UL58
CPRRFCFSPLDSSAT	CAA35427	UL28
QYEPFASAPHPASLL	CAA35311	IRS1
LAGQQLITQTMPHTP	CAA35319	UL117
TTTARSLKAKTMEMR	CAA35262	US30
LRNMTLTLMRRVEGN	CAA35359	UL85
PNTYIHKTETDERGQ	CAA35356	UL82(pp71)
QRHTGLPVVFPEDLA	CAA35352	UL79
GRLRRWLSQPEVCVL	CAA35367	UL93
LCTVISCVENCNLTR	CAA35339	UL103
LSLQEFCVLIAALYP	CAA35407	UL48(pp212)
GQVLPVVWPPGWNLV	CAA35423	UL24
IEANSVTFKRQVELE	CAA35415	UL15
WLHRDPRGPGCDKNE	AAA85885.1	UL146Toledo
LLIQDGMYGRGEKEL	CAA35323	UL121
EMCPGTTIDVSAESS	CAA35351	UL78

VGGMRQLLFNKLNNL	CAA35384	UL69
TTCVETMCNEYKVTS	CAA35325	UL123(pp72=MIprotein=IE1)
KGDDGVPGGGAGGGG	CAA35372	UL57
RPTPGWHDAALLMDD	CAA35362	UL88
SVEEEDDFGASLCKV	CAA35404	UL45
DFRDLLNFIRQRLCC	CAA35289	US22
NRLCLDLARDEARTV	CAA35369	UL95
ATQEPLDTGLYAPSH	CAA35269	TRS1part
QIPWDDTHRQCAGSV	CAA35310	J1I
HVSTHAGWAAAVVTV	CAA35434	UL1
EQHAAKKQNIYERIP	CAA35458	TRL10
PARKPSASRRLFGSS	CAA35431	UL32(pp150)
QKVFYLCLMPAMTNN	CAA35360	UL86(MCP=majorcapsidprotein)
LYKGTDGLPTTDYLS	CAA35443	UL10
LTTRNAGKYTRHHRD	CAA35433	TRL14
GGPSDGLGGGRGGGG	CAA35372	UL57
ISFNFFQSYNQYYVF	CAA35390	UL75(gH)
RGVFRAKPYSFLIKN	CAA35421	UL22
PLCASEPEDDDEDPT	CAA35311	IRS1
DFMRDFTQLLESCDI	CAA35411	UL52
GEERGGGGKPPLGSP	CAA35376	UL61
QSREEFDILRYSTLN	CAA35363	UL89
NSNFWHGPERLLLSQ	CAA35311	IRS1
QFLNQVDLTETLERY	CAA35390	UL75(gH)
ARNNTRGGGGGGGGR	CAA35315	UL113
RTTQCVAAHLLEQRA	CAA35370	UL96
PAPAPTPTFAGTQTP	CAA35431	UL32(pp150)
FYGTRYIRDELPAAQ	CAA35280	US13
AQYTCGFLVRIELGV	CAA35383	UL68
TPLSEAMFAGFEEAS	CAA35356	UL82(pp71)
LDKLTFRPCSEEQRA	CAA35363	UL89
YTTSSGAKISGVMVS	CAA35360	UL86(MCP=majorcapsidprotein)
LSELLNKWVSQRRAV	CAA35413	UL54
IACLIRQSVQSSTLI	CAA35363	UL89
TDADLTPTLTVRVRH	CAA35358	UL84
PEGLCAQDGLYLALG	CAA35311	IRS1
TWPLRLLLGFYSTVG	CAA35283	US16
MFLGYSDCVDPGLAV	CAA35438	UL5
DIVDKCLNMWERKAA	CAA35340	UL105
GASIALEDLLPMRLA	CAA35358	UL84
KTTHAYTNAAFTSSD	AAA85878.1	UL139Toledo
RQKRRTGAWRPFHDC	CAA35452	TRL5
MLSGPRWISLSDGAP	P09724	US20
YKQRVKYVEDKVVDP	CAA35414	UL55(gB)

LRQRKDDLSYKDIPR	CAA35413	UL54
NVSASTRCLLESVYT	CAA35405	UL46
PDVLLARMLKWYHWR	CAA35404	UL45
AREFLLSHDAALFRA	CAA35334	UL98
KYAACVPQVRMDYSS	CAA35314	US3
PVAAGNNYYAYRDSP	CAA35392	UL77
ILKLVVGEFAMSEAD	CAA35385	UL71
STALFASCYNTNSIR	CAA35363	UL89
GFFPGEINITFIHYG	CAA35417	UL18
EWQVFGTEAGGGAVR	CAA35338	UL102
GPHLLHRRLETLGCV	CAA35405	UL46
DWISKQPLRGRTRRD	CAA35296	IRL14
DSVIDLLTCRWVRYC	AAA85872.1	UL133Toledo
LIAVVYSSCCKHPGR	CAA35295	UL132
KELFFCLEPMEITRY	CAA35291	US24
SDIEELLEQAVQSVM	AAA85874.1	UL135Toledo
NHDIVPFMHAEDGRL	CAA35412	UL53
PRPPHLIEFPPSPPP	CAA35419	UL20
FTLINGVWVVVFLVN	CAA35283	US16
FHIAISTAFCGMIWL	CAA35280	US13
NETLYLLYNREGQTL	CAA35332	UL130(viralentry)
VAHLWGVTPSVWASR	CAA35404	UL45
RKEVNSQLSLGDPLF	P19893	UL122(IE2)
NARRPRTRVPPHEQK	CAA35393	UL34
NTSAWTLHAAGTESG	CAA35407	UL48(pp212)
RVMHPHAFGMRAVSQ	CAA35266	US33
LAVEQFISRFNSGYI	CAA35363	UL89
NPCRSIKRNAFQTAP	CAA35272	US5
NALTTKNSPRLRMRT	CAA35375	UL60
EGFVVIREQLSYLMT	CAA35407	UL48(pp212)
QYPRGFGDVSGYRVS	CAA35276	US9
EPQPNVRLLVIGPRT	CAA35415	UL15
TRHAVSTVLDRWSRD	CAA35334	UL98
LFHVAKLVVIGSYPE	CAA35338	UL102
SPGLLTIYSVLTTLS	CAA35284	US17
DDTHRQCAGSVGHNT	CAA35310	J1I
GNWSVTNLTESCINR	CAA35419	UL20
LTRKCLHDLLQYLDA	CAA35339	UL103
MPPPLSAQASVSYAL	CAA35311	IRS1
CRFQQEPMGGAARRI	CAA35360	UL86(MCP=majorcapsidprotein)
RGTVPLGWVFFVLCL	CAA35448	UL16
AVTQTASRDAADEVW	CAA35431	UL32(pp150)
FSVSFVFTLECGRCL	CAA35263	US31
VVTMAAAIGIGWYEP	CAA35322	UL120

SGSYAGLSLSSRRCD	CAA35353	UL80
VREHGVPIHADKYFE	CAA35413	UL54
QHRVRLRVLWYVNSF	CAA35367	UL93
DAQALGLDLATVVME	CAA35269	TRS1part
FTTLGLRCPDNRLSG	CAA35367	UL93
VTRQIHRAVPQHIAD	CAA35296	IRL14
YQVYFYGLQCPEQLV	CAA35261	US29
GYSAVFLLETEDAVT	CAA35339	UL103
LPSMIAFMAAVHFFC	CAA35336	UL100(gM)
ELGLAHSCNEAFLPL	CAA35363	UL89
LVSVYECVPDANTAP	CAA35427	UL28
WLDGGFSTAVEGDAK	CAA35363	UL89
TLPMGTTGSYTPPQD	AAA85878.1	UL139Toledo
GHHRRRPACALPHGW	CAA35347	UL109
LLLWKVTNADSVFAP	CAA35392	UL77
IFQMCSESRVLVGSY	P09724	US20
MLGSIEGLRACRPFD	CAA35289	US22
FLLWLSPVSSSPSRS	CAA35456	TRL8
PRRVASAAWRGEARR	AAA85873.1	UL134Toledo
IKHYQLGLHQFVDHT	CAA35359	UL85
FFSSPTSSPSHSFYI	CAA35383	UL68
MTLFCRTANSTAGYV	CAA74073	UL41alt
YGRDPEWVTQRFPDL	CAA35353	UL80
TVFDTTRLFEIAWSE	CAA35284	US17
TYLDGERAKGDLIFN	CAA35417	UL18
KETTRQGGAAFACTR	CAA35459	TRL11
LRDAWTHKRPKPRER	CAA35427	UL28
PSSSPKKTPEKRRKD	CAA35407	UL48(pp212)
TLPALQGGLWEDNDE	CAA35289	US22
GTVERFVDILQKDTF	CAA35340	UL105
RLCCGWLALGAVLPA	CAA35311	IRS1
GEPLKVTLWTRTRSI	CAA35430	UL31
KRIALAVATGQYVVC	CAA35426	UL27
AARLVSTYRDRDIDL	CAA35407	UL48(pp212)
FAAFRSSYCEGGGGS	CAA35280	US13
VLAYHLYGGDGATAA	CAA35386	UL70
VLSDLEAAACLLAAY	CAA35392	UL77
QTLVDVARGKFARER	CAA35405	UL46
FFSQCEHYPSFVKLN	CAA35261	US29
ERLPKLRYDKQLVGV	CAA35353	UL80
RERLGYNSKFYSPCA	CAA35360	UL86(MCP=majorcapsidprotein)
LSPEWVKSFDFREHF	CAA35290	US23
ACRRRPGDLGFTVFS	CAA35451	TRL3
TTALVADVHDLRHSD	CAA35447	UL14

RSIVIPQGTPIATLY	CAA35430	UL31
SAVLSGVYSYLMTHA	CAA35366	UL92
PPPDRSMLLSREEEL	CAA35438	UL5
PGLRRHRKNTTRSHA	CAA35457	TRL9
RLQDLRVTFHRVKPT	CAA35361	UL87
TVVHGQPGSHRHLGW	CAA35316	UL114
WMQYLLDHNSLASVP	P09704	US28
AAISEAEAASGSFGR	CAA35356	UL82(pp71)
SAEVTKRGYASYTID	CAA35395	UL36
PLDLELGLMRVDTHP	AAA85874.1	UL135Toledo
YDVVSTNIEFGAWPV	AAA85886.1	UL147Toledo
TIYNLTTRNAGKYTR	CAA35433	TRL14
GAYAVLMATSQRKSL	AAA85884.1	UL145Toledo
RVHNHILNGFLPVED	CAA35407	UL48(pp212)
YEFPAPTPQATTAGG	CAA35369	UL95
SRDLSRKTNLPIWVP	CAA35334	UL98
EVIASVGELVPEPRT	CAA35413	UL54
LRALKNTADAMERGL	CAA35360	UL86(MCP=majorcapsidprotein)
CQEYLHPFGFVEGPG	CAA35395	UL36
QLERINTLSESVFSP	CAA35386	UL70
DPHDRDEVARTDEWK	CAA35367	UL93
WYARDPAVTLSQLFP	CAA35392	UL77
YEDIDITGERQRLRF	CAA35262	US30
LINTGITVCTGFCGE	CAA35285	US18
NFEAVLARGMHVEAG	CAA35367	UL93
VTLHGLAQRALRDRF	CAA35367	UL93
HGDFGTGTFRTAVSP	CAA35271	US4
ERGEFGDEDEEQEND	CAA35394	UL35
PDKVVEFLSGSYAGL	CAA35353	UL80
HCTSHMYELSLSSFA	CAA35388	UL73(gN)
RAKRFDARADLAVYH	CAA35408	UL49
RLSERREHLVFMLWG	CAA35316	UL114
TSPNALLPEWMDAVH	AAA85875.1	UL136Toledo
MLILALWAPVAGSMP	CAA35278	US11
RLYDAITENLMHCVK	CAA35395	UL36
RDFILPNHYSKGTTV	CAA35413	UL54
LAVWLRASVLVAFQL	CAA35286	US19
PRAEIRRGGGDWADS	CAA35449	TRL1
RRGRKWGRLHAPECL	CAA35275	US8
ANLLRVCQLHAGSKI	CAA35427	UL28
HPTDEQYANMESRTD	CAA35384	UL69
TDEDFKPEDVKAWSH	CAA35423	UL24
TIRSILAADERLRIK	CAA35341	UL104
HGGPYLRRSLFAAGP	CAA35282	US15

NHPEQICRSPPPPLP	CAA35424	UL25
VIGPGHTQTVYFDAA	CAA35387	UL72
HGLHTLMRETALDAA	CAA35338	UL102
FHEQFLARRRHAEVG	CAA35451	TRL3
SHDGIPRQLAERLRL	CAA35430	UL31
ENFKQLEITPADLRT	CAA35385	UL71
QLTLIWPEKHSLVLR	CAA35428	UL29
RGVTTSPCPTRSLVY	P09724	US20
GTYVNSSVTCWGSNG	CAA35322	UL120
QALCDSPALSLCTDD	CAA35395	UL36
RRPARSGGDGGAPMT	CAA35337	UL101
EAVIDIFPTGQTMSF	CAA35341	UL104
LLLCGQAENLKGVWP	CAA35293	US26
LCSMTEELYLRLDGT	CAA35426	UL27
LNGGLDRHMHRIHPF	AAA85876.1	UL137Toledo
MSSGGGGGDHDHGLS	CAA35403	UL44(pp50)
LYDQTQPLLLAYPNT	CAA35449	TRL1
PCTHKALHHPIGGLF	AAA85894.1	UL152Towne
TYWYSGNAYNHTIDT	CAA35419	UL20
RNQSRNAIPRPSFPP	CAA35445	UL13
YRLADSLEELFRAGL	CAA35290	US23
PPLPGHARRPRRKRC	CAA35428	UL29
HCQENSETVALCTPR	CAA35360	UL86(MCP=majorcapsidprotein)
LVALLPHERHRELCH	CAA35393	UL34
LFEEKEPHIVQYVPA	CAA35339	UL103
PDRQIPKNITCANYS	CAA35437	UL4(gp48)
GLTSRTAMTIAKSQG	CAA35340	UL105
TCVDDLCRGYDLTRL	P16832	UL115(gL)
DYLFKRMIDLSSIST	CAA35414	UL55(gB)
GYYRELRFHNPDLSS	CAA35431	UL32(pp150)
HGGDSTHLRRRVPSC	CAA35384	UL69
SVSLVNLLTIIGCLW	CAA35351	UL78
HTLVMRLARDESPRP	CAA35407	UL48(pp212)
TSCISTAFTNVATLC	CAA35363	UL89
AAAPEGITLFYGLYN	P16832	UL115(gL)
DDDDYLDYRDERDTE	CAA35386	UL70
VREEIPASDDVLFFV	CAA35372	UL57
GTVSRCEAIGRPPLP	CAA74075	UL43rev
LSTVDDHKAWLDLDE	CAA35311	IRS1
GARTRDFRCLNYTHR	CAA35386	UL70
TATVTTPPMIDLTSH	CAA35319	UL117
PTGRSICPSQEPMSI	CAA35357	UL83(pp65)
VYPLAAELSHFLRAG	CAA35311	IRS1
PVEYPAGEVQYQRTK	CAA35441	UL8

LLRSTETAVNPSNAT	CAA35441	UL8
LLGEHGAALVSHTLR	CAA35333	UL97
AATPSRKKKARRGPK	CAA35376	UL61
VPRDRRGRSFCRPTR	CAA35391	UL76
PPNEHRFFSLRTRQT	CAA35387	UL72
REQRYSLFGRPVSRR	CAA35404	UL45
ARCAYVEAHREAQLT	CAA35428	UL29
GVDLRLPVLHPTSSP	CAA35408	UL49
NSSTHSQRNNGGGGR	CAA35371	UL56
DESGRPRRIANRIGD	CAA35289	US22
AKTITFRPPPCGTVP	CAA35389	UL74(gO)
DEVTVMSPSPEPVQQ	CAA35385	UL71
IRRLRDYLRFPTRLE	CAA35407	UL48(pp212)
RSIFFILSVMIGKGT	CAA35398	UL39
HEIQNDGQVLTVTVK	CAA35274	US7
QQINNFMTDFKKWLD	CAA35363	UL89
ILIGTLVPILLHEQK	CAA35434	UL1
EFALHVKGLKTAGVP	CAA35372	UL57
HPVPGVEFRGERETP	CAA35334	UL98
SPPPPLPPRDYPQRD	CAA35424	UL25
VYALPLKMLNIPSIN	CAA35357	UL83(pp65)
QLAEEPLSAYVNALH	CAA35360	UL86(MCP=majorcapsidprotein)
YVLLQPSEDVELREL	CAA35385	UL71
GTQDKGQKPNLLDRL	CAA35414	UL55(gB)
YRYSETCMEVTVRVG	CAA35443	UL10
FYTHYRSVNGDLAVE	CAA35406	UL47
PGAFMDEIIGGTNKI	CAA35363	UL89
REPTKDLDDSFDYLV	CAA35351	UL78
WRDALFTSTLLTVMV	CAA35281	US14
DVYQRRWKKTVLRCG	CAA35269	TRS1part
EVDPAADPTVGDKAG	CAA35311	IRS1
NAAPETHRLVAFLER	CAA35414	UL55(gB)
TTILLYPPTSTYNSL	CAA35442	UL9
NEPNVFSLTVHAPYD	CAA35430	UL31
ELLEQAVQSVMKDAE	AAA85874.1	UL135Toledo
AAMLYKISPVSLVLQ	CAA35360	UL86(MCP=majorcapsidprotein)
VFEEVSRLMRLHDST	CAA35319	UL117
QDHSRLVLDEAFPTF	CAA35407	UL48(pp212)
HDCCCHLGSSVFSRP	CAA35452	TRL5
FGRRYNVDAKDGGLD	CAA35372	UL57
GTVRAEGKKKLLLKH	CAA35393	UL34
SIQVLAANLDCVITG	CAA35340	UL105
ELQEHLFVRGGIVFN	CAA35315	UL113
FGDEDEEQENDGEPR	CAA35394	UL35

GLYAPSHVTSAFVPS	CAA35269	TRS1part
SFIVKQCHTQLAFYN	CAA35387	UL72
LDARSAFVEARGLYV	CAA35372	UL57
LVLLTVGVSARLRFI	CAA35314	US3
LRRLRLYPPERLHAL	CAA35408	UL49
MWAENYETTSPAPVL	AAA85880.1	UL141Toledo
YWESRTDHVPCFIFK	CAA35409	UL50
AEDKTFFHRVRILCG	CAA35428	UL29
RRSSSDIPFSCRDCE	CAA35415	UL15
VTLGGAGAWLLPRPE	CAA35368	UL94
AIVYSNYTVERVTLP	CAA35341	UL104
KVYDRWKDNKQYGQV	CAA35441	UL8
WSSSIVLYEHLDARV	CAA35380	UL65
HQPVIPPQPPAPTSE	AAA85872.1	UL133Toledo
GGGGSGLLPAKRSRL	CAA35372	UL57
GRDMLERTARFIKDN	CAA35413	UL54
ARFADDEQLPLHLVL	CAA35367	UL93
ETPSAREFLLSHDAA	CAA35334	UL98
RHCPPLDQELIMFGV	CAA35397	UL38
AVRAGLLCRTPEDLA	CAA35426	UL27
TYDCVLSDLEAAACL	CAA35392	UL77
NVNVGWFKAATAIVP	CAA35361	UL87
NREGQTLVERSSTWV	CAA35332	UL130(viralentry)
DSYGLLGNSVDALYI	CAA35353	UL80
ERNRHLGAFHLPAIR	CAA35426	UL27
KMRITPDAFEIQRSR	CAA35352	UL79
TASGEEVAVLSHHDS	CAA35336	UL100(gM)
LALHLPELTFEPTLD	CAA35358	UL84
MRNSLDEVSGTADVS	CAA35293	US26
GGVGGSSGGGGGSGL	CAA35372	UL57
FFRLILDEEQRIYDV	AAA85886.1	UL147Toledo
RDLLRHVPDSRFSDE	CAA35386	UL70
AAAKPLPSLCVRDYA	CAA35369	UL95
VAHQKHVSQFVLKEV	CAA35363	UL89
ALGVTCACQEPREVL	CAA35283	US16
GLSCPCPRPRTPTEP	AAA85874.1	UL135Toledo
NCYTDGDLLRTAMLL	CAA35279	US12
QGATVFEPEVGYYND	CAA35413	UL54
IGDIFNMPYFQISIF	CAA35329	UL127
FVGRPRHCRLEMLIL	CAA35416	UL17
SRLLMMSVYALSAII	CAA35390	UL75(gH)
TLLSAALPSAASSSP	CAA35354	UL80A
YSYNHHTVFRQMHAA	CAA35430	UL31
IHKTETDERGQWIML	CAA35356	UL82(pp71)

PAAEVECKKSQRILE	CAA35385	UL71
VLTAESAPATAEVCL	CAA35319	UL117
YNVDAKDGGLDGKGD	CAA35372	UL57
TQQCEMVTEGYTQPQ	CAA35407	UL48(pp212)
AFCGRHRDYNSPEES	CAA35363	UL89
ACPSVSDSLIPEETG	CAA35385	UL71
STQAFDPSRYLRQHG	CAA35415	UL15
CTPTGELTSGGGETP	CAA35354	UL80A
WYADVVRCVRAQVDL	CAA35367	UL93
EDVPSGKLFMHVTLG	CAA35357	UL83(pp65)
LSRLIEKKKRARLQR	CAA35384	UL69
VAELCERGRDDDPPP	CAA35286	US19
TQTEEKFTGAFNMMG	CAA35325	UL123(pp72=MIprotein=IE1)
DSPGGMDEPPSGWER	CAA35353	UL80
PGLDNDLMNEPMGLG	CAA35403	UL44(pp50)
SVDALYIRERLPKLR	CAA35353	UL80
VMRGYLYNTLKTEVF	CAA35405	UL46
LQRVVDAVKGEMLST	CAA35407	UL48(pp212)
DEEEKRRERQKRVDD	CAA35431	UL32(pp150)
TKDEDTLRSVQHFLW	CAA35279	US12
VLQPLITKGGLCSSM	CAA35430	UL31
TTLLMLTPVVWSARW	CAA35281	US14
RQEVYVEGTTCAQCY	CAA35371	UL56
LGCQIPIQYAAVDLT	CAA35351	UL78
TCAALGIWCLAGSSA	AAA85872.1	UL133Toledo
RGGSQLQMVGHPLRE	CAA35398	UL39
NVKKAPCPTGTVQIL	CAA35403	UL44(pp50)
PCDLDIHPSHRLLTL	CAA35273	US6
AAASSSSSPHGLPHV	CAA35408	UL49
VEDYLQDARRRADAQ	CAA35269	TRS1part
THDTIFFPENIPGVS	CAA35356	UL82(pp71)
REASPVPPRSPMPSH	AAA85873.1	UL134Toledo
SVMKRRIEEICMKVF	CAA35325	UL123(pp72=MIprotein=IE1)
IPASDDVLFFVDGCE	CAA35372	UL57
EVYISRARLVNRQIA	CAA35328	UL126
MMRRMRRAPAEAAEA	CAA35315	UL113
PPGSGLAKHPSVSAD	CAA35448	UL16
LEMLLDKFSTDQASL	CAA35385	UL71
NNTSTTPHTSVTSQA	CAA35460	TRL12
TSDLSTFTTVYSTFN	CAA35460	TRL12
ALPSAASSSPTTTTV	CAA35353	UL80
DVVVDATDSRLLMMS	CAA35390	UL75(gH)
GRRYAVFQPRRFTPR	CAA35420	UL21
QGLPDFISRQHVLYN	CAA35360	UL86(MCP=majorcapsidprotein)

LDAENLDCDPEVMAV	CAA35372	UL57
CLRDELHPDRDVILT	CAA35371	UL56
ASARGRYLRSLLAFR	CAA35428	UL29
PLCRDELVAVAPGAA	CAA35407	UL48(pp212)
TCRLNVTELASIHPG	CAA35448	UL16
EQTIKRHPQLREMLR	CAA35407	UL48(pp212)
ERFAAAAKPLPSLCV	CAA35369	UL95
ERPVPCFVVDAVVHP	CAA35282	US15
RVLGYSTPTIYMTNL	CAA35432*	UL33
DSDGEEEEMASGLGD	CAA35312	US1
LDESHWVLGDSRPDD	CAA35311	IRS1
FTYWSRTSEVIVKRV	CAA35371	UL56
GVPAPMAATVARESL	CAA35372	UL57
PENAVTAISYHSILA	CAA35360	UL86(MCP=majorcapsidprotein)
TVSTPPPPPPDCSPP	CAA74074	UL42rev
VLQAVRGRFSGREVP	CAA35369	UL95
WTHKRPKPRERASGV	CAA35427	UL28
RSELVFGQSSRFQRT	CAA35282	US15
DRFRLIETPDENFLL	CAA35369	UL95
CRVDHLTWISKRVTT	CAA35430	UL31
ELSATLARDIVLVSA	CAA35448	UL16
GTHLAGFFGTSTQLA	CAA35386	UL70
KCRITEPITMLGAYS	CAA35458	TRL10
SDAPLPYFVPPRSGA	CAA35358	UL84
ASQAGCLSDVLYNTR	CAA35360	UL86(MCP=majorcapsidprotein)
LHAANHDIVPFMHAE	CAA35412	UL53
APLLQSATRGDSDMF	CAA35362	UL88
VRDQLSSQLVRPSRG	CAA35337	UL101
DWCSMRNSLDEVSGT	CAA35293	US26
ERTPCEQAAYAYSLV	CAA35340	UL105
AFRERARLPDTVCVH	CAA35386	UL70
TGLCSLAELSHFTQL	CAA35390	UL75(gH)
LGLHATTLLTVTLML	CAA35286	US19
SKATRRTSPRYYPPS	CAA35296	IRL14
HRLSFPGESTFCLTA	AAA85891.1	UL150Toledo
STNIEFGAWPVPTAY	AAA85886.1	UL147Toledo
RDLPTVYLVSAIFRE	CAA35334	UL98
LCCEWYVVGLVGYYL	CAA35289	US22
SLAPANKRKTHRTIV	CAA35319	UL117
TSLSPCSTSCPPSPA	P09724	US20
VVYISPFYNGTNRNA	CAA35414	UL55(gB)
VLCCFQENKSPHDTV	CAA35356	UL82(pp71)
TNRSLEYKNLPFTIP	P19893	UL122(IE2)
VGSERSLSYRYHLES	AAA85881.1	UL142Toledo

KRCCRICRWRSTSSS	CAA35420	UL21
RLLPRQFPPPPRFPP	CAA35415	UL15
QATTAGGGGGSGGGG	CAA35369	UL95
RLLAENLRGLNERLL	CAA35406	UL47
ERLLLSQIPVERQAL	CAA35311	IRS1
AAGNVEAQHDTATPH	CAA35442	UL9
ACMMTMYGGISLLSE	CAA35325	UL123(pp72=MIprotein=IE1)
LGCELLAGGRVFHCD	CAA35334	UL98
TSPLNGMCLDCNKTS	AAA85895.1	UL153Towne
EAGQIFVSQFAFRAG	CAA35356	UL82(pp71)
MRCFRWWLYSGWWWL	CAA35261	US29
RGQWIMLFLHHDSPH	CAA35356	UL82(pp71)
TFTLPQSTEEKYDKE	CAA35387	UL72
LFATEYDSAHIVANV	CAA35386	UL70
VSCVIDGGNMTSVWR	CAA35274	US7
TLLTVMVSVLASTYT	CAA35281	US14
IVLHLGNRCQPWRQV	AAA85882.1	UL143Toledo
YMWAGTGVMSTTPLT	CAA35411	UL52
LQTFTERGPPSEPAM	CAA35360	UL86(MCP=majorcapsidprotein)
ANVLSQTPTDHCVFP	CAA35386	UL70
CLDAQFVCMPELLES	CAA35387	UL72
KALNMLCYYTEKLEE	CAA35442	UL9
DRFLRVPLQRAPDPR	CAA35296	IRL14
SMVANLASELTMTYV	CAA35359	UL85
DGDLLRTAMLLYMDQ	CAA35279	US12
PVYRVHLPNDQHVFC	CAA35386	UL70
AAAFGLLQHGGPYLR	CAA35282	US15
DFSDATNMTSSTNVP	CAA35295	UL132
PGKRISLRTEISKDA	CAA35342	UL106
TVHAPYDIHFGVQPR	CAA35430	UL31
GGSGGGGGGGGGGLD	CAA35403	UL44(pp50)
LRETMNNLGVSDHAV	CAA35352	UL79
GSLEGVEERMLNVMK	CAA35293	US26
CSPNNTSSNTGNGSK	CAA35458	TRL10
LFQWLKRFKLLMEVY	CAA35285	US18
FSGNGVERSLNVSSM	CAA35372	UL57
LPRLSIASARWEALR	CAA35404	UL45
TQRQIVFRRLFDSGN	CAA35283	US16
MVDLNRRIFVAALNK	CAA35354	UL80A
PSPISTASTSSTPRS	CAA35356	UL82(pp71)
NVHVSPGWIEANSVT	CAA35415	UL15
TTYHVTQNINTVDMG	CAA35360	UL86(MCP=majorcapsidprotein)
GVPIHADKYFEQVLK	CAA35413	UL54
GGVGVSNDNHGAGGT	CAA35413	UL54

RAPQKVYDRWKDNKQ	CAA35441	UL8
PQEDDSVVMRCQTPD	CAA35384	UL69
KLMKVAMQPVSLRDP	CAA35295	UL132
DGLFLYRTTVSRGVD	CAA35342	UL106
PCTVMTHSWPMVSIR	AAA85880.1	UL141Toledo
HDLNKLTTGKMLFHV	CAA35360	UL86(MCP=majorcapsidprotein)
YGLLTLETAFSVLIS	CAA35281	US14
HVKDDLVKCAEGTVY	CAA35371	UL56
RLIGHLRHYLQNSFL	CAA35393	UL34
DFLSQTSIDIFKQKA	CAA35363	UL89
ICTYNFLKHKGDHNV	CAA35422	UL23
PSLHPSYPVPPPPSP	CAA35354	UL80A
AQTYTLEGYTSDNVL	CAA35340	UL105
PPSQATQPHHYSHHQ	CAA35412	UL53
DGELIYGSYLLYRKA	CAA35369	UL95
IEGLRACRPFDHMPA	CAA35289	US22
RWFPFRQSVFVERFV	CAA35375	UL60
SPSVLPAESPPSLSP	CAA35353	UL80
AVFQPRRFTPRPQHD	CAA35420	UL21
VSTVLDRWSRDLSRK	CAA35334	UL98
LVLLDKFGVVYLHKI	CAA35289	US22
IIGIYSKQTKYDACV	CAA35366	UL92
NPYAVAFQPLLAYAY	CAA35372	UL57
RCRKKREGSPKYSVS	CAA35355	UL81
AANPVMPATATVTTP	CAA35319	UL117
SDESSASSSSRICPL	CAA35276	US9
LLWCEYVYRHPQTPF	CAA35289	US22
LTINATLRLLIQDGM	CAA35323	UL121
PMRFINVKSHVSRPP	CAA35271	US4
TKNTHPSGTVPRRRS	CAA35415	UL15
TFLSNEYRTGISWSF	CAA35336	UL100(gM)
SRARLVNRQIAWRRH	CAA35328	UL126
PRYYPPSEATAGRWP	CAA35296	IRL14
LCLSASSSCAVDLGS	CAA35448	UL16
LTCKCSPNNTSSNTG	CAA35458	TRL10
ELSAQSPAADFSVSE	CAA35369	UL95
HFFTYHVNSSDKASS	CAA35417	UL18
GMRAVSQFLVTHPLG	CAA35266	US33
FENPDVHIPCDCITQ	CAA35412	UL53
LDKVHEPFEEMKCIG	CAA35325	UL123(pp72=MIprotein=IE1)
RAYLTPIRNRQEAVR	CAA35426	UL27
ICCEFGTTPGEPLKD	CAA35335	UL99(pp28)
PGWRLDFVEFEDIFG	CAA35293	US26
DKFGVVYLHKIEDSD	CAA35289	US22

FRGHLNRERWGILRD	CAA35377	UL62
DEGLEVRVPYELTLK	CAA35358	UL84
TYGTYICSPNPGRLR	CAA35323	UL121
GGHPVTHTVDMVDIT	CAA35314	US3
TWHRSLFPRDLLRHV	CAA35386	UL70
TGGGGDSTNHTHTQL	AAA85892.1	UL151Toledo
IPGAANGMPPLTPPH	CAA35395	UL36
SFRVGTHKYVLERDD	CAA35338	UL102
AGGGAVRLNATAFRE	CAA35338	UL102
GHGHAFAYLPGEDKV	CAA35427	UL28
VTFEFVPNTKKQKCG	CAA35403	UL44(pp50)
VPDLQCVSISNWTMA	CAA35413	UL54
ETAVSTPFFEIPNGA	CAA35367	UL93
QLNVYHQLCRALMNG	CAA35371	UL56
MLHVVPLEWTVEEVV	CAA35286	US19
WGSPPLPIIFSFVPD	CAA35348	UL110
RLHLIKHYQLGLHQF	CAA35359	UL85
PLSTPKNACLAEVGV	CAA35265	US34
RSDAAAVVVEPRPRP	AAA85873.1	UL134Toledo
GDSTNHTHTQLTSAV	AAA85892.1	UL151Toledo
KWTFALLVVAILGII	CAA74074	UL42rev
YRDISSTIATEKIPF	CAA35272	US5
ATRVEDMATFRTEKQ	CAA35274	US7
SKESTSGVTPEDSIA	CAA35360	UL86(MCP=majorcapsidprotein)
MESSAKRKMDPDNPD	P19893	UL122(IE2)
GSFVGMLSHVSPAQT	CAA35340	UL105
KLHGREKRLQLDRLV	CAA35361	UL87
THCAKLVVECGQLGS	CAA35362	UL88
EDTHLTCKCSPNNTS	CAA35458	TRL10
GLPIIGVMLVLIVAI	AAA85877.1	UL138Toledo
RALSAPPLTFLATTT	CAA35358	UL84
RTTRLPPHPGFFSWS	CAA35342	UL106
LDEGIMVVYKRNIVA	CAA35414	UL55(gB)
GILALGSFSSFYSQI	CAA35399	UL40
FQRPASGCLDAWARR	CAA35316	UL114
ANTTVSTCINASNGS	AAA85878.1	UL139Toledo
GSKCHAMCKCRITEP	CAA35458	TRL10
TPWGQLICCEESLES	CAA35293	US26
HNRQGLKMPVTVWLP	CAA35358	UL84
DLKVPTAFPQDHQPR	CAA35384	UL69
VSAITLFFFLLALRI	CAA35448	UL16
MVRDPLLRDPRATHL	CAA35424	UL25
SVAHNLYTTVNPLIE	CAA35406	UL47
MDVIRLGEKTVIMEN	CAA35360	UL86(MCP=majorcapsidprotein)

DVDEWFEQRRLAEKE	CAA35290	US23
SPLPLVSFTELLLPP	CAA35430	UL31
RILPTASDAMVAFIN	CAA35412	UL53
RDFHTEVPLYALHGF	CAA35267	US35
ITRIGRDMLERTARF	CAA35413	UL54
RSRHQRTPPSATTHG	AAA85891.1	UL150Toledo
SSQTGSFVGMLSHVS	CAA35340	UL105
FRQMHAAGTTTFFLG	CAA35430	UL31
RPPSSQHHAVSPELP	CAA35445	UL13
EGGWGGEEGEDDVLA	AAA85891.1	UL150Toledo
FTNNHCFILGADSAC	CAA35268	US36
LCAPVVRACRASSFG	CAA35310	J1I
SVCNQNVILPGVIFV	CAA35421	UL22
DLRQLSPRDAWIVLV	CAA35311	IRS1
MDDLPLNVGLPIIGV	AAA85877.1	UL138Toledo
FCRASRVLTDPEPIQ	CAA35429	UL30
RRRTDSSLEAGQIFV	CAA35356	UL82(pp71)
TQRRGAEIDRPMPVV	CAA35311	IRS1
ELSTGEDARFCRPRP	CAA35361	UL87
GWERYDGGHRGQSQK	CAA35354	UL80A
MYMCYRNIEFFTKNS	CAA35325	UL123(pp72=MIprotein=IE1)
GGWPEHAEAQWRQQV	CAA35312	US1
VEDVSREGAREGNDG	CAA35445	UL13
TAPDGAPGRWDGPAD	CAA35376	UL61
WCVDQRRTLEVFKEL	CAA35414	UL55(gB)
LGWQTLSNHVIRRLS	CAA35316	UL114
DLRTRFAELAKRRGT	CAA35407	UL48(pp212)
PPDSDLTRNTKQAQE	CAA35289	US22
RFTGKPTYNLLTYPV	CAA35274	US7
PRHTFDMDMMEMPAT	CAA74074	UL42rev
ELRDQLLDDVICCPE	CAA35290	US23
DDTTTAPDSRETLLT	CAA35340	UL105
HWYNQSCVGISNGEN	CAA35443	UL10
LTDLNIKGRCVVGEQ	CAA35356	UL82(pp71)
RKRYLRQELRDLGHR	CAA35392	UL77
FTIGTCGQVGPDVDV	CAA35413	UL54
VQYTEEDFEAHTKGA	CAA35406	UL47
PLHIGVAGLHTALMR	CAA35404	UL45
ALGPFVGKMGTVCSQ	CAA35395	UL36
LVWLGYPSLGYKCSD	CAA35281	US14
ISARWFRWDGNDSHL	CAA35440	UL7
AAGPSRGGRGAQPEP	CAA35376	UL61
IFVSQFAFRAGAIPL	CAA35356	UL82(pp71)
RDERDTEEDEDGKED	CAA35386	UL70

LVSLARTPLCRRRVG	CAA35333	UL97
TDKRFLNRELGDRLY	CAA35408	UL49
TTSATTTTTTTLSTT	CAA35388	UL73(gN)
TTQARTVERTGSAPK	CAA35310	J1I
RIERPSQSEVDCASL	CAA35418	UL19
FVFTLECGRCLVGET	CAA35263	US31
GDEEDEVTVMSPSPE	CAA35385	UL71
FGRLELDPNESPPDL	CAA35358	UL84
VASDIKAFSKNGLLW	CAA35289	US22
ELRCPCTHKALHHPI	AAA85894.1	UL152Towne
PTVVRRPLRVPVVNY	CAA35404	UL45
DKLEADADPALHRRP	AAA85891.1	UL150Toledo
SLKKKSNFTFCTPPS	CAA35330	UL128(viralentry)
SETVLTVWMSGLIRT	CAA35333	UL97
RDVNFHTPGRHAKTL	CAA35290	US23
KNTNRSFSCLTNHSL	CAA35444	UL11
TPMMGNPTFSGRSSM	AAA85876.1	UL137Toledo
FVEQLRELVRRDRLL	CAA35406	UL47
TDAGLICKNPNYSVC	CAA35409	UL50
YRELRQAFPGLDFEA	CAA35352	UL79
PLKMLNIPSINVHHY	CAA35357	UL83(pp65)
FLLMCALPTPGERSS	CAA35273	US6
MVATVTLFCFLTLAT	CAA35288	US21
ESLVRSVFEHRSLVT	CAA35372	UL57
SAQVKTIYRVFGFVS	CAA35340	UL105
LEFTANNRVSFHGVK	CAA35403	UL44(pp50)
QCVSISNWTMARKIG	CAA35413	UL54
TPALTPVTSPITPLC	CAA35393	UL34
YTPGLLDHHNVCDVE	CAA35369	UL95
DFRALVSPDRLVVGY	CAA35427	UL28
NTDFRVLELYSQKEL	CAA35414	UL55(gB)
LVLSRLPRSRFQRFW	CAA35387	UL72
RCGAYVATALYYVHF	CAA35282	US15
FNQLVFTAQLRHYCE	CAA35393	UL34
ETRGDLFSGDEDSDS	CAA35431	UL32(pp150)
TTTTRTTTTTTTKKT	CAA35444	UL11
TKTRSFLFILIVNNN	CAA35453	TRL4
PPLPTQQPEEQAKED	CAA35426	UL27
VMYKHTFKEPACTVS	CAA35361	UL87
PVQRRELSRFRKHVH	CAA35426	UL27
YVILTPLAVLTCGLQ	CAA35419	UL20
SVLGPISGHVLKAVF	CAA35357	UL83(pp65)
YQWWKPDTTSCIQKT	CAA35442	UL9
TDAYKETTRQGGAAF	CAA35459	TRL11

TSLDTNSKNYNQHAK	CAA35432*	UL33
RNQTILQRMPQTASK	CAA35332	UL130(viralentry)
SYDNIPPTSSSDEGE	CAA35335	UL99(pp28)
FVENKTASDSNKTTP	CAA35389	UL74(gO)
EVDCASLMETLKRIR	CAA35418	UL19
NKHTQHKRSTRRTSP	CAA35443	UL10
ASPEAEARYPRLTTY	CAA35394	UL35
TAYKAFLWKYAKRLN	AAA85886.1	UL147Toledo
TPEQSTPSRIRKAKL	CAA35431	UL32(pp150)
PGSPTDSARHMSDLA	CAA35423	UL24
SFGEIVAAAYDDSKF	CAA35341	UL104
ISLRTEISKDADPIS	CAA35342	UL106
VCYFSTSVRDVAEAV	CAA35351	UL78
RGRASCMLAVDPYDT	CAA35360	UL86(MCP=majorcapsidprotein)
LETSAADFEQLRRLC	CAA35411	UL52
SWENGTAPDGEPGVC	CAA35265	US34
NGNILFSLGTLYGHR	CAA35352	UL79
QIAWRRHPRCFDLHR	CAA35328	UL126
DAHTCEYLIDRRRHL	CAA35316	UL114
KNGHVRLFFSSLFEY	CAA35443	UL10
LAERLRLCRHMDPEQ	CAA35430	UL31
LSAINTTIARHEFSR	CAA35329	UL127
PDPERIDFRDLPTVY	CAA35334	UL98
TVTLQTISLSTNTTT	AAA85896.1	UL154Towne
DLLTLCLYENLVYLY	CAA35284	US17
YVRRVANTITEFFRM	CAA35291	US24
YVYTTSILIAVVTSV	CAA35374	UL59
AALQNLPQCSPDEIM	CAA35325	UL123(pp72=MIprotein=IE1)
PKCRNNSAPLTQMGG	CAA35328	UL126
MEPVNKAVFMDAHGG	CAA35427	UL28
LQPLDDNAKQELFRL	CAA35407	UL48(pp212)
ARDPGPEPGEERGGG	CAA35376	UL61
PLAFRLSDARTLGLD	CAA35311	IRS1
LAARALHMPTLANDV	CAA35352	UL79
TLETAFSVLISALVW	CAA35281	US14
QQKMEEQLQETRQQM	CAA35407	UL48(pp212)
ACVAGFTSGGGVVSW	CAA35404	UL45
KAYKGTVRAEGKKKL	CAA35393	UL34
VERVLAKCVRARDFN	CAA35338	UL102
SLLDLTVFAGTTTTT	CAA35384	UL69
GFMFQRSAKSRWFDV	CAA35266	US33
PYCRCKGKLGLRIIT	CAA35386	UL70
GGAGGGGGRDVSGGP	CAA35372	UL57
LITKPEVISVMKRRI	CAA35325	UL123(pp72=MIprotein=IE1)

TSERTVEKVTKMAPV	CAA35377	UL62
PALSSIVPVSTLAML	CAA35285	US18
TFLATTTTTTMMGVA	CAA35358	UL84
KARDHLAVLDRTEFD	CAA35431	UL32(pp150)
MDTIIHNSTRNNTPP	CAA35432*	UL33
FSGRREAIVRLEKTP	CAA35395	UL36
DLECAKQYWQELNDH	CAA35293	US26
NCQISSQTGSFVGML	CAA35340	UL105
PVVWPPGWNLVLQEI	CAA35423	UL24
KEKYEQHKITSYLTS	CAA35403	UL44(pp50)
DLARNGNILFSLGTL	CAA35352	UL79
GQRVTKVCTDYTSVT	AAA85883.1	UL144Toledo
PAPPADIDTGMSPWA	AAA85880.1	UL141Toledo
TRPMMSLAHINTVSC	CAA35385	UL71
HLVTDYGNVAFKYLA	CAA35407	UL48(pp212)
IEKKKRARLQRGAAS	CAA35384	UL69
RACAPGPLVHPSLYI	CAA35375	UL60
GRRMGGSSGGRGGTP	CAA35376	UL61
LLGSPFRQRVSAFVA	CAA35386	UL70
RCQQSCHGHFVRRLV	CAA35351	UL78
ATAAAPAWSGGENPI	CAA35340	UL105
LLDHNSLASVPCTLL	P09704	US28
TDAMFEAGNVPSALL	CAA35425	UL26
VYCVVNNRAYQELCE	CAA35340	UL105
GGEEGEDDVLATIRN	AAA85891.1	UL150Toledo
VQISVEDAKIFGAHM	CAA35332	UL130(viralentry)
ASLLGDLHRFLFGVD	CAA35408	UL49
FHHSRLCLTESSPSL	CAA35282	US15
CTDADDSWKQLGEDF	CAA35273	US6
NTLTVACPPREPPHR	CAA35445	UL13
DLVKVEEPVSRMIVC	CAA35368	UL94
APYQRDNFILRQTEK	CAA35390	UL75(gH)
KEDHDDGDERLFRDP	CAA35426	UL27
VTCNSTTTNTASITS	CAA35460	TRL12
VLENHVATHVLRGLL	CAA35361	UL87
DQTDAVLFFDSPENV	CAA35413	UL54
AVFGVHPETRQAHFL	CAA74075	UL43rev
MTVTFYGTRYIRDEL	CAA35280	US13
MSLLEREESWRRVVD	CAA35263	US31
SSPSTGSGTPRVTSP	P19893	UL122(IE2)
VVPRAVQSTTTVMTP	CAA35439	UL6
WRFEDGGAAQRLSLS	CAA35447	UL14
IKGSSVGVNSKVRAC	CAA35340	UL105
AFTTNRKASGTGVAA	CAA35363	UL89

SSSSAPLPPPPPPSG	CAA35315	UL113
TPRVTSPTHPLSQMN	P19893	UL122(IE2)
HLLPLLLCRLPFLLL	CAA35445	UL13
LHPTSSPCLALLRAK	CAA35408	UL49
ESFSALTVSEHVSYV	CAA35390	UL75(gH)
SLYLRGQPKFSSIWR	CAA35427	UL28
ETAATEARPYVNCRA	CAA35406	UL47
DSLTFVAESITEFLN	CAA35422	UL23
LARRRDWSYKRLEEP	CAA35320	UL118
DSNKTTPTSPSMGFQ	CAA35389	UL74(gO)
ATGAQPSHAPAQRVL	CAA35397	UL38
AFIRRRRPPHHTQLV	CAA35310	J1I
EWLVCRQAEREAVTA	CAA35408	UL49
TPQVPRCTSLYIGED	AAA85874.1	UL135Toledo
CGMSSRLERAVKRLQ	CAA35293	US26
RDVCIDYGLHRVFTQ	CAA35361	UL87
AYRRRRESSCAVLVH	AAA85880.1	UL141Toledo
GGRGGTPGRGPERAA	CAA35376	UL61
PSRKLKRKKNGHVRL	CAA35443	UL10
LMSTTCRCWSSSIVL	CAA35380	UL65
AVFDVLSRENLERRG	CAA35406	UL47
LTVKPKPLETTTASN	CAA35440	UL7
GFVEGPGFMRYQLIV	CAA35395	UL36
ENSDQEESEQSDEEE	CAA35325	UL123(pp72=MIprotein=IE1)
RRSMPAPNGPLCALL	CAA35415	UL15
NRLLGGLFPPELQKY	CAA35391	UL76
LSFPITRRIQSRRFP	CAA35453	TRL4
TVIAAQNLSAILNRT	CAA35340	UL105
PSASRRLFGSSADED	CAA35431	UL32(pp150)
FVALQTPYVSLMIFN	CAA35432*	UL33
QGFPVYEVRVDPLTR	CAA35413	UL54
TALNTPSRTHHAAPH	CAA35310	J1I
FGRPNAAPETHRLVA	CAA35414	UL55(gB)
SRAKRRRRRRAPKND	CAA35369	UL95
GGLFPPELQKYRRRL	CAA35391	UL76
DLKYILTRLEYLYKV	CAA35413	UL54
IPNDVSESFERYKEL	CAA35412	UL53
DLFETGAARSFFFPG	CAA35338	UL102
AVRRTTCGKRVASQS	AAA85890.1	UL149Toledo
KEQMALKVTCNAFYG	CAA35413	UL54
GRRPVGFCFIIFFYF	CAA35453	TRL4
TAARPEPCRGLRRGA	CAA35376	UL61
EAFNTILGFLAQNTT	CAA35363	UL89
MFDEDDDDELSLPRN	CAA35395	UL36

RFIVQYLNTLLITMM	AAA85887.1	UL148Toledo
SPYLSSVWVPMSVLA	AAA85873.1	UL134Toledo
GTHVNPWVCEEPKHE	AAA85896.1	UL154Towne
CDGPPGSPTDSARHM	CAA35423	UL24
KFRQELHCLLAEFRQ	P09704	US28
ERQRLRFHLSGLGCP	CAA35262	US30
RYIRVGDNLNTFMCL	CAA35423	UL24
NVPSVLMKRYRAREQ	CAA35404	UL45
RTGYRWAPSSWWRAR	CAA35409	UL50
CFKENYFTGHHEDEN	CAA35460	TRL12
DRSSLYEANPELRLP	CAA35428	UL29
LPSAVPLPPPPAPPP	CAA35453	TRL4
YSVWCGMSSRLERAV	CAA35293	US26
QQRQLLDGYQKKVQQ	CAA35407	UL48(pp212)
VLASTYTWLHKTLLC	CAA35281	US14
ACEALKKALRRHRFL	CAA35396	UL37
TLHNLKLFCYLVSTA	CAA35407	UL48(pp212)
QQTRHTCLQLVARFF	CAA35339	UL103
LSLGIPHNWFLQVRP	CAA35290	US23
LRLLACPDRPIIGDT	CAA35424	UL25
TTKLTTVATTSATTT	CAA35388	UL73(gN)
LRVGKGRCTDIYALD	AAA85873.1	UL134Toledo
TMRVRVLLQEHEHCL	CAA35320	UL118
SPYQRLETRDWDEEE	CAA35295	UL132
ASTCDIEVDCDVSDL	CAA35413	UL54
FDDYGNTKSYLGAYT	CAA35451	TRL3
SEPLRATTPSGATSV	CAA35393	UL34
DRGGGGGSGTRKMSS	CAA35403	UL44(pp50)
HGNWTVEIPISVTSP	CAA35417	UL18
NFIETRSLNVTRYRR	CAA35386	UL70
STKDTSLQAPPSYEE	CAA35414	UL55(gB)
NPANWPRERAWALKN	CAA35431	UL32(pp150)
AVHVLELEDYDRRCR	AAA85886.1	UL147Toledo
QVDLTETLERYQQRL	CAA35390	UL75(gH)
LLFWHDLCWLFRRLF	CAA35416	UL17
MSRVTDPEHLMMNVN	CAA35340	UL105
RSPTYVNLTPPEHRR	CAA35389	UL74(gO)
RLLSVRVRVRQISSD	CAA35406	UL47
VKIDEVSRMFRNTNR	P19893	UL122(IE2)
FAVNNETLQLSRYLA	CAA35386	UL70
SPLLLVFAPSLLWCL	CAA35280	US13
LGGRSGNFPPPRPMP	CAA35376	UL61
IPGTSGLLPSCEEDE	CAA35447	UL14
SCDTRIILRGKTLLL	CAA35436	UL3

SPFYNGTNRNASYFG	CAA35414	UL55(gB)
WVTGFVLLGLLTLFA	CAA35438	UL5
SSKSATCALGLTLQS	CAA35430	UL31
NEDTPADEEAEDSVF	CAA35424	UL25
VLPRVHGPRSSSEDE	CAA35293	US26
ASNVTEKASYVQGCI	CAA35430	UL31
ILGSFGRRYNVDAKD	CAA35372	UL57
LVTHCLNTRSRTYVA	CAA35439	UL6
NLPQCSPDEIMAYAQ	CAA35325	UL123(pp72=MIprotein=IE1)
LVRGLELARQHSSPA	CAA35394	UL35
SVPVSQRMEHGQEET	CAA35340	UL105
TVNPLIEDVMRSSAG	CAA35406	UL47
YISFHDACILVPAKV	CAA35444	UL11
RVFGFVSKHVPLADS	CAA35340	UL105
DSKPDTIRRCFLNHT	CAA35442	UL9
VRVGLALLIDDFRYE	CAA35428	UL29
ASPWSTLTANQNPSP	CAA35332	UL130(viralentry)
MTLRTFLQTYFSSDK	CAA35282	US15
SALLEYDDTQGVINI	CAA35390	UL75(gH)
EHTGVSSVTLLKIFS	CAA35424	UL25
QQGEDAVVRRCLREY	CAA35385	UL71
GCLSDVLYNTRHRER	CAA35360	UL86(MCP=majorcapsidprotein)
ARHAAPVSADWFRFS	CAA35364	UL90
SRTCLLCALYSQNRL	CAA35369	UL95
NCQDLLSDLRLLELP	CAA35283	US16
AYDSLMVIFFCPPNQ	P09724	US20
PRNYSATLTLASRDC	CAA35277	US10
KAGHPEGLCAQDGLY	CAA35311	IRS1
PGTSVLWAPDVVITT	CAA35362	UL88
VRNNKRATCWVVIFW	CAA35259	US27
PIFQIYYLLHAANHD	CAA35412	UL53
CSSSSVLSSALPSVT	CAA35338	UL102
QRPQWAHGLDIVEED	CAA35445	UL13
GCDLLREVQRNLTRT	CAA35284	US17
WFISVFGHTEGQVLL	CAA35386	UL70
HRGELNLMTPSPSHG	CAA35384	UL69
VQVSGPRENAVSPAT	AAA85875.1	UL136Toledo
WHQNQRDFPKCRNNS	CAA35328	UL126
PFRSDSYGLLGNSVD	CAA35353	UL80
TGLTPKRFMELLDRA	CAA35394	UL35
MCRYTPRLDEIHKNT	CAA35444	UL11
DAEIVERALVSAVIL	CAA35431	UL32(pp150)
THPVVKGGVRNQNDN	CAA35417	UL18
TLLNCAVTKLPCTLR	CAA35403	UL44(pp50)

KRSWDTTVYHRRRKH	CAA35416	UL17
IKTAFGMRVLGLGTV	CAA35423	UL24
MLFFIWAMFTTCRAV	CAA35336	UL100(gM)
GLAKHPSVSADEELS	CAA35448	UL16
SENTTTVMSTLTKYA	CAA35396	UL37
IAADRYRVLHKRTYA	CAA35432*	UL33
YRSELLCTSAFLGYS	CAA35339	UL103
LGGNVCRFQVVVTMM	CAA35383	UL68
VGPDVDVYEFPSEYE	CAA35413	UL54
PLHPDAQHTLPLHHS	CAA35449	TRL1
DGRKPPGPGVCLSPD	AAA85885.1	UL146Toledo
ALLVVAILGIIFLAV	CAA74074	UL42rev
IRWWQHNSKKCNQTE	CAA35296	IRL14
RGPSLAHYVTACLFV	CAA35413	UL54
MQLCSHSISSQRHVA	AAA85891.1	UL150Toledo
HLCPHHHERAVDHKR	CAA35291	US24
DCLHTVAVRTMAFLR	CAA35408	UL49
CTDSVLDYLGRRQDE	CAA35391	UL76
LKLSTNQPPIFQIYY	CAA35412	UL53
CDLRLHDNSISELQR	CAA35293	US26
LKPYKTAIQQLRSVI	CAA35403	UL44(pp50)
RTAIGGLHSNYTNLT	CAA35419	UL20
ITLTFTAIVVVILRR	CAA35282	US15
YKGKVHLADFMRDFT	CAA35411	UL52
MNPADADEEQRVSSV	CAA35404	UL45
FRTPNLWLPTTDVQG	AAA85892.1	UL151Toledo
PSSVVPASVESELSS	CAA35397	UL38
FLQDGVSFIIYKFTY	CAA35327	UL125
QELAQYEPFASAPHP	CAA35311	IRS1
IQPASQKSSLFVSEI	CAA35406	UL47
PMPLQKLLICDPHAR	CAA35333	UL97
ELKPQYAETYASVSE	CAA35407	UL48(pp212)
SITEQYNTASNVTEK	CAA35430	UL31
RLLIMGLFSLDRSYE	CAA35359	UL85
NSTSWQIPKLMKVAM	CAA35295	UL132
GDAKYGIRNQHLSIR	CAA35262	US30
SQEPMSIYVYALPLK	CAA35357	UL83(pp65)
HVRRFRPHEVGGHAT	CAA35426	UL27
KDRRRGVFRAKPYSF	CAA35421	UL22
AAPERDIRDLFKKQV	CAA35341	UL104
ENVGLYVAYVVNDGE	AAA85887.1	UL148Toledo
FVGTKFRQELHCLLA	P09704	US28
GIPKNIISWSSFFDV	CAA35344	UL108
PLPSPLLYECHNSTL	CAA35443	UL10

VASLETAVSTPFFEI	CAA35367	UL93
NVLFDADLIKNRVML	CAA35372	UL57
YVAAEPLAYERDKLL	CAA35341	UL104
TVRRVAAQVNNQSRS	CAA35424	UL25
LGHTTPSSDYNNDVI	CAA35431	UL32(pp150)
MLSSASPSPAKSAPP	CAA35431	UL32(pp150)
KRLCDLPLVSSRLLP	CAA35315	UL113
GLKMPVTVWLPRSWL	CAA35358	UL84
TGPVTWEKGDALCVL	CAA35359	UL85
GYIKASQELVSYTIK	CAA35363	UL89
EARGLYVPAVSETLF	CAA35372	UL57
FVGKMGTVCSQGAYV	CAA35395	UL36
WRDHALRGRWGTAYS	CAA35263	US31
RGIAAFLGFWSIFTV	AAA85880.1	UL141Toledo
CQKAVTLGGAGAWLL	CAA35368	UL94
MFRNTNRSLEYKNLP	P19893	UL122(IE2)
PTSSPSHSFYIYTCP	CAA35383	UL68
PTPWRLRNHDCGTYR	AAA85880.1	UL141Toledo
RHHELRYKWLIRKDR	CAA35293	US26
DDGKTSRRRRCSLGR	CAA35380	UL65
YSECGDGSPAVYTCV	P16832	UL115(gL)
IYYLVCWIKISMRKD	CAA35336	UL100(gM)
NNECVGEFANETSGW	CAA35259	US27
SSQRHVASSMHCRSR	AAA85891.1	UL150Toledo
RSPPLQRLAGEIYRL	CAA35290	US23
FLRTFRNQQVNKRLT	CAA35455	TRL7
KIRTLKLCVFIFPSP	CAA35373	UL58
LWRGVLTTIEVSWRP	CAA35376	UL61
IPNQGRSLNKRLQGL	CAA35371	UL56
FDNCNYTINSTKHII	CAA35434	UL1
RLYPKPRYDYTLASA	CAA35374	UL59
SCHFGECDMPVQRLT	CAA35404	UL45
SADLEQPPRQRRRMV	CAA35394	UL35
GVLKRALVRLHRVRD	CAA35394	UL35
VLRSGGRLEALWTLR	CAA35278	US11
CPRYLVPLRALGLHD	CAA35408	UL49
TWLAGLIFSVPAAVY	CAA35432*	UL33
PVSATPRHRRRPERS	CAA35310	J1I
PGRGTVLRLSLQEFC	CAA35407	UL48(pp212)
LYALHGFRSDNNTAY	CAA35267	US35
TVVHEVDPAADPTVG	CAA35311	IRS1
LAVLFQDVRYIATKM	CAA35281	US14
MGGGGNGRGSGEERR	CAA35456	TRL8
RLPQFSSALSDAELS	CAA35426	UL27

QTLRTFWPQISQYDL	CAA35407	UL48(pp212)
RAAALALHFLTSRKG	CAA35424	UL25
HRRRKHLAVQRYAPC	CAA35361	UL87
TITHNLTITSCYKTA	AAA85896.1	UL154Towne
GLSTFNPAGATRMEL	CAA35404	UL45
PTEISEATHPVLATM	Q7M6N6	UL48A
TAESLRLAPYPADLK	CAA35384	UL69
MGAELCKRICCEFGT	CAA35335	UL99(pp28)
RLGEKTVIMENLRRV	CAA35360	UL86(MCP=majorcapsidprotein)
AAVAGVPVAANPVMP	CAA35319	UL117
AVHYAAGNVEAQHDT	CAA35442	UL9
FTQLELRNSYQIPFL	CAA35361	UL87
FIQLRSRSALGPFVG	CAA35395	UL36
GYHPGQKVHWYNQSC	CAA35443	UL10
QRGDPFDKNYVGNSG	CAA35403	UL44(pp50)
VPFMHAEDGRLHMHV	CAA35412	UL53
PSLFCDCPGGGGASG	CAA35361	UL87
IDLLLQRGPQYSEHP	CAA35357	UL83(pp65)
RPVLAQRNRHPRCRR	CAA35283	US16
LNFVNAGQKYAAFLK	CAA35410	UL51
RKSASSATACTSGVM	CAA35357	UL83(pp65)
ATLVEHAEGMASEMC	CAA35351	UL78
ESASSSGSIILAEKS	CAA35387	UL72
LDVTTPDAKALMEKP	CAA35407	UL48(pp212)
PPLPPSSRHALGGTG	CAA35384	UL69
ALAADIGYGVYVDKA	CAA35338	UL102
IYGYGTRPVPDLQCV	CAA35413	UL54
LALSTPFLMEHTMPV	P19893	UL122(IE2)
LRRHERLHRHDRRGL	CAA35400	UL41
YNARLTRGYVRYTLS	AAA85887.1	UL148Toledo
EHRHMTAHIIARNTN	CAA35267	US35
METKLKGGAGAFATS	CAA35360	UL86(MCP=majorcapsidprotein)
STSSRGSAASGDGYH	CAA35333	UL97
VKSTTGMKTVAFDLS	CAA35431	UL32(pp150)
ESLFDGPRRRDRQAA	AAA85891.1	UL150Toledo
HINTVSCPTVMRFDQ	CAA35385	UL71
GSNTTPSKNVTLSQG	P16845	UL22A
HEPRVVTHTAERVSE	CAA35338	UL102
RPLAWRRRCSGGGLK	CAA35407	UL48(pp212)
TSESINCSTATAPAT	CAA35461	TRL13
HYPEKCRRHHERQRR	AAA85875.1	UL136Toledo
CRRPSPGRRDVRRER	CAA35381	UL66
EVIHDALADDEEQGE	CAA35290	US23
RRCARPRGVRLRRNE	CAA35310	J1I

PRRRAPCGPQRPAEI	CAA35416	UL17
NTDATSRLTRVPIFS	CAA35422	UL23
NTQGCGYKYDWSNVV	CAA35430	UL31
MDTVSATKFYEAFVS	CAA35334	UL98
SHLSLVVPVGCLFLL	CAA35286	US19
LLPTLDGTFHQGCYV	CAA35417	UL18
IPLTLVDALEQLACS	CAA35356	UL82(pp71)
LPDVSSLCAAAAATA	CAA35340	UL105
WPPRLPHLFRTPNLW	AAA85892.1	UL151Toledo
PDHRAELCRRSRASA	CAA35428	UL29
SSRHSGKCRRQRRAL	CAA35358	UL84
IRYIPATQGDVYHGR	CAA35436	UL3
YAYDTREKYMVLVSH	CAA35291	US24
WSEADLLTLCLYENL	CAA35284	US17
LSILSTMQPSTLETF	CAA35390	UL75(gH)
AQNRTQSSLLTLYLE	CAA35371	UL56
DSFVCLRPVDFQRLT	CAA35407	UL48(pp212)
AVQSVMKDAESMQMT	AAA85874.1	UL135Toledo
YHWHDTFKLVRMFLS	AAA85877.1	UL138Toledo
QTITFRDATFTIPDP	CAA35359	UL85
NLMHCVKLVTTDSPL	CAA35395	UL36
KNDAATPSFLRRHDV	CAA35369	UL95
YVLFRRDTDTELDTV	CAA35406	UL47
INSLNVNIRGSYPEF	CAA35361	UL87
RLLRVFPSFVALNRL	CAA35391	UL76
LISRTTTTRKPGQKT	CAA35443	UL10
FALLHGIQTFSYGLD	CAA35407	UL48(pp212)
PNTNPQDSAHFPTEN	CAA35449	TRL1
ATAPIGISSLILTWT	CAA35281	US14
WERLDPNTLVLHRYD	CAA35291	US24
TDLYYYRPTAVVVMN	CAA35393	UL34
VNITKSPGPRRINIT	AAA85885.1	UL146Toledo
LIAAYRSERCQQCCS	AAA85883.1	UL144Toledo
SVITVHRFRPTADAD	CAA35393	UL34
LESEDGAPLCRGCAL	CAA35408	UL49
CVSSSSSVPSVPTSV	CAA35361	UL87
AFEDNPVFNKLHESN	CAA35371	UL56
CRRPHHLTYRHTAYT	CAA35460	TRL12
VSESFERYKELIQEL	CAA35412	UL53
GLNERDGFRQRLLLP	AAA85891.1	UL150Toledo
IARFGARAGNQCVCN	CAA35407	UL48(pp212)
TVANKRDEKHRHVVN	Q7M6N6	UL48A
RNGFTVLCPKNMIIK	CAA35357	UL83(pp65)
VARTAAAVSAAGVGP	CAA35315	UL113

AASGTETEAAGGDAP	CAA35311	IRS1
LSPVFPGGETARKDK	CAA35413	UL54
LRRLEVHDALLFHYE	CAA35392	UL77
GLYLALGAGFRVFVY	CAA35311	IRS1
SGNAYNHTIDTCKNT	CAA35419	UL20
DARQKYALEAYMPEA	CAA35372	UL57
PVFVHEQQYLRSGLT	CAA35392	UL77
RHDASENAVRRRHER	CAA35445	UL13
HQDPNRVLDCIRPVV	CAA35319	UL117
LIIRPTIWLPGTAAG	CAA35387	UL72
RVCDRVLEDHLHTPR	CAA35406	UL47
GKFSFTCANHLILQI	CAA35352	UL79
TSPRDYNVTGLPKGF	CAA35443	UL10
LLCSITYESTGRGIR	CAA35273	US6
WLEQHQEEGKATQCL	CAA35404	UL45
FPTCFTLSLISRTTT	CAA35443	UL10
RTKLVLFYFSPCHQC	CAA35278	US11
IRKPPWLMEQPPPPS	CAA35311	IRS1
IRHLVRSYADMNISL	CAA35293	US26
EDGETVAAVSAFACP	CAA35385	UL71
GIILLILYLIAAYRS	AAA85883.1	UL144Toledo
AAAAATAAAPAWSGG	CAA35340	UL105
YRLPAQDVVTSWIEA	CAA35430	UL31
FSGPSVPWRDEKRAC	CAA35381	UL66
LLHPSPAPMPPATHG	CAA35327	UL125
VNETFRCSVTSDARK	CAA74075	UL43rev
AVVGCSVFMIFLCAY	AAA85872.1	UL133Toledo
HHDANDTNNTNGHAT	CAA35432*	UL33
WLTIILYVFMWTYLV	CAA35274	US7
VLRIMTLRTFLQTYF	CAA35282	US15
THLWPQELQALCDSP	CAA35395	UL36
HVLVRLFHKRGVIQH	CAA35392	UL77
LIVLIGQRGGIYCYD	CAA35395	UL36
AQPVTPRTREGAGVR	CAA35343	UL107
RELSRFRKHVHDLKR	CAA35426	UL27
LVYKRRVGAPQRLCA	P09724	US20
GASPAVSSLAWPHDG	CAA35353	UL80
RLRMSNIPRSSARLL	CAA35362	UL88
WKLVGKSRKHREYRA	CAA35368	UL94
LGHKLFLGYYAMALS	CAA35284	US17
GAACRALENGKLTHC	CAA35333	UL97
AQRNRHPRCRRPFRQ	CAA35283	US16
WRPPKTSRPWKPGQR	CAA35422	UL23
HDLRHSDRSCDLAFG	CAA35447	UL14

ICVSQKILRCLKTGE	CAA35378	UL63
AYTNAAFTSSDATLP	AAA85878.1	UL139Toledo
QFHQGEPLGHKKEKP	CAA35279	US12
CVPGVSRACVGCVGG	CAA35310	J1I
FLDGLSYDDPPNEQT	CAA35407	UL48(pp212)
TAGAGKTSSIQVLAA	CAA35340	UL105
LKRDLFAARQSSTLL	CAA35354	UL80A
VSKDLASYRSFSQQL	CAA35390	UL75(gH)
LRASVLVAFQLTATV	CAA35286	US19
CHLGSSVFSRPRAAK	CAA35452	TRL5
DVYSVTLENGVTHRF	CAA35413	UL54
PAPCLQTFTERGPPS	CAA35360	UL86(MCP=majorcapsidprotein)
AVFQETGTARRIPNC	CAA35333	UL97
PGAEHMRDVSYKLFV	CAA35406	UL47
AVEAVWQRLEPGRVA	CAA35407	UL48(pp212)
SLSDFTYWSRTSEVI	CAA35371	UL56
SDARERGEFGDEDEE	CAA35394	UL35
RHPAPKRRGKCSDFS	CAA35292	US25
FQALKSALLKLHNVT	CAA35319	UL117
SQLESALDWFLRPTR	CAA35289	US22
TLASRDCYERFVCPV	CAA35277	US10
EFKTTYSDADDQSVR	CAA35391	UL76
QYATACAVAAATWPP	AAA85892.1	UL151Toledo
NSQESAAPQPPRSPR	CAA35315	UL113
RAHRVGTVIVEGRTD	CAA35377	UL62
HHHERAVDHKRDILF	CAA35291	US24
VHTVLLSWKVLLTMV	CAA35432*	UL33
TTPPMIDLTSHHRPL	CAA35319	UL117
SLVVDARSGQVLHND	CAA35447	UL14
ILVPAKVGTNVTLNA	CAA35444	UL11
PSKTTTQISTTTNTN	CAA35461	TRL13
EAEWRQTQMDVGGLI	CAA35397	UL38
PDEQARVFCLSADWI	CAA35316	UL114
MPQTASKPSDGNVQI	CAA35332	UL130(viralentry)
SLLTAVRRHLNQRLC	CAA35311	IRS1
YSDPLKTRLLRGLIR	CAA35394	UL35
EDAIRCDYGVFQFRN	CAA35289	US22
FVIGVLEQAHFVVIG	CAA35427	UL28
SEYRVEYSEARCVLR	CAA35278	US11
ASQLKTADSPTLFLL	CAA35456	TRL8
QEEAGKLTVTSVEDV	CAA35407	UL48(pp212)
IRLEPFQKNLLIHVI	CAA35363	UL89
VTHVTLLQIFRLRSS	AAA85891.1	UL150Toledo
TIYMTNLYSTNFLTL	CAA35432*	UL33

HPICTYDPPKPGRRK	CAA35443	UL10
ERREKRSGASEGEDG	CAA35456	TRL8
TFVTLVRGLELARQH	CAA35394	UL35
RYVELICSREKARRR	CAA35341	UL104
NKAVFMDAHGGIHVL	CAA35427	UL28
LKESPSRKLKRKKNG	CAA35443	UL10
PCFVVDAVVHPLALD	CAA35282	US15
DGLGGGRGGGGGGDS	CAA35372	UL57
DHSSLSPSAVASHHS	CAA35426	UL27
TTVVRKYWTFANPNR	CAA35315	UL113
HVLRGLLSLTEWNRE	CAA35361	UL87
TTTTRKPGQKTTLSR	CAA35443	UL10
NVSDPKNYCRRKCPP	CAA35443	UL10
TKEERDISTTYHVTQ	CAA35360	UL86(MCP=majorcapsidprotein)
SPPSCSSSSATWLEE	CAA35338	UL102
LTVFAGTTTTTASNH	CAA35384	UL69
ACLLKLHGREKRLQL	CAA35361	UL87
VFFIVVVAGFASSEA	CAA35349	UL111
SSCTVGFATVALIAA	CAA35432*	UL33
YSTALFFLFLILDRL	CAA35351	UL78
PSHIHTMIFSPAWNL	AAA85873.1	UL134Toledo
NEPMGLGGLGGGGGG	CAA35403	UL44(pp50)
RAEIAEALERVAERC	CAA35334	UL98
PATSTASHHPHASPR	CAA35449	TRL1
SGPPLPVLPVDDWLN	CAA35394	UL35
PCMISHTNNVNVGWF	CAA35361	UL87
PFCALTEVESRRWWW	CAA35291	US24
KNTPFCTPNVQTRRG	P19893	UL122(IE2)
ALEDLLPMRLASPET	CAA35358	UL84
RCRLQPSLREPPTPA	CAA35393	UL34
LQSVRSLMKRTHRAS	CAA35430	UL31
LLRKLVVEKIFAVYN	CAA35384	UL69
SGNFPPPRPMPGTGL	CAA35376	UL61
KATQIVEMTHKTPSA	AAA85891.1	UL150Toledo
HSQRNNGGGGRARGG	CAA35371	UL56
GEDGLPIDKPEFPPA	AAA85874.1	UL135Toledo
PRNVMTHEEAESRLY	CAA35395	UL36
EFMDYVILTPLAVLT	CAA35419	UL20
SVQDTIQHMRFLYLL	CAA35407	UL48(pp212)
PHRLEQLFSSVRLTL	CAA35288	US21
NVTVLAGQQLITQTM	CAA35319	UL117
ESAPATAEVCLGDAL	CAA35319	UL117
ILLHVPTHGLLYTVL	CAA35359	UL85
AYSAWGAGSFVATLI	CAA35458	TRL10

KWDVFAYDSGILFFL	CAA35397	UL38
EAESRLYDAITENLM	CAA35395	UL36
GKKHDRGGGGGSGTR	CAA35403	UL44(pp50)
LSAERTVRWMLAFLE	CAA35407	UL48(pp212)
RSCIIKALTPRLSRG	CAA35367	UL93
PMFFNVPRWNTKLYV	CAA35389	UL74(gO)
FFPKLQGNYNEQHYR	CAA35437	UL4(gp48)
GGGGGKKHDRGGGGG	CAA35403	UL44(pp50)
LRRLVTHQGAEEAIV	CAA35341	UL104
VPEAYLQLSFGEIVA	CAA35341	UL104
QHQFLRRTYGPQHRL	CAA35370	UL96
VRLEKTPTCQHPKKT	CAA35395	UL36
CNTKLLLPVALIPVV	CAA35434	UL1
PSTVNSTATGVTSDS	CAA35326	UL124
RGLQNKTEDFLHWLL	CAA35437	UL4(gp48)

Peptides recognized at 12 month after HSCT. 4 / 4 patients, light blue, 3 / 4 patients dark blue; 2 / 4 patients light yellow and 1 / 4 patients, green.

Peptide	UL-ORF	
FRVEENQCWFHMGML	CAA35314	US3
VFPCFVPEPWQLMNL	CAA35387	UL72
PFHELRTWEIMEHMR	CAA35360	UL86(MCP=majorcapsidprotein)
HALALWMDWADVRSC	CAA35367	UL93
ACRDWDSMHCTPFWS	AAA85887.1	UL148Toledo
FIALIVVCIMGWWKL	CAA35440	UL7
GWLHRHFPWMFSDQW	CAA35278	US11
AAHDVWCNCGDWQGH	CAA35312	US1
VRATAGRWLPLCWPP	CAA35427	UL28
WCCCMDWKAHVEYAH	CAA35312	US1
GNARLDALMSASEWW	CAA35404	UL45
FFGMCQLWKDWVTNA	CAA35419	UL20
AGRWLPLCWPPLHGI	CAA35427	UL28
CVWCTGLAWLMAWNM	CAA35279	US12
GQNLKYQEFFWDAND	CAA35357	UL83(pp65)
PPDCSPPPYRPPYCL	CAA74074	UL42rev
RSAHFRVEENQCWFH	CAA35314	US3
KNCSRTDVWHDIEWI	AAA85895.1	UL153Towne
QWGRYENGSTPVLWC	CAA35439	UL6
SGYLFFGMCQLWKDW	CAA35419	UL20
HVSRNMSWRVVWELC	CAA35367	UL93
CSFFSWGRHHNATWD	CAA35320	UL118
EPHGQWEFMFREQRG	CAA35311	IRS1
MGCDVHDPSWQCQWG	AAA85872.1	UL133Toledo

WHSRGSTWLYRETCN	CAA35414	UL55(gB)
LMIPKDMYLTWEETR	CAA35405	UL46
MLTPVVWSARWDQMF	CAA35281	US14
YLLSYWESRTDHVPC	CAA35409	UL50
SSFWYHGNCELCGWN	CAA35434	UL1
HDLRGMMDYHDGLSR	CAA35429	UL30
FLRFERYDTDYLLRR	CAA35408	UL49
MGESYFLQDEKCVFW	CAA35374	UL59
INHIVNHDLFRWSVM	CAA35443	UL10
PCNYSSFWYHGNCEL	CAA35434	UL1
NMTSVWRFEGQFNPH	CAA35274	US7
YPAPEHVHRWSYLCP	CAA35316	UL114
LTLMNNCVCDGAVWN	CAA35273	US6
LCRGLRRVWMTVWAS	CAA35386	UL70
VSHETLERYRVCEPH	CAA35340	UL105
LWAREPHGQWEFMFR	CAA35311	IRS1
EAEREYLYRDLHSKW	CAA35406	UL47
SMDTAGMYECVLRNY	AAA85880.1	UL141Toledo
LWLPLLIAWSEWGNC	CAA35447	UL14
LQLPWVPRPSSFMYD	CAA35274	US7
SGFFDLSRWFGENMD	CAA35454	TRL6
DLRSNPYPIRWCYCW	CAA35420	UL21
LAPGPFHELRTWEIM	CAA35360	UL86(MCP=majorcapsidprotein)
TTLPVWTPECKGWTY	CAA35396	UL37
SRLRSINCGWEGERH	CAA35447	UL14
QWEFMFREQRGDPIN	CAA35311	IRS1
RWMTVMRGYSECGDG	P16832	UL115(gL)
RALAGCMHIHAFAQW	CAA35422	UL23
QNYTCRVTHGNWTVE	CAA35417	UL18
RSTSYHETGVYQMWV	CAA35319	UL117
FGWCSVNRYDWRQQG	CAA35413	UL54
FGNNFFVRTGHMVLA	CAA35336	UL100(gM)
WKPACPDEEPDRCWT	AAA85880.1	UL141Toledo
FPMNVYRHDEVDRWI	CAA35360	UL86(MCP=majorcapsidprotein)
FASWDLIERIFEHMY	CAA35404	UL45
YFRIPQKLWLLWQHD	CAA35444	UL11
LEMVYPAPEHVHRWS	CAA35316	UL114
TMRGGGWREDVLMDR	CAA35392	UL77
LDTHYRETMDWRLCG	CAA35316	UL114
EQYHHDERGAYFEWN	CAA35314	US3
ETFSQPMHRAMAYVC	CAA35384	UL69
WRDMLHDLFCGCHYP	AAA85875.1	UL136Toledo
AWRRVADDSHDLWCC	CAA35312	US1
WPDGSYRDWEFLARD	CAA35367	UL93
QPLDGWSWIASPWKG	CAA35431	UL32(pp150)
TAFAHEYHNWLRSPF	CAA35360	UL86(MCP=majorcapsidprotein)

VPYKWMPSSFIVKQC	CAA35387	UL72
FVDDCMRVFAANNQH	CAA35422	UL23
AFYWRLFLQSQHVEA	CAA35434	UL1
DGTRYQMCVMKLESW	CAA35332	UL130(viralentry)
YLETVGGMRQLLFNK	CAA35384	UL69
YHGNCELCGWNGYLR	CAA35434	UL1
FWECRLRVWWLSDAG	AAA85875.1	UL136Toledo
QTEKWHNVDWIHYEY	CAA35433	TRL14
SYVVCSQHGAFFPAR	AAA85892.1	UL151Toledo
GLQEAYILDKGRRYM	CAA35419	UL20
WKAWVGLWTSMGPLI	CAA35313	US2
IRLYDWSEINDWRVM	CAA35422	UL23
SDFDADCWCMWGRFG	CAA35449	TRL1
RDGEWIICFCCNGRY	CAA35368	UL94
HGPALIAWVEEMLRY	CAA35407	UL48(pp212)
FFLAPSMAQFWHGAI	CAA35397	UL38
FVAGHGETDFYMNWT	AAA85887.1	UL148Toledo
GLADWNVVRCRGTGF	CAA35449	TRL1
KFSSIWRGLRDAWTH	CAA35427	UL28
PHAGVIDCAPFHGVW	CAA35338	UL102
QVTIPCTVMTHSWPM	AAA85880.1	UL141Toledo
LCRGDRFRAEMLNNW	CAA35290	US23
MDLPTTVVRKYWTFA	CAA35315	UL113
YVDEIVEGAWFKHTF	CAA35427	UL28
DYTTGVCVMRRLINH	CAA35443	UL10
NAYLDTHYRETMDWR	CAA35316	UL114
LGWAACRDWDSMHCT	AAA85887.1	UL148Toledo
RRDHVWSYVGRVCTF	CAA35351	UL78
NFPLNSMFYYRDRQE	CAA35408	UL49
SFVAGYEGFGWDGET	CAA35417	UL18
YCVEYLLSYWESRTD	CAA35409	UL50
FKPEDVKAWSHYLCC	CAA35423	UL24
HLLCYWCSESYRRLN	CAA35447	UL14
PFDECPDTHFAFWTH	CAA35395	UL36
WDSMHCTPFWSTDLE	AAA85887.1	UL148Toledo
SSMLCVWCTGLAWLM	CAA35279	US12
ERLNMSAYNVMHLHT	CAA35336	UL100(gM)
HGPLCPLVFQGWAYA	CAA35399	UL40
LSLSSFAAWWTMLNA	CAA35388	UL73(gN)
GEVREFKHLVYFHHA	CAA35386	UL70
SEATHPVLATMLSKY	Q7M6N6	UL48A
RVMVGSNHVEPLGWL	CAA35422	UL23
MEDYRTFAGTLSRHP	CAA35352	UL79
NETRRRLSTADWLWW	CAA35456	TRL8
NMRYCVRFASDSDFQ	CAA35387	UL72
CLKQDCDQSWLLEYS	CAA35411	UL52

RIHRFWECRLRVWWL	AAA85875.1	UL136Toledo
VIDCAPFHGVWAEQG	CAA35338	UL102
DRWCLCNAWRDHALR	CAA35263	US31
TTFNPMFFNVPRWNT	CAA35389	UL74(gO)
QRPMVQYDDYWNAVM	CAA35395	UL36
LPLCWPPLHGIMLGD	CAA35427	UL28
LGFADWSQTLIDDYF	CAA35426	UL27
AYYLRWHACVPQKCE	CAA35275	US8
WSLCPPNRECYFPTV	CAA35404	UL45
GLPRCEWFERTIYQE	CAA35404	UL45
PSHVTSAFVPSVYMP	CAA35269	TRS1part
MACSYYDNVVLRELY	CAA35411	UL52
GDIGVFPCFVPEPWQ	CAA35387	UL72
TINWYLQRSMRDDNW	CAA35314	US3
FPLPTAFAHEYHNWL	CAA35360	UL86(MCP=majorcapsidprotein)
NGVAWEHRLSSVWRD	CAA35281	US14
LGTGFHRAEGSFCGC	AAA85892.1	UL151Toledo
ACLLAAYGHALWEGR	CAA35392	UL77
DLTIKMWFLLGAPMI	CAA35351	UL78
YAVQLHAETTRTWRW	CAA35449	TRL1
LWTGPNYEISWLKQN	CAA35417	UL18
VDCNLSMMWMRFFVC	CAA35313	US2
EETILTPRDVEYWKL	CAA35425	UL26
SATWLEERDEWVRSL	CAA35338	UL102
DLSRWFGENMDEYSG	CAA35454	TRL6
LHQGFYLMDELRYVK	CAA35390	UL75(gH)
DTAPRWMTVMRGYSE	P16832	UL115(gL)
SCYIAGGRWRFEDGG	CAA35447	UL14
LCAWTFGLAGPCAAW	CAA35420	UL21
VGEFMVRDPLLRDPR	CAA35424	UL25
CLGGYCDLIREKEVH	CAA35404	UL45
WFGENMDEYSGDVWH	CAA35454	TRL6
IDGGNMTSVWRFEGQ	CAA35274	US7
DEDPETYMGFLWDIP	CAA35395	UL36
DNLTLWTGPNYEISW	CAA35417	UL18
YTNTSCSPQFMCINE	CAA35434	UL1
TDYYRTMTTFVHQSH	CAA35444	UL11
PLFVDDYRATDDEWT	CAA35360	UL86(MCP=majorcapsidprotein)
IDDFRYESIGPVDRS	CAA35428	UL29
VLEQAHFVVIGWMEP	CAA35427	UL28
PAALGGCCCAAGGDW	CAA35338	UL102
WFRWDGNDSHLICFY	CAA35440	UL7
VDCYWRKLFGGDDPG	CAA35338	UL102
CFMAMGESYFLQDEK	CAA35374	UL59
AYVNALHDHRLWPPF	CAA35360	UL86(MCP=majorcapsidprotein)
LCYLQCCGRWCPTPG	AAA85880.1	UL141Toledo

CELCGWNGYLRNVTH	CAA35434	UL1
PVADYMFAQSSKQYG	CAA35392	UL77
DYFLLPAGWACANPR	CAA35426	UL27
LCWHRVEGGISGPRG	CAA35358	UL84
PPDPQHFRWLNAGAF	CAA35426	UL27
SCSPQFMCINETKGL	CAA35434	UL1
ELPSRDGIRWQYQEL	CAA35445	UL13
ELCRHALALWMDWAD	CAA35367	UL93
TVNRTCDLLTPPPWY	CAA35398	UL39
TEHSYALWASLWSRC	CAA35386	UL70
GFLLYRHHERLFPEC	CAA35372	UL57
RCCELYGEYERRFAD	AAA85877.1	UL138Toledo
FFDQFDTNNAMGTYR	CAA35384	UL69
LRLVWPDGSYRDWEF	CAA35367	UL93
CYKETMIYDMVQYGH	AAA85872.1	UL133Toledo
QELQYLVEEQRRRNQ	CAA35445	UL13
REFMLARDLLALWRL	CAA74075	UL43rev
RQELYLMGSLVHSML	CAA35390	UL75(gH)
SSVFETRASGRFFHR	CAA35427	UL28
EEDEWLREIQGATYQ	CAA35445	UL13
LPELDREQWERPRWD	CAA35311	IRS1
TGPRHVIWPGTSVLW	CAA35362	UL88
GEQGCGNFTTFNPMF	CAA35389	UL74(gO)
PPMAAGSWRLCRCEA	CAA35409	UL50
GRCPHENFPFWDRFG	CAA35293	US26
IIVIDECGLMLRYML	CAA35340	UL105
EHCQNDFFGEFRALH	CAA35428	UL29
QKLGWCLADDIHTSF	CAA35405	UL46
LYAAEQLHEQLDRFL	CAA35406	UL47
PATFFCESDDAKYLC	CAA35368	UL94
STCFLTRLNNAPFDM	CAA35363	UL89
AFFTRHWGAEAYEPL	CAA35352	UL79
LYCGPRSFCARDTCF	CAA35369	UL95
SWGRHHNATWDLFTY	CAA35320	UL118
FEGSCVSLGWPSQCI	CAA35428	UL29
HFHTMRDYQRPMVQY	CAA35395	UL36
RWLIRCCELYGEYER	AAA85877.1	UL138Toledo
TQTGMGGGRLPPLWL	CAA35447	UL14
LKDLYAAFCECGDGR	CAA35426	UL27
DSLMEFVTRGMTRCH	CAA35422	UL23
SGLLLLFTCCFCCFW	AAA85878.1	UL139Toledo
DNEIHNPAVFTWPPW	CAA35357	UL83(pp65)
YEPAHLAASDLLNWY	CAA35406	UL47
LDALMSASEWWVESA	CAA35404	UL45
HCTPFWSTDLEQMTD	AAA85887.1	UL148Toledo
DLTYQRLIYWACTLM	CAA35406	UL47

AYVCCQEYLHPFGFV	CAA35395	UL36
KYQEFFWDANDIYRI	CAA35357	UL83(pp65)
GVPLPPDPQHFRWLN	CAA35426	UL27
TMVWGAAPVIMMTWF	CAA35432*	UL33
HVPTQPLDGWSWIAS	CAA35431	UL32(pp150)
PSLKDSCHLCAWTFG	CAA35420	UL21
AVFTTHRFTYLPVGS	CAA35387	UL72
LRVDYTSSAYMWNMQ	CAA35313	US2
HFADYVDPHYPGWGR	CAA35354	UL80A
FERDYVDEIVEGAWF	CAA35427	UL28
FAYDSGILFFLAPSM	CAA35397	UL38
YHECSQTMLHEYVRK	CAA35333	UL97
EKLIQIIYNFYTFFM	CAA35406	UL47
LHDLFCGCHYPEKCR	AAA85875.1	UL136Toledo
CSSSSATWLEERDEW	CAA35338	UL102
STPPLGKLWLYAMAT	CAA35407	UL48(pp212)
GWNGYLRNVTHYYTN	CAA35434	UL1
FSCRDCESSASWLSS	CAA35415	UL15
RTAAHLSQNMRDMYL	CAA35311	IRS1
FQGWAYAVYHQGDMA	CAA35399	UL40
DWIHYEYPTHKMCEL	CAA35433	TRL14
DFLHWLLGWGHKSIC	CAA35437	UL4(gp48)
RTDVWHDIEWIKYGP	AAA85895.1	UL153Towne
EQKKAFYWRLFLQSQ	CAA35434	UL1
SPSRCFMAMGESYFL	CAA35374	UL59
YHLRRDYWLTDPKIG	CAA35447	UL14
CTNTTTTVTCDGFNY	CAA35461	TRL13
ETGIFTFIMEDYRTF	CAA35352	UL79
LNAVHVHDGDYVVYWS	CAA35444	UL11
GIGWYEPEVSMAYIY	CAA35322	UL120
FLPSFSQVTSSMTCD	CAA35368	UL94
AEVCVRTVVDCYWRK	CAA35338	UL102
LDAFLLNWLHHGLDL	CAA35407	UL48(pp212)
QRSYFYCEYSDTDRL	CAA35413	UL54
THSFYLVNAMSRNLF	CAA35389	UL74(gO)
VHDPSWQCQWGVPTI	AAA85872.1	UL133Toledo
WHNVDWIHYEYPTHK	CAA35433	TRL14
TENGSFVAGYEGFGW	CAA35417	UL18
LWYVNSFWRSRELSY	CAA35367	UL93
LMKTVQQLHRIWPFC	CAA35291	US24
LSAFCYAAPATWFHH	CAA35282	US15
HVHDGDYVVYWSFGGG	CAA35444	UL11
QALTELEYQAMGAVW	CAA35311	IRS1
MATLVFPQDMLQCLW	CAA35407	UL48(pp212)
RPPPPPMAAGSWRLC	CAA35409	UL50
YRHHERLFPECGLPC	CAA35372	UL57

CLTERFDPHEGAWER	CAA35291	US24
TMNMTEFPQYYILAG	CAA35389	UL74(gO)
VTPCLHQGFYLMDEL	CAA35390	UL75(gH)
WPNGWFFTYCDLLRV	CAA74075	UL43rev
LDMSSLYYNETMFVE	CAA35389	UL74(gO)
SPSMGFQRTFIDPLW	CAA35389	UL74(gO)
LAPYRFMIAYCPFDE	CAA35384	UL69
MALVDQESCLRGFYS	CAA35374	UL59
CLRGFYSVFLRHCDV	CAA35374	UL59
PVIADIVDKCLNMWE	CAA35340	UL105
HLYTVNCEASYSHDQ	CAA35321	UL119
MPVSDSFVCLRPVDF	CAA35407	UL48(pp212)
LTSIATNSHYTMFVL	CAA35418	UL19
PWQLMNLPPPNEHRF	CAA35387	UL72
NKTEDFLHWLLGWGH	CAA35437	UL4(gp48)
WLLGWGHKSICSFFP	CAA35437	UL4(gp48)
VVSQTATRVRTWFVE	CAA35445	UL13
NVFGQRSYFYCEYSD	CAA35413	UL54
RDISYMGDSLTAFLF	CAA35336	UL100(gM)
NFMTDFKKWLDGGFS	CAA35363	UL89
ASNNPCNYSSFWYHG	CAA35434	UL1
SPYDVINLFVDDCMR	CAA35422	UL23
WHNHGNKWTLDTCYY	CAA35444	UL11
FWFRCPRRFCFSPLD	CAA35427	UL28
LASIHPGETWTLHGM	CAA35448	UL16
NNTTSPWIYAIPMGA	AAA85881.1	UL142Toledo
KGRCTDIYALDFWKR	AAA85873.1	UL134Toledo
ARRRYHLRRDYWLTD	CAA35447	UL14
RTMAFLRFERYDTDY	CAA35408	UL49
PETQFYYTRHEVFNE	CAA35386	UL70
PFTELGWAACRDWDS	AAA85887.1	UL148Toledo
RWQYQELQYLVEEQR	CAA35445	UL13
SVALTSLCHLLCYWC	CAA35447	UL14
EGFTRSGENETFLWY	CAA35440	UL7
SRKTPQPYWPHLYRE	CAA35352	UL79
SCFVHDLRGMMDYHD	CAA35429	UL30
HNDASCYIAGGRWRF	CAA35447	UL14
LRLSWPNGWFFTYCD	CAA74075	UL43rev
ELIMFGVIEAWEEAS	CAA35397	UL38
YRIADNFHMFLKCGL	CAA35289	US22
VWDVQHRVRLRVLWY	CAA35367	UL93
LPFWSTLLPCALRCH	CAA35279	US12
RPFFSDAPLPYFVPP	CAA35358	UL84
WKPSRLPGEDSWYDL	CAA35269	TRS1part
STWDCLSVAWIRHYN	CAA35460	TRL12
GFYQIRKPPWLMEQP	CAA35311	IRS1

IIYIQNSAECWSVRE	CAA35420	UL21
FGYTHPDRHPVYFFK	CAA35386	UL70
PHDPYLETVGGMRQL	CAA35384	UL69
AWLDLDESHWVLGDS	CAA35311	IRS1
VNHDLFRWSVMTAMI	CAA35443	UL10
EADRALREFLEAPWE	CAA35406	UL47
GDYVYWSFGGGGANR	CAA35444	UL11
CPLVFQGWAYAVYHQ	CAA35399	UL40
FDGCYHSEAYRMLFQ	CAA35393	UL34
RFCNNYCFARDCFTH	CAA74075	UL43rev
GAMYLWTDHIYSDSL	CAA35422	UL23
SHDLWCCCMDWKAHV	CAA35312	US1
HPSEQNQNFNLQIHP	AAA85896.1	UL154Towne
AYVLSSMLCVWCTGL	CAA35279	US12
RRLQPMVLLGAWQEL	CAA35311	IRS1
YADNDDYGLYVDWCV	CAA35341	UL104
SVMTAMIFYRYSETC	CAA35443	UL10
YLVERCQQSCHGHFV	CAA35351	UL78
PSPPRYPFLVGWSWG	CAA35453	TRL4
DMFHHDQWKLACIDS	CAA35333	UL97
NLFVFCTERDYRKFH	CAA35393	UL34
SVQHFLWMVRLYGTV	CAA35279	US12
AQWRQQVHAAHDVWC	CAA35312	US1
FWSTDLEQMTDSVRR	AAA85887.1	UL148Toledo
SWWLMPPPVAELCER	CAA35286	US19
QLLFYMWAGTGVMST	CAA35411	UL52
AEAFCTSYGFFPGEI	CAA35417	UL18
AWIASKNVQYEFMGL	CAA35430	UL31
NWTLRRSQTHYLEEM	AAA85887.1	UL148Toledo
IYGTLDMSSLYYNET	CAA35389	UL74(gO)
VMLDYYWIQLITNND	CAA35419	UL20
SDDIVIQISCVCYET	CAA35413	UL54
HDERGAYFEWNIGGH	CAA35314	US3
PHVWMPPQTTPHDWK	CAA35390	UL75(gH)
VVWSARWDQMFSYLA	CAA35281	US14
AGMYECVLRNYSHGL	AAA85880.1	UL141Toledo
RYVTVKDQWHSRGST	CAA35414	UL55(gB)
QYTLNATVEWYNKSE	CAA35419	UL20
IIDETLSYMKYHHLH	CAA35384	UL69
SPSTCSFFWHWCLIA	CAA35276	US9
YVYTSWCQSLRFSEP	CAA35372	UL57
HQDSWRDMLHDLFCG	AAA85875.1	UL136Toledo
YHETGVYQMWVSGAT	CAA35319	UL117
YENGSTPVLWCTLWG	CAA35439	UL6
EDGMVSPERPAFMEH	CAA35456	TRL8
FVPEPWQLMNLPPPN	CAA35387	UL72

HGIMLGDTQYFGVVR	CAA35427	UL28
PMTTGSRVVKYYDGS	CAA35337	UL101
GVCLSPDHLFSKWLD	AAA85885.1	UL146Toledo
PGQMSAWLRDDVCDL	CAA35384	UL69
LGASGAMYLWTDHIY	CAA35422	UL23
FRCRHTFARDYVVEN	CAA35363	UL89
TMLGAYSAWGAGSFV	CAA35458	TRL10
YEPVRDYMTYMNLAE	CAA35369	UL95
TLIDDYFLLPAGWAC	CAA35426	UL27
TCNGSLYTVYKHSNL	AAA85895.1	UL153Towne
WERPRWDALHLHPRA	CAA35311	IRS1
PMVLLGAWQELAQYE	CAA35311	IRS1
WMTVWASLFGYTHPD	CAA35386	UL70
LPYMYKMDQDDGYFM	CAA35386	UL70
EQVSKRSWDTTVYHR	CAA35416	UL17
STLLPCALRCHAYWL	CAA35279	US12
VIQISCVCYETGGNT	CAA35413	UL54
KEKLHCQQNFPLNSM	CAA35408	UL49
STELLDVMQKYFSLD	CAA35386	UL70
MLGIRAMLVMLDYYW	CAA35419	UL20
TEGQVLLTMAYYLFE	CAA35386	UL70
HTPDQNHIEQPFYLM	CAA35363	UL89
CRDDEEFCHQFLRAY	CAA35426	UL27
NTSHHSVVWQRYDIY	CAA35459	TRL11
YLSPERMFFHPGLIS	CAA35406	UL47
YFYIGRADAEDCWKP	AAA85880.1	UL141Toledo
QFTTVAMVHYHQEYT	AAA85877.1	UL138Toledo
LGAWQELAQYEPFAS	CAA35311	IRS1
RSMLLSREEELVPWS	CAA35438	UL5
IGQRGGIYCYDDLRD	CAA35395	UL36
NDQHVFCAVASETWH	CAA35386	UL70
TATRVRTWFVERTTF	CAA35445	UL13
DLAGFFAKGMIRCDP	CAA35428	UL29
AYTSKIGVLVVVCGF	CAA35451	TRL3
VPFVPHACPHYAVPF	CAA35430	UL31
CAEGTVYPSEWMVVK	CAA35371	UL56
TVWRNLFYVYYELAR	CAA35361	UL87
EETVWSLCPPNRECY	CAA35404	UL45
ADFSVSEAWRFEEAV	CAA35369	UL95
HNPAVFTWPPWQAGI	CAA35357	UL83(pp65)
FPRDALLGRLYFISS	CAA35358	UL84
HASYHANYGAYAVLM	AAA85884.1	UL145Toledo
MFPPMYPVLLLTASP	CAA35420	UL21
DSCHLCAWTFGLAGP	CAA35420	UL21
AGVSRLREVWDVQHR	CAA35367	UL93
GLFAVYSFFERDYVD	CAA35427	UL28

TDIYALDFWKRHFLA	AAA85873.1	UL134Toledo
MAAMEANIFCTFDHK	CAA35359	UL85
TGFRAHDSEDGVSVW	CAA35449	TRL1
TDTVYCVEYLLSYWE	CAA35409	UL50
DMLQCLWLELKPQYA	CAA35407	UL48(pp212)
ALDFWKRHFLARNVF	AAA85873.1	UL134Toledo
LRPATFGLETWAMYT	CAA35399	UL40
KDLDDSFDYLVERCQ	CAA35351	UL78
VAPPMWEIHHINKFA	CAA35414	UL55(gB)
CDPRMFLRLTHPELC	CAA35333	UL97
QLPYLSAERTVRWML	CAA35407	UL48(pp212)
FMTPKWDVFAYDSGI	CAA35397	UL38
RFFFRLTGQDEAHSF	CAA35339	UL103
HMYELSLSSFAAWWT	CAA35388	UL73(gN)
GAAPVIMMTWFYAFF	CAA35432*	UL33
NYLKHHYDLCFTCDR	AAA85896.1	UL154Towne
FFAKGMIRCDPVHES	CAA35428	UL29
GQYECRPQLQLPWVP	CAA35274	US7
WLFRRLFFPREDSEP	CAA35416	UL17
ARWEALRADMLEFGL	CAA35404	UL45
YVRYSHRLHTYAVCE	CAA35338	UL102
QYLNTLLITMMAAIW	AAA85887.1	UL148Toledo
ERLFPECGLPCLQFW	CAA35372	UL57
DYWNAVMLYRGDVES	CAA35395	UL36
NAPFPHLRWPVDLIP	CAA35423	UL24
HSLKILHSRILCEWH	AAA85896.1	UL154Towne
NTCVAYVNRVRTDMG	CAA35360	UL86(MCP=majorcapsidprotein)
HLQRIYSMMIEGASR	CAA35394	UL35
GLRRYLRRFEGSCVS	CAA35428	UL29
RLTHHASYHANYGAY	AAA85884.1	UL145Toledo
EEYVRSFCTRDLGTI	CAA35386	UL70
PIRFLRENTTQCTYN	CAA35390	UL75(gH)
FAIVSFKHMGPFEGY	CAA35351	UL78
QAMGAVWRAAFLANS	CAA35311	IRS1
RWDALHLHPRAALWA	CAA35311	IRS1
PACTFTFGSWNVAEA	CAA35427	UL28
HPTTGAYFDNGWKWT	CAA74074	UL42rev
LHPFFDFTHCQENSE	CAA35360	UL86(MCP=majorcapsidprotein)
CYASELCDESVRRFV	CAA35404	UL45
AVETLHCMRYLTSSL	CAA35423	UL24
MMTMWCLTLFVLWML	CAA35417	UL18
VKNLTMNMTEFPQYY	CAA35389	UL74(gO)
LRGDSCFVHDLRGMM	CAA35429	UL30
RTMTTFVHQSHNWHN	CAA35444	UL11
CGGPIGPRELSKWMT	CAA35292	US25
SICVNVFGQRSYFYC	CAA35413	UL54

NVVSYVCEEHLHSFT	CAA35363	UL89
GVEMPEMTWDLDVRN	AAA85875.1	UL136Toledo
YLASNAVLALRIIRL	CAA35426	UL27
PLYFEAECNRNYTLH	CAA35318	UL116
QRNCTHSFYLVNAMS	CAA35389	UL74(gO)
CWTVIQRYRLPGDCY	AAA85880.1	UL141Toledo
VWSYVGRVCTFYVTC	CAA35351	UL78
MYCFLFLQKDTFFHE	CAA35451	TRL3
EDSWYDLDETFWVLG	CAA35269	TRS1part
DMFDGVVASAYHRLR	CAA35362	UL88
ASVSYALRYDDESWR	CAA35311	IRS1
FFFPVGLYLPEDRGY	CAA35360	UL86(MCP=majorcapsidprotein)
AWFKHTFAGMYELSQ	CAA35427	UL28
NTMFENASTWTFSFG	CAA35392	UL77
LISISFLLVSFINCK	CAA35389	UL74(gO)
ATVQGQNLKYQEFFW	CAA35357	UL83(pp65)
GYDQLAARHFADYVD	CAA35354	UL80A
YLQNSFLHLLMNSGL	CAA35393	UL34
REYVGDYMSRIILCC	CAA35385	UL71
RLPGEDSWYDLDETF	CAA35269	TRS1part
HGGIHVLLYGTMLVK	CAA35427	UL28
YCWTMFPPMYPVLLL	CAA35420	UL21
GNKWTLDTCYYVYVT	CAA35444	UL11
EWNIGGHPVTHTVDM	CAA35314	US3
NLAFTYGSWGVAMLL	CAA35439	UL6
HLAASDLLNWYIVPV	CAA35406	UL47
LHPFGFVEGPGFMRY	CAA35395	UL36
MERRRGTVPLGWVFF	CAA35448	UL16
FTFGSWNVAEADEAN	CAA35427	UL28
SRERFAPEDFSFQWF	CAA35340	UL105
EEMFEALRIYYGDDP	CAA35360	UL86(MCP=majorcapsidprotein)
VWNAFRLIERHGFFA	CAA35273	US6
DATDSRLLMMSVYAL	CAA35390	UL75(gH)
MVTNLTVGRYDCLRC	CAA35448	UL16
VAFTWQHNESVVDLW	CAA35448	UL16
RLWCFCQDWKCHALY	CAA35264	US32
GGGVVSWRPESPSPD	CAA35404	UL45
LHSYFEDVERAAQGR	CAA35425	UL26
AETLRGFIRQGSFWF	CAA35427	UL28
RVTFSNIATHYHYNA	CAA35269	TRS1part
HLHGLPVNPHDPYLE	CAA35384	UL69
PHYTKLPKYDPDEFW	CAA35433	TRL14
TEPITMLGAYSAWGA	CAA35458	TRL10
QKYFSLDNFLHDYVE	CAA35386	UL70
FCTRDLADLCVRRDY	CAA35428	UL29
LINVTEEYTDYYRTM	CAA35444	UL11

RFMIAYCPFDEQSLL	CAA35384	UL69
EWHTNYLKHHYDLCF	AAA85896.1	UL154Towne
FDSAYQPAESMLFSE	CAA35372	UL57
ELQMMQDWVVERCNR	CAA35394	UL35
RQRERSAPKPQELLF	CAA35367	UL93
LLPRDVVEHWLHAQG	CAA35353	UL80
LPHEDAFYTGLSVWR	CAA35430	UL31
HRRAVNLSTLNSLWW	CAA35389	UL74(gO)
IKHLLSHDMVWPCPW	CAA35413	UL54
FCSSSPYQRLETRDW	CAA35295	UL132
HQCQTYYVECEPRCL	CAA35278	US11
PMEITRYVHRNEGRC	CAA35291	US24
NEYRTGISWSFGMLF	CAA35336	UL100(gM)
DAELMDHTSLYADPF	CAA35340	UL105
YMEFGIPLKEEHVAY	CAA35340	UL105
LMNLTYLWYLGDYGA	CAA35277	US10
RVVSYCQNNVKMVDR	CAA35411	UL52
MLSHYGTVYVYDWET	CAA35289	US22
RQGSFWFRCPRRFCF	CAA35427	UL28
LGDTQYFGVVRDHKT	CAA35427	UL28
VMKLESWAHVFRDYS	CAA35332	UL130(viralentry)
LAARHFADYVDPHYP	CAA35354	UL80A
DLADLCVRRDYEGLR	CAA35428	UL29
IPLCTGVIQKLGWCL	CAA35405	UL46
WEFLARDLLREEMEA	CAA35367	UL93
VLLTMAYYLFEGQYS	CAA35386	UL70
EVFALMIPKDMYLTW	CAA35405	UL46
AKTMEMRFTIAWMWF	CAA35262	US30
DVIYWAVSQNYDYAL	CAA35424	UL25
AQGTDLIRFERNIIC	CAA35414	UL55(gB)
YFMHRRLLPLFIVPD	CAA35386	UL70
DVKAWSHYLCCQTRL	CAA35423	UL24
PHDAATFYCPFLYPS	CAA35332	UL130(viralentry)
YATSLRRLDEELRHR	CAA35424	UL25
ELGDRLYQRFLREWL	CAA35408	UL49
RPAFMEHSRPVGYHP	CAA35456	TRL8
PSTLETFPDLFCLPL	CAA35390	UL75(gH)
VKAAWSLKELHTHQL	P19893	UL122(IE2)
ELEYQAMGAVWRAAF	CAA35311	IRS1
YYTSAFVFPTKDVAL	CAA35357	UL83(pp65)
LIAYLRYYHQDSWRD	AAA85875.1	UL136Toledo
CEWFERTIYQEGKFI	CAA35404	UL45
TDEQCCLLLQSAWTH	CAA35338	UL102
YDLDETFWVLGSNRK	CAA35269	TRS1part
EVSIRVHLCYWPEIV	CAA35447	UL14
PHYDLPLICAATWTA	CAA35355	UL81

VLGLSFGEFFENGLF	CAA35427	UL28
GAMYYGSGCRFDTVE	AAA85875.1	UL136Toledo
VFCAVASETWHRSLF	CAA35386	UL70
MDHTSLYADPFFLKY	CAA35340	UL105
DDHKAWLDLDESHWV	CAA35311	IRS1
ILHSRILCEWHTNYL	AAA85896.1	UL154Towne
ATPSFLRRHDVLERF	CAA35369	UL95
DLPLWCLCRLKCERH	CAA35408	UL49
EDAVCWLRRTAIVMR	CAA35281	US14
TYYVECEPRCLVPWV	CAA35278	US11
PKFFPPPMCRVPYNE	CAA35289	US22
PEMTWDLDVRNKWRR	AAA85875.1	UL136Toledo
EYLYRDLHSKWGVHL	CAA35406	UL47
RPLLRAWSLGLDTMA	CAA35423	UL24
VIGWMEPVNKAVFMD	CAA35427	UL28
PPRPRRRPTWMTAVF	AAA85875.1	UL136Toledo
VGRVCTFYVTCLMLF	CAA35351	UL78
HDWPEVSIRVHLCYW	CAA35447	UL14
LMRLGFTYFASWDLI	CAA35404	UL45
ANDIYRIFAELEGVW	CAA35357	UL83(pp65)
LRRVWMTVWASLFGY	CAA35386	UL70
TCGQVGPDVDVYEFP	CAA35413	UL54
LGCILDHQDGWGDHC	CAA35384	UL69
AVSETLFYYVYTSWC	CAA35372	UL57
KNTVSGYLFFGMCQL	CAA35419	UL20
FADYVDPHYPGWGRR	CAA35353	UL80
GGCCCAAGGDWLSAV	CAA35338	UL102
YMPPTVPYPDPAARL	CAA35269	TRS1part
FLRRHDVLERFAAAA	CAA35369	UL95
FVIPVTSVIFIHCYE	CAA35282	US15
FETSGGLVVFWQGIK	CAA35414	UL55(gB)
SRLKLVLSFVWLVGL	CAA35438	UL5
SLMDPLSPSRWEVAL	CAA35420	UL21
SHATCRYQVFVDAYG	CAA35428	UL29
DLIERIFEHMYFAAV	CAA35404	UL45
DAFYTGLSVWRGGEP	CAA35430	UL31
IYVAMSRVTDPEHLM	CAA35340	UL105
FYILCYDLFTSCGNR	CAA35368	UL94
LLTVTQSRWTIHHMY	CAA35440	UL7
VSEAWRFEEAVNMAL	CAA35369	UL95
STSTIAYRPDSSFMK	CAA35389	UL74(gO)
GFQRTFIDPLWDYLD	CAA35389	UL74(gO)
FDGQHFFTYHVNSSD	CAA35417	UL18
SVYLSPYLSSVWVPM	AAA85873.1	UL134Toledo
RDYEGLRRYLRRFEG	CAA35428	UL29
ALDEEDLEQYLLVWS	CAA35341	UL104

KDYTFSWYKDSLKAL	CAA35442	UL9
HGYWGKFRFCGVQEP	CAA35404	UL45
FNELMLWLGYYRELR	CAA35431	UL32(pp150)
FKTTVTSPNYPELCY	CAA35360	UL86(MCP=majorcapsidprotein)
AGPCAAWLSTRRELV	CAA35420	UL21
RFLWQRRQRARMLQH	CAA35396	UL37
ETYMGFLWDIPSCDR	CAA35395	UL36
LLLYRDGEWIICFCC	CAA35368	UL94
RIYTSLLDECACRDF	CAA35413	UL54
RRVRIFMIVCVLWCV	CAA35331	UL129(viralentry)
ISWMANVSAAYPTYL	CAA35417	UL18
TEYTCSFFSWGRHHN	CAA35320	UL118
RNPAKGCLGFLLYRH	CAA35372	UL57
LDVMQKYFSLDNFLH	CAA35386	UL70
AGGRWRFEDGGAAQR	CAA35447	UL14
RALENGKLTHCSDAC	CAA35333	UL97
EALRIYYGDDPERYN	CAA35360	UL86(MCP=majorcapsidprotein)
VGLLCMRIRSLLCSP	CAA35399	UL40
NVMHLHTPMLFLDSV	CAA35336	UL100(gM)
MILWSPSTCSFFWHW	CAA35276	US9
MSPTMVTIPPPQIPF	CAA35385	UL71
TLILAARDADEWFRH	CAA35311	IRS1
VLRFFGATEHGYSIC	CAA35413	UL54
LGGSPACTFTFGSWN	CAA35427	UL28
RFLTQLWENEYFRTF	CAA35341	UL104
NDFFGEFRALHLIGT	CAA35428	UL29
IVYDYDGPETRPGIY	CAA35405	UL46
RPIIGDTGGSSSSQR	CAA35424	UL25
LEDDDEYDELWFPLY	CAA35318	UL116
PEHGGEVREFKHLVY	CAA35386	UL70
QWELVLPWIVPMPLA	CAA35359	UL85
LYTELHPFFDFTHCQ	CAA35360	UL86(MCP=majorcapsidprotein)
CNRLCDALYFCYTQA	CAA35394	UL35
LLPYGDRLEVACIFP	CAA35447	UL14
LFSRDVSWYHSMSFS	P09704	US28
PRHCRLEMLILDEQV	CAA35416	UL17
VWCNCGDWQGHALRS	CAA35312	US1
GWLALGAVLPARWLG	CAA35311	IRS1
GLNAACAVYDHRLAF	CAA35408	UL49
VGGMRQLLFNKLNNL	CAA35384	UL69
QDEKNVTCQLTFWEA	CAA35414	UL55(gB)
RHGLLHCEAIYGEQM	CAA35293	US26
PDPAARLCRDMRRVT	CAA35269	TRS1part
MYENYIVPEDKREMW	CAA35325	UL123(pp72=MIprotein=IE1)
DAVVRRCLREYVGDY	CAA35385	UL71
CCYGIITTLRPGLWC	CAA35400	UL41

LFLQKDTFFHEQFLA	CAA35451	TRL3
CAAGGDWLSAVGHVL	CAA35338	UL102
ECACRDFILPNHYSK	CAA35413	UL54
DLWLYQNDTVIRNFS	CAA35448	UL16
EQLHEQLDRFLRHQH	CAA35406	UL47
VYYHVVDFERLNMSA	CAA35336	UL100(gM)
LVGWSWGTGRRPVGF	CAA35453	TRL4
PVDDWLNFRVDLFGD	CAA35394	UL35
AHLHEEILRYDGLCR	CAA35341	UL104
FERECYRVSVADNLG	CAA35424	UL25
NFEFLVRYYIGPWYA	CAA35392	UL77
MGGGRLPPLWLPLLI	CAA35447	UL14
QNSAECWSVRETKRC	CAA35420	UL21
IDDDTPMLLIFGHLP	CAA35390	UL75(gH)
NYASLLAFSHHPEFP	CAA35406	UL47
PQPYWPHLYRELRQA	CAA35352	UL79
GYRSQSVLTWTHECN	CAA35417	UL18
KYAESDYIFLQDMCP	CAA35396	UL37
WAHGLDIVEEDEWLR	CAA35445	UL13
TTTVTCDGFNYTVHK	CAA35461	TRL13
DIGYGVYVDKAFAML	CAA35338	UL102
IFNSYATTAWPMQCE	CAA35432*	UL33
LLIAWSEWGNCCLDA	CAA35447	UL14
SELYLGASGAMYLWT	CAA35422	UL23
YDDDEELTRLLAVWD	CAA35334	UL98
ALLFHYEHGLGRLLS	CAA35392	UL77
YWCSESYRRLNTEEE	CAA35447	UL14
RNNLDNGSDRRLPEA	CAA35367	UL93
VDLTFFFPVGLYLPE	CAA35360	UL86(MCP=majorcapsidprotein)
EDSLDKLIAWMTWLS	CAA35284	US17
REHPELELKYLNMMK	CAA35412	UL53
CGNFTTFNPMFFNVP	CAA35389	UL74(gO)
WVMFIHNKRCTDLDF	CAA35340	UL105
TSPSWFISVFGHTEG	CAA35386	UL70
FRLAAFFTRHWGAEA	CAA35352	UL79
AFLGFWSIFTVCFLC	AAA85880.1	UL141Toledo
CSIGHYYSTSPLNGM	AAA85895.1	UL153Towne
LKLFCYLVSTAWHQR	CAA35407	UL48(pp212)
DWSQTLIDDYFLLPA	CAA35426	UL27
DLRAANATYAQMVKK	CAA35407	UL48(pp212)
CDGAVWNAFRLIERH	CAA35273	US6
WFFTYCDLLRVGYFG	CAA74075	UL43rev
EVHDALLFHYEHGLG	CAA35392	UL77
APPTPETARVQRLLW	CAA35426	UL27
VRHALCWHRVEGGIS	CAA35358	UL84
NDNRAEAFCTSYGFF	CAA35417	UL18

RLREKWDTRGYLYKG	CAA35443	UL10
GSNHVEPLGWLVSPY	CAA35422	UL23
HIRQQFDWLEEPLLR	CAA35384	UL69
AYGAVFAYDAQEDCL	CAA35428	UL29
VDPASRERLLCFSPA	CAA35355	UL81
ALSSKDYTFSWYKDS	CAA35442	UL9
MRCQTPDYEDMLCYS	CAA35384	UL69
HDCGTYRGFQRNYFY	AAA85880.1	UL141Toledo
PRYHIRYFSYGNSVD	CAA35275	US8
TSLCHLLCYWCSESY	CAA35447	UL14
HSLTEIDLEHCQNDF	CAA35428	UL29
IAHTSPFESYVRWEE	CAA35396	UL37
RYPFLVGWSWGTGRR	CAA35453	TRL4
VKDQWHSRGSTWLYR	CAA35414	UL55(gB)
VAILCYLAYHWHDTF	AAA85877.1	UL138Toledo
AAIMSLLDEAEWRQT	CAA35397	UL38
RLPPLWLPLLIAWSE	CAA35447	UL14
DAAVTMRGGGWREDV	CAA35392	UL77
VVGIFDGQHFFTYHV	CAA35417	UL18
CCWVTLAHAGNPYED	CAA35275	US8
SNVTIKGNSTWDCLS	CAA35460	TRL12
VLTCGLQEAYILDKG	CAA35419	UL20
RWHACVPQKCEKSLC	CAA35275	US8
VQDSVSRDLGFADWS	CAA35426	UL27
FSPCHQCQTYYVECE	CAA35278	US11
EAAEETAAGEASAVA	CAA35447	UL14
AEMLNNWDGWDAFTI	CAA35290	US23
FYRAFRSGRFDLCTD	CAA35391	UL76
YQMCVMKLESWAHVF	CAA35332	UL130(viralentry)
YYFDSLYTYGWVLRT	CAA35442	UL9
NQHVCIVADSLMEFV	CAA35422	UL23
RFTEDTFVETFCDFL	CAA35426	UL27
CCLLLQSAWTHLYDV	CAA35338	UL102
MAMYTSESERDWRRV	CAA35264	US32
GMIRCDPVHESICAR	CAA35428	UL29
LQDFDVQHLRRLNEC	CAA35424	UL25
RIRLRNSWVASEDEL	CAA35418	UL19
GYEGFGWDGETLMEL	CAA35417	UL18
EVHRPVVRVFVDMWD	CAA35404	UL45
NCTQWSVIYSGFQPP	CAA35417	UL18
RLYQRFLREWLVCRQ	CAA35408	UL49
TTPTSPSMGFQRTFI	CAA35389	UL74(gO)
FVFPYLVLPNCCQVS	CAA35429	UL30
RFVARRNDVDFWLLR	CAA35338	UL102
ATAPSFDEAFLTDRL	CAA35407	UL48(pp212)
WSLKELHTHQLCPRS	P19893	UL122(IE2)

RHHTLMSTTCRCWSS	CAA35380	UL65
FGVIEAWEEASVRPT	CAA35397	UL38
VIKDCFLNLLDRWRP	CAA35422	UL23
GAVFGYCPLDGHVYP	CAA35311	IRS1
MSVKGVEMPEMTWDL	AAA85875.1	UL136Toledo
WLNAGAFRRLVHEAQ	CAA35426	UL27
LVRATDRHGDTVVYK	CAA35384	UL69
SYHSILADFNSYKAH	CAA35360	UL86(MCP=majorcapsidprotein)
ETFWVLGSNRKNDVY	CAA35269	TRS1part
VFVDAYGAVFAYDAQ	CAA35428	UL29
YLKGNCTQWSVIYSG	CAA35417	UL18
SHNLWCTCGNWQSHV	CAA35263	US31
LVFMLWGADAHTCEY	CAA35316	UL114
YFYVSPYTTEEMLRE	CAA35340	UL105
PYFVFLAYVYSMDCL	CAA35408	UL49
EWKLHAALFPYRALD	CAA35341	UL104
TLVECYVMHGREPVR	CAA35334	UL98
AELLDLTYQRLIYWA	CAA35406	UL47
DSIYFTFNKVFRSMH	CAA35366	UL92
FRHGAGEVVRLYRCN	CAA35311	IRS1
VAFQPLLAYAYFRSV	CAA35372	UL57
SAMYYFYVSPYTTEE	CAA35340	UL105
FTFIMEDYRTFAGTL	CAA35352	UL79
LHFTMFDSGVDRDYA	CAA35290	US23
FSPLGSFTRLGYDRL	CAA35362	UL88
RPEGYTLFFYILCYD	CAA35368	UL94
VTPDLDFYWVLPGGF	CAA35367	UL93
LAGPIQNYSITYLWF	CAA35389	UL74(gO)
GRIDGTHLAGFFGTS	CAA35386	UL70
EWKGAGVSRLREVWD	CAA35367	UL93
WLDLGPHLLHRRLET	CAA35405	UL46
SLYACTRCFRTHLCD	CAA35366	UL92
LTSHYSGIYYFDSLY	CAA35442	UL9
YRRRGLTEVLAYHLY	CAA35386	UL70
GFTSGGGVVSWRPES	CAA35404	UL45
DKVYVLGLSFGEFFE	CAA35427	UL28
PPPIDLSIPHVWMPP	CAA35390	UL75(gH)
DAFICTDYVYCALRL	CAA35424	UL25
DFLDAALDFNYLDLS	CAA35390	UL75(gH)
YSGIYYFDSLYTYGW	CAA35442	UL9
TVHRRFHTDMFHHDQ	CAA35333	UL97
NATVEWYNKSEGDVP	CAA35419	UL20
SINCGWEGERHRVVH	CAA35447	UL14
ALFDFLRVVRQQDAF	CAA35424	UL25
ERGLLHSYFEDVERA	CAA35425	UL26
AHSCNEAFLPLMAFC	CAA35363	UL89

HYEVNGTELRCRCLH	AAA85885.1	UL146Toledo
VRTWFVERTTFWRRT	CAA35445	UL13
HCCFQNFTATTTKGY	CAA35388	UL73(gN)
CDALYFCYTQAPETR	CAA35394	UL35
VTSVIFIHCYETSHP	CAA35282	US15
WPPLHGIMLGDTQYF	CAA35427	UL28
RAMLVMLDYYWIQLI	CAA35419	UL20
ITGSEFEGDFARYRS	CAA35407	UL48(pp212)
PRLADDVSREIAAWE	CAA35392	UL77
CTFYVTCLMLFVPYY	CAA35351	UL78
SQLPEKYIGFYQIRK	CAA35311	IRS1
WHDIEWIKYGPRAHQ	AAA85895.1	UL153Towne
AIKIWEFPCLRLHDG	CAA35400	UL41
FLSYRWLIRCCELYG	AAA85877.1	UL138Toledo
NSTSWQIPKLMKVAM	CAA35295	UL132
FPHLGYPCVYYHVVD	CAA35336	UL100(gM)
LFFFKIPQRLREKWD	CAA35443	UL10
TTSIRGLTCDPRMFL	CAA35333	UL97
VRQQDAFICTDYVYC	CAA35424	UL25
KKTAVCLISDEGYVF	CAA35423	UL24
EDVLMDRVRKRYLRQ	CAA35392	UL77
RHLDELARYGMMYTE	CAA35290	US23
FYCEYSDTDRLREVI	CAA35413	UL54
HFEAIFGTFCNRLEW	CAA35360	UL86(MCP=majorcapsidprotein)
PPVPVYAVHGLHTLM	CAA35338	UL102
RRPACALPHGWSVMN	CAA35347	UL109
VEYHHEVTSEFFGRV	CAA35361	UL87
TLRDYNVLFYTAHYT	CAA35424	UL25
TVCLLCELMACSYYD	CAA35411	UL52
KSNFTFCTPPSPPCC	CAA35330	UL128(viralentry)
QARVRDSHDRWCLCN	CAA35263	US31
WQHNESVVDLWLYQN	CAA35448	UL16
HLDLRPEELRDPFQI	CAA35358	UL84
PHRALFRLCLGLWVS	CAA35445	UL13
RSFCTRDLGTIIPTH	CAA35386	UL70
VQTLRKEMCAKSENS	AAA85873.1	UL134Toledo
LAEETARFVELAGCW	CAA35404	UL45
MDWKAHVEYAHPASE	CAA35312	US1
VIDRRITTFGWCSVN	CAA35413	UL54
LNQVFLCPTSPSWFI	CAA35386	UL70
GQSRNSVWHLLRMDT	CAA35334	UL98
RSRHETGIFTFIMED	CAA35352	UL79
YLMGSLVHSMLVHTT	CAA35390	UL75(gH)
HNEEPATFFCESDDA	CAA35368	UL94
THFAVQYTEEDFEAH	CAA35406	UL47
LRGDEEFIYHAGPLE	CAA35367	UL93

STLTKYAESDYIFLQ	CAA35396	UL37
MPAETFTCPKDKRPW	CAA35449	TRL1
PTAATGSLDYRWLGC	CAA35351	UL78
LTVFTVYLLSHLPSQ	CAA35390	UL75(gH)
EYFFQHMVGRLGVGP	CAA35407	UL48(pp212)
CPDEEPDRCWTVIQR	AAA85880.1	UL141Toledo
VYSQYNHTAKTITFR	CAA35389	UL74(gO)
PWIRESKMWVLPPPL	CAA35419	UL20
YLYHADHQALTARFF	CAA35405	UL46
LYVTYIYYTLCTPNC	CAA35378	UL63
TEEYTDYYRTMTTFV	CAA35444	UL11
QYVLVDTFGVVYGYD	CAA35395	UL36
PCLSPDMASCHFGEC	CAA35404	UL45
HTFAGMYELSQILHD	CAA35427	UL28
RREHLVFMLWGADAH	CAA35316	UL114
HDSHGLWCDCGDWRE	CAA35264	US32
VQGSRTRRPIPPILQ	AAA85892.1	UL151Toledo
LPEQVKAFCITPTQW	CAA35407	UL48(pp212)
LPNCSTITTTAGQDA	CAA35439	UL6
YLRRFEGSCVSLGWP	CAA35428	UL29
HYYSTSPLNGMCLDC	AAA85895.1	UL153Towne
TTNLVSKWMTQHFQS	CAA35372	UL57
FYSVFLRHCDVEPAI	CAA35374	UL59
KQFYGGLIFHTTWVT	CAA35438	UL5
EVNTISVRYLYHADH	CAA35405	UL46
WCLADDIHTSFLVHK	CAA35405	UL46
SYRDWEFLARDLLRE	CAA35367	UL93
VVDYSHNLWCTCGNW	CAA35263	US31
RDSHDRWCLCNAWRD	CAA35263	US31
CVLYVTPDLDFYWVL	CAA35367	UL93
NLPGYEHASEGWRFC	CAA35264	US32
FFWDANDIYRIFAEL	CAA35357	UL83(pp65)
TLDTCYYVYVTQNGT	CAA35444	UL11
LLAFSHHPEFPSIFA	CAA35406	UL47
EQIQSQVDEIQDLRT	CAA35341	UL104
TCFGREKNGCPFPAL	CAA35369	UL95
LFSKWLDKHNDNRWY	AAA85885.1	UL146Toledo
MAYYLFEGQYSTIST	CAA35386	UL70
LALLSFEETVHMYTT	CAA35340	UL105
AIKFHDLNKLTTGKM	CAA35360	UL86(MCP=majorcapsidprotein)
RRHVDGISCQDHFRA	CAA35399	UL40
TYSKPHDAATFYCPF	CAA35332	UL130(viralentry)
YEDDDYYYYREDEPR	CAA35275	US8
AETLRYVYYRNVDSA	CAA35367	UL93
TFGLAGPCAAWLSTR	CAA35420	UL21
VYVTVDCNLSMMWMR	CAA35313	US2

YTLFFYILCYDLFTS	CAA35368	UL94
HVLRYGYTGIFDDTS	CAA35417	UL18
PVVTLLLARQRDGLA	CAA35449	TRL1
TLKNSHTLRIYRRFY	CAA35358	UL84
TWTLHGMCISICYYE	CAA35448	UL16
TGSLDYRWLGCQIPI	CAA35351	UL78
NIEVSRPSVLCCFQE	CAA35356	UL82(pp71)
AQLSDVIYWAVSQNY	CAA35424	UL25
VSRDLGFADWSQTLI	CAA35426	UL27
VCGTCPQLVSGFVWY	CAA35372	UL57
ATLKSRPGFPCHVVW	CAA35283	US16
ACALPHGWSVMNSCS	CAA35347	UL109
FRRSYAYIYTTYLLG	CAA35414	UL55(gB)
YPRGYTLFVCDVEET	CAA35425	UL26
EVLINYCDIADNWVM	CAA35340	UL105
IFGTFCNRLEWVYFL	CAA35360	UL86(MCP=majorcapsidprotein)
LRYAQRNCTHSFYLV	CAA35389	UL74(gO)
PYLYRLNFCLIDTCL	CAA35378	UL63
KGATEAEREYLYRDL	CAA35406	UL47
YLRQLCSMTEELYLR	CAA35426	UL27
LRRFLRGDSCFVHDL	CAA35429	UL30
AMYSVELAVCYFSTS	CAA35351	UL78
YLALRDDGRPLAWRR	CAA35407	UL48(pp212)
ALDPHAFHLLLNTYG	CAA35390	UL75(gH)
DSDLYRIADNFHMFL	CAA35289	US22
YRIFAELEGVWQPAA	CAA35357	UL83(pp65)
VYSFFERDYVDEIVE	CAA35427	UL28
FHDRCASYNDTFYPT	CAA35382	UL67
LDIVEEDEWLREIQG	CAA35445	UL13
CPPNGNCEFPTCFTL	CAA35443	UL10
VWTPECKGWTYWTTL	CAA35396	UL37
SFPFATADIAEKMWA	AAA85880.1	UL141Toledo
YECVPDANTAPEIWV	CAA35427	UL28
VHMGEAARLHFTMFD	CAA35290	US23
SVFSIYWQKHSDLVY	CAA35362	UL88
LLNWLHHGLDLQRMH	CAA35407	UL48(pp212)
PRAALWAREPHGQWE	CAA35311	IRS1
VTHYYTNTSCSPQFM	CAA35434	UL1
ACYTVFGLGSIHPRF	CAA35328	UL126
DKGRRYMYLFSVSCA	CAA35419	UL20
AVNLSTLNSLWWWLQ	CAA35389	UL74(gO)
EHPTFTSQYRIQGKL	CAA35357	UL83(pp65)
VMTKPYFVFLAYVYS	CAA35408	UL49
PACDDGLFLYRTTVS	CAA35342	UL106
EIVRSLVVDARSGQV	CAA35447	UL14
HGMCISICYYENVTE	CAA35448	UL16

YAPLREELGYVRFET	CAA74075	UL43rev
EGELFFFSKNLYGNG	CAA35394	UL35
EPNCEQPEPAHWLEY	CAA35263	US31
YAAFCECGDGRDNGG	CAA35426	UL27
SGTGVAAVGAYRHQF	CAA35363	UL89
AQGQQAATVRAEFFW	CAA35376	UL61
KSGDSGFFDLSRWFG	CAA35454	TRL6
RLLEDAAVTMRGGGW	CAA35392	UL77
SPSRDRFVQLLFYMW	CAA35411	UL52
FYKAHCTSHMYELSL	CAA35388	UL73(gN)
VVAYTGAVYACDVRD	CAA35423	UL24
MEANKRDRQHQLATT	CAA35367	UL93
IGKHFTPVKFVYEVW	AAA85881.1	UL142Toledo
DIEDFERECYRVSVA	CAA35424	UL25
IAHWQTLVDVARGKF	CAA35405	UL46
TWTHECNTTENGSFV	CAA35417	UL18
ATADIAEKMWAENYE	AAA85880.1	UL141Toledo
ADAVIHASGKQMWQA	CAA35357	UL83(pp65)
LPPDSGSRGIVYCYV	CAA35391	UL76
YLDDCRDDEEFCHQF	CAA35426	UL27
PLLRHLDKYYAGLPP	P16832	UL115(gL)
RGKTLLLSSHWVPYP	CAA35436	UL3
EKYWRMRTTHTVEFY	CAA35375	UL60
GWEGERHRVVHYIPG	CAA35447	UL14
YVREDTAVYYLARNL	CAA35423	UL24
ADEWFRHGAGEVVRL	CAA35311	IRS1
IPEGFFGITFYKCLD	CAA35387	UL72
STPVLWCTLWGSRTR	CAA35439	UL6
IDLRTEHSYALWASL	CAA35386	UL70
RIYRRFYGPYLGVFV	CAA35358	UL84
AHPHHEYLSDLYTPC	CAA35390	UL75(gH)
YRAVACRSTIFSPED	CAA35368	UL94
GNNMTTLPVWTPECK	CAA35396	UL37
LLTSFGCLTDCWPFE	CAA35290	US23
ERLFRDPLTTYEYLD	CAA35426	UL27
ISTVEEYVRSFCTRD	CAA35386	UL70
RKTANMFMPGAFMDE	CAA35363	UL89
HTFWRLPVAVFFEPH	CAA35392	UL77
MKYHHLHGLPVNPHD	CAA35384	UL69
SPAFYYEALFLYMLD	CAA35407	UL48(pp212)
TASRDAADEVWALRD	CAA35431	UL32(pp150)
GQVLPVVWPPGWNLV	CAA35423	UL24
LHGPAPLSCNVTQWG	CAA35439	UL6
EVACIFPAHDWPEVS	CAA35447	UL14
MLGASVDRTYRLNRI	CAA35372	UL57
NMPRAFSFYLLTSAQ	CAA35430	UL31

LENCAFCQSALLEYD	CAA35390	UL75(gH)
DTFIERTPCEQAAYA	CAA35340	UL105
HATHSLQYAEGLRQL	CAA35391	UL76
HFTPVKFVYEVWRGQ	AAA85881.1	UL142Toledo
IAAPHLPLYNEFTSF	CAA35319	UL117
SGAYTEHVYECDLSC	CAA35434	UL1
AVEFQNYVKNSVRHM	CAA35406	UL47
AFVNPRHQYYFQMLI	CAA35334	UL98
QLVFLAVTIYYLVCW	CAA35336	UL100(gM)
LEYWNALFPVEVRSH	CAA35397	UL38
YVFVWYRGYEFPAPT	CAA35369	UL95
EIEGAEDKTFFHRVR	CAA35428	UL29
YALRYDDESWRPLST	CAA35311	IRS1
DRFVQLLFYMWAGTG	CAA35411	UL52
ADSLPQLLERGLLHS	CAA35425	UL26
LRDLKLCDGSYEEGF	CAA35407	UL48(pp212)
SGTVLRLSWPNGWFF	CAA74075	UL43rev
PWSRLIITKQFYGGL	CAA35438	UL5
KGTVFLCCTGFMPPL	CAA35284	US17
PRPTYVLVTVNSLAR	CAA35407	UL48(pp212)
PFDKNYVGNSGKSRG	CAA35403	UL44(pp50)
PGFFSWSNPACDDGL	CAA35342	UL106
LVANLPHEDAFYTGL	CAA35430	UL31
RCTDLDFGDLLKYME	CAA35340	UL105
QNLWTDLVTRHKMSG	CAA35407	UL48(pp212)
DFISRQHVLYNGCCV	CAA35360	UL86(MCP=majorcapsidprotein)
VVAAPGPSVRYRAHI	CAA35426	UL27
YHQGDMALMTLDVYC	CAA35399	UL40
METHLYSDLAFEARF	CAA35367	UL93
PTLTFSTIHSTTPWL	AAA85895.1	UL153Towne
FGSLISLLMAFMYYH	AAA85894.1	UL152Towne
RLLVPWIRESKMWVL	CAA35419	UL20
GRADAEDCWKPACPD	AAA85880.1	UL141Toledo
LVLIVAILCYLAYHW	AAA85877.1	UL138Toledo
FAPEDFSFQWFRSIS	CAA35340	UL105
VVELAYSDRRDHVWS	CAA35351	UL78
QFVLKEVEFRCRHTF	CAA35363	UL89
ASAAAGASSTWLAQC	CAA35392	UL77
NYFTGHHEDENFYLL	CAA35460	TRL12
INRGESYLTTIWLLN	CAA35419	UL20
FCNTTACNSPFLASG	CAA35322	UL120
PEFLYSLGVYRLHVN	CAA35361	UL87
RDPLTTYEYLDDCRD	CAA35426	UL27
PVYVGGFLARYDQSP	CAA35353	UL80
LIPEETGVTRPMMSL	CAA35385	UL71
VCASESSTSWAVTSN	CAA35439	UL6

RIQSLGNEIRCMLLP	CAA35419	UL20
ELASDLAGFFAKGMI	CAA35428	UL29
MIDLTSHHRPLTLFT	CAA35319	UL117
CCSACYKETMIYDMV	AAA85872.1	UL133Toledo
RVSTIRLYDWSEIND	CAA35422	UL23
YVPKEDDFCHKICYA	CAA35333	UL97
YMCPGIFDFLRYAHA	CAA35407	UL48(pp212)
TGVIQKLGWCLADDI	CAA35405	UL46
ATFTVHVRDATLHRV	CAA35338	UL102
LALLIDDFRYESIGP	CAA35428	UL29
FYTAHYTSRGALYLY	CAA35424	UL25
VAAVGAYRHQFLIYG	CAA35363	UL89
LTLGQWELVLPWIVP	CAA35359	UL85
LYVPAVSETLFYYVY	CAA35372	UL57
HLPLYNEFTSFRLPT	CAA35319	UL117
NMDEYSGDVWHLEVS	CAA35454	TRL6
EGRVNYASLLAFSHH	CAA35406	UL47
LRVGYFGHLNIKGLE	CAA74075	UL43rev
ITFNSSCLYITDKSF	CAA35403	UL44(pp50)
SETASTVSEDAVCWL	CAA35281	US14
SDEGYVFCYVREDTA	CAA35423	UL24
ATNSHYTMFVLDHGS	CAA35418	UL19
FLRVVRQQDAFICTD	CAA35424	UL25
LLAELMARSVAHNLY	CAA35406	UL47
RGDSDMFDGVVASAY	CAA35362	UL88
TWEKGDALCVLPPLF	CAA35359	UL85
RIICQKMWYFYLIGT	CAA35352	UL79
HTYAVCEKFIENLRF	CAA35338	UL102
CQLWKDWVTNASHDT	CAA35419	UL20
TVYFDAAYVHAPGIC	CAA35387	UL72
IPSWHVFASLDDLVP	CAA35356	UL82(pp71)
SAWLRDDVCDLQKRP	CAA35384	UL69
CLFLEPEERELIGRC	CAA35426	UL27
FFFSKNLYGNGEVFR	CAA35394	UL35
EVAIAECAAHMIISV	CAA35363	UL89
WGHKSICSFFPKLQG	CAA35437	UL4(gp48)
VCLISDEGYVFCYVR	CAA35423	UL24
REDYAQLSDVIYWAV	CAA35424	UL25
ISADPFSRDASWAAM	CAA35404	UL45
IFSTNQGGFMLPIYE	P19893	UL122(IE2)
HGETDFYMNWTLRRS	AAA85887.1	UL148Toledo
ESYLTTIWLLNCADN	CAA35419	UL20
PAVYTCVDDLCRGYD	P16832	UL115(gL)
QHFRWLNAGAFRRLV	CAA35426	UL27
AHTQSWYWLRILTSH	CAA35460	TRL12
GRVRNFEFLVRYYIG	CAA35392	UL77

CYDLFTSCGNRCDIP	CAA35368	UL94
VRTDTFEVDMLLYSG	CAA35360	UL86(MCP=majorcapsidprotein)
EMRFTIAWMWFPSVL	CAA35262	US30
HTFSGVRRPFTELGW	AAA85887.1	UL148Toledo
RFLREWLVCRQAERE	CAA35408	UL49
PQETTEYTCSFFSWG	CAA35320	UL118
RHALELQMMQDWVVE	CAA35394	UL35
ADLAVYHRNQWCHQR	CAA35408	UL49
PVRRYSVWCGMSSRL	CAA35293	US26
VLHSVMTLAAMLYKI	CAA35360	UL86(MCP=majorcapsidprotein)
HHYDLCFTCDRNLSL	AAA85896.1	UL154Towne
KIGEYLLEQGFPVYE	CAA35413	UL54
SIASARWEALRADML	CAA35404	UL45
LLACEDTAARCAYVE	CAA35428	UL29
IGLANLFSWDRSVAG	CAA35425	UL26
DAEDDVVFASELCFY	CAA35362	UL88
GDALCVLPPLFHGPL	CAA35359	UL85
HATCVLYFVAEEVHT	CAA35432*	UL33
EDTAARCAYVEAHRE	CAA35428	UL29
NYFLCQVCLYELDED	CAA35362	UL88
RNGFRHRDHFHTMRD	CAA35395	UL36
LFNDKCAFKLDLLRM	Q7M6N6	UL48A
FVAYAVARNRRDYTE	CAA35386	UL70
DTAVYYLARNLMEFA	CAA35423	UL24
LLLSSHWVPYPLRIP	CAA35436	UL3
LWVLSRGHREFYVYD	CAA35338	UL102
TWFYAFFYSTVQRTS	CAA35432*	UL33
ASSRANGTISWMANV	CAA35417	UL18
HVIWPGTSVLWAPDV	CAA35362	UL88
LVWGDERLVGPFNFF	CAA35338	UL102
AGGTRVPCVDRWPFF	CAA35340	UL105
QFMGYGTKNGLKNTW	AAA85891.1	UL150Toledo
CPFLYPSPPRSPLQF	CAA35332	UL130(viralentry)
LASYLCCPEPLRFVG	CAA35422	UL23
GLLFSAMYYFYVSPY	CAA35340	UL105
IPNGNDGRGCTSEGV	CAA35413	UL54
AAMQSVRDGLFCLGC	CAA35353	UL80
NETFLWYNLTVKPKP	CAA35440	UL7
WPHLYRELRQAFPGL	CAA35352	UL79
PHDGVYLPKDAFFSL	CAA35354	UL80A
FCQDWKCHALYAEWD	CAA35264	US32
IQNYSITYLWFDFYS	CAA35389	UL74(gO)
IQRNYLKGNCTQWSV	CAA35417	UL18
VQVPIRTRRLLVPWI	CAA35419	UL20
LDGTFHQGCYVAIFC	CAA35417	UL18
ENASTWTFSFGIWYY	CAA35392	UL77

NVERVRAVFAALYRA	CAA35361	UL87
QFWHGAIVLEYWNAL	CAA35397	UL38
TQQVSSQIRTRWEES	CAA35445	UL13
VDMVRHRIKEHMLKK	CAA35325	UL123(pp72=MIprotein=IE1)
YYGFKDYIGSLHGLT	CAA35372	UL57
ANFTFYYSCYNLTVS	CAA35437	UL4(gp48)
DANFTFYYSCYNLTV	CAA35437	UL4(gp48)
MARGTYGTYICSPNP	CAA35323	UL121
MCLSFDSNYCRNILK	CAA35431	UL32(pp150)
YTGRLIMNVRRSWEE	CAA35431	UL32(pp150)
VTGIYKHFFCDPQCA	CAA35411	UL52
TFNVSMDTAGMYECV	AAA85880.1	UL141Toledo
ASRGTVFEEETVWSL	CAA35404	UL45
YSVIRVSTIRLYDWS	CAA35422	UL23
HIPCNVHVSPGWIEA	CAA35415	UL15
SLLDECACRDFILPN	CAA35413	UL54
ISICYYENVTEDEII	CAA35448	UL16
VGCTPDMGRCLCYVP	CAA35368	UL94
QKEGHLYTVNCEASY	CAA35321	UL119
RPTRQLVLFMTPKWD	CAA35397	UL38
YQPAESMLFSEWPLV	CAA35372	UL57
ESLERFLTQLWENEY	CAA35341	UL104
GYPCVYYHVVDFERL	CAA35336	UL100(gM)
RLLPMVLLAAYCYCV	CAA35437	UL4(gp48)
HLLGTESDDEETTVW	CAA74073	UL41alt
VGLRLHDCAAFESCC	CAA35438	UL5
HAALFPYRALDEEDL	CAA35341	UL104
RRDADGQVIRESACY	CAA35425	UL26
ELYRALDAYRARIAV	CAA35367	UL93
DQYVKVYLESFCEDV	CAA35357	UL83(pp65)
TDIPERIYSLSDFTY	CAA35371	UL56
CRYQVFVDAYGAVFA	CAA35428	UL29
ASELRPGSGGWPEHA	CAA35312	US1
LVEPCARVYEIKCRY	CAA35334	UL98
ANRDDNFFAERTSGC	CAA35436	UL3
VQIGFLHTQLVMVPF	CAA35430	UL31
PRLTEVYQTLRDYNV	CAA35424	UL25
VINLFVDDCMRVFAA	CAA35422	UL23
FSFSPGPVVLLWCCL	P16832	UL115(gL)
TETALDYALGSWLFG	CAA35407	UL48(pp212)
TKRGYASYTIDDPFD	CAA35395	UL36
YNHTIDTCKNTVSGY	CAA35419	UL20
FLAYVYSMDCLHTVA	CAA35408	UL49
EWIKYGPRAHQLCSI	AAA85895.1	UL153Towne
PQKLWLLWQHDKHGI	CAA35444	UL11
NLFYVYYELARDLGS	CAA35361	UL87

RVPCVDRWPFFPFRA	CAA35340	UL105
TKKPTTTTRTTTTTT	CAA35444	UL11
SVIRCLGGYCDLIRE	CAA35404	UL45
FCDSDIEDFERECYR	CAA35424	UL25
VDFWLLRFQPGENEV	CAA35338	UL102
LWILSLLAVTLTVAL	P16845	UL22A
IADNWVMFIHNKRCT	CAA35340	UL105
LEEAAPFGRVSVTRH	CAA35430	UL31
HQLQSISELCYLIYV	CAA35424	UL25
CLVIRRRWRLVRDEG	CAA35422	UL23
EGSFCGCEGRSFFRT	AAA85892.1	UL151Toledo
MRIEWVWWLFGYFVS	AAA85881.1	UL142Toledo
MWSRVVFLRSSETQT	CAA35447	UL14
LIAVSVLSSRSKESL	CAA35276	US9
SCTGVALLAPERTVR	CAA35282	US15
SVTVEQPSTSADGSN	P16845	UL22A
QPASAAGTGFGIMDY	CAA35351	UL78
FISLNSQVRKTANMF	CAA35363	UL89
YEYPTHKMCELGNYH	CAA35433	TRL14
CAEKSDDIVIQISCV	CAA35413	UL54
DYSNTHSTRYVTVKD	CAA35414	UL55(gB)
HNLCYSTLLVPGGEY	CAA35413	UL54
PLDQELIMFGVIEAW	CAA35397	UL38
VAGVAADGSVLCYEI	CAA35425	UL26
NYCFARDCFTHPESV	CAA74075	UL43rev
ESFCEDVPSGKLFMH	CAA35357	UL83(pp65)
VGLWTSMGPLIRLPD	CAA35313	US2
RPMGEVAYYGGCCMV	CAA35261	US29
SDRNALWREMDTVSR	CAA35404	UL45
QRLQINDLLAYWPVI	CAA35340	UL105
QNYDYALYASTPALF	CAA35424	UL25
KELRMCLSFDSNYCR	CAA35431	UL32(pp150)
YALYASTPALFDFLR	CAA35424	UL25
LLNFIRQRLCCEWYV	CAA35289	US22
LDLPYPRGYTLFVCD	CAA35425	UL26
EANIFCTFDHKLSIA	CAA35359	UL85
DDVRGFTVFSHAACG	CAA35420	UL21
TPYTIYGTLDMSSLY	CAA35389	UL74(gO)
YHLKLRPATFGLETW	CAA35399	UL40
AVQTFCDTCPRYLVP	CAA35408	UL49
SHWVPYPLRIPHYPP	CAA35436	UL3
EFFKDSVIDLLTCRW	AAA85872.1	UL133Toledo
YLYRQNLQRLNENHR	CAA35424	UL25
GLRRCNFITVPEELP	CAA35422	UL23
MTLVLFATEYDSAHI	CAA35386	UL70
GFSKIIYIQNSAECW	CAA35420	UL21

CLSFDIECMSGEGGF	CAA35413	UL54
SLDNFLHDYVETHLL	CAA35386	UL70
SITYLWFDFYSTQLR	CAA35389	UL74(gO)
LLLVDEAHFIKKEAF	CAA35363	UL89
CGHCLNLGKEKLHCQ	CAA35408	UL49
FCLEPMEITRYVHRN	CAA35291	US24
ALPSSGYHFGFVRQN	CAA35406	UL47
GISLLSEFCRVLCCY	CAA35325	UL123(pp72=MIprotein=IE1)
LWLGVEYHHEVTSEF	CAA35361	UL87
PEEKGGGEGGGLRWF	CAA35375	UL60
DCGDWREHLYCVYDS	CAA35264	US32
LPPHPGFFSWSNPAC	CAA35342	UL106
MFDSGVDRDYARQFR	CAA35290	US23
KAKREVNTISVRYLY	CAA35405	UL46
DVELRELQAFLDENF	CAA35385	UL71
QLVLFMTPKWDVFAY	CAA35397	UL38
GKVLHLNKGWLCATI	CAA35315	UL113
LDFYWVLPGGFAVSS	CAA35367	UL93
DCLYELASDLAGFFA	CAA35428	UL29
PGPSVRYRAHIQKFE	CAA35426	UL27
HYRYEVANLTYNCTY	CAA35437	UL4(gp48)
GAYFDNGWKWTFALL	CAA74074	UL42rev
SLFNVNDIYELLYFL	CAA35392	UL77
ELDAMDEDELQQLSR	CAA35384	UL69
AFSFYLLTSAQRGYD	CAA35430	UL31
DRPMPVVPEECYDQR	CAA35311	IRS1
HVHDLKRIRFTEDTF	CAA35426	UL27
SYVVTNQYLIKGISY	CAA35390	UL75(gH)
RSSARLLEHCVGLAG	CAA35362	UL88
FFENGLFAVYSFFER	CAA35427	UL28
ARESRTPLCYASELC	CAA35404	UL45
SSNVFDLEEIMREFN	CAA35414	UL55(gB)
EEEEEKEKLLTYKDI	CAA35419	UL20
YASYTIDDPFDECPD	CAA35395	UL36
GNSSESESKTTHAYT	AAA85878.1	UL139Toledo
CAALSDDIKRYVTEF	CAA35360	UL86(MCP=majorcapsidprotein)
YHSEAYRMLFQIGHT	CAA35393	UL34
ACRSTIFSPEDDSSC	CAA35368	UL94
AQDVVTSWIEALRDA	CAA35430	UL31
AFRELLACEDTAARC	CAA35428	UL29
VVERCNRLCDALYFC	CAA35394	UL35
RDEVARTDEWKGAGV	CAA35367	UL93
KKCNQTEKWHNVDWI	CAA35433	TRL14
DGLYLYNAFRRTTSI	CAA35333	UL97
LSVTTVFYTWCSDLP	CAA35280	US13
DFGVVADLLKWIGPH	CAA35403	UL44(pp50)

ALLTLLSSDTAPRWM	P16832	UL115(gL)
MPSVVDISHFLKKQH	CAA35381	UL66
MQDWVVERCNRLCDA	CAA35394	UL35
RDDEAVLARLFEVRE	CAA35338	UL102
KELRSSNVFDLEEIM	CAA35414	UL55(gB)
TCDLLTPPPWYPITV	CAA35398	UL39
SFEETVHMYTTFRDI	CAA35340	UL105
RVLRRLLEDAAVTMR	CAA35392	UL77
SWDIQDEKNVTCQLT	CAA35414	UL55(gB)
DAITDAELMDHTSLY	CAA35340	UL105
TQGVINIMYMHDSDD	CAA35390	UL75(gH)
TRISTVNLYLSPERM	CAA35406	UL47
YNEQHYRYEVANLTY	CAA35437	UL4(gp48)
CYRVSVADNLGFEPS	CAA35424	UL25
VFAYDAQEDCLYELA	CAA35428	UL29
LQMNLSKISWLERHC	CAA35397	UL38
GHTQTVYFDAAYVHA	CAA35387	UL72
VSLMIFNSYATTAWP	CAA35432*	UL33
GWLVSPYDVINLFVD	CAA35422	UL23
TFIDPLWDYLDSLLF	CAA35389	UL74(gO)
YLCLMPAMTNNRACG	CAA35360	UL86(MCP=majorcapsidprotein)
TSAGEEMFEALRIYY	CAA35360	UL86(MCP=majorcapsidprotein)
EQESSSFFHSKAHFF	CAA35382	UL67
HLLRMDTVSATKFYE	CAA35334	UL98
SRPGFPCHVVWAPEV	CAA35283	US16
LHCEAIYGEQMRTPL	CAA35293	US26
AQSVQDTIQHMRFLY	CAA35407	UL48(pp212)
FDMLNVVSYVCEEHL	CAA35363	UL89
KLISLYVTYIYYTLC	CAA35378	UL63
VAWITCAALGIWCLA	AAA85872.1	UL133Toledo
DLQRLVVTRVWPPLL	CAA35386	UL70
PMGVLMNLTYLWYLG	CAA35277	US10
AVWATPCLASPWSTL	CAA35332	UL130(viralentry)
ESYIPGALCLYMDLM	CAA35288	US21
GVTLLVVAVVSLGRW	CAA35322	UL120
SPGWIEANSVTFKRQ	CAA35415	UL15
LVVLLDELGAVFGYC	CAA35311	IRS1
GGGSVIGSTGGNDET	CAA35340	UL105
DDSWKQLGEDFAHQC	CAA35273	US6
TYILPADCRYAPLFA	CAA35319	UL117
TNQPPIFQIYYLLHA	CAA35412	UL53
NGTNRNASYFGENAD	CAA35414	UL55(gB)
IYNAQDHTVVRVLRP	CAA35407	UL48(pp212)
ISDTDLQRLVVTRVW	CAA35386	UL70
DCYRSQPHPPKFLPV	AAA85880.1	UL141Toledo
LFLMNTFLLHQEGFR	CAA35334	UL98

PAMQRLLECRFQQEP	CAA35360	UL86(MCP=majorcapsidprotein)
DASWAAMCKWMSTLS	CAA35404	UL45
LLDRWRPPKTSRPWK	CAA35422	UL23
LLLARQRDGLADWNV	CAA35449	TRL1
CFLNLLDRWRPPKTS	CAA35422	UL23
WEHRLSSVWRDALFT	CAA35281	US14
FEDVERAAQGRLRHG	CAA35425	UL26
SVGELVPEPRTPYAV	CAA35413	UL54
LDDAFLDTLALLYNN	P16832	UL115(gL)
YATTAWPMQCEHLTL	CAA35432*	UL33
WSVIYSGFQPPVTHP	CAA35417	UL18
FQIFTQQCEMVTEGY	CAA35407	UL48(pp212)
KDDLSYKDIPRCFVA	CAA35413	UL54
TPCSSSGRRDHSLER	CAA35390	UL75(gH)
VDMDCEKSAYMLEAG	CAA35385	UL71
PEAVLAARALHMPTL	CAA35352	UL79
DDPERYNIHFEAIFG	CAA35360	UL86(MCP=majorcapsidprotein)
PVCLAPDHHLSKWLD	AAA85894.1	UL152Towne
AEAALKDLYAAFCEC	CAA35426	UL27
RRESSCAVLVHHVGR	AAA85880.1	UL141Toledo
IITHAVIINYYYVAQ	CAA35424	UL25
LREYLADLLYLNKAE	CAA35334	UL98
SKNVQYEFMGLIFTV	CAA35430	UL31
AADGSVLCYEISREN	CAA35425	UL26
ELTALAPVGPAAFLY	CAA35372	UL57
PYPLRIPHYPPSWSR	CAA35436	UL3
CSQGAYVCCQEYLHP	CAA35395	UL36
HRLRECYHPAFRPMP	CAA35333	UL97
YNVLFYTAHYTSRGA	CAA35424	UL25
ARTDEWKGAGVSRLR	CAA35367	UL93
FLPRTPSPSNTVCCI	AAA85891.1	UL150Toledo
IMATQLRDLATWVYT	CAA35389	UL74(gO)
EVYQTLRDYNVLFYT	CAA35424	UL25
AYSSGSSASSSGFVA	CAA35263	US31
VAVCQTLRTFWPQIS	CAA35407	UL48(pp212)
ASTSPETQFYYTRHE	CAA35386	UL70
LFVDDVGLYSTALFF	CAA35351	UL78
NPWVCEEPKHEWDTS	AAA85896.1	UL154Towne
SVLCYEISRENFVVR	CAA35425	UL26
PNYEISWLKQNKTYI	CAA35417	UL18
ACEAVSPYDRFRLIE	CAA35369	UL95
VDTFGVVYGYDPAMD	CAA35395	UL36
SLHHFELSYRFHDED	CAA35395	UL36
RDGIRWQYQELQYLV	CAA35445	UL13
IHRSASHLTAYESYL	CAA35430	UL31
DWSEINDWRVMVGSN	CAA35422	UL23

MTTSAMTAPDTRRQL	CAA35429	UL30
NTTVVAMALCYGFGN	CAA35336	UL100(gM)
LKFGFQYHLEGWFPL	CAA35396	UL37
IDTCKNTVSGYLFFG	CAA35419	UL20
RQAGVTGIYKHFFCD	CAA35411	UL52
LRLCRHMDPEQDYRL	CAA35430	UL31
VAPDCVLSYVESRFH	CAA35372	UL57
KNTADAMERGLIHSF	CAA35360	UL86(MCP=majorcapsidprotein)
KNITNLAFTYGSWGV	CAA35439	UL6
VSSHLLTVTQSRWTI	CAA35440	UL7
FLEAPWESAPQPPRL	CAA35406	UL47
WVASEDELDVSRGDA	CAA35418	UL19
REFYVYDGYSGHGPV	CAA35338	UL102
LDFGDLLKYMEFGIP	CAA35340	UL105
RRACEDAIRCDYGVF	CAA35289	US22
RFSRNPSLFFSGDAL	CAA35430	UL31
VALRHVVCAHELVCS	CAA35357	UL83(pp65)
MLFLDSVQLVCYAVF	CAA35336	UL100(gM)
AYDDSKFCRYVELIC	CAA35341	UL104
NNGALTLVIPSWHVF	CAA35356	UL82(pp71)
ATEYFALLHGIQTFS	CAA35407	UL48(pp212)
LWGADAHTCEYLIDR	CAA35316	UL114
RGLQNKTEDFLHWLL	CAA35437	UL4(gp48)
GHGAMDLTCQKAVTL	CAA35368	UL94
SQRMEHGQEETHDIP	CAA35340	UL105
LLADYAETFSPLGSF	CAA35362	UL88
RVAEEWKLHAALFPY	CAA35341	UL104
MYLDMCTSSGHRPRP	CAA35311	IRS1
GHRVQTYCEDLEGRV	CAA35392	UL77
GEVVNTMFENASTWT	CAA35392	UL77
TGQYVVCTLLDYKTF	CAA35426	UL27
LKRIRFTEDTFVETF	CAA35426	UL27
ATQFTTVAMVHYHQE	AAA85877.1	UL138Toledo
RGLTLESDLAVWAAL	CAA35426	UL27
RYLGYMSQRLSSLEK	CAA35372	UL57
GDSGGMMGRGGRMLG	CAA35372	UL57
FDRTVVIQAYVLSSM	CAA35279	US12
HLALVCGTCPQLVSG	CAA35372	UL57
QAATVRAEFFWGAAG	CAA35376	UL61
QKDDKRSLFCYMREI	CAA35343	UL107
VADDSHDLWCCCMDW	CAA35312	US1
YQEGKFIFELYRLPR	CAA35404	UL45
MYATDPHDRDEVART	CAA35367	UL93
RVKRKKLQTFGYLSF	CAA35453	TRL4
NMFMPGAFMDEIIGG	CAA35363	UL89
ASEMEEPSNSTSWQI	CAA35295	UL132

IFKNTGCAVSLCCFV	CAA35409	UL50
SGILFFLAPSMAQFW	CAA35397	UL38
GLEKTFLCCDKFLLP	CAA74075	UL43rev
QFLQEECMWKLVGKS	CAA35368	UL94
QLVMVPFVPHACPHY	CAA35430	UL31
FPYRALDEEDLEQYL	CAA35341	UL104
GIYVLTSSIAHWQTL	CAA35405	UL46
WTHNTEVMKFKETSF	CAA35395	UL36
DRFQNFEAVLARGMH	CAA35367	UL93
RFSERPDEILVRWEE	AAA85877.1	UL138Toledo
LALTAEFGLGCLEAY	CAA35423	UL24
SYLMTHAGRYADVIQ	CAA35366	UL92
GAFRRLVHEAQYLPE	CAA35426	UL27
EVVVSSPRTHYLMLL	CAA35390	UL75(gH)
YEPEVSMAYIYQYND	CAA35322	UL120
AGASSTWLAQCAERP	CAA35392	UL77
ASHLTAYESYLVSIT	CAA35430	UL31
DLMYLFVSVLYFMPS	CAA35288	US21
PCTVMTHSWPMVSIR	AAA85880.1	UL141Toledo
RQHAHTMDDLVMVFH	CAA35397	UL38
IPAGFCSSSPYQRLE	CAA35295	UL132
EHGGVLPSFFFSGSA	CAA35422	UL23
GFTYFASWDLIERIF	CAA35404	UL45
KDSLKALNMLCYYTE	CAA35442	UL9
FSHAACGASLMDPLS	CAA35420	UL21
CESNIIVIDECGLML	CAA35340	UL105
LSMDTFQLFTLTMSF	CAA35336	UL100(gM)
RVLQYLIHAFQIDFL	CAA35363	UL89
LSYDDHEVELYRALD	CAA35367	UL93
GDWLSAVGHVLGRLP	CAA35338	UL102
NQWKEPDVYYTSAFV	CAA35357	UL83(pp65)
LLPRQYTLNATVEWY	CAA35419	UL20
HEVTSEFFGRVLAQL	CAA35361	UL87
GTELRLCNLRCFENS	CAA35423	UL24
PTHASMGEFARLLLG	CAA35386	UL70
VSRSGFRGFVQEGLR	CAA74075	UL43rev
AALQWLDLGPHLLHR	CAA35405	UL46
RVRMFYAVFTTLGLR	CAA35367	UL93
LERTARFIKDNFSEP	CAA35413	UL54
LRYSTLNTNAYDYFG	CAA35363	UL89
MREIIAVGNFSKYML	CAA35343	UL107
LICKNPNYSVCDAML	CAA35409	UL50
YMLQVVVFFYYFYNA	CAA35340	UL105
VTCLMLFVPYYCFRV	CAA35351	UL78
ADSASDFDADCWCMW	CAA35449	TRL1
TVFEEETVWSLCPPN	CAA35404	UL45

GYISEHVTSACASAG	CAA35338	UL102
HDSGLYVCICDPSYE	CAA35397	UL38
SIRNPSYAAEMTRLF	CAA35340	UL105
EPLSLFLMNTFLLHQ	CAA35334	UL98
LRDTLPFWSTLLPCA	CAA35279	US12
SAYHRLRMSNIPRSS	CAA35362	UL88
AHQLCSIGHYYSTSP	AAA85895.1	UL153Towne
SKNGLLWCEYVYRHP	CAA35289	US22
TIYNVTTEHAGKYVL	AAA85895.1	UL153Towne
MSLLHTFWRLPVAVF	CAA35392	UL77
MSASEWWVESALEKL	CAA35404	UL45
CYVIEFKTTYSDADD	CAA35391	UL76
AGRLYFIGLVSVYEC	CAA35427	UL28
SNEEAETLRYVYYRN	CAA35367	UL93
FRLLRGIFLITLVIW	CAA35435	UL2
GIFDFLRYAHAKPRP	CAA35407	UL48(pp212)
LEVPGRCPHENFPFW	CAA35293	US26
VRIELGVYFFSSPTS	CAA35383	UL68
ILQKDTFIERTPCEQ	CAA35340	UL105
GLNLKTLLVDLFYRP	CAA35360	UL86(MCP=majorcapsidprotein)
ERTIRSEAEDSYHFS	CAA35414	UL55(gB)
TLEDALNDMYLLLTL	CAA35394	UL35
GCDSPRHLYISLYLL	CAA35405	UL46
YVFCYVREDTAVYYL	CAA35423	UL24
VYLHSVESYSLQFHD	CAA35382	UL67
PGSRTLEDALNDMYL	CAA35394	UL35
VLQTKAHIHPGFALT	CAA35360	UL86(MCP=majorcapsidprotein)
YDFLISADPFSRDAS	CAA35404	UL45
MGDFQGIFECQYSAD	CAA35290	US23
WWTMLNALILMGAFC	CAA35388	UL73(gN)
FYVRQKTYHLLGTES	CAA74073	UL41alt
SVRWHATHSLQYAEG	CAA35391	UL76
LYGEYERRFADLSSL	AAA85877.1	UL138Toledo
MCTDPRRTAGWERLT	AAA85884.1	UL145Toledo
SLWRARMSAALTRTA	CAA35409	UL50
FVVFIINASFIWSWT	CAA35322	UL120
RSVSLRIKRELLCLH	CAA35426	UL27
LGNYWLHRDPRGPGC	AAA85885.1	UL146Toledo
HQHDGGGDEDRLPFY	CAA35406	UL47
LLCFSPACFSHSLYL	CAA35355	UL81
FHIAISTAFCGMIWL	CAA35280	US13
IKSVFVFPYLVLPNC	CAA35429	UL30
RVSSSVSECYVQHGV	CAA35276	US9
LRDFKELFFCLEPME	CAA35291	US24
QQVHAAHDVWCNCGD	CAA35312	US1
AAMLYKISPVSLVLQ	CAA35360	UL86(MCP=majorcapsidprotein)

YKHFFCDPQCAGNIR	CAA35411	UL52
NEVRPHAGVIDCAPF	CAA35338	UL102
WPVPTAYKAFLWKYA	AAA85886.1	UL147Toledo
STNFFFSQCEHYPSF	CAA35261	US29
EDDFCHKICYAVDMS	CAA35333	UL97
GDRLEVACIFPAHDW	CAA35447	UL14
RGSYPEFLYSLGVYR	CAA35361	UL87
NYHQTTPRHDICFDC	CAA35433	TRL14
IGLRRDLLEDFRYIC	CAA35396	UL37
AADLLFVCTLPLWMQ	P09704	US28
TLRLFKTTVTSPNYP	CAA35360	UL86(MCP=majorcapsidprotein)
IRFHTDFRGEVVNTM	CAA35392	UL77
LYVCICDPSYEFLQM	CAA35397	UL38
YPYRVCSMAQGTDLI	CAA35414	UL55(gB)
LSQILHDRANLLRVC	CAA35427	UL28
LDDFMRRQRGRHLDL	CAA35425	UL26
QIIYNFYTFFMCLYV	CAA35406	UL47
GGIYCYDDLRDCVYE	CAA35395	UL36
CYLQHQLQSISELCY	CAA35424	UL25
YSRVIGGTVFVAYHR	CAA35414	UL55(gB)
ATYRGRLMVMGDYSV	CAA35422	UL23
EIALGYRSQSVLTWT	CAA35417	UL18
EDTVFDLKDVDEWFE	CAA35290	US23
GEFRALHLIGTVSHA	CAA35428	UL29
WYVNPFSLAHLLDAI	CAA35318	UL116
QQRAFCRASRVLTDP	CAA35429	UL30
PKVVVNTDDFLKKML	CAA35404	UL45
FCDTCPRYLVPLRAL	CAA35408	UL49
DTVLLMHFFYTHYRS	CAA35406	UL47
VVVFFYYFYNALGDT	CAA35340	UL105
ECYFPTVVRRPLRVP	CAA35404	UL45
YYLARNLMEFARVGL	CAA35423	UL24
AYVYHYEVNGTELRC	AAA85885.1	UL146Toledo
SVCDAMLKTDTVYCV	CAA35409	UL50
SVESYSLQFHDRCAS	CAA35382	UL67
VLSNFPHLGYPCVYY	CAA35336	UL100(gM)
ITEHRDLFADVFRRP	CAA35394	UL35
TETCDLDGYMCPGIF	CAA35407	UL48(pp212)
TMCNLALSTPFLMEH	P19893	UL122(IE2)
PETARVQRLLWHSLR	CAA35426	UL27
RNSWVASEDELDVSR	CAA35418	UL19
ARSKYPYHFFATSTG	CAA35414	UL55(gB)
AMLKTDTVYCVEYLL	CAA35409	UL50
FMDAHGGIHVLLYGT	CAA35427	UL28
QLGLHQFVDHTRGYV	CAA35359	UL85
MMTDRTERRRRLTHA	CAA35342	UL106

CLSADWIRFLSLPDH	CAA35316	UL114
WTHLYDVLFRGFAGQ	CAA35338	UL102
NTFLLHQEGFRNLPF	CAA35334	UL98
VIGTIGLANLFSWDR	CAA35425	UL26
TEFPQYYILAGPIQN	CAA35389	UL74(gO)
EAKRILVKGHGAMDL	CAA35368	UL94
ITARNMPRAFSFYLL	CAA35430	UL31
SWRPLSTVDDHKAWL	CAA35311	IRS1
FVVNDGTRYQMCVMK	CAA35332	UL130(viralentry)
SETVLTVWMSGLIRT	CAA35333	UL97
YFIGLVSVYECVPDA	CAA35427	UL28
RWCPTPGRGRRGGEG	AAA85880.1	UL141Toledo
LLLSRMEALEWFKKF	P09724	US20
LLNCADNNTYWYSGN	CAA35419	UL20
DHEVELYRALDAYRA	CAA35367	UL93
LSKKLDAFLLNWLHH	CAA35407	UL48(pp212)
SLAELSHFTQLLAHP	CAA35390	UL75(gH)
LPFTVLRLSYAYRIF	CAA35334	UL98
YYHGVHSRELRCPCT	AAA85894.1	UL152Towne
FCNRLEWVYFLTSGL	CAA35360	UL86(MCP=majorcapsidprotein)
LINLSMDRYCVIVWG	CAA35259	US27
LIPIVVAYTGAVYAC	CAA35423	UL24
PVERQALTELEYQAM	CAA35311	IRS1
DERLVGPFNFFYGNG	CAA35338	UL102
GMYELSQILHDRANL	CAA35427	UL28
YCLVSSPSPRHTFDM	CAA74074	UL42rev
FMLIPWRVTAPYLRD	CAA35279	US12
ATDDEWTLQKVFYLC	CAA35360	UL86(MCP=majorcapsidprotein)
WVWWLFGYFVSSVGS	AAA85881.1	UL142Toledo
RKAAHHTALHDCLAL	CAA35358	UL84
NEYKVTSDACMMTMY	CAA35325	UL123(pp72=MIprotein=IE1)
FLVGVELMITHFQRT	CAA35424	UL25
LCQLLLLYRDGEWII	CAA35368	UL94
DIPCLSNHHDDSDAI	CAA35340	UL105
SSMAVYDEETMRQSQ	CAA35430	UL31
HLMMNVNPLRLPYEK	CAA35340	UL105
LGWQTLSNHVIRRLS	CAA35316	UL114
RRLITADEERRGPER	CAA35341	UL104
TWTFSFGIWYYRLKR	CAA35392	UL77
ESWAHVFRDYSVSFQ	CAA35332	UL130(viralentry)
ETFPDLFCLPLGESF	CAA35390	UL75(gH)
RLLICPRGFELLPEF	CAA35419	UL20
FDLKDVDEWFEQRRL	CAA35290	US23
EAWEEASVRPTRQLV	CAA35397	UL38
ELCDESVRRFVLRHM	CAA35404	UL45
HAGFQVRAASVMTRR	CAA35445	UL13

KFLSVIYYSSCTVGF	CAA35432*	UL33
HLPTLYQLSFGGPLG	CAA35368	UL94
LNKPTCPYGVDHQLF	CAA35422	UL23
QLREMLRRDEQTRLR	CAA35407	UL48(pp212)
DVFGMRQIVTQAGEP	CAA35407	UL48(pp212)
VEAQHDTATPHTMWI	CAA35442	UL9
RTHRAAVFFHATFMA	CAA35426	UL27
CRVLCCYVLEETSVM	CAA35325	UL123(pp72=MIprotein=IE1)
ELARYGMMYTEAVYR	CAA35290	US23
VCELAFSFASVFFDS	CAA35372	UL57
YMHDSDDVLFALDPY	CAA35390	UL75(gH)
YTSRMVTNLTVGRYD	CAA35448	UL16
IYYGDDPERYNIHFE	CAA35360	UL86(MCP=majorcapsidprotein)
CYVMHGREPVRDGLG	CAA35334	UL98
RSRELSYDDHEVELY	CAA35367	UL93
DMGVRVQDLFRVFPM	CAA35360	UL86(MCP=majorcapsidprotein)
YCDLLRVGYFGHLNI	CAA74075	UL43rev
ALLEKMQVVFDPYGR	CAA35407	UL48(pp212)
RPTPLASYACYTVFG	CAA35328	UL126
LVVAVVSLGRWDVVT	CAA35322	UL120
LVSPDRLVVGYFDSL	CAA35427	UL28
SRILPVGSMYRGSDA	CAA35265	US34
VNHVHRRRRRICHLP	CAA35368	UL94
ADNNTYWYSGNAYNH	CAA35419	UL20
AEQTWYADVVRCVRA	CAA35367	UL93
VASETWHRSLFPRDL	CAA35386	UL70
EPDVYYTSAFVFPTK	CAA35357	UL83(pp65)
YLLEQGFPVYEVRVD	CAA35413	UL54
DFSFQWFRSISRVER	CAA35340	UL105
LFEGQYSTISTVEEY	CAA35386	UL70
KLCDGSYEEGFVVIR	CAA35407	UL48(pp212)
DCESSASWLSSFTPA	CAA35415	UL15
GAIVLEYWNALFPVE	CAA35397	UL38
AWIRHYNRSTHGHHL	CAA35460	TRL12
DEGRDAQRLASYLCC	CAA35422	UL23
AGRDDDTFVFMGARH	CAA35364	UL90
FGCLTDCWPFEVAPA	CAA35290	US23
DWFGAVYAIQMDDPN	CAA35291	US24
CHGWTQQVSSQIRTR	CAA35445	UL13
LVVTRVWPPLLEHLT	CAA35386	UL70
NRRSSVFSIYWQKHS	CAA35362	UL88
NFYTFFMCLYVYSPT	CAA35406	UL47
LKFVLQDFDVQHLRR	CAA35424	UL25
AGATMTLVLFATEYD	CAA35386	UL70
VWDDEPLSLFLMNTF	CAA35334	UL98
TAPVEWKSPDRQIPK	CAA35437	UL4(gp48)

LTLWIACTGAVMVGD	CAA35280	US13
CGCEGRSFFRTLGTG	AAA85892.1	UL151Toledo
DVAFTSHEHFGLLCP	CAA35357	UL83(pp65)
GYDLRFSRNPSLFFS	CAA35430	UL31
IYNVSESSGGTYYQK	CAA35439	UL6
LWAREPHGQLAFLLR	CAA35269	TRS1part
HDGVYLPKDAFFSLL	CAA35353	UL80
FTSCGNRCDIPSMTR	CAA35368	UL94
FCTFDHKLSIADVGK	CAA35359	UL85
WLTIILYVFMWTYLV	CAA35274	US7
WKGHKPFRFEAHGSL	CAA35431	UL32(pp150)
FVWLVGLRLHDCAAF	CAA35438	UL5
QSKCAFLKGYLSEGC	CAA35385	UL71
ATFYCPFLYPSPPRS	CAA35332	UL130(viralentry)
TEELYLRLDGTLCLF	CAA35426	UL27
IDYGLHRVFTQLELR	CAA35361	UL87
RSPMPSHIHTMIFSP	AAA85873.1	UL134Toledo
FPGGETARKDKFLHM	CAA35413	UL54
EFARVGLRAVETLHC	CAA35423	UL24
IKTAFGMRVLGLGTV	CAA35423	UL24
TDPSRCDPYQVYFYG	CAA35261	US29
PQLLERGLLHSYFED	CAA35425	UL26
DMALMTLDVYCCRQT	CAA35399	UL40
TAYESYLVSITEQYN	CAA35430	UL31
DNFFAERTSGCITRL	CAA35436	UL3
EIAYRDVIHTTLRRM	CAA35291	US24
FLNRELGDRLYQRFL	CAA35408	UL49
FNYTVHKRCDRSYEV	CAA35461	TRL13
FNMEFSSACVHGQDI	CAA35403	UL44(pp50)
IWVSGHGHAFAYLPG	CAA35427	UL28
VDQCVALVFYYDSGM	CAA35404	UL45
YIVPEDKREMWMACI	CAA35325	UL123(pp72=MIprotein=IE1)
ISSFTSTNLTHTAVH	CAA35442	UL9
YAAVDLTIKMWFLLG	CAA35351	UL78
HYTMFVLDHGSVRIE	CAA35418	UL19
NVTCQLTFWEASERT	CAA35414	UL55(gB)
PDDEVAHLWGVTPSV	CAA35404	UL45
GHSYVLRAIAHTSPF	CAA35396	UL37
KQRTVPRCFCCGSLQ	CAA35457	TRL9
KDADPISTVTETRWF	CAA35342	UL106
STSELTGVCYAFNVP	CAA35404	UL45
LFVLWMLRVVGMHVL	CAA35417	UL18
LVQLRLDVNPDLMYA	CAA35367	UL93
RRLHRLWCFCQDWKC	CAA35264	US32
VTNLTESCINRGESY	CAA35419	UL20
VLANRVLQYLIHAFQ	CAA35363	UL89

DREQWERPRWDALHL	CAA35311	IRS1
FYYFYNALGDTRLYR	CAA35340	UL105
VRAEFFWGAAGEGSV	CAA35376	UL61
RLHRRTVLFNELMLW	CAA35431	UL32(pp150)
RQHVLYNGCCVVTAP	CAA35360	UL86(MCP=majorcapsidprotein)
MNSLLAELNRLGVAH	CAA35365	UL91
AEALERVAERCDDRH	CAA35334	UL98
MLCYYTEKLEEIDSK	CAA35442	UL9
YLLTSAQRGYDLRFS	CAA35430	UL31
VQRRGLFFFSNGKSE	CAA35379	UL64
LLFPALCFCLLCEAV	CAA35318	UL116
GMVRKSYTHVAWTIV	CAA35313	US2
ISTAFCGMIWLGIPD	CAA35280	US13
ITPDVIYEVCMLVPQ	CAA35368	UL94
VRRYSTVSPGKEVTL	AAA85887.1	UL148Toledo
ALYYVHFPFFSDLAN	CAA35282	US15
TLFKAFQITKRTYKG	CAA35411	UL52
WASLFGYTHPDRHPV	CAA35386	UL70
EEDPIRYYVSVYDEL	CAA35295	UL132
RRKMMYMCYRNIEFF	CAA35325	UL123(pp72=MIprotein=IE1)
EWKSPDRQIPKNITC	CAA35437	UL4(gp48)
MIFLCAYLIRYREFF	AAA85872.1	UL133Toledo
VFIFVDRLFQHFSFL	CAA35365	UL91
NVTELASIHPGETWT	CAA35448	UL16
WKLVGKSRKHREYRA	CAA35368	UL94
RVTYDGELIYGSYLL	CAA35369	UL95
AHAKPRPTYVLVTVN	CAA35407	UL48(pp212)
SRDYVHVRLLSYRGD	AAA85887.1	UL148Toledo
WREHLYCVYDSHFQR	CAA35264	US32
DVTFGVLKRALVRLH	CAA35394	UL35
QLETLSRPDEPCCTP	AAA85880.1	UL141Toledo
KHVSQFVLKEVEFRC	CAA35363	UL89
VSESEFIVRYHTEHE	CAA35460	TRL12
LAVATGQYVVCTLLD	CAA35426	UL27
HAVLNAGPGKRFFSP	CAA35430	UL31
SRKGFLFTKQIKSSK	CAA35372	UL57
RLRVWWLSDAGVRET	AAA85875.1	UL136Toledo
QFFSILATMLSKGIM	CAA35351	UL78
DFFVVTVSIDDDTPM	CAA35390	UL75(gH)
LGLPQSAWRRWRSHV	CAA35439	UL6
WEELERKCLARIQER	CAA35431	UL32(pp150)
SITEFLNIGLRRCNF	CAA35422	UL23
LLSYRGDPLVFKHTF	AAA85887.1	UL148Toledo
RRNSNLFVFCTERDY	CAA35393	UL34
LVSNFTSNISARWFR	CAA35440	UL7
LPRLSIASARWEALR	CAA35404	UL45

TTPVAHLHEEILRYD	CAA35341	UL104
CASYNDTFYPTNFTP	CAA35382	UL67
VTVTGNNMTTLPVWT	CAA35396	UL37
PNYAGALGRTAHWLF	CAA35279	US12
VSRREIWRLADSVDM	CAA35293	US26
VIMMTWFYAFFYSTV	CAA35432*	UL33
TTPRHDICFDCNDTS	CAA35433	TRL14
ATLLCRVDHLTWISK	CAA35430	UL31
SFGEFFENGLFAVYS	CAA35427	UL28
KDNQCMTDYDYLEVS	P09704	US28
TQSRWTIHHMYNKLL	CAA35440	UL7
LFSTVTPCLHQGFYL	CAA35390	UL75(gH)
KAHIHPGFALTAVRT	CAA35360	UL86(MCP=majorcapsidprotein)
SVGVSSLPMRELAWR	CAA35312	US1
KANWARESRTPLCYA	CAA35404	UL45
YDVSEYVFSGRSVLD	CAA35427	UL28
RDCVFDIATTLEHLS	CAA35406	UL47
VQVFIDLRTEHSYAL	CAA35386	UL70
VARLLRGDEEFIYHA	CAA35367	UL93
KMLRAHGTPVAEDFM	CAA35334	UL98
ALTAVRTDTFEVDML	CAA35360	UL86(MCP=majorcapsidprotein)
SSQIRTRWEESNVVS	CAA35445	UL13
YRFHDEDPETYMGFL	CAA35395	UL36
GEDDVLATIRNTLSA	AAA85891.1	UL150Toledo
IFIHCYETSHPSNIG	CAA35282	US15
ANGTISWMANVSAAY	CAA35417	UL18
WRGWLLFPALCFCLL	CAA35318	UL116
RDKAAFTSSVSTRTP	CAA35438	UL5
NVAPGERDLTRRIIT	CAA35424	UL25
TRQAHFLARGLLGFF	CAA74075	UL43rev
VIYNNTQGCGYKYDW	CAA35430	UL31
VYCALRLLACPDRPI	CAA35424	UL25
SGRFNRRSSVFSIYW	CAA35362	UL88
ELLIEDFDIYVDSFP	CAA35431	UL32(pp150)
EEFCHQFLRAYLTPI	CAA35426	UL27
LIITKQFYGGLIFHT	CAA35438	UL5
DGQVIRESACYVSRV	CAA35425	UL26
SVTCYARTDCKGPFT	CAA35321	UL119
RWWLYSGWWWLTFGC	CAA35261	US29
QCLILMSVCAFCWLV	CAA35288	US21
LWFDFYSTQLRKPAK	CAA35389	UL74(gO)
LLLFQRPQWAHGLDI	CAA35445	UL13
MRPGLPPYLTVFTVY	CAA35390	UL75(gH)
TESCINRGESYLTTI	CAA35419	UL20
VELAGCWGLYAAILC	CAA35404	UL45
IDSYRRAFCTLADAI	CAA35333	UL97

NVVTPKAAYAELFFL	CAA35430	UL31
SLYADPFFLKYVKPP	CAA35340	UL105
LLKEAIKIWEFPCLR	CAA35400	UL41
MGTVCSQGAYVCCQE	CAA35395	UL36
SVAASAARFDEIRRR	CAA74075	UL43rev
AVSSLAWPHDGVYLP	CAA35353	UL80
GNEALTTVVLNGSQT	CAA74075	UL43rev
TESDDEETTVWEKRR	CAA74073	UL41alt
FGALIIFLAYVYHYE	AAA85885.1	UL146Toledo
CIICVGSPTQTEALE	CAA35340	UL105
AFCQSALLEYDDTQG	CAA35390	UL75(gH)
SCDLAFGSRSQTRYL	CAA35447	UL14
SLHPIDVNGSLFWHQ	CAA35328	UL126
VTEDEIIGVAFTWQH	CAA35448	UL16
MRNSLDEVSGTADVS	CAA35293	US26
TLHVNHSCDYSVRQS	CAA35318	UL116
WLLPRPEGYTLFFYI	CAA35368	UL94
SAFARQELYLMGSLV	CAA35390	UL75(gH)
TDTRHCTSCTHPYVI	CAA35323	UL121
ERTIYQEGKFIFELY	CAA35404	UL45
SLSFRLITETAGTYT	CAA35447	UL14
LHDCAAFESCCYDIT	CAA35438	UL5
INIMYMHDSDDVLFA	CAA35390	UL75(gH)
VASCKFLSVIYYSSC	CAA35432*	UL33
ANNRVSFHGVKNMRI	CAA35403	UL44(pp50)
QRLIYWACTLMPYVL	CAA35406	UL47
DGMYGRGEKELCIAH	CAA35323	UL121
GTIEGDIGVFPCFVP	CAA35387	UL72
SWRRVVDYSHNLWCT	CAA35263	US31
QYVKVQFGYHLGAFF	CAA35336	UL100(gM)
VFSLTVHAPYDIHFG	CAA35430	UL31
CTDYVYCALRLLACP	CAA35424	UL25
VLGSNRKNDVYQRRW	CAA35269	TRS1part
PRYRCLSFDIECMSG	CAA35413	UL54
DLFETGAARSFFFPG	CAA35338	UL102
KFDLMSLLEREESWR	CAA35263	US31
VSCPTVMRFDQRLLE	CAA35385	UL71
EGFVVIREQLSYLMT	CAA35407	UL48(pp212)
RWGTAYSSGSSASSS	CAA35263	US31
WAVSQNYDYALYAST	CAA35424	UL25
GTPQFFDQFDTNNAM	CAA35384	UL69
PVKLEFEKVFVSLMM	CAA35413	UL54
QIWSPTPWRLRNHDC	AAA85880.1	UL141Toledo
GRLMVMGDYSVIRVS	CAA35422	UL23
ESIMFAIVSFKHMGP	CAA35351	UL78
RLVLDEAFPTFPLYD	CAA35407	UL48(pp212)

VYAVHGLHTLMRETA	CAA35338	UL102
AMIFYRYSETCMEVT	CAA35443	UL10
ASAAAAAAACEDLSE	CAA35340	UL105
CYYVYVTQNGTLPTT	CAA35444	UL11
PPRRRPARTDLYYYR	CAA35393	UL34
QDESVAHLLAATRRR	CAA35391	UL76
GIVYCYVIEFKTTYS	CAA35391	UL76
IHASGKQMWQARLTV	CAA35357	UL83(pp65)
DYRWLGCQIPIQYAA	CAA35351	UL78
EDHMLVFDPHSSAEC	CAA35407	UL48(pp212)
ERLLFEDRRLMAYYA	CAA35278	US11
FKFFHQDPNRVLDCI	CAA35319	UL117
RQFPPPPRFPPRCSD	CAA35415	UL15
IMATYLCDDIHAIRF	CAA35363	UL89
GGLIGSVIDLPLWCL	CAA35408	UL49
FLRHCDVEPAIATFD	CAA35374	UL59
CIGLTMQSMYENYIV	CAA35325	UL123(pp72=MIprotein=IE1)
HLTIQVLESWFTPWV	AAA85881.1	UL142Toledo
AAGTGFGIMDYVELA	CAA35351	UL78
RGQPKFSSIWRGLRD	CAA35427	UL28
LATSDGLYLYNAFRR	CAA35333	UL97
CILLIGLRRDLLEDF	CAA35396	UL37
VFPQDMLQCLWLELK	CAA35407	UL48(pp212)
VDLFYRPAFLLMPAA	CAA35360	UL86(MCP=majorcapsidprotein)
DEFSFCDSDIEDFER	CAA35424	UL25
RQLKGALVDFDFLRL	CAA35391	UL76
YSDCVDPGLAVYRVS	CAA35438	UL5
GNEQVSRIACTSCED	CAA35412	UL53
FETGGDVGREFMLAR	CAA74075	UL43rev
GPCRDPPRGFTVFRT	CAA35377	UL62
ARDPLYAAEQLHEQL	CAA35406	UL47
LWCTLWGSRTRVSLG	CAA35439	UL6
AACSYAHTLSLHSDM	AAA85892.1	UL151Toledo
MWRTRWEDGAPTFTR	CAA35290	US23
LTPPSNGCTVDVGRN	CAA35262	US30
ASTPALFDFLRVVRQ	CAA35424	UL25
LIASLDEAETQPLYR	CAA35386	UL70
LMGGATQEPLDTGLY	CAA35269	TRS1part
LQSAWTHLYDVLFRG	CAA35338	UL102
LLQYCIVRFIGTRLF	CAA35274	US7
GDYMSRIILCCEQNE	CAA35385	UL71
SVHLVLSNFPHLGYP	CAA35336	UL100(gM)
RYNIHFEAIFGTFCN	CAA35360	UL86(MCP=majorcapsidprotein)
CGYKYDWSNVVTPKA	CAA35430	UL31
TEFPHYHRHDGGFPL	CAA35360	UL86(MCP=majorcapsidprotein)
IYILVGTQMRKDMWT	CAA35259	US27

GSFTRLGYDRLVSAD	CAA35362	UL88
DLDGYMCPGIFDFLR	CAA35407	UL48(pp212)
ALREFLEAPWESAPQ	CAA35406	UL47
DSRFSDEALTETVWL	CAA35386	UL70
VVIQAYVLSSMLCVW	CAA35279	US12
KVYLESFCEDVPSGK	CAA35357	UL83(pp65)
KTHLASFLSAFARQE	CAA35390	UL75(gH)
VEPLGWLVSPYDVIN	CAA35422	UL23
FYAVFTTLGLRCPDN	CAA35367	UL93
AERRAANWRRQMRRL	CAA35264	US32
LHQTPHMWARSIRLI	CAA35393	UL34
PSVWASRGTVFEEET	CAA35404	UL45
LITKGGLCSSMAVYD	CAA35430	UL31
QPFMRPHERNGFTVL	CAA35357	UL83(pp65)
PPEPLREYLADLLYL	CAA35334	UL98
LELEDYDRRCRCNNQ	AAA85886.1	UL147Toledo
ESSTSWAVTSNRLPN	CAA35439	UL6
TARFVELAGCWGLYA	CAA35404	UL45
GGLIFHTTWVTGFVL	CAA35438	UL5
GKSRKHREYRAVACR	CAA35368	UL94
AVWRAAFLANSTGRA	CAA35311	IRS1
RLVFEAAQRGLFDYS	CAA35361	UL87
INPILYALLGHDFLQ	CAA35432*	UL33
RERASGVHLQRYVRA	CAA35427	UL28
DMSDESYRLGQGSFG	CAA35333	UL97
AECHNAAVYHCEGLH	CAA35407	UL48(pp212)
VVTMAAAIGIGWYEP	CAA35322	UL120
DTFVETFCDFLELVQ	CAA35426	UL27
TVTLEKEQRVRMFYA	CAA35367	UL93
TWDLFTYPIYAVYGT	CAA35320	UL118
YVDPHYPGWGRRYEP	CAA35354	UL80A
TVYLVSAIFREREES	CAA35334	UL98
TLERYRVCEPHEETT	CAA35340	UL105
ETDERGQWIMLFLHH	CAA35356	UL82(pp71)
RNASYFGENADKFFI	CAA35414	UL55(gB)
MLIQQYVLSQYYIKK	CAA35334	UL98
QHRVRLRVLWYVNSF	CAA35367	UL93
LFRLCLGLWVSSYLV	CAA35445	UL13
HDLNKLTTGKMLFHV	CAA35360	UL86(MCP=majorcapsidprotein)
PECGLPCLQFWQKVC	CAA35372	UL57
RARLPDTVCVHYVYL	CAA35386	UL70
IAQVFDGCYHSEAYR	CAA35393	UL34
GDGVFITESSVFETR	CAA35427	UL28
NEYFRTFRLRRLVTH	CAA35341	UL104
DIPSCDRCVRRRRFK	CAA35395	UL36
TADACNEGVKAAWSL	P19893	UL122(IE2)

TCDGITPDVIYEVCM	CAA35368	UL94
GGLNLDDFMRRQRGR	CAA35425	UL26
HPSLEHGGVLPSFFF	CAA35422	UL23
CFLRTCLRLVTPVGF	CAA35338	UL102
LGSWLFGIPVCLGVH	CAA35407	UL48(pp212)
ILVRWEEVSSQCSYA	AAA85877.1	UL138Toledo
RNLMEFARVGLRAVE	CAA35423	UL24
DIFKQKATVFLVPRR	CAA35363	UL89
TKVCTDYTSVTCTPC	AAA85883.1	UL144Toledo
SMRDDNWGLLFRTLL	CAA35314	US3
RKLDQSDWVRGAWIV	AAA85884.1	UL145Toledo
IPDYRSVSLRIKREL	CAA35426	UL27
LGAVLPARWLGCAAG	CAA35311	IRS1
HPHASPRSDHTLFPV	CAA35449	TRL1
YEISRENFVVRAADS	CAA35425	UL26
FRRFYHECSQTMLHE	CAA35333	UL97
MLRLLSVEEICEEHT	CAA35424	UL25
DIYRRLLRVFPSFVA	CAA35391	UL76
EAELLLPRDVVEHWL	CAA35353	UL80
EIDLEHCQNDFFGEF	CAA35428	UL29
GSVIDLPLWCLCRLK	CAA35408	UL49
PWRDIDSVTFVPRNL	CAA35318	UL116
RSEAEDSYHFSSAKM	CAA35414	UL55(gB)
HFLARGLLGFFRVGF	CAA74075	UL43rev
FCESDDAKYLCAVGS	CAA35368	UL94
TRLASDSLCLFHSSF	CAA35436	UL3
PGPLVHPSLYIGFPC	CAA35375	UL60
SVLDQRLWWEIQYSS	CAA35323	UL121
NTNGHATCVLYFVAE	CAA35432*	UL33
YLRLDGTLCLFLEPE	CAA35426	UL27
VERVTLPYLCHILAL	CAA35341	UL104
PETLQIFYNFHEGKI	CAA35394	UL35
RSASLSFLDWPDGSV	CAA74075	UL43rev
PGVLLVWGDERLVGP	CAA35338	UL102
LAVGPDDEVAHLWGV	CAA35404	UL45
SICFGVPGETGGGCF	CAA35372	UL57
ARRGGGGGVGSNSSR	CAA35358	UL84
TCNAFYGFTGVVNGM	CAA35413	UL54
MSMTASSSTPRPTPK	CAA35340	UL105
TSIICEEDLDGDCRQ	CAA35333	UL97
RVSAFVAYAVARNRR	CAA35386	UL70
AHDSEDGVSVWRQHL	CAA35449	TRL1
ARTGSLHHFELSYRF	CAA35395	UL36
DRDHDDAPPTYEQAM	CAA74074	UL42rev
RHSMRCRRRDMASSA	AAA85891.1	UL150Toledo
HHLGHRKNAHTQSWY	CAA35460	TRL12

NRILDYCRKMRLIDP	CAA35372	UL57
ALLGRLYFISSKHTL	CAA35358	UL84
AAEGDEFSFCDSDIE	CAA35424	UL25
GSDDYVWLSRLLDLA	CAA35334	UL98
TFYKCLDAQFVCMPE	CAA35387	UL72
LWSGSLPHLPVYDVR	CAA35445	UL13
RCLVPWVPLWSSLED	CAA35278	US11
DCLHTVAVRTMAFLR	CAA35408	UL49
RLLECRFQQEPMGGA	CAA35360	UL86(MCP=majorcapsidprotein)
PREPPHRALFRLCLG	CAA35445	UL13
TLIEYSLPVPFHRFY	CAA35360	UL86(MCP=majorcapsidprotein)
FITESSVFETRASGR	CAA35427	UL28
CLIDTCLELCPPTFS	CAA35378	UL63
RRNDVDFWLLRFQPG	CAA35338	UL102
FLCPTSPSWFISVFG	CAA35386	UL70
SRQTTIMVTKYSEKS	CAA35360	UL86(MCP=majorcapsidprotein)
DMYPVCMAKTNSPNY	CAA35413	UL54
EWYNKSEGDVPEEFM	CAA35419	UL20
PWRVTAPYLRDTLPF	CAA35279	US12
PVVESLYLVSRSGFR	CAA74075	UL43rev
TVMFLTRRTRDGFCG	CAA35418	UL19
LGSFSSFYSQIARSL	CAA35399	UL40
LRSAACRRRPGDLGF	CAA35451	TRL3
CAYLIRYREFFKDSV	AAA85872.1	UL133Toledo
GWPSQCIYVVGGEHS	CAA35428	UL29
DARLSYVMLTVYPCS	CAA35276	US9
EANALLLSRMEALEW	P09724	US20
ALKRAMYSVELAVCY	CAA35351	UL78
WQARLTVSGLAWTRQ	CAA35357	UL83(pp65)
IMRHGPCLIRHSPRC	CAA35404	UL45
CQKAVTLGGAGAWLL	CAA35368	UL94
TLPYLCHILALGTLD	CAA35341	UL104
ECQYSADVLRDWVRN	CAA35290	US23
GVDRDYARQFRWLCR	CAA35290	US23
LWTDHIYSDSLTFVA	CAA35422	UL23
LLLCAVWATPCLASP	CAA35332	UL130(viralentry)
ALPPQLLFHYRNLVA	CAA35360	UL86(MCP=majorcapsidprotein)
DRLVVGYFDSLSSLY	CAA35427	UL28
EWTLQKVFYLCLMPA	CAA35360	UL86(MCP=majorcapsidprotein)
ANRYRHYEFQTLSLG	CAA35411	UL52
TSPAYDVSEYVFSGR	CAA35427	UL28
LGQQPTTVPPPIDLS	CAA35390	UL75(gH)
YGSGCRFDTVEMVDE	AAA85875.1	UL136Toledo
VRNQNDNRAEAFCTS	CAA35417	UL18
RVWPPLLEHLTQHYE	CAA35386	UL70
EKEQRVRMFYAVFTT	CAA35367	UL93

NVGPIYLCPVPAFFT	CAA35411	UL52
LFTTEDSLDKLIAWM	CAA35284	US17
LHMVLPRRLHLEPAF	CAA35413	UL54
LSEFCRVLCCYVLEE	CAA35325	UL123(pp72=MIprotein=IE1)
DAIKFLNHQCRVCHF	CAA35333	UL97
FVGRFVNEGVLSPDQ	CAA35423	UL24
LYSDLAFEARFADDE	CAA35367	UL93
VDAFLLIRTFVARCI	CAA35360	UL86(MCP=majorcapsidprotein)
TRLSYGRSIFTEHVL	P16832	UL115(gL)
LSGAQTLHLRLVWPD	CAA35367	UL93
DVRRFLLCMRVYSTV	CAA35284	US17
LLWCEYVYRHPQTPF	CAA35289	US22
NMTTARESSVHDARN	CAA35295	UL132
QLVARFFFRLTGQDE	CAA35339	UL103
AVFCRVHGLRGFQVS	CAA35396	UL37
AARDADEWFRHGAGE	CAA35311	IRS1
LLGNSVDALYIRERL	CAA35353	UL80
CMSELSALGNVLGFC	CAA35333	UL97
PHYAVPFTTPGKPGC	CAA35430	UL31
YHWHDTFKLVRMFLS	AAA85877.1	UL138Toledo
SNRATSQRASLNAVH	AAA85891.1	UL150Toledo
RSDAAAVVVEPRPRP	AAA85873.1	UL134Toledo
GQVLHNDASCYIAGG	CAA35447	UL14
TVNLYLSPERMFFHP	CAA35406	UL47
EEFIYHAGPLEPPSK	CAA35367	UL93
DVPEEFMDYVILTPL	CAA35419	UL20
LICSALYLLFEEKEP	CAA35339	UL103
YCHTRYETFLRVMGD	CAA35290	US23
QRCVRVGWKAVLGLN	CAA35408	UL49
IKHEGLVKTLVECYV	CAA35334	UL98
IGVMLVLIVAILCYL	AAA85877.1	UL138Toledo
LSLHLDDYPYLMVEI	CAA35361	UL87
ALHDHRLWPPFVTHL	CAA35360	UL86(MCP=majorcapsidprotein)
AYCPFDEQSLLDLTV	CAA35384	UL69
WGVTPSVWASRGTVF	CAA35404	UL45
ALTARFFVPEGLVEF	CAA35405	UL46
AVDLDRPPLWSGSLP	CAA35445	UL13
RVPQYDFLISADPFS	CAA35404	UL45
IGTGKMRITPDAFEI	CAA35352	UL79
SQRLVGEFMVRDPLL	CAA35424	UL25
SQTMLHEYVRKNVER	CAA35333	UL97
PLPQRLLPRQFPPPP	CAA35415	UL15
GEPSAAEGDEFSFCD	CAA35424	UL25
PGIASFAATLLHRYP	CAA35450	TRL2
RLLAVWDDEPLSLFL	CAA35334	UL98
YAVDVLKSGRCQMLD	CAA35390	UL75(gH)

LVFDPHSSAECHNAA	CAA35407	UL48(pp212)
TQTEALESRYDHYTQ	CAA35340	UL105
FGTKDDVRGFTVFSH	CAA35420	UL21
VFTAQLRHYCEHQDK	CAA35393	UL34
CVPELRVDYTSSAYM	CAA35313	US2
HDICFDCNDTSLTIY	CAA35433	TRL14
LRRLNECIPMPAFAL	CAA35424	UL25
LYPEYIYTVLKYPVQ	CAA35407	UL48(pp212)
FQRNYFYIGRADAED	AAA85880.1	UL141Toledo
NANVLTVCRHVEAHK	CAA35361	UL87
EVKLAICHDNYYISR	CAA35411	UL52
IAEKMWAENYETTSP	AAA85880.1	UL141Toledo
MDILTTCVETMCNEY	CAA35325	UL123(pp72=MIprotein=IE1)
ALLFAFLHYFTTLKQ	CAA35417	UL18
WSRTIPNRIRYIPAT	CAA35436	UL3
CRWVRYCSCSCKCSC	AAA85872.1	UL133Toledo
LNVNTHFAVQYTEED	CAA35406	UL47
VYLIIVYDYDGPETR	CAA35405	UL46
ARWDQMFSYLAKLGT	CAA35281	US14
LVVVTEFSEIFGIPQ	CAA35406	UL47
WCLTLFVLWMLRVVG	CAA35417	UL18
ILLHVPTHGLLYTVL	CAA35359	UL85
DGPETPWGQLICCEE	CAA35293	US26
LTETVWLHDDDVAST	CAA35386	UL70
LLRNSFHRYAVDVLK	CAA35390	UL75(gH)
LFLYMLDVATVPEAE	CAA35407	UL48(pp212)
SIVRQAMQHAGFQVR	CAA35445	UL13
GLFFFSNGKSESYTF	CAA35379	UL64
MTDSVRRYSTVSPGK	AAA85887.1	UL148Toledo
LDDYPYLMVEIGRVL	CAA35361	UL87
ITVAVAVCQTLRTFW	CAA35407	UL48(pp212)
SISELCYLIYVQLPS	CAA35424	UL25
YVEAHREAQLTLIWP	CAA35428	UL29
LTVGRYDCLRCENGT	CAA35448	UL16
NGTYVSGLYNCTDCT	AAA85883.1	UL144Toledo
PSPPWSKLTYSKPHD	CAA35332	UL130(viralentry)
EDTVGAASHHHRPCV	CAA35384	UL69
HARFPVAGLRRYCMS	CAA35333	UL97
FHQALRRLFAPLCVH	CAA35408	UL49
VLLFHRGLETLLLRE	CAA35352	UL79
ACAVYDHRLAFDVIL	CAA35408	UL49
KMVFLISISFLLVSF	CAA35389	UL74(gO)
SLVSGLLFSAMYYFY	CAA35340	UL105
LQDTVSESEFIVRYH	CAA35460	TRL12
NDLAFLVGVELMITH	CAA35424	UL25
RLLEYRRVVAYDADA	CAA35386	UL70

RDVCIDYGLHRVFTQ	CAA35361	UL87
EWDGKESDDESSASS	CAA35264	US32
LKDSDFLDAALDFNY	CAA35390	UL75(gH)
FRSGRFDLCTDSVLD	CAA35391	UL76
NNHHHHHHHHNAVTD	CAA35315	UL113
FNERLPVFNFVADFD	CAA35386	UL70
GPSSLAPGRCFSCVP	CAA35310	J1I
NMRDMYLDMCTSSGH	CAA35311	IRS1
LLSVRHLSLIAYMLL	AAA85886.1	UL147Toledo
MPLSGGRHYHPGTFD	CAA35392	UL77
GVQLSPAFYYEALFL	CAA35407	UL48(pp212)
RRLAILCLLSFMLPI	CAA35279	US12
SKFCRYVELICSREK	CAA35341	UL104
INDLLAYWPVIADIV	CAA35340	UL105
IVAAAYDDSKFCRYV	CAA35341	UL104
GIPLKEEHVAYVDRF	CAA35340	UL105
RCDPYQVYFYGLQCP	CAA35261	US29
TTTFFLGDMQLPADN	CAA35430	UL31
PRLENYFLCQVCLYE	CAA35362	UL88
ETVWCLDRDRGVLYY	CAA35293	US26
RENLLTLGQWELVLP	CAA35359	UL85
LCYGFGNNFFVRTGH	CAA35336	UL100(gM)
MRVISRARSACTWTS	P09724	US20
SVGASRDYVHVRLLS	AAA85887.1	UL148Toledo
ARSLAADYLCCDDTL	CAA35289	US22
VFGHTEGQVLLTMAY	CAA35386	UL70
YLQRSMRDDNWGLLF	CAA35314	US3
NNSTPLLGNFMYLTS	CAA35403	UL44(pp50)
SSSTDLRSNPYPIRW	CAA35420	UL21
CRVDADLGLLYAVCL	CAA35323	UL121
YEHASEGWRFCSRRE	CAA35264	US32
AAWLSTRRELVGGFS	CAA35420	UL21
QPEVCVLYVTPDLDF	CAA35367	UL93
DLTMTRNPQPFMRPH	CAA35357	UL83(pp65)
MYPVLLLTASPVPTP	CAA35420	UL21
IPIQYAAVDLTIKMW	CAA35351	UL78
SHDELMHTDYLLHIR	CAA35384	UL69
QRLWWEIQYSSGRLT	CAA35323	UL121
YPPSWSRTIPNRIRY	CAA35436	UL3
VQKIDFVDALKTLCH	CAA35360	UL86(MCP=majorcapsidprotein)
ISALSESCNQTCSCQ	CAA35461	TRL13
VSSHTSPAYDVSEYV	CAA35427	UL28
SDDVLFALDPYNEVV	CAA35390	UL75(gH)
EDLDRMEAGLSPYSV	CAA35424	UL25
GLYASENYNGNYELT	CAA35441	UL8
YYAYDLAMSFRVGTH	CAA35338	UL102

VPEVFCTRDLADLCV	CAA35428	UL29
HLVTDYGNVAFKYLA	CAA35407	UL48(pp212)
FRQRLFSRDVSWYHS	P09704	US28
FSRLHPKLKGTVQFR	CAA35336	UL100(gM)
PVEVRSHVRQHAHTM	CAA35397	UL38
RMRRATLPRALARAC	CAA35375	UL60
YGRSIFTEHVLGFEL	P16832	UL115(gL)
SGLVYRELHDFYGYL	CAA35265	US34
CFRVLPRPLELLDYL	CAA35367	UL93
SLGRYALWIYNIYSS	CAA35380	UL65
VMVRIFSTNQGGFML	P19893	UL122(IE2)
GHHEDENFYLLVTPK	CAA35460	TRL12
KKREDALLKQMRSEY	CAA35366	UL92
AKRPLITKPEVISVM	CAA35325	UL123(pp72=MIprotein=IE1)
AAAAEADRALREFLE	CAA35406	UL47
APVSADWFRFSGRSP	CAA35364	UL90
APVVGYDQLAARHFA	CAA35354	UL80A
DGELIYGSYLLYRKA	CAA35369	UL95
PSYAAEMTRLFLSHV	CAA35340	UL105
YGMMYTEAVYRQPQT	CAA35290	US23
LVLLTVGVSARLRFI	CAA35314	US3
DAAAEVLSWCGLPDI	CAA35338	UL102
IKAFSKNGLLWCEYV	CAA35289	US22
SHVEVQAYFKRLHEQ	CAA35340	UL105
RVYELVSETLFGQRC	CAA35408	UL49
MMEMPATMHPTTGAY	CAA74074	UL42rev
LYFWNNDVFRKLLRA	AAA85879.1	UL140Toledo
LIAWVEEMLRYVESK	CAA35407	UL48(pp212)
FGFVRQNVVFYLLSH	CAA35406	UL47
KLLRFVVNDGTRYQM	CAA35332	UL130(viralentry)
PVKYRTHRAAVFFHA	CAA35426	UL27
LSRPDEPCCTPALGR	AAA85880.1	UL141Toledo
PTAREGELFFFSKNL	CAA35394	UL35
KYAACVPQVRMDYSS	CAA35314	US3
FFMCLYVYSPTFLFD	CAA35406	UL47
CNEGVKAAWSLKELH	P19893	UL122(IE2)
CGFLVRIELGVYFFS	CAA35383	UL68
SITWLFATTLFIGYM	CAA35327	UL125
ANVYLCPGYLHFSAY	CAA35394	UL35
FSPLERSLSGLLRLC	CAA35386	UL70
RRVIHDSHGLWCDCG	CAA35264	US32
DIRTCRVDADLGLLY	CAA35323	UL121
LILDEQVSKRSWDTT	CAA35416	UL17
YNCTDCTQCNVTQVM	AAA85883.1	UL144Toledo
KNSVRHMSSFVSSDI	CAA35406	UL47
GSYEEGFVVIREQLS	CAA35407	UL48(pp212)

DGVLDAVWRVQGTFY	CAA35275	US8
FVERPETLQIFYNFH	CAA35394	UL35
YRSELLCTSAFLGYS	CAA35339	UL103
FGTLPASHAQYGFRL	CAA35435	UL2
VTTEHAGKYVLQRYS	AAA85895.1	UL153Towne
MISSVSTNDVRRFLL	CAA35284	US17
FQNVQSLHRRVRIFM	CAA35331	UL129(viralentry)
STMQPSTLETFPDLF	CAA35390	UL75(gH)
SVTFADTDTVWRNLF	CAA35361	UL87
GFVEGPGFMRYQLIV	CAA35395	UL36
IVLFFARRAFNKKYH	CAA35460	TRL12
LQDQYTGDVEAFYLI	CAA35459	TRL11
SAFVEARGLYVPAVS	CAA35372	UL57
PDANTAPEIWVSGHG	CAA35427	UL28
ILTRLEYLYKVDSQR	CAA35413	UL54
KARHMVEAIRTTVRG	CAA35424	UL25
IYVQLPSLREDYAQL	CAA35424	UL25
VQFGYHLGAFFGLCG	CAA35336	UL100(gM)
EEILRYDGLCRHQKI	CAA35341	UL104
RVHLCYWPEIVRSLV	CAA35447	UL14
KRPPETFSQPMHRAM	CAA35384	UL69
QSVLTWTHECNTTEN	CAA35417	UL18
TLNDSGAYTEHVYEC	CAA35434	UL1
TGQTMSFLRLLHGFL	CAA35341	UL104
VVVSVALIALYMGSH	CAA35433	TRL14
LGNEIRCMLLPRQYT	CAA35419	UL20
EAGRQEPETPRVSGR	CAA35367	UL93
FTIWQARVVRGDFAE	CAA35290	US23
TETYHLQRIYSMMIE	CAA35394	UL35
MGPFEGYSMSADRAA	CAA35351	UL78
GLRNYAPLREELGYV	CAA74075	UL43rev
YVLVTVNSLARAVVF	CAA35407	UL48(pp212)
KARLPAKAEGWLVSL	AAA85887.1	UL148Toledo
LTGVCYAFNVPSVLM	CAA35404	UL45
VDVYEFPSEYELLLG	CAA35413	UL54
THSTRYVTVKDQWHS	CAA35414	UL55(gB)
ARFADDEQLPLHLVL	CAA35367	UL93
SFDEAFLTDRLQQLI	CAA35407	UL48(pp212)
GALNLCLPLMQKFPK	P19893	UL122(IE2)
VSCVIDGGNMTSVWR	CAA35274	US7
QNVILPGVIFVSVGG	CAA35421	UL22
VGYRLLKEAIKIWEF	CAA35400	UL41
CVLPPLFHGPLAREN	CAA35359	UL85
IYWQKHSDLVYALTG	CAA35362	UL88
AACLTYLRETFEGTI	CAA35360	UL86(MCP=majorcapsidprotein)
CVRFASDSDFQTTFT	CAA35387	UL72

TTVWEKRRMESDTDF	CAA74073	UL41alt
RREIFIVETGLCSLA	CAA35390	UL75(gH)
TPTPTPLSEAMFAGF	CAA35356	UL82(pp71)
YEVINVTGYVGGNIT	CAA35461	TRL13
GLTEVLAYHLYGGDG	CAA35386	UL70
LMQLSKSNPVADYMF	CAA35392	UL77
LESDLAVWAALRGVP	CAA35426	UL27
ARVFCLSADWIRFLS	CAA35316	UL114
SLYYNETMFVENKTA	CAA35389	UL74(gO)
VCMLVPQDEAKRILV	CAA35368	UL94
QAVAPVYVGGFLARY	CAA35353	UL80
VVPHLHCLINPILYA	CAA35432*	UL33
YRRVVAYDADAAPEA	CAA35386	UL70
VYLPKDAFFSLLGAS	CAA35354	UL80A
RKNAPMESGEEEFLL	CAA35259	US27
NNTNGTHVNPWVCEE	AAA85896.1	UL154Towne
AFAYLPGEDKVYVLG	CAA35427	UL28
FTEEIQLHSLYACTR	CAA35366	UL92
AYAYSLVSGLLFSAM	CAA35340	UL105
ETALDAAAEVLSWCG	CAA35338	UL102
RDMRRVTFSNIATHY	CAA35269	TRS1part
YVATALYYVHFPFFS	CAA35282	US15
TKIPVLANRVLQYLI	CAA35363	UL89
QDSAVASGPGRIPQP	CAA35333	UL97
VQRLLDLTQMVMRLV	CAA35406	UL47
DLFCLPLGESFSALT	CAA35390	UL75(gH)
RFDLCTDSVLDYLGR	CAA35391	UL76
DLLKYMEFGIPLKEE	CAA35340	UL105
SARRLICSALYLLFE	CAA35339	UL103
YSLQFHDRCASYNDT	CAA35382	UL67
TPDPSRLRSINCGWE	CAA35447	UL14
HCGRFLRYHLLPLLL	CAA35445	UL13
SWSNPACDDGLFLYR	CAA35342	UL106
RVRILCGDTGTVYAA	CAA35428	UL29
SGPPAEQTWYADVVR	CAA35367	UL93
GPEAMDSQAPYPSED	CAA35289	US22
LEFTANNRVSFHGVK	CAA35403	UL44(pp50)
AGVALYAVAFAWVLS	CAA35351	UL78
YVGLLSVTTVFYTWC	CAA35280	US13
TFFHRVRILCGDTGT	CAA35428	UL29
VCNGIMFLHALHLGG	CAA35407	UL48(pp212)
SSDKASSRANGTISW	CAA35417	UL18
RLSERHRLFDLPVYC	CAA35340	UL105
RTPLCYASELCDESV	CAA35404	UL45
SRGHREFYVYDGYSG	CAA35338	UL102
PLHIGVAGLHTALMR	CAA35404	UL45

LDHLTLHLVPCTKCK	CAA35275	US8
DLELQETLATEYFAL	CAA35407	UL48(pp212)
FFTKNQTSTVCLLCE	CAA35411	UL52
KRYFRPLLRAWSLGL	CAA35423	UL24
TPVRRPMGEVAYYGG	CAA35261	US29
LSVISYCYYRISRIV	P09704	US28
EELTRLLAVWDDEPL	CAA35334	UL98
SLFPVYHVGKLLDAL	CAA35369	UL95
ADVNSVIRCLGGYCD	CAA35404	UL45
MVEAIRTTVRGDTVR	CAA35424	UL25
VALVFYYDSGMTPDV	CAA35404	UL45
MDDLRDTLMAYGCIA	CAA35395	UL36
TVMRFDQRLLEEGDE	CAA35385	UL71
STTSSRKGFLFTKQI	CAA35372	UL57
QVLRLFYDLRDLKLC	CAA35407	UL48(pp212)
EDLEQYLLVWSASLR	CAA35341	UL104
ECNTTENGSFVAGYE	CAA35417	UL18
SHFHEAALAVRYETP	CAA35282	US15
PCDLDIHPSHRLLTL	CAA35273	US6
ADSAVSHETLERYRV	CAA35340	UL105
LKRFKLLMEVYHGLV	CAA35285	US18
TVFDTTRLFEIAWSE	CAA35284	US17
VERLLATSDGLYLYN	CAA35333	UL97
HDLCWLFRRLFFPRE	CAA35416	UL17
CAPDFNMEFSSACVH	CAA35403	UL44(pp50)
YHVNSSDKASSRANG	CAA35417	UL18
EAARLHFTMFDSGVD	CAA35290	US23
YPTAVDLAKRALWTP	CAA35390	UL75(gH)
NSGRYSRRCFKENYF	CAA35460	TRL12
FFSSPTSSPSHSFYI	CAA35383	UL68
RLLIMGLFSLDRSYE	CAA35359	UL85
VPSVYMPPTVPYPDP	CAA35269	TRS1part
NTEYVAPPMWEIHHI	CAA35414	UL55(gB)
VEVRAARCPRTGLWI	CAA35367	UL93
WFISVFGHTEGQVLL	CAA35386	UL70
TFEVDMLLYSGKSCT	CAA35360	UL86(MCP=majorcapsidprotein)
RFFVPEGLVEFEAQP	CAA35405	UL46
VIWTVVWLKLLRDAL	CAA35435	UL2
ESVRRFVLRHMEDLP	CAA35404	UL45
AALPGEDEAWIASKN	CAA35430	UL31
FRQMHAAGTTTFFLG	CAA35430	UL31
KKLLLLDVPRLENYF	CAA35362	UL88
SQLSERVAYHLKLRP	CAA35399	UL40
RERCSFVNRRITRPR	CAA35405	UL46
TRRNAVDLDRPPLWS	CAA35445	UL13
PLHGVVGGFAAGHCG	CAA35338	UL102

SVADNLGFEPSVVAP	CAA35424	UL25
GALVDFDFLRLPRGG	CAA35391	UL76
LTTFVKHIDAAVFKT	CAA35406	UL47
AHRAKDAAHSAWILI	CAA35443	UL10
CHKICYAVDMSDESY	CAA35333	UL97
PLWDYLDSLLFLDEI	CAA35389	UL74(gO)
QLVLADLLRECTSPL	CAA35411	UL52
ALWAPVAGSMPELSL	CAA35278	US11
VMEAGGQMIHKKTKK	CAA35269	TRS1part
VIREQLSYLMTGTVR	CAA35407	UL48(pp212)
LHAETTRTWRWAQRG	CAA35449	TRL1
LSFSIVAAALWKVDY	CAA35323	UL121
RRICHLPTLYQLSFG	CAA35368	UL94
HDTFLEVVDVFGMRQ	CAA35407	UL48(pp212)
LAQMDNFSISQELLL	CAA35392	UL77
TTRLFEIAWSEADLL	CAA35284	US17
MRDYQRPMVQYDDYW	CAA35395	UL36
RHSYLKDSDFLDAAL	CAA35390	UL75(gH)
VGYFDSLSSLYLRGQ	CAA35427	UL28
LVFDQQGEDAVVRRC	CAA35385	UL71
GPPIPRPATRSSDPS	CAA35415	UL15
PFDHMPAADFRDLLN	CAA35289	US22
QSATRGDSDMFDGVV	CAA35362	UL88
WKCHALYAEWDGKES	CAA35264	US32
TSGLLGASMDLCFGV	CAA35334	UL98
YAETFSPLGSFTRLG	CAA35362	UL88
VCRQCGHCLNLGKEK	CAA35408	UL49
LLGTVATSFCHRVSD	CAA35430	UL31
FSSMCAGFVIGEEDR	CAA35348	UL110
FNMPYFQISIFPISP	CAA35329	UL127
RVLGYSTPTIYMTNL	CAA35432*	UL33
PDMASCHFGECDMPV	CAA35404	UL45
LKIVRLICVLLFHRG	CAA35352	UL79
GTHKYVLERDDEAVL	CAA35338	UL102
QTEKWHNVDWISKQP	CAA35296	IRL14
WEFPCLRLHDGRLFL	CAA35400	UL41
GDLHRFLFGVDLRLP	CAA35408	UL49
ILYQYADNDDYGLYV	CAA35341	UL104
LGHKLFLGYYAMALS	CAA35284	US17
ATLRLLIQDGMYGRG	CAA35323	UL121
VVCTLLDYKTFGTRT	CAA35426	UL27
VKNTNIPLGIHAVWA	AAA85895.1	UL153Towne
LKWIGPHTRVKRNVK	CAA35403	UL44(pp50)
LNDIERFLCKMNYVY	CAA35371	UL56
RPGSGGWPEHAEAQW	CAA35312	US1
PAWKTVRAFSRAYHH	CAA35264	US32

IPKKPHYTKLPKYDP	CAA35433	TRL14
HNPDLSSVLEEFEVR	CAA35431	UL32(pp150)
RVMHPHAFGMRAVSQ	CAA35266	US33
GVSGLARHTVFELCR	CAA35386	UL70
GRCQMLDRRTVEMAF	CAA35390	UL75(gH)
LNITNSGRYSRRCFK	CAA35460	TRL12
FSLPAFVNPRHQYYF	CAA35334	UL98
VKEFCLRHQLDPPLL	P16832	UL115(gL)
SPTYHESQMINKRVK	CAA35453	TRL4
CEEDLDGDCRQLFPE	CAA35333	UL97
WYARDPAVTLSQLFP	CAA35392	UL77
TFRPPPCGTVPSMTC	CAA35389	UL74(gO)
VRLFFSSLFEYRKHN	CAA35443	UL10
TEFSEIFGIPQGLFQ	CAA35406	UL47
VPEDEWQVFGTEAGG	CAA35338	UL102
YNARLTRGYVRYTLS	AAA85887.1	UL148Toledo
ELYRLPRLSIASARW	CAA35404	UL45
GETLMELKDNLTLWT	CAA35417	UL18
IHNKRCTDLDFGDLL	CAA35340	UL105
SWLERHCPPLDQELI	CAA35397	UL38
RFSNLEQRVARLLRG	CAA35367	UL93
RVVEQVLRLFYDLRD	CAA35407	UL48(pp212)
QLSYLMTGTVRDNVP	CAA35407	UL48(pp212)
LTSSIAHWQTLVDVA	CAA35405	UL46
PADEEAEDSVFTSTR	CAA35424	UL25
LFVCTLPLWMQYLLD	P09704	US28
NQTSTVCLLCELMAC	CAA35411	UL52
MAWRSGLCETDSRTL	CAA35368	UL94
TCAALGIWCLAGSSA	AAA85872.1	UL133Toledo
FTRNDEFLYCHTRYE	CAA35290	US23
RILLSHDELMHTDYL	CAA35384	UL69
RHRGTPESPRLTEVY	CAA35424	UL25
LCVRDYALRNADRVT	CAA35369	UL95
ECGQLGSSRLRWRDG	CAA35362	UL88
GNWSVTNLTESCINR	CAA35419	UL20
PYVVFMGRFSRVYAY	CAA35291	US24
AVSEFMKNTHVLIRN	CAA35389	UL74(gO)
HSPQWASLLQLHHGL	CAA35280	US13
CLAYRSLTRKKLEQH	CAA35458	TRL10
YNDPVAVFDFASLYP	CAA35413	UL54
HVDLDFGVVADLLKW	CAA35403	UL44(pp50)
WRFEDGGAAQRLSLS	CAA35447	UL14
LSRFNVGDFHGASWE	CAA35265	US34
FSTTVLPRVHGPRSS	CAA35293	US26
RCFGCVGARPGGCVP	CAA35310	J1I
HPGFALTAVRTDTFE	CAA35360	UL86(MCP=majorcapsidprotein)

GTGLGDGGCAGRRWX	AAA85892.1	UL151Toledo
FNYLDLSALLRNSFH	CAA35390	UL75(gH)
RRHIIPGAANGMPPL	CAA35395	UL36
EHEAIPKVASLFWTL	AAA85891.1	UL150Toledo
TQHRSPSRCFMAMGE	CAA35374	UL59
RPAFSLFPARPGCHI	CAA35358	UL84
STPSRIRKAKLSSPM	CAA35431	UL32(pp150)
LPLMFYREIKHLLSH	CAA35413	UL54
GARTRDFRCLNYTHR	CAA35386	UL70
RSLLAFRELLACEDT	CAA35428	UL29
TSSMTCDGITPDVIY	CAA35368	UL94
FVACNGVAWEHRLSS	CAA35281	US14
VLPSFFFSGSARFLF	CAA35422	UL23
PSPQYQHVATHALWV	CAA35444	UL11
EAEIAARLVSTYRDR	CAA35407	UL48(pp212)
SLGDQIWSPTPWRLR	AAA85880.1	UL141Toledo
RIFEHMYFAAVRASV	CAA35404	UL45
VGQDKLVRLARDLRG	CAA35428	UL29
TGGNDETAFFQDDDT	CAA35340	UL105
FGRLHCQVLRLITNV	CAA35356	UL82(pp71)
LQRYTAESLRLAPYP	CAA35384	UL69
NIISWSSFFDVLLSS	CAA35344	UL108
DRPSFYRAFRSGRFD	CAA35391	UL76
MSRFVFDPKADYGGV	CAA35313	US2
FEIQRSRHETGIFTF	CAA35352	UL79
YLSGRNQTILQRMPQ	CAA35332	UL130(viralentry)
HGIQTFSYGLDFRSQ	CAA35407	UL48(pp212)
REEYHGVYEHLAGLI	CAA35407	UL48(pp212)
SVCVKTGLVYEALYP	CAA35366	UL92
GGQMIHKKTKKPKGK	CAA35269	TRS1part
QVLFVRHVLMPRDVA	CAA35283	US16
VGPQVTELYERYQHE	CAA35407	UL48(pp212)
RLPYATWNFSQLHLG	AAA85880.1	UL141Toledo
VAHLWGVTPSVWASR	CAA35404	UL45
PLAFRLSDARTLGLD	CAA35311	IRS1
VAEEVHTVLLSWKVL	CAA35432*	UL33
SAGLGSFRLFQLIMR	CAA35404	UL45
RCGAYVATALYYVHF	CAA35282	US15
YMSQRLSSLEKDHLM	CAA35372	UL57
YDHRLAFDVILPCAA	CAA35408	UL49
LRQDIRHLVRSYADM	CAA35293	US26
RPDEILVRWEEVSSQ	AAA85877.1	UL138Toledo
AEDVVMFTCVMGKKG	CAA35395	UL36
LRNQQFMGYGTKNGL	AAA85891.1	UL150Toledo
EPSVVAPQHVEYLKF	CAA35424	UL25
CVFARSDEQKLHLPD	CAA35404	UL45

LIYHTTLLMLTPVVW	CAA35281	US14
LSFPSPTYHESQMIN	CAA35453	TRL4
GGLHSNYTNLTEAFR	CAA35419	UL20
CRVPYNECGVELPGG	CAA35289	US22
AVFFQYVKVQFGYHL	CAA35336	UL100(gM)
ATIGAGLYIGKHFTP	AAA85881.1	UL142Toledo
HHHPGPPHPPLSHPA	CAA35352	UL79
APLSCNVTQWGRYEN	CAA35439	UL6
EICMKVFAQYILGAD	CAA35325	UL123(pp72=MIprotein=IE1)
FWVLLSCPRLLEYSS	AAA85880.1	UL141Toledo
LFIIAFFSREPTKDL	CAA35351	UL78
RERMKHDPENVIYFR	CAA35295	UL132
VCDVEETILTPRDVE	CAA35425	UL26
LFDTITVRRVAEEWK	CAA35341	UL104
ELELKYLNMMKMAIT	CAA35412	UL53
AARALLADYAETFSP	CAA35362	UL88
DFVDALKTLCHPVLH	CAA35360	UL86(MCP=majorcapsidprotein)
DVVFASELCFYCSGR	CAA35362	UL88
VASALFVYFRYERPV	CAA35283	US16
SMAEYRSELLCTSAF	CAA35339	UL103
AVSGASGGFLLHRKT	CAA35362	UL88
VCLPPCLSPDMASCH	CAA35404	UL45
HFRRRSASLSFLDWP	CAA74075	UL43rev
DFLELVQRIPDYRSV	CAA35426	UL27
CLLAWLAFPDVQGQC	CAA35285	US18
FLHTQLVMVPFVPHA	CAA35430	UL31
NGNILFSLGTLYGHR	CAA35352	UL79
RHWGAEAYEPLIRII	CAA35352	UL79
SNYTNLTEAFRFTPA	CAA35419	UL20
NRKASGTGVAAVGAY	CAA35363	UL89
KDAFFSLLGASRSAV	CAA35354	UL80A
THVKTSAGEEMFEAL	CAA35360	UL86(MCP=majorcapsidprotein)
PRVYYQTLEGYADRV	CAA35278	US11
VLFYHTPDQNHIEQP	CAA35363	UL89
LQLHKTHLASFLSAF	CAA35390	UL75(gH)
FAMSEADSLEMLLDK	CAA35385	UL71
LLSHDAALFRATLKR	CAA35334	UL98
YLIHAFQIDFLSQTS	CAA35363	UL89
GVVYGYDPAMDAVYR	CAA35395	UL36
GQLSLSTFTMSTVGF	CAA35404	UL45
CTRCFRTHLCDLGSG	CAA35366	UL92
RVLLQEHEHCLLNGS	CAA35320	UL118
DEIYAVLRRDGGALP	CAA35367	UL93
TYLCDDIHAIRFRVS	CAA35363	UL89
TMRKLKRKQAPVKEQ	CAA35389	UL74(gO)
KKTQSTTTPYFSYTT	CAA35389	UL74(gO)

TWTLFVACNGVAWEH	CAA35281	US14
MDTVSATKFYEAFVS	CAA35334	UL98
LQRQREEYHGVYEHL	CAA35407	UL48(pp212)
LPCLQFWQKVCSNAL	CAA35372	UL57
GSIFSWRDGNEALTT	CAA74075	UL43rev
VVTKKDNQCMTDYDY	P09704	US28
DSAGGGDGGGAVLMQ	CAA35392	UL77
TTETNMTTARESSVH	CAA35295	UL132
YSVSSDAPSSFELVR	CAA35424	UL25
LRKQLLYGVCKTLFD	CAA35341	UL104
RFLTSKVNYSNDMDN	CAA35322	UL120
SATKFYEAFVSGCLP	CAA35334	UL98
QAMQHAGFQVRAASV	CAA35445	UL13
CSHNLSIILYEEESQ	CAA35318	UL116
VPGTESLERFLTQLW	CAA35341	UL104
DAQEDCLYELASDLA	CAA35428	UL29
RHEYTACIRMLSGDV	CAA35426	UL27
SENGNLQVTYVRHYL	CAA35315	UL113
KQLVLFLRACLLKLH	CAA35361	UL87
YFTTLKQYLRNLAFA	CAA35417	UL18
SYSCGEGALPALGRY	CAA35407	UL48(pp212)
LEADPTAREGELFFF	CAA35394	UL35
PPNEHRFFSLRTRQT	CAA35387	UL72
MLLRYITFHREKVLY	CAA35444	UL11
FMARAEAALKDLYAA	CAA35426	UL27
QRALAVFDVLSRENL	CAA35406	UL47
HFGLLCPKSIPGLSI	CAA35357	UL83(pp65)
YRVCILGVEARLRPY	CAA35375	UL60
NNWDGWDAFTIWQAR	CAA35290	US23
AFVFPTKDVALRHVV	CAA35357	UL83(pp65)
LHDCLALHLPELTFE	CAA35358	UL84
LITLVIWTVVWLKLL	CAA35435	UL2
WLGCAAGPATGTAAG	CAA35311	IRS1
RFMRQRYLLATRLAD	CAA35372	UL57
VYHGRRLGRDPYSRR	CAA35436	UL3
NCYTDGDLLRTAMLL	CAA35279	US12
VYLDASNNPCNYSSF	CAA35434	UL1
LLLGYVLARTVYRVS	CAA35275	US8
QLLTRVHNHILNGFL	CAA35407	UL48(pp212)
RGIFLITLVIWTVVW	CAA35435	UL2
VFPTATLKSRPGFPC	CAA35283	US16
IEKANIPLGIHAVWA	CAA35433	TRL14
AMLTACVEVWARELL	CAA35338	UL102
VQYDTFLSNEYRTGI	CAA35336	UL100(gM)
RVLRCYLQHQLQSIS	CAA35424	UL25
RRGRVKIDEVSRMFR	P19893	UL122(IE2)

MGMPHYLMYSHTNNE	CAA35259	US27
LGRYETVWCLDRDRG	CAA35293	US26
FLNIGLRRCNFITVP	CAA35422	UL23
MAAVAGRDDDTFVFM	CAA35364	UL90
CWLRRTAIVMRVYGL	CAA35281	US14
FLVAVTADYQEHDLL	CAA35372	UL57
PEGLCAQDGLYLALG	CAA35311	IRS1
YVCVEGTEQLIENPC	CAA35360	UL86(MCP=majorcapsidprotein)
GWSRIIVLLPLMCMA	CAA35323	UL121
IVAAALWKVDYDRSV	CAA35323	UL121
RRWKKTVLRCGLEID	CAA35269	TRS1part
RVFGFVSKHVPLADS	CAA35340	UL105
QLRQQLTVRWQLFRL	CAA35445	UL13
TYGTYICSPNPGRLR	CAA35323	UL121
CCKHPGRFRFADEEA	CAA35295	UL132
AAAPEGITLFYGLYN	P16832	UL115(gL)
PGTLMLVMVYTTLTT	CAA35279	US12
SALFVMLRQLDDLIR	CAA35359	UL85
LTEVESRRWWWAVRA	CAA35291	US24
SKQPVKNLTMNMTEF	CAA35389	UL74(gO)
MRCFRWWLYSGWWWL	CAA35261	US29
LVSETLFGQRCVRVG	CAA35408	UL49
VTLGDGLDYHIGVKD	CAA35368	UL94
NDLLKFFVDRLCCET	CAA35293	US26
WLLWQHDKHGIVLIP	CAA35444	UL11
FTYPIYAVYGTRLNA	CAA35320	UL118
VRGRFSGREVPAWPA	CAA35369	UL95
CEMYVTLQLRCRWYL	CAA35290	US23
IPASDDVLFFVDGCE	CAA35372	UL57
MSREAAGGVPERGTA	CAA35341	UL104
QNHAQFPHVLGASPR	CAA35372	UL57
VHPSLYIGFPCGIPG	CAA35375	UL60
YLPKDAFFSLLGASR	CAA35353	UL80
FSVSFVFTLECGRCL	CAA35263	US31
TIFSPEDDSSCILCQ	CAA35368	UL94
IRCGKVNDKAQYLLG	CAA35330	UL128(viralentry)
AQVLNHAVCLDAELH	CAA35386	UL70
PQIYARSLAADYLCC	CAA35289	US22
GRGAEEVTWLLNDSD	CAA35312	US1
NLVLQEIETDEDFKP	CAA35423	UL24
LAFEARFADDEQLPL	CAA35367	UL93
CSGLTYLAGNNPTRW	CAA35285	US18
KALHHPIGGLFWVGR	AAA85894.1	UL152Towne
CLDRDRGVLYYLAHS	CAA35293	US26
VRSKELPSDHESLEA	CAA35326	UL124
GSSSSQRLVGEFMVR	CAA35424	UL25

LHRTPSAAFAVASTR	AAA85892.1	UL151Toledo
ALVFSANSVLYQHRL	CAA35372	UL57
GTVERFVDILQKDTF	CAA35340	UL105
TVYPTYDCVLSDLEA	CAA35392	UL77
VYTASAARAALQWLD	CAA35405	UL46
NNLYEEATSSYAIRS	CAA35412	UL53
PVYDLQRYTAESLRL	CAA35384	UL69
RYETFLRVMGDFQGI	CAA35290	US23
YLYNTLKTEVFALMI	CAA35405	UL46
CLLQRTVTTYVCRLP	CAA35348	UL110
VLAYHLYGGDGATAA	CAA35386	UL70
GCWGLYAAILCLDKV	CAA35404	UL45
RACAPGPLVHPSLYI	CAA35375	UL60
AAVFFHATFMARAEA	CAA35426	UL27
DQTDAVLFFDSPENV	CAA35413	UL54
SDDDDFFVYVEEIEP	CAA35293	US26
LRYYHQDSWRDMLHD	AAA85875.1	UL136Toledo
ETVRRPFFSDAPLPY	CAA35358	UL84
GFQQVSTGPECRNET	CAA35332	UL130(viralentry)
RFIVQYLNTLLITMM	AAA85887.1	UL148Toledo
EREAVTALFQRMVMT	CAA35408	UL49
VQYDDYWNAVMLYRG	CAA35395	UL36
ENVIYFRKDGNLDTS	CAA35295	UL132
RYSQRTTQCVAAHLL	CAA35370	UL96
FQVRNAFMKVKPVAQ	CAA35394	UL35
RHLELGVVIAICMAM	CAA35275	US8
RVYQKVLTFRRSYAY	CAA35414	UL55(gB)
HLLEQRAAVQQELQR	CAA35370	UL96
MQLCSHSISSQRHVA	AAA85891.1	UL150Toledo
LPDVSSLCAAAAATA	CAA35340	UL105
IQGATYQLSIVRQAM	CAA35445	UL13
MLFFQRYAPAFVTGY	CAA35413	UL54
YECDLSCNITTYNEY	CAA35434	UL1
VRVGLALLIDDFRYE	CAA35428	UL29
RSRSALGPFVGKMGT	CAA35395	UL36
KQCHTQLAFYNKHII	CAA35387	UL72
HRDNGQEENYYVTVL	CAA35433	TRL14
KTFFIYNVSESSGGT	CAA35439	UL6
CVFEHEFEKIKRPIF	CAA35344	UL108
LIWVQCLILMSVCAF	CAA35288	US21
VSWRPESPSPDGTPS	CAA35404	UL45
LCELMACSYYDNVVL	CAA35411	UL52
HLFVTDKRFLNRELG	CAA35408	UL49
SSGWQDRLNYTVINS	AAA85881.1	UL142Toledo
QSLIHNEEPATFFCE	CAA35368	UL94
IRLEPFQKNLLIHVI	CAA35363	UL89

YALALFAAARQEEAG	CAA35390	UL75(gH)
SSDEDESGRPRRIAN	CAA35289	US22
TERQSQLPEKYIGFY	CAA35311	IRS1
GKLFARGSIVGNMSR	CAA35313	US2
TPGRGRRGGEGYRRL	AAA85880.1	UL141Toledo
HLLPLLLCRLPFLLL	CAA35445	UL13
VMEACVFEHEFEKIK	CAA35344	UL108
VVFVHHVVKYSIMAD	CAA35404	UL45
TGAVYACDVRDDRYI	CAA35423	UL24
AQTYTTGTLTRYSQR	CAA35386	UL70
MAFAYALALFAAARQ	CAA35390	UL75(gH)
ARRLLPELDREQWER	CAA35311	IRS1
TTVMTERQSQLPEKY	CAA35311	IRS1
MRRHANDDFYKAHCT	CAA35388	UL73(gN)
CNVTQWGRYENGSTP	CAA35439	UL6
GWSWIASPWKGHKPF	CAA35431	UL32(pp150)
ATITFGRITCCHPLA	AAA85882.1	UL143Toledo
LLVVFFVIYAREEEK	CAA35458	TRL10
VSTNTALISTPWLTN	CAA35460	TRL12
FWSIFTVCFLCYLCY	AAA85880.1	UL141Toledo
DTELDTVLLMHFFYT	CAA35406	UL47
ASVDLCKSGLPRCEW	CAA35404	UL45
VILTQLETLSRPDEP	AAA85880.1	UL141Toledo
RDADRDNYGRCVRHA	CAA35430	UL31
YVLERDDEAVLARLF	CAA35338	UL102
GRRLSTELLDVMQKY	CAA35386	UL70
PLLGNFMYLTSSKDL	CAA35403	UL44(pp50)
MRELAWRRVADDSHD	CAA35312	US1
HVCTTVDYGLTSRTA	CAA35340	UL105
EHVHRWSYLCPPEQV	CAA35316	UL114
NVTEVHGEVACFRND	CAA35396	UL37
LCCPEPLRFVGSICT	CAA35422	UL23
KQRSRTLTFVSVLLI	CAA35432*	UL33
WVLPPPLPPRPPHLI	CAA35419	UL20
GCSWKTFFIYNVSES	CAA35439	UL6
QYEFMGLIFTVNVDS	CAA35430	UL31
GRASVVFVHHVVKYS	CAA35404	UL45
YTGDVEAFYLIIHPR	CAA35459	TRL11
YVYSPVVESLYLVSR	CAA74075	UL43rev
RGQRPTGHFHLFFVV	CAA35343	UL107
RQHSSPAFQPMLYNL	CAA35394	UL35
EFEGDFARYRSSQQK	CAA35407	UL48(pp212)
QFCRGPLLYVHRRCC	CAA35361	UL87
LPTNHEREILDLMRH	CAA35385	UL71
ALHLIGTVSHATCRY	CAA35428	UL29
GKITTETYHLQRIYS	CAA35394	UL35

ARFIKDNFSEPCFLH	CAA35413	UL54
ADTDTVWRNLFYVYY	CAA35361	UL87
RCQQCCSIGKIFYRT	AAA85883.1	UL144Toledo
TDPEHLMMNVNPLRL	CAA35340	UL105
PTKPVTSMWNSSWTS	CAA35294	UL131
TRSLTSIQNLLCAIP	CAA35415	UL15
NFIETRSLNVTRYRR	CAA35386	UL70
ELVGGFSKIIYIQNS	CAA35420	UL21
ALGAGFRVFVYDLAN	CAA35311	IRS1
NHRGMLRLLSVEEIC	CAA35424	UL25
RVAFGCSWKTFFIYN	CAA35439	UL6
RHRDHFHTMRDYQRP	CAA35395	UL36
QYSTISTVEEYVRSF	CAA35386	UL70
LHHGLDLQRMHDYLS	CAA35407	UL48(pp212)
DLSIPHVWMPPQTTP	CAA35390	UL75(gH)
SQDHVQIVYGSTRIC	CAA35319	UL117
LDEGIMVVYKRNIVA	CAA35414	UL55(gB)
YLGPDLFETGAARSF	CAA35338	UL102
FHRAEGSFCGCEGRS	AAA85892.1	UL151Toledo
PQSICRFCIDRLRDI	CAA35438	UL5
TACIRMLSGDVQRLI	CAA35426	UL27
RLQYVYLIIVYDYDG	CAA35405	UL46
NTSAWTLHAAGTESG	CAA35407	UL48(pp212)
PVVRVFVDMWDVAAI	CAA35404	UL45
LRCRETSAMWSFEYD	CAA35333	UL97
GYNSKFYSPCAQYFN	CAA35360	UL86(MCP=majorcapsidprotein)
IRQSVQSSTLIRVLF	CAA35363	UL89
IALMAYTYNRMVRFI	CAA35259	US27
KVLLTMVWGAAPVIM	CAA35432*	UL33
DRFRAEMLNNWDGWD	CAA35290	US23
VYRHDEVDRWIRHAA	CAA35360	UL86(MCP=majorcapsidprotein)
TYALVSKDLASYRSF	CAA35390	UL75(gH)
TYLRETFEGTILDKI	CAA35360	UL86(MCP=majorcapsidprotein)
MTLFCRTANSTAGYV	CAA74073	UL41alt
EQRQLLGTVATSFCH	CAA35430	UL31
LKGENAPFPHLRWPV	CAA35423	UL24
TQQCEMVTEGYTQPQ	CAA35407	UL48(pp212)
PWESAPQPPRLRMTP	CAA35406	UL47
SYGNSVDNLRRLHYE	CAA35275	US8
IFCNQNYTCRVTHGN	CAA35417	UL18
SMSFVYLHSVESYSL	CAA35382	UL67
RCHENGIYYGTRSMR	CAA35422	UL23
EHTLNDLAFLVGVEL	CAA35424	UL25
FLINLTSHYSGIYYF	CAA35442	UL9
RSAAVGYYDEEEKRR	CAA35431	UL32(pp150)
GGWREDVLMDRVRKR	CAA35392	UL77

RLVELGFNHDTCAAY	CAA35406	UL47
ILVEGTATATEALYI	CAA35277	US10
FVIGVLEQAHFVVIG	CAA35427	UL28
GGSSSRTTSNSSRST	CAA35424	UL25
CPRGFELLPEFTEEE	CAA35419	UL20
YAELFFLLCSTSESS	CAA35430	UL31
VLRHCCFQNFTATTT	CAA35388	UL73(gN)
LATRSLAWVDCCRVA	CAA35282	US15
HNVTREVNVRKRAYL	CAA35371	UL56
AQNRTQSSLLTLYLE	CAA35371	UL56
DFDFLRLPRGGGQVW	CAA35391	UL76
EDMATFRTEKQWQQD	CAA35274	US7
NRNYTLHVNHSCDYS	CAA35318	UL116
WTSCTSLSPCSTSCP	P09724	US20
HTCYQTAFVRVRTRL	CAA35372	UL57
LCAPVVRACRASSFG	CAA35310	J1I
VDVLVHGNVDAFLLI	CAA35360	UL86(MCP=majorcapsidprotein)
ISTSTVSGTRNTGNN	CAA35318	UL116
DSAAKIQERYAELQK	CAA35363	UL89
GRVSEAEALLNQQCE	CAA35392	UL77
RPVSRGYGVSCASRT	CAA35361	UL87
KQVEDSIYFTFNKVF	CAA35366	UL92
ALKVTCNAFYGFTGV	CAA35413	UL54
LEEFEVRCVAVARRG	CAA35431	UL32(pp150)
LNLFLSMSLCVPPYF	CAA35268	US36
DDVSREIAAWEGPVA	CAA35392	UL77
IIFLAYVYHYEVNGT	AAA85885.1	UL146Toledo
LPGPCIASTPKKHRG	CAA35357	UL83(pp65)
TVRRVAAQVNNQSRS	CAA35424	UL25
SDAVVCELAFSFASV	CAA35372	UL57
GGEEGEDDVLATIRN	AAA85891.1	UL150Toledo
GFGIMDYVELATRTL	CAA35351	UL78
VDISHFLKKQHKKKM	CAA35381	UL66
VTLGGAGAWLLPRPE	CAA35368	UL94
LPGEDKVYVLGLSFG	CAA35427	UL28
ETAGTYTCVLGNETH	CAA35447	UL14
TFLCCDKFLLPVGTV	CAA74075	UL43rev
MGMQCNTKLLLPVAL	CAA35434	UL1
DDGDERLFRDPLTTY	CAA35426	UL27
TPCPNGTYVSGLYNC	AAA85883.1	UL144Toledo
LSMSLCVPPYFIHPT	CAA35268	US36
YLVLPNCCQVSVDRS	CAA35429	UL30
MPLGEIEGAEDKTFF	CAA35428	UL29
TMARKIGEYLLEQGF	CAA35413	UL54
ERYDTDYLLRRLRLY	CAA35408	UL49
EMMVRDVPKMVFLIS	CAA35389	UL74(gO)

GEDEAWIASKNVQYE	CAA35430	UL31
RDYLRFPTRLEFIPL	CAA35407	UL48(pp212)
SASSSSRICPLSNSK	CAA35276	US9
VTPSDLERLFAERRY	CAA35276	US9
GEVFQGDKYESWLRP	P16832	UL115(gL)
FDTNNAMGTYRCGAV	CAA35384	UL69
PDYEDMLCYSDDMDD	CAA35384	UL69
SVPAAVYTTVVMHHD	CAA35432*	UL33
RSNNTDTIFVSLLTG	CAA35419	UL20
ITLLVLFIVYVTVDC	CAA35313	US2
HRFFSLRTRQTLVIG	CAA35387	UL72
FDARADLAVYHRNQW	CAA35408	UL49
LRLPRGGGQVWSVVP	CAA35391	UL76
VSFFIKSVFVFPYLV	CAA35429	UL30
DLERLFAERRYLTFL	CAA35276	US9
STWLAQCAERPLPGN	CAA35392	UL77
NYPELCYLVDVLVHG	CAA35360	UL86(MCP=majorcapsidprotein)
LSAQASVSYALRYDD	CAA35311	IRS1
LNISLENCAFCQSAL	CAA35390	UL75(gH)
PLLAYAYFRSVFYVI	CAA35372	UL57
GRVLAQLHRDRARVM	CAA35361	UL87
NNTHLALVGVIVFIA	CAA35440	UL7
GVSFIIYKFTYTTPP	CAA35327	UL125
ELATRTLLTMRLGIL	CAA35351	UL78
FLQDGVSFIIYKFTY	CAA35327	UL125
PPPRFPPRCSDAGTI	CAA35415	UL15
RSHPGFFSCRYHPST	CAA35342	UL106
ICANVEDYLQDARRR	CAA35269	TRS1part
SWAVTSNRLPNCSTI	CAA35439	UL6
LGAMDADEPLFVDDY	CAA35360	UL86(MCP=majorcapsidprotein)
AYQELCELADPLGDS	CAA35340	UL105
RYMYLFSVSCAGITG	CAA35419	UL20
TMYGGISLLSEFCRV	CAA35325	UL123(pp72=MIprotein=IE1)
CTSYGFFPGEINITF	CAA35417	UL18
YRRLSVTFADTDTVW	CAA35361	UL87
NAMGTYRCGAVSDLI	CAA35384	UL69
VELMITHFQRTIRVL	CAA35424	UL25
TRTHYLRQLCSMTEE	CAA35426	UL27
QLCLTEATSLLHRHR	CAA35406	UL47
RSVRLPYMYKMDQDD	CAA35386	UL70
NTQGCGYKYDWSNVV	CAA35430	UL31
EFCVLIAALYPEYIY	CAA35407	UL48(pp212)
VFFIVVVAGFASSEA	CAA35349	UL111
LTPQALVARGPSLAH	CAA35413	UL54
SRLTWRLTWLTCPAP	CAA35364	UL90
DVVEHWLHAQGQGQP	CAA35353	UL80

EIMEHMRLRPPPDYE	CAA35360	UL86(MCP=majorcapsidprotein)
DMVQAVEAVWQRLEP	CAA35407	UL48(pp212)
RDLPTVYLVSAIFRE	CAA35334	UL98
RYLWTPDPSRLRSIN	CAA35447	UL14
NRTRSAQVKTIYRVF	CAA35340	UL105
WKLLVVTQGQLRVIG	CAA35425	UL26
FSRVAVSLRRRALQV	CAA35384	UL69
VSLYIPEGFFGITFY	CAA35387	UL72
VATSFCHRVSDKITA	CAA35430	UL31
GSVTAGRALSYHVLE	CAA35361	UL87
ERRARRARRFCLDYE	CAA35384	UL69
HNSRTWDNVIKTVKN	AAA85895.1	UL153Towne
LPGVIFVSVGGGPPL	CAA35421	UL22
GSGPSLWRARMSAAL	CAA35409	UL50
AEVPVLDSSSSGGDS	CAA35369	UL95
LLLHVESFLRAAKDL	CAA35400	UL41
RLITETAGTYTCVLG	CAA35447	UL14
LFATTLFIGYMPIHC	CAA35327	UL125
SGCITRLASDSLCLF	CAA35436	UL3
SPDHLFSKWLDKHND	AAA85885.1	UL146Toledo
HLNQRLCCGWLALGA	CAA35311	IRS1
ISFNFFQSYNQYYVF	CAA35390	UL75(gH)
DMGLGYTSNTCVAYV	CAA35360	UL86(MCP=majorcapsidprotein)
SLLGCDPGASLRRLW	CAA35286	US19
DVQHLRRLNECIPMP	CAA35424	UL25
GVNESAFGLTHLQSC	CAA35399	UL40
VAHLLAATRRRLLRT	CAA35391	UL76
TVPRITCYHQLLGAL	CAA35395	UL36
LPRPLELLDYLRQSG	CAA35367	UL93
TVPYPDPAARLCRDM	CAA35269	TRS1part
SVCNQNVILPGVIFV	CAA35421	UL22
GLWCDCGDWREHLYC	CAA35264	US32
CRLVLFVDDVGLYST	CAA35351	UL78
RLTQIHDLLHVIETL	CAA35407	UL48(pp212)
YWESRTDHVPCFIFK	CAA35409	UL50
DVICCPERLIVLGKC	CAA35290	US23
DALLKQMRSEYGNAP	CAA35366	UL92
RFVDILQKDTFIERT	CAA35340	UL105
RLLRVFPSFVALNRL	CAA35391	UL76
ATEKIPFVENAVLKE	CAA35272	US5
DAADEVWALRDQTAE	CAA35431	UL32(pp150)
YVILTPLAVLTCGLQ	CAA35419	UL20
RACVIGYQGTVERFV	CAA35340	UL105
LRGNQRNRIRWWQHN	CAA35296	IRL14
PSMAQFWHGAIVLEY	CAA35397	UL38
AAYGHALWEGRDPPD	CAA35392	UL77

FALLHGIQTFSYGLD	CAA35407	UL48(pp212)
VVDFERLNMSAYNVM	CAA35336	UL100(gM)
QWFRSISRVERTTDN	CAA35340	UL105
PFSLAHLLDAIYNVL	CAA35318	UL116
GWFKAATAIVPRVSG	CAA35361	UL87
GVYQMWVSGATKKDL	CAA35319	UL117
CKKRYIGKVEGASGL	CAA35413	UL54
TRVFFSPCAPHVAFI	CAA35310	J1I
GNCCLDAPPVVRSPC	CAA35447	UL14
TNLYSTNFLTLTVLP	CAA35432*	UL33
VGFDRVPQYDFLISA	CAA35404	UL45
VLDSSSSGGDSGPED	CAA35369	UL95
MELHSRGRHDAPSLS	CAA35384	UL69
HVGHHLALRGSSLHL	CAA35282	US15
TVECVNDILDACSHP	CAA35365	UL91
ALRADMLEFGLRNCQ	CAA35404	UL45
SAATASSSSSSPPSR	CAA35428	UL29
GITQNDPFIRFHTDF	CAA35392	UL77
AAYCYCVFGTCSIGT	CAA35437	UL4(gp48)
SDEALTETVWLHDDD	CAA35386	UL70
AHGTPVAEDFMTRVA	CAA35334	UL98
SEAAVRLSRLSLDEV	CAA35413	UL54
HAAGTTTFFLGDMQL	CAA35430	UL31
TAPYLRDTLPFWSTL	CAA35279	US12
PTCPYGVDHQLFDDA	CAA35422	UL23
CIVADSLMEFVTRGM	CAA35422	UL23
LLKIYQAGRRFHYAV	CAA35293	US26
AVVGCSVFMIFLCAY	AAA85872.1	UL133Toledo
GAYFEWNIGGHPVTH	CAA35314	US3
VPTTQFCRGPLLYVH	CAA35361	UL87
ALDAYRARIAVEYVL	CAA35367	UL93
GRYLRSLLAFRELLA	CAA35428	UL29
SAQVKTIYRVFGFVS	CAA35340	UL105
VCMFITYTLGNEHPS	P09724	US20
NLDCDPEVMAVYEIL	CAA35372	UL57
IFMIVCVLWCVWICL	CAA35331	UL129(viralentry)
LCYLIYVQLPSLRED	CAA35424	UL25
RSSNTLQHMSKKQES	AAA85882.1	UL143Toledo
PRRVASAAWRGEARR	AAA85873.1	UL134Toledo
LLIPKSFTLTRIHPE	CAA35356	UL82(pp71)
LVDRACEFFYFDVSR	CAA35293	US26
PSYEEQESSSFFHSK	CAA35382	UL67
LPWTTVFAAFRSSYC	CAA35280	US13
RGAKSTALFASCYNT	CAA35363	UL89
VLENHVATHVLRGLL	CAA35361	UL87
RFWETPTLIMKEESA	CAA35387	UL72

GILALGSFSSFYSQI	CAA35399	UL40
PHACPHYAVPFTTPG	CAA35430	UL31
LEMLLDKFSTDQASL	CAA35385	UL71
DLRQLSPRDAWIVLV	CAA35311	IRS1
SPFESYVRWEETNVT	CAA35396	UL37
GFMFQRSAKSRWFDV	CAA35266	US33
REFVFYLNGTYTVVR	CAA35320	UL118
IYGYGTRPVPDLQCV	CAA35413	UL54
VQIVYGSTRICKSLA	CAA35319	UL117
FDEQSLLDLTVFAGT	CAA35384	UL69
AEDCWKPACPDEEPD	AAA85880.1	UL141Toledo
DAYARVCDRVLEDHL	CAA35406	UL47
VTSPNYPELCYLVDV	CAA35360	UL86(MCP=majorcapsidprotein)
AMVYIRRSCLVHSAC	CAA35426	UL27
LLGRLNVYEVARFSL	CAA35334	UL98
IQEFMITCLSQTPPR	CAA35390	UL75(gH)
PDRQIPKNITCANYS	CAA35437	UL4(gp48)
SLNVSSMSGQDYRFM	CAA35372	UL57
RTTTTTTTKKTTTTS	CAA35444	UL11
YVRAILESERRIREG	CAA35397	UL38
AAMDHSEFLTSFRRE	CAA35319	UL117
RQAFPGLDFEAAVFD	CAA35352	UL79
TAYKAFLWKYAKRLN	AAA85886.1	UL147Toledo
FPPSPPPSPGPMHMV	CAA35419	UL20
DSFDYLVERCQQSCH	CAA35351	UL78
HESQMINKRVKRKKL	CAA35453	TRL4
IPLTLVDALEQLACS	CAA35356	UL82(pp71)
LYYLAHSLDDFARHG	CAA35293	US26
DFVILVDRACEFFYF	CAA35293	US26
YVLARTVYRVSSAYY	CAA35275	US8
AYAVYHQGDMALMTL	CAA35399	UL40
PDLTLSSLTLYQDGI	CAA35358	UL84
NTASTTFVTSVFSTP	CAA35460	TRL12
NDPFIRFHTDFRGEV	CAA35392	UL77
FNGTTETCDLDGYMC	CAA35407	UL48(pp212)
IHHPKLQPGVGLWID	AAA85887.1	UL148Toledo
NTVSVLAFALPLDRV	CAA35356	UL82(pp71)
RAFVGNDHFILANAY	CAA35316	UL114
HEYLSDLYTPCSSSG	CAA35390	UL75(gH)
KESRGQSRNSVWHLL	CAA35334	UL98
YRARIAVEYVLIRAV	CAA35367	UL93
VMGLVDMDCEKSAYM	CAA35385	UL71
LAAGSVALTSLCHLL	CAA35447	UL14
TLHNLKLFCYLVSTA	CAA35407	UL48(pp212)
LERLEREWQEEAGKL	CAA35407	UL48(pp212)
ADPLGDSPQPVELWF	CAA35340	UL105

RLIGHLRHYLQNSFL	CAA35393	UL34
TCRCWSSSIVLYEHL	CAA35380	UL65
REQTRYSQRTTQCVA	CAA35370	UL96
MRQSYRYASGAVVRR	CAA35293	US26
RLFALIQLCRVLLPE	CAA35406	UL47
DAQRLASYLCCPEPL	CAA35422	UL23
RTVEMAFAYALALFA	CAA35390	UL75(gH)
VLQPLITKGGLCSSM	CAA35430	UL31
NDTNNTNGHATCVLY	CAA35432*	UL33
FWQIQSLLGYISEHV	CAA35338	UL102
LAVEQFISRFNSGYI	CAA35363	UL89
VQNAFTEEIQLHSLY	CAA35366	UL92
WSEWGNCCLDAPPVV	CAA35447	UL14
AHELVCSMENTRATK	CAA35357	UL83(pp65)
LCELADPLGDSPQPV	CAA35340	UL105
LNEHSQLSERVAYHL	CAA35399	UL40
DDYGLYVDWCVTVGL	CAA35341	UL104
DDYVTAVSGYLGEAA	CAA35361	UL87
HLYGGDGATAAAISD	CAA35386	UL70
KEQMALKVTCNAFYG	CAA35413	UL54
SVPCTLLTACFYVAM	P09704	US28
IKGNSTWDCLSVAWI	CAA35460	TRL12
RLELVASVFEHLTVE	CAA35365	UL91
KLCGAMISSSCSTTC	AAA85891.1	UL150Toledo
GALQCHECQNEMCEL	CAA35384	UL69
TFGLETWAMYTVGIL	CAA35399	UL40
LHTALMRLGFTYFAS	CAA35404	UL45
YRATCEDRTRTLVTR	CAA35459	TRL11
CKVSPPIQATRMLMG	CAA35404	UL45
WFEQRRLAEKERWHL	CAA35290	US23
NNDTRSNNTDTIFVS	CAA35419	UL20
LLCTSAFLGYSAVFL	CAA35339	UL103
TAYLHMFHPHTSPPF	CAA35267	US35
RLCASLRTAQTYTTG	CAA35386	UL70
EICEEHTLNDLAFLV	CAA35424	UL25
HVIRRLSERREHLVF	CAA35316	UL114
QWGVPTIIVAWITCA	AAA85872.1	UL133Toledo
RDCYERFVCPVYDSG	CAA35277	US10
ISFFLGILAYDSLMV	P09724	US20
CSCKCISGPCSRCCS	AAA85872.1	UL133Toledo
VISTSQHDTNRVTWF	CAA35373	UL58
SPLLLVFAPSLLWCL	CAA35280	US13
SLLRHLFQPCHAQRG	AAA85891.1	UL150Toledo
RAMVMGEDTVPYNKP	CAA35411	UL52
NPYAVAFQPLLAYAY	CAA35372	UL57
GRLGVGPQVTELYER	CAA35407	UL48(pp212)

FVGRPRHCRLEMLIL	CAA35416	UL17
LLELEIALGYRSQSV	CAA35417	UL18
MVDLNRRIFVAALNK	CAA35354	UL80A
RHEVFNERLPVFNFV	CAA35386	UL70
LGCELLAGGRVFHCD	CAA35334	UL98
YSLFGRPVSRRLSDL	CAA35404	UL45
RLYPKPRYDYTLASA	CAA35374	UL59
RTQMELDAADYAACA	CAA35449	TRL1
VFPSFVALNRLLGGL	CAA35391	UL76
PLFIVPDAYREHPLG	CAA35386	UL70
CHTETTIIRFKETNT	CAA35460	TRL12
VVLRELYRRVVSYCQ	CAA35411	UL52
PNCSHRLRECYHPAF	CAA35333	UL97
FQRMVMTKPYFVFLA	CAA35408	UL49
GVTVYVEVQGQYGLR	CAA35406	UL47
ISCVENCNLTRKCLH	CAA35339	UL103
ELWFPLYFEAECNRN	CAA35318	UL116
LKLSVVRLDNFSVEL	CAA35405	UL46
EVPAWPASSRTCLLC	CAA35369	UL95
RCRDGQLSLSTFTMS	CAA35404	UL45
NTGTEVDQCLAYRSL	CAA35458	TRL10
FTVNVDSLCVDAEQR	CAA35430	UL31
GFTVFSHAACGASLM	CAA35420	UL21
HRRRRRICHLPTLYQ	CAA35368	UL94
SDAPSSFELVRETGG	CAA35424	UL25
SACTWTSCTSLSPCS	P09724	US20
TTYNLLFHPPPFTTV	CAA35394	UL35
QSPAADFSVSEAWRF	CAA35369	UL95
TCRVQESAPGVLLVW	CAA35338	UL102
SDDIKRYVTEFPHYH	CAA35360	UL86(MCP=majorcapsidprotein)
HVETLRRFLRGDSCF	CAA35429	UL30
LTLVIPSWHVFASLD	CAA35356	UL82(pp71)
NKSSYTQIICMFIIF	AAA85896.1	UL154Towne
MRSEYGNAPVFGSGV	CAA35366	UL92
KWMSTLSCGVSVSVN	CAA35404	UL45
EENDSTTPVSNCRVP	CAA35315	UL113
FRLIERHGFFAVTLY	CAA35273	US6
NCQISSQTGSFVGML	CAA35340	UL105
ASDYTPTFDDLENIT	CAA35318	UL116
CVSKITFNSSCLYIT	CAA35403	UL44(pp50)
LLFLDEIRNFSLRSP	CAA35389	UL74(gO)
PVVGYDQLAARHFAD	CAA35353	UL80
SPHTTVAKAVFTTHR	CAA35387	UL72
RCFSCVPRDPCCRPP	CAA35310	J1I
CVVKDGVLDAVWRVQ	CAA35275	US8
AQMSYGACLLGEHGA	CAA35333	UL97

NTGNGSKCHAMCKCR	CAA35458	TRL10
VPATVPAALSILSTM	CAA35390	UL75(gH)
ITCYHQLLGALGHEV	CAA35395	UL36
TNVIPRESAEVPVLD	CAA35369	UL95
LILTESLAFCHCCLN	P09704	US28
CNHFLRDLLTDRFEG	CAA35446	UL12
EANGNHPEQICRSPP	CAA35424	UL25
RKKLEQHAAKKQNIY	CAA35458	TRL10
RPPPDYEETLRLFKT	CAA35360	UL86(MCP=majorcapsidprotein)
MDRVRKRYLRQELRD	CAA35392	UL77
PFCALTEVESRRWWW	CAA35291	US24
TLYQDGILRFNVTCD	CAA35358	UL84
VTYSITTAARRVSTS	CAA35389	UL74(gO)
PCFVVDAVVHPLALD	CAA35282	US15
RRSQTHYLEEMALQV	AAA85887.1	UL148Toledo
ECQDPVRRMLLDKEQ	CAA35413	UL54
RVSVSELEAVYREIL	CAA35338	UL102
ATRTTSPNALLPEWM	AAA85875.1	UL136Toledo
IGGTVFVAYHRDSYE	CAA35414	UL55(gB)
FLKRTVKLTRNKTKH	CAA35396	UL37
WNVVRCRGTGFRAHD	CAA35449	TRL1
LRTWRLLPMVLLAAY	CAA35437	UL4(gp48)
TVALQTPGRRLQPMV	CAA35311	IRS1
YVRRVANTITEFFRM	CAA35291	US24
TSEKATLVEHAEGMA	CAA35351	UL78
EYVFSGRSVLDSVSG	CAA35427	UL28
AAEDPMALYRQVLRD	CAA35291	US24
ITTYNEYEILNYFDN	CAA35434	UL1
EPCALSLGVPEDEWQ	CAA35338	UL102
NNAPFDMLNVVSYVC	CAA35363	UL89
LAELYVFVWYRGYEF	CAA35369	UL95
RTAIGGLHSNYTNLT	CAA35419	UL20
TPLAVLTCGLQEAYI	CAA35419	UL20
ATLVEHAEGMASEMC	CAA35351	UL78
HVVCAHELVCSMENT	CAA35357	UL83(pp65)
GHRTVAQMSKALKKT	CAA35406	UL47
YDWLCLTERFDPHEG	CAA35291	US24
GTMQLAQRLCELLMC	CAA35385	UL71
TVSLGITSLLTCVMR	CAA35405	UL46
NVSASTRCLLESVYT	CAA35405	UL46
MVLLAAYCYCVFGTC	CAA35437	UL4(gp48)
AYSAWGAGSFVATLI	CAA35458	TRL10
FPGESTFCLTAVSEC	AAA85891.1	UL150Toledo
PTPWRLRNHDCGTYR	AAA85880.1	UL141Toledo
YEKNTAITPYICRAL	CAA35340	UL105
LQQQYDWLCLTERFD	CAA35291	US24

LSTFTMSTVGFDRVP	CAA35404	UL45
VQHTYFTGSEVENVS	CAA35357	UL83(pp65)
SGLRPQETTEYTCSF	CAA35320	UL118
MELKDNLTLWTGPNY	CAA35417	UL18
AMVAFINQTSNIMKN	CAA35412	UL53
PWATRGIAAFLGFWS	AAA85880.1	UL141Toledo
SASWLSSFTPAAALP	CAA35415	UL15
SMKPINEDLDEGIMV	CAA35414	UL55(gB)
TLARDIVLVSAITLF	CAA35448	UL16
PVEEKKHPVPYFKQW	CAA35320	UL118
LLKHMIGISIGYVAH	CAA35363	UL89
VAILGIIFLAVVFTV	CAA74074	UL42rev
KLLMEVYHGLVWQLA	CAA35285	US18
TSDLSTFTTVYSTFN	CAA35460	TRL12
DALNCPLLNEPNVFS	CAA35430	UL31
VYALPLKMLNIPSIN	CAA35357	UL83(pp65)
YCVFGTCSIGTTTAP	CAA35437	UL4(gp48)
VNLNDTFQSIFPGTI	CAA35387	UL72
DKAFAMLTACVEVWA	CAA35338	UL102
KVRNIMKDKNTPFCT	P19893	UL122(IE2)
PGGDSDYHGLSGVDG	CAA35411	UL52
QMGAPTAERVARRRS	CAA35415	UL15
DVEFVPGESLKWNVR	CAA35313	US2
NVARCVFARSDEQKL	CAA35404	UL45
ITRYLAQFRGTMDDD	CAA35430	UL31
HAALDLAPAAAFGLL	CAA35282	US15
KMQVVFDPYGRQHGP	CAA35407	UL48(pp212)
LDVVVSEIRSAHFRV	CAA35314	US3
VPQNITTSQHATTTM	AAA85881.1	UL142Toledo
VGYGKVIRCIKTQER	CAA35371	UL56
TSLGVSLYIPEGFFG	CAA35387	UL72
CVSLGWPSQCIYVVG	CAA35428	UL29
GKSLHVCTTVDYGLT	CAA35340	UL105
MCRYTPRLDEIHKNT	CAA35444	UL11
PQTSDLECAKQYWQE	CAA35293	US26
SPRQQACVPRTKSHR	CAA35273	US6
GAHPGARCHVCLPGA	CAA35282	US15
VGFVAVAVTDEQCCL	CAA35338	UL102
QITDITSLVRLVYIL	CAA35390	UL75(gH)
TFDRHVLVRLFHKRG	CAA35392	UL77
RLVQALKRAMYSVEL	CAA35351	UL78
CYRLHKPTFISLNSQ	CAA35363	UL89
GAEEAIVYSNYTVER	CAA35341	UL104
ITLTFTAIVVVILRR	CAA35282	US15
MYPRVMHAVCFLALS	CAA35458	TRL10
TRMAIVRLSLNLFAL	CAA35358	UL84

VREHGVPIHADKYFE	CAA35413	UL54
REAVMSPTMVTIPPP	CAA35385	UL71
LMMICKKRYIGKVEG	CAA35413	UL54
CRRESLRTLPWLFWV	AAA85880.1	UL141Toledo
CAFCWLVLPHRLEQL	CAA35288	US21
HVATHVLRGLLSLTE	CAA35361	UL87
VHGNVDAFLLIRTFV	CAA35360	UL86(MCP=majorcapsidprotein)
FELLPEFTEEEEEKE	CAA35419	UL20
LASYACYTVFGLGSI	CAA35328	UL126
LLCARHLDELARYGM	CAA35290	US23
RGTVPLGWVFFVLCL	CAA35448	UL16
DLLREVQRNLTRTMA	CAA35284	US17
LYNAVKEFCLRHQLD	P16832	UL115(gL)
PKAAYAELFFLLCST	CAA35430	UL31
DELRYVKITLTEDFF	CAA35390	UL75(gH)
VGFATVALIAADRYR	CAA35432*	UL33
HINTVSCPTVMRFDQ	CAA35385	UL71
YGYTGIFDDTSHMTL	CAA35417	UL18
GLVKTLVECYVMHGR	CAA35334	UL98
MPAADFRDLLNFIRQ	CAA35289	US22
DLSALLRNSFHRYAV	CAA35390	UL75(gH)
TGFMPPLSLMVPTIC	CAA35284	US17
TASLRALAGCMHIHA	CAA35422	UL23
CSGGGLKNVVTTRYK	CAA35407	UL48(pp212)
SESEFIVRYNPEHED	AAA85896.1	UL154Towne
VSVYDELTASEMEEP	CAA35295	UL132
KRVASEGLRFFRLNA	CAA35338	UL102
RIPRQNLMNPSLMNQ	CAA35293	US26
LFYDLRDLKLCDGSY	CAA35407	UL48(pp212)
VGATDLRQLSPRDAW	CAA35311	IRS1
RLPALLRERVSVSEL	CAA35338	UL102
FRGQLLDRRAFLRSQ	CAA35432*	UL33
YYEALFLYMLDVATV	CAA35407	UL48(pp212)
LEQRVARLLRGDEEF	CAA35367	UL93
HCQENSETVALCTPR	CAA35360	UL86(MCP=majorcapsidprotein)
CAATWTATCVPRQTS	CAA35355	UL81
RLSFFDDYGNTKSYL	CAA35451	TRL3
LWMDWADVRSCIIKA	CAA35367	UL93
CLRHQLDPPLLRHLD	P16832	UL115(gL)
QKILRCLKTGETCVW	CAA35378	UL63
SRHAGWDGRGLLGPC	CAA35377	UL62
SPCLQPVRDRNRERN	CAA35447	UL14
IEVRLSRRIPDCVLV	CAA35391	UL76
AISYGRDLWHHETRE	CAA35351	UL78
VVGEFAMSEADSLEM	CAA35385	UL71
TRSLLLICGYYPPRE	CAA35277	US10

CQHPKKTPDPMIMFD	CAA35395	UL36
EGITLFYGLYNAVKE	P16832	UL115(gL)
ELSATLARDIVLVSA	CAA35448	UL16
HIYSDSLTFVAESIT	CAA35422	UL23
LLFWHDLCWLFRRLF	CAA35416	UL17
ERTPCEQAAYAYSLV	CAA35340	UL105
VLIEAALRQFVHDSQ	CAA35372	UL57
NETLYLLYNREGQTL	CAA35332	UL130(viralentry)
RLRVLWYVNSFWRSR	CAA35367	UL93
YRLPAQDVVTSWIEA	CAA35430	UL31
LDARSAFVEARGLYV	CAA35372	UL57
QFVHDSQQSVKLAPH	CAA35372	UL57
TRQLLVFAHSYALVT	CAA35360	UL86(MCP=majorcapsidprotein)
VMVSVLASTYTWLHK	CAA35281	US14
RWFPFRQSVFVERFV	CAA35375	UL60
AAELSHFLRAGVLGA	CAA35311	IRS1
MAQRNGMSPRPPPLG	CAA35311	IRS1
FKLRRGCRAPPTPET	CAA35426	UL27
FQKTTSSVDTSPPYC	CAA35386	UL70
GLKMPVTVWLPRSWL	CAA35358	UL84
FQSIHGAFSTTSSRK	CAA35372	UL57
HAMCKCRITEPITML	CAA35458	TRL10
PGPVVLLWCCLLLPI	P16832	UL115(gL)
RQGGAAFACTRQNLT	CAA35459	TRL11
GTQWSTNFFFSQCEH	CAA35261	US29
EGWLVSLDRFIVQYL	AAA85887.1	UL148Toledo
MTLDVYCCRQTSNNT	CAA35399	UL40
MLLDKEQMALKVTCN	CAA35413	UL54
ELYRDPQFQQINNFM	CAA35363	UL89
NFHEGKITTETYHLQ	CAA35394	UL35
LNVYEVARFSLPAFV	CAA35334	UL98
FYKVGNITLYTELHP	CAA35360	UL86(MCP=majorcapsidprotein)
QYPRGFGDVSGYRVS	CAA35276	US9
YPRLTTYNLLFHPPP	CAA35394	UL35
AGRALSYHVLENHVA	CAA35361	UL87
KEEHVAYVDRFVRPP	CAA35340	UL105
VERPQLLDTETISML	CAA35360	UL86(MCP=majorcapsidprotein)
CRLGTMCNLALSTPF	P19893	UL122(IE2)
RQTALREIALEERAL	CAA35386	UL70
ARDCFTHPESVAPAY	CAA74075	UL43rev
CPVYDSGTPMGVLMN	CAA35277	US10
TFLATTTTTTMMGVA	CAA35358	UL84
HAVNQRSCLRRPCGP	CAA35407	UL48(pp212)
RPVGYHPQEDESQLP	CAA35456	TRL8
QVIGDQYVKVYLESF	CAA35357	UL83(pp65)
RFIINYVGKWHMQTL	CAA35259	US27

RFLFGVDLRLPVLHP	CAA35408	UL49
IAVEYVLIRAVRDEI	CAA35367	UL93
LNLTTENSGKYYFKR	CAA35437	UL4(gp48)
QSLLGYISEHVTSAC	CAA35338	UL102
RLFEVREVCFLRTCL	CAA35338	UL102
WSSSIVLYEHLDARV	CAA35380	UL65
HCTSHMYELSLSSFA	CAA35388	UL73(gN)
DLASLALTAEFGLGC	CAA35423	UL24
YAYIYTTYLLGSNTE	CAA35414	UL55(gB)
VMTLAAMLYKISPVS	CAA35360	UL86(MCP=majorcapsidprotein)
RFTVISCNHFLRDLL	CAA35446	UL12
IEDLSANFRRQLAPY	CAA35318	UL116
LQQHVIGHRRGFATA	CAA35386	UL70
PISTVTETRWFLLNF	CAA35342	UL106
EPQVLDFTVRGDKLW	CAA35339	UL103
FILANAYLDTHYRET	CAA35316	UL114
AYVNRVRTDMGVRVQ	CAA35360	UL86(MCP=majorcapsidprotein)
DRGVLYYLAHSLDDF	CAA35293	US26
CQVCLYELDEDEMGE	CAA35362	UL88
NKIILGNYWLHRDPR	AAA85885.1	UL146Toledo
LSDVSEYRVEYSEAR	CAA35278	US11
NLYTTVNPLIEDVMR	CAA35406	UL47
QCVSISNWTMARKIG	CAA35413	UL54
YAALVGQDKLVRLAR	CAA35428	UL29
NGTTKIIERLYVRLG	CAA35448	UL16
IWTVVWLKLLRDALL	CAA35435	UL2
KWTFALLVVAILGII	CAA74074	UL42rev
ETDSRTLKQFLQEEC	CAA35368	UL94
MPPPLSAQASVSYAL	CAA35311	IRS1
IADYNDGGDMGSRFD	CAA35296	IRL14
IGDIFNMPYFQISIF	CAA35329	UL127
IFVSQFAFRAGAIPL	CAA35356	UL82(pp71)
IMLFLHHDSPHPPTS	CAA35356	UL82(pp71)
YKGKVHLADFMRDFT	CAA35411	UL52
ISVRYLYHADHQALT	CAA35405	UL46
KRIALAVATGQYVVC	CAA35426	UL27
DEPCCTPALGRYSLG	AAA85880.1	UL141Toledo
LRLLELPSSDAMTLT	CAA35283	US16
VSPERPAFMEHSRPV	CAA35456	TRL8
WSSFFDVLLSSRSCF	CAA35344	UL108
RLLAENLRGLNERLL	CAA35406	UL47
GTLYGHRLFRLAAFF	CAA35352	UL79
NKAVFMDAHGGIHVL	CAA35427	UL28
RDYMTYMNLAELYVF	CAA35369	UL95
STPWLTNCSATTYTT	CAA35460	TRL12
SRDTALAADIGYGVY	CAA35338	UL102

VLCMGGGTRLYIYEP	CAA35290	US23
ITVRRVAEEWKLHAA	CAA35341	UL104
QYATACAVAAATWPP	AAA85892.1	UL151Toledo
KIFAVYNAPNLHTLL	CAA35384	UL69
LVTLIAEHLADGALP	CAA35360	UL86(MCP=majorcapsidprotein)
LKLSTNQPPIFQIYY	CAA35412	UL53
DFRALVSPDRLVVGY	CAA35427	UL28
TRDLGTIIPTHASMG	CAA35386	UL70
RLLDFRLYAQGTYAV	CAA35372	UL57
KSEGDVPEEFMDYVI	CAA35419	UL20
AGAPGTYVNSSVTCW	CAA35322	UL120
DENFYLLVTPKNHTE	CAA35460	TRL12
YVLIRAVRDEIYAVL	CAA35367	UL93
GSFVGMLSHVSPAQT	CAA35340	UL105
CGMSSRLERAVKRLQ	CAA35293	US26
EGVIFGHSGLHLFTI	CAA35413	UL54
STSVIATTQKEGHLY	CAA35321	UL119
TPWGQLICCEESLES	CAA35293	US26
VKHIDAAVFKTVRDC	CAA35406	UL47
HGVLVAAWLVRGNFS	CAA35276	US9
VIMRLMKTVQQLHRI	CAA35291	US24
ERKEFLVRQYVLVDT	CAA35395	UL36
HFFTYHVNSSDKASS	CAA35417	UL18
DLLTLCLYENLVYLY	CAA35284	US17
NYSFFLTVLVPIVCM	P09724	US20
MDWRFTVMWTILISA	CAA35461	TRL13
EILRQLETTISTKYN	CAA35389	UL74(gO)
FLHFSVYTHRAEVVA	CAA35356	UL82(pp71)
FFFDIDLLLQRGPQY	CAA35357	UL83(pp65)
ATVALSVLSWWLMPP	CAA35286	US19
NLQARDASGLMFPII	CAA35406	UL47
LRAVAQVLNHAVCLD	CAA35386	UL70
RAWSLGLDTMARFII	CAA35423	UL24
VMRGYLYNTLKTEVF	CAA35405	UL46
QGIFECQYSADVLRD	CAA35290	US23
CPLLNEPNVFSLTVH	CAA35430	UL31
ESLAFCHCCLNPLLY	P09704	US28
STALFASCYNTNSIR	CAA35363	UL89
LSELLNKWVSQRRAV	CAA35413	UL54
IGMIGVVCFVFGVFI	CAA74073	UL41alt
VTFSNIATHYHYNAQ	CAA35269	TRS1part
MDPPLPSLHSPQWAS	CAA35280	US13
LFASKYKYVFEEVSR	CAA35319	UL117
LYCVYDSHFQRRPTT	CAA35264	US32
AAFESCCYDITEAES	CAA35438	UL5
SQSYMDRLQVSGEQY	CAA35314	US3

PSQSEVDCASLMETL	CAA35418	UL19
AAAFGLLQHGGPYLR	CAA35282	US15
GGNATYILPADCRYA	CAA35319	UL117
RVRAFRRFYHECSQT	CAA35333	UL97
QGQYGLRVPTTRFVE	CAA35406	UL47
YDVLFRGFAGQPPLR	CAA35338	UL102
PSHIHTMIFSPAWNL	AAA85873.1	UL134Toledo
RIDFRDLPTVYLVSA	CAA35334	UL98
DSVIDLLTCRWVRYC	AAA85872.1	UL133Toledo
IFVSVGGGPPLTESY	CAA35421	UL22
CEYLIDRRRHLVLKS	CAA35316	UL114
YIGPWYARDPAVTLS	CAA35392	UL77
ELFRAGLMKVYVRRR	CAA35290	US23
EFMDYVILTPLAVLT	CAA35419	UL20
KTTTSTTHHRHSNPK	CAA35444	UL11
LTDLNIKGRCVVGEQ	CAA35356	UL82(pp71)
SLAWPHDGVYLPKDA	CAA35354	UL80A
RAPSHRVMHQMQQTL	CAA35407	UL48(pp212)
TDTIFVSLLTGANGV	CAA35419	UL20
FEIAWSEADLLTLCL	CAA35284	US17
PYCRCKGKLGLRIIT	CAA35386	UL70
SLLAVTLTVALAAPS	P16845	UL22A
DLARNGNILFSLGTL	CAA35352	UL79
QRRLGRGLSGQRSHR	CAA35282	US15
HREKVLYLAIACFFG	CAA35444	UL11
VTSWIEALRDADRDN	CAA35430	UL31
GTCSIGTTTAPVEWK	CAA35437	UL4(gp48)
DIEYESYIPGALCLY	CAA35288	US21
VIYEVCMLVPQDEAK	CAA35368	UL94
VSSLFVAGHGETDFY	AAA85887.1	UL148Toledo
VPEAYLQLSFGEIVA	CAA35341	UL104
NRKNDVYQRRWKKTV	CAA35269	TRS1part
SAVLQTEALDAIMEE	CAA35407	UL48(pp212)
LRNMTLTLMRRVEGN	CAA35359	UL85
YVFIKSDPLFEDRLL	CAA35320	UL118
LTALLSAFCYAAPAT	CAA35282	US15
DTNTTTEPGLLDVFI	CAA35407	UL48(pp212)
KIGVLVVVCGFYFFL	CAA35451	TRL3
FAVASTREQYATACA	AAA85892.1	UL151Toledo
LCCEWYVVGLVGYYL	CAA35289	US22
AVAVTDEQCCLLLQS	CAA35338	UL102
FVGKMGTVCSQGAYV	CAA35395	UL36
VLGNTRRYFDLSVLR	CAA35404	UL45
MAMVLLLGYVLARTV	CAA35275	US8
ELTTEFDYDEDATPC	P09704	US28
PGALCLYMDLMYLFV	CAA35288	US21

MSSCRVRAFRRFYHE	CAA35333	UL97
RLRRETVRRPFFSDA	CAA35358	UL84
LAWPHDGVYLPKDAF	CAA35353	UL80
QSHNWHNHGNKWTLD	CAA35444	UL11
PRTGGRFIRRTASGT	CAA35415	UL15
LYEANPELRLPFKKR	CAA35428	UL29
ESKMWVLPPPLPPRP	CAA35419	UL20
MKTTPLPSPLLYECH	CAA35443	UL10
SSAAKIERIVDKVKS	CAA35340	UL105
RRRRQSINDEMKERT	CAA74075	UL43rev
CVLSYVESRFHNKFL	CAA35372	UL57
AVSYQGATVFEPEVG	CAA35413	UL54
RRRQWMREAAQAAAQ	CAA35333	UL97
FLGDMQLPADNFLTS	CAA35430	UL31
RLPFLLLFQRPQWAH	CAA35445	UL13
QTLHLRLVWPDGSYR	CAA35367	UL93
KIERIVDKVKSLSRE	CAA35340	UL105
PLGQMIVPPTPDIGF	CAA35387	UL72
TPDYEDMLCYSDDMD	CAA35384	UL69
AALRQFVHDSQQSVK	CAA35372	UL57
AGNRGKKTIITEYRI	CAA35294	UL131
MGLIFTVNVDSLCVD	CAA35430	UL31
HTMDDLVMVFHQLDY	CAA35397	UL38
WRKLFGGDDPGPTCR	CAA35338	UL102
KKMMWFVVLTVSFSY	CAA35381	UL66
PQHPANRYRHYEFQT	CAA35411	UL52
PMALYRQVLRDFKEL	CAA35291	US24
AVFHVICAVLLTLMI	AAA85875.1	UL136Toledo
MTVTFYGTRYIRDEL	CAA35280	US13
SFLFQAEESGPRRLE	CAA35365	UL91
TTVMSTLTKYAESDY	CAA35396	UL37
LSAERTVRWMLAFLE	CAA35407	UL48(pp212)
RSHVRQHAHTMDDLV	CAA35397	UL38
IYDRVPDCPKGRQHR	CAA35423	UL24
YHIGVKDSEGRWLPV	CAA35368	UL94
RLSLDEVKKYGVPRG	CAA35413	UL54
DSPDLRYYMPLSGGR	CAA35392	UL77
ARKHSETVLTVWMSG	CAA35333	UL97
GPSRPQSGPWLPARF	CAA35409	UL50
MNLPPPNEHRFFSLR	CAA35387	UL72
DEEHRTQMELDAADY	CAA35449	TRL1
QLHEANVYLCPGYLH	CAA35394	UL35
GMDEPPSGWERYDGG	CAA35353	UL80
QLGNECCPPCGSGQR	AAA85883.1	UL144Toledo
PRSPQLRGLIAALRR	CAA35408	UL49
VVKVARKHSETVLTV	CAA35333	UL97

EPLRFVGSICTYNFL	CAA35422	UL23
NITLKNAIRLRNGTM	CAA35461	TRL13
GNQTWYVNPFSLAHL	CAA35318	UL116
PMLRDRDHDDAPPTY	CAA74074	UL42rev
MRLARDESPRPTFFD	CAA35407	UL48(pp212)
SHTNNVNVGWFKAAT	CAA35361	UL87
FDEIRRRRQSINDEM	CAA74075	UL43rev
PQSGPWLPARFATLG	CAA35409	UL50
HILNGFLPVEDLKQM	CAA35407	UL48(pp212)
WYRGYEFPAPTPQAT	CAA35369	UL95
LSPRDAWIVLVATVV	CAA35311	IRS1
SSAVSSSSNNHHHHH	CAA35315	UL113
VYELAPTMKDFLRNG	CAA35395	UL36
LIGSKEELQHVWSNV	CAA35406	UL47
YFGHLNIKGLEKTFL	CAA74075	UL43rev
DPPRGPEWMSGVHLD	CAA35310	J1I
KLQPGVGLWIDFCVY	AAA85887.1	UL148Toledo
PEVISVMKRRIEEIC	CAA35325	UL123(pp72=MIprotein=IE1)
QFDWLEEPLLRKLVV	CAA35384	UL69
THKMCELGNYHQTTP	CAA35433	TRL14
RRLLPLFIVPDAYRE	CAA35386	UL70
TTSDSTCFLTRLNNA	CAA35363	UL89
DEETTVWEKRRMESD	CAA74073	UL41alt
ISCNHFLRDLLTDRF	CAA35446	UL12
FDPSRYLRQHGARCP	CAA35415	UL15
KAFMEANGNHPEQIC	CAA35424	UL25
FTIGTCGQVGPDVDV	CAA35413	UL54
ASIVATRVEDMATFR	CAA35274	US7
TFVHQSHNWHNHGNK	CAA35444	UL11
AKLSSPMTTTSTSQK	CAA35431	UL32(pp150)
PIPQRLHLIKHYQLG	CAA35359	UL85
SRERLLCFSPACFSH	CAA35355	UL81
VLASTYTWLHKTLLC	CAA35281	US14
TCKVILLNNTKNPDI	CAA35321	UL119
LLEEGDEEDEVTVMS	CAA35385	UL71
FIENLRFRSRRAFWQ	CAA35338	UL102
ARHVGEFNVLKVNES	CAA35409	UL50
ENYRQGPGFLLEKQH	CAA35457	TRL9
DTEVQRIEENLEGVR	CAA35431	UL32(pp150)
LFFVHARHDTLLPHR	CAA35400	UL41
VTLFTVNRTCDLLTP	CAA35398	UL39
RYASRREDSMSLGAR	CAA35341	UL104
RPRRKRCLVPEVFCT	CAA35428	UL29
SSCLYITDKSFQPKT	CAA35403	UL44(pp50)
TCDGFNYTVHKRCDR	CAA35461	TRL13
CDLQKRPPETFSQPM	CAA35384	UL69

SHAAVDRPRRTRRGD	CAA35457	TRL9
YTEKLEEIDSKPDTI	CAA35442	UL9
APLDHAQRQGLPDFI	CAA35360	UL86(MCP=majorcapsidprotein)
FPGFPPVPVYAVHGL	CAA35338	UL102
LIREQVVFTVCDVSP	CAA35359	UL85
CFGRYTVPFSGPSVP	CAA35381	UL66
PDILSVTCYARTDCK	CAA35321	UL119
GTLLPLGRPYGFYAR	CAA35311	IRS1
TGAARSFFFPGFPPV	CAA35338	UL102
PDVLLARMLKWYHWR	CAA35404	UL45
KQHKKKMMWFVVLTV	CAA35381	UL66
ALPVAAEDPMALYRQ	CAA35291	US24
FSQVTSSMTCDGITP	CAA35368	UL94
KELFFCLEPMEITRY	CAA35291	US24
TRQQNQWKEPDVYYT	CAA35357	UL83(pp65)
CAVMAPRTLILTVGL	CAA35399	UL40
ERTLEDALAVELVNE	CAA74075	UL43rev
SHRSVIYAMVATVTL	CAA35288	US21
NQWLLPAGVASCKFL	CAA35432*	UL33
MENWSALELLPKVGI	CAA35360	UL86(MCP=majorcapsidprotein)
LDLTQMVMRLVELGF	CAA35406	UL47
DRFRLIETPDENFLL	CAA35369	UL95
LELLDYLRQSGLTVT	CAA35367	UL93
TVNPLIEDVMRSSAG	CAA35406	UL47
PLVHPDHRAELCRRS	CAA35428	UL29
FRERPAGGDRRWLLP	CAA35338	UL102
IATTQKEGHLYTVNC	CAA35321	UL119
MLLVKCQELLMRLDR	CAA35359	UL85
SAVLSGVYSYLMTHA	CAA35366	UL92
IAASGAAPTPPVPFN	CAA35407	UL48(pp212)
LILMVCASESSTSWA	CAA35439	UL6
GSLFWHQNQRDFPKC	CAA35328	UL126
PQWALRQIADFALQL	CAA35390	UL75(gH)
PPPSPGPMHMVVCMP	CAA35419	UL20
DRPPLWSGSLPHLPV	CAA35445	UL13
EECMWKLVGKSRKHR	CAA35368	UL94
LSVLSWWLMPPPVAE	CAA35286	US19
TFLSNEYRTGISWSF	CAA35336	UL100(gM)
ELPSVGDRVFYVLTA	CAA35413	UL54
YEANPELRLPFKKRR	CAA35428	UL29
IFIAGNSAYEYVDYL	CAA35414	UL55(gB)
WYGRETRKRNSHKKV	CAA35452	TRL5
DFKKWLDGGFSTAVE	CAA35363	UL89
VPMEAVRHPLLFWRR	CAA35261	US29
RCLCYVPCGPMTQSL	CAA35368	UL94
DESSASSSGEAPEQQ	CAA35264	US32

DIFPTGQTMSFLRLL	CAA35341	UL104
YRPPYCLVSSPSPRH	CAA74074	UL42rev
LSRLKTTPNKHTQHK	CAA35443	UL10
VSSRVTLHGLAQRAL	CAA35367	UL93
LGPAVVAAPGPSVRY	CAA35426	UL27
AWMTWLSSRATGATN	CAA35284	US17
TAPKTLIEYSLPVPF	CAA35360	UL86(MCP=majorcapsidprotein)
RFCLDYEPVPRKFRR	CAA35384	UL69
DVPRLGAMDADEPLF	CAA35360	UL86(MCP=majorcapsidprotein)
ERNRHLGAFHLPAIR	CAA35426	UL27
PIISTRISTVNLYLS	CAA35406	UL47
TTTNSSTEGNWSVTN	CAA35419	UL20
SKSNPVADYMFAQSS	CAA35392	UL77
CNSENEDDTTVEGTS	CAA35289	US22
PPLVEKYWRMRTTHT	CAA35375	UL60
IEANSVTFKRQVELE	CAA35415	UL15
FEAAVFDETRAARLS	CAA35352	UL79
NFLTEEPFQRGDPFD	CAA35403	UL44(pp50)
LALHLPELTFEPTLD	CAA35358	UL84
AANKTLFKTIDEYLL	CAA35360	UL86(MCP=majorcapsidprotein)
DWIRFLSLPDHDTVL	CAA35316	UL114
SRFQRFWETPTLIMK	CAA35387	UL72
IIMAHNLCYSTLLVP	CAA35413	UL54
SHDGSDELILDAVKG	AAA85880.1	UL141Toledo
DMQLPADNFLTSPHP	CAA35430	UL31
TPPPADFQQPVFKTL	CAA35407	UL48(pp212)
QKQHRHGGSGGHNKR	CAA35353	UL80
LPPYLTVFTVYLLSH	CAA35390	UL75(gH)
VDAAGAPFDDDDYLD	CAA35386	UL70
FKRLHEQIRLSERHR	CAA35340	UL105
YQKLDALTELYRDPQ	CAA35363	UL89
LRRLRLYPPERLHAL	CAA35408	UL49
PCAQYFNTEEIIAAN	CAA35360	UL86(MCP=majorcapsidprotein)
VDSLCVDAEQRQLLG	CAA35430	UL31
EAMFAGFEEASGDED	CAA35356	UL82(pp71)
SPFLASGMMISAPLK	CAA35322	UL120
NNLDLGCILDHQDGW	CAA35384	UL69
VPPDEREEDTLREMA	CAA35424	UL25
ERLPKLRYDKQLVGV	CAA35353	UL80
LPSMIAFMAAVHFFC	CAA35336	UL100(gM)
QLDPPLLRHLDKYYA	P16832	UL115(gL)
VFVPHNRQGLKMPVT	CAA35358	UL84
EAGQIFVSQFAFRAG	CAA35356	UL82(pp71)
GKTWFIIPIISFLLK	CAA35363	UL89
MRVDTHPPTPQVPRC	AAA85874.1	UL135Toledo
YFHTRWIKSLQENHT	AAA85881.1	UL142Toledo

FGWDGETLMELKDNL	CAA35417	UL18
REQQRLDKSLAATAP	AAA85890.1	UL149Toledo
EMWMACIKELHDVSK	CAA35325	UL123(pp72=MIprotein=IE1)
VRRSWEELERKCLAR	CAA35431	UL32(pp150)
AQYTCGFLVRIELGV	CAA35383	UL68
GDQKPGAEHMRDVSY	CAA35406	UL47
RSFFFPGFPPVPVYA	CAA35338	UL102
FLNHQCRVCHFDITP	CAA35333	UL97
EADSLEMLLDKFSTD	CAA35385	UL71
AAYAQMALIQPASQK	CAA35406	UL47
HDTCAAYAQMALIQP	CAA35406	UL47
ERVAERCDDRHGGSD	CAA35334	UL98
DVSWYHSMSFSRRSS	P09704	US28
LGTYHHYLIDNGTLS	CAA35281	US14
GDVQRLIRLFKGEAA	CAA35426	UL27
TALISTPWLTNCSAT	CAA35460	TRL12
RSLTRKKLEQHAAKK	CAA35458	TRL10
CNFITVPEELPHTAS	CAA35422	UL23
TSPRFLEIVRRASEK	CAA35353	UL80
SNIPRSSARLLEHCV	CAA35362	UL88
RRGFATAHQQLAQAL	CAA35386	UL70
RSLMKRTHRASRHAV	CAA35430	UL31
KNHFWKNGDISYTET	CAA35329	UL127
LLGEHGAALVSHTLR	CAA35333	UL97
CSPDEIMAYAQKIFK	CAA35325	UL123(pp72=MIprotein=IE1)
TPFLMEHTMPVTHPP	P19893	UL122(IE2)
SSGSIILAEKSVNMR	CAA35387	UL72
SYCRLDFFRPSAPVS	CAA35377	UL62
ALRIIRLLRASIRHE	CAA35426	UL27
PEQPSRYLRRRMFVE	CAA35394	UL35
RRCSLGRYALWIYNI	CAA35380	UL65
SVAILIVEDDNDAYP	CAA35435	UL2
PVVPEECYDQRFTTE	CAA35311	IRS1
FCGMIWLGIPDSHNI	CAA35280	US13
PWLPARFATLGPLVL	CAA35409	UL50
AQRLSLSFRLITETA	CAA35447	UL14
CDPVHESICARLQPN	CAA35428	UL29
SRMPKNHFWKNGDIS	CAA35329	UL127
AVLARLFEVREVCFL	CAA35338	UL102
PPWQAGILARNLVPM	CAA35357	UL83(pp65)
ELLGKATQQLPYLSA	CAA35407	UL48(pp212)
HVLVRLFHKRGVIQH	CAA35392	UL77
LHRRLETLGCVKTVS	CAA35405	UL46
PMFFNVPRWNTKLYV	CAA35389	UL74(gO)
SYVQGCIFLSFPVIY	CAA35430	UL31
EECYDQRFTTEGHQV	CAA35311	IRS1

STNIEFGAWPVPTAY	AAA85886.1	UL147Toledo
RVEARVIYGDTDSVF	CAA35413	UL54
QLQQVVHHVRVARKG	CAA35386	UL70
PVYRVHLPNDQHVFC	CAA35386	UL70
QRIEENLEGVRRNMF	CAA35431	UL32(pp150)
SGRFFHRRRSRFDFR	CAA35427	UL28
SIFPGTIEGDIGVFP	CAA35387	UL72
MDRLQVSGEQYHHDE	CAA35314	US3
PCVPARRPRYSKDDD	CAA35384	UL69
APCGPQRPAEIPKRR	CAA35416	UL17
RVRWEVYISRARLVN	CAA35328	UL126
EYDDTQGVINIMYMH	CAA35390	UL75(gH)
GTIIPTHASMGEFAR	CAA35386	UL70
TACGQAGCSFCTDHE	CAA35368	UL94
SVMKRRIEEICMKVF	CAA35325	UL123(pp72=MIprotein=IE1)
YGTRSMRKLNKPTCP	CAA35422	UL23
QDHSRLVLDEAFPTF	CAA35407	UL48(pp212)
VSEVDDYVTAVSGYL	CAA35361	UL87
TDGQYATSLRRLDEE	CAA35424	UL25
STHGHHLGHRKNAHT	CAA35460	TRL12
PRRRVYSVRCDHCVE	CAA35344	UL108
VAQKKARHMVEAIRT	CAA35424	UL25
HTEHGLLVSMAEYRS	CAA35339	UL103
VEEMLRYVESKPTNE	CAA35407	UL48(pp212)
LRLTSHGHGLLCARC	CAA35408	UL49
SPPPYRPPYCLVSSP	CAA74074	UL42rev
QFLNQVDLTETLERY	CAA35390	UL75(gH)
RDFHTEVPLYALHGF	CAA35267	US35
VREVCFLRTCLRLVT	CAA35338	UL102
AYLGLSKKLDAFLLN	CAA35407	UL48(pp212)
DRLREVIASVGELVP	CAA35413	UL54
NNRRQHPPPPHTFTN	CAA35268	US36
WREQADRARGTFAWR	CAA35265	US34
TSPHGLGLAGYGGRI	CAA35386	UL70
MVRSLEEIIYIIYSD	CAA35382	UL67
VTVIMIYVLIHFNVP	CAA35434	UL1
CTLADAIKFLNHQCR	CAA35333	UL97
SEFFGRVLAQLHRDR	CAA35361	UL87
SCAGITGTVSIILVS	CAA35419	UL20
SVDTVLYQPPPSWKP	AAA85874.1	UL135Toledo
GSPQLLPYGDRLEVA	CAA35447	UL14
VLVDLGLPQSAWRRW	CAA35439	UL6
HVMLRTEDGIITAAK	CAA35291	US24
AVELVNETFRCSVTS	CAA74075	UL43rev
HHHRPCVPARRPRYS	CAA35384	UL69
QEQGVEDFSLENLRR	CAA35372	UL57

YSQFVDHNLSSEITK	CAA35340	UL105
SYQIPFLAKQLVLFL	CAA35361	UL87
LRGSLLIYVGLVTMF	CAA35280	US13
RSIFFILSVMIGKGT	CAA35398	UL39
TLVPILLHEQKKAFY	CAA35434	UL1
TAVSGYLGEAAAPRI	CAA35361	UL87
FQRPASGCLDAWARR	CAA35316	UL114
VCHSQHERPSLYHDL	CAA35460	TRL12
LSVEEICEEHTLNDL	CAA35424	UL25
GFRGFVQEGLRNYAP	CAA74075	UL43rev
TGMKTVAFDLSSPQK	CAA35431	UL32(pp150)
LIAALYPEYIYTVLK	CAA35407	UL48(pp212)
NDGRGCTSEGVIFGH	CAA35413	UL54
FFGITFYKCLDAQFV	CAA35387	UL72
DLDEEDTSIYLSPPP	CAA35335	UL99(pp28)
ERDWRRVIHDSHGLW	CAA35264	US32
DAFFSLLGASRSAVP	CAA35353	UL80
IRTRRLLVPWIRESK	CAA35419	UL20
ETGGGCFLVNAGEDE	CAA35372	UL57
FLTLALLSDADWLQK	P09724	US20
TETISMLTFGSMSER	CAA35360	UL86(MCP=majorcapsidprotein)
DKLIAWMTWLSSRAT	CAA35284	US17
CVMGKKGHRNHRFSG	CAA35395	UL36
SQSDLIIRPTIWLPG	CAA35387	UL72
AAAAACEDLSELCES	CAA35340	UL105
PDMGRCLCYVPCGPM	CAA35368	UL94
RRRKENYRQGPGFLL	CAA35457	TRL9
KTGLVYEALYPVARS	CAA35366	UL92
AEIPKRRKKAAVLLF	CAA35416	UL17
LLLGVVNALENVTVY	CAA35392	UL77
LALLYNNPDQLRALL	P16832	UL115(gL)
EVCLSVYPSVYLSPY	AAA85873.1	UL134Toledo
NPTTYTTSSGAKISG	CAA35360	UL86(MCP=majorcapsidprotein)
INDWRVMVGSNHVEP	CAA35422	UL23
VAHQKHVSQFVLKEV	CAA35363	UL89
RARSGPSRPQSGPWL	CAA35409	UL50
PYELTLKNSHTLRIY	CAA35358	UL84
VYYELARDLGSHGTE	CAA35361	UL87
RTLTFVSVLLISFVA	CAA35432*	UL33
TRDRDYVLKFLTRLA	CAA35360	UL86(MCP=majorcapsidprotein)
GDAPQDRTRLPQFSS	CAA35426	UL27
DGLFLYRTTVSRGVD	CAA35342	UL106
YTLFVCDVEETILTP	CAA35425	UL26
QSYRSTYMILLLTWL	CAA35432*	UL33
LMSVCAFCWLVLPHR	CAA35288	US21
ALAADIGYGVYVDKA	CAA35338	UL102

SPHPPTSVFLHFSVY	CAA35356	UL82(pp71)
PMPAFALTSLVDPVL	CAA35424	UL25
YQFGQIGYFSGNGVE	CAA35372	UL57
ISLRTEISKDADPIS	CAA35342	UL106
SSTSPVYDLQRYTAE	CAA35384	UL69
WWWAVRANLATPWYV	CAA35291	US24
FPTCFTLSLISRTTT	CAA35443	UL10
DTLLYVASRNGLFAV	CAA35403	UL44(pp50)
TYWYSGNAYNHTIDT	CAA35419	UL20
NNECVGEFANETSGW	CAA35259	US27
EDDFGASLCKVSPPI	CAA35404	UL45
EANNFVIGVLEQAHF	CAA35427	UL28
AARCPRTGLWIVRDR	CAA35367	UL93
LTEWNRELPSLFCDC	CAA35361	UL87
REQRYSLFGRPVSRR	CAA35404	UL45
LRENTTQCTYNSSLR	CAA35390	UL75(gH)
TSHTTVCISPHTTVA	CAA35387	UL72
MRYQLIVLIGQRGGI	CAA35395	UL36
ACGASLMDPLSPSRW	CAA35420	UL21
PGRVRRDSAWDVRPL	CAA35431	UL32(pp150)
DALTELYRDPQFQQI	CAA35363	UL89
RYNALTVRSRDSLLL	CAA35445	UL13
SPYLSSVWVPMSVLA	AAA85873.1	UL134Toledo
TLTEDFFVVTVSIDD	CAA35390	UL75(gH)
LARCPTKPVTSMWNS	CAA35294	UL131
QEPETPRVSGRRLPF	CAA35367	UL93
RELLCLHVFKLRRGC	CAA35426	UL27
LARDLGSHGTEDRPV	CAA35361	UL87
DVASIGDIASYRLSP	CAA35329	UL127
DVARVQDSVSRDLGF	CAA35426	UL27
ALRELVYFFRNHEYF	CAA35407	UL48(pp212)
RCRQQIPWDDTHRQC	CAA35310	J1I
WLIRKDRFIVRPDWC	CAA35293	US26
PPCGTVPSMTCLSEM	CAA35389	UL74(gO)
RVLSVSEVDDYVTAV	CAA35361	UL87
EWQVFGTEAGGGAVR	CAA35338	UL102
SMRKLNKPTCPYGVD	CAA35422	UL23
LDLARDEARTVSYSP	CAA35369	UL95
LGCQIPIQYAAVDLT	CAA35351	UL78
GRRYAVFQPRRFTPR	CAA35420	UL21
VTSVEDVQRVLARAP	CAA35407	UL48(pp212)
GEINITFIHYGDKVP	CAA35417	UL18
EERYAMACLPRDLSL	CAA35361	UL87
LFNTINFHYEAGAIA	CAA35413	UL54
QCIYVVGGEHSPHSL	CAA35428	UL29
LVLLDWFGAVYAIQM	CAA35291	US24

VFIFPSPSFYQKTHF	CAA35373	UL58
QLNVYHQLCRALMNG	CAA35371	UL56
PEAAVASLETAVSTP	CAA35367	UL93
LRCFENSVEGGHLLR	CAA35423	UL24
QWHSDLTTELLWHAH	CAA35411	UL52
DVEEDLTMTRNPQPF	CAA35357	UL83(pp65)
TINGNVTFRGLQNKT	CAA35437	UL4(gp48)
YVKITLTEDFFVVTV	CAA35390	UL75(gH)
RQEVYVEGTTCAQCY	CAA35371	UL56
STRCLLESVYTASAA	CAA35405	UL46
ALIVVGGSAMPRRVL	CAA35361	UL87
TSAFVPSVYMPPTVP	CAA35269	TRS1part
VTNVLSPVFPGGETA	CAA35413	UL54
TSVDTQHRSPSRCFM	CAA35374	UL59
SPPPRVILSVRDKIC	CAA35386	UL70
GIILLILYLIAAYRS	AAA85883.1	UL144Toledo
FACRIQKKKDRRRGV	CAA35421	UL22
FRCSVTSDARKDLQK	CAA74075	UL43rev
EKFTGAFNMMGGCLQ	CAA35325	UL123(pp72=MIprotein=IE1)
VMRGAVSEFLPQSPG	CAA35356	UL82(pp71)
FKTVRDCVFDIATTL	CAA35406	UL47
IPDCVLVFLPPDSGS	CAA35391	UL76
SIQVLAANLDCVITG	CAA35340	UL105
KYHMLQDTVSESEFI	CAA35460	TRL12
SVRDGLFCLGCVTSP	CAA35353	UL80
HVPRSCVLHLFVTDK	CAA35408	UL49
LAVLFQDVRYIATKM	CAA35281	US14
SPPSCSSSSATWLEE	CAA35338	UL102
VTTIIVLICFKFPQK	CAA35442	UL9
DLLTRYASRREDSMS	CAA35341	UL104
MLFFIWAMFTTCRAV	CAA35336	UL100(gM)
LHCLINPILYALLGH	CAA35432*	UL33
LALSTPFLMEHTMPV	P19893	UL122(IE2)
FERYKELIQELCQSS	CAA35412	UL53
LSPVFPGGETARKDK	CAA35413	UL54
CSPPLKESPSRKLKR	CAA35443	UL10
LDESPHSATSPHGLG	CAA35386	UL70
AWPMQCEHLTLRRTI	CAA35432*	UL33
EHCWLQAQIRRLRDY	CAA35407	UL48(pp212)
LKESPSRKLKRKKNG	CAA35443	UL10
NHSSVARTAAAVSAA	CAA35315	UL113
TLFKTIDEYLLRAKD	CAA35360	UL86(MCP=majorcapsidprotein)
GWDGRGLLGPCRDPP	CAA35377	UL62
LYRERRVPCIICVGS	CAA35340	UL105
AECAAHMIISVLSLH	CAA35363	UL89
HLAYNPFRMPTTSTA	CAA35431	UL32(pp150)

MMRRMRRAPAEAAEA	CAA35315	UL113
RFSGHNGIYDRVPDC	CAA35423	UL24
WRDHALRGRWGTAYS	CAA35263	US31
QLWENEYFRTFRLRR	CAA35341	UL104
QNYVKNSVRHMSSFV	CAA35406	UL47
QAAAASQSPPKDMVD	CAA35354	UL80A
AALDFNYLDLSALLR	CAA35390	UL75(gH)
GSAGVSTSLCSVERM	CAA35369	UL95
HGKSDFVAVFSALNK	CAA35372	UL57
AYCLPHALAFLTLLM	CAA35286	US19
RMETGCDSPRHLYIS	CAA35405	UL46
TPESPRLTEVYQTLR	CAA35424	UL25
QQGEDAVVRRCLREY	CAA35385	UL71
TPPHINDTCNMTGPL	CAA35432*	UL33
ADYAACAQARQHLYD	CAA35449	TRL1
GNCEFPTCFTLSLIS	CAA35443	UL10
GYCPLDGHVYPLAAE	CAA35311	IRS1
DVSWDPRIRPDYPQT	CAA35293	US26
ELGLAHSCNEAFLPL	CAA35363	UL89
SFRVGTHKYVLERDD	CAA35338	UL102
HPFWQQCVRRRRTSR	AAA85876.1	UL137Toledo
LVTHCLNTRSRTYVA	CAA35439	UL6
ILCLDKVCRQLHGQD	CAA35404	UL45
HVDKVNTRTWSASIV	CAA35336	UL100(gM)
SALLEYDDTQGVINI	CAA35390	UL75(gH)
RPKKCQTHAPHHVHH	CAA35311	IRS1
ASNVTEKASYVQGCI	CAA35430	UL31
FRLGNAKMLELQMDL	CAA35407	UL48(pp212)
VDLLGALNLCLPLMQ	P19893	UL122(IE2)
RLPRSRFQRFWETPT	CAA35387	UL72
QFWQKVCSNALPKNV	CAA35372	UL57
SRPSVLCCFQENKSP	CAA35356	UL82(pp71)
YRHTWDRHDEGAAQG	CAA35357	UL83(pp65)
LRDAIHELKRDLFAA	CAA35353	UL80
QVNKRLTKKNHLGIK	CAA35455	TRL7
FLCCTGFMPPLSLMV	CAA35284	US17
PATMHPTTGAYFDNG	CAA74074	UL42rev
MTSRRAPDGGLNLDD	CAA35425	UL26
PEFTEEEEEKEKLLT	CAA35419	UL20
GAPPSSGNNSNFWHG	CAA35311	IRS1
KYSIMADSVCLPPCL	CAA35404	UL45
ERMVELSAQSPAADF	CAA35369	UL95
ALYLQPVPLGHKLFL	CAA35284	US17
MNKFSNTRIGFTCAV	CAA35399	UL40
FENPDVHIPCDCITQ	CAA35412	UL53
YQVYFYGLQCPEQLV	CAA35261	US29

LAPGRCFSCVPRDPC	CAA35310	J1I
DKHADEEHRTQMELD	CAA35449	TRL1
YGRQHGPALIAWVEE	CAA35407	UL48(pp212)
CEQAAYAYSLVSGLL	CAA35340	UL105
FVVYAIISIIYFLLI	CAA35336	UL100(gM)
SFGEIVAAAYDDSKF	CAA35341	UL104
KRSDETSSRGRLPGA	AAA85874.1	UL135Toledo
MIVPPTPDIGFTHTP	CAA35387	UL72
KVSRKKREDALLKQM	CAA35366	UL92
VPTHGLLYTVLNTGP	CAA35359	UL85
FRNQQVNKRLTKKNH	CAA35455	TRL7
DYNVTGLPKGFADSF	CAA35443	UL10
VLSDLEAAACLLAAY	CAA35392	UL77
KSNSASVFNIELIAF	CAA35409	UL50
VRQFSQSDLIIRPTI	CAA35387	UL72
PEAQCTREPGFLGRR	CAA35386	UL70
MDEDELQQLSRLIEK	CAA35384	UL69
DAMFEAGNVPSALLP	CAA35425	UL26
RAVRDEIYAVLRRDG	CAA35367	UL93
GPEWMSGVHLDGCAP	CAA35310	J1I
VIGPGHTQTVYFDAA	CAA35387	UL72
FVGEHVKVLEVRAPL	CAA35360	UL86(MCP=majorcapsidprotein)
EAEWRQTQMDVGGLI	CAA35397	UL38
GARAGNQCVCNGIMF	CAA35407	UL48(pp212)
DGDLLRTAMLLYMDQ	CAA35279	US12
HTPQAVATFKFFHQD	CAA35319	UL117
MNQGLCVYYSDEEED	CAA35293	US26
FFSGDALNCPLLNEP	CAA35430	UL31
NRMVRFIINYVGKWH	CAA35259	US27
PSCEEDERELCVPFI	CAA35447	UL14
DFRDLLNFIRQRLCC	CAA35289	US22
VPCFIFKNTGCAVSL	CAA35409	UL50
NPSLFFSGDALNCPL	CAA35430	UL31
AAYPTYLDGERAKGD	CAA35417	UL18
PDVPREAVMSPTMVT	CAA35385	UL71
HVLKAVFSRGDTPVL	CAA35357	UL83(pp65)
SSQTGSFVGMLSHVS	CAA35340	UL105
FFSQCEHYPSFVKLN	CAA35261	US29
RRRRWRGWLLFPALC	CAA35318	UL116
SPFGSGSRRGSQIPA	CAA35295	UL132
MTTTTHSTAAIMSLL	CAA35397	UL38
VLRTARDLGLSASML	CAA35428	UL29
QLTLIWPEKHSLVLR	CAA35428	UL29
QLAGLTALLSAFCYA	CAA35282	US15
DSLEELFRAGLMKVY	CAA35290	US23
FDVVLTFVPSGFVMG	CAA35266	US33

YVTLKNCSRTDVWHD	AAA85895.1	UL153Towne
QTSIDIFKQKATVFL	CAA35363	UL89
IRYYVSVYDELTASE	CAA35295	UL132
KWDVFAYDSGILFFL	CAA35397	UL38
IVLHLGNRCQPWRQV	AAA85882.1	UL143Toledo
KISMCAPDFNMEFSS	CAA35403	UL44(pp50)
ASFLSAFARQELYLM	CAA35390	UL75(gH)
FDVRQFVFDTARLVN	CAA35315	UL113
YFLTSGLAAAAHAIK	CAA35360	UL86(MCP=majorcapsidprotein)
SKYTRMSSLFNDKCA	Q7M6N6	UL48A
DDVWTSGSDSDEELV	CAA35357	UL83(pp65)
LCHPVLHEPAPCLQT	CAA35360	UL86(MCP=majorcapsidprotein)
LPVCHHTLKRDVLRW	CAA35289	US22
MLDRRTVEMAFAYAL	CAA35390	UL75(gH)
ERFVCPVYDSGTPMG	CAA35277	US10
TCPNVLHSVMTLAAM	CAA35360	UL86(MCP=majorcapsidprotein)
VTAVQRALAVFDVLS	CAA35406	UL47
AFLKGYLSEGCLPHT	CAA35385	UL71
YDGPETRPGIYVLTS	CAA35405	UL46
LRTQLDVLYSDPLKT	CAA35394	UL35
MCRRPDCGFSFSPGP	P16832	UL115(gL)
RSSLLTSRSGGALRG	AAA85891.1	UL150Toledo
RSGENETFLWYNLTV	CAA35440	UL7
QQTRHTCLQLVARFF	CAA35339	UL103
VCLILSFSIVAAALW	CAA35323	UL121
ESLVRSVFEHRSLVT	CAA35372	UL57
YNTMTISSVLLALLL	CAA35417	UL18
KLPYSITVTYDHRTS	CAA35343	UL107
GTHLAGFFGTSTQLA	CAA35386	UL70
RGGGQVWSVVPSLVF	CAA35391	UL76
NFYYGFCKSSELLKL	CAA35412	UL53
TEVMKFKETSFSVVR	CAA35395	UL36
KVQHVEAVLRQVYTP	CAA35369	UL95
ITHFQRTIRVLRCYL	CAA35424	UL25
LCVYYSDEEEDQEED	CAA35293	US26
YLAPIALMAYTYNRM	CAA35259	US27
LINTGITVCTGFCGE	CAA35285	US18
KHPAICANVEDYLQD	CAA35269	TRS1part
FSSACVHGQDIVRES	CAA35403	UL44(pp50)
NVNIRGSYPEFLYSL	CAA35361	UL87
WSFGMLFFIWAMFTT	CAA35336	UL100(gM)
VEIGRVLSVSEVDDY	CAA35361	UL87
DDDEAALPGEDEAWI	CAA35430	UL31
GVLGGLRDILYQYAD	CAA35341	UL104
YSGEYDVLITDGDGS	P16845	UL22A
FPPRCSDAGTIRNTS	CAA35415	UL15

LSREVVCSGLNACFY	CAA35259	US27
YCDLIREKEVHRPVV	CAA35404	UL45
LTRKCLHDLLQYLDA	CAA35339	UL103
TWPLRLLLGFYSTVG	CAA35283	US16
MMKMAITGKESICLP	CAA35412	UL53
LAANLDCVITGTTVI	CAA35340	UL105
EVHLPLKPVSLDRLR	CAA35369	UL95
ASQLKTADSPTLFLL	CAA35456	TRL8
FCRASRVLTDPEPIQ	CAA35429	UL30
PPPNCASQVGGPLCY	CAA35349	UL111
GPGFMRYQLIVLIGQ	CAA35395	UL36
WLDGGFSTAVEGDAK	CAA35363	UL89
QLITNNDTRSNNTDT	CAA35419	UL20
FTEHTHKVVSFFIKS	CAA35429	UL30
LGESVAGNSICFGVP	CAA35372	UL57
KFHQGIAQLKRAPAE	CAA35393	UL34
LATVVMEAGGQMIHK	CAA35269	TRS1part
LSPEWVKSFDFREHF	CAA35290	US23
RGMTRCHENGIYYGT	CAA35422	UL23
SHRLHTYAVCEKFIE	CAA35338	UL102
WGRRYEPAPSLHPSY	CAA35354	UL80A
FLDTLALLYNNPDQL	P16832	UL115(gL)
LEDIERLLFEDRRLM	CAA35278	US11
CITKICVSQKILRCL	CAA35378	UL63
QEVLSNEEAETLRYV	CAA35367	UL93
TPSRTHHAAPHRRCF	CAA35310	J1I
VMGDYSVIRVSTIRL	CAA35422	UL23
EDKREMWMACIKELH	CAA35325	UL123(pp72=MIprotein=IE1)
ASLNAVHHKLCGAMI	AAA85891.1	UL150Toledo
CVGARPGGCVPGVSR	CAA35310	J1I
ALWREMDTVSRHSAG	CAA35404	UL45
TMRVRVLLQEHEHCL	CAA35320	UL118
LGCVTSPRFLEIVRR	CAA35353	UL80
AGYGGRIDGTHLAGF	CAA35386	UL70
WMQYLLDHNSLASVP	P09704	US28
LRCRWYLLGAVGSYR	CAA35290	US23
AYDSLMVIFFCPPNQ	P09724	US20
DGTLCLFLEPEEREL	CAA35426	UL27
EVSYPIILNVELMLG	P09704	US28
ACPSVSDSLIPEETG	CAA35385	UL71
SYDNIPPTSSSDEGE	CAA35335	UL99(pp28)
SRTTSNSSRSTSPVA	CAA35424	UL25
NLFSWDRSVAGVAAD	CAA35425	UL26
VTSDACMMTMYGGIS	CAA35325	UL123(pp72=MIprotein=IE1)
DYEPVPRKFRRERSP	CAA35384	UL69
YYRRRDSPGGMDEPP	CAA35354	UL80A

VQSSTLIRVLFYHTP	CAA35363	UL89
FVGAVPRRVPLPQMG	CAA35415	UL15
IFTEHVLGFELVPPS	P16832	UL115(gL)
VTAIGQTEPIAFVHL	CAA35431	UL32(pp150)
RWELICSRVLTSVFF	CAA35280	US13
EAQYLPEISRAAKRI	CAA35426	UL27
LSWSSDESSASSSSR	CAA35276	US9
MIIMMYACLSLVFFL	CAA35383	UL68
MNNNILTYSHLRILR	P21600	Y9K
LCKRICCEFGTTPGE	CAA35335	UL99(pp28)
HAGWAAAVVTVIMIY	CAA35434	UL1
PGWRLDFVEFEDIFG	CAA35293	US26
PKTINNSTPLLGNFM	CAA35403	UL44(pp50)
IISFLLKHMIGISIG	CAA35363	UL89
RYIRVGDNLNTFMCL	CAA35423	UL24
YDVVSTNIEFGAWPV	AAA85886.1	UL147Toledo
NSKVRACVIGYQGTV	CAA35340	UL105
AQVGRYCLQDAVLVR	CAA35413	UL54
VSHTVSCVIDGGNMT	CAA35274	US7
SEGWRFCSRRERRED	CAA35264	US32
AFTTNRKASGTGVAA	CAA35363	UL89
IMFLHALHLGGTSAV	CAA35407	UL48(pp212)
VLLWCCLLLPIVSSV	P16832	UL115(gL)
EGGHLLRNIKTAFGM	CAA35423	UL24
NFGVPSPVYALEALV	CAA35394	UL35
FAVNNETLQLSRYLA	CAA35386	UL70
TACNSPFLASGMMIS	CAA35322	UL120
FSGRSSMVTVLCPDL	AAA85876.1	UL137Toledo
DCKGPFTQVGYLSAF	CAA35321	UL119
ERRILQQQYDWLCLT	CAA35291	US24
LDYALGSWLFGIPVC	CAA35407	UL48(pp212)
TLLMVLRIMTLRTFL	CAA35282	US15
FGLIFFVFISLLWLL	CAA35421	UL22
KEPHIVQYVPATFVL	CAA35339	UL103
NCGIVNNDGAVSEGQ	CAA35261	US29
VALIALYMGSRRVPR	AAA85895.1	UL153Towne
LTVYPCSACNRSVLH	CAA35276	US9
PCGSGQRVTKVCTDY	AAA85883.1	UL144Toledo
QMLALIDDELDAMDE	CAA35384	UL69
RLQLEPVVENVGLYV	AAA85887.1	UL148Toledo
AALRGVPLPPDPQHF	CAA35426	UL27
VSRYSGWSTEYTQWH	CAA35411	UL52
FFWGAAGEGSVTGQA	CAA35376	UL61
TITHNLTITSCYKTA	AAA85896.1	UL154Towne
VVVILRRGWSWCFKI	CAA35282	US15
TSTYNSLTISSFTST	CAA35442	UL9

RPSRSYATRCGAYVA	CAA35282	US15
EHTGVSSVTLLKIFS	CAA35424	UL25
YWLERRRRPGTLMLV	CAA35279	US12
HTTERREIFIVETGL	CAA35390	UL75(gH)
LETLGCVKTVSLGIT	CAA35405	UL46
QPVPLGHKLFLGYYA	CAA35284	US17
AAVQRIHQLSPAHLM	CAA35318	UL116
EQPPRQRRRMVSVTL	CAA35394	UL35
ERPVPCFVVDAVVHP	CAA35282	US15
HFFMWGRESMSFVYL	CAA35382	UL67
VITRSLLLICGYYPP	CAA35277	US10
FAAFRSSYCEGGGGS	CAA35280	US13
ITRIGRDMLERTARF	CAA35413	UL54
LKKYTQTEEKFTGAF	CAA35325	UL123(pp72=MIprotein=IE1)
LDAASQSDPLPGGDG	CAA35296	IRL14
QLQSVSLFLEGLTAY	CAA35330	UL128(viralentry)
RLMAYYALTIKSAQY	CAA35278	US11
LHRVFTQLELRNSYQ	CAA35361	UL87
FTQLELRNSYQIPFL	CAA35361	UL87
TSQRKSLVLHRYSAV	AAA85884.1	UL145Toledo
YSVWCGMSSRLERAV	CAA35293	US26
GGLCSSMAVYDEETM	CAA35430	UL31
CLNTRSRTYVALNML	CAA35439	UL6
VLRHLNQVFLCPTSP	CAA35386	UL70
HHERQRRRRQAMDVP	AAA85875.1	UL136Toledo
SGHRPRPPAPPRPKK	CAA35311	IRS1
ASQAGCLSDVLYNTR	CAA35360	UL86(MCP=majorcapsidprotein)
SAFLGYSAVFLLETE	CAA35339	UL103
ASAAWRGEARRADRR	AAA85873.1	UL134Toledo
TMSTVGFDRVPQYDF	CAA35404	UL45
DPNVMRRHANDDFYK	CAA35388	UL73(gN)
HVPRRNPAKGCLGFL	CAA35372	UL57
RSPTSPSTRNGAAAS	CAA35384	UL69
PENEGEYENLLRELY	CAA35409	UL50
EEPFQRGDPFDKNYV	CAA35403	UL44(pp50)
LLPETSGGTVVVNHS	CAA35315	UL113
CSEEQRASYQKLDAL	CAA35363	UL89
RPAPPALSSPETGDD	AAA85875.1	UL136Toledo
GSSGGGGGSGLLPAK	CAA35372	UL57
PLIMDLPSLSVELSA	AAA85891.1	UL150Toledo
EEPSNSTSWQIPKLM	CAA35295	UL132
GNNYYAYRDSPDLRY	CAA35392	UL77
YFRGRGSGSVKYQAL	CAA35336	UL100(gM)
RCPRKKEERTTRSPV	CAA35415	UL15
NRRSALLRAKSRALG	CAA35396	UL37
GGGGSGLLPAKRSRL	CAA35372	UL57

DDIHTSFLVHKELKL	CAA35405	UL46
NPDLMYATDPHDRDE	CAA35367	UL93
VNSFMKGIRDVGFGK	CAA35446	UL12
CFYKRGEGLSTPYVG	CAA35440	UL7
PALSSPETGDDSNDD	AAA85875.1	UL136Toledo
VNTDDFLKKMLLCAL	CAA35404	UL45
GLDQYLESVKKHKRL	CAA35330	UL128(viralentry)
MSRRSGRGPVGDGRE	CAA35457	TRL9
PRTRLKVLVEVLGAL	CAA35361	UL87
LPLYNEFTSFRLPTS	CAA35319	UL117
RLFFPREDSEPLMSD	CAA35416	UL17
HLSGVPVTPNVDLLA	CAA35406	UL47
QIFYNFHEGKITTET	CAA35394	UL35
RTTRLPPHPGFFSWS	CAA35342	UL106
HLSKWLDGKKDNSWH	AAA85894.1	UL152Towne
RFPTRENRTKTRSFL	CAA35453	TRL4
LIGLLHQTPHMWARS	CAA35393	UL34
KFERLHVRRFRPHEV	CAA35426	UL27
FYREIKHLLSHDMVW	CAA35413	UL54
HRRATAMSVIKDCFL	CAA35422	UL23
WHNVDWISKQPLRGR	CAA35296	IRL14
HEREILDLMRHSPDV	CAA35385	UL71
FDTPDLALAPDLVSS	AAA85887.1	UL148Toledo
GYYRELRFHNPDLSS	CAA35431	UL32(pp150)
LAVWAALRGVPLPPD	CAA35426	UL27
RRLFIEVRLSRRIPD	CAA35391	UL76
MIEGASRQTGLTPKR	CAA35394	UL35
THDTIFFPENIPGVS	CAA35356	UL82(pp71)
RVALHTYGAVGWQLA	CAA35282	US15
TTLEHLSGVPVTPNV	CAA35406	UL47
GIPGEKLRRTVVTTT	AAA85880.1	UL141Toledo
ASRQTGLTPKRFMEL	CAA35394	UL35
QTLVDVARGKFARER	CAA35405	UL46
GDEEDEVTVMSPSPE	CAA35385	UL71
VPVTPNVDLLAELMA	CAA35406	UL47
LNGGLDRHMHRIHPF	AAA85876.1	UL137Toledo
AGRITPPPADFQQPV	CAA35407	UL48(pp212)
PSSSQALSVPSLSSE	CAA35380	UL65
LLPEPRRDHHDGMVA	CAA35291	US24
HLRHYLQNSFLHLLM	CAA35393	UL34
TPVEDVSESLVAKRY	CAA35278	US11
TLMPYVLFRRDTDTE	CAA35406	UL47
DLALAPDLVSSLFVA	AAA85887.1	UL148Toledo
REKNGCPFPALLPKL	CAA35369	UL95
RAPDGGLNLDDFMRR	CAA35425	UL26
DPCCRPPGTSSFPRG	CAA35310	J1I

HLHRSLSGGPDVLYA	AAA85890.1	UL149Toledo
FARLLLGSPFRQRVS	CAA35386	UL70
LLDGYQKKVQQDLQR	CAA35407	UL48(pp212)
KPPSLALLSFEETVH	CAA35340	UL105
HTYGAVGWQLAGLTA	CAA35282	US15
YLRKKEDPFVQNVLR	CAA35334	UL98
RASTRHHELRYKWLI	CAA35293	US26
PPLEDPPRGPEWMSG	CAA35310	J1I
VFVDMWDVAAIRVIN	CAA35404	UL45
QNLQRLNENHRGMLR	CAA35424	UL25
APYQRDNFILRQTEK	CAA35390	UL75(gH)
ILKALQPLDDNAKQE	CAA35407	UL48(pp212)
GHHRRRPACALPHGW	CAA35347	UL109
GAGAWLLPRPEGYTL	CAA35368	UL94
LSDAELSNHANRCRR	CAA35426	UL27
WSKLTYSKPHDAATF	CAA35332	UL130(viralentry)
FGDEDEEQENDGEPR	CAA35394	UL35
RLTLLNLTTENSGKY	CAA35437	UL4(gp48)
KGCLGFLLYRHHERL	CAA35372	UL57
KEDPFVQNVLRRHDA	CAA35334	UL98
YTVPFSGPSVPWRDE	CAA35381	UL66
PPRLCKTGPGPPPLP	CAA35311	IRS1
MVRDPLLRDPRATHL	CAA35424	UL25
LRKGLAQNPVQRREL	CAA35426	UL27
FAPRTGKFTLADLLG	CAA35392	UL77
MLLFAAVMVLVDLGL	CAA35439	UL6
LCHVLIGLLHQTPHM	CAA35393	UL34
HYHRHDGGFPLPTAF	CAA35360	UL86(MCP=majorcapsidprotein)
LQLHHGLMWLRRFAV	CAA35280	US13
NRLLGGLFPPELQKY	CAA35391	UL76
ELRLERNRHLGAFHL	CAA35426	UL27
GTVSLKGENAPFPHL	CAA35423	UL24
VEDYLQDARRRADAQ	CAA35269	TRS1part
LPDGLAPGPFHELRT	CAA35360	UL86(MCP=majorcapsidprotein)
VPKKHVPTQPLDGWS	CAA35431	UL32(pp150)
NGNSPWAPTAPLPGD	CAA35431	UL32(pp150)
FMPLTYPPGTELRLC	CAA35423	UL24
GVCVMRRLINHIVNH	CAA35443	UL10
PPSPGPMHMVVCMPA	CAA35419	UL20
RMGTEALLFKHAGAA	CAA35333	UL97
LYIRERLPKLRYDKQ	CAA35353	UL80
LRDAWTHKRPKPRER	CAA35427	UL28
VFRVPEQPSRYLRRR	CAA35394	UL35
KDIEVQVPIRTRRLL	CAA35419	UL20
VTTTTSEKATLVEHA	CAA35351	UL78
LHLHPRAALWAREPH	CAA35311	IRS1

FCTERDYRKFHQGIA	CAA35393	UL34
SHLICFYKRGEGLST	CAA35440	UL7
TRAARLSQRLCHPRL	CAA35352	UL79
ERLHRHDRRGLFRFR	CAA35400	UL41
RWAPSSWWRARSGPS	CAA35409	UL50
ILRQTEKHELLVLVK	CAA35390	UL75(gH)
YVQRHQGTTVALRNP	CAA35289	US22
RLDVNPDLMYATDPH	CAA35367	UL93
LCVPPYFIHPTVTPF	CAA35268	US36
SSSSNNHHHHHHHHN	CAA35315	UL113
YSPIVIQDCAAAVTD	CAA35369	UL95
RMLSGDVQRLIRLFK	CAA35426	UL27
SYEFLQMNLSKISWL	CAA35397	UL38
MEARAIIRRTAHHWA	CAA35356	UL82(pp71)
PSLKIFIAGNSAYEY	CAA35414	UL55(gB)
TDYLLRRLRLYPPER	CAA35408	UL49
GLFWVGRDPPNPPEC	AAA85894.1	UL152Towne
QPPVTHPVVKGGVRN	CAA35417	UL18
LDEAETQPLYRHLFR	CAA35386	UL70
PTASPAKSDKLEADA	AAA85891.1	UL150Toledo
SHGAAAGPPEADENN	CAA35393	UL34
LSLTLFDEPPPLVET	CAA35278	US11
ETPRVSGRRLPFDDL	CAA35367	UL93
LPSDRHRIHPEVVQR	CAA35340	UL105
VFFVLCLSASSSCAV	CAA35448	UL16
LALHFLTSRKGVTDG	CAA35424	UL25
GGLFPPELQKYRRRL	CAA35391	UL76
VSHVIRSSLILYATE	CAA35281	US14
HHLAEDTVGAASHHH	CAA35384	UL69
RVQRLLWHSLRHGDA	CAA35426	UL27
CKTGPGPPPLPPKQR	CAA35311	IRS1
ATAGRWPVDRFLRVP	CAA35296	IRL14
PLPPRDYPQRDERDR	CAA35424	UL25
YLLRAKDCIRGDTDT	CAA35360	UL86(MCP=majorcapsidprotein)
EKLRRTVVTTTPARR	AAA85880.1	UL141Toledo
GCPFPALLPKLFYEP	CAA35369	UL95
EGQDHGDAVHHSLDV	CAA35261	US29
KKEERTTRSPVRLRV	CAA35415	UL15
SRYLRRRMFVERPET	CAA35394	UL35
RPHPQQQQHHHPGPP	CAA35352	UL79
RRMTVPVPRRYPKGA	CAA35291	US24
NVNVGWFKAATAIVP	CAA35361	UL87
ACCCVKQEIPYQDID	CAA35259	US27
GFASSEAPRKKTIRT	CAA35349	UL111
VTHQGAEEAIVYSNY	CAA35341	UL104
TWFPEHVRGTDPRHV	CAA35373	UL58

QRLRIRLPHRYQRLR	CAA35448	UL16
HRRAGTMQLAQRLCE	CAA35385	UL71
QQQQHHHPGPPHPPL	CAA35352	UL79
DRRGLDEVRMGTEAL	CAA35333	UL97
RLLPRQFPPPPRFPP	CAA35415	UL15
ALRLPEQTVCHLSTF	CAA35361	UL87
WQIPKLMKVAMQPVS	CAA35295	UL132
LVLSRLPRSRFQRFW	CAA35387	UL72
RLHLIKHYQLGLHQF	CAA35359	UL85
LHQEGFRNLPFTVLR	CAA35334	UL98
GPCLIRHSPRCDLLL	CAA35404	UL45
VMRSSAGSLRNYLRH	CAA35406	UL47
GRKGPGPPSSDASTA	CAA35414	UL55(gB)
AIPHRQPRTQSKNQK	CAA35415	UL15
VDRPRRTRRGDASPR	CAA35457	TRL9
PCTHKALHHPIGGLF	AAA85894.1	UL152Towne
VSNDNHGAGGTAAVS	CAA35413	UL54
PKGWHVMLRTEDGII	CAA35291	US24
LARQAVQTFCDTCPR	CAA35408	UL49
ATDAHTPLLQACREL	CAA35360	UL86(MCP=majorcapsidprotein)
DSVVMRCQTPDYEDM	CAA35384	UL69
VCWIKISMRKDKGMS	CAA35336	UL100(gM)
TWHRSLFPRDLLRHV	CAA35386	UL70
TKLPKYDPDEFWTKA	CAA35433	TRL14
KPPHPATSTASHHPH	CAA35449	TRL1
YGRDPEWVTQRFPDL	CAA35353	UL80
QLLTQLHEANVYLCP	CAA35394	UL35
SSFYSQIARSLGVLP	CAA35399	UL40
SHGGSPPQVPHKQPI	CAA35384	UL69
LALLLQVAERLGAVR	CAA35341	UL104
MKGIRDVGFGKPPRI	CAA35446	UL12
VRVLVMEACVFEHEF	CAA35344	UL108
DVLYNTRHRERLGYN	CAA35360	UL86(MCP=majorcapsidprotein)
IFFPENIPGVSIEAG	CAA35356	UL82(pp71)
PFFPFRALLVTGTAG	CAA35340	UL105
DLKYILTRLEYLYKV	CAA35413	UL54
IRRLRDYLRFPTRLE	CAA35407	UL48(pp212)
SLLDEAEWRQTQMDV	CAA35397	UL38
DPAVTLSQLFPGLAL	CAA35392	UL77
VDPHYPGWGRRYEPA	CAA35353	UL80
SYGSDVEDPRDDLAE	CAA35394	UL35
PLFPELAEESLKTFE	P19893	UL122(IE2)
IEALRDADRDNYGRC	CAA35430	UL31
VYHRNQWCHQRQPRS	CAA35408	UL49
LNLMTPSPSHGGSPP	CAA35384	UL69
AIIRRTAHHWAVRLT	CAA35356	UL82(pp71)

HIIMGTAGFRGGHRA	CAA35384	UL69
VCALFNQLVFTAQLR	CAA35393	UL34
GKKTIITEYRITGTR	CAA35294	UL131
SHDGIPRQLAERLRL	CAA35430	UL31
VACPPREPPHRALFR	CAA35445	UL13
HRHGGSGGHNKRRKE	CAA35354	UL80A
ARLRPYGASPSIRYT	CAA35375	UL60
CYDDLRDCVYELAPT	CAA35395	UL36
PPPLPSPPRYPFLVG	CAA35453	TRL4
LRRLDEELRHRGTPE	CAA35424	UL25
DSRDRHHHDPFIDTP	CAA35330	UL128(viralentry)
SNYTVERVTLPYLCH	CAA35341	UL104
RELSRFRKHVHDLKR	CAA35426	UL27
LCCEQNERHRRAGTM	CAA35385	UL71
RRRTDSSLEAGQIFV	CAA35356	UL82(pp71)
LSVPTLIMVRVKNRP	CAA35372	UL57
QDKVVSYARDELTKR	CAA35393	UL34
RRPASLLRHLFQPCH	AAA85891.1	UL150Toledo
LVKKPGQMSAWLRDD	CAA35384	UL69
YRACPIHVRTEPERV	CAA35361	UL87
AAGTTSPPAASGTET	CAA35311	IRS1
PRIQVHYKLLFGLNV	CAA35361	UL87
LRRHERLHRHDRRGL	CAA35400	UL41
TVVHGQPGSHRHLGW	CAA35316	UL114
FRRGFWTLTPGKPDK	CAA35356	UL82(pp71)
LSLQEFCVLIAALYP	CAA35407	UL48(pp212)
TGLCSLAELSHFTQL	CAA35390	UL75(gH)
DQIMGDKPTLVTLLT	CAA35430	UL31
ALRRDDEDWKPSRLP	CAA35269	TRS1part
GGGEGGGLRWFPFRQ	CAA35375	UL60
KDFLRNGFRHRDHFH	CAA35395	UL36
GLQYRLPYATWNFSQ	AAA85880.1	UL141Toledo
KRSWDTTVYHRRRKH	CAA35416	UL17
VVQREKQQLKAWEER	CAA35431	UL32(pp150)
SSPSPRHTFDMDMME	CAA74074	UL42rev
AFLDENFKQLEITPA	CAA35385	UL71
TAPLPGDMNPANWPR	CAA35431	UL32(pp150)
EGRWLPVKTDVWDLV	CAA35368	UL94
EQTIKRHPQLREMLR	CAA35407	UL48(pp212)
YISFHDACILVPAKV	CAA35444	UL11
PVQRRELSRFRKHVH	CAA35426	UL27
GTSTQLARQLERINT	CAA35386	UL70
RACRPFDHMPAADFR	CAA35289	US22
TEKHELLVLVKKAQL	CAA35390	UL75(gH)
RHPHRPHPQQQQHHH	CAA35352	UL79
VPVPRRYPKGARTMH	CAA35291	US24

DAHTCEYLIDRRRHL	CAA35316	UL114
PEWVTQRFPDLTAAD	CAA35353	UL80
GGTAAVSYQGATVFE	CAA35413	UL54
RHDASENAVRRRHER	CAA35445	UL13
HHYPSAAERKHRHLP	CAA35357	UL83(pp65)
YPGWGRRYEPAPSLH	CAA35353	UL80
LNIKGLEKTFLCCDK	CAA74075	UL43rev
AMISSSCSTTCTPLI	AAA85891.1	UL150Toledo
TTYEYLDDCRDDEEF	CAA35426	UL27
AQLNRHSYLKDSDFL	CAA35390	UL75(gH)
RRRLLRTARGKRAAL	CAA35391	UL76
IPRCFVANAEGRAQV	CAA35413	UL54
AVSPELPSRDGIRWQ	CAA35445	UL13
PNYKLNTMAELYLRQ	CAA35413	UL54
VPNVERRGGEDAVAA	CAA35290	US23
DEELRHRGTPESPRL	CAA35424	UL25
RKGVTDGQYATSLRR	CAA35424	UL25
MKREGSIFSWRDGNE	CAA74075	UL43rev
IFQMCSESRVLVGSY	P09724	US20
RDRHRRDRRDSGEYC	CAA35424	UL25
LDLRNLLHHHPPHDL	CAA35386	UL70
DNPLTTNLVSKWMTQ	CAA35372	UL57
HRNEGRCLSLGPPKG	CAA35291	US24
DDEDWKPSRLPGEDS	CAA35269	TRS1part
FLALHLGRRPRVRQT	CAA35386	UL70
HQLLGALGHEVPERK	CAA35395	UL36
GGMDEPPSGWERYDG	CAA35354	UL80A
DHYPPPLPPSSRHAL	CAA35384	UL69
NRLCLDLARDEARTV	CAA35369	UL95
AARHFADYVDPHYPG	CAA35353	UL80
ISLPISAPPGWRLDF	CAA35293	US26
PVTLGSGHGYHPGQK	CAA35443	UL10
KKLPAGGRLPVYRLG	CAA35407	UL48(pp212)
NDGGDMGSRFDNLPG	CAA35296	IRL14
SLGDPLFPELAEESL	P19893	UL122(IE2)
FPSWARRWTQRHDSE	CAA35312	US1
FQYHLEGWFPLRVLN	CAA35396	UL37
ITADPTHPHHGSRTH	CAA35407	UL48(pp212)
SQAGAHSLTPLSMDV	CAA35360	UL86(MCP=majorcapsidprotein)
RDYPQRDERDRHRRD	CAA35424	UL25
LLQPPCRPRSSSPGT	CAA35392	UL77
DTGIYHHGRSVRLPY	CAA35386	UL70
FLLHRKTMKLAACLC	CAA35362	UL88
QALSVPSLSSEKKTA	CAA35380	UL65
RAANCPRPQRNLPYA	CAA35310	J1I
HRVRVEDLVLSSVLS	CAA35413	UL54

TEISKDADPISTVTE	CAA35342	UL106
LQISEKMSRGQPLSS	CAA35352	UL79
LLLTRIRFSNQRCFG	CAA35445	UL13
GGRGGGGGGDSGGMM	CAA35372	UL57
SNDKQKDDKRSLFCY	CAA35343	UL107
VSESFERYKELIQEL	CAA35412	UL53
FLQIVPRGVMFDGQT	CAA35413	UL54
YILSKQNQQHLIPQW	CAA35390	UL75(gH)
WLHRDPRGPGCDKNE	AAA85885.1	UL146Toledo
YLDYRDERDTEEDED	CAA35386	UL70
SVDALYIRERLPKLR	CAA35353	UL80
AVFGVHPETRQAHFL	CAA74075	UL43rev
STASTSSTPRSRPRI	CAA35356	UL82(pp71)
QPGENEVRPHAGVID	CAA35338	UL102
VGYYDEEEKRRERQK	CAA35431	UL32(pp150)
QLKLEILRQLETTIS	CAA35389	UL74(gO)
SVALYNETFGKQLSI	CAA35371	UL56
DKPKGRPKDKPPCEP	AAA85872.1	UL133Toledo
ALPQRRPPPLPQRLL	CAA35415	UL15
SLRFSEPRVLIEAAL	CAA35372	UL57
PQKSGTGPQPGSAGM	CAA35431	UL32(pp150)
NQWCHQRQPRSPQLR	CAA35408	UL49
LFRVFPMNVYRHDEV	CAA35360	UL86(MCP=majorcapsidprotein)
LDHQDGWGDHCSTLK	CAA35384	UL69
WLDGKKDNSWHRVLV	AAA85894.1	UL152Towne
DWCSMRNSLDEVSGT	CAA35293	US26
AAADPETGNERRENR	CAA35430	UL31
FQENKSPHDTVDLTD	CAA35356	UL82(pp71)
MVFVSGTALGTGFHR	AAA85892.1	UL151Toledo
RRPPPLPQRLLPRQF	CAA35415	UL15
QTPVNGNSPWAPTAP	CAA35431	UL32(pp150)
AELYHLPVLEAVRKA	CAA35341	UL104
FVAESITEFLNIGLR	CAA35422	UL23
PLHPDAQHTLPLHHS	CAA35449	TRL1
PECDKPQHYLLPPRG	AAA85894.1	UL152Towne
PTGRSICPSQEPMSI	CAA35357	UL83(pp65)
LFRRRLPRAPVNTVM	CAA35418	UL19
FHPGLISRLLSEEVS	CAA35406	UL47
DVVNHLLKLLPLYRQ	CAA35385	UL71
ATCALGLTLQSVRSL	CAA35430	UL31
PPPPPRHPSCSPTMV	CAA35409	UL50
SVDRQFRRTTYDRWD	CAA35261	US29
PALLPKLFYEPVRDY	CAA35369	UL95
LIDDELDAMDEDELQ	CAA35384	UL69
MGEDTVPYNKPRRHP	CAA35411	UL52
PYLGVFVPHNRQGLK	CAA35358	UL84

HESICARLQPNVPLV	CAA35428	UL29
DDVLSLWSRRLLVGK	CAA35392	UL77
LYSQNRLCLDLARDE	CAA35369	UL95
MPSTSYGSDVEDPRD	CAA35394	UL35
QIAWRRHPRCFDLHR	CAA35328	UL126
LCLSASSSCAVDLGS	CAA35448	UL16
VVRDHKTYRRFSCLR	CAA35427	UL28
FLLWLSPVSSSPSRS	CAA35456	TRL8
ETFCDFLELVQRIPD	CAA35426	UL27
HMRLRPPPDYEETLR	CAA35360	UL86(MCP=majorcapsidprotein)
TGEDTFSAHGKSDFV	CAA35372	UL57
QRGDPFDKNYVGNSG	CAA35403	UL44(pp50)
GSGCALVSTLEGSVC	CAA35366	UL92
GTRRSRLTWRLTWLT	CAA35364	UL90
QHQFLRRTYGPQHRL	CAA35370	UL96
QGLPDFISRQHVLYN	CAA35360	UL86(MCP=majorcapsidprotein)
HVLGFELVPPSLFNV	P16832	UL115(gL)
EKQWQQDLQYRREFV	CAA35274	US7
GNGEVFRVPEQPSRY	CAA35394	UL35
IVFRRLFDSGNNDDY	CAA35283	US16
AREFLLSHDAALFRA	CAA35334	UL98
FVSNFPRKLGINSHN	CAA35443	UL10
CCIRRRLHERTIRHS	AAA85891.1	UL150Toledo
TSSVDTSPPYCRCKG	CAA35386	UL70
TTFWRRTWVPRQNPA	CAA35445	UL13
APHVAFIRRRRPPHH	CAA35310	J1I
HHHHHHNAVTDVAAG	CAA35315	UL113
ETGVTRPMMSLAHIN	CAA35385	UL71
LKRDLFAARQSSTLL	CAA35354	UL80A
YEAGAIARLAKIPLR	CAA35413	UL54
KSQRILEALDILILK	CAA35385	UL71
PISGHVLKAVFSRGD	CAA35357	UL83(pp65)
FVFTLECGRCLVGET	CAA35263	US31
CSQHGAFFPARHLHR	AAA85892.1	UL151Toledo
LSKISWLERHCPPLD	CAA35397	UL38
IVVCIMGWWKLLCSK	CAA35440	UL7
QKHKPPDKPPRLCKT	CAA35311	IRS1
TVHALSRPFNGTTET	CAA35407	UL48(pp212)
VEEPVSRMIVCSCPV	CAA35368	UL94
KIRTLKLCVFIFPSP	CAA35373	UL58
SLGRALRRDDEDWKP	CAA35269	TRS1part
RQKRRTGAWRPFHDC	CAA35452	TRL5
QSDWVRGAWIVSETF	AAA85884.1	UL145Toledo
VCEKFIENLRFRSRR	CAA35338	UL102
KATQQLPYLSAERTV	CAA35407	UL48(pp212)
STKDTSLQAPPSYEE	CAA35414	UL55(gB)

DWISKQPLRGRTRRD	CAA35296	IRL14
VFDPYGRQHGPALIA	CAA35407	UL48(pp212)
RTFAGTLSRHPHRPH	CAA35352	UL79
HLGAFHLPAIRHLTA	CAA35426	UL27
LLLNTYGRPIRFLRE	CAA35390	UL75(gH)
RGAWMPAETFTCPKD	CAA35449	TRL1
RLPDNGFQLLIPKSF	CAA35356	UL82(pp71)
DSFVCLRPVDFQRLT	CAA35407	UL48(pp212)
AITGKESICLPFNFH	CAA35412	UL53
VLHEPAPCLQTFTER	CAA35360	UL86(MCP=majorcapsidprotein)
DPFFLKYVKPPSLAL	CAA35340	UL105
EVGYYNDPVAVFDFA	CAA35413	UL54
LLWHSLRHGDAPQDR	CAA35426	UL27
SQSPPRSCLVAPQSS	AAA85890.1	UL149Toledo
CLVHSACATAHGKYD	CAA35426	UL27
PPLPPRPPHLIEFPP	CAA35419	UL20
AMTAPDTRRQLQHVE	CAA35429	UL30
PWWRRLRVKRPKFPS	CAA35312	US1
ELRFHNPDLSSVLEE	CAA35431	UL32(pp150)
RDSPGGMDEPPSGWE	CAA35354	UL80A
FLTSRKGVTDGQYAT	CAA35424	UL25
HPLSQMNHPPLPDPL	P19893	UL122(IE2)
LIMNVRRSWEELERK	CAA35431	UL32(pp150)
PVLQMIRVPRQDTTT	CAA35407	UL48(pp212)
AHAARSAAVGYYDEE	CAA35431	UL32(pp150)
LIVEDDNDAYPSFGT	CAA35435	UL2
GPAHTRFQGPDSMPS	CAA35394	UL35
SGVHLQRYVRATAGR	CAA35427	UL28
HLNKGWLCATIMQHG	CAA35315	UL113
VTCNSTTTNTASITS	CAA35460	TRL12
VKDSEGRWLPVKTDV	CAA35368	UL94
NHGHDKCMQTSHLLC	CAA35450	TRL2
AGNPYEDDDYYYYRE	CAA35275	US8
VNDGPLLFPHRKKKN	CAA35383	UL68
AHFVVIGWMEPVNKA	CAA35427	UL28
RWQLFRLRCHGWTQQ	CAA35445	UL13
TGKFTLADLLGSDAV	CAA35392	UL77
HGGPYLRRSLFAAGP	CAA35282	US15
NYSGTINGNVTFRGL	CAA35437	UL4(gp48)
VGSERSLSYRYHLES	AAA85881.1	UL142Toledo
CRFDTVEMVDETRPA	AAA85875.1	UL136Toledo
GFRNLPFTVLRLSYA	CAA35334	UL98
VLSPNLWRLPWTTVF	CAA35280	US13
CRRPSPGRRDVRRER	CAA35381	UL66
LPHERHRELCHVLIG	CAA35393	UL34
QRGERGAWMPAETFT	CAA35449	TRL1

LEHAELRLERNRHLG	CAA35426	UL27
LIEDVMRSSAGSLRN	CAA35406	UL47
RLFHKRGVIQHLPGY	CAA35392	UL77
ISAPPGWRLDFVEFE	CAA35293	US26
KETTRQGGAAFACTR	CAA35459	TRL11
QLRRAYQEHRRRKHL	CAA35361	UL87
TETLERYQQRLNTYA	CAA35390	UL75(gH)
YVCEEHLHSFTEKGD	CAA35363	UL89
GTILDKILNVEAMHT	CAA35360	UL86(MCP=majorcapsidprotein)
DPMIMFDEDDDDELS	CAA35395	UL36
PPNRECYFPTVVRRP	CAA35404	UL45
SGLLPSCEEDERELC	CAA35447	UL14
SQTYEDPAHGNWLKE	CAA74075	UL43rev
FVEQLRELVRRDRLL	CAA35406	UL47
QTNSHVSTHAGWAAA	CAA35434	UL1
PSLERTGYRWAPSSW	CAA35409	UL50
ALGVTCACQEPREVL	CAA35283	US16
KGQKPNLLDRLRHRK	CAA35414	UL55(gB)
TVTLQTISLSTNTTT	AAA85896.1	UL154Towne
HRKNGYRHLKDSDEE	CAA35414	UL55(gB)
CIVRFIGTRLFYFLQ	CAA35274	US7
HVFASLDDLVPLTVS	CAA35356	UL82(pp71)
DDDTEGDPDHYPPPL	CAA35384	UL69
ATFLSKKQEVNMSDS	CAA35414	UL55(gB)
ETDPALIMRDRLRRL	CAA35407	UL48(pp212)
TPSPSHGGSPPQVPH	CAA35384	UL69
TLASRDCYERFVCPV	CAA35277	US10
PHSAESTVRHDASEN	CAA35445	UL13
ELKPQYAETYASVSE	CAA35407	UL48(pp212)
QRDERDRHRRDRRDS	CAA35424	UL25
HRGELNLMTPSPSHG	CAA35384	UL69
NSVWHLLRMDTVSAT	CAA35334	UL98
RHMDPEQDYRLPAQD	CAA35430	UL31
SLYLRGQPKFSSIWR	CAA35427	UL28
PFFLEYAKHHPKLSR	CAA35259	US27
QAGCSFCTDHEGHVD	CAA35368	UL94
EVWALRDQTAESPVE	CAA35431	UL32(pp150)
NAQAVRRHINRLFFR	AAA85886.1	UL147Toledo
RIFAKMLRAHGTPVA	CAA35334	UL98
AVFQPRRFTPRPQHD	CAA35420	UL21
RDNFILRQTEKHELL	CAA35390	UL75(gH)
RFKVCDVGRRHIIPG	CAA35395	UL36
ANKRYNTMTISSVLL	CAA35417	UL18
QFRRTTYDRWDGRRW	CAA35261	US29
RVLSAPPSLPLPVSE	CAA35397	UL38
WDLDVRNKWRRRKAL	AAA85875.1	UL136Toledo

RTTRSPVRLRVNIRN	CAA35415	UL15
IRHLVRSYADMNISL	CAA35293	US26
IDNFLEHSSPSRDRF	CAA35411	UL52
NEQAYQMLLALARLD	CAA35414	UL55(gB)
ALLIRMETGCDSPRH	CAA35405	UL46
YLLPPRGKPVCLAPD	AAA85894.1	UL152Towne
APDGVGYRLLKEAIK	CAA35400	UL41
LIIRPTIWLPGTAAG	CAA35387	UL72
AFRFTPANTTTNSST	CAA35419	UL20
PPFDFTGQQLRRAYQ	CAA35361	UL87
PGLDFEAAVFDETRA	CAA35352	UL79
TVDYGLTSRTAMTIA	CAA35340	UL105
HDGGFPLPTAFAHEY	CAA35360	UL86(MCP=majorcapsidprotein)
TVPEELPHTASLRAL	CAA35422	UL23
ALGHWALLSICTVAA	CAA35396	UL37
GSISKAYKGTVRAEG	CAA35393	UL34
PGKRISLRTEISKDA	CAA35342	UL106
RRFSCLRQAGRLYFI	CAA35427	UL28
RIPHYPPSWSRTIPN	CAA35436	UL3
AQKSYRAIFLPAWSK	CAA35377	UL62
WPPRLPHLFRTPNLW	AAA85892.1	UL151Toledo
HYNRSTHGHHLGHRK	CAA35460	TRL12
AFDRERRTWQRACFR	CAA35367	UL93
VALIPVVIILIGTLV	CAA35434	UL1
EEPELEDDDEYDELW	CAA35318	UL116
LATETTALVADVHDL	CAA35447	UL14
YAQKIFKILDEERDK	CAA35325	UL123(pp72=MIprotein=IE1)
SEPCFLHNFFNQEDY	CAA35413	UL54
PPSEATAGRWPVDRF	CAA35296	IRL14
AEGTTARYSAKRQNR	CAA35363	UL89
VVTQGQLRVIGTIGL	CAA35425	UL26
LTTVVLNGSQTYEDP	CAA74075	UL43rev
GSSAVATASTRGKTR	AAA85879.1	UL140Toledo
VSGYRVSSSVSECYV	CAA35276	US9
SRFVESAQGKSLHVC	CAA35340	UL105
SLQYAEGLRQLKGAL	CAA35391	UL76
VEDFSLENLRRVLDA	CAA35372	UL57
NALTTKNSPRLRMRT	CAA35375	UL60
LSHFTQLLAHPHHEY	CAA35390	UL75(gH)
QPGSAGMGGAKTPSD	CAA35431	UL32(pp150)
LLLCGQAENLKGVWP	CAA35293	US26
ALWKVDYDRSVAVGP	CAA35323	UL121
DRHRRDRRDSGEYCC	CAA35424	UL25
APQPPRLRMTPDTDH	CAA35406	UL47
KRYVTEFPHYHRHDG	CAA35360	UL86(MCP=majorcapsidprotein)
VREEIPASDDVLFFV	CAA35372	UL57

SSDASTAAPPYTNEQ	CAA35414	UL55(gB)
GVLQFRGHLNRERWG	CAA35377	UL62
RVARRRSSSDIPFSC	CAA35415	UL15
TLNTNAYDYFGKTLY	CAA35363	UL89
SYKDIPRCFVANAEG	CAA35413	UL54
RNPANWFLVMREQAA	CAA35289	US22
RGRHLDLPYPRGYTL	CAA35425	UL26
DEEEEELLALAGEGK	CAA35409	UL50
FLRTFRNQQVNKRLT	CAA35455	TRL7
ERCDDRHGGSDDYVW	CAA35334	UL98
NTDFRVLELYSQKEL	CAA35414	UL55(gB)
RLPGDCYRSQPHPPK	AAA85880.1	UL141Toledo
AGLRQLRQQLTVRWQ	CAA35445	UL13
WNTKLYVGPTKVNVD	CAA35389	UL74(gO)
PSPSFYQKTHFSSCK	CAA35373	UL58
EEEADKQRTKNTHPS	CAA35415	UL15
FSPARSGRRGRRGLY	CAA35430	UL31
LERQQHQFLRRTYGP	CAA35370	UL96
PHQPLEATLELLLGL	CAA35407	UL48(pp212)
VDGDGKVLHLNKGWL	CAA35315	UL113
DEVDRWIRHAAGVER	CAA35360	UL86(MCP=majorcapsidprotein)
THVAALVFSANSVLY	CAA35372	UL57
GVAGLHTALMRLGFT	CAA35404	UL45
WKSWLPKFGKNLPPP	CAA35375	UL60
DLHLTDTRHCTSCTH	CAA35323	UL121
VADLLKWIGPHTRVK	CAA35403	UL44(pp50)
PLGRPYGFYARVTPR	CAA35311	IRS1
NSQLSLGDPLFPELA	P19893	UL122(IE2)
LDGHVYPLAAELSHF	CAA35311	IRS1
GLGGLGGGGGGGGKK	CAA35403	UL44(pp50)
IQQFYQWWKPDTTSC	CAA35442	UL9
TRGPGLRDGGDGGVC	AAA85892.1	UL151Toledo
YKVDSQRFCKLPTAQ	CAA35413	UL54
YRYSETCMEVTVRVG	CAA35443	UL10
DKILNVEAMHTVLRA	CAA35360	UL86(MCP=majorcapsidprotein)
ERSSTWVKKVIWYLS	CAA35332	UL130(viralentry)
NPFRMPTTSTASQNT	CAA35431	UL32(pp150)
TVQTARDPLYAAEQL	CAA35406	UL47
YSGWWWLTFGCARTV	CAA35261	US29
TFDGVRSPPDVVRLY	CAA35374	UL59
ECLVETTEAVFRLRQ	CAA35275	US8
EQECGTRLHVAWPER	CAA35395	UL36
VRVWYTALGTAWRTS	AAA85891.1	UL150Toledo
VTPGKQEITDAMFEA	CAA35425	UL26
VTQNINTVDMGLGYT	CAA35360	UL86(MCP=majorcapsidprotein)
DYATRLQDLRVTFHR	CAA35360	UL86(MCP=majorcapsidprotein)

DAGDEVHLPLKPVSL	CAA35369	UL95
SDLYTPCSSSGRRDH	CAA35390	UL75(gH)
MFREQRGDPINDPLA	CAA35311	IRS1
LRPWKSTAKHPWFQI	CAA35313	US2
SAEVTKRGYASYTID	CAA35395	UL36
EPLSAYVNALHDHRL	CAA35360	UL86(MCP=majorcapsidprotein)
CPRRFCFSPLDSSAT	CAA35427	UL28
GILPLFIIAFFSREP	CAA35351	UL78
NIYERIPYRPSRQKD	CAA35458	TRL10
LCSVERMVELSAQSP	CAA35369	UL95
GDEDYSGEYDVLITD	P16845	UL22A
CMTDYDYLEVSYPII	P09704	US28
GRAKDKPKGRPKDKP	AAA85872.1	UL133Toledo
VGDNLNTFMCLGLNL	CAA35423	UL24
TYTCVLGNETHSLAT	CAA35447	UL14
RSPPLQRLAGEIYRL	CAA35290	US23
WRDALFTSTLLTVMV	CAA35281	US14
KKTPDPMIMFDEDDD	CAA35395	UL36
YRAIFLPAWSKNAPR	CAA35377	UL62
RAVRECMRECQDPVR	CAA35413	UL54
AVYAIQMDDPNHYVR	CAA35291	US24
FNQLVFTAQLRHYCE	CAA35393	UL34
PPDKPPRLCKTGPGP	CAA35311	IRS1
GRLTCQRVRRLLPCD	CAA35273	US6
LLPRIRITPISTSPR	CAA35449	TRL1
INEDLDEGIMVVYKR	CAA35414	UL55(gB)
TRSVYSQHVTSSEAV	CAA35414	UL55(gB)
RRERWRTTNTDRWCS	CAA35381	UL66
GLPIIGVMLVLIVAI	AAA85877.1	UL138Toledo
PSGNVLRFFGATEHG	CAA35413	UL54
KAGTGASEKRFQALR	CAA35407	UL48(pp212)
ADRVGSGEGGDVGEQ	CAA35407	UL48(pp212)
LGQGSFGEVWPLDRY	CAA35333	UL97
AARLVSTYRDRDIDL	CAA35407	UL48(pp212)
YMVLVSHNLDELARY	CAA35291	US24
ARLDARLERELQKKL	CAA35407	UL48(pp212)
AFIRRRRPPHHTQLV	CAA35310	J1I
LTPPPWYPITVKNTH	CAA35398	UL39
RARASAVAGGRGGDN	CAA35391	UL76
SFVNRRITRPRQIPL	CAA35405	UL46
RLNTEEESEAAEETA	CAA35447	UL14
GAESVLATLAAVRTR	CAA35423	UL24
SVDRSRVPETGGRWL	CAA35429	UL30
TDAMFEAGNVPSALL	CAA35425	UL26
ASLLGDLHRFLFGVD	CAA35408	UL49
RIRLPHRYQRLRTED	CAA35448	UL16

PHTRPAAEVECKKSQ	CAA35385	UL71
LFHVAKLVVIGSYPE	CAA35338	UL102
FYYDSGMTPDVLLAR	CAA35404	UL45
VETGGRGAEEVTWLL	CAA35312	US1
STAHEEEADKQRTKN	CAA35415	UL15
KKLVEMMEQHDRGSD	CAA35341	UL104
RRITRPRQIPLCTGV	CAA35405	UL46
PLSPSRWEVALFPSS	CAA35420	UL21
IERLRNYTLESVYTT	AAA85881.1	UL142Toledo
ILFDGHDLLFSTVTP	CAA35390	UL75(gH)
ANGMPPLTPPHVYMN	CAA35395	UL36
TKDEDTLRSVQHFLW	CAA35279	US12
FLILDRLSAISYGRD	CAA35351	UL78
VVALVNFLRHLTQKP	CAA35431	UL32(pp150)
SSSTSVDTVLYQPPP	AAA85874.1	UL135Toledo
YHRRRKHLPRRRAPC	CAA35416	UL17
RSQTRYLWTPDPSRL	CAA35447	UL14
LSFPITRRIQSRRFP	CAA35453	TRL4
KTAWLRHFNISTHGK	AAA85896.1	UL154Towne
HRSTLIGSKEELQHV	CAA35406	UL47
VRAQLDLRNLLHHHP	CAA35386	UL70
FEIPDVSTPGTPTSI	AAA85874.1	UL135Toledo
WALLSICTVAAGSIA	CAA35396	UL37
HLSQNMRDMYLDMCT	CAA35311	IRS1
IHKTETDERGQWIML	CAA35356	UL82(pp71)
ENAASVADTAESTDA	CAA35358	UL84
AVFDVLSRENLERRG	CAA35406	UL47
AYPSFGTLPASHAQY	CAA35435	UL2
AEQQLFQWLKRFKLL	CAA35285	US18
VLVFLPPDSGSRGIV	CAA35391	UL76
TSPNALLPEWMDAVH	AAA85875.1	UL136Toledo
DEFLYCHTRYETFLR	CAA35290	US23
IIIFYFRIPQKLWLL	CAA35444	UL11
LAQNPVQRRELSRFR	CAA35426	UL27
RYDELRDAIHELKRD	CAA35353	UL80
FPTATQRQIVFRRLF	CAA35283	US16
LYAAILCLDKVCRQL	CAA35404	UL45
CEPSRNRTAVSEFMK	CAA35389	UL74(gO)
RCFDLHRRHRDRSSL	CAA35328	UL126
DDNDAYPSFGTLPAS	CAA35435	UL2
DAKPGLNERDGFRQR	AAA85891.1	UL150Toledo
DNTRNELKATTGSNF	CAA35454	TRL6
LSSSLSGSHGISSAD	CAA35394	UL35
DYSVRQSSVSFPPWR	CAA35318	UL116
GQVRDDVLSLWSRRL	CAA35392	UL77
ACEALKKALRRHRFL	CAA35396	UL37

TFYPEKGIVARVGWS	CAA35275	US8
SRARSACTWTSCTSL	P09724	US20
DSPGGMDEPPSGWER	CAA35353	UL80
NRQEAVRAGLLCRTP	CAA35426	UL27
DRNRERNPGSPQLLP	CAA35447	UL14
TDCWPFEVAPAARLA	CAA35290	US23
KQMRSEYGNAPVFGS	CAA35366	UL92
VKSLSRERFAPEDFS	CAA35340	UL105
RIILRGKTLLLSSHW	CAA35436	UL3
HCTSCTHPYVISLVT	CAA35323	UL121
GAPFDDDDYLDYRDE	CAA35386	UL70
HHDGMVATPYVVFMG	CAA35291	US24
SVYTHRAEVVARHNP	CAA35356	UL82(pp71)
HAVLSRKTPQPYWPH	CAA35352	UL79
RLCCGWLALGAVLPA	CAA35311	IRS1
DAWIVLVATVVHEVD	CAA35311	IRS1
HRRRKHLAVQRYAPC	CAA35361	UL87
LSLGVPEDEWQVFGT	CAA35338	UL102
LFKGEAALLRKGLAQ	CAA35426	UL27
LYPPTSTYNSLTISS	CAA35442	UL9
YNQASKQQTAPPCLL	CAA35348	UL110
AGEKNGGGSRAKRRR	CAA35369	UL95
KAECSEVIVFDAKHL	CAA35334	UL98
CHECQNEMCELRIQR	CAA35384	UL69
ETNQTHDTIFFPENI	CAA35356	UL82(pp71)
SSRHSGKCRRQRRAL	CAA35358	UL84
LDAENLDCDPEVMAV	CAA35372	UL57
RHHELRYKWLIRKDR	CAA35293	US26
LDEVRMGTEALLFKH	CAA35333	UL97
TWLAGLIFSVPAAVY	CAA35432*	UL33
LFDEPPPLVETEPLP	CAA35278	US11
YTVSVNDGPLLFPHR	CAA35383	UL68
RRLLVGKLGRDVPVF	CAA35392	UL77
HLLRLDESPHSATSP	CAA35386	UL70
HQCLQAAKKRPKTHK	CAA35273	US6
SQEPMSIYVYALPLK	CAA35357	UL83(pp65)
CITPTQWPAMQLNKL	CAA35407	UL48(pp212)
PATSTASHHPHASPR	CAA35449	TRL1
SAARAALQWLDLGPH	CAA35405	UL46
TLRTFLQTYFSSDKL	CAA35282	US15
EREESWRRVVDYSHN	CAA35263	US31
CRGLPVKYRTHRAAV	CAA35426	UL27
DEEQENDGEPREAQL	CAA35394	UL35
KGQRILVARHLEYTS	CAA35407	UL48(pp212)
CELGNYHQTTPRHDI	CAA35433	TRL14
LQDARRRADAQALGL	CAA35269	TRS1part

LTHLQSCCLNEHSQL	CAA35399	UL40
QSAWRRWRSHVDDEE	CAA35439	UL6
TLMRETALDAAAEVL	CAA35338	UL102
EGSYATAERILGCDV	CAA35291	US24
PLKMLNIPSINVHHY	CAA35357	UL83(pp65)
ATDEFHQALRRLFAP	CAA35408	UL49
LGALALGRESAPAAE	CAA35311	IRS1
QICRSPPPPLPPRDY	CAA35424	UL25
RERLGYNSKFYSPCA	CAA35360	UL86(MCP=majorcapsidprotein)
IVTLKDIEEIKPSAY	CAA35409	UL50
TSNRLPNCSTITTTA	CAA35439	UL6
TSDDDDQEKETENPQ	CAA35293	US26
LVHQSRLVTYSDPFP	CAA35291	US24
GGRHYHPGTFDRHVL	CAA35392	UL77
VFWGLYVKGWLHRHF	CAA35278	US11
FSQFVPGTESLERFL	CAA35341	UL104
NPAAEAQELAVIPPA	CAA35445	UL13
NVFIVQTLRKEMCAK	AAA85873.1	UL134Toledo
VMWRNRRSALLRAKS	CAA35396	UL37
GSFRLFQLIMRHGPC	CAA35404	UL45
ETQPLYRHLFRTPVL	CAA35386	UL70
DYEETLRLFKTTVTS	CAA35360	UL86(MCP=majorcapsidprotein)
HSEFLTSFRREVDRQ	CAA35319	UL117
PDLTAADRDGLRAQW	CAA35353	UL80
YGTMLVKLAETLRGF	CAA35427	UL28
AELCRRSRASARGRY	CAA35428	UL29
RNNAGRYDDDHEVQE	CAA35445	UL13
MYLQANRDDNFFAER	CAA35436	UL3
PSRARRRQWMREAAQ	CAA35333	UL97
TYLDGERAKGDLIFN	CAA35417	UL18
NTRHRERLGYNSKFY	CAA35360	UL86(MCP=majorcapsidprotein)
KKSIMKFLLNVSDSK	CAA35341	UL104
LTNCSATTYTTYNRT	CAA35460	TRL12
LRYRQNPFCEPSRNR	CAA35389	UL74(gO)
CDPGASLRRLWLPSV	CAA35286	US19
DDDDYLDYRDERDTE	CAA35386	UL70
MSGVHLDGCAPGRRL	CAA35310	J1I
FHATFMARAEAALKD	CAA35426	UL27
TTVVRKYWTFANPNR	CAA35315	UL113
IWLRCVPELRVDYTS	CAA35313	US2
TVAAVSAFACPSVSD	CAA35385	UL71
TKKPKGKEDESLMKG	CAA35269	TRS1part
GHDLLFSTVTPCLHQ	CAA35390	UL75(gH)
DWKGSHTTSGLHRPH	CAA35390	UL75(gH)
KDKFLHMVLPRRLHL	CAA35413	UL54
GKYYFKREDANFTFY	CAA35437	UL4(gp48)

FLCYLCYLQCCGRWC	AAA85880.1	UL141Toledo
QHNSKKCNQTEKWHN	CAA35433	TRL14
PPPFTTVEAVDLCAE	CAA35394	UL35
RNGLFAVENFLTEEP	CAA35403	UL44(pp50)
ERVAYHLKLRPATFG	CAA35399	UL40
YSLPVPFHRFYSNPT	CAA35360	UL86(MCP=majorcapsidprotein)
AFVVWIGSGLNIIWW	CAA35262	US30
RKYWTFANPNRILHQ	CAA35315	UL113
MDLCFGVLKQGSGRT	CAA35334	UL98
VLARLQQHVIGHRRG	CAA35386	UL70
VGGGPPLTESYVLAG	CAA35421	UL22
QLLEKESRGQSRNSV	CAA35334	UL98
ALLISTQAFDPSRYL	CAA35415	UL15
HEQQYLRSGLTCLAG	CAA35392	UL77
LRQLAQSVQDTIQHM	CAA35407	UL48(pp212)
VGPDVDVYEFPSEYE	CAA35413	UL54
RRGAQLCLTEATSLL	CAA35406	UL47
TVHAPYDIHFGVQPR	CAA35430	UL31
PFRQRVSAFVAYAVA	CAA35386	UL70
TLFIGYMPIHCPSET	CAA35327	UL125
YAHPASELRPGSGGW	CAA35312	US1
PPLTESYVLAGGGIT	CAA35421	UL22
PSRKLKRKKNGHVRL	CAA35443	UL10
HNTLTALNTPSRTHH	CAA35310	J1I
AGGQKKKTPAPKHPK	CAA35426	UL27
RAKRFDARADLAVYH	CAA35408	UL49
KVCSNALPKNVPIGD	CAA35372	UL57
IHELKRDLFAARQSS	CAA35353	UL80
RSRFDFRALVSPDRL	CAA35427	UL28
HWVLGDSRPDDIKQR	CAA35311	IRS1
WERLDPNTLVLHRYD	CAA35291	US24
LADLLYLNKAECSEV	CAA35334	UL98
GVSDHAVLSRKTPQP	CAA35352	UL79
VGVTERESYVKASVS	CAA35353	UL80
IIGIYSKQTKYDACV	CAA35366	UL92
GKLHLSYNATAQELL	CAA35320	UL118
SSPCLALLRAKRFDA	CAA35408	UL49
PPDSDLTRNTKQAQE	CAA35289	US22
VRLSRLSLDEVKKYG	CAA35413	UL54
EATALGRELRRRWAG	CAA35311	IRS1
PTKVNVDSQTIYFLG	CAA35389	UL74(gO)
LYAVAFAWVLSIVAA	CAA35351	UL78
KQELFRLGNAKMLEL	CAA35407	UL48(pp212)
GKRVEPTGTCVRAGE	CAA35377	UL62
IEVSWRPTVDPERFR	CAA35376	UL61
PLALEINQRLLIMGL	CAA35359	UL85

RLEMLILDEQVSKRS	CAA35416	UL17
YRDISSTIATEKIPF	CAA35272	US5
HVEYLKFVLQDFDVQ	CAA35424	UL25
AFLTLLMCQPSPQAF	CAA35286	US19
DMAVTAPLTDVDLLK	AAA85877.1	UL138Toledo
LVHHVGRDGDGEGEA	AAA85880.1	UL141Toledo
MPAMTNNRACGLGLN	CAA35360	UL86(MCP=majorcapsidprotein)
QGHHNLLCYRRLSVT	CAA35361	UL87
ENNVVEVSSSTGGAH	CAA35338	UL102
ALGFLFVLDVNVSRF	CAA35340	UL105
FPRKLGINSHNTDTT	CAA35443	UL10
DDVLFFVDGCEALAA	CAA35372	UL57
RVKRNVKKAPCPTGT	CAA35403	UL44(pp50)
VTVYSIYQTHIHTTI	CAA35442	UL9
WWKRLRHSTRRWLFR	CAA35263	US31
VRMDYSSQTINWYLQ	CAA35314	US3
LPRRLHLEPAFLPYS	CAA35413	UL54
RALPSYCRLDFFRPS	CAA35377	UL62
FAGTQTPVNGNSPWA	CAA35431	UL32(pp150)
GQLRVIGTIGLANLF	CAA35425	UL26
HYPGWGRRYEPAPSL	CAA35354	UL80A
SYRWIQRKRLEDPLP	CAA35396	UL37
CVARLQAQPSSRHIP	CAA35424	UL25
LPSLREDYAQLSDVI	CAA35424	UL25
RLFLSHVEVQAYFKR	CAA35340	UL105
VSTLAMLRGFAEFRP	CAA35285	US18
GLAQRALRDRFQNFE	CAA35367	UL93
QFVFDTARLVNCVDG	CAA35315	UL113
TVLCPDLRPSLSLLY	AAA85876.1	UL137Toledo
STVVRENAISFNFFQ	CAA35390	UL75(gH)
YTRHHRDNGQEENYY	CAA35433	TRL14
SRAKRRRRRRAPKND	CAA35369	UL95
MAVYEILSVREEIPA	CAA35372	UL57
NPSPRHDVSAYLCSL	CAA35450	TRL2
CELTRNMHTTHSITA	CAA35390	UL75(gH)
LYVYSPTFLFDHRRR	CAA35406	UL47
ADIDTGMSPWATRGI	AAA85880.1	UL141Toledo
VTLHGLAQRALRDRF	CAA35367	UL93
SENAVRRRHERRRYN	CAA35445	UL13
CARDTCFGREKNGCP	CAA35369	UL95
SPLPLVSFTELLLPP	CAA35430	UL31
ELSKWMTGTAECPTV	CAA35292	US25
TDKRFLNRELGDRLY	CAA35408	UL49
VYPLAAELSHFLRAG	CAA35311	IRS1
LRCENGTTKIIERLY	CAA35448	UL16
ERLSGQVRDDVLSLW	CAA35392	UL77

TLIRVLFYHTPDQNH	CAA35363	UL89
PGPRRINITLIGVRG	AAA85885.1	UL146Toledo
YQWWKPDTTSCIQKT	CAA35442	UL9
LRDCVYELAPTMKDF	CAA35395	UL36
ENCNLTRKCLHDLLQ	CAA35339	UL103
MYRGSDALPAGLYRP	CAA35265	US34
PSPVYALEALVDFQV	CAA35394	UL35
GDLQLLKVLVQKRLQ	CAA35386	UL70
SHEELVLCPPEMEER	CAA35362	UL88
GHRLFRLAAFFTRHW	CAA35352	UL79
LYDQTQPLLLAYPNT	CAA35449	TRL1
VRRDDVNAERPVPCF	CAA35282	US15
IQMDDPNHYVRRVAN	CAA35291	US24
GLGRLLSVTLPRHRV	CAA35392	UL77
SLLDLTVFAGTTTTT	CAA35384	UL69
LFTHFVGRPRHCRLE	CAA35416	UL17
NGFQLLIPKSFTLTR	CAA35356	UL82(pp71)
VELRQYDPVAALFFF	CAA35357	UL83(pp65)
SQSPPKDMVDLNRRI	CAA35353	UL80
YKQRVKYVEDKVVDP	CAA35414	UL55(gB)
ASLMETLKRIRLRNS	CAA35418	UL19
KSFTLTRIHPEYIVQ	CAA35356	UL82(pp71)
GWDAFTIWQARVVRG	CAA35290	US23
VFHQLDYEKQVLEAR	CAA35397	UL38
WTLLKATQIVEMTHK	AAA85891.1	UL150Toledo
PPFVTHLPRNMEGVQ	CAA35360	UL86(MCP=majorcapsidprotein)
FPPPDPPSQPAEDAR	CAA35445	UL13
RNYTLESVYTTTVPQ	AAA85881.1	UL142Toledo
MINKRVKRKKLQTFG	CAA35453	TRL4
NHAVCLDAELHTLLD	CAA35386	UL70
CAQDGLYLALGAGFR	CAA35311	IRS1
TRNNTPPHINDTCNM	CAA35432*	UL33
RRAFCTLADAIKFLN	CAA35333	UL97
RELLFHERLKSALDK	CAA35363	UL89
TSSASTSVIATTQKE	CAA35321	UL119
STQAFDPSRYLRQHG	CAA35415	UL15
DFDLRLRDGVSGLAR	CAA35386	UL70
ILESERRIREGKIPM	CAA35397	UL38
VSAFACPSVSDSLIP	CAA35385	UL71
TCVDDLCRGYDLTRL	P16832	UL115(gL)
PDHRAELCRRSRASA	CAA35428	UL29
APLLERPLPVYRVHL	CAA35386	UL70
NAVLALRIIRLLRAS	CAA35426	UL27
KIPQRLREKWDTRGY	CAA35443	UL10
PDYNERCVAVFQETG	CAA35333	UL97
AAGEQQQPPSLVGTG	CAA35333	UL97

SEPYPDYNERCVAVF	CAA35333	UL97
PLGWVFFVLCLSASS	CAA35448	UL16
AVFSRGDTPVLPHET	CAA35357	UL83(pp65)
RYRVLHKRTYARQSY	CAA35432*	UL33
HAAKRVASEGLRFFR	CAA35338	UL102
SPGLLTIYSVLTTLS	CAA35284	US17
RHHHDPFIDTPGRTQ	CAA35330	UL128(viralentry)
LSVFVDQCVALVFYY	CAA35404	UL45
LHVRRFRPHEVGGHA	CAA35426	UL27
RRNVVFKANCQISSQ	CAA35340	UL105
FYEAFVSGCLPGAAA	CAA35334	UL98
RPRYTKLPKYDPDEF	AAA85895.1	UL153Towne
ILIGTLVPILLHEQK	CAA35434	UL1
SSTSSGQSSGDESNC	CAA35263	US31
RCRLQPSLREPPTPA	CAA35393	UL34
FGAKTFLVKSMVNNT	CAA35322	UL120
LESVYTTTVPQNITT	AAA85881.1	UL142Toledo
PHSATSPHGLGLAGY	CAA35386	UL70
ACIKELHDVSKGAAN	CAA35325	UL123(pp72=MIprotein=IE1)
LSTIPHQPLEATLEL	CAA35407	UL48(pp212)
RHMEDLPKLAEETAR	CAA35404	UL45
GGRLEALWTLRGNLS	CAA35278	US11
METVSTQRETASSET	CAA35281	US14
VNFLRHLTQKPDVDL	CAA35431	UL32(pp150)
IVDLVERVLAKCVRA	CAA35338	UL102
TVYLLSHLPSQRYGA	CAA35390	UL75(gH)
IVGNMSRFVFDPKAD	CAA35313	US2
TTLSVMWRNRRSALL	CAA35396	UL37
IQFGIKHEGLVKTLV	CAA35334	UL98
QNRISDDLIIAVIMA	CAA35363	UL89
RRRSLPQQRRRPSSS	P09724	US20
KESICLPFNFHSHRQ	CAA35412	UL53
TFVTLVRGLELARQH	CAA35394	UL35
VVARHNPYPHLRRLP	CAA35356	UL82(pp71)
RDTLTVLYRSPSNCY	CAA35279	US12
ELQEHLFVRGGIVFN	CAA35315	UL113
ITKAGEDALRPWKST	CAA35313	US2
VANTITEFFRMGLLK	CAA35291	US24
SAGSLRNYLRHTRLC	CAA35406	UL47
DHIFMDILTTCVETM	CAA35325	UL123(pp72=MIprotein=IE1)
QLRHYCEHQDKVVSY	CAA35393	UL34
NAAPETHRLVAFLER	CAA35414	UL55(gB)
HVRRFRPHEVGGHAT	CAA35426	UL27
SQRIGEQDAEHLRTD	CAA35459	TRL11
VRNNKRATCWVVIFW	CAA35259	US27
PIPAPRKNLSTPPTK	AAA85874.1	UL135Toledo

RALVFSSMCAGFVIG	CAA35348	UL110
WHLGQRIVNAYRTEA	CAA35290	US23
ISARWFRWDGNDSHL	CAA35440	UL7
VLPRVHGPRSSSEDE	CAA35293	US26
HKIEDSDLYRIADNF	CAA35289	US22
QFISRFNSGYIKASQ	CAA35363	UL89
RRPLRVPVVNYAWLE	CAA35404	UL45
DKAPKPSKQSKKKKK	CAA35335	UL99(pp28)
STFTTVYSTFNTSYA	CAA35460	TRL12
VSTGPECRNETLYLL	CAA35332	UL130(viralentry)
TKTRSFLFILIVNNN	CAA35453	TRL4
EKGKKGGACSLPGSR	CAA35377	UL62
TLGSSLFNVNDIYEL	CAA35392	UL77
TADEERRGPERVGRF	CAA35341	UL104
DAVWRVQGTFYPEKG	CAA35275	US8
PRYRQHLVPSGNVLR	CAA35413	UL54
PGAEHMRDVSYKLFV	CAA35406	UL47
QTVQPSFLSFTCRLQ	AAA85887.1	UL148Toledo
CNTKLLLPVALIPVV	CAA35434	UL1
ASARGRYLRSLLAFR	CAA35428	UL29
TSCISTAFTNVATLC	CAA35363	UL89
DDETKRSTKHCVTAT	CAA35347	UL109
DNVAGSISKAYKGTV	CAA35393	UL34
RLLEHCVGLAGAKKL	CAA35362	UL88
PELPALPVAAEDPMA	CAA35291	US24
PAPPADIDTGMSPWA	AAA85880.1	UL141Toledo
ATGAQPSHAPAQRVL	CAA35397	UL38
PTLLRRRSLPQQRRR	P09724	US20
PLHVVSTNGCGPSSS	CAA35412	UL53
AIHELKRDLFAARQS	CAA35354	UL80A
AFRLRLRRETVRRPF	CAA35358	UL84
RYCLQDAVLVRDLFN	CAA35413	UL54
GPDDPRRAGGPYGFH	CAA35341	UL104
LLISFVALQTPYVSL	CAA35432*	UL33
PWPRPAASCFRCPLR	CAA35347	UL109
LDFFRPSAPVSVHGF	CAA35377	UL62
QEPMGGAARRIPHFY	CAA35360	UL86(MCP=majorcapsidprotein)
LVSHTLRFVEAKMSS	CAA35333	UL97
ETLKRIRLRNSWVAS	CAA35418	UL19
ELRDQLLDDVICCPE	CAA35290	US23
EAGAAATVAPLTPPA	CAA35385	UL71
PVEYPAGEVQYQRTK	CAA35441	UL8
PVKGRGSRVGVPSLK	CAA35431	UL32(pp150)
QRDFPKCRNNSAPLT	CAA35328	UL126
FYLNGTYTVVRLHVQ	CAA35320	UL118
GDVGREFMLARDLLA	CAA74075	UL43rev

VLSRENLERRGAQLC	CAA35406	UL47
ESSVVLQPLITKGGL	CAA35430	UL31
SVRTEVCLSVYPSVY	AAA85873.1	UL134Toledo
PRPVDSYVMLHSNAR	CAA35272	US5
LVSYTIKLSHDPIEY	CAA35363	UL89
VFPEDLARNGNILFS	CAA35352	UL79
SRLERAVKRLQQRIP	CAA35293	US26
AHPKILKKCGEKRLH	CAA35431	UL32(pp150)
KISMRKDKGMSLNQS	CAA35336	UL100(gM)
SEIREKLIQIIYNFY	CAA35406	UL47
ALLHGKRDEGSFTSP	CAA35284	US17
RWRSHVDDEERGLLM	CAA35439	UL6
SSPFLNLGLSFPSPT	CAA35453	TRL4
QLALLAASGWTLSGL	AAA85878.1	UL139Toledo
DETAFFQDDDTTTAP	CAA35340	UL105
KAYKGTVRAEGKKKL	CAA35393	UL34
PGPPSSDASTAAPPY	CAA35414	UL55(gB)
NSSVTCWGSNGTFGA	CAA35322	UL120
TLKKNVGPIYLCPVP	CAA35411	UL52
TGVVYRDISSTIATE	CAA35272	US5
ETTEAVFRLRQWVPT	CAA35275	US8
RILPTASDAMVAFIN	CAA35412	UL53
VVGLPFFLEYAKHHP	CAA35259	US27
WWLSDAGVRETDPPR	AAA85875.1	UL136Toledo
SFAATLLHRYPINPS	CAA35450	TRL2
GFAEFRPHTTNFAHL	CAA35285	US18
LVNRQIAWRRHPRCF	CAA35328	UL126
RVQGTFYPEKGIVAR	CAA35275	US8
SGTSCRRPSPGRRDV	CAA35381	UL66
VIYYSSCTVGFATVA	CAA35432*	UL33
RPARTDLYYYRPTAV	CAA35393	UL34
FTVCFLCYLCYLQCC	AAA85880.1	UL141Toledo
THFGNYVVGEIIPLQ	CAA35360	UL86(MCP=majorcapsidprotein)
GTYSTLDRALLEKMQ	CAA35407	UL48(pp212)
EEVFLLLNRCHGPLS	CAA35265	US34
PAFRPMPLQKLLICD	CAA35333	UL97
LYELDEDEMGEEMLG	CAA35362	UL88
AFLHYFTTLKQYLRN	CAA35417	UL18
AMDAVYRLAEDVVMF	CAA35395	UL36
LFGIPVCLGVHVADL	CAA35407	UL48(pp212)
SLYTREPVMPLGEIE	CAA35428	UL29
MVLTWLHHPVSNSHI	AAA85885.1	UL146Toledo
FYNKHIIWLSRERKV	CAA35387	UL72
HPIGGLFWVGRDPPN	AAA85894.1	UL152Towne
YHHITFCSVPATDGR	CAA35343	UL107
ILKKCGEKRLHRRTV	CAA35431	UL32(pp150)

CTDADDSWKQLGEDF	CAA35273	US6
GATKKDLFDAVTLCA	CAA35319	UL117
FHRRRSRFDFRALVS	CAA35427	UL28
LFQAVGSPRLFALIQ	CAA35406	UL47
ELQKLWLGVEYHHEV	CAA35361	UL87
TLLKIFSQVPPDERE	CAA35424	UL25
LRWIRPWVRGRHRAT	CAA35276	US9
IYSMMIEGASRQTGL	CAA35394	UL35
KSESYTFLVVTASFL	CAA35379	UL64
LSHDMVWPCPWRETL	CAA35413	UL54
DNFHMFLKCGLLKLR	CAA35289	US22
KTSLTIYNVTTEHAG	AAA85895.1	UL153Towne
PAGEVQYQRTKTHYS	CAA35441	UL8
VHVRLLSYRGDPLVF	AAA85887.1	UL148Toledo
SPEPVQQQPPVEPVQ	CAA35385	UL71
YHPLSDDISESEFIV	AAA85896.1	UL154Towne
ARDLLREEMEANKRD	CAA35367	UL93
GVAAFVGAVPRRVPL	CAA35415	UL15
THKMPPDHLPEQVKA	CAA35407	UL48(pp212)
GRCVRHAKIHRSASH	CAA35430	UL31
VSKDLASYRSFSQQL	CAA35390	UL75(gH)
ESLKTFEQVTEDCNE	P19893	UL122(IE2)
IAADRYRVLHKRTYA	CAA35432*	UL33
VSSIFSGLLSSGSQK	CAA35431	UL32(pp150)
NFSLRSPTYVNLTPP	CAA35389	UL74(gO)
LSTLGTCPVRYKESR	CAA35433	TRL14
LQRVVDAVKGEMLST	CAA35407	UL48(pp212)
ARCAYVEAHREAQLT	CAA35428	UL29
VTLQLRCRWYLLGAV	CAA35290	US23
GAYAVLMATSQRKSL	AAA85884.1	UL145Toledo
INGILEGKDESAPGK	CAA35410	UL51
LGLHATTLLTVTLML	CAA35286	US19
AAVSSSSTSHATSST	CAA35414	UL55(gB)
DTLLELLIEDFDIYV	CAA35431	UL32(pp150)
RDVVDHRTETHAYET	AAA85890.1	UL149Toledo
KTASPTCVKTPSFRR	CAA35380	UL65
QLRGLIAALRRYAGK	CAA35408	UL49
STLHSRSTSHSPSQQ	CAA35358	UL84
NHPEQICRSPPPPLP	CAA35424	UL25
PSSRHALGGTGGHII	CAA35384	UL69
LFGKKLISLYVTYIY	CAA35378	UL63
VTASGFHFSHRSVIY	CAA35288	US21
KRCCRICRWRSTSSS	CAA35420	UL21
AVFRLRQWVPTDLDH	CAA35275	US8
DVDEWFEQRRLAEKE	CAA35290	US23
ASISGCDLLREVQRN	CAA35284	US17

GLTSRTAMTIAKSQG	CAA35340	UL105
SSGRRDHSLERLTRL	CAA35390	UL75(gH)
TITRSQLESALDWFL	CAA35289	US22
WDPPPLRRPSRARRR	CAA35333	UL97
PPDSVATVLGELPQL	CAA35392	UL77
RFEGEVLESVLKRCR	CAA35404	UL45
IHKKTKKPKGKEDES	CAA35269	TRS1part
ACMMTMYGGISLLSE	CAA35325	UL123(pp72=MIprotein=IE1)
NGSSLVYRLEDIPSV	CAA35407	UL48(pp212)
LCIITDAYKETTRQG	CAA35459	TRL11
FIGFQMPRLGGRSGN	CAA35376	UL61
RCRKKREGSPKYSVS	CAA35355	UL81
HGGKKKPPSTTSKTL	CAA35407	UL48(pp212)
EVDCASLMETLKRIR	CAA35418	UL19
DDDSRDELYDVPGIY	CAA35362	UL88
RISELDNEKVRNIMK	P19893	UL122(IE2)
HDVLERFAAAAKPLP	CAA35369	UL95
LTLPPLPANTSAWTL	CAA35407	UL48(pp212)
TVGDGTDVGVAAFVG	CAA35415	UL15
RAEIAEALERVAERC	CAA35334	UL98
FCCGSLQFPHHRPGL	CAA35457	TRL9
GNITLYTELHPFFDF	CAA35360	UL86(MCP=majorcapsidprotein)
FRAKRWELICSRVLT	CAA35280	US13
RWPVDRFLRVPLQRA	CAA35296	IRL14
LYNVTLNDSGAYTEH	CAA35434	UL1
KMSHIYVAMSRVTDP	CAA35340	UL105
SLRTLPWLFWVLLSC	AAA85880.1	UL141Toledo
PDDIKQRRLLKATQR	CAA35311	IRS1
VPTAGAITRRPPGAH	CAA35282	US15
KKACALTRRSRHRLR	CAA35396	UL37
TTIIRFKETNTTGIE	CAA35460	TRL12
MELLDRAPLGQESEP	CAA35394	UL35
PGLDNDLMNEPMGLG	CAA35403	UL44(pp50)
QRRIPRPVDSYVMLH	CAA35272	US5
PDEQARVFCLSADWI	CAA35316	UL114
FQTSATIATTILFML	CAA35279	US12
GSSASSSGFVAESKF	CAA35263	US31
TRLAFVGRFVNEGVL	CAA35423	UL24
KTVLRCGLEIDRPMP	CAA35269	TRS1part
MWAENYETTSPAPVL	AAA85880.1	UL141Toledo
RGQWIMLFLHHDSPH	CAA35356	UL82(pp71)
SPEWVKSFDFREHFV	CAA35290	US23
WMTGTAECPTVKSPH	CAA35292	US25
YGLLTLETAFSVLIS	CAA35281	US14
DIVDKCLNMWERKAA	CAA35340	UL105
ATVVVNSPSVRTEVC	AAA85873.1	UL134Toledo

VASVFEHLTVECVND	CAA35365	UL91
DLAPAAAFGLLQHGG	CAA35282	US15
GKLEYRHTWDRHDEG	CAA35357	UL83(pp65)
FYLMDELRYVKITLT	CAA35390	UL75(gH)
IVRPDWCSMRNSLDE	CAA35293	US26
AAAIGIGWYEPEVSM	CAA35322	UL120
GGDGPEGEAIHLRGR	CAA35371	UL56
PNTYIHKTETDERGQ	CAA35356	UL82(pp71)
EFPSEYELLLGFMLF	CAA35413	UL54
KYKYVFEEVSRLMRL	CAA35319	UL117
DPEVMAVYEILSVRE	CAA35372	UL57
MEPEENNVVPNVERR	CAA35290	US23
LQLDRLVFEAAQRGL	CAA35361	UL87
VLFGRLHPHHVQEVK	CAA35411	UL52
ERCVAVFQETGTARR	CAA35333	UL97
SRSGRAAALALHFLT	CAA35424	UL25
ALEDLLPMRLASPET	CAA35358	UL84
FRNHEYFFQHMVGRL	CAA35407	UL48(pp212)
LETSAADFEQLRRLC	CAA35411	UL52
ANLLRVCQLHAGSKI	CAA35427	UL28
EEMALQVEILKPRGV	AAA85887.1	UL148Toledo
AKEESDSEDSVTFEF	CAA35403	UL44(pp50)
GSRRVPRRPRYTKLP	AAA85895.1	UL153Towne
VWRFEGQFNPHIASE	CAA35274	US7
AKMLATLFLLNRTRD	CAA35360	UL86(MCP=majorcapsidprotein)
GDAGSVAAMFQMSPP	CAA35413	UL54
EEETITDSIAAACRP	CAA35349	UL111
ASLAPQLRAVAGFLN	CAA35440	UL7
HANRCRRKAPLELGP	CAA35426	UL27
KSDPLFEDRLLAYGV	CAA35320	UL118
KTPDELDKYSGSPQT	CAA35358	UL84
IQRKRLEDPLPPWLR	CAA35396	UL37
ADWFRFSGRSPVGTR	CAA35364	UL90
MFDEDDDDELSLPRN	CAA35395	UL36
DLSSPQKSGTGPQPG	CAA35431	UL32(pp150)
FGPRRLTPGSENNTV	CAA35356	UL82(pp71)
HSVETLPALQGGLWE	CAA35289	US22
GFLGTCRGQSMHQVL	CAA35341	UL104
TLQHMSKKQESIATI	AAA85882.1	UL143Toledo
VRKRAYLQKVSEVGY	CAA35371	UL56
HLHELSILKSLSMLF	P21600	Y9K
LRQRKDDLSYKDIPR	CAA35413	UL54
TLVLGLLVLSVVAES	CAA35388	UL73(gN)
KHCVTATHPWPRPAA	CAA35347	UL109
WPRERAWALKNPHLA	CAA35431	UL32(pp150)
VFLLRIYILIWVQCL	CAA35288	US21

GTTIDVSAESSSVLC	CAA35351	UL78
GTIRNTSSTAGGRGR	CAA35415	UL15
RSTSPVAPGEPSAAE	CAA35424	UL25
HIPVEHTGVSSVTLL	CAA35424	UL25
PVVRSPCLQPVRDRN	CAA35447	UL14
ISTTQLTSTLQTTEM	AAA85896.1	UL154Towne
LTDRIDSQLVLSRLP	CAA35387	UL72
LLRSTETAVNPSNAT	CAA35441	UL8
DARQKYALEAYMPEA	CAA35372	UL57
WTYWTTLSVMWRNRR	CAA35396	UL37
EKIRDLRTRFAELAK	CAA35407	UL48(pp212)
LPVYRLGDEVPRRLE	CAA35407	UL48(pp212)
PEQTANRRTPSRVRR	CAA35292	US25
RIPYRPSRQKDNSPL	CAA35458	TRL10
PLASINLMFNGSCTV	AAA85882.1	UL143Toledo
INVKSHVSRPPPLHG	CAA35271	US4
RLSTQLLTRVHNHIL	CAA35407	UL48(pp212)
DDTHRQCAGSVGHNT	CAA35310	J1I
SPVRLRVNIRNRFIP	CAA35415	UL15
LDVLYSDPLKTRLLR	CAA35394	UL35
QRIVNAYRTEAEVSE	CAA35290	US23
YTPGLLDHHNVCDVE	CAA35369	UL95
ENVGLYVAYVVNDGE	AAA85887.1	UL148Toledo
LHGRLNGGLDRHMHR	AAA85876.1	UL137Toledo
TLPMGTTGSYTPPQD	AAA85878.1	UL139Toledo
HLPRVLFKAPYQRDN	CAA35390	UL75(gH)
YMWAGTGVMSTTPLT	CAA35411	UL52
QPPPPARKPSASRRL	CAA35431	UL32(pp150)
SDAPLPYFVPPRSGA	CAA35358	UL84
VFVSLMMICKKRYIG	CAA35413	UL54
AFQITKRTYKGKVHL	CAA35411	UL52
QRRRRQAMDVPDPEL	AAA85875.1	UL136Toledo
DIVLVSAITLFFFLL	CAA35448	UL16
WIGSGLNIIWWTGIV	CAA35262	US30
GCLSDVLYNTRHRER	CAA35360	UL86(MCP=majorcapsidprotein)
AIISIIYFLLIEAVF	CAA35336	UL100(gM)
YAEKHGGRIDGVSLL	CAA35285	US18
KIQNTRSLTSIQNLL	CAA35415	UL15
FHTYDQTDAVLFFDS	CAA35413	UL54
TGNYVGCTPDMGRCL	CAA35368	UL94
SSDLGKLLNPCVFIW	CAA35452	TRL5
ELVQERLSGQVRDDV	CAA35392	UL77
QFMCINETKGLQLYN	CAA35434	UL1
ETPSAREFLLSHDAA	CAA35334	UL98
RATFLVLVAFGLLLH	CAA35295	UL132
TLIMVRVKNRPIYRA	CAA35372	UL57

GVPAPMAATVARESL	CAA35372	UL57
GWERYDGGHRGQSQK	CAA35354	UL80A
ITSIQKYLNTTCIER	AAA85881.1	UL142Toledo
RNADRVTYDGELIYG	CAA35369	UL95
RSGFWGSPPLPIIFS	CAA35348	UL110
RRLLPCDLDIHPSHR	CAA35273	US6
VSKDDLYNREGYGFK	CAA35379	UL64
HRLSFPGESTFCLTA	AAA85891.1	UL150Toledo
VERRCHPHREAAMQT	CAA35407	UL48(pp212)
DGNDSHLICFYKRGE	CAA35440	UL7
YQKGYNCTDKHITLS	CAA35439	UL6
HRAEVVARHNPYPHL	CAA35356	UL82(pp71)
ATTCQKRRYGTFRTG	CAA35271	US4
GLRDILYQYADNDDY	CAA35341	UL104
NVHVSPGWIEANSVT	CAA35415	UL15
LLPKVGIPTDFLTHV	CAA35360	UL86(MCP=majorcapsidprotein)
HMYNKLLILALFTPV	CAA35440	UL7
SLLTAVRRHLNQRLC	CAA35311	IRS1
NEERQQKLRLCGSGC	CAA35335	UL99(pp28)
PHRQQHRRLRRHERL	CAA35400	UL41
LVTEAVVWGNARLDA	CAA35404	UL45
DVGLYSTALFFLFLI	CAA35351	UL78
FDIATTLEHLSGVPV	CAA35406	UL47
TTVEAVDLCAENLSD	CAA35394	UL35
TIDDPFDECPDTHFA	CAA35395	UL36
VPRRPRYTKLPKYDP	AAA85895.1	UL153Towne
LCQVGPQKSTPPLGK	CAA35407	UL48(pp212)
NGMPPLTPPHVYMNN	CAA35395	UL36
ASRTAALQCQRDMLL	CAA35262	US30
IFSFVPDGRLSVRAL	CAA35348	UL110
VGSHPLGQMIVPPTP	CAA35387	UL72
QETLATEYFALLHGI	CAA35407	UL48(pp212)
NPSLMNQGLCVYYSD	CAA35293	US26
QAVQSVMKDAESMQM	AAA85874.1	UL135Toledo
KPQHYLLPPRGKPVC	AAA85894.1	UL152Towne
RARAPSYEEQESSSF	CAA35382	UL67
DARSGQVLHNDASCY	CAA35447	UL14
DFLSQTSIDIFKQKA	CAA35363	UL89
MLGSIEGLRACRPFD	CAA35289	US22
TNASHDTVRIQSLGN	CAA35419	UL20
VRLEKTPTCQHPKKT	CAA35395	UL36
EKSTESSYKSLKHLH	P21600	Y9K
DVNVSRFVESAQGKS	CAA35340	UL105
RAYLTPIRNRQEAVR	CAA35426	UL27
PKRFMELLDRAPLGQ	CAA35394	UL35
VQVACCVYLRWIRPW	CAA35276	US9

HSYALVTLIAEHLAD	CAA35360	UL86(MCP=majorcapsidprotein)
TSNITCNGSLYTVYK	AAA85895.1	UL153Towne
EWLVCRQAEREAVTA	CAA35408	UL49
EMALKAFMEANGNHP	CAA35424	UL25
ITIPFELSAACLTYL	CAA35360	UL86(MCP=majorcapsidprotein)
SRDTDVVNHLLKLLP	CAA35385	UL71
GGWPEHAEAQWRQQV	CAA35312	US1
GARDAELYHLPVLEA	CAA35341	UL104
IFSQVPPDEREEDTL	CAA35424	UL25
CTDSVLDYLGRRQDE	CAA35391	UL76
RRSALRSLLRKRRQR	CAA35412	UL53
DPARRPLNGAMYYGS	AAA85875.1	UL136Toledo
HLIEFPPSPPPSPGP	CAA35419	UL20
PPLPGHARRPRRKRC	CAA35428	UL29
HNGIYDRVPDCPKGR	CAA35423	UL24
ITHRKDPLTTKWKTV	CAA35454	TRL6
QVDLTETLERYQQRL	CAA35390	UL75(gH)
SEPEDDDEDPTYDEL	CAA35311	IRS1
KTTHAYTNAAFTSSD	AAA85878.1	UL139Toledo
EEMKCIGLTMQSMYE	CAA35325	UL123(pp72=MIprotein=IE1)
EENEEEEEELFPSCK	CAA35393	UL34
YSYNHHTVFRQMHAA	CAA35430	UL31
DAQALGLDLATVVME	CAA35269	TRS1part
QSKKKKKPSKHHHHQ	CAA35335	UL99(pp28)
RRSSSDIPFSCRDCE	CAA35415	UL15
LLRNIKTAFGMRVLG	CAA35423	UL24
RALVRLHRVRDALGL	CAA35394	UL35
NDERRNNAGRYDDDH	CAA35445	UL13
VHTVLLSWKVLLTMV	CAA35432*	UL33
PLLRDPRATHLRQKL	CAA35424	UL25
DTTVYHRRRKHLPRR	CAA35416	UL17
YVASRNGLFAVENFL	CAA35403	UL44(pp50)
AGAFATSETHFGNYV	CAA35360	UL86(MCP=majorcapsidprotein)
PDKVVEFLSGSYAGL	CAA35353	UL80
AEGIIRATELVLLFL	CAA35450	TRL2
MRRAPAEAAEAPPQS	CAA35315	UL113
SAARALVNTFRQYFF	CAA35362	UL88
NIPNKIQNTRSLTSI	CAA35415	UL15
TRIPHLAGVLSALKS	CAA35371	UL56
HLGRNANVLTVCRHV	CAA35361	UL87
PMEIKLSRLSVPTLI	CAA35372	UL57
TVLIGDTTLSTLGTC	CAA35433	TRL14
RGVTTSPCPTRSLVY	P09724	US20
MDDLPLNVGLPIIGV	AAA85877.1	UL138Toledo
RGLSTSGRSVKKDPA	CAA35337	UL101
LEHSSPSRDRFVQLL	CAA35411	UL52

SDNNTAYLHMFHPHT	CAA35267	US35
FEAHGSLAPAAEAHA	CAA35431	UL32(pp150)
CRTANSTAGYVDMNV	CAA74073	UL41alt
TRLPVVPKQPKKEPC	CAA35372	UL57
TTKPSCSFAQPVTPR	CAA35343	UL107
PRSNVMRGAVSEFLP	CAA35356	UL82(pp71)
TTLLMLTPVVWSARW	CAA35281	US14
PALHRRPASLLRHLF	AAA85891.1	UL150Toledo
MIIHAATPVDLLGAL	P19893	UL122(IE2)
ETRASGRFFHRRRSR	CAA35427	UL28
TRNPQPFMRPHERNG	CAA35357	UL83(pp65)
LCSMTEELYLRLDGT	CAA35426	UL27
CIASPPSRLPSAVPL	CAA35453	TRL4
GLYAPSHVTSAFVPS	CAA35269	TRS1part
PLTAPIWYPNYAGAL	CAA35279	US12
TENSGKYYFKREDAN	CAA35437	UL4(gp48)
ACVTGTQWSTNFFFS	CAA35261	US29
AQLQFTYDTLRGYIN	CAA35414	UL55(gB)
NSMFYYRDRQEKSVI	CAA35408	UL49
DPSYVREHGVPIHAD	CAA35413	UL54
GASIALEDLLPMRLA	CAA35358	UL84
YICRALKDKRTTLIF	CAA35340	UL105
RFRKHVHDLKRIRFT	CAA35426	UL27
DLFSGDEDSDSSDGY	CAA35431	UL32(pp150)
RSAETTQARTVERTG	CAA35310	J1I
VNNRAYQELCELADP	CAA35340	UL105
MDVNYFKIPNNPRGR	CAA35360	UL86(MCP=majorcapsidprotein)
RTGYRWAPSSWWRAR	CAA35409	UL50
DLVMVFHQLDYEKQV	CAA35397	UL38
ASGCLDAWARRGVLL	CAA35316	UL114
FVSVLLISFVALQTP	CAA35432*	UL33
MMSVYALSAIIGIYL	CAA35390	UL75(gH)
WLRKKKACALTRRSR	CAA35396	UL37
NVDSQTIYFLGLTAL	CAA35389	UL74(gO)
LRGKHIRLYVAAEPL	CAA35341	UL104
FYGTRYIRDELPAAQ	CAA35280	US13
LVSLARTPLCRRRVG	CAA35333	UL97
ELRGVKKKKPTAAAL	CAA35385	UL71
SSVSTTTVLGHATFS	CAA35333	UL97
GTYVNSSVTCWGSNG	CAA35322	UL120
VMLALDAENLDCDPE	CAA35372	UL57
LLFFVRRALNKKYHP	AAA85896.1	UL154Towne
GGVGENLYVHADDVE	CAA35313	US2
LVFFQQKADRPSFYR	CAA35391	UL76
APATLQEEETAASHE	CAA35358	UL84
QLDDLIREQVVFTVC	CAA35359	UL85

PTTVPPPIDLSIPHV	CAA35390	UL75(gH)
NENVTLNHLAVVRAM	CAA35411	UL52
HKYKVTAKNPCRSIK	CAA35272	US5
IALDRYYAIVYMRYR	P09704	US28
RLIRLFKGEAALLRK	CAA35426	UL27
LQGLYLRQYDPPALR	CAA35289	US22
TLPLHHSNTQPHVQT	CAA35449	TRL1
WLAFPDVQGQCANGI	CAA35285	US18
SSGNNSNFWHGPERL	CAA35311	IRS1
PNQCIRHAVCLYLDS	P09724	US20
RECTSPLGAAHEDVA	CAA35411	UL52
FYTHYRSVNGDLAVE	CAA35406	UL47
MDARAVAKRPRDPAD	CAA35405	UL46
REIAAWEGPVAAGNN	CAA35392	UL77
VVFTEDHMLVFDPHS	CAA35407	UL48(pp212)
WRRRKALSRIHRFWE	AAA85875.1	UL136Toledo
VMYKHTFKEPACTVS	CAA35361	UL87
LSFLDWPDGSVTEGV	CAA74075	UL43rev
GLLRDPRLMNRQKER	CAA35315	UL113
AWEGPVAAGNNYYAY	CAA35392	UL77
KAISRDKAAFTSSVS	CAA35438	UL5
LREEEDDDDDEDFED	CAA35336	UL100(gM)
IDSVTFVPRNLSNCS	CAA35318	UL116
SAFFVPRDRRGRSFC	CAA35391	UL76
RGALNLSLCRLVLFV	CAA35351	UL78
LTRRIITHAVIINYY	CAA35424	UL25
LTHIDHIFMDILTTC	CAA35325	UL123(pp72=MIprotein=IE1)
GARQLEMVYPAPEHV	CAA35316	UL114
NVKMVDRIQLVLADL	CAA35411	UL52
TRRWLFRRRRARYTP	CAA35263	US31
REPVMPLGEIEGAED	CAA35428	UL29
SSTAGVSRRVRATRK	CAA35371	UL56
GFCLMRLLDRRGLDE	CAA35333	UL97
GETRPPPDKFDLMSL	CAA35263	US31
TNRVTWFPEHVRGTD	CAA35373	UL58
TTCTPLIMDLPSLSV	AAA85891.1	UL150Toledo
DLSSTPTPTPLSEAM	CAA35356	UL82(pp71)
SSRFQRTQRTMRREK	CAA35282	US15
LSRLIEKKKRARLQR	CAA35384	UL69
CDKFLLPVGTVSRCE	CAA74075	UL43rev
TALLLRYAQRNCTHS	CAA35389	UL74(gO)
SSCTVGFATVALIAA	CAA35432*	UL33
RQRRRMVSVTLFSPY	CAA35394	UL35
ADFALQLHKTHLASF	CAA35390	UL75(gH)
FFVFISLLWLLMFAA	CAA35421	UL22
SEWMVVKYMGFFNFS	CAA35371	UL56

SAFGLTHLQSCCLNE	CAA35399	UL40
SSWWRARSGPSRPQS	CAA35409	UL50
TTQCTYNSSLRNSTV	CAA35390	UL75(gH)
FVGTKFRQELHCLLA	P09704	US28
GGCSRRNSNLFVFCT	CAA35393	UL34
MENLRRVYKNTDTKD	CAA35360	UL86(MCP=majorcapsidprotein)
VTAISYHSILADFNS	CAA35360	UL86(MCP=majorcapsidprotein)
VRSNGVLQFRGHLNR	CAA35377	UL62
EIYQLFEYTRLGVWL	CAA35259	US27
KVIIKPPVPPAPIML	P19893	UL122(IE2)
STPFFEIPNGAGTSS	CAA35367	UL93
KTPTCQHPKKTPDPM	CAA35395	UL36
WSEADLLTLCLYENL	CAA35284	US17
IRYFSYGNSVDNLRR	CAA35275	US8
QIPWDDTHRQCAGSV	CAA35310	J1I
WASLLQLHHGLMWLR	CAA35280	US13
AIANGTTHKPSTASS	AAA85895.1	UL153Towne
SQKTNNTTSPWIYAI	AAA85881.1	UL142Toledo
LVLSQFFSILATMLS	CAA35351	UL78
LNVTFNQSRRKWHSV	CAA35396	UL37
VAAWLVRGNFSDTAP	CAA35276	US9
KEAWLQRGRRAKAMH	CAA35289	US22
NFFYGNGGAGGSPLH	CAA35338	UL102
RLSERREHLVFMLWG	CAA35316	UL114
EFKTTYSDADDQSVR	CAA35391	UL76
ILMGAFCIVLRHCCF	CAA35388	UL73(gN)
FGRPNAAPETHRLVA	CAA35414	UL55(gB)
CQRDMLLRQRRRARR	CAA35262	US30
DYLLHIRQQFDWLEE	CAA35384	UL69
PEGLVEFEAQPGALL	CAA35405	UL46
ESRRWWWAVRANLAT	CAA35291	US24
SPDGTPSVLSLTRDS	CAA35404	UL45
IPFVENAVLKERAFI	CAA35272	US5
DNNCSRRHRVDGARR	CAA35447	UL14
LLRERVSVSELEAVY	CAA35338	UL102
LVCYAVFMQLVFLAV	CAA35336	UL100(gM)
VSEIRSAHFRVEENQ	CAA35314	US3
PDPERIDFRDLPTVY	CAA35334	UL98
HAKRSVSFNFPSGTW	CAA35432*	UL33
GVWQPAAQPKRRRHR	CAA35357	UL83(pp65)
DDDDDEKNIFTPIKK	CAA35431	UL32(pp150)
MTNPGLYASENYNGN	CAA35441	UL8
MNPVDQPPPPLPTQQ	CAA35426	UL27
PWLMEQPPPPSRQTK	CAA35311	IRS1
AGNVPRDSIPCITNV	CAA74075	UL43rev
LCNAWRDHALRGRWG	CAA35263	US31

SFGGPLGPASIDFLP	CAA35368	UL94
CLRQAGRLYFIGLVS	CAA35427	UL28
GQRLREFVFYLNGTY	CAA35320	UL118
NFSDITTNILQDGLK	CAA35448	UL16
APPRPKKCQTHAPHH	CAA35311	IRS1
SMNCHLNCTRNHTQI	CAA35396	UL37
GSLEGVEERMLNVMK	CAA35293	US26
HDLRHSDRSCDLAFG	CAA35447	UL14
LDILILKLVVGEFAM	CAA35385	UL71
TRWKVIGIGYGWSVI	CAA35285	US18
FWYFSYRWIQRKRLE	CAA35396	UL37
RWLSQPEVCVLYVTP	CAA35367	UL93
LFATEYDSAHIVANV	CAA35386	UL70
PECRNETLYLLYNRE	CAA35332	UL130(viralentry)
PYLMVEIGRVLSVSE	CAA35361	UL87
RPEARCRQQIPWDDT	CAA35310	J1I
DRFLRVPLQRAPDPR	CAA35296	IRL14
NSVCVSVVLYTIVMS	CAA35285	US18
ILVKGHGAMDLTCQK	CAA35368	UL94
PPPAPPPLPSPPRYP	CAA35453	TRL4
NQTCILFDGHDLLFS	CAA35390	UL75(gH)
SLLTPDEQARVFCLS	CAA35316	UL114
REPTKDLDDSFDYLV	CAA35351	UL78
LFIFFGLIFFVFISL	CAA35421	UL22
ADLLKGQRILVARHL	CAA35407	UL48(pp212)
WRPPKTSRPWKPGQR	CAA35422	UL23
QPVRDRNRERNPGSP	CAA35447	UL14
LCGDTGTVYAALVGQ	CAA35428	UL29
LQACRELFLAVQFVG	CAA35360	UL86(MCP=majorcapsidprotein)
VCYFSTSVRDVAEAV	CAA35351	UL78
PAAEVECKKSQRILE	CAA35385	UL71
HTCLQLVARFFFRLT	CAA35339	UL103
TQRFPDLTAADRDGL	CAA35353	UL80
PIFQIYYLLHAANHD	CAA35412	UL53
LRYVESKPTNELSQR	CAA35407	UL48(pp212)
VLLALLLCALLFAFL	CAA35417	UL18
VAPPGEKKELPAQAA	CAA35261	US29
PWKPGQRVALVWPKD	CAA35422	UL23
IKHYQLGLHQFVDHT	CAA35359	UL85
RSLGVLPNDHHYALK	CAA35399	UL40
HHTVFRQMHAAGTTT	CAA35430	UL31
PGKEVTLQLHGNQTV	AAA85887.1	UL148Toledo
TLLTVMVSVLASTYT	CAA35281	US14
IQPASQKSSLFVSEI	CAA35406	UL47
DEYDELWFPLYFEAE	CAA35318	UL116
WLVLPHRLEQLFSSV	CAA35288	US21

RYRAREQRYSLFGRP	CAA35404	UL45
GCVKTVSLGITSLLT	CAA35405	UL46
PRSPGPSSLAPGRCF	CAA35310	J1I
QYRREFVKRQLAPKP	CAA35274	US7
HVSPAQTYTLEGYTS	CAA35340	UL105
STTTPYFSYTTSAAL	CAA35389	UL74(gO)
SCHFGECDMPVQRLT	CAA35404	UL45
IHDLLHVIETLVRDP	CAA35407	UL48(pp212)
KNNVPRRRCFLRAVI	CAA35393	UL34
RTLLTMRLGILPLFI	CAA35351	UL78
SLFFFSRLHPKLKGT	CAA35336	UL100(gM)
TYSDADDQSVRWHAT	CAA35391	UL76
RVMMALRLPEQTVCH	CAA35361	UL87
ACVGEHHGRDVNFHT	CAA35290	US23
LAVYRVSRSRLKLVL	CAA35438	UL5
MLRGDSAAKIQERYA	CAA35363	UL89
AADFEQLRRLCAPLA	CAA35411	UL52
AQYGFRLLRGIFLIT	CAA35435	UL2
LLRKLVVEKIFAVYN	CAA35384	UL69
NMFCVKPLDLNLDRH	CAA35431	UL32(pp150)
MTLRTFLQTYFSSDK	CAA35282	US15
YDVPGIYMIRVNDGG	CAA35362	UL88
KRAVEKRKQDSTRQK	CAA35452	TRL5
LQTERGGSQLQMVGH	CAA35398	UL39
TNQYLIKGISYPVST	CAA35390	UL75(gH)
HLYDLNITNSGRYSR	CAA35460	TRL12
RVAALARDEGLRDIL	CAA35334	UL98
ERRGPERVGRFRNGG	CAA35341	UL104
IKNRVMLALDAENLD	CAA35372	UL57
ASLFWTLLKATQIVE	AAA85891.1	UL150Toledo
CEKSAYMLEAGAAAT	CAA35385	UL71
SFGEVWPLDRYRVVK	CAA35333	UL97
FVSLLTGANGVTRTA	CAA35419	UL20
CRVCRGRVAGVPAGC	CAA35310	J1I
YGTVYVYDWETDGLY	CAA35289	US22
DGRKPPGPGVCLSPD	AAA85885.1	UL146Toledo
TSPRDYNVTGLPKGF	CAA35443	UL10
FSSLFEYRKHNDVHI	CAA35443	UL10
TLPLWMQYLLDHNSL	P09704	US28
SLGTTTQGWDPPPLR	CAA35333	UL97
KRDLFAARQSSTLLS	CAA35353	UL80
TTMMGVASTDDDSLL	CAA35358	UL84
NPANWPRERAWALKN	CAA35431	UL32(pp150)
DVVVDATDSRLLMMS	CAA35390	UL75(gH)
LAREACEALKKALRR	CAA35396	UL37
YINRALAQIAEAWCV	CAA35414	UL55(gB)

ATLVASIVATRVEDM	CAA35274	US7
LRDMNVKESPGRCYS	CAA35414	UL55(gB)
LDFTVRGDKLWLHTE	CAA35339	UL103
HYEHGLGRLLSVTLP	CAA35392	UL77
QARHVEPTKIVLFAL	CAA35408	UL49
RARMLQHNGPQQSHH	CAA35396	UL37
DSYGLLGNSVDALYI	CAA35353	UL80
LRRMFGLPPPPSVAP	CAA35261	US29
LEKDHLMLSDAVVCE	CAA35372	UL57
SPSTRNGAAASEHHL	CAA35384	UL69
MEPTPMLRDRDHDDA	CAA74074	UL42rev
YGSTRICKSLAPANK	CAA35319	UL117
TFTLPQSTEEKYDKE	CAA35387	UL72
PLLEHLTQHYEPHVS	CAA35386	UL70
DSEERASQQAENDST	CAA35312	US1
FLFILSMDTFQLFTL	CAA35336	UL100(gM)
SCALSVLDQRLWWEI	CAA35323	UL121
SQRRYLLPEPALAPL	CAA35386	UL70
WRPFHDCCCHLGSSV	CAA35452	TRL5
PMIAVLANVVELAYS	CAA35351	UL78
PSSPGSLEGVEERML	CAA35293	US26
PIWYPNYAGALGRTA	CAA35279	US12
LPVKTDVWDLVKVEE	CAA35368	UL94
HLCPHHHERAVDHKR	CAA35291	US24
NREGQTLVERSSTWV	CAA35332	UL130(viralentry)
TYDCVLSDLEAAACL	CAA35392	UL77
IAEHLADGALPPQLL	CAA35360	UL86(MCP=majorcapsidprotein)
YRPEEEVFLLLNRCH	CAA35265	US34
DVTASAVRAFVGALP	CAA35406	UL47
ILVMIAGLAVIVTSV	CAA35288	US21
QNEMCELRIQRALAP	CAA35384	UL69
TLATKHPAICANVED	CAA35269	TRS1part
PMPLQKLLICDPHAR	CAA35333	UL97
GSVFAAPNRIIDLIT	CAA35371	UL56
FCGVQEPARELPSDR	CAA35404	UL45
LTWEETRGRLQYVYL	CAA35405	UL46
GNGGAGGSPLHGVVG	CAA35338	UL102
NVTFRGLQNKTEDFL	CAA35437	UL4(gp48)
DHPTANKRYNTMTIS	CAA35417	UL18
CFYICLFAGVCFLIN	CAA35259	US27
MPELLESRLQVPQLD	CAA35387	UL72
PGCDKNEHLLYPDGR	AAA85885.1	UL146Toledo
HHGQFMPLTYPPGTE	CAA35423	UL24
PPPMCRVPYNECGVE	CAA35289	US22
HVKVLEVRAPLDHAQ	CAA35360	UL86(MCP=majorcapsidprotein)
PKPGRRKTMKTTPLP	CAA35443	UL10

GNPTFSGRSSMVTVL	AAA85876.1	UL137Toledo
VVAKRLPRPDTPRTP	CAA35335	UL99(pp28)
QIEIHIPCNVHVSPG	CAA35415	UL15
SPATSPLSMLSSASP	CAA35431	UL32(pp150)
IRVSRCRKKREGSPK	CAA35355	UL81
LDAVNVRESFGRLLH	CAA35339	UL103
AVFMQLVFLAVTIYY	CAA35336	UL100(gM)
YQEHDLLDVAPDCVL	CAA35372	UL57
HQMQQTLTTKMQDFL	CAA35407	UL48(pp212)
KWGRLHAPECLVETT	CAA35275	US8
INDTCNMTGPLFAIR	CAA35432*	UL33
SAPADIAKVLISLKP	CAA35274	US7
VERVLAKCVRARDFN	CAA35338	UL102
VYRLHVNIDHFFLPA	CAA35361	UL87
HLAVLDRTEFDTDVR	CAA35431	UL32(pp150)
PDDEIARLSALFVML	CAA35359	UL85
PVADYVLLQPSEDVE	CAA35385	UL71
NSTAGYVDMNVCIGM	CAA74073	UL41alt
LQRGPQYSEHPTFTS	CAA35357	UL83(pp65)
VPQHIADYNDGGDMG	CAA35296	IRL14
MTLIYVDADNTRNEL	CAA35454	TRL6
ACVEVWARELLSSST	CAA35338	UL102
QDLVQATRRILKLGP	CAA35409	UL50
SENTTTVMSTLTKYA	CAA35396	UL37
WVLPGGFAVSSRVTL	CAA35367	UL93
RSSETQTGMGGGRLP	CAA35447	UL14
MGLFSLDRSYEEVKA	CAA35359	UL85
LMHTDYLLHIRQQFD	CAA35384	UL69
DTFQSIFPGTIEGDI	CAA35387	UL72
IEKKKRARLQRGAAS	CAA35384	UL69
DYCRKMRLIDPVTGE	CAA35372	UL57
LVQRIPDYRSVSLRI	CAA35426	UL27
HDCCCHLGSSVFSRP	CAA35452	TRL5
PENAVTAISYHSILA	CAA35360	UL86(MCP=majorcapsidprotein)
GERAKGDLIFNQTEQ	CAA35417	UL18
LENVTVYPTYDCVLS	CAA35392	UL77
LVSFTELLLPPPSVA	CAA35430	UL31
REELGYVRFETGGDV	CAA74075	UL43rev
LAANHRRSRITYATT	AAA85891.1	UL150Toledo
EPVVENVGLYVAYVV	AAA85887.1	UL148Toledo
SSLGLGAVRRESDRR	AAA85877.1	UL138Toledo
AIYDHREADAQTFAA	CAA35360	UL86(MCP=majorcapsidprotein)
FLLVTNVIPRESAEV	CAA35369	UL95
VALIALYMGSHRIPK	CAA35433	TRL14
MWSFEYDRDGDVTSV	CAA35333	UL97
SMVANLASELTMTYV	CAA35359	UL85

REGKIPMTFVDRDSL	CAA35397	UL38
SEYRVEYSEARCVLR	CAA35278	US11
SICPSQEPMSIYVYA	CAA35357	UL83(pp65)
FIQLRSRSALGPFVG	CAA35395	UL36
VVPRAVQSTTTVMTP	CAA35439	UL6
CTPALGRYSLGDQIW	AAA85880.1	UL141Toledo
VLMVLTGFGVQLSPA	CAA35407	UL48(pp212)
NPINNHVDADSSQGG	CAA35340	UL105
LICSREKARRRQMSR	CAA35341	UL104
LFVYFRYERPVLAQR	CAA35283	US16
TDEDFKPEDVKAWSH	CAA35423	UL24
SQHDTNRVTWFPEHV	CAA35373	UL58
TSRHRSIFFILSVMI	CAA35398	UL39
FRALLVTGTAGAGKT	CAA35340	UL105
LKVLVEVLGALQSIS	CAA35361	UL87
CLPFNFHSHRQHTCL	CAA35412	UL53
GVRRNMFCVKPLDLN	CAA35431	UL32(pp150)
VDQVLSLGIPHNWFL	CAA35290	US23
PTTRFVEQLRELVRR	CAA35406	UL47
FMHVTLGSDVEEDLT	CAA35357	UL83(pp65)
RFSVSLATLNDIERF	CAA35371	UL56
GNKTAGEKNGGGSRA	CAA35369	UL95
YAVSVTPATKTSIYG	CAA35413	UL54
YVDADNTRNELKATT	CAA35454	TRL6
EGIQLMGGATQEPLD	CAA35269	TRS1part
NMFHTRQLLVFAHSY	CAA35360	UL86(MCP=majorcapsidprotein)
MAAGHESDDDEWEDL	CAA35290	US23
VSPYDRFRLIETPDE	CAA35369	UL95
DLKRQWSGLSLHCAW	CAA35459	TRL11
TYVRKLALEDSSMLL	CAA35359	UL85
SSSGFVAESKFTWWK	CAA35263	US31
SLRHGDAPQDRTRLP	CAA35426	UL27
TAESLRLAPYPADLK	CAA35384	UL69
LISRTTTTRKPGQKT	CAA35443	UL10
HVLRGLLSLTEWNRE	CAA35361	UL87
LVLLLGRYETVWCLD	CAA35293	US26
APLLQSATRGDSDMF	CAA35362	UL88
NVGDFHGASWENGTA	CAA35265	US34
LDESHWVLGDSRPDD	CAA35311	IRS1
HNPYPHLRRLPDNGF	CAA35356	UL82(pp71)
YRELRQAFPGLDFEA	CAA35352	UL79
ELHTHQLCPRSSDYR	P19893	UL122(IE2)
GVLKRALVRLHRVRD	CAA35394	UL35
QGCSCPLDAPVAILM	CAA35453	TRL4
EFALHVKGLKTAGVP	CAA35372	UL57
ADMNISLPISAPPGW	CAA35293	US26

LPHLFRTPNLWLPTT	AAA85892.1	UL151Toledo
IRNRFIPANIPNKIQ	CAA35415	UL15
ALVARGPSLAHYVTA	CAA35413	UL54
PTSVFLHFSVYTHRA	CAA35356	UL82(pp71)
WCVTVGLVPLLDVKT	CAA35341	UL104
PRGVPSCLFNKVWVS	CAA35439	UL6

Peptides recognized at 24 month after HSCT. 4 / 4 patients, light blue, 3 / 4 patients dark blue; 2 / 4 patients light yellow and 1 / 4 patients, green.
