# Supplementary material for: Whole CMV Proteome Pattern Recognition Analysis after HSCT Identifies Unique Epitope Targets Associated with the CMV Status
Source: PLoS One. 2014 Apr 16;9(4):e89648. doi: 10.1371/journal.pone.0089648 (PMC3989190; doi:10.1371/journal.pone.0089648)
Supplement: Table S4 — Epitopes Predicted By PAM - Serum CCMV Epitope Recognition After HSCT. (PDF) [file pone.0089648.s008.pdf]

Supplementary Table S4
EPITOPES PREDICTED BY PAM - SERUM CMV EPITOPE RECOGNITION AFTER HSCT

Time	Epitope	HCMV ORF	Average Q-value
6 months after HSCT			
Serologic group D-R+			
	YLSPERMFFHPGLIS	UL47	0.724
	MEPTPMLRDRDHDDA	UL42 rev	-0.6094
	PSPPRYPFLVGWSWG	TRL 4	0.5304
	SPSMGFQRTFIDPLW	UL74 (gO)	0.5232
	HNLCYSTLLVPGGEY	UL54	0.4803
	NFEFLVRYYIGPWYA	UL77	0.4525
	LAPGRCFSCVPRDPC	J1I	-0.4474
	VRLSRLSLDEVKKYG	UL54	-0.4324
	ILVKGHGAMDLTCQK	UL94	-0.4196
	IVLFFARRAFNKKYH	TRL12	0.3985
	GWNGYLRNVTHYYTN	UL 1	0.3827
	WPNGWFFTYCDLLRV	UL43 rev	0.3698
	NITLKNAIRLRNGTM	TRL13	-0.3038
	RGTMDDDEAALPGED	UL31	-0.2884
	IADNWVMFIHNKRCT	UL105	0.2817
	VVGIFDGQHFFTYHV	UL18	0.2709
	LFFFKIPQRLREKWD	UL10	0.2644
	YLKGNCTQWSVIYSG	UL18	0.2613
	GNEQVSRIACTSCED	UL53	-0.2485
	PHRALFRLCLGLWVS	UL13	0.2304
	FRCSVTSDARKDLQK	UL43 rev	-0.2234
	FDTNNAMGTYRCGAV	UL69	-0.2216
	TGPRHVIWPGTSVLW	UL88	0.2149
	EKYWRMRTTHTVEFY	UL60	0.2001
	TQSRWTIHHMYNKLL	UL 7	0.1958
	PCDLDIHPSHRLLTL	US 6	0.1948
	RGLQNKTEDFLHWLL	UL 4 (gp48)	0.1935
	INRGESYLTTIWLLN	UL20	0.1884
	SSSSNNHHHHHHHHN	UL113	-0.1836
	LHVRRFRPHEVGGHA	UL27	-0.1656
	KKMMWFVVLTVSFSY	UL66	-0.1582
	MLGIRAMLVMLDYYW	UL20	0.1527
	EVQRVMGLVDMDCEK	UL71	-0.1514
	DDDTEGDPDHYPPPL	UL69	-0.1424
	NSTSWQIPKLMKVAM	UL132	0.142
	WFFTYCDLLRVGYFG	UL43 rev	0.1361
	VTPDLDFYWVLPGGF	UL93	0.1312
	NFLTEEPFQRGDPFD	UL44 (pp50)	-0.1274
	YVFCYVREDTAVYYL	UL24	0.1256

LAARALHMPTLANDV	UL79	-0.1235
RKKLEQHAAKKQNIY	TRL10	-0.117
LKRKQAPVKEQFEKK	UL74 (gO)	-0.1147
VGYYDEEEKRRERQK	UL32 (pp150)	-0.1121
WLLGWGHKSICSFFP	UL 4 (gp48)	0.1048
HYRYEVANLTYNCTY	UL 4 (gp48)	0.1037
TCDGITPDVIYEVCM	UL 4 (gp48)	-0.0952
QPGENEVRPHAGVID	UL102	0.0879
EPHGQWEFMFREQRG	IRS1	-0.0861
HLTIQVLESWFTPWV	UL142 Toledo	0.0846
TEGQVLLTMAYYLFE	UL70	0.0798
DIPSCDRCVRRRRFK	UL36	0.0787
KQFYGGLIFHTTWVT	UL 5	0.0713
RCRQQIPWDDTHRQC	J1I	-0.0709
RAMLVMLDYYWIQLI	UL20	0.0706
LGHKLFLGYYAMALS	US17	0.0703
MRRAPAEAAEAPPQS	UL113	-0.0702
FCNRLEWVYFLTSGL	UL86	0.0689
GEVVNTMFENASTWT	UL77	0.0661
PSLKDSCHLCAWTFG	UL21	0.0614
QLVLFMTPKWDVFAY	UL38	0.0598
RACRPFDHMPAADFR	US22	0.0466
PWSRLIITKQFYGGL	UL 5	0.0466
HFTPVKFVYEVWRGQ	UL142 Toledo	0.0402
INDWRVMVGSNHVEP	UL23	-0.0392
LPHEDAFYTGLSVWR	UL31	0.0258
CGFLVRIELGVYFFS	UL68	0.0254
FVSNFPRKLGINSHN	UL10	-0.0239
DSAAKIQERYAELQK	UL89	-0.0236
EVAIAECAAHMIISV	UL89	0.0231
WMTGTAECPTVKSPH	US25	-0.0213
RYDELRDAIHELKRD	UL80	-0.0165
DDDEAALPGEDEAWI	UL31	-0.0108
ERLLFEDRRLMAYYA	US11	0.0102
YLRRFEGSCVSLGWP	UL29	0.007
YRSELLCTSAFLGYS	UL103	0.0036

Serologic group D+R-

GNEQVSRIACTSCED	UL53	-0.6638
SLVSGLLFSAMYYFY	UL105	0.6196
NITLKNAIRLRNGTM	TRL13	-0.5458
VTEDEIIGVAFTWQH	UL16	0.5174
TCDGITPDVIYEVCM	UL94	-0.4764
PVYDPSSSPKKTPEK	UL48	-0.4322
ILVKGHGAMDLTCQK	UL94	-0.3686
YTGDVEAFYLIIHPR	TRL11	0.368
RGTMDDDEAALPGED	UL31	-0.3258
TLDTCYYVYVTQNGT	UL11	0.3174
QLVFLAVTIYYLVCW	UL100	0.3054

MSVKGVEMPEMTWDL	UL136 Toledo	-0.2628
FAYDSGILFFLAPSM	UL38	0.2366
LVLIVAILCYLAYHW	UL138 Toledo	0.2362
LAPGRCFSCVPRDPC	J1I	-0.2294
QTMELMIRTVPRITC	UL36	-0.2188
ILLHVPTHGLLYTVL	UL85	0.2186
RACRPFDHMPAADFR	US22	0.2054
NFYTFFMCLYVYSPT	UL47	0.2038
QLQQVVHHVRVARKG	UL70	-0.195
DLLTRYASRREDSMS	UL104	-0.1904
LKLSVVRLDNFSVEL	UL46	-0.1902
LPGEDKVYVLGLSFG	UL28	0.1868
AQGTDLIRFERNIIC	UL55 (gB)	-0.1868
PKRFMELLDRAPLGQ	UL35	-0.1798
AIPHRQPRTQSKNQK	UL15	-0.1784
RNTVESTTSSEPTTE	UL132	0.1734
ELCDESVRRFVLRHM	UL45	-0.172
EGGHLLRNIKTAFGM	UL24	-0.1676
CSCKCISGPCSRCCS	UL133 Toledo	-0.166
RSSNTLQHMSKKQES	UL143 Toledo	0.1594
REFVFYLNGTYTVVR	UL118	0.1568
EKEQRVRMFYAVFTT	UL93	0.156
CDLQKRPPETFSQPM	UL69	-0.1552
CPFLYPSPPRSPLQF	UL130	0.1488
TIFSPEDDSSCILCQ	UL94	-0.1476
SCDLAFGSRSQTRYL	UL14	0.144
VAILCYLAYHWHDTF	UL138 Toledo	0.144
NISNVTYNGQRLREF	UL118	0.139
TCPNVLHSVMTLAAM	UL86	-0.1322
LREYLADLLYLNKAE	UL98	-0.129
YNVLFYTAHYTSRGA	UL25	0.129
TRLASDSLCLFHSSF	UL 3	0.1286
TTAAAATTNTTVATT	UL116	0.1282
MRRAPAEAAEAPPQS	UL113	-0.1212
ERTPCEQAAYAYSLV	UL105	0.121
LRYYHQDSWRDMLHD	UL136 Toledo	0.102
MKREGSIFSWRDGNE	UL43 rev	-0.0916
EVQRVMGLVDMDCEK	UL71	-0.091
FIPANIPNKIQNTRS	UL15	-0.0894
SHEELVLCPPEMEER	UL88	-0.087
DDDTEGDPDHYPPPL	UL69	-0.08
SPLSMLSSASPSPAK	UL32 (pp150)	-0.0794
RWCPTPGRGRRGGEG	UL141 Toledo	0.0746
FVSNFPRKLGINSHN	UL141 Toledo	-0.0696
ISALSESCNQTCSCQ	TRL13	-0.0656
HNVTREVNVRKRAYL	UL56	0.0624
MLGIRAMLVMLDYYW	UL20	0.0612
LRENTTQCTYNSSLR	UL75 (gH)	-0.0532
LVFDQQGEDAVVRRC	UL71	-0.05

	TEGQVLLTMAYYLFE	UL70	0.0468
	MQLCSHSISSQRHVA	UL150 Toledo	0.0442
	TPGRGRRGGEGYRRL	UL141 Toledo	0.0426
	YVFCYVREDTAVYYL	UL24	0.0412
	WFFTYCDLLRVGYFG	UL43 rev	0.0398
	NEYRTGISWSFGMLF	UL100	0.0394
	IAVEYVLIRAVRDEI	UL93	0.038
	VGYYDEEEKRRERQK	UL32 (pp150)	-0.0378
	FRCSVTSDARKDLQK	UL43 rev	-0.0362
	LIMNVRRSWEELERK	UL32 (pp150)	-0.0304
	LLFPALCFCLLCEAV	UL116	0.0278
	PYPADLKVPTAFPQD	UL69	-0.0272
	YCDLLRVGYFGHLNI	UL43 rev	0.025
	VTCLMLFVPYYCFRV	UL78	0.0236
	NVTEVHGEVACFRND	UL37	-0.023
	TLHNLKLFCYLVSTA	UL48	0.023
	ARKHSETVLTVWMSG	UL97	-0.0176
	HEREILDLMRHSPDV	UL71	-0.0162
	FGALIIFLAYVYHYE	UL146 Toledo	0.0154
	LETLGCVKTVSLGIT	UL46	-0.0146
	ALKRAMYSVELAVCY	UL78	0.0114
	VGRVCTFYVTCLMLF	UL78	0.0108
	HREKVLYLAIACFFG	UL11	0.009
	RYLWTPDPSRLRSIN	UL14	-0.0048
Serologic group D+R+			
	SQKPVLGKRVATPHA	UL32 (pp150)	0.7497
	TTSTSQKPVLGKRVA	UL32 (pp150)	0.4908
	FGYTHPDRHPVYFFK	UL70	0.3366
	IITHAVIINYYYVAQ	UL25	0.0542
12 months after HSCT			
Serologic group D-R+			
	QLSYLMTGTVRDNVP	UL48	-0.915
	EFARVGLRAVETLHC	UL24	-0.4136
	LCYLQCCGRWCPTPG	UL141 Toledo	-0.3832
	VYAVHGLHTLMRETA	UL102	-0.3785
	VDLTFFFPVGLYLPE	UL86	-0.2676
	LDLPYPRGYTLFVCD	UL26	-0.2346
	CAPDFNMEFSSACVH	UL44 (pp50)	0.1933
	AYYLRWHACVPQKCE	US8	-0.1872
	SQLSERVAYHLKLRP	UL40	-0.156
	ANVYLCPGYLHFSAY	UL35	-0.1535
	EDAVCWLRRTAIVMR	US14	-0.1348
	LIAVSVLSSRSKESL	US 9	-0.083
	WGHKSICSFFPKLQG	UL 4 (gp48)	-0.0722
	CASYNDTFYPTNFTP	UL67	-0.0711
	VVGIFDGQHFFTYHV	UL18	-0.0442
	YALYASTPALFDFLR	UL25	-0.0407
	CRYQVFVDAYGAVFA	UL29	-0.0382

FGNNFFVRTGHMVLA	UL100	-0.029
ALLGRLYFISSKHTL	UL84	-0.0256
AQLDLEADPTAREGE	UL35	0.0207
YCDLIREKEVHRPVV	UL45	0.0081
SQKPVLGKRVATPHA	UL32 (pp150)	0.0036

Serologic group D+R-

FVGRFVNEGVLSPDQ	UL24	-0.8944
YTGRLIMNVRRSWEE	UL24	-0.7996
ATDDEWTLQKVFYLC	UL86	-0.7366
DQTDAVLFFDSPENV	UL54	-0.7312
LELEDYDRRCRCNNQ	UL147 Toledo	-0.6318
EFARVGLRAVETLHC	UL24	-0.5586
PACDDGLFLYRTTVS	UL106	-0.5524
YVATALYYVHFPFFS	US15	-0.5316
LHAETTRTWRWAQRG	TRL 1	-0.4844
RRNDVDFWLLRFQPG	UL102	-0.4392
STSTIAYRPDSSFMK	UL74 (gO)	-0.4068
GYNSKFYSPCAQYFN	UL86	-0.3946
VRRYSTVSPGKEVTL	UL148 Toledo	-0.3258
TIDDPFDECPDTHFA	UL36	0.3112
LDFYWVLPGGFAVSS	UL93	-0.304
FFFPVGLYLPEDRGY	UL86	-0.2948
NQTSTVCLLCELMAC	UL52	-0.2882
WQHNESVVDLWLYQN	UL16	-0.2512
AIKFHDLNKLTTGKM	UL86	-0.2474
WLDLGPHLLHRRLET	UL46	-0.2308
KKCNQTEKWHNVDWI	IRL14	-0.2098
SSQIRTRWEESNVVS	UL13	-0.2064
FYRAFRSGRFDLCTD	UL76	-0.1972
CAPDFNMEFSSACVH	UL44 (pp50)	0.1946
GARTRDFRCLNYTHR	UL70	-0.194
LVFDQQGEDAVVRRC	UL71	-0.1684
ELYRLPRLSIASARW	UL45	-0.1524
YWCSESYRRLNTEEE	UL14	-0.1242
AYYLRWHACVPQKCE	US 8	-0.1192
MLFFIWAMFTTCRAV	UL100	0.1172
KRYFRPLLRAWSLGL	UL24	-0.1054
FKFFHQDPNRVLDCI	UL117	-0.1054
LCRGLRRVWMTVWAS	UL70	-0.0878
VDLTFFFPVGLYLPE	UL86	-0.0818
ARRRYHLRRDYWLTD	UL14	-0.0728
TRLSYGRSIFTEHVL	UL115	-0.0694
VTKLYTSRMVTNLTV	UL16	0.0616
YADNDDYGLYVDWCV	UL104	-0.06
NTSHHSVVWQRYDIY	TRL11	-0.0562
FVGKMGTVCSQGAYV	UL36	0.05
FIALIVVCIMGWWKL	UL 7	-0.048
KNCSRTDVWHDIEWI	UL153 Towne	-0.0414

ANVYLCPGYLHFSAY	UL35	-0.0316
EIALGYRSQSVLTWT	UL18	-0.0218
FLRFERYDTDYLLRR	UL49	-0.017
FVVNDGTRYQMCVMK	UL130	-0.0142
TAFAHEYHNWLRSPF	UL86	-0.014
LAEETARFVELAGCW	UL45	-0.0126
RDVCIDYGLHRVFTQ	UL87	-0.0112
ALVARGPSLAHYVTA	UL54	0.0108
ARSKYPYHFFATSTG	UL55 (gB)	-0.0086
LCYGFGNNFFVRTGH	UL100	-0.0082
ACYTVFGLGSIHPRF	UL126	-0.007

Serologic group D+R+

PNCCQVSVDRSRVPE	UL30	1.121
RLPRPDTPRTPRQKK	UL99 (pp28)	0.8275
IRKPPWLMEQPPPPS	IRS1	0.5896
MFLGYSDCVDPGLAV	UL 5	0.4723
DDDEAALPGEDEAWI	UL31	0.4309
YLASNAVLALRIIRL	UL27	-0.4081
TMSTVGFDRVPQYDF	UL45	0.4043
VQRLLDLTQMVMRLV	UL47	-0.4003
HDSLESRRLREEEDD	UL100	0.3524
HNGIYDRVPDCPKGR	UL24	0.2523
DESGRPRRIANRIGD	US22	0.2426
TTTFFLGDMQLPADN	UL31	0.2254
ESDEEEAIVAYTLAT	UL123 (IE1)	0.2171
MESSAKRKMDPDNPD	UL122 (IE2)	0.2167
DAQEDCLYELASDLA	UL29	0.2055
FGNNFFVRTGHMVLA	UL100	-0.193
PDTPRTPRQKKISQR	UL99 (pp28)	0.1748
MEPTPMLRDRDHDDA	UL42 rev	0.1734
GALVDFDFLRLPRGG	UL76	-0.1561
VVAKRLPRPDTPRTP	UL99 (pp28)	0.1496
LSVEEICEEHTLNDL	UL25	0.1433
LRDCVYELAPTMKDF	UL36	0.1296
DLFSGDEDSDSSDGY	UL32 (pp150)	0.1136
MYCFLFLQKDTFFHE	TRL 3	-0.1127
GDRLEVACIFPAHDW	UL14	0.1062
WLTIILYVFMWTYLV	US 7	-0.1032
TPEQSTPSRIRKAKL	UL32 (pp150)	0.0961
LSDVTQRRNRPLRCL	UL35	0.0875
TKLPKYDPDEFWTKA	TRL14	0.0801
LSMDTFQLFTLTMSF	UL100	-0.0794
RNGATFSKGDIEGNF	US30	0.0698
DEPCCTPALGRYSLG	UL141 Toledo	0.0689
YPAVTTVYPPSSTAK	UL32 (pp150)	0.0682
KKTPDPMIMFDEDDD	UL36	0.0616
YQKGYNCTDKHITLS	UL 6	0.0566
MIDLTSHHRPLTLFT	UL117	-0.0461

VYYELARDLGSHGTE	UL87	0.043
SQKPVLGKRVATPHA	UL32 (pp150)	0.0385
VMVRIFSTNQGGFML	UL122 (IE2)	-0.0329
YCWTMFPPMYPVLLL	UL21	-0.0275
HGGIHVLLYGTMLVK	UL28	-0.023
YDDESWRPLSTVDDH	IRS1	0.0202
QQQQRHAAFSLVSPQ	UL32 (pp150)	0.0187
SYVVTNQYLIKGISY	UL75 (gH)	-0.0066
VVGIFDGQHFFTYHV	UL18	-0.004
FQTSATIATTILFML	US12	-0.0033
IDDDTPMLLIFGHLP	UL75 (gH)	-0.0009
24 months after HSCT		
Serologic group D-R+		
PEGLVEFEAQPGALL	UL46	-0.5929
FSGPSVPWRDEKRAC	UL66	-0.592
QFWQKVCSNALPKNV	UL57	-0.5679
CKKRYIGKVEGASGL	UL54	-0.5176
DWISKQPLRGRTRRD	IRL14	-0.4775
LFNDKCAFKLDLLRM	UL48.5 (UL48-49)	-0.4743
TTVMTERQSQLPEKY	IRS1	-0.4727
ERFAAAAKPLPSLCV	UL95	-0.3688
NNRLAEGIIRATELV	TRL 2	-0.3683
SICFGVPGETGGGCF	UL57	0.2966
SAYHRLRMSNIPRSS	UL88	-0.283
AKLSSPMTTTSTSQK	UL32 (pp150)	-0.2397
ILQKDTFIERTPCEQ	UL105	-0.2374
PPEPLREYLADLLYL	UL98	0.2323
AMLTACVEVWARELL	UL102	-0.2056
HDVLERFAAAAKPLP	UL95	-0.1997
QLETLSRPDEPCCTP	UL141 Toledo	0.1959
QDKVVSYARDELTKR	UL34	-0.185
ILTRLEYLYKVDSQR	UL54	-0.1842
EFEGDFARYRSSQQK	UL48	-0.1768
QTEKWHNVDWISKQP	IRL14	-0.1721
LKDALGRQVSLRSYD	UL99 (pp28)	0.1663
SQKPVLGKRVATPHA	UL32 (pp150)	0.1604
IVAAALWKVDYDRSV	UL121	-0.1568
YLVLPNCCQVSVDRS	UL30	-0.1564
TVYPTYDCVLSDLEA	UL77	0.1525
ALLTLLSSDTAPRWM	UL115	0.1501
PGEPLKDALGRQVSL	UL99 (pp28)	0.1357
HLFVTDKRFLNRELG	UL49	-0.1312
FGTTPGEPLKDALGR	UL99 (pp28)	0.1228
GTHKYVLERDDEAVL	UL102	-0.0988
SPSMGFQRTFIDPLW	UL74 (gO)	-0.0965
PELLTGPPAPNLPGP	UL61	-0.0866
PLLRHLDKYYAGLPP	UL115	-0.0814
FETSGGLVVFWQGIK	UL55 (gB)	-0.0743

YNECGVELPGGDSSD	US22	-0.0691
ARHVGEFNVLKVNES	UL50	-0.0686
VPLNPHYGKSYDNDD	UL69	-0.0616
SLKPTLGGKAVVGRP	UL32 (pp150)	0.0608
MFDSGVDRDYARQFR	US23	-0.0587
TRPFKVIIKPPVPPA	UL122 (IE2)	0.0574
TERQSQLPEKYIGFY	IRS1	-0.0542
LCAPVVRACRASSFG	J1I	-0.0502
LEFTANNRVSFHGVK	UL44 (pp50)	-0.0385
NITLKNAIRLRNGTM	TRL13	-0.0293
TSPNALLPEWMDAVH	UL136 Toledo	0.025
TVQTARDPLYAAEQL	UL47	-0.0238
AIVYSNYTVERVTLP	UL104	-0.0213
FETGGDVGREFMLAR	UL43 rev	-0.0166
YVTQNGTLPTTTTKK	UL11	-0.0052
LLEEGDEEDEVTVMS	UL71	-0.0025
LSNEGVLPNPRKPQA	UL48	-0.0004
Serologic group D+R-		
LCAPVVRACRASSFG	J1I	-1.6143
HNPAVFTWPPWQAGI	UL83 (pp65)	-0.8739
FETSGGLVVFWQGIK	UL55 (gB)	-0.4401
HDSLESRRLREEEDD	UL100	0.2181
ESPVPATIPLSSVIV	UL123 (IE1)	0.0481
EIDLEHCQNDFFGEF	UL29	-0.027
Serologic group D+R+		
ANGMPPLTPPHVYMN	UL36	0.8028
ASELRPGSGGWPEHA	US 1	-0.6582
EIDLEHCQNDFFGEF	UL29	-0.4846
TNQYLIKGISYPVST	UL75 (gH)	0.4478
TGMKTVAFDLSSPQK	UL32 (pp150)	0.4418
LICKNPNYSVCDAML	UL50	0.4228
ERDWRRVIHDSHGLW	US32	0.4154
AFIRRRRPPHHTQLV	J1I	0.333
DEPCCTPALGRYSLG	UL141 Toledo	0.3114
RSAHFRVEENQCWFH	US 3	-0.3086
HNPAVFTWPPWQAGI	UL83 (pp65)	-0.2832
ERFVCPVYDSGTPMG	US10	0.2818
YVDPHYPGWGRRYEP	UL80	0.2346
DYVLKFLTRLAEAAT	UL86	0.2286
PLFIVPDAYREHPLG	UL70	0.2188
LLKHMIGISIGYVAH	UL89	0.1648
CCSACYKETMIYDMV	UL133 Toledo	0.1374
YVTQNGTLPTTTTKK	UL11	-0.0936
FAALQEQGVEDFSLE	UL57	0.0908
PRHTFDMDMMEMPAT	UL42 rev	0.0886
TFYKCLDAQFVCMPE	UL72	0.0746
CGHCLNLGKEKLHCQ	UL49	-0.0706
ERTIRSEAEDSYHFS	UL55 (gB)	-0.064

RWKDNKQYGQVFMTD	UL 8	0.0636
GMRAVSQFLVTHPLG	US33	0.0354
GGEEGEDDVLATIRN	UL150 Toledo	-0.0124
MAQRNGMSPRPPPLG	IRS1	0.0106
LQLDRLVFEAAQRGL	UL87	0.0104
SVMLAKRPLITKPEV	UL123 (IE1)	0.0014

The peptides listed here are detectable in serum from individuals in the respective group (D-R+, D+R- or D+R+) and differentially recognized in the D-R- group at all time points post-HSCT. The average Q-value is the absolute difference between the Q-score for D-R- and the Q-score for each respective group. Higher average Q-value indicates the probability that the peptide is differently recognized between the respective group and the D-R- (reference) group.
