# Supplementary material for: Whole CMV Proteome Pattern Recognition Analysis after HSCT Identifies Unique Epitope Targets Associated with the CMV Status
Source: PLoS One. 2014 Apr 16;9(4):e89648. doi: 10.1371/journal.pone.0089648 (PMC3989190; doi:10.1371/journal.pone.0089648)

Supplementary Table S5. MaSigPro analysis.

I) Significantly recognized peptides for the 3 groups vs. D-R- over time (number of peptides corresponding to the total numbers of peptides in the Venn diagram circles in Figure 4 A).

	D-R+ vs D-R-	D+R- vs D-R-	D+R+ vs D-R-
1	NGVWVVVFLVNVLIV	NGVWVVVFLVNVLIV	NGVWVVVFLVNVLIV
2	LSTPLQLGEANDESQ	TEPTTLPIVSVSELA	LSTPLQLGEANDESQ
3	LERNVDLTFFFPVGL	FNTNRVINMKAALSS	LERNVDLTFFFPVGL
4	LAVVFTVVINRDSAN	AGGSPLHGVVGGFAA	FNTNRVINMKAALSS
5	SWRPLSTVDDHKAWL	LFEDRLLAYGVLAFL	LFEDRLLAYGVLAFL
6	AGGRWRFEDGGAAQR	TQIYNGPCLGTEARL	TQIYNGPCLGTEARL
7	PNCCQVSVDRSRVPE	LAVVFTVVINRDSAN	LAVVFTVVINRDSAN
8	PEGLVEFEAQPGALL	VYAIFIFQLAFSFGL	VYAIFIFQLAFSFGL
9	GSLFWHQNQRDFPKC	SWRPLSTVDDHKAWL	RDDDEDNHVVPDNQN
10	VMNFIITTRDFSNDD	PEGLVEFEAQPGALL	GLYASENYNGNYELT
11	LLLCRLPFLLLFQRP	YLMGRDKALAVEQFI	AGGRWRFEDGGAAQR
12	DRDHDDAPPTYEQAM	NFSSVRVLVMEACVF	PNCCQVSVDRSRVPE
13	AALRQFVHDSQQSVK	PIFVSKKGQISIFLT	PEGLVEFEAQPGALL
14	PGGRAGGEEEGYGGR	GSLFWHQNQRDFPKC	YLMGRDKALAVEQFI
15	QSQAVQIGFLHTQLV	VMNFIITTRDFSNDD	PIFVSKKGQISIFLT
16	GGGAICVPNADAHAV	DLFRHHVLTVFDFHL	VMNFIITTRDFSNDD
17	CDRCVRRRRFKVCDV	KRQVLYFKLQVSYGK	KRQVLYFKLQVSYGK
18	NCQFLAVGPDDEVAH	YLDPAFTTNRKASGT	YLDPAFTTNRKASGT
19	IIMAHNLCYSTLLVP	KLSRLSVPTLIMVRV	LLLCRLPFLLLFQRP
20	LPHLFRTPNLWLPTT	VRACLNPGIYILVGT	KLSRLSVPTLIMVRV
21	MDPPLPSLHSPQWAS	QRIILALTVSFIYGL	VRACLNPGIYILVGT
22	IPLLLIVTPVVFDPQ	KVFLSLVFTLLMVLR	PRNVMTHEEAESRLY
23	VSATCDLDLEVDDAV	AALRQFVHDSQQSVK	WLPRSWLELTVLVSD
24	HDSLESRRLREEEDD	PGGRAGGEEEGYGGR	DRDHDDAPPTYEQAM
25	DDNSDGDATITINAS	VVLAAAAAQAAAASQ	AALRQFVHDSQQSVK
26	PKPLETTTASNVTTI	SLDRSYEEVKAAVQQ	PGGRAGGEEEGYGGR
27	AKTMEMRFTIAWMWF	YKISPVSLVLQTKAH	YKISPVSLVLQTKAH
28	MLWILVLFALAASAS	LVDSVSATCDLDLEV	LVDSVSATCDLDLEV
29	TSPHGLGLAGYGGRI	PDAVLDDVVAAIGSQ	RVDDTAVVTAGDPRS
30	ITLLVLFIVYVTVDC	AQWRQQVHAAHDVWC	AQWRQQVHAAHDVWC
31	DECGLMLRYMLQVVV	QSQAVQIGFLHTQLV	QSQAVQIGFLHTQLV
32	YGSGCRFDTVEMVDE	LHKTLLCLYTVFVGC	CDRCVRRRRFKVCDV
33		CHVLRQAGVTGIYKH	NCQFLAVGPDDEVAH
34		LPHLFRTPNLWLPTT	IIMAHNLCYSTLLVP
35		IPLLLIVTPVVFDPQ	MDPPLPSLHSPQWAS
36		ERFVCPVYDSGTPMG	ERFVCPVYDSGTPMG

37	PHYAVPFTTPGKPGC	PHYAVPFTTPGKPGC
38	ILREKTQLLTAIVSK	KFHQGIAQLKRAPAE
39	AATDSILDNPTTYTT	AATDSILDNPTTYTT
40	VSATCDLDLEVDDAV	HDSLESRRLREEEDD
41	ETTIQRLQINDLLAY	WMLAFLEEALAQITA
42	WSLKELHTHQLCPRS	MNLVMLILALWAPVA
43	MNLVMLILALWAPVA	DDNSDGDATITINAS
44	PCTVMTHSWPMVSIR	PCTVMTHSWPMVSIR
45	DDNSSADNIAASGAA	PKPLETTTASNVTTI
46	YVLFRRDTDTELDTV	AKTMEMRFTIAWMWF
47	PAGLYRPEEEVFLLL	DDNSSADNIAASGAA
48	MLWILVLFALAASAS	PAGLYRPEEEVFLLL
49	TSPHGLGLAGYGGRI	LMTGTVRDNVPLLQE
50	VSNMTNHTLNSTEIY	TMCNLALSTPFLMEH
51	LTFVPSGFVMGHVAI	MLWILVLFALAASAS
52	ITLLVLFIVYVTVDC	TSPHGLGLAGYGGRI
53	DECGLMLRYMLQVVV	VSNMTNHTLNSTEIY
54	YGSGCRFDTVEMVDE	LTFVPSGFVMGHVAI
55		ITLLVLFIVYVTVDC
56		DECGLMLRYMLQVVV
57		YGSGCRFDTVEMVDE

II) Peptides from the comparison of the 3 groups vs D-R- (see previous table) clustered by their "recognition profiles" (intensity values across all the samples).

a) D-R+ vs. D-R-

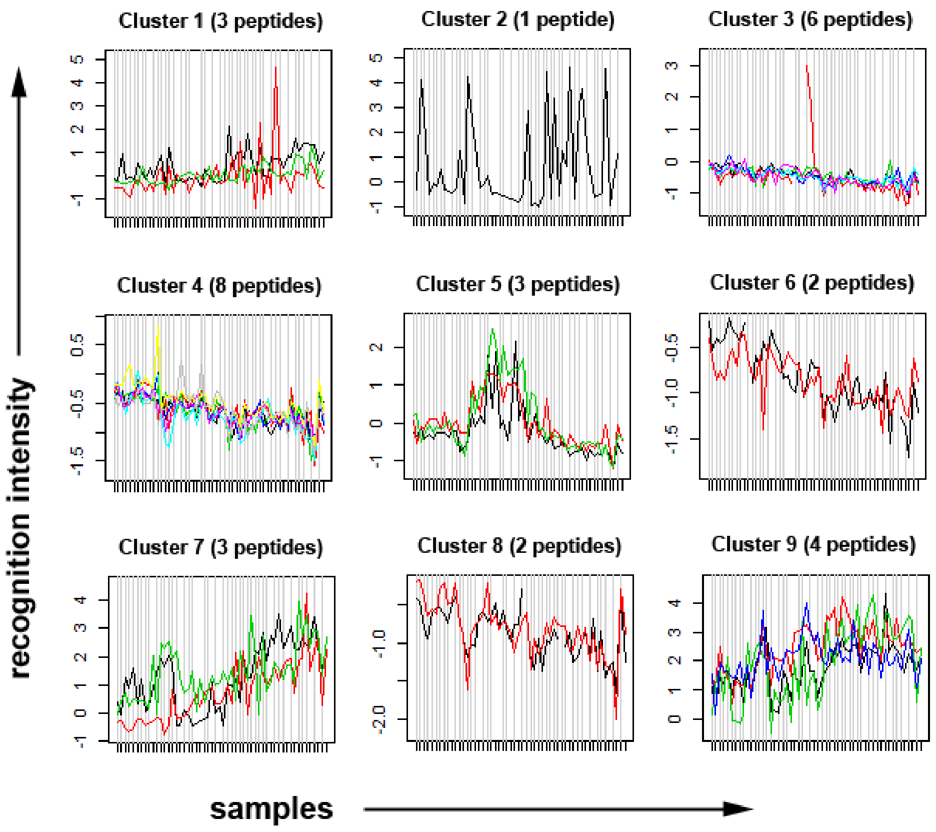

Peptides list by cluster number

	cluster n.	protein
PNCCQVSVDRSRVPE	1	CAA35429
PEGLVEFEAQPGALL	1	CAA35405
LPHLFRTPNLWLPTT	1	AAA85892.1
HDSLESRRLREEEDD	2	CAA35336
LERNVDLTFFFPVGL	3	CAA35360
AALRQFVHDSQQSVK	3	CAA35372
QSQAVQIGFLHTQLV	3	CAA35430
GGGAICVPNADAHAV	3	CAA35311
CDRCVRRRRFKVCDV	3	CAA35395
DECGLMLRYMLQVVV	3	CAA35340
NGVWVVVFLVNVLIV	4	CAA35283
LSTPLQLGEANDESQ	4	CAA35358
LAVVFTVVINRDSAN	4	CAA74074

VMNFIITTRDFSNDD	4	CAA35393
LLLCRLPFLLLFQRP	4	CAA35445
NCQFLAVGPDDEVAH	4	CAA35404
PKPLETTTASNVTTI	4	CAA35440
MLWILVLFALAASAS	4	AAA85878.1
IIMAHNLCYSTLLVP	5	CAA35413
TSPHGLGLAGYGGRI	5	CAA35386
ITLLVLFIVYVTVDC	5	CAA35313
VSATCDLDLEVDDAV	6	CAA35370
DDNSDGDATITINAS	6	CAA35334
GSLFWHQNQRDFPKC	7	CAA35328
MDPPLPSLHSPQWAS	7	CAA35280
YGSGCRFDTVEMVDE	7	AAA85875.1
PGGRAGGEEEGYGGR	8	CAA35392
IPLLLIVTPVVFDPQ	8	CAA35334
SWRPLSTVDDHKAWL	9	CAA35311
AGGRWRFEDGGAAQR	9	CAA35447
DRDHDDAPPTYEQAM	9	CAA74074
AKTMEMRFTIAWMWF	9	CAA35262

Group averages of recognition levels by time points and cluster. (red= D-R-, green=D-R+, blue=D+R-, cyan= D+R+).

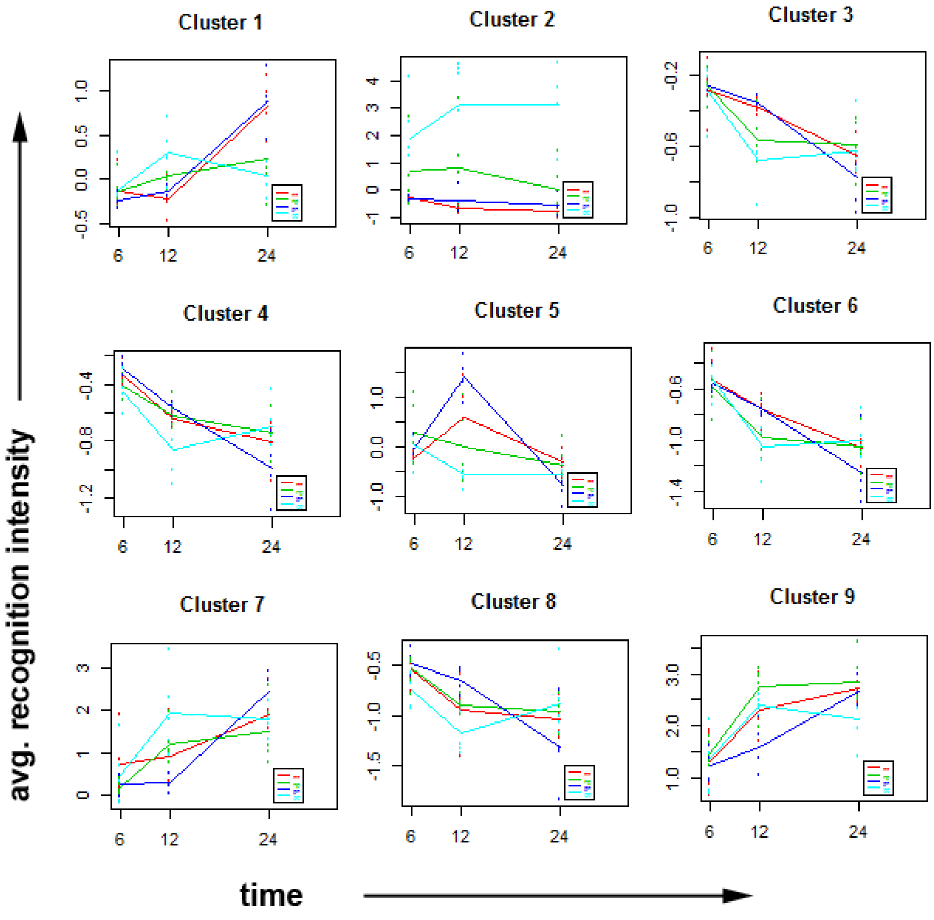

b) D+R- vs. D-R-

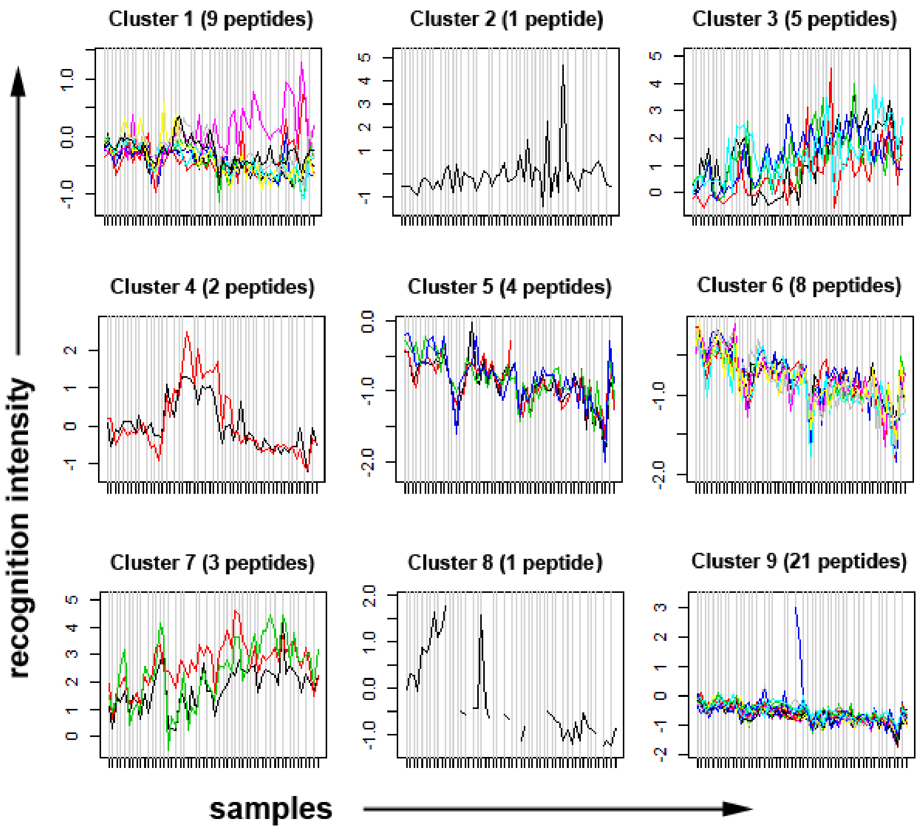

Peptide list by cluster number

	cluster n.	protein
AGGSPLHGVVGGFAA	1	CAA35338
NFSSVRVLVMEACVF	1	CAA35344
VRACLNPGIYILVGT	1	CAA35259
QSQAVQIGFLHTQLV	1	CAA35430
CHVLRQAGVTGIYKH	1	CAA35411
LPHLFRTPNLWLPTT	1	AAA85892.1
YVLFRRDTDTELDTV	1	CAA35406
VSNMTNHTLNSTEIY	1	CAA35259
LTFVPSGFVMGHVAI	1	CAA35266
PEGLVEFEAQPGALL	2	CAA35405
GSLFWHQNQRDFPKC	3	CAA35328
ERFVCPVYDSGTPMG	3	CAA35277
PHYAVPFTTPGKPGC	3	CAA35430
PCTVMTHSWPMVSIR	3	AAA85880.1
YGSGCRFDTVEMVDE	3	AAA85875.1
TSPHGLGLAGYGGRI	4	CAA35386
ITLLVLFIVYVTVDC	4	CAA35313
PIFVSKKGQISIFLT	5	CAA35344
PGGRAGGEEEGYGGR	5	CAA35392

YKISPVSLVLQTKAH	5	CAA35360
IPLLLIVTPVVFDPQ	5	CAA35334
TEPTTLPIVSVSELA	6	AAA85874.1
TQIYNGPCLGTEARL	6	CAA35396
DLFRHHVLTVFDFHL	6	CAA35411
KLSRLSVPTLIMVRV	6	CAA35372
LVDSVSATCDLDLEV	6	CAA35370
ILREKTQLLTAIVSK	6	CAA35410
AATDSILDNPTTYTT	6	CAA35360
VSATCDLDLEVDDAV	6	CAA35370
SWRPLSTVDDHKAWL	7	CAA35311
AQWRQQVHAAHDVWC	7	CAA35312
WSLKELHTHQLCPRS	7	Mocarski
VVLAAAAAQAAAASQ	8	CAA35354
NGVWVVVFLVNVLIV	9	CAA35283
FNTNRVINMKAALSS	9	CAA35361
LFEDRLLAYGVLAFL	9	CAA35320
LAVVFTVVINRDSAN	9	CAA74074
VYAIFIFQLAFSFGL	9	P09724
YLMGRDKALAVEQFI	9	CAA35363
VMNFIITTRDFSNDD	9	CAA35393
KRQVLYFKLQVSYGK	9	CAA35361
YLDPAFTTNRKASGT	9	CAA35363
QRIILALTVSFIYGL	9	CAA35279
KVFLSLVFTLLMVLR	9	CAA35282
AALRQFVHDSQQSVK	9	CAA35372
SLDRSYEEVKAAVQQ	9	CAA35359
PDAVLDDVVAAIGSQ	9	CAA35331
LHKTLLCLYTVFVGC	9	CAA35281
ETTIQRLQINDLLAY	9	CAA35340
MNLVMLILALWAPVA	9	CAA35278
DDNSSADNIAASGAA	9	CAA35407
PAGLYRPEEEVFLLL	9	CAA35265
MLWILVLFALAASAS	9	AAA85878.1
DECGLMLRYMLQVVV	9	CAA35340

Group averages of recognition levels by time points and cluster. (red= D-R-, green=D-R+, blue=D+R-, cyan= D+R+).

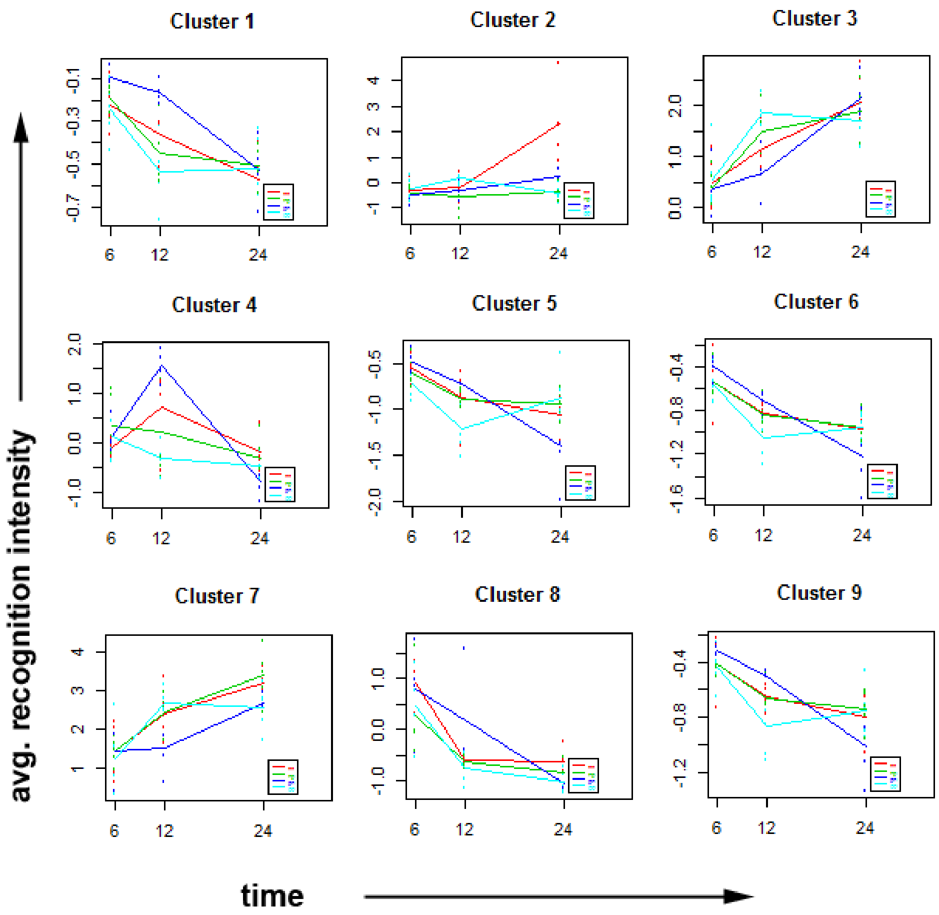

c) D+R+ vs. D-R-

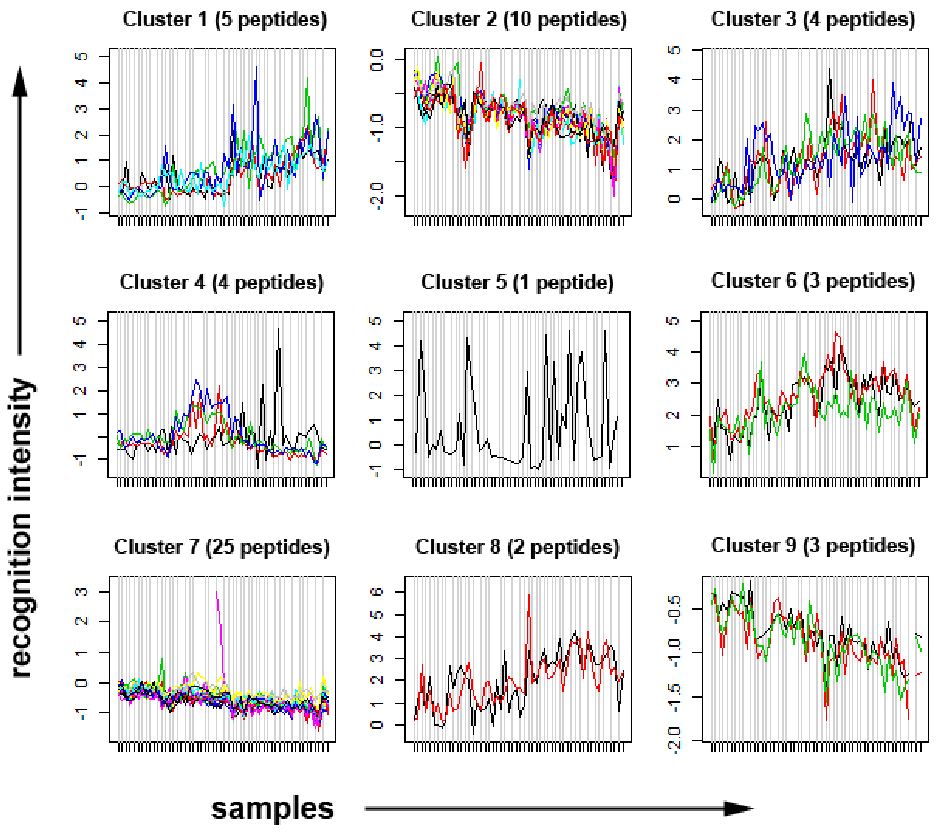

Peptide list by cluster number

	cluster n.	protein
PNCCQVSVDRSRVPE	1	CAA35429
PRNVMTHEEAESRLY	1	CAA35395
MDPPLPSLHSPQWAS	1	CAA35280
ERFVCPVYDSGTPMG	1	CAA35277
KFHQGIAQLKRAPAE	1	CAA35393
TQIYNGPCLGTEARL	2	CAA35396
PIFVSKKGQISIFLT	2	CAA35344
LLLCRLPFLLLFQRP	2	CAA35445
KLSRLSVPTLIMVRV	2	CAA35372
PGGRAGGEEEGYGGR	2	CAA35392
YKISPVSLVLQTKAH	2	CAA35360
AATDSILDNPTTYTT	2	CAA35360
MNLVMLILALWAPVA	2	CAA35278
DDNSDGDATITINAS	2	CAA35334
LMTGTVRDNVPLLQE	2	CAA35407
GLYASENYNGNYELT	3	CAA35441
PHYAVPFTTPGKPGC	3	CAA35430
PCTVMTHSWPMVSIR	3	AAA85880.1
YGSGCRFDTVEMVDE	3	AAA85875.1

PEGLVEFEAQPGALL	4	CAA35405
IIMAHNLCYSTLLVP	4	CAA35413
TSPHGLGLAGYGGRI	4	CAA35386
ITLLVLFIVYVTVDC	4	CAA35313
HDSLESRRLREEEDD	5	CAA35336
AGGRWRFEDGGAAQR	6	CAA35447
AQWRQQVHAAHDVWC	6	CAA35312
AKTMEMRFTIAWMWF	6	CAA35262
NGVWVVVFLVNVLIV	7	CAA35283
LSTPLQLGEANDESQ	7	CAA35358
LERNVDLTFFFPVGL	7	CAA35360
FNTNRVINMKAALSS	7	CAA35361
LFEDRLLAYGVLAFL	7	CAA35320
LAVVFTVVINRDSAN	7	CAA74074
VYAIFIFQLAFSFGL	7	P09724
RDDDEDNHVVPDNQN	7	CAA35290
YLMGRDKALAVEQFI	7	CAA35363
VMNFIITTRDFSNDD	7	CAA35393
KRQVLYFKLQVSYGK	7	CAA35361
YLDPAFTTNRKASGT	7	CAA35363
VRACLNPGIYILVGT	7	CAA35259
AALRQFVHDSQQSVK	7	CAA35372
QSQAVQIGFLHTQLV	7	CAA35430
CDRCVRRRRFKVCDV	7	CAA35395
NCQFLAVGPDDEVAH	7	CAA35404
WMLAFLEEALAQITA	7	CAA35407
PKPLETTTASNVTTI	7	CAA35440
DDNSSADNIAASGAA	7	CAA35407
PAGLYRPEEEVFLLL	7	CAA35265
MLWILVLFALAASAS	7	AAA85878.1
VSNMTNHTLNSTEIY	7	CAA35259
LTFVPSGFVMGHVAI	7	CAA35266
DECGLMLRYMLQVVV	7	CAA35340
DRDHDDAPPTYEQAM	8	CAA74074
TMCNLALSTPFLMEH	8	Mocarski
WLPRSWLELTVLVSD	9	CAA35358
LVDSVSATCDLDLEV	9	CAA35370
RVDDTAVVTAGDPRS	9	AAA85891.1

Group averages of recognition levels by time points and cluster. (red= D-R-, green=D-R+, blue=D+R-, cyan= D+R+).

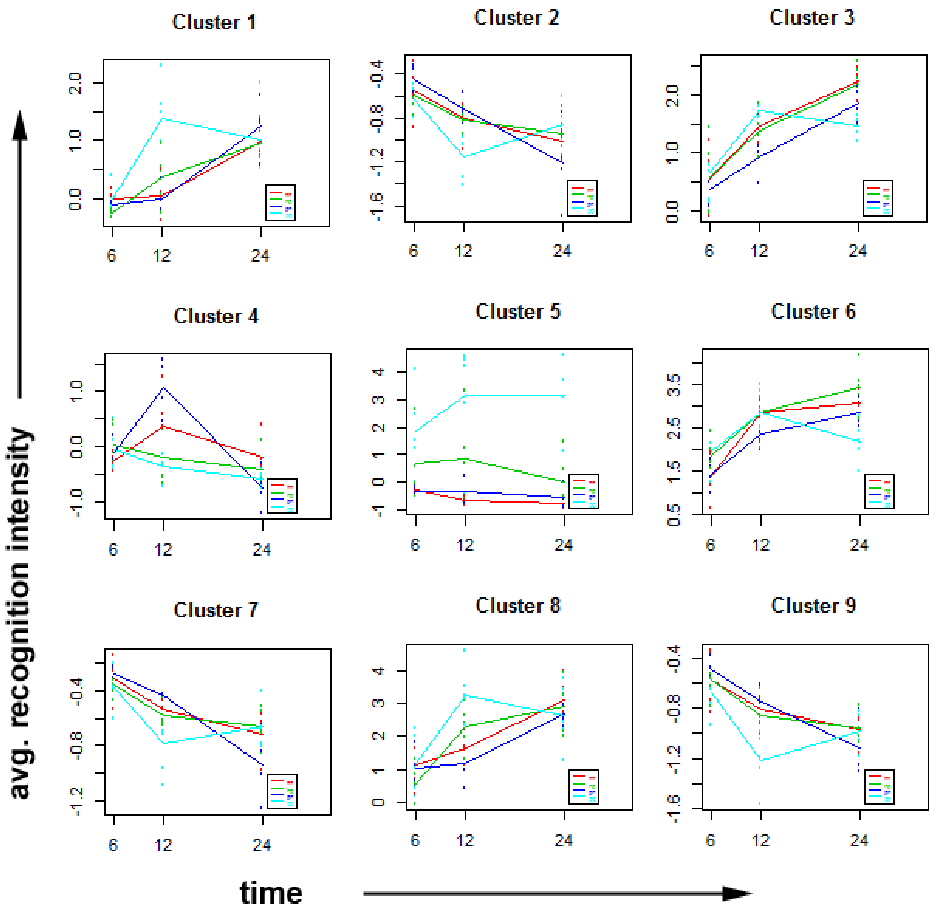

Supplement: Table S5 — MaSigPro analysis. (PDF) [file pone.0089648.s009.pdf]
